# Thiol-Yne click chemistry of acetylene-enabled macrocyclization

**DOI:** 10.1038/s41467-022-32723-0

**Published:** 2022-08-25

**Authors:** Shiwei Lü, Zipeng Wang, Shifa Zhu

**Affiliations:** Key Laboratory of Functional Molecular Engineering of Guangdong Province, School of Chemistry and Chemical Engineering, South China University of Technology, Guangzhou, China

**Keywords:** Synthetic chemistry methodology, Photocatalysis

## Abstract

Macrocycles have fascinated scientists for over half a century due to their aesthetically appealing structures and broad utilities in chemical, material, and biological research. However, the efficient preparation of macrocycles remains an ongoing research challenge in organic synthesis because of the high entropic penalty involved in the ring-closing process. Herein we report a photocatalyzed thiol-yne click reaction to forge diverse sulfur-containing macrocycles (up to 35-membered ring) and linear C2-linked 1,2-(S-S/S-P/S-N) functionalized molecules, starting from the simplest alkyne, acetylene. Preliminary mechanistic experiments support a visible light-mediated radical-polar crossover dihydrothiolation process. This operationally straightforward reaction is also amenable to the synthesis of organometallic complexes, bis-sulfoxide ligand and a pleuromutilin antibiotic drug Tiamulin, which provides a practical route to synthesize highly valued compounds from the feedstock acetylene gas.

## Abstract (web summary)

Thiol–yne coupling is a reliable method to link two molecular units, but has not been extensively explored for the construction of macrocycles. Here, the authors use gaseous acetylene, the simplest alkyne unit, to synthesize a variety of macrocycles under photocatalytic conditions.

## Introduction

**Fig. 1:**
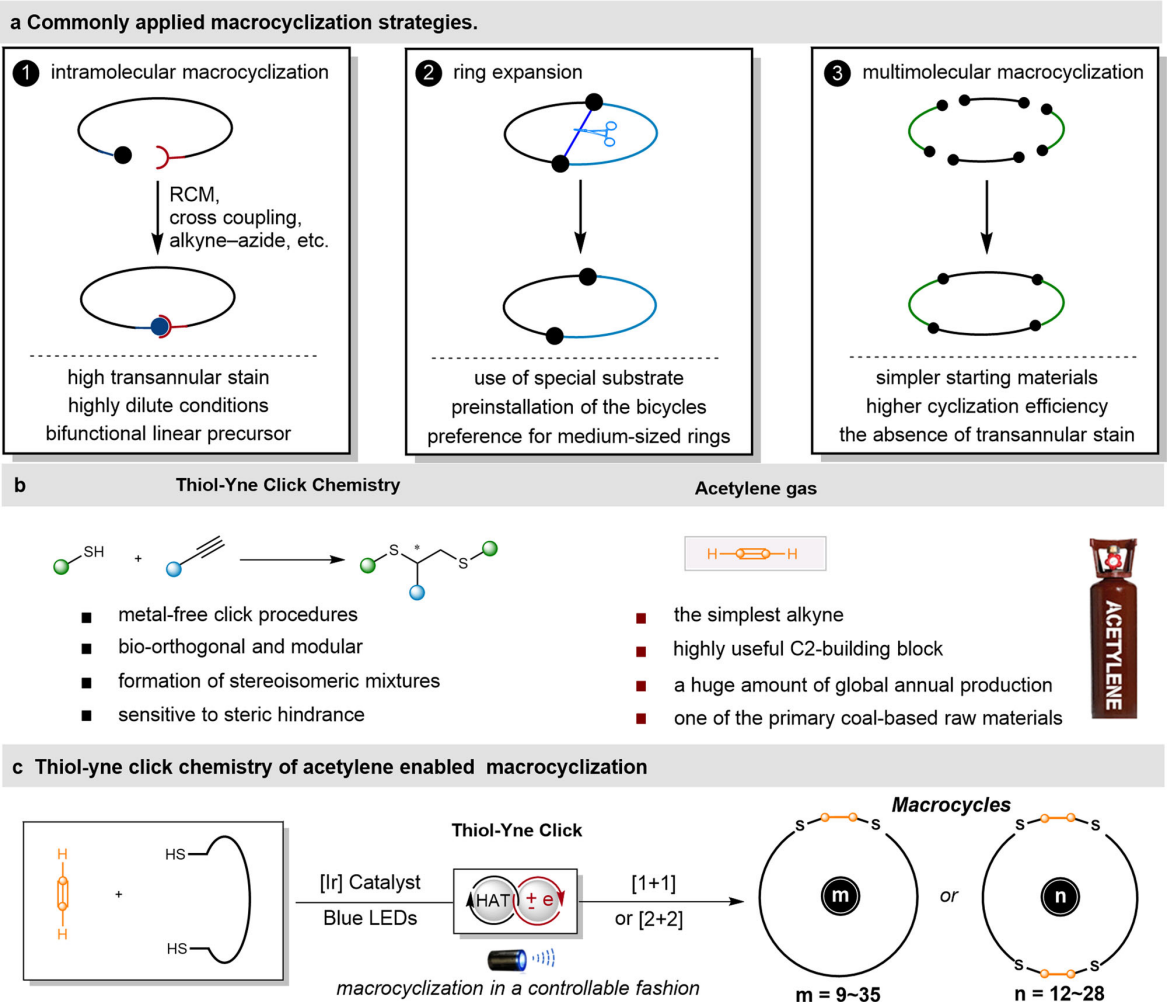
Thiol-yne click chemistry of acetylene that enabled macrocyclization. **a** A general illustration of the three most commonly applied macrocyclization strategies. **b** The characteristics of the thiol-yne click chemistry and acetylene gas. **c** Photoredox catalysis thiol-yne click reaction that enable macrocyclization (this work).

Macrocycles, by virtue of their intrinsic three-dimensional conformation and widespread occurrence in nature, play a pivotal role in multidisciplinary fields spanning from active pharmaceutical ingredients, natural products^1^ to functional materials^2^. However, the efficient preparation of macrocycles is a longstanding challenge because of the high entropic penalty during the ring-closing process^3,4^. According to the cyclization processes, the macrocyclization strategies can be generally classified into three categories^5^, including unimolecular macrocyclization, ring expansion, and multimolecular macrocyclization (Fig. 1a). Among them, the unimolecular macrocyclization has revolutionized as the most commonly used strategy. For example, the ring closing metathesis (RCM), cross coupling, copper-catalyzed azide-alkyne cycloaddition (CuAAC), etc have been extensively used to forge the macrocycles. However, these toolkits are often bias for specific procedures, such as template-induced preorganization^6,7^, highly dilute conditions^8^ and multistep synthesis of the bifunctional precursor^9^. Furthermore, the cyclization efficiency is significantly impacted by the transannular strain, bond angle and length deformations and unfavorable eclipsed conformations, when tying the two “ends” of a molecule together during the ring closure process^10^. To obviate these problems, ring expansion of a fused bicycle precursor was developed for the synthesis of macrocycles. Nevertheless, the preinstallation of the well-designed bicycles was required, which therefore greatly limited the application of this strategy^11^. As an emerging strategy, the multimolecular macrocyclization approach has received great attentions, owing to the advantages such as the absence of transannular strain, high cyclization efficiency and simple starting materials^12^. For example, Ugi multimolecular reactions were utilized for the one-pot synthesis of peptoid-based cryptands, cages, and cryptophanes^13^. The Yudin’s aziridine–aldehyde reaction could realize the diastereoselective condensation among amphoteric substrate, oligopeptides, and isocyanide to produce aziridine-containing cyclopeptides^14^. Nonetheless, the assembly of multicomponet molecules in a well-controlled sequence to form the entropy unfavorable macrocycles is still a highly challenging process, especially not overemphasizing the use of protected oligopeptides reagents^12^.

Radical thiol–yne coupling^15^ is a metal-free click reaction, which could date back to more than eighty years ago (Fig. 1b left). This reaction has emerged as an attractive synthetic tool for the synthesis of diverse functional materials and polymer settings^15–17^, such as network structures, hyperbranched polymers, and dendrimers. Much progress on the synthesis of branched and linear molecules has been made, but however, the utilization of radical thiol-yne click reaction as a reliable strategy for macrocyclization from simple alkynes and dithiols has rarely been reported^6,18,19^. This is mainly due to the inherent properties of thiol-yne click chemistry. Firstly, regio- and stereoselective thiol-yne coupling is rather challenged, and so is in macrocyclization, which gives rise to the mixtures of regio- and stereoisomers^18,19^. Secondly, the addition of thiyl radicals to the π-bond is sensitive to the steric hindrance. The reaction often stops after the first addition and therefore results in the formation of E/Z mixtures of vinyl thioethers^18^. A stepwise strategy or/and higher energetic UV-light irradiation (rather than mild visible-light irradiation) was usually adopted to enable the second addition to take place^17^. As such, direct use of mild visible light mediated thiol–yne click reaction in one pot manner would clearly be advantageous^20,21^, which is due to the extremely mild conditions^22^ and the standardization of reaction setups^23^.

Acetylene is one of the primary coal-based feedstocks with a huge amount of global annual production (Fig. 1a, right)^24^. As the simplest alkyne and also a unique C2-building block with versatile reactivity, acetylene has been used to access a broad range of valuable products in the first half of the 20th century and has had a paramount impact on chemical industry over the last few decades^25^. However, in contrast to the substituted alkynes^26^, the directly utilization of the dangerous acetylene gas in organic synthesis has still remained largely unexplored due to the special properties^27–29^. In consideration of the small size and diterminal properties^30^, we reasoned that acetylene as alkyne moiety in radical thiol-yne macrocyclization reaction would not only obviate the inherent steric hindrance and stereochemistry issues, but also provide new avenues for the direct transformation of gaseous acetylene into value-added commodity chemicals. However, the application of acetylene in the radical multimolecular macrocyclization might encounter the following potential challenges: (1) the diminished rate of the addition of thiyl radical to the unactivated diterminal acetylene when compared with its homologs^31,32^; (2) acetylene may suffer from polymerization under radical system (i.e. linear oligomer and cyclic oligomer)^33–35^; (3) the safety issues related to the manipulation of gaseous acetylene^36^.

Herein we report the visible light-mediated thiol-acetylene click chemistry-enabled multimolecular reaction for the synthesis of diverse sulfuric macrocycles (up to 35-membered ring) and the synthesis of linear C2-linked 1,2-(S-S/S-P/S-N) functionalized molecules in a one-pot manner. Preliminary mechanistic experiments support a visible light-mediated radical-polar crossover dihydrothiolation process. Meanwhile, the merit of this strategy as a synthetic platform has been demonstrated by the modification of several axial and spiro chiral skeletons as well as the synthesis of organometallic complexes, bis-sulfoxide ligand and pleuromutilin antibiotic drug tiamulin, providing a new avenue for the value-added functionalization of the feedstock acetylene.

## Results and discussion

### Reaction optimization

To investigate the photocatalyzed thiol-yne click reaction-based multimolecular macrocyclization, linear diester-tethered dithiol **1** and gaseous acetylene (in balloon) were initially applied as the model substrates for the synthesis of 19-membered sulfuric macrocycle **3**. After systematic screening various reaction parameters (Table 1, entries 1–13 and see Supplementary Tables 1–7), the optimal reaction conditions were established by using [Ir(d(*t*Bu)(CF_3_)ppy)_2_(dtbbpy)][Cl] (**PC1**) as a visible-light photocatalyst and *N,N*-diisopropylethylamine as a base in the presence of H_2_O as hydrogen donor in a solution of DMF at ambient temperature (Table 1, entry 1). Interestingly, under the conventional radical-initiation conditions (AIBN, peroxides and ultraviolet-light irradiation), the reaction resulted in multiple products, among which the thiyl radical homocoupling product of disulfide dominated (entries 9–10). Significantly, the reaction yields of macrocycle **3** were found to be independent on the dithiol concentration in the range of 0.05–0.1 M, which can’t be achieved in the traditional macrocyclization strategy. Finally, control experiments verified that photocatalyst, blue lights, and base were all necessary for the effective implementation of this reaction (entries 11–13). It is worthy to note that, in most cases, substantial amounts of yellow and unidentified insoluble precipitates could often be observed. These unidentified precipitates might be the linear oligomers or polymers formed from the similar thiol-acetylene click reaction. Furthermore, trace amount of vinyl thioether from mono hydrothiolation of acetylene (less than 5% based on ^1^H-NMR analysis of the crude reaction mixture) were also observed as the side product of this reaction.

**Table 1:** Optimization of the reaction conditions^a^


Entry	Deviation from standard conditions	Yield of 3 (%)^b^
1	none	29 (28)^c^
2	[Ir(bpy)_2_(dtbbpy)][PF_6_] instead of **PC1**	23
3	*fac*-Ir(ppy)_3_ instead of **PC1**	n.d.
4	MesAcr^+^ClO_4_^-^ instead of **PC1**	17
5	TPT^+^ instead of **PC1**	n.d.
6	DPZ instead of **PC1**	n.d.
7	Eosin Y instead of **PC1**	18
8	1.0 eq. of Hantzsch ester instead of water	trace
9	1.0 eq. of cyclohexadiene instead of water	n.r.
10	AIBN under 80 ^o^C instead of blue light and **PC1**	14
11	UV-light irradiation instead of blue light and **PC1**	trace
12	No light (dark, 48 h)	n.r.
13	No **PC1**	trace
14	No base	n.r.


^a^Standard reaction conditions: **1** (0.1 mmol), a balloon was filled with acetylene gas until its size was roughly 5 cm in diameter, [Ir(d(*t*Bu)(CF_3_)ppy)_2_(dtbbpy)][Cl] (1 mol%), DIPEA (1.5 equiv), DMF/H_2_O (50:1, 1 mL), r.t., 12 h. ^b^Yield of crude product by ^1^H NMR using 1,3,5-trimethoxylbenzene as internal standard. ^c^Isolated yield is shown in parentheses.

### Substrate scope

**Fig. 2:**
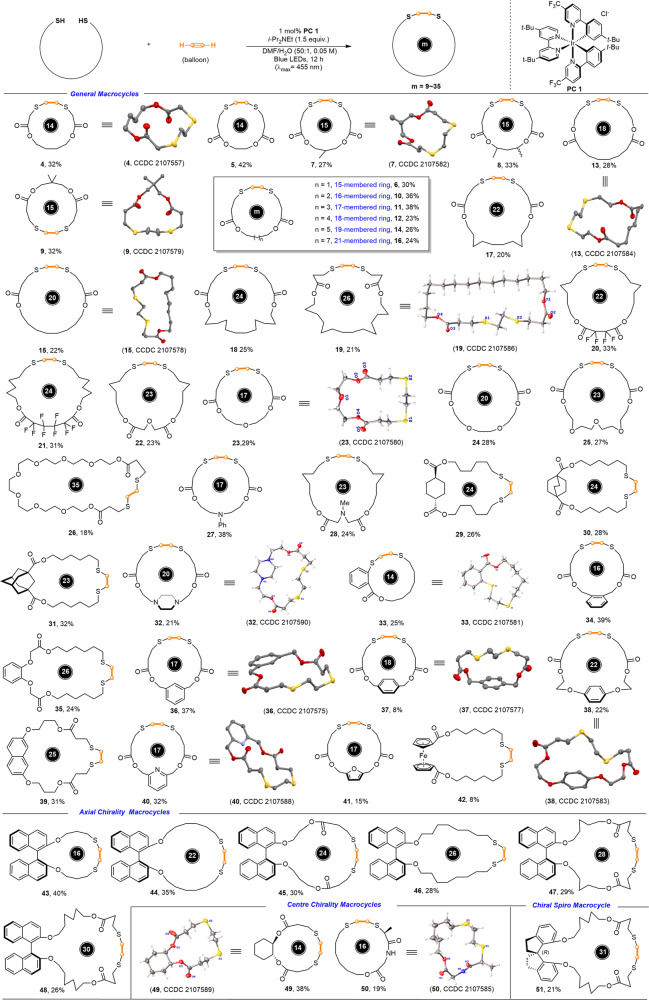
Substrate scope for synthesis of [1 + 1] patter-based macrocycles. Reaction conditions: DMF (*N,N*-dimethylformamide), H_2_O (50:1, 0.05 M), disulfide (0.25 mmol), **PC1** (2.5 μmol, 1.0 mol%) and a balloon was filled with acetylene gas until its size was roughly 20 cm in diameter.

With the optimized conditions in hand, we investigated the scope of the photocatalyzed acetylene-enabled macrocyclization protocol. As shown in (Fig. 2), a variety of ester-tethered dithiols with different linkers could be applied as suitable substrates, giving the target macrocycle products in acceptable yields under the standard reaction conditions. For example, the macrocycles (**4**–**21**) with the ring sizes ranging from 14 to 26 could be easily assembled in 20–42% yields when linear diester-linked dithiols were utilized as substrates. The introduction of methyl group (**7**–**9**) or fluorine atom (**20**–**21**) onto the diester-linker displayed no observable effect on the macrocyclization efficiency. In addition to the modulation of the substituents on the linking-chain, the introduction of heteroatoms such as O and N into the linkers was also well compatible with the standard catalytic system, giving rise to the corresponding O,S- and O,S,N-decorating macrocycles (**22**–**28**), respectively, in 18–38% yields. It is particularly worth mentioning that the 35-membered macrocyclic compound (**26**), which represents a new and unique oxo-thiocrown ether, could be facilely accessed in 18% yield. The RN-tethered ring compounds (**27** and **28)**, which were difficult to access through the traditional S_N_2 reaction of dihaloalkane and dithiol due to the competing cross reaction of amine with alkyl halide^37^, were also successfully assembled in 38 and 24% yields, respectively. Compared with the flexible linear dithiols, the relatively rigid ring-tethered dithiols were supposed to be more challenging substrates for macrocyclization reactions due to the higher transannular strain and the greater bond angle and length deformations during the ring closure process. The dithiols with linkers of cycloalkanes (**29**–**31**), heterocycloalkane (**32**), arenes (**33**–**39**), heteroarenes (**40**–**41**) and even ferrocene (**42**) were applicable to this unique system, affording the desired macrocycles in 8–39% yields with the ring sizes ranging from 16 to 26. For example, *trans*−1,4-cyclohexanedicarboxylate (**29**), 1,4-bicyclo[2.2.2]octanedicarboxylate (**30**) and 1,3-adamantanedicarboxylate (**31**) were all suitable linkers. The dithiols tethered with the *ortho*- and *meta*-disubstituted benzene ring could be employed as suitable substrates, delivering the desired macrocycles (**33**–**36**) in 24–39% yields. However, *para*-substituted substrate gave the corresponding 18-membered ring product (**37**) only in 8% yield. Further extending the side chain length of the *para*-substituted dithiol greatly improved the reaction efficiency, furnishing the corresponding product (**38**) with 22% yield. In addition to benzene ring, naphthalene, pyridine and furan could also be introduced as the tethers of the dithiols and served as a segment of the resulting macrocycles (**39**–**41**). Strikingly, the ferrocene tether was also enabled the macrocyclization under standard procedure, albeit in a relatively lower yield (**42**, 8%). It is worth to note that many of the resulting macrocycles readily recrystallized to give colorless and odor-free crystalline solids suitable for X-ray diffraction analysis. The X-ray structures of (**4**, **7**, **13**, and **19**) clearly indicated that the S-C-C-S units preferred the *anti*-conformation, which was opposite to the oxo-analogues (*cis*-conformation). The trans conformational preference of S-C-C-S might originate from the S^….^S repulsive interaction^38,39^ that acted as unique “hubs” in the backbone of macrocycles to modulate conformation. Considering that 1,1′-bi-2-naphthol (BIONOL) is an efficient axial-chiral element for asymmetric catalysis^40,41^, (*R*)-( + )-BINOL-derived dithiols were also employed as the substrates to test the above the radical thiol-acetylene macrocyclization reaction. We were pleased to find that a set of linear axially chiral-linked dithiols can successfully afford the desired axially chiral macrocyclic crown thioethers in acceptable isolated yields of pure isomers owing to the enlist acetylene, orthogonal to previous methods^17^. As shown in (Fig. 2, bottom), crown thioethers (**58** and **59**) with central chirality could be easily assembled when (1 *S*, 2 *S*)-trans-1,2-cyclohexanediol and (*S*)-(2-mercaptopropanoyl)glycine derived dithiols were employed. The X-ray structures of macrocycles (**49** and **50**) indicated that unique chiral macrocycle cavities were created. In addition to the centrally and axially chiral elements, the spiro chirality could be introduced as well with (*R*)−2,2′,3,3′-tetrahydro-1,1′-spirobi[indene]−7,7′-diol-derived dithiol, furnishing the corresponding 31-membered thio-containing macrolide (**51**) in 21% yield. These privileged and elegant chiral macrocyclic skeletons could be potentially utilized in asymmetric catalysis in the future. It should be noted that acetylene was replaced with ethynylbenzene or 1-hexyne, no any macrocyclization products were detected under the standard conditions, but only produced the undesired linear vinyl thioether (see Supplementary Information for details). These results indicated that the small sized diterminal acetylene as the linker is essential for the current macrocyclization protocol. Actually, recent reports on visible-light-promoted hydrothiolation of the substituted alkynes are also mainly limited to monoaddition^21,22^, presumably due to the inherent steric hindrance of the substituted alkynes^15^.

**Fig. 3:**
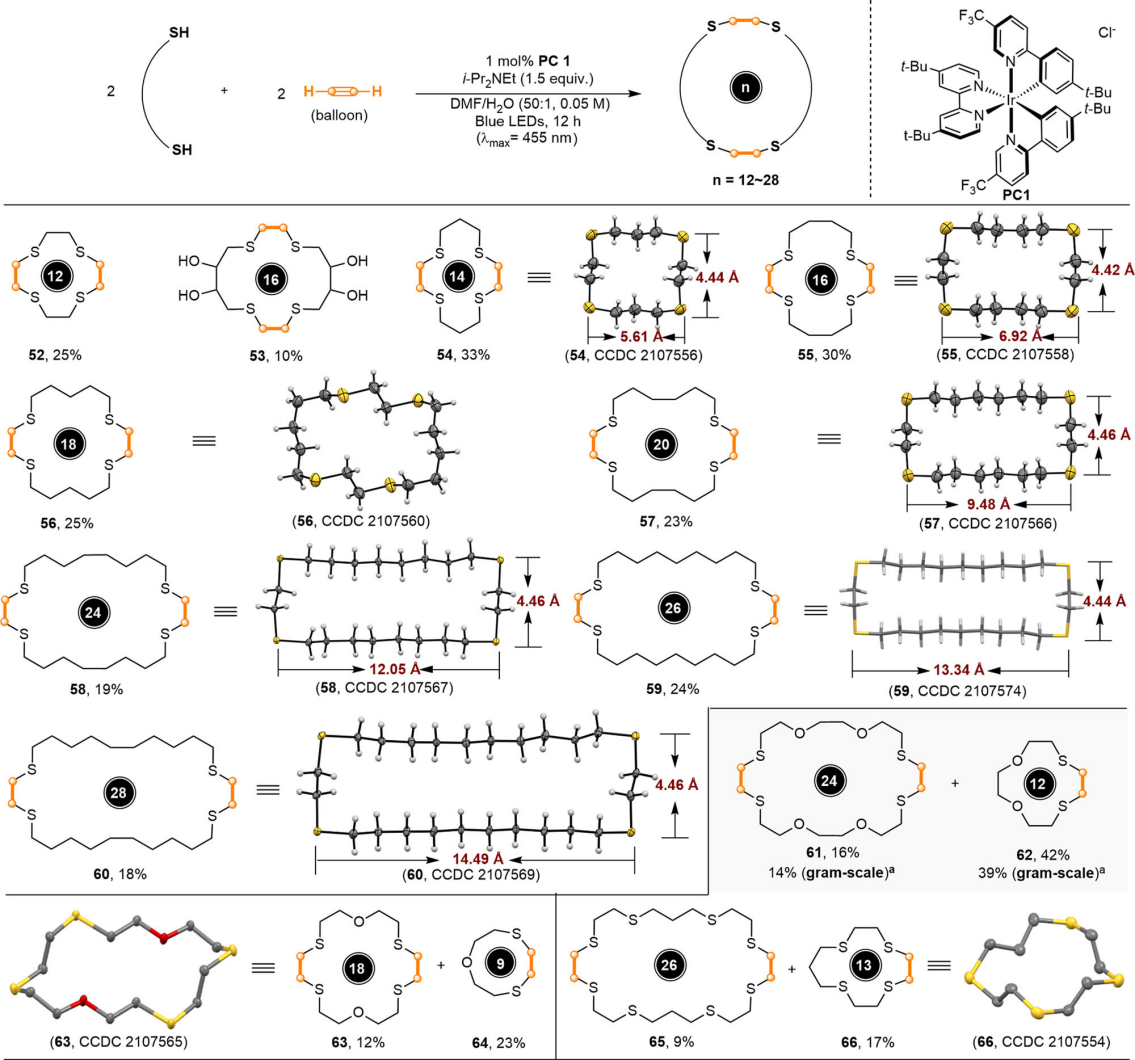
Substrate scope for synthesis of [2 + 2] and [1 + 1] patter-based macrocycles. Reaction conditions: Disulfide (0.25 mmol), **PC1** (2.5 μmol, 1.0 mol%) and a balloon was filled with acetylene gas until its size was roughly 20 cm in diameter. ^a^10 mmol-scale.

Interestingly, when the dithiols based on linkers other than esters were employed as the substrates, a highly modular and unexpected 2 + 2 process instead of 1 + 1 macrocyclization process occurred. As shown in (Fig. 3), different macrocyclic crown thioethers (**52**–**66**) with ring sizes from 12 to 28 atoms were successfully constructed in 9–42% yields in the presence of gaseous acetylene. In terms of molecular constitution, this multimolecular macrocyclization incorporated four sulfur atoms in the final products, in which eight chemical bonds (four C–S and four C–H bonds) were created. For the 1,n-dithiols with all-carbon linkage, four-molecule macrocyclization products (**52**–**60**) were selectively formed, with yields ranging from 10 to 33%.

Noteworthy, the hydroxyl group was well-tolerated for this reaction, producing the corresponding hydroxyl-decorating thioether (**54**) in 10% yield. Further investigations indicated that the use of sulfur/oxygen-embeded dithiols compounds as substrates gave both of four-molecule and two-molecule macrocyclization products (**61**–**66**). Gram-scale synthesis of (**61** and **62**) was also successfully performed without dramatic erosion in product yields. Like the macrocycles listed in (Fig. 2), most macrocyclic crown thioethers in (Fig. 3) facilely recrystallized as well to give colorless and odor-free crystalline solids suitable for X-ray diffraction analysis. The crystal structures showed that the macrocyclic crown thioethers linked with all-carbon linkages generally adopted unique quadrangular conformation, usually with the sulfur atoms sitting at the corners (**53**, **55**, **57**, **59** and **60**). The heights of the rectangles were almost constant (about 4.4 Å). The widths, however, depended on the carbon atom number of the dithiol linker. Among them, crown thioethers (**58**–**60**) derived from the dithiols with the linkages containing more than 16 carbon atoms have the width greater than 12 Å, which were on the order of nanometers. The crystal structures of crown thioethers (**56**, **63**, and **66**) were twisted and the cavity became broader compared with the cavity of other crown thioethers. All the crystal structures showed that the S-C-C-S units uniformly adopted the *anti*-conformation.

The unique structure conformation, in combination with the easy accessibility may open new doors for potential applications of these sulfuric macrocycles in other disciplines^42,43^. The different reaction selectivity of [1 + 1] and [2 + 2] patters might be attributed to the ring sizes of the resulting macrocycles. Cyclization for the dithiols in (Fig. 3) in the [1 + 1] pattern would provide the corresponding thioethers with smaller ring size ranging from 6 to 14, which should be unfavorable due to the higher ring strain. Results for compounds (**61**–**66**) indicate a more complex situation, with some role being played by the presence of strongly coordinating atoms, as in this case, [1 + 1] coexists with [2 + 2] cyclization, but [1 + 1] being predominant.

**Fig. 4:**
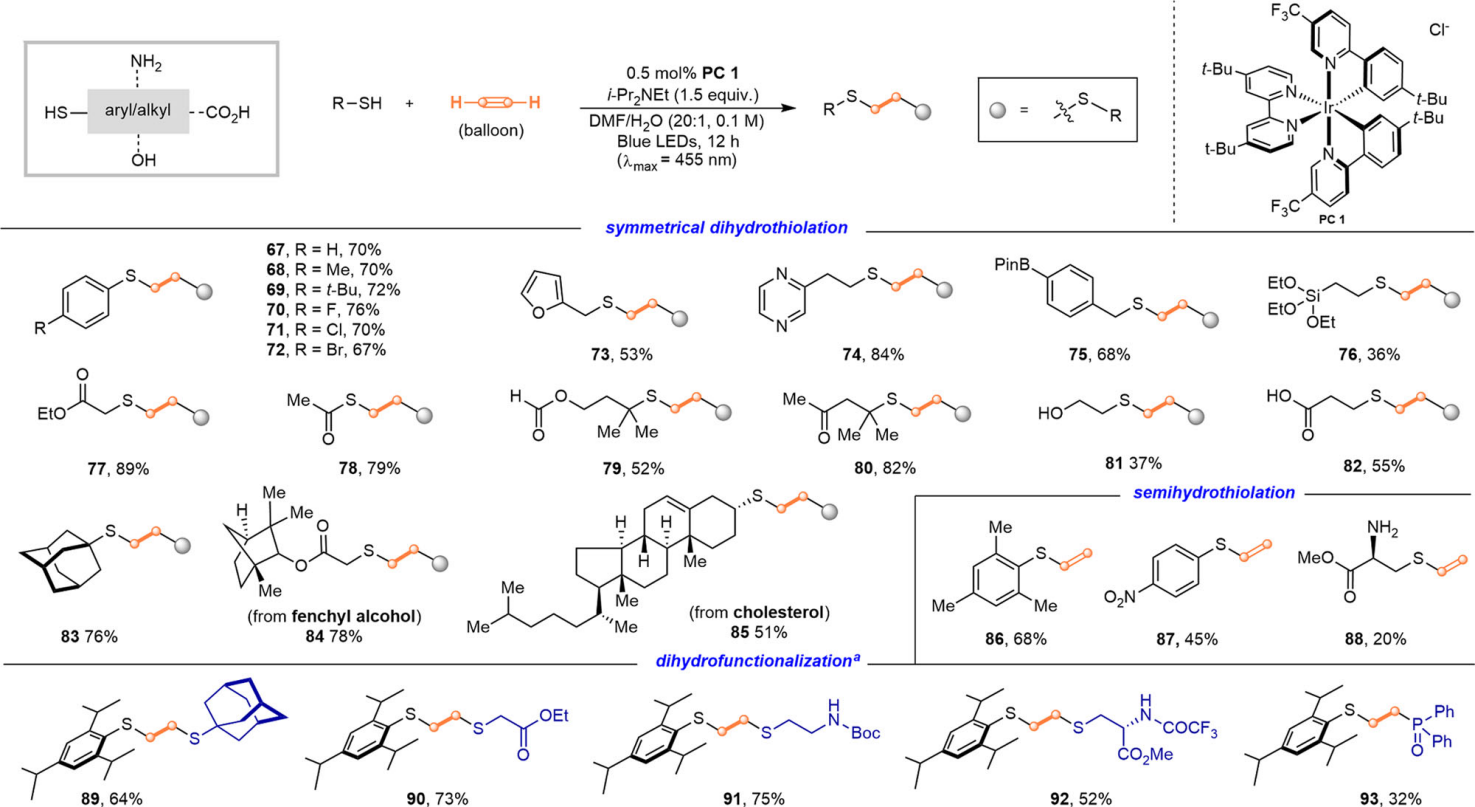
Substrate scope for synthesis of linear C2-linked 1,2-bisthioether. Reaction conditions: Thiols (0.5 mmol), **PC1** (0.5 mol%) and a balloon was filled with acetylene gas until its size was roughly 20 cm in diameter. 4-Chlorobenzyl-protected amine **88** was isolated instead of the free amine **88**. **a** Unsymmetrical dihydrofunctionalization reaction: reactions were conducted with 0.25 mmol of 2,4,6-triisopropylbenzenethiol and 1.1 equiv. of another thiol-coupling partner.

After the successful realization of this visible light-mediated thiol-acetylene click chemistry-enabled macrocyclization, we also extended this protocol to direct assembly of linear C2-linked 1,2-bisthioether using readily available thiols. As shown in (Fig. 4), the catalytic method is well compatible with a wide array of linear aromatic and aliphatic thiols bearing different reactive functional groups under slightly modified reaction conditions, as exemplified by the formation of symmetric C2-linked 1,2-(S-S) scaffolds (**67**–**88**) with the yields ranging from 37 to 84%. This thiol-acetylene click reaction showed extraordinary functional group tolerance and biocompatibility. The furyl (**73**), pyrazinyl (**74**), halogens (**70**–**72**), boronate ester (**76**), silicate ester (**77**), ester (**77**–**79**), ketone (**80**), and even unprotected hydroxyl (**81**) and carboxylic acid (**80**) were well tolerated. It seemed that the steric hinderance had no obvious influence on the reaction performance. The tertiary alkyl thiols successfully gave the desired products (**79**, **80**, and **83**) in 52–82% yields. This methodology can be also used for the late-stage modification of nature products containing competing functional groups. For example, thiols derived from fenchyl alcohol and cholesterol could be easily glued on the acetylene as expected, affording the corresponding double hydrothiolation products (**84** and **85**) in 78 and 51% yield, respectively. Interestingly, sterically bulky 2,4,6-trimethylbenzenethiol and electron-deficient 4-nitrothiophenol proceeded well, but leading to the formation of monohydrothiolation (**86** and **87**) in 68 and 45% yield, respectively. For cysteine methyl ester, a unique amino acid derived thiol, also provided vinyl thioether (**88**, 20%). In addition to symmetrical dihydrothiolation, this approach has also been used for the construction of unsymmetrical linear C2-linked 1,2-bisthioethers (**89**–**92**), which were difficult to be accessed in other methods. It is worth noting that this method can even been extended to assemble the C2-linked 1,2-(S-P)-molecule (**93**, 32%) in the presence of 2,4,6-triisopropylbenzenethiol and diphenylphosphine oxide, albeit in a relatively lower yield.

### Applications

**Fig. 5:**
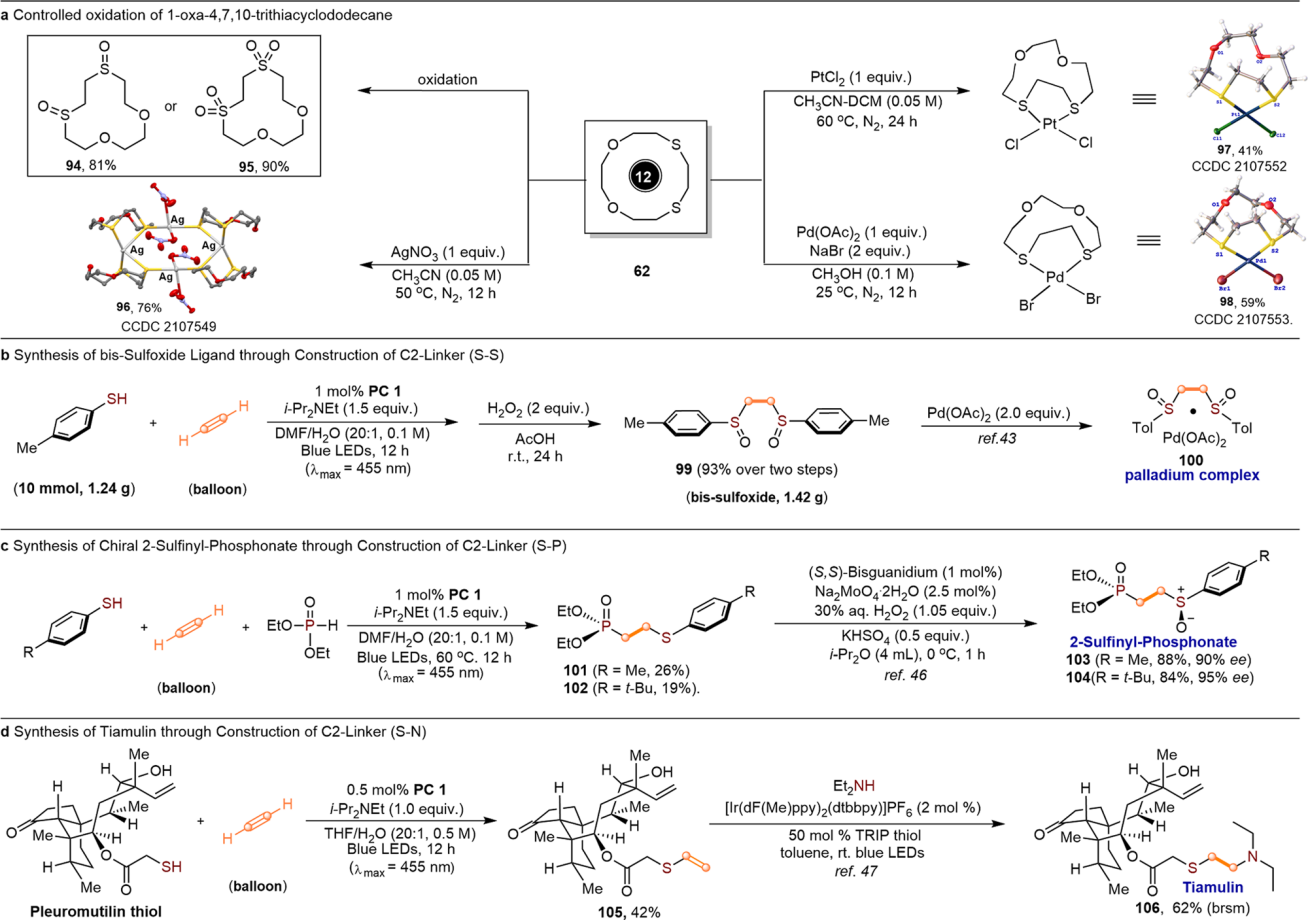
Applications. **a** Controlled oxidation of 1-oxa-4,7,10-trithiacyclododecane. **b** Synthesis of bis-sulfoxide ligand. **c** Synthesis of chiral 2-sulfinyl-phosphonate. **d** Synthesis of tiamulin.

To further demonstrate the potential synthetic applications of this unique methodology, further transformations of the macrocyclic crown thioether, the synthesis of chiral ligands and drug molecule were also performed. As shown in (Fig. 5a top left), the macrocyclic thioether, 1,4-dioxa-7,10-dithiacyclododecane (**62**) could be selectively oxidized to the corresponding sulfoxide (**94**) and sulfone (**95**) in excellent yields. It’s well-known that the crown ethers are excellent ligands that can be coordinated to most of the metal elements^44,45^. Therefore, three most commonly-used transition metal salts (Ag, Pt, and Pd) were then tested as the complexes core to coordinate with the oxo-thiocrown ether (**62**). It is interesting to note that a stable and new 4:4 polynuclear metal cluster (**96**) was isolated as the major product when AgNO_3_ was applied. For the metal salts of PtCl_2_ and Pd(OAc)_2_/NaBr, the 1:1 bidentate complexes (**97** and **98**) were obtained in 41% and 59% yield, respectively. The crystal structures of (**96**–**98**) clearly showed that the coordinating sulfur atoms adopted an exodentate orientation, which was opposite to the endodentate orientation commonly found in oxa- and aza-crowns (Fig. 5a). Furthermore, the dihydrothiolation reaction for the linear dithioether could be carried out in gram-scales, followed by the selective oxidation with aqueous hydrogen peroxide to afford the bis-sulfoxide derivative (**99**) in 93% yield over two steps. This bis-sulfoxide (**99**) is an excellent bidentate ligand and could form palladium(II) acetate complex (**100**) via routine metal complexation, which is a well-known catalyst widely used in transition-metal-catalyzed allylic C-H functionalization^46^ (Fig. 5b). Beyond enabling linear 1,2-(S-S) backbone formation, this existing protocol has also aided the downstream dihydrofunctionalization with two different coupling partners. For example, when arylthiol and diethyl phosphonate were added as the co-coupling partners under the modified reaction conditions in a one-pot manner, the S-C-C-P containing products 2-(arylthio)ethyl)phosphonates (**101** and **102**) were successfully produced, albeit in relatively lower yields. Considering that chiral sulfoxides skeletons are prevalent structural motifs in pharmaceuticals^47^ and chiral ligands^48^, the molecules (**101** and **102**) could be asymmetrically oxidized to give the chiral sulfoxides (2-sunfinyl-phosphonates (**103** and **104**) in excellent yields (84–88%) and enantioselectivities (91–95% ee) with the aid of bisguanidinium-catalyzed sulfoxidation reaction developed by Tan (Fig. 5c)^49^. Finally, to further showcase the power of this methodology, the total synthesis of Tiamulin, a densely functionalized pleuromutilin antibiotic drug that contains a unique C2-linker (S-C-C-N) fragment, was also accomplished with this new acetylene-based hydrofunctionalization as key step. As shown in (Fig. 5d), the synthesis relies on two successive photocatalytic hydrofunctionalization reactions. The key intermediate, vinyl thioether (**93**), could be synthesized in 42% yield from the commercially available pleuromutilin thiol through hydrothiolation of acetylene (1 atm) under the standard conditions. With the key intermediate (**105**) in hand, the desired Tiamulin (**106**) could be eventually accessed in 62% yield (base on recovering starting materials) through the photocatalytic hydroamination reaction^50^.

### Mechanistic discussion

**Fig. 6:**
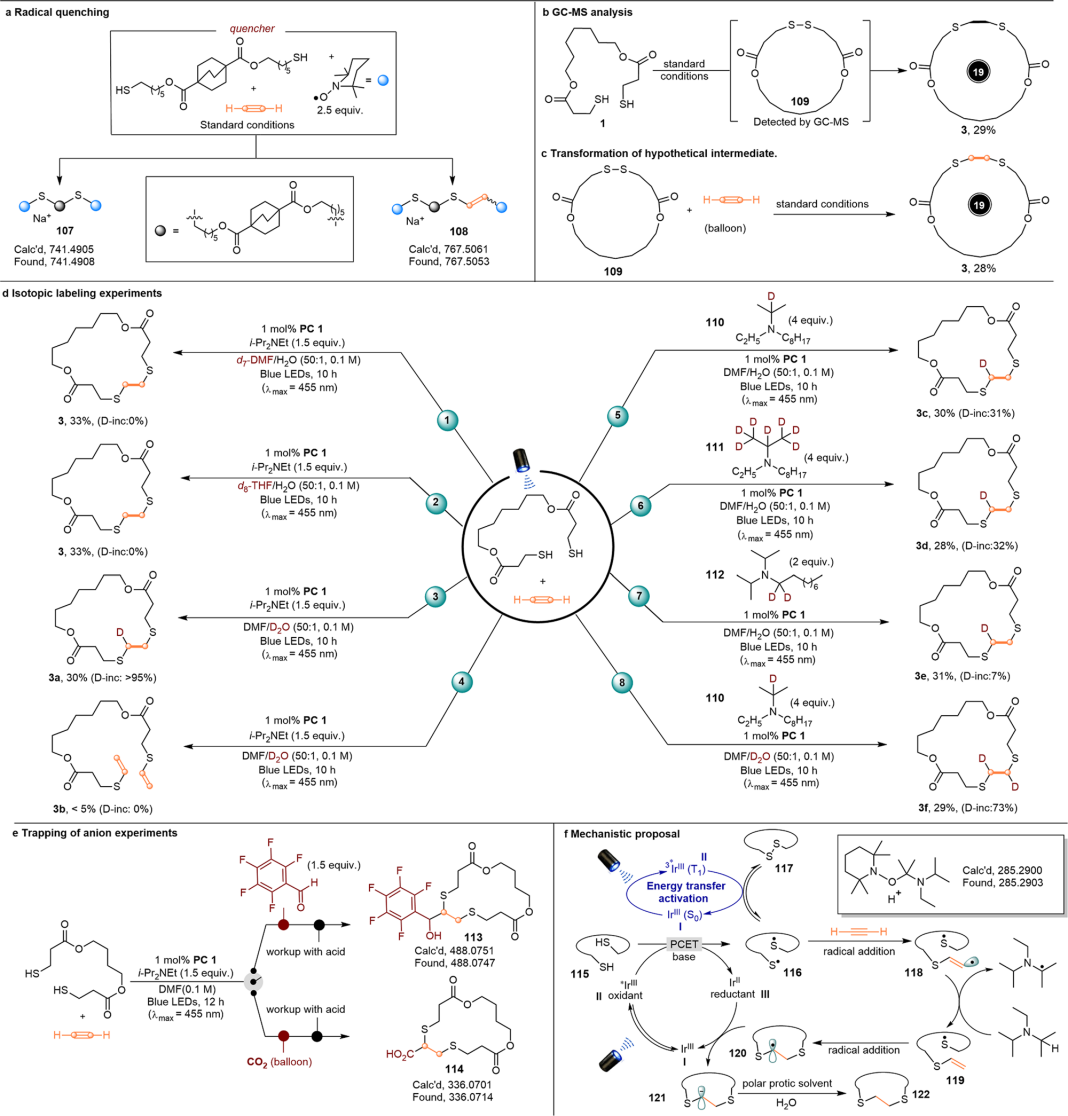
Mechanistic studies. **a** Radical trapping experiment. **b** GC-MS analysis. **c** Transformation of hypothetical intermediate. **d** Isotopic labeling experiments. **e** Trapping of carbanion intermediate. f Proposed reaction mechanism.

Despite sulfur radical addition to substituted alkynes being well documented^33^, however, the acetylene-based system is much different due to the terminal sigma-vinyl radical intermediate. To depict the detailed mechanistic profile, extensive control reactions were then designed and conducted. First, the classic radical quenching experiments reactions were conducted under the standard conditions. As shown in (Fig. 6a), when radical quencher 2,2,6,6-tetramethyl-1-piperinedinyloxy (TEMPO) was subjected to the standard conditions, the radical coupling products (**107** and **108**) could be detected by HRMS, which indicated that the corresponding thiol radical and *sigma*-vinyl radical intermediates were involved in the reaction system. Inspired by Glorius’ seminal report^51^, wherein [Ir(dF(CF_3_)ppy)_2_(dtbbpy)][PF_6_] was employed as photocatalyst and dimethyl disulfide was demonstrated as the competent quenchant, we then wondered that the disulfide might also be the key intermediate in our system. As expected, the cyclic disulfide intermediate (**109**) was observed during the reactions (Fig. 6b), as judged from the analysis of the crude reaction mixture by GC-MS (see Supplementary Figs. 8 and 9). Furthermore, as shown in (Fig. 6c), the desired macrocyclic product (**3**) was obtained in 28% when the linear dithiol was replaced by the cyclic disulfide (**109**) under the standard conditions. These results supported that the cyclic disulfide was the key intermediate in the multimolecular macrocyclization protocol. Further, we also investigated the hydrogen sources of the newly formed C-H bonds in the final products by isotope labeling experiments (Fig. 6d). Conducting the reaction in *d*_7_-DMF/H_2_O or *d*_8_-THF/H_2_O produced the product (**3**) without D-incorporation, ruling out the possibility of HAT with solvent of *d*_7_-DMF or *d*_8_-THF (Fig. 6d, eqs. 1 and 2). As exemplified by using D_2_O in place of H_2_O, the mono-deuterated macrocycle (**3a**) was obtained with >95% D incorporation, accompanied by the formation of trace amount of non-deuterium linear vinyl thioether (**3b**) (Fig. 6d, eqs. 3 and 4). On the basis of these control experiment results (Fig. 6d, eqs. 1–4), it could be deduced that H_2_O was only involved in the second hydrothiolation process (forming sp^3^ C-H) and the solvents of DMF and THF were excluded for both HAT processes. As organic amine often provides the hydrogen source in photoredox system, we further speculated the base of DIPEA might be the first HAT source (forming sp^2^ C-H). To verify this hypothesis, more isotope labeling experiments were then designed. As the deuterated DIPEA is not commercially available, we synthesized DIPEA analogues **110**–**112** deuterated at different specific positions. Firstly, the desired product (**3c**) with 31% D-incorporation was afforded when deuterated amine **110** (*d*_1_-isopropyl with deuterium labelling at tertiary-position) was employed under the standard reaction conditions (Fig. 6d, eq. 5), which inferred that the tertiary C-H of the isopropyl was one of the HAT sources. Secondly, when the amine with a higher degree of deuterium labelling (*d*_7_-isopropyl) was utilized as the base **111** instead under the same reaction conditions, the product (**3d**) with similar D-incorporation (32%) was obtained (Fig. 6d, eq. 6), which implied that the methyl groups of isopropyl were not involved in the HAT process. Thirdly, using the deuterated amine **112** (*d*_2_-octyl with deuterium labelling at the secondary-position adjacent to nitrogen) as the base gave the product (**3c**) in only 7% D-incorporation (Fig. 6d, eq. 7). Collectively, these isotope labeling experiments suggested that organic base DIPEA should be the first HAT source for the radical thiol-acetylene click reaction. This conjecture was also supported by the TEMPO trapping experiment, in which the formation of TEMPO-DIPEA could be detected by HRMS (see Supplementary Fig. 7).

In addition, it can also be deduced that the HAT rates of different hydrogen atoms of DIPEA are in the following order: tertiary C-H > secondary C-H » primary C-H. Finally, high level of total deuterium incorporation was achieved when the combination of deuterated amine **110** and D_2_O were applied as the D-sources (Fig. 6d, eq. 8). Together, this series of experiments strongly suggested that polar protic solvent water and organic base DIPEA were the species responsible for the hydrogen sources of two sequential HAT processes. Based on the above reaction results, we envisaged that radical-to-polar crossover photoredox catalysis was more likely to operate for this system. This was also confirmed by the electrophile-trapping experiments^52^, in which the corresponding coupling products (**113** and **114**) were detected by HRMS when the electrophilic pentafluorobenzaldehyde and CO_2_ were added in the reaction system (Fig. 6e). These results strongly supported the existence of carbanion intermediate (see Supplementary Figs. 15 and 16).

Based on the control experiment results and literature precedents^51–54^, a plausible reaction mechanism is then proposed for this photoredox catalysis (Fig. 6f). The photocatalyst Ir[*t*Bu(CF_3_)ppy]_2_(dtbbpy)[Cl] is initially promoted to singlet excited state by excitation with visible light and undergoes subsequent intersystem crossing. Concomitantly in solution, a Lewis base can coordinate to the thiol substrate **115**, which undergoes a proton-coupled electron transfer (PCET) with photoexcited catalysts to yield the reduced Ir^II^ (**III**) and R-S• radical (**116**)^55^, which is then followed by rapid homo-coupling to give the disulfide specie (**117**) as a radical reservoir or intercepted by acetylene. Moreover, Stern–Volmer luminescence quenching studies further revealed that the complex of **1** and DIPEA was the most effective quencher to the excited photocatalyst, which was consistent with the proposed PCET pathway (see Supplementary Figs. 17–21). Alternatively, as shown in blue cycle, the excited state Ir[*t*Bu(CF_3_)ppy]_2_(dtbbpy)[Cl] (**II**) also acts as a triplet sensitizer, which then transfers the triplet energy to the disulfide (**117**), thereby enables access to homolytic S-S bond cleavage, again generating the corresponding thiyl radical (**116**)^51^. In turn, intermolecular addition of this open-shell intermediate to the acetylene gas gives vinyl radical intermediate (**118**), which is then followed by the HAT with DIPEA to yield vinyl thioether (**119**) Simultaneously, another electrophilic R-S• radical is captured by vinyl thioether (**119**) to give rise to the π-radical intermediate (**120**), due to the Kharasch effect^56^. Single electron reduction of the radical (**120**) by Ir^II^ (**III**) to generate the kinetically and thermodynamically stabilized carbanion (**121**) resting on the neighboring heteroatom^57^, which can be intercepted by water, leading to the formation of the corresponding product (**122**).

## Discussion

We have developed an efficient visible-light-mediated radical thiol-acetylene click chemistry-enabled macrocyclization. A set of structurally diverse sulfur-containing macrocycles (with the ring sizes up to 35 atoms) can be prepared using readily available acetylene gas (in balloon) and linear dithiols. Furthermore, we have extended the range of homologous coupling partners to different coupling partners for synthesis linear C2-linked 1,2-(S-S/S-P/S-N) functionalized molecules in one pot manner. The unique structural property of acetylene provides a new chance to addressing the long-standing challenge of thiol-yne click chemistry. The radical-polar crossover mechanism is supported by various experimental probes, including radical capture, kinetic analyses, isotopic labeling and intermediate capture. This operationally straightforward reaction is also amenable to the synthesis of organometallic complexes, bis-sulfoxide ligand and a pleuromutilin antibiotic drug Tiamulin. We hope that this work could inspire organic chemists to develop more practical strategies for the value-added functionalization of the feedstock acetylene gas by leveraging the reliable click chemistry.

## Methods

### General procedure for macrocyclization

To an oven-dried 25 mL Schlenk tube, [Ir[d(*t*Bu)(CF_3_)ppy]_2_(dtbbpy)[Cl] (1.0 mol%), linear dithiol precursor (1.0 equiv.), DMF (0.05 M), *N*,*N*-diisopropylethylamine (1.5 equiv.) and H_2_O (DMF/H_2_O = 50/1) were added sequentially under N_2_. The tube was degassed through three freeze-pump-thaw cycles under acetylene and then an acetylene gas balloon was attached through a three-way valve. After that, the tube was placed in the Parallel Light Reactor, which cooled with the recirculated cooling water. The reaction mixture was stirred for 12 h, quenched via exposure to air, diluted with brine (4 mL) and then extracted by EtOAc (2 × 5 mL). The combined organic layer was dried over Na_2_SO_4_, filtered and evaporated. The residue was purified by flash column chromatography on silica gel to obtain the product.

## Supplementary information

Supplementary Information

## Data Availability

The authors declare that all other data supporting the findings of this study are available within the article and Supplementary Information files. The X-ray crystallographic coordinates for structures reported in this study have been deposited at the Cambridge Crystallographic Data Centre (CCDC). These data can be obtained free of charge from the Cambridge Crystallographic Data Centre via http://www.ccdc.cam.ac.uk/data_request/cif.
