# Supplementary material for: Thiol-Yne click chemistry of acetylene-enabled macrocyclization
Source: Nat Commun. 2022 Aug 25;13:5001. doi: 10.1038/s41467-022-32723-0 (PMC9411599; doi:10.1038/s41467-022-32723-0)
Supplement: Supplementary file 1 — Supplementary Information [file 41467_2022_32723_MOESM1_ESM.pdf]

Supplementary Information

Thiol-Yne Click Chemistry of Acetylene-Enabled Macrocyclization

Shiwei Lü, Zipeng Wang & Shifa Zhu*

Key Laboratory of Functional Molecular Engineering of Guangdong Province, School of Chemistry and Chemical Engineering, South China University of Technology, Guangzhou 510641, China.

Table of Contents

Supplementary Methods 2

1. General reagent information 2

2. General analytical information 2

3. Optimization of the thiol-yne click chemistry of acetylene-enabled macrocyclization 3

4. General Procedures and Characterization Data of Substrates 7

5. General procedure of thiol-yne click chemistry of acetylene-enabled macrocyclization 27

6. Gram-Scale Reaction and Synthetic Transformation 63

7. Control Experiments for Mechanistic Investigation 71

8. X-ray structure analysis 86

Supplementary NMR Spectra and HPLC Spectra 114

Supplementary Reference 284

Supplementary Methods

1. General reagent information

All reactions were carried out in flame-dried sealed tubes with magnetic stirring. Unless otherwise noted, all experiments were performed under acetylene atmosphere. A part of solvent were treated with 4 Å molecular sieves or sodium and distilled prior to use. All of the other reagents were purchased from Sigma-Aldrich, Alfa Aesar, Acros, TCI, J&K, Energy Chemical and were used as received. Flash chromatography was performed using glass columns (*Synthware*) with silica gel (Huanghai, 300-400 mesh).

2. General analytical information

^{1}H NMR, ^{13}C NMR, ^{31}P NMR and ^{19}F NMR spectra were recorded on a Bruker Avance DPX 600 fourier Transform spectrometer (400 MHz or 500 MHz respectively). The data of ^{1}H NMR, ^{13}C NMR and ^{19}F NMR spectra are reported as follows: chemical shift (δ ppm), multiplicity (s = singlet, d = doublet, t = triplet, q = quartet, m = multiplet, dd = doublet of doublet, dt = doublet of triplet), integration, coupling constant (Hz) and assignment. Chemical shifts (ppm) are reported in ppm using TMS as internal standard and spin-spin coupling constants (*J*) are given in Hz. IR spectra were obtained on a Nicolet iS10 spectrophotometer and reported in terms of frequency of absorption (cm^{-1}) in potassium bromide (KBr) pellet. GC analyses were performed on an Thermo Fisher TRACE 1300 gas chromatograph. High Resolution on Mass spectra were obtained an Agilent 6540 Series Q-TOF system equipped with ESI. Fluorescence spectra were recorded on Hitachi F-4500 fluorescence spectrophotometers. Melting points were determined using a hot stage apparatus. Photochemical experiments have been performed in a Parallel Light Reactor (designed by WATTCAS: WP-TEC-1020HSL, 10 W, λ_{max} = 455 nm, tube about 2-5 cm away from lights). The temperature of the reaction was maintained at approximately room temperature with the recirculated water or fans. The reaction setups can be seen below (**Supplementary Figure 1 and 2**). Kessil PR160-370 and Kessil PR160-390 lights be used for Gram-scale synthesis. Organic solutions were concentrated under reduced pressure on a Heidolph Hei-VAP rotary evaporator using a water bath. Reactions were monitored by GC-MS analysis, thin-layer chromatography (TLC) was carried out on 0.20 mm Huanghai silica gel plates (HSGF254) using UV detection under 245 nm as a visualizing agent. X-ray crystallography analysis was performed on Rigaku Oxford Diffraction Supernova Dual Source. Visualization of the developed chromatogram was performed by fluorescence quenching, anisaldehyde, ceric ammonium molybdate, $KMnO_4$ stain or Phosphomolybdic acid hydrate (10g of PMA + 100mL ethanol).

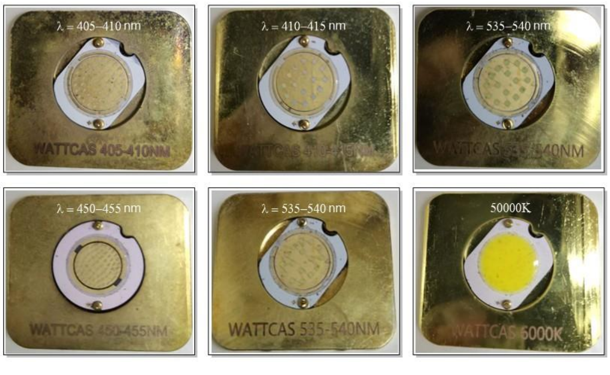

Supplementary Figure 1. Picture of the light source embedded in a conductive metal carrier

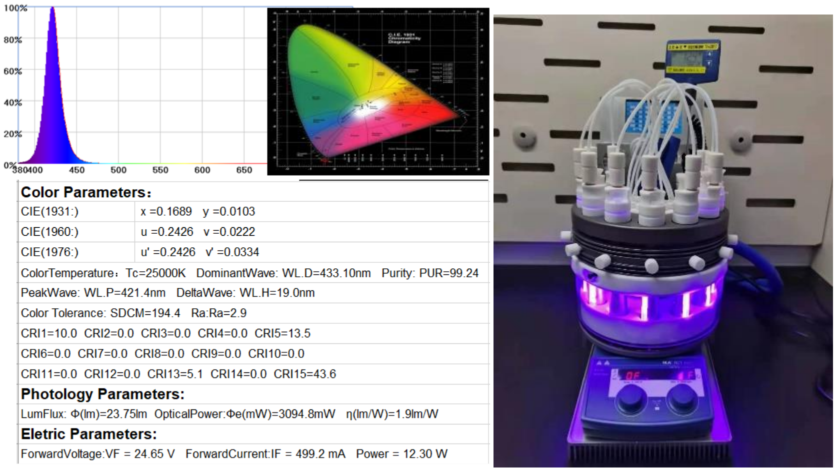

Supplementary Figure 2. Emission spectra of the blue LED light source and picture of experimental setup used within the project

3. Optimization of the thiol-yne click chemistry of acetylene-enabled macrocyclization

Supplementary Table 1. The effect of the metal-catalysts on the acetylene-enabled macrocyclization[a]

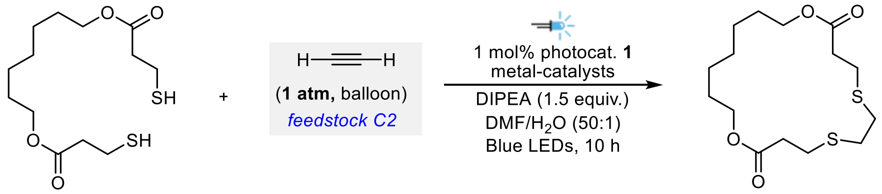

Entry	metal-catalysts (0.25 equiv.)	Yield (%)[b]	photocat. **1**
1	$NiCl_2$·glyme	21	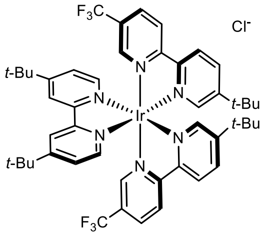
2	$Ni(acac)_2$	15	
3	NiI_2	ND	
4	CuCl	23	
5	$Cu(OAc)_2·H_2O$	<5	
6	$Cu(CH_3CN)_4PF_6$	16	
7	$CuSO_4$	<5	
8	$Pd(PPh_3)_4$	ND	
9	$PdCl_2(PPh_3)_2$	ND	
10	$Fe(OTf)_3$	<5	
11	$Fe(acac)_3$	<5	
12	NaCl	<5	

[a]Reaction conditions: heptane-1,7-diyl bis(3-mercaptopropanoate) (0.1 mmol), acetylene gas (a balloon was filled with acetylene gas until its size was roughly 5 cm in diameter), DIPEA (1.5 equiv.), and photocatalyst (1 mol%) in degassed solvent and irradiated with a 10 W blue LED lamp for 10 h. [b]Yields determined by ^1H NMR using 1,3,5-trimethoxybenzene as an internal standard.

Supplementary Table 2. The effect of the bases on the acetylene-enabled macrocyclization[a]

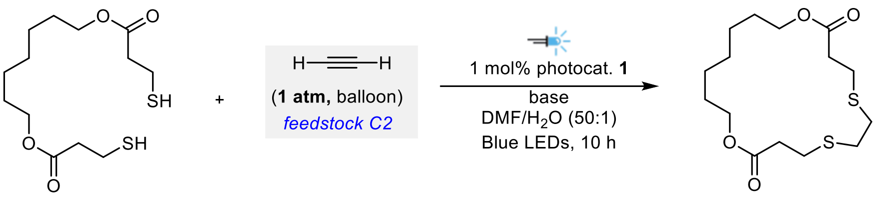

Entry	base (1.5 equiv.)	Yield (%)[b]	photocat. **1**
1	K_2CO_3	ND	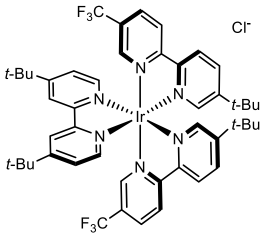
2	Cs_2CO_3	ND	
3	Na_2CO_3	ND	
4	$NaHCO_3$	trace	
5	DMAP	trace	
6	DIPEA	33	
7	TEA	10	
8	no	ND	

[a]Reaction conditions: heptane-1,7-diyl bis(3-mercaptopropanoate) (0.1 mmol), acetylene gas (a balloon was filled with acetylene gas until its size was roughly 5 cm in diameter), base (1.5 equiv.), and photocatalyst (1 mol%) in degassed solvent and irradiated with a 10 W blue LED lamp for 10 h. [b]Yields determined by ^1H NMR using 1,3,5-trimethoxybenzene as an internal standard.

Supplementary Table 3. The effect of the solvents on the acetylene-enabled macrocyclization[a]

Learning from on the Wikipedia[1], acetylene has better solubility in in acetone, dimethylformamide (DMF) and DMSO.

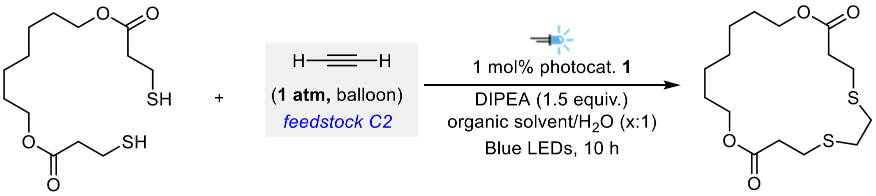

Entry	solvent	Yield (%)[b]	photocat. **1**
1	DMSO	10	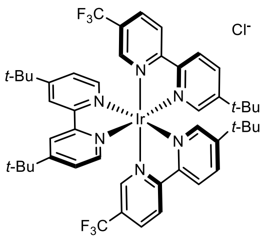
2	DCM	<5	
3	$CHCl_3$	<5	
4	Dioxane	23	
5	THF	25	
6	Et_2O	<5	
7	DME	20	
8	DMF	29	
9	Acetone	21	

[a]Reaction conditions: heptane-1,7-diyl bis(3-mercaptopropanoate) (0.1 mmol), acetylene gas (a balloon was filled with acetylene gas until its size was roughly 5 cm in diameter), solvent (2 mL), DIPEA (1.5 equiv.), and photocatalyst (1 mol%) in degassed solvent and irradiated with a 10 W blue LED lamp for 10 h. [b]Yields determined by ^1H NMR using 1,3,5-trimethoxybenzene as an internal standard.

Supplementary Table 4. Screening of hydrogen donor reagents[a]

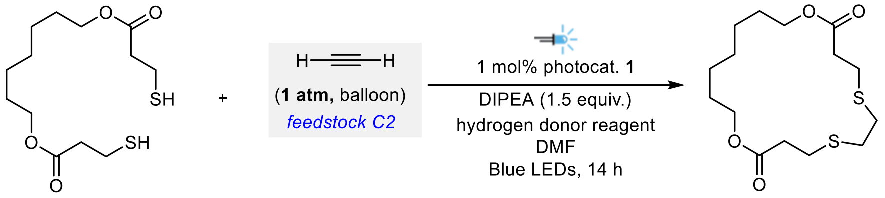

Entry	hydrogen donor reagent (1 equiv.)	Yield (%)[b]	photocat. **1**
1	hantzsch ester	trace	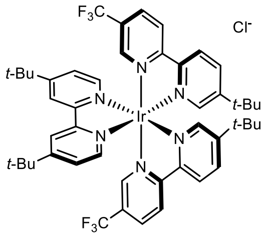
2	cyclohexadiene	ND	
3	EtOH	20	
4	hexafluorisopropanol	trace	
5	2,4,6-triisopropylbenzenethiol	ND	

[a]Reaction conditions: heptane-1,7-diyl bis(3-mercaptopropanoate) (0.1 mmol), acetylene gas (a balloon was filled with acetylene gas until its size was roughly 5 cm in diameter), DIPEA (1.5 equiv.), and photocatalyst (1 mol%) in degassed solvent and irradiated with a 10 W blue LED lamp for 14 h. [b]Yields determined by ^{1}H NMR using 1,3,5-trimethoxybenzene as an internal standard.

Supplementary Table 5. The effect of the radical-initiation method on the acetylene-enabled macrocyclization[a]

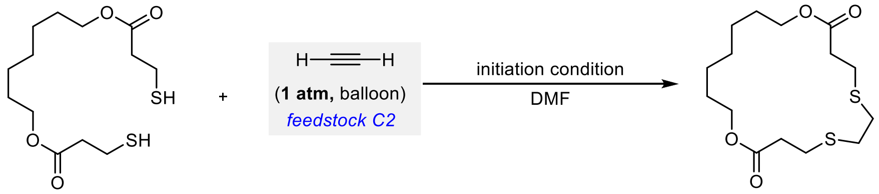

Entry	Initiation condition	Yield (%)[b]	photocat. **1**
1	(AIBN 10%), 80 °C	14	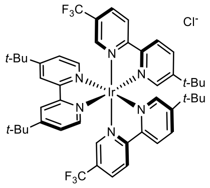
2	(TBHP 1 eq., CuI 10%), r.t.	ND	
3	(DTBP 1 eq., CuI 10%), r.t.	ND	
4	UV irradiation (l< 300 nm)	trace	

[a]Reaction conditions: heptane-1,7-diyl bis(3-mercaptopropanoate) (0.1 mmol), acetylene gas (a balloon was filled with acetylene gas until its size was roughly 5 cm in diameter). TBHP (~ 5.5 mol/L in decane(over molecular sieve 4 Å) in degassed solvent and reaction for 10 h. [b]Yields determined by ^{1}H NMR using 1,3,5-trimethoxybenzene as an internal standard.

Supplementary Table 6. The effect of the photocatalysts on the acetylene-enabled macrocyclization[a]

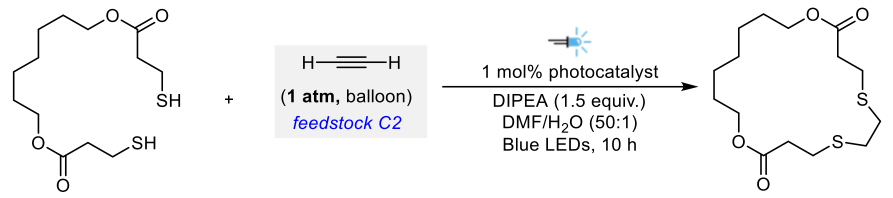

Entry	photocatalyst	Yield (%)[c]
1	[Ir(d(*t*Bu)(CF_3)ppy)$_2$(dtbbpy)](Cl)	29
2	[Ir(bpy)$_2$(dtbbpy)](PF_6)	23
3	*fac*-Ir(ppy)$_3$	ND
4	$MesAcr^+ClO_4^-$	17
5	TPT	ND
6	DPZ[b]	ND
7	Eosin Y	18
8	no	ND

[a]Reaction conditions: heptane-1,7-diyl bis(3-mercaptopropanoate) (0.1 mmol), acetylene gas (a balloon was filled with acetylene gas until its size was roughly 5 cm in diameter), DIPEA (1.5 equiv.), and photocatalyst (1 mol%) in degassed solvent and irradiated with a 10 W blue LED lamp for 10 h. [b]We gratefully acknowledge Prof. Zhiyong Jiang (Henan Normal University) for providing the photocatlyst PDZ. [c]Yields determined by ^{1}H NMR using 1,3,5-trimethoxybenzene as an internal standard.

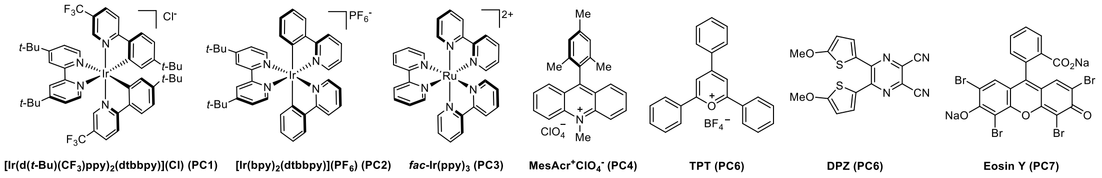

Supplementary Table 7. The effect of concentration on the acetylene-enabled macrocyclization[a]

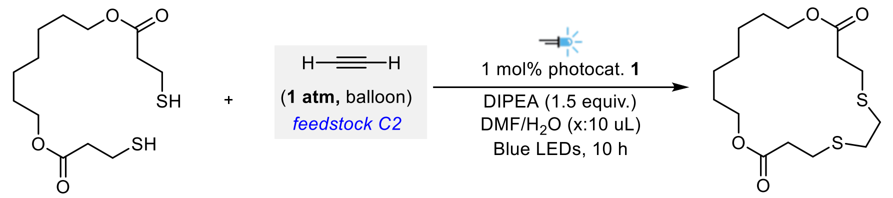

Entry	DMF/H_2O	concentration	Yield (%)[b]	photocat. **1**
1	50/1	0.025 M	30	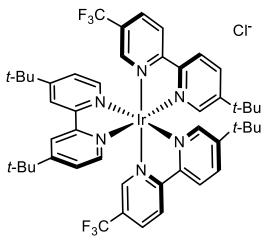
2	50/1	0.05 M	29	
3	50/1	0.075 M	26	
4	50/1	0.1 M	28	
6	25/1	0.05 M	23	
7	100/1	0.05 M	24	

[a]Reaction conditions: heptane-1,7-diyl bis(3-mercaptopropanoate) (0.1 mmol), acetylene gas, DIPEA (1.5 equiv.), and photocatalyst (1 mol%) in degassed solvent and irradiated with a 10 W blue LED lamp for 10 h. [b]Yields determined by ^{1}H NMR using 1,3,5-trimethoxybenzene as an internal standard.

4. General Procedures and Characterization Data of Substrates

General procedure A:

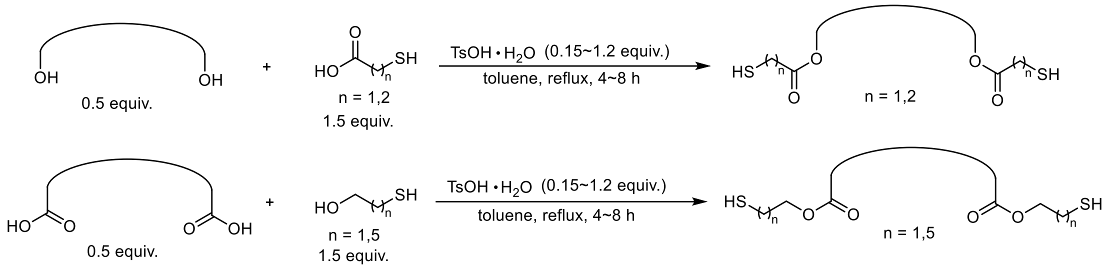

Following reported procedure with slight modification[2], a 50 mL round-bottom flask equipped with a Teflon-coated magnetic stir bar was charged with linear diol or linear carboxylic acid (0.5 equiv.), mercaptan carboxylic acid or mercaptoethanol (1.5 equiv.), *p*-toluenesulfonic acid monohydrate (0.15 ~ 1.2 equiv.), and toluene (35 mL). Then the flask equipped with graham condenser and Dean Stark for collecting water. The reaction mixture was then connected to a Schlenk line degas via vacuum evacuation, backfilled with nitrogen, and warmed to reflux temperature. After refluxed in toluene for 3 ~ 8 h, the mixture was poured into saturated $NaHCO_3$ solution followed by extracting with ethyl acetate. After washing with 10% HCl and saturated NaCl solution, the organic layer was dried over anhydrous $MgSO_4$. The mixture was filtered to remove the solid residues. The filtrate was concentrated in vacuo with the aid of a rotary evaporator. The residue was purified by flash column chromatography with silica gel mainly using petroleum ether and ethyl acetate as an eluent to afford the linear disulfide precursors.

General procedure B:

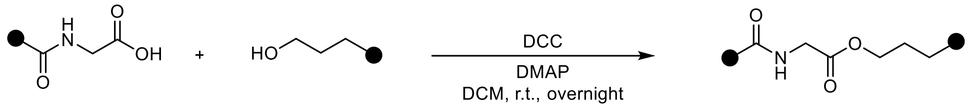

According to the reported procedure with slight modification[3], an oven-dried screw-capped Schlenk tube (50 mL) equipped with a Teflon-coated magnetic stir bar were added the corresponding carboxylic acid (5 mmol, 1.0 equiv.), alcohols (5 mmol, 1.0 equiv.) *N*,*N'*-dicyclohexylcarbodiimide (1.03 g, 5 mmol, 1.0 equiv.), 4-dimethylaminopyridine (32 mg, 0.2 mmol, 4 mol %), and dry dichloromethane (35 mL) under N_2 atmosphere. The mixture was allowed to stir until all the acid was consumed. The mixture was filtered through a silica gel plug, eluting with DCM, and the resulting filtrate concentrated in vacuo. The residue was purified by chromatography on silica gel, eluting with petroleum ether and ethyl acetate to give the corresponding amides.

General procedure C:

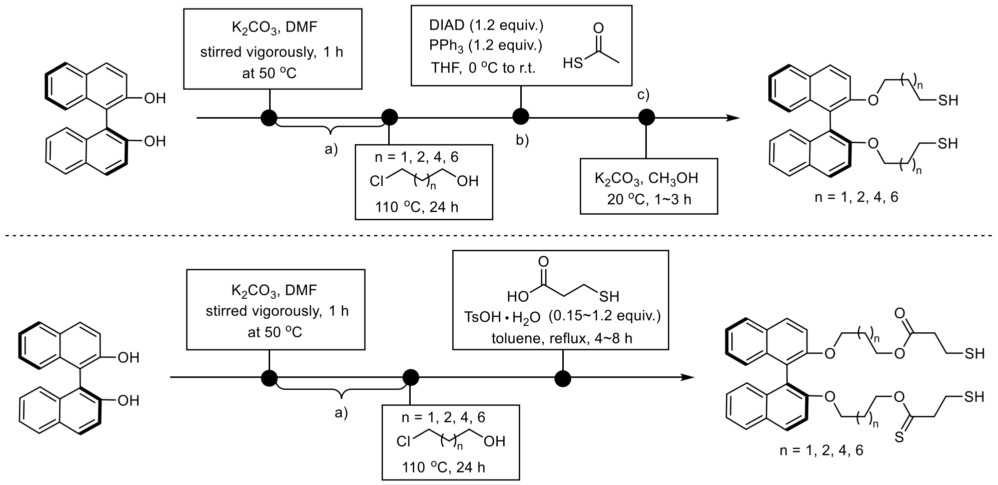

Step1: According to the reported procedure,[4] an oven-dried screw-capped Schlenk tube containing a stir bar was charged under nitrogen with the corresponding (*S*)-(+)-1,1'-Bi(2-naphthol) (5 mmol, 1.0 equiv.), anhydrous DMF (60 mL) and potassium carbonate (20 mmol, 4.0 equiv.) were added. After addition, the mixture was stirred at 50 °C for 1 h. Subsequently, halogenated alcohol (20 mmol, 4.0 equiv.) was added dropwise, and stirred continually for 24 h under refluxing (110 °C). After completing, the reaction was cooling to room temperature. Then, the reaction was filtered through a pad of silica gel (about a centimeter tall) and rinsed with more CH_2Cl_2 (3 × 5 mL). The filtrate was diluted with H_2O (45 mL) and extracted by DCM (3 × 20 mL), followed by concentrated under vacuum. The residue was purified by flash column chromatography with silica gel using ethyl acetate as an eluent to afford the title product.

Step2: The following procedure is adapted from the literature[5], to an oven dried round bottom flask equipped with a stir bar was added triphenylphosphine (15 mmol, 1.5 equiv.). The flask was evacuated and backfilled or continuously purged with argon, and then dry THF (30 mL) was added. The mixture was cooled to 0 °C, a yellow liquid of diisopropyl azodiformate (15 mmol, 1.5 equiv.) was added to an efficiently stirred solution and continue stirred at 0 °C for 30 min. The corresponding diols (10 mmol, 1.0 equiv.) and thiolacetic acid (15 mmol, 1.5 equiv.) in 20 mL of tetrahydrofuran was added dropwise over 15 min and the mixture was stirred for 1 h at 0 °C and at 22~25 °C for 1 h. A clear yellow solution resulted. Upon complete consumption, the resulting in a yellow precipitate mixture was filtered through a small pad of silica gel, washed with EtOAc (10 mL), and evaporated. Purification by column chromatography (Eluent: petroleum ether/ethyl acetate) afforded the title compound. Sometimes, the crude material was carried forward to next step reaction without further purification.

Step3: According to the literature procedure[6], an oven-dried Schlenk tube Schlenk flask was degassed, flushed with nitrogen, and charged with above *S*-(4-Cyanobenzyl) ethanethioate, CH_3OH (10 mL), potassium carbonate (1.2 equiv.). The reaction mixture was stirred at 20 °C until the ester was fully consumed by TLC. The reaction mixture was then neutralized with diluted hydrochloric acid to PH ~ 6, which then extracted with DCM, water, and then dried with Na_2SO_4, filtered and evaporated. Purification by column chromatography (Eluent: petroleum ether/ethyl acetate) afforded the title compound.

General procedure D:

Syntheses of Fenchyl alcohol derivatives

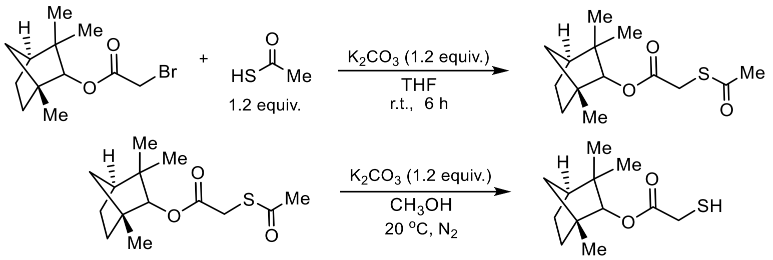

To a solution of 1,3,3-Trimethylbicyclo[2.2.1]heptan-2-yl 2-bromoacetate (1.38 g, 5 mmol, 1 equiv.) and potassium carbonate (829 mg, 6 mmol, 1.2 equiv.) in dry THF (15 mL), was added ethanethioic *S*-acid (457 mg, 6 mmol, 1.2 equiv.). The reaction mixture was stirred at room temperature for 6 h under nitrogen atmosphere. After the reaction was completed by TLC monitoring, the reaction mixture was evaporated in vacuo. The residue was extraction with DCM (3 × 10 mL), the combined organic layers washed with water, dried over anhydrous Na_2SO_4 and concentrated to dryness. The corresponding *S*-(4-Cyanobenzyl) ethanethioate was directly used in the next step without further purification. According to the literature procedure,[3] an oven-dried Schlenk tube Schlenk flask was degassed, flushed with argon, and charged with above *S*-(4-cyanobenzyl) ethanethioate, CH_3OH (10 mL), potassium carbonate (829 mg, 6 mmol, 1.2 equiv.). The reaction mixture was stirred at 20 °C until the *S*-(4-Cyanobenzyl) ethanethioate was fully consumed by TLC. The reaction mixture was then neutralized with diluted hydrochloric acid to ph ~ 6, which then extracted with DCM (3 × 10 mL), water (5 mL), and then dried (Na_2SO_4) filtered and evaporated. Purification by flash column chromatography on silica gel (Eluent: petroleum ether /EtOAc = 25/1) gave the title compound (856 mg, 75%) as a faint yellow oil.

1,3,3-Trimethylbicyclo[2.2.1]heptan-2-yl 2-mercaptoacetate (84a)

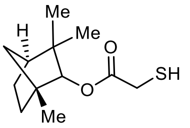

^{1}H NMR (400 MHz, $CDCl_3$) δ 4.39 (d, J = 1.2 Hz, 1H), 3.30 (d, J = 8.2 Hz, 2H), 1.99 (t, J = 8.2 Hz, 1H), 1.80 – 1.71 (m, 3H), 1.71 – 1.66 (m, 1H), 1.59 (d, J = 10.4 Hz, 1H), 1.46 (ddd, J = 17.2, 8.0, 3.8 Hz, 1H), 1.20 (d, J = 10.4 Hz, 1H), 1.11 (s, 3H), 1.06 (s, 3H), 0.81 (s, 3H); **^{13}C NMR** (101 MHz, $CDCl_3$) δ 171.3, 87.5, 48.4, 48.3, 41.3, 39.6, 29.7, 26.6, 26.5, 25.8, 20.2, 19.4; **HRMS** (ESI) calcd. for $C_{12}H_{20}O_2SNa$ $[M+Na]^+$ m/z 251.1082, found 251.1093.

Syntheses of (3*R*)-Cholestanethiol

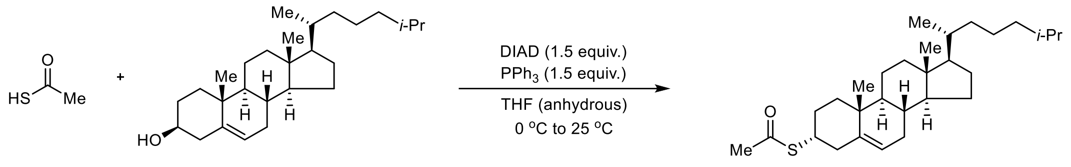

According to the literature procedure,[5] to an oven dried round bottom flask equipped with a stir bar was added triphenylphosphine (3.93 g, 15 mmol, 1.5 equiv.). The flask was evacuated and backfilled or continuously purged with argon, and then dry THF (30 mL) was added. The mixture was cooled

to 0 °C (in an ice/water bath), a yellow liquid of diisopropyl azodiformate (3.03 g, 15 mmol, 1.5 equiv.) was added to an efficiently stirred solution and continue stirred at 0 °C for 30 min. A white precipitate cholesterol (3.87 g, 10 mmol, 1.0 equiv.) and thiolacetic acid (1.14 g, 15 mmol, 1.5 equiv.) in 20 mL of tetrahydrofuran was added dropwise over 15 min and the mixture was stirred for 1 h at 0 °C and at 22 ~ 25°C for 1 h. A clear yellow solution resulted. Purification by flash column chromatography on silica gel (Eluent: petroleum ether /EtOAc = 100/1) gave the title compound (3.87 g, 90 %) as a yellow solid.

***S*-((3*R*,8*S*,9*S*,10*R*,13*R*,14*S*,17*R*)-10,13-dimethyl-17-((*R*)-6-methylheptan-2-yl) 2,3,4,7,8,9,10, 11,12,13,14,15,16,17-tetradecahydro-1*H*-cyclopenta[*a*]phenanthren-3-yl) ethanethioate (85a)**

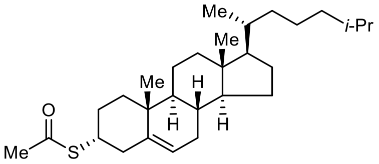

^{1}H NMR (400 MHz, $CDCl_3$) δ 5.32 (d, *J* = 2.9 Hz, 1H), 4.00 (s, 1H), 2.77 (d, *J* = 14.5 Hz, 1H), 2.29 (s, 3H), 2.17 – 1.92 (m, 4H), 1.91 – 1.77 (m, 1H), 1.71 (d, *J* = 12.1 Hz, 2H), 1.66 – 1.21 (m, 10H), 1.21 – 1.07 (m, 5H), 1.07 – 0.96 (m, 5H), 0.92 (d, *J* = 6.3 Hz, 3H), 0.87 (d, *J* = 6.6 Hz, 6H), 0.68 (s, 3H); **^{3}C NMR** (101 MHz, $CDCl_3$) δ 195.7, 195.7, 139.1, 122.6, 56.7, 56.2, 50.2, 43.2, 42.3, 39.8, 39.5, 37.7, 37.2, 36.2, 35.8, 35.6, 31.8, 31.7, 31.0, 28.3, 28.0, 27.6, 24.3, 23.9, 22.8, 22.6, 20.8, 19.2, 18.7, 11.9.

(3*R*)-Cholestanethiol (85b)

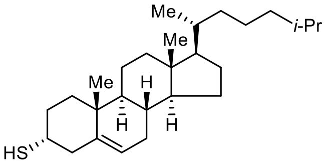

S-((3*R*,8*S*,9*S*,10*R*,13*R*,14*S*,17*R*)-10,13-dimethyl-17-((*R*)-6-methylheptan-2-yl)-2,3,4,7,8,9,10,11, 12,13,14,15,16,17-tetradecahydro-1H-cyclopenta[a]phenanthren-3-yl) (3.87 g, 9.0 mmol, 1 equiv.) was dissolved in 20 mL of anhydrous ether and added dropwise to a suspension of lithium aluminum hydride (1.37 g, 4.0 equiv.) in 25 ml of anhydrous ether under a nitrogen atmosphere. The reaction mixture was stirred at room temperature for 30 min and the excess lithium aluminum hydride was destroyed by the careful addition of 10 mL of 1N hydrochloric acid solution. The reaction mixture was then extracted with ether (3 × 20 mL), water (10 mL), and then dried (Na_2SO_4) filtered and evaporated to give (3.33 g 8.3 mmol) of (3*R*)-cholestanethiol in 92% yield as a white solid, which was employed without further purification for the next step.
^{1}H NMR (400 MHz, $CDCl_3$) δ 5.37 – 5.20 (m, 1H), 3.32 (s, 1H), 2.74 (d, *J* = 12.9 Hz, 1H), 2.07 – 1.86 (m, 4H), 1.77 (ddd, *J* = 19.0, 9.3, 5.1 Hz, 1H), 1.63 – 1.40 (m, 8H), 1.37 – 1.14 (m, 6H), 1.12 – 0.89 (m, 12H), 0.85 (d, *J* = 6.5 Hz, 3H), 0.80 (dd, *J* = 6.6, 1.2 Hz, 6H), 0.61 (s, 3H); **^{13}C NMR** (101 MHz, $CDCl_3$) δ 137.9, 124.0, 56.8, 56.2, 50.3, 42.3, 40.7, 39.8, 39.6, 37.9, 37.3, 36.2, 35.9, 33.1, 31.9, 31.8, 30.2, 28.3, 28.0, 24.3, 23.9, 22.9, 22.6, 20.8, 19.2, 18.8, 11.9. The analytical datas are in agreement with the literature.[5]

Syntheses of TRIP

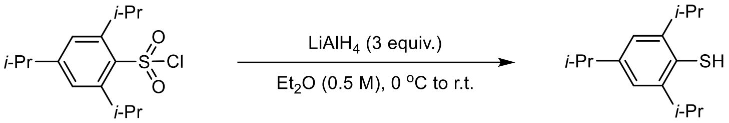

2,4,6- Triisopropylbezene-1-thiol (generally shortened to **TRIP** thiol) was synthesized according to the reported procedure,[7] to a dry 50-mL round bottom flask, lithium aluminum hydride ($LiAlH_4$) (0.80 g, 20.0 mmol, 2.0 equiv.) was added. Anhydrous Et_2O (20 mL) was then added to form a suspension and cooled to -6 °C. To this mixture, a solution of 2,4,6-triisopropylbenzene-1-sulfonyl chloride (3.02 g, 10 mmol, 1.0 equiv.) in Et_2O (15 mL) was added slowly. After completion of addition, an additional load of $LiAlH_4$ (0.38 g, 10 mmol, 1.0 equiv.) was added. The reaction was allowed to warm to rt as it was stirred overnight. Upon completion detected by TLC, the reaction was cooled to 0 °C and diluted with 20 mL Et_2O. The reaction was quenched with water (1 mL per gram of $LiAlH_4$), 15% (w/w) NaOH solution (1 mL per gram of $LiAlH_4$) and an additional water added until the resulting white slurry becomes clear under conditions of effective stir. The mixture was dried over $MgSO_4$ and filtered through a short silica gel pad and washed with Et_2O. The light yellow liquid was then concentrated and distilled at reduced pressure (-10 mbar) to provide 1.65 g of 2,4,6- triisopropylbezene-1-thiol (7 mmol, 70% yield) as a colorless oil.

2,4,6-Triisopropylbenzenethiol (87a)

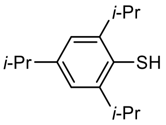

^{1}H NMR (400 MHz, $CDCl_3$) δ 7.11 (s, 2H), 3.68 – 3.54 (m, 2H), 3.16 (s, 1H), 2.97 (dt, J = 13.6, 6.8 Hz, 1H), 1.39 – 1.30 (m, 18H); **^{13}C NMR** (101 MHz, $CDCl_3$) δ 148.2, 147.2, 124.4, 121.4, 34.2, 31.9, 24.1, 23.3. The analytical datas are in agreement with the literature.[7]

Ethane-1,2-diyl bis(3-mercaptopropanoate) (5a)

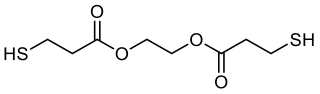

Prepared according to the general procedure **A**, ethane-1,2-diol (2.5 mmol, 1.0 equiv.), 3-mercaptopropanoic acid (7.5 mmol, 3.0 equiv.), *p*-toluenesulfonic acid monohydrate (0.5 mmol, 0.2 equiv.) were used. Purification by column chromatography (Eluent: petroleum ether/ethyl acetate = 6/1) afforded the title compound as a colorless oil (411 mg, 69% yield).

^{1}H NMR (500 MHz, $CDCl_3$) δ 4.27 (s, 4H), 2.75 – 2.69 (m, 4H), 2.62 (t, J = 6.6 Hz, 4H), 1.60 (t, J = 8.3 Hz, 2H); **^{13}C NMR** (126 MHz, $CDCl_3$) δ 171.3, 62.3, 38.3, 19.6; **IR** (KBr, cm^{-1}) 3296, 2880, 1738, 1515, 1263, 742; **HRMS** (ESI) calcd. For $C_8H_{14}NaO_4S_2^+$ $[M+Na]^+$ m/z 261.0226, found: 261.0222.

Propane-1,3-diyl bis(3-mercaptopropanoate) (6a)

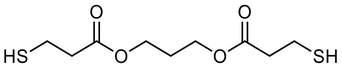

Prepared according to the general procedure **A**, propane-1,3-diol (2.5 mmol, 1.0 equiv.), 3-mercaptopropanoic acid (7.5 mmol, 3.0 equiv.), *p*-toluenesulfonic acid monohydrate (0.5 mmol, 0.2

equiv.) were used. Purification by column chromatography (Eluent: petroleum ether/ethyl acetate = 10/1) afforded the title compound as a colorless oil (510 mg, 81% yield).

^{1}H NMR (500 MHz, $CDCl_3$) δ 4.14 (t, *J* = 6.3 Hz, 4H), 2.71 (dd, *J* = 15.0, 6.7 Hz, 4H), 2.59 (t, *J* = 6.8 Hz, 4H), 2.04 – 1.88 (m, 2H), 1.58 (t, *J* = 8.3 Hz, 2H); **^{13}C NMR** (126 MHz, $CDCl_3$) δ 171.5, 61.2, 38.4, 27.9, 19.7; **IR** (KBr, cm^{-1}) 3295, 2896, 1733, 1507, 1195; **HRMS** (ESI) calcd. For $C_9H_{16}NaO_4S_2^+$ $[M+Na]^+$ m/z 275.0382, found: 275.0387.

2-Methylpropane-1,3-diyl bis(3-mercaptopropanoate) (7a)

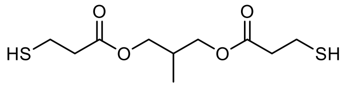

Prepared according to the general procedure **A**, 2-methylpropane-1,3-diol (2.5 mmol, 1.0 equiv.), 3-mercaptopropanoic acid (7.5 mmol, 3.0 equiv.), *p*-toluenesulfonic acid monohydrate (0.5 mmol, 0.2 equiv.) were used. Purification by column chromatography (Eluent: petroleum ether/ethyl acetate = 10/1) afforded the title compound as a colorless oil (546 mg, 82% yield).

^{1}H NMR (500 MHz, $CDCl_3$) δ 4.00 (d, *J* = 6.0 Hz, 4H), 2.71 (dd, *J* = 14.5, 7.3 Hz, 4H), 2.60 (t, *J* = 6.8 Hz, 4H), 2.13 (dq, *J* = 12.9, 6.4 Hz, 1H), 1.57 (t, *J* = 8.3 Hz, 2H), 0.94 (d, *J* = 6.9 Hz, 3H); **^{13}C NMR** (126 MHz, $CDCl_3$) δ 170.5, 64.9, 37.4, 31.4, 18.7, 12.8; **IR** (KBr, cm^{-1}) 2963, 1734, 1376, 1155; **HRMS** (ESI) calcd. $C_{10}H_{18}NaO_4S_2^+$ $[M+Na]^+$ m/z 289.0539, found: 289.0535.

(±)-Pentane-2,4-diyl bis(3-mercaptopropanoate) (8a)

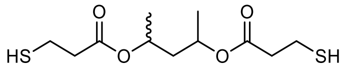

Prepared according to the general procedure **A**, (±)-pentane-2,4-diyl biol (2.5 mmol, 1.0 equiv.), 3-mercaptopropanoic acid (7.5 mmol, 3.0 equiv.), *p*-toluenesulfonic acid monohydrate (0.5 mmol, 0.2 equiv.) were used. Purification by column chromatography (Eluent: petroleum ether/ethyl acetate = 9/1) afforded the title compound as a colorless oil (602 mg, 86% yield).

^{1}H NMR (500 MHz, $CDCl_3$) δ 5.43 – 4.59 (m, 2H), 2.76 (dd, *J* = 14.9, 6.8 Hz, 4H), 2.67 – 2.55 (m, 4H), 1.80 – 1.75 (m, 2H), 1.65 (t, *J* = 8.4 Hz, 2H), 1.25 (s, 3H), 1.24 (s, 3H); **^{13}C NMR** (126 MHz, $CDCl_3$) δ 171.1, 67.5, 42.3, 38.7, 20.5, 19.8; **IR** (KBr, cm^{-1}) 2957, 1738, 1396, 1137; **HRMS** (ESI) calcd. For $C_{11}H_{20}NaO_4S_2^+$ $[M+Na]^+$ m/z 303.0695, found:303.0692.

2,2-Dimethylpropane-1,3-diyl bis(3-mercaptopropanoate) (9a)

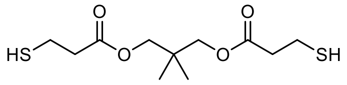

Prepared according to the general procedure **A**, 2,2-dimethylpropane-1,3-diol (2.5 mmol, 1.0 equiv.), 3-mercaptopropanoic acid (7.5 mmol, 3.0 equiv.), *p*-toluenesulfonic acid monohydrate (0.5 mmol, 0.2 equiv.) were used. Purification by column chromatography (Eluent: petroleum ether/ethyl acetate = 9/1) afforded the title compound as a colorless oil (553 mg, 79% yield).

^{1}H NMR (500 MHz, $CDCl_3$) δ 3.87 (d, *J* = 3.4 Hz, 4H), 2.75 – 2.68 (m, 4H), 2.64 – 2.58 (m, 4H), 1.61 – 1.52 (m, 2H), 0.92 (d, *J* = 3.5 Hz, 6H); **^{13}C NMR** (126 MHz, $CDCl_3$) δ 171.4, 69.2, 38.4, 34.7, 21.8, 19.7; **IR** (KBr, cm^{-1}) 3195, 2969, 1728, 1438, 1095; **HRMS** (ESI) calcd. For $C_{11}H_{20}NaO_4S_2^+$ $[M+Na]^+$ m/z 303.0695, found: 303.0692.

Butane-1,4-diyl bis(3-mercaptopropanoate) (10a)

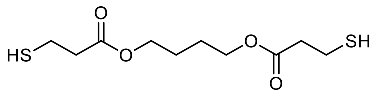

Prepared according to the general procedure **A**, butane-1,4-diol (2.5 mmol, 1.0 equiv.), 3-mercaptopropanoic acid (7.5 mmol, 3.0 equiv.), *p*-toluenesulfonic acid monohydrate (0.5 mmol, 0.2 equiv.) were used. Purification by column chromatography (Eluent: petroleum ether/ethyl acetate = 9/1) afforded the title compound as a colorless oil (586 mg, 88% yield).

^{1}H NMR (500 MHz, $CDCl_3$) δ 4.15 (s, 4H), 2.78 (dd, *J* = 15.0, 6.8 Hz, 4H), 2.65 (t, *J* = 6.8 Hz, 4H), 1.74 (s, 4H), 1.64 (t, *J* = 8.3 Hz, 2H); **^{13}C NMR** (126 MHz, $CDCl_3$) δ 171.5, 64.1, 38.4, 25.3, 19.7; **IR** (KBr, cm^{-1}) 3298, 2894, 1740, 1506, 1265; **HRMS** (ESI) calcd. For $C_{10}H_{18}O_4NaS_2^+$ $[M+Na]^+$ m/z 289.0539, found: 289.0540.

Pentane-1,5-diyl bis(3-mercaptopropanoate) (11a)

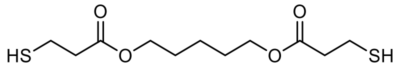

Prepared according to the general procedure **A**, pentane-1,5-diol (2.5 mmol, 1.0 equiv.), 3-mercaptopropanoic acid (7.5 mmol, 3.0 equiv.), *p*-toluenesulfonic acid monohydrate (0.5 mmol, 0.2 equiv.) were used. Purification by column chromatography (Eluent: petroleum ether/ethyl acetate = 10/1) afforded the title compound as a colorless oil (602 mg, 86% yield).

^{1}H NMR (500 MHz, $CDCl_3$) δ 4.05 (t, *J* = 6.6 Hz, 4H), 2.71 (dd, *J* = 14.4, 7.5 Hz, 4H), 2.59 (t, *J* = 6.7 Hz, 4H), 1.67 – 1.55 (m, 6H), 1.46 – 1.29 (m, 2H); **^{13}C NMR** (126 MHz, $CDCl_3$) δ 171.6, 64.5, 38.5, 28.2, 22.5, 19.8; **IR** (KBr, cm^{-1}) 3132, 2921, 2852, 1731, 1629, 1399, 1131; **HRMS** (ESI) calcd. For $C_{11}H_{20}NaO_4S_2^+$ $[M+Na]^+$ m/z 303.0695, found: 303.0693.

Hexane-1,6-diyl bis(3-mercaptopropanoate) (12a)

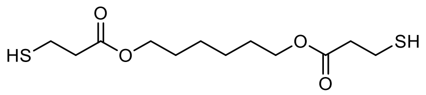

Prepared according to the general procedure **A**, hexane-1,6-diol (2.5 mmol, 1.0 equiv.), 3-mercaptopropanoic acid (7.5 mmol, 3.0 equiv.), *p*-toluenesulfonic acid monohydrate (0.5 mmol, 0.2 equiv.) were used. Purification by column chromatography (Eluent: petroleum ether/ethyl acetate = 10/1) afforded the title compound as a colorless oil (676 mg, 92% yield).

^{1}H NMR (500 MHz, $CDCl_3$) δ 4.04 (t, *J* = 6.6 Hz, 4H), 2.71 (dd, *J* = 14.6, 7.4 Hz, 4H), 2.58 (t, *J* = 6.7 Hz, 4H), 1.62 – 1.54 (m, 6H), 1.38 – 1.27 (m, 4H); **^{13}C NMR** (126 MHz, $CDCl_3$) δ 171.6, 64.6, 38.5, 28.5, 25.6, 19.8; **IR** (KBr, cm^{-1}) 3138, 2927, 1731, 1394, 1157; **HRMS** (ESI) calcd. For $C_{12}H_{22}NaO_4S_2^+$ $[M+Na]^+$ m/z 317.0852, found: 317.0847.

Heptane-1,7-diyl bis(3-mercaptopropanoate) (14a)

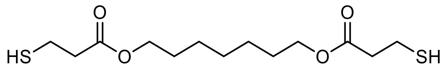

Prepared according to the general procedure **A**, heptane-1,7-diol (2.5 mmol, 1.0 equiv.), 3-mercaptopropanoic acid (7.5 mmol, 3.0 equiv.), *p*-toluenesulfonic acid monohydrate (0.5 mmol, 0.2 equiv.) were used. Purification by column chromatography (Eluent: petroleum ether/ethyl acetate = 11/1) afforded the title compound as a colorless oil (701 mg, 91% yield).

^{1}H NMR (500 MHz, $CDCl_3$) δ 4.11 (t, *J* = 6.7 Hz, 4H), 2.78 (dd, *J* = 15.0, 6.9 Hz, 4H), 2.65 (t, *J* = 6.8 Hz, 4H), 1.68 – 1.58 (m, 6H), 1.36 (s, 6H); **^{13}C NMR** (126 MHz, $CDCl_3$) δ 171.7, 64.7, 38.5,

28.8, 28.5, 25.7, 19.8; **IR** (KBr, cm^{-1}) 2935, 1733, 1439, 1351, 1161; **HRMS** (ESI) calcd. For $C_{13}H_{24}NaO_4S_2^+$ $[M+Na]^+$ m/z 331.1008, found: 331.1006.

Octane-1,8-diyl bis(3-mercaptopropanoate) (15a)

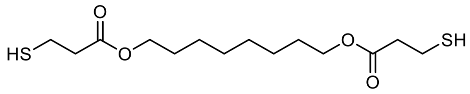

Prepared according to the general procedure **A**, octane-1,8-diol (2.5 mmol, 1.0 equiv.), 3-mercaptopropanoic acid (7.5 mmol, 3.0 equiv.), *p*-toluenesulfonic acid monohydrate (0.5 mmol, 0.2 equiv.) were used. Purification by column chromatography (Eluent: petroleum ether/ethyl acetate = 10/1) afforded the title compound as a colorless oil (692 mg, 86% yield).
^{1}H NMR (500 MHz, $CDCl_3$) δ 4.10 (t, *J* = 6.7 Hz, 4H), 2.78 (dd, *J* = 14.6, 7.4 Hz, 4H), 2.65 (t, *J* = 6.8 Hz, 4H), 1.70 – 1.56 (m, 6H), 1.33 (s, 8H); **^{13}C NMR** (126 MHz, $CDCl_3$) δ 171.7, 64.8, 38.5, 29.0, 28.5, 25.8, 19.8; **IR** (KBr, cm^{-1}) 2931, 1733, 1443, 1160; **HRMS** (ESI) calcd. For $C_{14}H_{26}NaO_4S_2^+$ $[M+Na]^+$ m/z 345.1165, found: 345.1164.

Nonane-1,9-diyl bis(3-mercaptopropanoate) (16a)

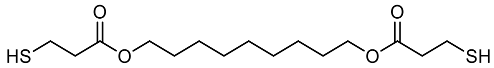

Prepared according to the general procedure **A**, nonane-1,9-diol (2.5 mmol, 1.0 equiv.), 3-mercaptopropanoic acid (7.5 mmol, 3.0 equiv.), *p*-toluenesulfonic acid monohydrate (0.5 mmol, 0.2 equiv.) were used. Purification by column chromatography (Eluent: petroleum ether/ethyl acetate = 12/1) afforded the title compound as a colorless oil (773 mg, 82% yield).
^{1}H NMR (500 MHz, $CDCl_3$) δ 4.03 (t, *J* = 6.7 Hz, 4H), 2.71 (dd, *J* = 14.9, 7.0 Hz, 4H), 2.58 (t, *J* = 6.8 Hz, 4H), 1.68 – 1.49 (m, 6H), 1.33 – 1.17 (m, 10H); **^{13}C NMR** (126 MHz, $CDCl_3$) δ 171.7, 64.8, 38.5, 29.3, 29.1, 28.6, 25.9, 19.8; **IR** (KBr, cm^{-1}) 3139, 2923, 1960, 1734, 1578, 1400, 1147; **HRMS** (ESI) calcd. For $C_{15}H_{28}NaO_4S_2^+$ $[M+Na]^+$ m/z 359.1321, found: 359.1320.

Decane-1,10-diyl bis(3-mercaptopropanoate) (17a)

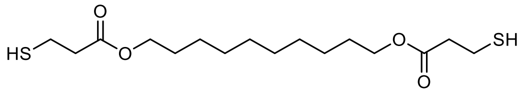

Prepared according to the general procedure **A**, decane-1,10-diol (2.5 mmol, 1.0 equiv.), 3-mercaptopropanoic acid (7.5 mmol, 3.0 equiv.), *p*-toluenesulfonic acid monohydrate (0.5 mmol, 0.2 equiv.) were used. Purification by column chromatography (Eluent: petroleum ether/ethyl acetate = 10/1) afforded the title compound as a colorless oil (692 mg, 79% yield).
^{1}H NMR (500 MHz, $CDCl_3$) δ 4.03 (t, *J* = 6.7 Hz, 4H), 2.71 (dd, *J* = 14.5, 7.4 Hz, 4H), 2.58 (t, *J* = 6.8 Hz, 4H), 1.61 – 1.51 (m, 6H), 1.32 – 1.16 (m, 12H); **^{13}C NMR** (126 MHz, $CDCl_3$) δ 171.7, 64.9, 38.5, 29.4, 29.2, 28.6, 25.9, 19.8; **IR** (KBr, cm^{-1}) 2919, 1747, 1489, 1338, 1269; **HRMS** (ESI) calcd. For $C_{16}H_{30}NaO_4S_2^+$ $[M+Na]^+$ m/z 373.1478, found: 373.1479.

Dodecane-1,12-diyl bis(3-mercaptopropanoate) (18a)

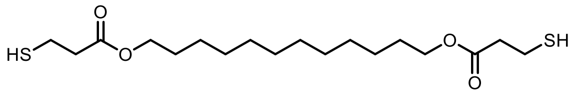

Prepared according to the general procedure **A**, dodecane-1,12-diol (2.5 mmol, 1.0 equiv.), 3-

mercaptopropanoic acid (7.5 mmol, 3.0 equiv.), *p*-toluenesulfonic acid monohydrate (0.5 mmol, 0.2 equiv.) were used. Purification by column chromatography (Eluent: petroleum ether/ethyl acetate = 10/1) afforded the title compound as a colorless oil (786 mg, 83% yield).
^{1}H NMR (500 MHz, $CDCl_3$) δ 4.10 (t, *J* = 6.7 Hz, 4H), 2.77 (dd, *J* = 14.9, 7.0 Hz, 4H), 2.65 (t, *J* = 6.8 Hz, 4H), 1.67 – 1.59 (m, 6H), 1.38 – 1.24 (m, 16H); **^{13}C NMR** (126 MHz, $CDCl_3$) δ 171.7, 64.9, 38.5, 29.4, 29.2, 28.6, 25.9, 19.8; **IR** (KBr, cm^{-1}) 2940, 1734, 1441, 1267; **HRMS** (ESI) calcd. For $C_{18}H_{34}NaO_4S_2^+$ $[M+Na]^+$ m/z 401.1791, found: 401.1792.

Tetradecane-1,14-diyl bis(3-mercaptopropanoate) (19a)

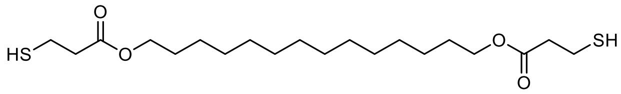

Prepared according to the general procedure **A**, tetradecane-1,14-diol (2.5 mmol, 1.0 equiv.), 3-mercaptopropanoic acid (7.5 mmol, 3.0 equiv.), *p*-toluenesulfonic acid monohydrate (0.5 mmol, 0.2 equiv.) were used. Purification by column chromatography (Eluent: petroleum ether/ethyl acetate = 10/1) afforded the title compound as a colorless oil (905 mg, 89% yield).
^{1}H NMR (500 MHz, $CDCl_3$) δ 4.10 (t, *J* = 6.7 Hz, 4H), 2.77 (dd, *J* = 14.5, 7.2 Hz, 4H), 2.64 (t, *J* = 6.8 Hz, 4H), 1.70 – 1.54 (m, 6H), 1.47 – 1.11 (m, 20H); **^{13}C NMR** (126 MHz, $CDCl_3$) δ 171.6, 64.8, 38.5, 29.5, 29.5, 29.5, 29.2, 28.6, 25.9, 19.8; **IR** (KBr, cm^{-1}) 2945, 1740, 1541, 1338; **HRMS** (ESI) calcd. For $C_{20}H_{38}NaO_4S_2^+$ $[M+Na]^+$ m/z 429.2104, found: 429.2105.

Bis(6-mercaptohexyl) 2,2'-oxydiacetate (22a)

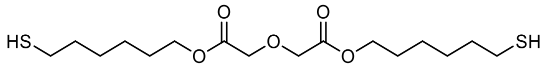

Prepared according to the general procedure **A**, 2,2'-oxydiacetic acid (2.5 mmol, 1.0 equiv.), 6-mercaptohexan-1-ol (7.5 mmol, 3.0 equiv.), *p*-toluenesulfonic acid monohydrate (0.5 mmol, 0.2 equiv.) were used. Purification by column chromatography (Eluent: petroleum ether/ethyl acetate = 8/1) afforded the title compound as a colorless oil (816 mg, 89% yield).
^{1}H NMR (500 MHz, $CDCl_3$) δ 4.28 – 4.21 (m, 4H), 4.16 (t, *J* = 6.5 Hz, 4H), 2.58 – 2.46 (m, 4H), 1.75 – 1.54 (m, 8H), 1.50 – 1.30 (m, 10H); **^{13}C NMR** (126 MHz, $CDCl_3$) δ 169.7, 68.1, 64.9, 33.7, 28.4, 27.8, 25.3, 24.4; **IR** (KBr, cm^{-1}) 2944, 1748, 1508, 1207, 1137; **HRMS** (ESI) calcd. For $C_{16}H_{30}NaO_5S_2^+$ $[M+Na]^+$ m/z 389.1427, found: 389.1430.

Bis(6-mercaptohexyl) 2,2,3,3-tetrafluorosuccinate (20a)

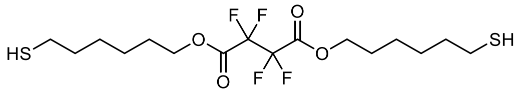

Prepared according to the general procedure **A**, 2,2,3,3-tetrafluorosuccinic acid (2.5 mmol, 1.0 equiv.), 6-mercaptohexan-1-ol (7.5 mmol, 3.0 equiv.), *p*-toluenesulfonic acid monohydrate (0.5 mmol, 0.2 equiv.) were used. Purification by column chromatography (Eluent: petroleum ether/ethyl acetate = 10/1) afforded the title compound as a colorless oil (929 mg, 88% yield).
^{1}H NMR (500 MHz, $CDCl_3$) δ 4.35 (t, *J* = 6.6 Hz, 4H), 2.53 (q, *J* = 7.4 Hz, 4H), 1.78 – 1.71 (m, 4H), 1.66 – 1.60 (m, 4H), 1.49 – 1.36 (m, 8H), 1.34 (t, *J* = 7.8 Hz, 2H); **^{13}C NMR** (126 MHz, $CDCl_3$) δ 159.4 (t, $^2J_{CF}$ = 30.2 Hz), 110.1 (t, $^2J_{CF}$ = 30.5 Hz), 108.0 (tt, $^1J_{CF}$ = 264.6 Hz, $^2J_{CF}$ = 32.8 Hz), 105.9 (t, $^2J_{CF}$ = 31.5 Hz), 68.0, 33.7, 28.0, 27.7, 25.0, 24.4; **^{19}F NMR** (471 MHz, $CDCl_3$) δ -119.9, -119.9; **IR** (KBr, cm^{-1}) 2932, 2860, 1774, 1458, 1316, 1100; **HRMS** (ESI) calcd. For

$C_{16}H_{26}F_4NaO_4S_2^+$ [M+Na]$^+$ m/z 445.1101, found: 445.1100.

Bis(6-mercaptohexyl) 2,2,3,3,4,4,5,5-octafluorohexanedioate (21a)

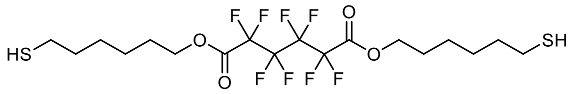

Prepared according to the general procedure **A**, 2,2,3,3,4,4,5,5-octafluorohexanedioic acid (2.5 mmol, 1.0 equiv.), 6-mercaptohexan-1-ol (7.5 mmol, 3.0 equiv.), *p*-toluenesulfonic acid monohydrate (0.5 mmol, 0.2 equiv.) were used. Purification by column chromatography (Eluent: petroleum ether/ethyl acetate = 10/1) afforded the title compound as a colorless oil (1.17 g, 90% yield).

^{1}H NMR (500 MHz, $CDCl_3$) δ 4.37 (t, *J* = 6.6 Hz, 4H), 2.53 (q, *J* = 7.4 Hz, 4H), 1.80 – 1.70 (m, 4H), 1.63 (p, *J* = 7.1 Hz, 4H), 1.48 – 1.37 (m, 8H), 1.34 (t, *J* = 7.8 Hz, 2H); **^{13}C NMR** (126 MHz, $CDCl_3$) δ 158.4 (td, $^2J_{CF}$ = 29.0 Hz, $^3J_{CF}$ = 2.5 Hz), 112.5 (t, $^2J_{CF}$ = 31.5 Hz), 110.4 (m), 108.1 (m), 105.8 (t, $^2J_{CF}$ = 31.5 Hz), 68.3, 33.7, 27.9, 27.6, 24.9 24.9, 24.3, 24.3; **^{19}F NMR** (471 MHz, $CDCl_3$) δ -118.7, -118.7, -122.7; **IR** (KBr, cm^{-1}) 2904, 1747, 1506, 1338, 1193; **HRMS** (ESI) calcd. For $C_{18}H_{26}F_8NaO_4S_2^+$ [M+Na]$^+$ m/z 545.1037, found: 545.1036.

***trans*-Bis(6-mercaptohexyl) cyclohexane-1,4-dicarboxylate (29a)**

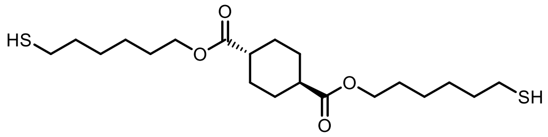

Prepared according to the general procedure **A**, cyclohexane-1,4-dicarboxylic acid (2.5 mmol, 1.0 equiv.), 6-mercaptohexan-1-ol (7.5 mmol, 3.0 equiv.), *p*-toluenesulfonic acid monohydrate (0.5 mmol, 0.2 equiv.) were used. Purification by column chromatography (Eluent: petroleum ether/ethyl acetate = 10/1) afforded the title compound as a colorless oil (839 mg, 83% yield).

^{1}H NMR (500 MHz, $CDCl_3$) δ 3.99 (t, *J* = 6.5 Hz, 4H), 2.46 (q, *J* = 7.3 Hz, 4H), 2.20 (s, 2H), 1.98 (d, *J* = 8.0 Hz, 4H), 1.61 – 1.49 (m, 8H), 1.44 – 1.24 (m, 14H); **^{13}C NMR** (126 MHz, $CDCl_3$) δ 175.4, 64.2, 42.5, 33.8, 28.5, 28.0, 27.9, 25.4, 24.5; **IR** (KBr, cm^{-1}) 2939, 1729, 1502, 1249, 738; **HRMS** (ESI) calcd. For $C_{20}H_{36}NaO_4S_2^+$ [M+Na]$^+$ m/z 427.1947, found: 427.1943.

Bis(6-mercaptohexyl) bicyclo[2.2.2]octane-1,4-dicarboxylate (30a)

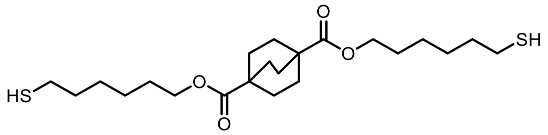

Prepared according to the general procedure **A**, bicyclo[2.2.2]octane-1,4-dicarboxylic acid (2.5 mmol, 1.0 equiv.), 6-mercaptohexan-1-ol (7.5 mmol, 3.0 equiv.), *p*-toluenesulfonic acid monohydrate (0.5 mmol, 0.2 equiv.) were used. Purification by column chromatography (Eluent: petroleum ether/ethyl acetate = 10/1) afforded the title compound as a colorless oil (711 mg, 66% yield).

^{1}H NMR (500 MHz, $CDCl_3$) δ 4.04 (t, *J* = 6.5 Hz, 4H), 2.53 (q, *J* = 7.4 Hz, 4H), 1.81 (s, 12H), 1.61 (q, *J* = 6.7 Hz, 8H), 1.42 (dt, *J* = 14.3, 7.0 Hz, 4H), 1.35 (dt, *J* = 15.3, 7.7 Hz, 6H); **^{13}C NMR** (126 MHz, $CDCl_3$) δ 177.44, 64.2, 38.7, 33.8, 28.5, 27.9, 27.8, 25.4, 24.5; **IR** (KBr, cm^{-1}) 2926, 1747, 1730, 1456, 1252, 1074; **HRMS** (ESI) calcd. For $C_{22}H_{38}NaO_4S_2^+$ [M+Na]$^+$ m/z 453.2104, found:

453.2105.

Bis(6-mercaptohexyl)-adamantane-1,3-dicarboxylate (31a)

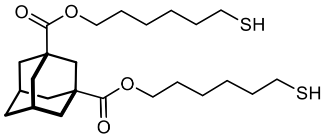

Prepared according to the general procedure **A**, adamantane-1,3-dicarboxylic acid (2.5 mmol, 1.0 equiv.), 6-mercaptohexan-1-ol (7.5 mmol, 3.0 equiv.), *p*-toluenesulfonic acid monohydrate (0.5 mmol, 0.2 equiv.) were used. Purification by column chromatography (Eluent: petroleum ether/ethyl acetate = 10/1) afforded the title compound as a colorless oil (912 mg, 80% yield).

^{1}H NMR (500 MHz, $CDCl_3$) δ 3.99 (t, *J* = 6.1 Hz, 4H), 2.61 – 2.36 (m, 4H), 2.09 (s, 2H), 1.94 (s, 2H), 1.79 (q, *J* = 12.5 Hz, 8H), 1.59 (d, *J* = 28.9 Hz, 10H), 1.32 (dq, *J* = 32.4, 7.4 Hz, 10H); **^{13}C NMR** (126 MHz, $CDCl_3$) δ 176.8, 64.2, 41.0, 39.8, 38.0, 35.4, 33.8, 28.5, 28.0, 27.8, 25.4, 24.5; **IR** (KBr, cm^{-1}) 3167, 2925, 1734, 1613, 1399, 1100; **HRMS** (ESI) calcd. For $C_{24}H_{40}NaO_4S_2^+$ $[M+Na]^+$ m/z 479.2260, found: 479.2256.

Oxybis(ethane-2,1-diyl) bis(3-mercaptopropanoate) (23a)

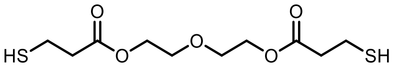

Prepared according to the general procedure **A**, 2,2'-oxybis(ethan-1-ol) (2.5 mmol, 1.0 equiv.), 3-mercaptopropanoic acid (7.5 mmol, 3.0 equiv.), *p*-toluenesulfonic acid monohydrate (0.5 mmol, 0.2 equiv.) were used. Purification by column chromatography (Eluent: petroleum ether/ethyl acetate = 4/1) afforded the title compound as a colorless oil (542 mg, 77% yield).

^{1}H NMR (500 MHz, $CDCl_3$) δ 4.28 (m, 4H), 3.71 (m, 4H), 2.78 (dd, *J* = 14.8, 6.9 Hz, 4H), 2.70 (t, *J* = 6.7 Hz, 4H), 1.68 (t, *J* = 8.3 Hz, 2H); **^{13}C NMR** (126 MHz, $CDCl_3$) δ 171.5, 69.0, 63.6, 38.4, 19.7; **IR** (KBr, cm^{-1}) 3117, 2951, 1744, 1514, 1398, 1128; **HRMS** (ESI) calcd. For $C_{10}H_{18}NaO_5S_2^+$ $[M+Na]^+$ m/z 305.0488, found: 305.0485.

(Ethane-1,2-diylbis(oxy))bis(ethane-2,1-diyl) bis(3-mercaptopropanoate) (24a)

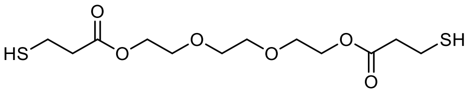

Prepared according to the general procedure **A**, 2,2'-(ethane-1,2-diylbis(oxy))bis(ethan-1-ol)) (2.5 mmol, 1.0 equiv.), 3-mercaptopropanoic acid (7.5 mmol, 3.0 equiv.), *p*-toluenesulfonic acid monohydrate (0.5 mmol, 0.2 equiv.) were used. Purification by column chromatography (Eluent: petroleum ether/ethyl acetate = 1/1) afforded the title compound as a colorless oil (587 mg, 72% yield).

^{1}H NMR (500 MHz, $CDCl_3$) δ 4.31 – 4.22 (m, 4H), 3.71 (dd, *J* = 4.0, 3.1 Hz, 4H), 3.65 (d, *J* = 1.5 Hz, 4H), 2.78 (dd, *J* = 13.9, 7.0 Hz, 4H), 2.69 (t, *J* = 6.4 Hz, 4H), 1.69 (td, *J* = 8.3, 0.8 Hz, 2H); **^{13}C NMR** (126 MHz, $CDCl_3$) δ 171.5, 70.5, 69.1, 63.7, 38.4, 19.7; **IR** (KBr, cm^{-1}) 3202, 2948, 1730, 1581, 1440, 1242, 1105; **HRMS** (ESI) calcd. For $C_{12}H_{22}NaO_6S_2^+$ $[M+Na]^+$ m/z 349.0750, found: 349.0753.

((Oxybis(ethane-2,1-diyl))bis(oxy))bis(ethane-2,1-diyl) bis(3-mercaptopropanoate) (25a)

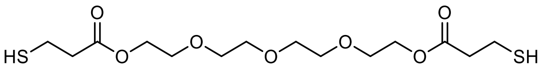

Prepared according to the general procedure **A**, 2,2'-((oxybis(ethane-2,1-diyl))bis(oxy))bis(ethan-1-ol) (2.5 mmol, 1.0 equiv.), 3-mercaptopropanoic acid (7.5 mmol, 3.0 equiv.), *p*-toluenesulfonic acid monohydrate (0.5 mmol, 0.2 equiv.) were used. Purification by column chromatography (Eluent: petroleum ether/ethyl acetate = 1/1) afforded the title compound as a colorless oil (587 mg, 88% yield).

^{1}H NMR (500 MHz, $CDCl_3$) δ 4.30 – 4.22 (m, 4H), 3.73 – 3.70 (m, 4H), 3.66 (d, *J* = 1.1 Hz, 8H), 2.78 (dd, *J* = 14.6, 7.2 Hz, 4H), 2.69 (t, *J* = 6.6 Hz, 4H), 1.70 (t, *J* = 8.3 Hz, 2H); **^{13}C NMR** (126 MHz, $CDCl_3$) δ 171.5, 70.6, 70.5, 69.0, 63.7, 38.4, 19.7; **IR** (KBr, cm^{-1}) 3170, 2956, 1730, 1630, 1397, 1129; **HRMS** (ESI) calcd. For $C_{14}H_{26}NaO_7S_2^+$ $[M+Na]^+$ m/z 393.1012, found: 393.1020.

3,6,9,12,15,18,21-Heptaoxatricosane-1,23-diyl bis(3-mercaptopropanoate) (26a)

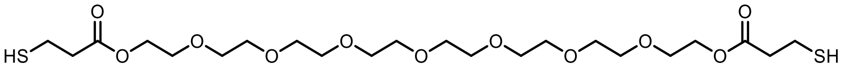

Prepared according to the general procedure **B**, 3,6,9,12,15,18,21-heptaoxatricosane-1,2,3-diol (2.5 mmol, 1.0 equiv.), 3-mercaptopropanoic acid (7.5 mmol, 3.0 equiv.), *p*-toluenesulfonic acid monohydrate (0.5 mmol, 0.2 equiv.) were used. Purification by column chromatography (Eluent: ethyl acetate) afforded the title compound as a colorless oil (1.08 g, 79% yield).

^{1}H NMR (500 MHz, $CDCl_3$) δ 4.27 (dd, *J* = 9.5, 5.0 Hz, 4H), 3.72 (dd, *J* = 9.5, 4.8 Hz, 4H), 3.65 (d, *J* = 4.7 Hz, 24H), 2.82 – 2.73 (m, 4H), 2.69 (dd, *J* = 12.0, 5.9 Hz, 4H), 1.76 – 1.65 (m, 2H); **^{13}C NMR** (126 MHz, $CDCl_3$) δ 171.5, 70.6, 70.5, 69.0, 63.7, 38.4, 19.7; **IR** (KBr, cm^{-1}) 3128, 2919, 1398, 1115; **HRMS** (ESI) calcd. For $C_{22}H_{43}O_{11}S_2^+$ $[M+H]^+$ m/z 547.2241, found: 547.2239.

(1,4-Phenylenebis(oxy))bis(ethane-2,1-diyl)bis(2-mercaptobenzoate) (52a)

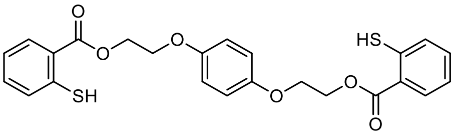

Prepared according to the general procedure **A**, 2,2'-(1,4-phenylenebis(oxy))bis(ethan-1-ol) (2.5 mmol, 1.0 equiv.), 2-mercaptobenzoic acid (7.5 mmol, 3.0 equiv.), *p*-toluenesulfonic acid monohydrate (2.5 mmol, 1.0 equiv.) were used. Purification by column chromatography (Eluent: petroleum ether/ethyl acetate = 4/1) afforded the title compound as a white solid (1.08 g, 69% yield).

^{1}H NMR (500 MHz, $CDCl_3$) δ 7.94 (d, J = 7.8 Hz, 2H), 7.22 (dd, J = 4.6, 1.1 Hz, 4H), 7.06 (ddd, J = 8.3, 4.9, 3.6 Hz, 2H), 6.81 (s, 4H), 4.59 (s, 2H), 4.58 – 4.50 (m, 4H), 4.27 – 4.10 (m, 4H); **^{13}C NMR** (126 MHz, $CDCl_3$) δ 166.6, 153.1, 138.4, 132.6, 131.9, 130.9, 125.7, 124.7, 115.9, 66.7, 63.7; **Mp**: 91.1 – 104.5 °C; **IR** (KBr, cm^{-1}) 3308, 2925, 1701, 1560, 1442, 1222, 923; **HRMS** (ESI) calcd. For $C_{24}H_{22}NaO_6S_2^+$ $[M+Na]^+$ m/z 493.0750, found: 493.0749.

6-Mercaptohexyl 2-mercaptobenzoate (33a)

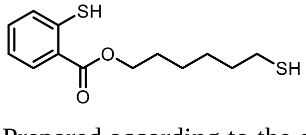

Prepared according to the general procedure **A**, 2-mercaptobenzoic acid (2.5 mmol, 1.0 equiv.), 6-mercaptohexan-1-ol (3.25 mmol, 1.3 equiv.), *p*-toluenesulfonic acid monohydrate (1.25 mmol, 0.5

equiv.) were used. Purification by column chromatography (Eluent: petroleum ether/ethyl acetate = 10/1) afforded the title compound as a colorless oil (560 mg, 83% yield).

^{1}H NMR (500 MHz, $CDCl_3$) δ 7.93 (d, *J* = 7.9 Hz, 1H), 7.23 (d, *J* = 3.8 Hz, 2H), 7.08 (dt, *J* = 8.3, 4.2 Hz, 1H), 4.60 (s, 1H), 4.25 (t, *J* = 6.6 Hz, 2H), 2.45 (q, *J* = 7.4 Hz, 2H), 1.85 – 1.63 (m, 2H), 1.62 – 1.51 (m, 2H), 1.38 (s, 4H), 1.26 (t, *J* = 7.8 Hz, 1H); **^{13}C NMR** (126 MHz, $CDCl_3$) δ 166.7, 138.2, 132.4, 131.6, 131.0, 126.2, 124.7, 65.2, 33.9, 28.6, 28.0, 25.6, 24.5; **IR** (KBr, cm^{-1}) 3306, 2821, 1735, 1560, 1440, 1349, 1251, 919; **HRMS** (ESI) calcd. For $C_{13}H_{18}NaO_2S_2^+$ $[M+Na]^+$ m/z 293.0640, found: 293.0637.

1,2-Phenylenebis(methylene) bis(3-mercaptopropanoate) (34a)

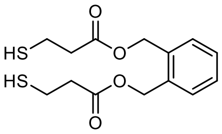

Prepared according to the general procedure **A**, 1,2-phenylenedimethanol (2.5 mmol, 1.0 equiv.), 3-mercaptopropanoic acid (7.5 mmol, 3.0 equiv.), *p*-toluenesulfonic acid monohydrate (0.125 mmol, 0.05 equiv.) were used. Purification by column chromatography (Eluent: petroleum ether/ethyl acetate = 10/1) afforded the title compound as a colorless oil (628 mg, 80% yield).

^{1}H NMR (500 MHz, $CDCl_3$) δ 7.43 – 7.38 (m, 2H), 7.37 – 7.33 (m, 2H), 5.24 (s, 4H), 2.77 (dd, *J* = 14.1, 7.4 Hz, 4H), 2.68 (t, *J* = 6.6 Hz, 4H), 1.63 (t, *J* = 8.3 Hz, 2H); **^{13}C NMR** (126 MHz, $CDCl_3$) δ 171.3, 134.3, 129.9, 128.9, 64.0, 38.4, 19.7; **IR** (KBr, cm^{-1}) 3137, 2925, 1541, 1398, 1141; **HRMS** (ESI) calcd. For $C_{14}H_{18}NaO_4S_2^+$ $[M+Na]^+$ m/z 337.0539, found: 337.0541.

1,3-Phenylenebis(methylene) bis(3-mercaptopropanoate) (36a)

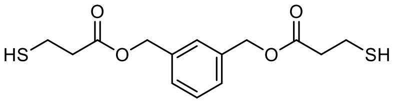

Prepared according to the general procedure **A**, 1,3-phenylenedimethanol (2.5 mmol, 1.0 equiv.), 3-mercaptopropanoic acid (7.5 mmol, 3.0 equiv.), *p*-toluenesulfonic acid monohydrate (0.125 mmol, 0.05 equiv.) were used. Purification by column chromatography (Eluent: petroleum ether/ethyl acetate = 10/1) afforded the title compound as a colorless oil (706 mg, 90% yield).

^{1}H NMR (500 MHz, $CDCl_3$) δ 7.40 – 7.35 (m, 2H), 7.32 (d, *J* = 7.7 Hz, 2H), 2.79 (dd, *J* = 14.3, 7.6 Hz, 4H), 2.71 (t, *J* = 6.6 Hz, 4H), 1.64 (t, *J* = 8.3 Hz, 2H); **^{13}C NMR** (126 MHz, $CDCl_3$) δ 171.4, 136.2, 128.9, 128.2, 128.0, 66.2, 38.5, 19.8; **IR** (KBr, cm^{-1}) 3133, 2916, 1398, 1385, 1129; **HRMS** (ESI) calcd. For $C_{14}H_{18}NaO_4S_2^+$ $[M+Na]^+$ m/z 337.0539, found: 337.0541.

1,4-Phenylenebis(methylene) bis(3-mercaptopropanoate) (37a)

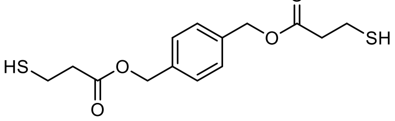

Prepared according to the general procedure **A**, 1,4-phenylenedimethanol (2.5 mmol, 1.0 equiv.), 3-mercaptopropanoic acid (7.5 mmol, 3.0 equiv.), *p*-toluenesulfonic acid monohydrate (0.125 mmol, 0.05 equiv.) were used. Purification by column chromatography (Eluent: petroleum ether/ethyl acetate = 10/1) afforded the title compound as a colorless oil (651 mg, 83% yield).

^{1}H NMR (500 MHz, $CDCl_3$) δ 7.36 (s, 4H), 5.15 (s, 4H), 2.78 (dd, *J* = 14.3, 7.6 Hz, 4H), 2.70 (t, *J*

= 6.6 Hz, 4H), 1.63 (t, *J* = 8.3 Hz, 2H); **^{13}C NMR** (126 MHz, $CDCl_3$) δ 1171.4, 135.9, 128.5, 66.1, 38.5, 19.8; **IR** (KBr, cm^{-1}) 3128, 2920, 1740, 1640, 1457, 1398, 1266, 1143, 742; **HRMS** (ESI) calcd. For $C_{14}H_{18}NaO_4S_2^+$ $[M+Na]^+$ m/z 337.0539, found: 337.0537.

1,1'-Ferrocenedicarboxylic acid bis(bis(6-mercaptohexyl) (42a)

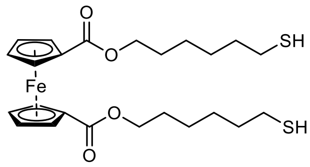

Prepared according to the general procedure **A**, 1,1'-ferrocenedicarboxylic acid (2.5 mmol, 1.0 equiv.), 6-mercaptohexan-1-ol (7.5 mmol, 3.0 equiv.), *p*-toluenesulfonic acid monohydrate (5.0 mmol, 2.0 equiv.) were used. Purification by column chromatography (Eluent: petroleum ether/ethyl acetate = 6/1) afforded the title compound as a red black oil (670 mg, 53% yield).
^{1}H NMR (500 MHz, $CDCl_3$) δ 4.82 (s, 4H), 4.40 (s, 4H), 4.22 (t, *J* = 6.7 Hz, 4H), 2.55 (q, *J* = 7.4 Hz, 4H), 1.74 (p, *J* = 6.7 Hz, 4H), 1.66 (q, *J* = 7.2 Hz, 4H), 1.55 – 1.42 (m, 8H), 1.36 (t, *J* = 7.8 Hz, 2H); **^{13}C NMR** (126 MHz, $CDCl_3$) δ 170.5, 73.1, 72.8, 71.5, 64.4, 33.9, 28.7, 28.0, 25.5, 24.6; **IR** (KBr, cm^{-1}) 3170, 3073, 2978, 1890, 1592, 1387, 1135, 909; **HRMS** (ESI) calcd. For $C_{24}H_{34}FeNaO_4S_2^+$ $[M+Na]^+$ m/z 529.1140, found: 529.1136.

Pyridine-2,6-diylbis(methylene) bis(3-mercaptopropanoate) (40a)

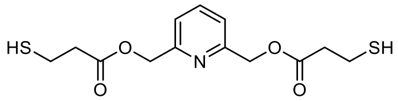

Prepared according to the general procedure **A**, pyridine-2,6-diyldimethanol (2.5 mmol, 1.0 equiv.), 3-mercaptopropanoic acid (7.5 mmol, 3.0 equiv.), *p*-toluenesulfonic acid monohydrate (2.75 mmol, 1.1 equiv.) were used. Purification by column chromatography (Eluent: petroleum ether/ethyl acetate = 1/1) afforded the title compound as a pink oil (567 mg, 72% yield).
^{1}H NMR (500 MHz, $CDCl_3$) δ 7.73 (t, *J* = 7.8 Hz, 1H), 7.31 (d, *J* = 8.0 Hz, 2H), 5.26 (s, 4H), 2.85 – 2.80 (m, 4H), 2.80 – 2.76 (m, 4H), 1.72 (t, *J* = 8.1 Hz, 2H); **^{13}C NMR** (126 MHz, $CDCl_3$) δ 171.2, 155.4, 137.6, 121.0, 66.8, 38.4, 19.7; **IR** (KBr, cm^{-1}) 3184, 2964, 1732, 1661, 1394, 1148, 750; **HRMS** (ESI) calcd. For $C_{13}H_{18}NO_4S_2^+$ $[M+H]^+$ m/z 316.0672, found: 316.0678.

(Phenylazanediyl)bis(ethane-2,1-diyl) bis(3-mercaptopropanoate) (27a)

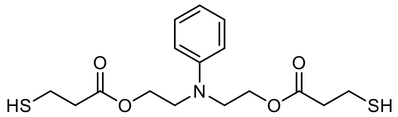

Prepared according to the general procedure **A**, pyridine-2,6-diyldimethanol (2.5 mmol, 1.0 equiv.), 3-mercaptopropanoic acid (7.5 mmol, 3.0 equiv.), *p*-toluenesulfonic acid monohydrate (2.75 mmol, 1.1 equiv.) were used. Purification by column chromatography (Eluent: petroleum ether/ethyl acetate = 3/1) afforded the title compound as a colorless oil (794 mg, 89% yield).
^{1}H NMR (500 MHz, $CDCl_3$) δ 7.24 (dd, *J* = 13.8, 6.2 Hz, 2H), 6.74 (dd, *J* = 16.4, 8.0 Hz, 3H), 4.29 (t, *J* = 6.2 Hz, 4H), 3.64 (t, *J* = 6.2 Hz, 4H), 2.74 (dd, *J* = 14.6, 7.3 Hz, 4H), 2.63 (t, *J* = 6.7 Hz, 4H), 1.62 (t, *J* = 8.4 Hz, 2H); **^{13}C NMR** (126 MHz, $CDCl_3$) δ 171.6, 147.2, 129.5, 117.2, 112.2, 61.7, 49.7, 38.4, 19.7; **IR** (KBr, cm^{-1}) 2959, 1732, 1697, 1541, 1385, 1193; **HRMS** (ESI) calcd. For $C_{16}H_{23}NNaO_4S_2^+$ $[M+Na]^+$ m/z 380.0961, found: 380.0952.

Bis(6-mercaptohexyl) 2,2'-(methylazanediyl)diacetate (28a)

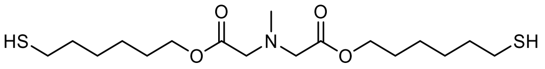

Prepared according to the general procedure **A**, 2,2'-(methylazanediyl)diacetic acid (2.5 mmol, 1.0 equiv.), 6-mercaptohexan-1-ol (7.5 mmol, 3.0 equiv.), *p*-toluenesulfonic acid monohydrate (2.75 mmol, 1.1 equiv.) were used. Purification by column chromatography (Eluent: petroleum ether/ethyl acetate = 4/1) afforded the title compound as a colorless oil (682 mg, 72% yield).

^{1}H NMR (500 MHz, $CDCl_3$) δ 4.12 (t, *J* = 6.7 Hz, 4H), 3.50 (s, 4H), 2.65 – 2.47 (m, 7H), 1.70 – 1.55 (m, 10H), 1.47 – 1.39 (m, 4H), 1.39 – 1.29 (m, 4H); **^{13}C NMR** (126 MHz, $CDCl_3$) δ 170.9, 64.5, 57.2, 42.2, 33.8, 28.5, 27.9, 25.4, 24.5; **IR** (KBr, cm^{-1}) 3291, 2935, 1730, 1443, 1189, 742; **HRMS** (ESI) calcd. For $C_{17}H_{33}NNaO_4S_2^+$ $[M+Na]^+$ m/z 402.1743, found: 402.1742.

Piperazine-1,4-diylbis(ethane-2,1-diyl) bis(3-mercaptopropanoate) (32a)

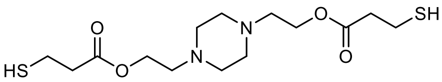

Prepared according to the general procedure **A**, 2,2'-(methylazanediyl)diacetic acid (2.5 mmol, 1.0 equiv.), 3-mercaptopropanoic acid (7.5 mmol, 3.0 equiv.), *p*-toluenesulfonic acid monohydrate (5.5 mmol, 2.2 equiv.) were used. Purification by column chromatography (Eluent: DCM/CH_3OH = 30/1) afforded the title compound as a yellowish liquid (403 mg, 46% yield).

^{1}H NMR (500 MHz, $CDCl_3$) δ 4.24 (t, *J* = 5.8 Hz, 4H), 2.77 (dd, *J* = 14.2, 7.1 Hz, 4H), 2.68 (d, *J* = 6.6 Hz, 3H), 2.65 (d, *J* = 4.4 Hz, 2H), 2.63 (d, *J* = 5.8 Hz, 3H), 2.54 (s, 8H), 1.86 (t, *J* = 8.3 Hz, 2H); **^{13}C NMR** (126 MHz, $CDCl_3$) δ 171.5, 61.8, 56.6, 53.2, 38.6, 19.9; **IR** (KBr, cm^{-1}) 3303, 2942, 1732, 1457, 1160; **HRMS** (ESI) calcd. For $C_{14}H_{27}N_2O_4S_2^+$ $[M+H]^+$ m/z 351.1407, found: 351.1403.

Furan-2,5-diylbis(methylene) bis(3-mercaptopropanoate) (41a)

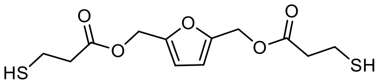

Prepared according to the general procedure **B**, furan-2,5-diyldimethanol (5 mmol, 1.0 equiv.), 3-mercaptopropanoic acid (10 mmol, 2.0 equiv.), *N*,*N'*-dicyclohexylcarbodiimide (10 mmol, 2.0 equiv.), 4-dimethylaminopyridine (0.2 mmol, 4 mol %) and dichloromethane (40 mL) were used. Purification by column chromatography (Eluent: petroleum ether/ethyl acetate = 6/1) afforded the title compound as a colorless liquid (714 mg, 47% yield).

^{1}H NMR (500 MHz, $CDCl_3$) δ 6.36 (d, *J* = 3.1 Hz, 1H), 6.25 (d, *J* = 3.0 Hz, 1H), 5.05 (s, 2H), 4.58 (s, 2H), 3.14 (t, *J* = 6.9 Hz, 2H), 2.87 (t, *J* = 6.6 Hz, 2H), 2.82 – 2.74 (m, 2H), 2.66 (t, *J* = 6.9 Hz, 2H), 1.61 (t, *J* = 8.2 Hz, 2H); **^{13}C NMR** (126 MHz, $CDCl_3$) δ 197.2, 171.3, 155.1, 149.0, 111.7, 111.7, 108.6, 58.5, 57.3, 47.5, 34.2, 23.9, 19.9; **IR** (KBr, cm^{-1}) 3482, 3162, 1731, 1635, 1401, 1384, 1113; **HRMS** (ESI) calcd. For $C_{12}H_{16}NaO_5S_2^+$ $[M+Na]^+$ m/z 327.0331, found: 327.0327.

(Naphthalene-2,7-diylbis(oxy))bis(propane-3,1-diyl) bis(3-mercaptopropanoate) (39a)

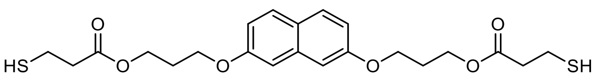

Prepared according to the general procedure **A**, 3,3'-(naphthalene-2,7-diylbis(oxy))bis(propan-1-ol) (2.5 mmol, 1.0 equiv.), 3-mercaptopropanoic acid (7.5 mmol, 3.0 equiv.), *p*-toluenesulfonic acid monohydrate (0.25 mmol, 0.1 equiv.) were used. Purification by column chromatography (Eluent:

petroleum ether/ethyl acetate = 4/1) afforded the title compound as a colorless oil (249 mg, 22% yield).
^{1}H NMR (500 MHz, $CDCl_3$) δ 7.64 (d, *J* = 8.9 Hz, 2H), 7.03 (d, *J* = 1.4 Hz, 2H), 6.98 (dd, *J* = 8.9, 2.0 Hz, 2H), 4.34 (t, *J* = 6.3 Hz, 4H), 4.13 (t, *J* = 6.1 Hz, 4H), 2.75 (dd, *J* = 14.5, 7.3 Hz, 4H), 2.64 (t, *J* = 6.7 Hz, 4H), 2.17 (p, *J* = 6.1 Hz, 4H), 1.63 (t, *J* = 8.3 Hz, 2H); **^{13}C NMR** (126 MHz, $CDCl_3$) δ 171.6, 157.3, 135.8, 129.2, 124.4, 116.3, 106.1, 64.3, 61.7, 38.5, 28.6, 19.8; **IR** (KBr, cm^{-1}) 3063, 2945, 1740, 1512, 1254, 741; **HRMS** (ESI) calcd. For $C_{22}H_{28}NaO_6S_2^+$ $[M+Na]^+$ m/z 475.1220, found: 475.1212.

Bis(6-mercaptohexyl) 2,2'-(1,2-phenylenebis(oxy))diacetate (35a)

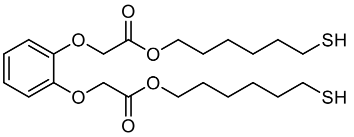

Prepared according to the general procedure **A**, 2,2'-(1,2-phenylenebis(oxy))diacetic acid (2.5 mmol, 1.0 equiv.), 6-mercaptohexan-1-ol (7.5 mmol, 3.0 equiv.), *p*-toluenesulfonic acid monohydrate (0.125 mmol, 0.05 equiv.) were used. Purification by column chromatography (Eluent: petroleum ether/ethyl acetate = 5/1) afforded the title compound as a colorless oil (961 mg, 84% yield).
^{1}H NMR (500 MHz, $CDCl_3$) δ 6.87 (d, J = 4.0 Hz, 2H), 6.84 – 6.78 (m, 2H), 4.65 (s, 4H), 4.12 (t, *J* = 6.5 Hz, 4H), 2.43 (q, *J* = 7.1 Hz, 4H), 1.64 – 1.55 (m, 4H), 1.51 (q, *J* = 7.3 Hz, 4H), 1.39 – 1.28 (m, 4H), 1.25 (q, *J* = 7.5 Hz, 6H); **^{13}C NMR** (126 MHz, $CDCl_3$) δ 169.1, 148.0, 122.5, 115.0, 66.6, 65.2, 33.8, 28.4, 27.9, 25.3, 24.5; **IR** (KBr, cm^{-1}) 3137, 2925, 1541, 1398, 1141; **HRMS** (ESI) calcd. For $C_{14}H_{18}NaO_4S_2^+$ $[M+Na]^+$ m/z 337.0539, found: 337.0541.

(1,4-Phenylenebis(oxy))bis(ethane-2,1-diyl) bis(3-mercaptopropanoate) (38)

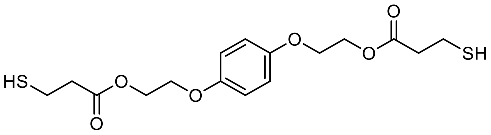

Prepared according to the general procedure A, 2,2'-(1,4-phenylenebis(oxy))bis(ethan-1-ol) (2.5 mmol, 1.0 equiv.), 3-mercaptopropanoic acid (7.5 mmol, 3.0 equiv.), *p*-toluenesulfonic acid monohydrate (0.25 mmol, 0.10 equiv.) were used. Purification by column chromatography (Eluent: petroleum ether/ethyl acetate = 1/1) afforded the title compound as a colorless oil (654 mg, 70% yield).
^{1}H NMR (500 MHz, $CDCl_3$) δ 6.85 (s, 4H), 4.43 (m, 4H), 4.13 (m, 4H), 2.77 (dd, *J* = 13.9, 7.2 Hz, 4H), 2.69 (t, *J* = 6.5 Hz, 4H), 1.67 (t, *J* = 8.3 Hz, 2H); **^{13}C NMR** (126 MHz, $CDCl_3$) δ 171.5, 153.0, 115.8, 66.6, 63.1, 38.4, 19.7; **IR** (KBr, cm^{-1}) 3125, 2920, 1747, 1397, 1164, 1115; **HRMS** (ESI) calcd. For $C_{14}H_{18}NaO_4S_2^+$ $[M+Na]^+$ m/z 337.0539, found: 337.0537.

(*R*)-(+)-3,3'-(([1,1'-Binaphthalene]-2,2'-diylbis(oxy))bis(propane-3,1-diyl)) diethanethioate (43a)

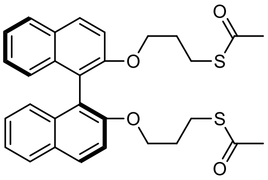

Prepared according to the general procedure **C**, (*R*)-(+)-1,1'-bi(2-naphthol) (1.43 g, 5 mmol, 1.0 equiv.), 3-chloropropan-1-ol (1.89 g, 20 mmol, 4.0 equiv.), anhydrous DMF (60 mL), potassium carbonate (2.76g, 20 mmol, 4.0 equiv.), triphenylphosphine (1.96g, 7.5 mmol, 1.5 equiv.), diisopropyl azodiformate (1.52 g, 7.5 mmol, 1.5 equiv.), thiolacetic acid (570 mg, 7.5 mmol, 1.5 equiv.) and dry THF (50 mL) were used. Purification by column chromatography (Eluent: petroleum ether/ethyl acetate = 12/1) afforded the title compound as a colorless liquid (1.29 g, 79% yield).
^{1}H NMR (500 MHz, $CDCl_3$) δ 7.93 (d, *J* = 9.0 Hz, 2H), 7.85 (d, *J* = 8.1 Hz, 2H), 7.39 (d, *J* = 9.0 Hz, 2H), 7.31 (t, *J* = 7.3 Hz, 2H), 7.22 (t, *J* = 7.5 Hz, 2H), 7.15 (d, *J* = 8.4 Hz, 2H), 4.03 (dt, *J* = 11.0, 5.8 Hz, 2H), 3.99 – 3.89 (m, 2H), 2.42 (dt, *J* = 13.7, 6.9 Hz, 2H), 2.33 (dt, *J* = 13.9, 7.1 Hz, 2H), 2.21 (s, 6H), 1.67 (tt, *J* = 14.5, 7.2 Hz, 4H); **^{13}C NMR** (126 MHz, $CDCl_3$) δ 196.0, 154.0, 134.1, 129.4, 129.3, 127.9, 126.3, 125.4, 123.7, 120.6, 115.7, 68.0, 30.5, 29.4, 25.5.

(*R*)-(+)-3,3'-([1,1'-Binaphthalene]-2,2'-diylbis(oxy))bis(propane-1-thiol) (43b)

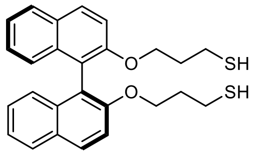

Prepared according to the general procedure **C**, (*R*)-(+)-(([1,1'-binaphthalene]-2,2'-diylbis(oxy))bis(propane-3,1-diyl)) diethanethioate (778 mg, 1.5 mmol, 1 equiv.), THF (1.0 mL)/ CH_3OH (9.0 mL) and potassium carbonate (248 mg, 1.8 mmol, 1.2 equiv.) were used. Purification by column chromatography (Eluent: petroleum ether/ethyl acetate = 12/1) afforded the title compound as a colorless oil (398 mg, 61% yield).
^{1}H NMR (500 MHz, $CDCl_3$) δ 6.85 (s, 4H), 4.43 (m, 4H), 4.13 (m, 4H), 2.77 (dd, *J* = 13.9, 7.2 Hz, 4H), 2.69 (t, *J* = 6.5 Hz, 4H), 1.67 (t, *J* = 8.3 Hz, 2H); **^{13}C NMR** (126 MHz, $CDCl_3$) δ 154.1, 134.1, 129.4, 129.4, 128.0, 126.4, 125.4, 123.8, 120.7, 115.8, 67.3, 33.4, 20.7; **IR** (KBr, cm^{-1}) 3307, 2996, 1735, 1591, 1436, 916; **HRMS** (ESI) calcd. For $C_{26}H_{26}NaO_2S_2$+ $[M+Na]^+$ m/z 457.1266, found: 457.1254.

(*R*)-(+)-6,6'- (([1,1'-Binaphthalene]-2,2'-diylbis(oxy))bis(hexane-6,1-diyl)) diethanethioate (44a)

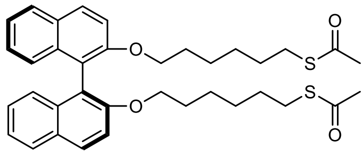

Prepared according to the general procedure **C**, (*R*)-(+)-1,1'-bi(2-naphthol) (1.43 g, 5 mmol, 1.0 equiv.), 6-chlorohexan-1-ol (2.73 g, 20 mmol, 4.0 equiv.), anhydrous DMF (60 mL), potassium carbonate (2.76g, 20 mmol, 4.0 equiv.), triphenylphosphine (1.96g, 7.5 mmol, 1.5 equiv.), diisopropyl azodiformate (1.52 g, 7.5 mmol, 1.5 equiv.), thiolacetic acid (570 mg, 7.5 mmol, 1.5 equiv.) and dry THF (50 mL) were used. Purification by column chromatography (Eluent: petroleum ether/ethyl acetate = 14/1) afforded the title compound as a colorless liquid (2.41 g, 80% yield).
^{1}H NMR (500 MHz, $CDCl_3$) δ 7.92 (d, *J* = 9.0 Hz, 2H), 7.84 (d, *J* = 8.2 Hz, 2H), 7.39 (d, *J* = 9.0 Hz, 2H), 7.29 (ddd, *J* = 8.0, 6.6, 1.2 Hz, 2H), 7.22 – 7.17 (m, 2H), 7.14 (d, *J* = 8.4 Hz, 2H), 3.96 (dt, *J* = 9.3, 6.0 Hz, 2H), 3.87 (dt, *J* = 9.3, 6.3 Hz, 2H), 2.65 (t, *J* = 7.4 Hz, 4H), 2.31 (s, 6H), 1.37 (dq, *J* = 14.0, 6.8 Hz, 4H), 1.24 (dtd, *J* = 14.4, 7.2, 3.2 Hz, 4H), 1.06 – 0.92 (m, 4H), 0.84 (dtd, *J* = 15.7, 8.2, 2.3 Hz, 4H); **^{13}C NMR** (126 MHz, $CDCl_3$) δ 196.0, 154.5, 134.2, 129.3, 129.1, 127.8, 126.1,

125.5, 123.5, 120.8, 116.0, 30.7, 29.2, 29.2, 29.0, 28.2, 25.1.

(*R*)-(+)-6,6'-([1,1'-Binaphthalene]-2,2'-diylbis(oxy))bis(hexane-1-thiol) (44b)

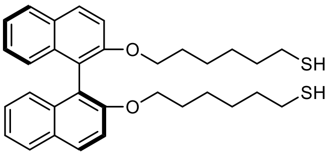

Prepared according to the general procedure **C**, (*R*)-(+)-(([1,1'-binaphthalene]-2,2'-diylbis(oxy))bis(hexane-6,1-diyl)) diethanethioate (904 mg, 1.5 mmol, 1 equiv.), THF (1.0 mL)/ CH_3OH (9.0 mL) and potassium carbonate (248 mg, 1.8 mmol, 1.2 equiv.) were used. Purification by column chromatography (Eluent: petroleum ether/ethyl acetate = 12/1) afforded the title compound as a colorless oil (567 mg, 73% yield).

^{1}H NMR (500 MHz, $CDCl_3$) δ 7.91 (d, *J* = 9.0 Hz, 2H), 7.84 (d, *J* = 8.1 Hz, 2H), 7.39 (d, *J* = 9.0 Hz, 2H), 7.29 (t, *J* = 7.3 Hz, 2H), 7.17 (dt, *J* = 15.3, 8.5 Hz, 4H), 3.96 (dt, *J* = 9.3, 6.0 Hz, 2H), 3.86 (dt, *J* = 9.2, 6.3 Hz, 2H), 2.26 (q, *J* = 7.4 Hz, 4H), 1.36 (tt, *J* = 14.3, 7.3 Hz, 4H), 1.27 (td, *J* = 7.4, 2.9 Hz, 4H), 1.21 ((t, *J* = 7.7 Hz, 2H)1.01 (ddt, *J* = 11.7, 8.2, 4.2 Hz, 4H), 0.83 (dt, *J* = 13.7, 7.1 Hz, 4H); **^{13}C NMR** (126 MHz, $CDCl_3$) δ 154.5, 134.3, 129.4, 129.1, 127.9, 126.1, 125.5, 123.5, 120.9, 116.0, 69.7, 33.8, 29.3, 27.8, 25.1, 24.4; **IR** (KBr, cm^{-1}) 3314, 2921, 1595, 1446, 919; **HRMS** (ESI) calcd. For $C_{32}H_{38}NaO_2S_2^+$ $[M+Na]^+$ m/z 541.2205, found: 541.2215.

(*R*)-(+)-([1,1'-Binaphthalene]-2,2'-diylbis(oxy))bis(propane-3,1-diyl)bis(3-mercaptopropanoate) (45a)

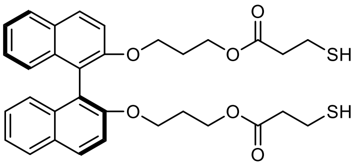

Prepared according to the general procedure **A** and **C**, (*R*)-(+)-3,3'-([1,1'-binaphthalene]-2,2'-diylbis(oxy))bis(propan-1-ol) (2.5 mmol, 1.0 equiv.), 3-mercaptopropanoic acid (7.5 mmol, 3.0 equiv.), *p*-toluenesulfonic acid monohydrate (0.125 mmol, 0.05 equiv.) were used. Purification by column chromatography (Eluent: petroleum ether/ethyl acetate = 6/1) afforded the title compound as a colorless oil (1.12 g, 78% yield).

^{1}H NMR (500 MHz, $CDCl_3$) δ 7.94 (d, *J* = 9.0 Hz, 2H), 7.86 (d, *J* = 8.1 Hz, 2H), 7.41 (d, *J* = 9.0 Hz, 2H), 7.31 (t, *J* = 7.4 Hz, 2H), 7.20 (t, *J* = 7.5 Hz, 2H), 7.13 (d, *J* = 8.5 Hz, 2H), 4.10 – 4.03 (m, 2H), 4.01 – 3.94 (m, 2H), 3.80 – 3.72 (m, 2H), 3.66 – 3.59 (m, 2H), 2.66 (dd, *J* = 14.9, 7.1 Hz, 4H), 2.48 (t, *J* = 6.8 Hz, 4H), 1.79 – 1.67 (m, 4H), 1.56 (t, *J* = 8.4 Hz, 2H); **^{13}C NMR** (126 MHz, $CDCl_3$) δ 171.3, 154.0, 134.0, 129.4, 128.0, 126.3, 125.4, 123.8, 120.6, 115.7, 66.1, 61.3, 38.3, 28.7, 19.7; **IR** (KBr, cm^{-1}) 3204, 2962, 1727, 1655, 1395, 1148, 1085, 806; **HRMS** (ESI) calcd. For $C_{32}H_{34}NaO_6S_2^+$ $[M+Na]^+$ m/z 601.1689, found: 601.1683.

(*R*)-(+)-(([1,1'-Binaphthalene]-2,2'-diylbis(oxy))bis(octane-8,1-diyl)) diethanethioate (46a)

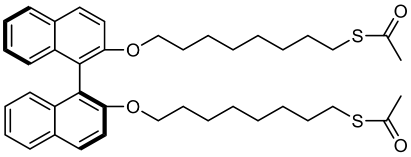

Prepared according to the general procedure **C**, (*R*)-(+)-1,1'-Bi(2-naphthol) (1.43 g, 5 mmol, 1.0 equiv.), 6-chlorohexan-1-ol (2.73 g, 20 mmol, 4.0 equiv.), anhydrous DMF (60 mL), potassium carbonate (2.76 g, 20 mmol, 4.0 equiv.), triphenylphosphine (1.96 g, 7.5 mmol, 1.5 equiv.), diisopropyl azodiformate (1.52 g, 7.5 mmol, 1.5 equiv.), thiolacetic acid (570 mg, 7.5 mmol, 1.5 equiv.) and dry THF (50 mL) were used. Purification by column chromatography (Eluent: petroleum ether/ethyl acetate = 12/1) afforded the title compound as a colorless liquid (2.13 g, 65% yield).

^{1}H NMR (500 MHz, $CDCl_3$) δ 7.91 (d, *J* = 9.0 Hz, 2H), 7.83 (d, *J* = 8.2 Hz, 2H), 7.39 (d, *J* = 9.0 Hz, 2H), 7.32 – 7.25 (m, 2H), 7.21 – 7.08 (m, 4H), 3.95 (dt, *J* = 9.0, 6.2 Hz, 2H), 3.91 – 3.83 (m, 2H), 2.82 (t, *J* = 7.4 Hz, 4H), 2.31 (s, 6H), 1.47 (p, *J* = 7.5 Hz, 4H), 1.36 (dq, *J* = 13.4, 6.7 Hz, 4H), 1.15 (p, *J* = 7.2 Hz, 4H), 1.03 – 0.91 (m, 8H), 0.86 (q, *J* = 6.5, 5.8 Hz, 4H); **^{13}C NMR** (126 MHz, $CDCl_3$) δ 196.0, 154.6, 134.3, 129.3, 129.1, 127.8, 126.1, 125.5, 123.5, 120.8, 115.9, 69.8, 30.7, 29.5, 29.4, 29.2, 28.9, 28.9, 28.7, 25.6.

(*R*)-(+)-8,8'-([1,1'-Binaphthalene]-2,2'-diylbis(oxy))bis(octane-1-thiol) (47b)

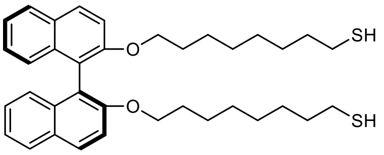

Prepared according to the general procedure **C**, (*R*)-(+)-(([1,1'-binaphthalene]-2,2'-diylbis(oxy))bis(octane-8,1-diyl)) diethanethioate (903 mg, 1.5 mmol, 1 equiv.), THF (1.0 mL)/ CH_3OH (9.0 mL) and potassium carbonate (248 mg, 1.8 mmol, 1.2 equiv.) were used. Purification by column chromatography (Eluent: petroleum ether/ethyl acetate = 12/1) afforded the title compound as a colorless oil (780 mg, 79% yield).

^{1}H NMR (500 MHz, $CDCl_3$) δ 7.91 (d, *J* = 9.0 Hz, 2H), 7.84 (d, *J* = 8.2 Hz, 2H), 7.39 (d, *J* = 9.0 Hz, 2H), 7.29 (t, *J* = 7.3 Hz, 2H), 7.18 (t, *J* = 7.5 Hz, 2H), 7.14 (d, *J* = 8.4 Hz, 2H), 3.95 (dt, *J* = 9.0, 6.2 Hz, 2H), 3.91 – 3.78 (m, 2H), 2.47 (q, *J* = 7.4 Hz, 4H), 1.59 – 1.47 (m, 4H), 1.37 (dq, *J* = 13.3, 6.7 Hz, 4H), 1.31 (t, *J* = 7.7 Hz, 2H), 1.18 (p, *J* = 7.2 Hz, 4H), 1.05 – 0.92 (m, 8H), 0.88 (q, J = 6.5, 6.0 Hz, 4H); **^{13}C NMR** (126 MHz, $CDCl_3$) δ 154.6, 134.3, 129.3, 129.1, 127.8, 126.0, 125.5, 123.4, 120.8, 115.9, 69.8, 34.0, 29.4, 29.0, 28.8, 28.2, 25.6, 24.7; **IR** (KBr, cm^{-1}) 3304, 2925, 1735, 1582, 1439, 1235, 914; **HRMS** (ESI) calcd. For $C_{36}H_{46}NaO_2S_2^+$ $[M+Na]^+$ m/z 597.2831, found: 597.2839.

(*R*)-(+)([1,1'-binaphthalene]-2,2'-diylbis(oxy))bis(pentane-5,1-diyl)bis(3-mercaptopropanoate) (47a)

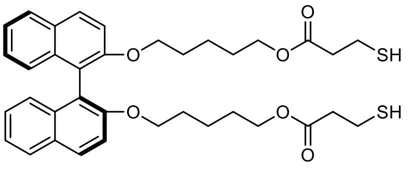

Prepared according to the general procedure **A** and **C**, (*R*)-(+)-5,5'-([1,1'-binaphthalene]-2,2'-diylbis(oxy))bis(pentan-1-ol) (2.5 mmol, 1.0 equiv.), 3-mercaptopropanoic acid (7.5 mmol, 3.0 equiv.), *p*-toluenesulfonic acid monohydrate (0.125 mmol, 0.05 equiv.) were used. Purification by column chromatography (Eluent: petroleum ether/ethyl acetate = 6/1) afforded the title compound as a colorless oil (1.25 g, 81% yield).

^{1}H NMR (500 MHz, $CDCl_3$) δ 7.93 (d, *J* = 9.0 Hz, 2H), 7.85 (d, *J* = 8.2 Hz, 2H), 7.39 (d, *J* = 9.0 Hz, 2H), 7.30 (ddd, *J* = 8.1, 6.5, 1.3 Hz, 2H), 7.24 – 7.17 (m, 2H), 7.15 (d, *J* = 8.4 Hz, 2H), 3.99 (dt,

J = 9.5, 5.9 Hz, 2H), 3.88 (ddd, J = 9.3, 6.9, 5.4 Hz, 2H), 3.84 – 3.65 (m, 4H), 2.73 (dt, J = 8.4, 6.9 Hz, 4H), 2.57 (t, J = 6.9 Hz, 4H), 1.60 (t, J = 8.3 Hz, 2H), 1.54 – 1.32 (m, 4H), 1.23 (ddd, J = 16.2, 8.2, 5.0 Hz, 4H), 0.89 (ttd, J = 10.6, 7.5, 7.1, 3.3 Hz, 4H); **^{13}C NMR** (126 MHz, $CDCl_3$) δ 171.5, 154.4, 134.2, 129.4, 129.2, 127.8, 126.1, 125.5, 123.65, 120.8, 115.9, 69.5, 64.6, 38.5, 28.9, 27.9, 22.1, 19.8.

(*R*)-(+)-([1,1'-binaphthalene]-2,2'-diylbis(oxy))bis(hexane-6,1-diyl)bis(3-mercaptopropanoate) (48a)

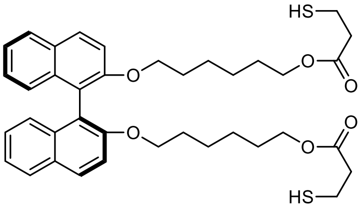

Prepared according to the general procedure **A** and **C**, (*R*)-(+)-6,6'-([1,1'-binaphthalene]-2,2'-diylbis(oxy))bis(hexan-1-ol) (2.5 mmol, 1.0 equiv.), 3-mercaptopropanoic acid (7.5 mmol, 3.0 equiv.), *p*-toluenesulfonic acid monohydrate (0.125 mmol, 0.05 equiv.) were used. Purification by column chromatography (Eluent: petroleum ether/ethyl acetate = 6/1) afforded the title compound as a colorless oil (1.32 g, 80% yield).

^{1}H NMR (500 MHz, $CDCl_3$) δ 7.91 (d, J = 9.0 Hz, 2H), 7.83 (d, J = 8.2 Hz, 2H), 7.39 (d, J = 9.0 Hz, 2H), 7.28 (t, J = 7.3 Hz, 2H), 7.20 – 7.13 (m, 4H), 3.99 – 3.93 (m, 8H), 3.92 – 3.83 (m, 4H), 2.73 (dd, J = 15.0, 6.9 Hz, 4H), 2.59 (t, J = 6.8 Hz, 2H), 1.61 (t, J = 8.4 Hz, 4H), 1.43 – 1.33 (m, 4H), 1.32 – 1.24 (m, 2H), 1.02 – 0.93 (m, 4H), 0.89 – 0.79 (m, 4H); **^{13}C NMR** (126 MHz, $CDCl_3$) δ 171.6, 154.5, 134.2, 129.3, 129.2, 127.9, 126.1, 125.5, 123.5, 120.8, 116.0, 69.6, 64.7, 38.5, 29.3, 28.4, 25.3, 25.3, 19; **IR** (KBr, cm^{-1}) 3307, 2940, 1726, 1581, 1488, 1235, 807; **HRMS** (ESI) calcd. For $C_{38}H_{46}NaO_6S_2^+$ $[M+Na]^+$ m/z 685.2628, found: 685.2626.

(1*S*, 2*S*)-*trans*-Cyclohexane-1,2-diyl bis(3-mercaptopropanoate) (49a)

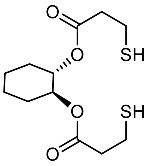

Prepared according to the general procedure **A**, (1*S*, 2*S*)-*trans*-1,2-cyclohexanediol (5.0 mmol, 1.0 equiv.), 3-mercaptopropanoic acid (15 mmol, 3.0 equiv.), *p*-toluenesulfonic acid monohydrate (1.0 mmol, 0.2 equiv.) were used. Purification by column chromatography (Eluent: petroleum ether/ethyl acetate = 6/1) afforded the title compound as a colorless oil (1.29 g, 88% yield).

^{1}H NMR (500 MHz, $CDCl_3$) δ 4.93 – 4.63 (m, 2H), 2.73 – 2.65 (m, 4H), 2.57 (dd, J = 12.4, 6.3 Hz, 4H), 2.00 (d, J = 10.1 Hz, 2H), 1.68 (s, 2H), 1.60 – 1.53 (m, 2H), 1.43 – 1.23 (m, 4H); **^{13}C NMR** (126 MHz, $CDCl_3$) δ 170.9, 73.9, 38.6, 30.2, 23.4, 19.8; **IR** (KBr, cm^{-1}) 2921, 1747, 1338, 1144; **HRMS** (ESI) calcd. For $C_{12}H_{20}NaO_4S_2^+$ $[M+Na]^+$ m/z 315.0695, found: 315.0697.

6-Mercaptohexyl (*R*)-(2-mercaptopropanoyl)glycinate (50a)

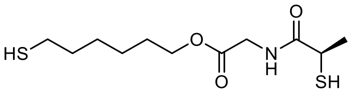

According to the general procedure **B**, an oven-dried screw-capped Schlenk tube (50 mL) equipped with a Teflon-coated magnetic stir bar were added the corresponding (*R*)-(2-mercaptopropanoyl)glycine (815 mg, 5 mmol, 1.0 equiv.), 6-mercaptohexan-1-ol (670 mg, 5 mmol, 1.0 equiv.) *N*,*N′*-dicyclohexylcarbodiimide (1.03 g, 5 mmol, 1.0 equiv.), 4-dimethylaminopyridine (32 mg, 0.2 mmol, 4 mol %), and dry dichloromethane (35 mL) under a N_2 atmosphere. The mixture was allowed to stir until all the acid was consumed (as indicated by TLC). The mixture was filtered through a silica gel plug, eluting with DCM, and the resulting filtrate concentrated *in vacuo*. Purification by column chromatography (Eluent: petroleum ether/ethyl acetate = 4/1) afforded the title compound as a colorless oil (1.29 g, 88% yield).

^{1}H NMR (500 MHz, $CDCl_3$) δ 7.20 (s, 1H), 4.15 (t, *J* = 6.6 Hz, 2H), 4.04 (dd, *J* = 5.1, 2.6 Hz, 2H), 3.53 (p, *J* = 7.3 Hz, 1H), 2.53 (q, *J* = 7.4 Hz, 2H), 2.19 (d, *J* = 8.5 Hz, 1H), 1.64 (dp, *J* = 21.6, 7.0 Hz, 4H), 1.56 (d, *J* = 7.1 Hz, 3H), 1.41 (ddt, *J* = 29.5, 15.5, 7.5 Hz, 6H); **^{13}C NMR** (126 MHz, $CDCl_3$) δ 173.4, 169.8, 65.4, 41.6, 37.5, 33.7, 28.3, 27.8, 25.3, 24.4, 22.0; **IR** (KBr, cm^{-1}) 3292, 2878, 1745, 1507, 1229; **HRMS** (ESI) calcd. For $C_{13}H_{23}NNaO_3S_2^+$ $[M+Na]^+$ m/z 328.1012, found: 328.1011.

(*R*)-((2,2',3,3'-Tetrahydro-1,1'-spirobi[indene]-7,7'-diyl)bis(oxy))bis(hexane-6,1-diyl) bis(3-mercaptopropanoate) (51a)

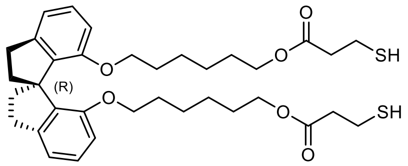

Prepared according to the general procedure **A** and **C**, (*R*)-6,6'-((2,2',3,3'-tetrahydro-1,1'-spirobi[indene]-7,7'-diyl)bis(oxy))bis(hexan-1-ol) (2.5 mmol, 1.0 equiv.), 3-mercaptopropanoic acid (7.5 mmol, 3.0 equiv.), *p*-toluenesulfonic acid monohydrate (0.125 mmol, 0.05 equiv.) were used. Purification by column chromatography (Eluent: petroleum ether/ethyl acetate = 5/1) afforded the title compound as a colorless oil (1.21 g, 77% yield).

^{1}H NMR (500 MHz, $CDCl_3$) δ 7.06 (t, *J* = 7.7 Hz, 2H), 6.79 (d, *J* = 7.4 Hz, 2H), 6.53 (d, *J* = 8.0 Hz, 2H), 4.05 (t, *J* = 6.7 Hz, 4H), 3.76 (q, *J* = 6.6, 6.0 Hz, 2H), 3.58 (q, *J* = 8.0, 6.9 Hz, 2H), 2.99 (t, *J* = 8.4 Hz, 4H), 2.77 (q, *J* = 7.2 Hz, 4H), 2.65 (t, *J* = 6.8 Hz, 4H), 2.40 – 2.23 (m, 2H), 2.15 (ddd, *J* = 11.6, 7.2, 3.0 Hz, 2H), 1.64 (t, *J* = 8.3 Hz, 2H), 1.51 (dt, *J* = 13.8, 6.7 Hz, 4H), 1.28 (ddq, *J* = 27.9, 14.3, 7.5 Hz, 4H), 1.13 (p, *J* = 7.7 Hz, 4H), 0.92 (dt, *J* = 15.0, 7.2 Hz, 4H); **^{13}C NMR** (126 MHz, $CDCl_3$) δ171.7, 155.8, 145.0, 137.2, 127.4, 116.5, 108.7, 66.8, 64.9, 59.2, 38.6, 38.2, 31.6, 29.2, 28.5, 25.6, 25.3, 19.8; **IR** (KBr, cm^{-1}) 3443, 3155, 1640, 1514, 1398, 1384, 1105, 648; **HRMS** (ESI) calcd. For $C_{35}H_{48}NaO_6S_2^+$ $[M+Na]^+$ m/z 651.2785, found: 651.2782.

5. General Procedure of Thiol-Yne Click Chemistry of Acetylene-Enabled Macrocyclization

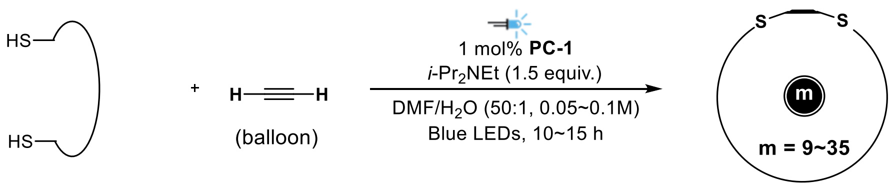

Method 1:

An oven-dried 25 mL Schlenk tube (sythware brand, 25mL, part #F580050) equipped with a Teflon-coated magnetic stir bar, was charged with [Ir[d(*t*Bu)(CF$_3$)ppy]$_2$(dtbbpy)[Cl] (1.0 mol %), linear dithiol precursor (1.0 equiv.), DMF (0.05 M), *N,N*-diisopropylethylamine (1.5 equiv.) and H_2O (DMF/H_2O = 50/1) under N_2. The tube was degassed through three freeze-pump-thaw cycles under acetylene and then an acetylene gas balloon was attached through a three-way valve. After that, the tube was placed in the Parallel Light Reactor, which cooled with the recirculated cooling water. The reaction mixture was detected by TLC (*staining with Iodine*), quenched via exposure to air, diluted with brine (4 mL) and then extracted by EtOAc (2 × 5 mL). The combined organic layer was dried over Na_2SO_4, filtered and evaporated. The residue was purified by flash column chromatography on silica gel to obtain the product.

Method 2:
To a dried Schlenk tube containing a stirrer bar was charged with the [Ir(d(*t*Bu)(CF$_3$)ppy)$_2$(dtbbpy)][Cl] (2.5 mg, 0.005 mmol, 1 mol %), linear dithiol precursor (0.25 ~ 1 mmol). Then, the tube filled with nitrogen was successively added *N,N*-diisopropylethylamine (0.38 mmol, 1.5 equiv.), water and DMF. The reaction mixture was then cooled to -78 ºC and connected to a Schlenk line degas via vacuum evacuation, backfilled with acetylene gas, and warmed to room temperature. The tube was then sealed and placed in the Parallel Light Reactor, which cooled with the recirculated cooling water. The corresponding reaction mixture was detected according to both TLC and GC-MS analysis. Finally, the reaction mixture was diluted with brine (4 mL) and extracted by EtOAc (2 × 5 mL). The combined organic layer was dried over Na_2SO_4, filtered and evaporated. The residue was purified by flash column chromatography on silica gel gave title compound.

Method 3:
The reaction mixture was stirred under two 420 ~ 435 nm Kessil lamps at room temperature for 10 ~ 14 hours (flask about 10 cm away from lights, fan for cooling) until the reaction completed and then quenched with brine (4 mL) and H_2O (5 mL). The organic phases were diluted with EtOAc (3 × 5 mL), dried over anhydrous Na_2SO_4 and concentrated to obtain the corresponding residue, which was then purified through flash chromatography on silica gel to afford the desired products.

Characterization Data for the Products

1,10-Dioxa-4,7-dithiacyclotetradecane-2,9-dione (4)

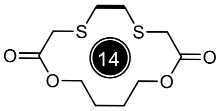

Prepared following the general procedure outlined above using [Ir[d(*t*Bu)(CF$_3$)ppy]$_2$(dtbbpy)[Cl] (5.4 mg, 5.0 μmol, 0.01 equiv.), butane-1,4-diyl bis(2-mercaptoacetate) (119 mg, 0.5 mmol, 1 equiv.), DMF (10 mL), *N,N*-diisopropylethylamine (97 mg, 0.75 mmol, 1.5 equiv.) and deionized water (200 μL). Purification by column chromatography (Eluent: petroleum ether/EtOAc = 8/1) afforded the title compound as a white solid (43 mg, 32% yield).
^{1}H NMR (500 MHz, $CDCl_3$) δ 4.26 (s, 4H), 3.25 (s, 4H), 2.86 (s, 4H), 1.86 (s, 4H); **^{13}C NMR** (126 MHz, $CDCl_3$) δ 170.3, 64.9, 33.6, 31.7, 25.6; **IR** (KBr, cm^{-1}) 2947, 1733, 1444, 1275, 964; **Mp**: 81.6 – 85.8 °C; **HRMS** (ESI) calcd. For $C_{10}H_{16}NaO_4S_2^+$ [M+Na]$^+$ m/z 287.0382, found: 287.0380.

1,4-Dioxa-8,11-dithiacyclotetradecane-5,14-dione (5)

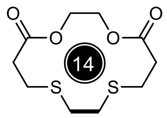

Prepared following the general procedure outlined above using [Ir[d(*t*Bu)(CF_3)ppy]$_2$(dtbbpy)[Cl] (5.4 mg, 5.0 μmol, 0.01 equiv.), ethane-1,2-diyl bis(3-mercaptopropanoate) (120 mg, 0.5 mmol, 1 equiv.), DMF (10 mL), *N,N*-diisopropylethylamine (97 mg, 0.75 mmol, 1.5 equiv.) and deionized water (200 μL). Purification by column chromatography (Eluent: petroleum ether/EtOAc = 8/1) afforded the title compound as a a clear oil (55 mg, 42% yield).

^{1}H NMR (500 MHz, $CDCl_3$) δ 4.40 (s, 4H), 2.91 (t, *J* = 5.0 Hz, 4H), 2.78 (s, 4H), 2.70 (t, *J* = 5.0 Hz, 4H); **^{13}C NMR** (126 MHz, $CDCl_3$) δ 171.7, 62.4, 35.7, 32.0, 27.4; **IR** (KBr, cm^{-1}) 2953, 1747, 1518, 1245, 1138; **HRMS** (ESI) calcd. For $C_{10}H_{16}NaO_4S_2^+$ $[M+Na]^+$ m/z 287.0382, found: 287.0384.

1,5-Dioxa-9,12-dithiacyclopentadecane-6,15-dione (6)

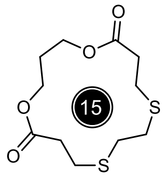

Prepared following the general procedure outlined above using [Ir[d(*t*Bu)(CF_3)ppy]$_2$(dtbbpy)[Cl] (2.7 mg, 2.5 μmol, 0.01 equiv.), propane-1,3-diyl bis(3-mercaptopropanoate) (63 mg, 0.25 mmol, 1 equiv.), DMF (5 mL), *N,N*-diisopropylethylamine (49 mg, 0.38 mmol, 1.5 equiv.) and deionized water (100 μL). Purification by column chromatography (Eluent: petroleum ether/EtOAc = 8/1) afforded the title compound as a white solid (21 mg, 30% yield).

^{1}H NMR (500 MHz, $CDCl_3$) δ 4.30 – 4.28 (m, 4H), 2.87 (t, *J* = 6.8 Hz, 4H), 2.73 (s, 4H), 2.65 (t, *J* = 6.8 Hz, 4H), 2.04 (dt, *J* = 10.9, 5.4 Hz, 2H); **^{13}C NMR** (126 MHz, CDCl3) δ 172.0, 61.9, 36.0, 32.4, 27.6, 27.3; **IR** (KBr, cm^{-1}) 3297, 2878, 1722, 1254, 1213; **Mp**: 79.6 – 82.9 °C; **HRMS** (ESI) calcd. For $C_{11}H_{18}NaO_4S_2^+$ $[M+Na]^+$ m/z 301.0539, found: 301.0535.

3-Methyl-1,5-dioxa-9,12-dithiacyclopentadecane-6,15-dione (7)

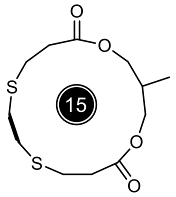

Prepared following the general procedure outlined above using [Ir[d(*t*Bu)(CF_3)ppy]$_2$(dtbbpy)[Cl] (2.7 mg, 2.5 μmol, 0.01 equiv.), 2-methylpropane-1,3-diyl bis(3-mercaptopropanoate) (67 mg, 0.25 mmol, 1 equiv.), DMF (5 mL), *N,N*-diisopropylethylamine (49 mg, 0.38 mmol, 1.5 equiv.) and deionized water (100 μL). Purification by column chromatography (Eluent: petroleum ether/EtOAc = 8/1) afforded the title compound as a white solid (20 mg, 27% yield).

^{1}H NMR (500 MHz, CDCl3) δ 4.24 (dd, *J* = 11.1, 3.4 Hz, 2H), 4.03 (dd, *J* = 11.0, 7.7 Hz, 2H), 2.87 (dd, *J* = 6.9, 6.3 Hz, 4H), 2.72 (s, 4H), 2.66 (t, *J* = 6.7 Hz, 4H), 2.33 – 2.15 (m, 1H), 1.02 (d, J = 7.1 Hz, 3H); **^{13}C NMR** (126 MHz, $CDCl_3$) δ 172.0, 66.2, 36.0, 32.5, 31.8, 27.6, 13.8; **IR** (KBr, cm^{-1}) 2895, 1738, 1339, 1203; **Mp**: 69.8 – 71.3 °C; **HRMS** (ESI) calcd. For $C_{12}H_{20}NaO_4S_2^+$ $[M+Na]^+$ m/z 315.0695, found: 315.0693.

(±)-2,4-Dimethyl-1,5-dioxa-9,12-dithiacyclopentadecane-6,15-dione (8)

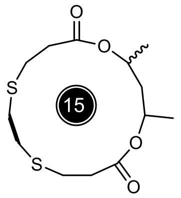

Prepared following the general procedure outlined above using [Ir[d(*t*Bu)(CF_3)ppy]$_2$(dtbbpy)[Cl] (2.7 mg, 2.5 μmol, 0.01 equiv.), (±)-pentane-2,4-diyl bis(3-mercaptopropanoate) (70 mg, 0.25 mmol, 1 equiv.), DMF (5 mL), *N,N*-diisopropylethylamine (49 mg, 0.38 mmol, 1.5 equiv.) and deionized water (100 μL). Purification by column chromatography (Eluent: petroleum ether/EtOAc = 8/1) afforded the title compound as a white solid (25 mg, 33% yield).

^{1}H NMR (500 MHz, CDCl3) δ 5.16 – 5.05 (m, 2H), 3.02 – 2.92 (m, 2H), 2.75 – 2.69 (m, 6H), 2.66 (dd, *J* = 10.6, 5.4 Hz, 2H), 2.64 – 2.56 (m, 2H), 1.84 (t, *J* = 5.0 Hz, 2H), 1.28 (s, 3H), 1.27 (s, 3H); **^{13}C NMR** (126 MHz, $CDCl_3$) δ 172.0, 67.7, 41.4, 36.6, 32.5, 27.0, 20.2; **IR** (KBr, cm^{-1}) 3146, 2923, 1738, 1398, 1385, 1338, 1133; **Mp**: 69.3 – 73.8 °C; **HRMS** (ESI) calcd. For $C_{13}H_{22}NaO_4S_2^+$ $[M+Na]^+$ m/z 329.0852, found: 329.0851.

3,3-Dimethyl-1,5-dioxa-9,12-dithiacyclopentadecane-6,15-dione (9)

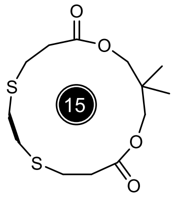

Prepared following the general procedure outlined above using [Ir[d(*t*Bu)(CF_3)ppy]$_2$(dtbbpy)[Cl] (2.7 mg, 2.5 μmol, 0.01 equiv.), 2,2-dimethylpropane-1,3-diyl bis(3-mercaptopropanoate) (70 mg, 0.25 mmol, 1 equiv.), DMF (5 mL), *N,N*-diisopropylethylamine (49 mg, 0.38 mmol, 1.5 equiv.) and deionized water (100 μL). Purification by column chromatography (Eluent: petroleum ether/EtOAc = 8/1) afforded the title compound as a white solid (24 mg, 32% yield).

^{1}H NMR (500 MHz, $CDCl_3$) δ 3.91 (s, 4H), 2.81 (m, 4H), 2.65 (s, 4H), 2.60 (m, 4H), 0.94 (s, 6H); **^{13}C NMR** (126 MHz, $CDCl_3$) δ 172.0, 69.2, 36.2, 34.1, 32.8, 27.7, 22.1; **IR** (KBr, cm^{-1}) 3168, 2962, 1734, 1401, 1129; **Mp**: 74.0 – 75.5 °C; **HRMS** (ESI) calcd. For $C_{13}H_{22}NaO_4S_2^+$ $[M+Na]^+$ m/z 329.0852, found: 329.0855.

1,12-Dioxa-5,8-dithiacyclohexadecane-2,11-dione (10)

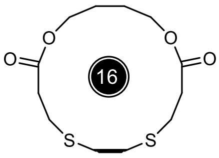

Prepared following the general procedure outlined above using [Ir[d(*t*Bu)(CF_3)ppy]$_2$(dtbbpy)[Cl] (2.7 mg, 2.5 μmol, 0.01 equiv.), butane-1,4-diyl bis(3-mercaptopropanoate) (67 mg, 0.25 mmol, 1 equiv.), DMF (5 mL), *N,N*-diisopropylethylamine (49 mg, 0.38 mmol, 1.5 equiv.) and deionized water (100 μL). Purification by column chromatography (Eluent: petroleum ether/EtOAc = 8/1) afforded the title compound as a white solid (26 mg, 36% yield).

^{1}H NMR (500 MHz, $CDCl_3$) δ 4.20 (s, 4H), 2.88 (t, *J* = 6.6 Hz, 4H), 2.77 (s, 4H), 2.64 (t, *J* = 6.6 Hz, 4H), 1.81 (s, 4H); **^{13}C NMR** (126 MHz, $CDCl_3$) δ 171.9, 64.2, 35.5, 31.9, 27.3, 25.4; **IR** (KBr,

cm^{-1}) 3294, 2889, 1732, 1560, 1241; **Mp**: 60.9 – 65.7 °C; **HRMS** (ESI) calcd. For $C_{12}H_{20}NaO_4S_2^+$ $[M+Na]^+$ m/z 315.0695, found: 315.0694.

1,12-Dioxa-5,8-dithiacycloheptadecane-2,11-dione (11)

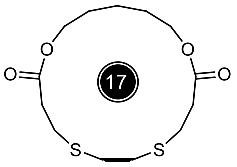

Prepared following the general procedure outlined above using [Ir[d(*t*Bu)(CF_3)ppy]$_2$(dtbbpy)[Cl] (2.7 mg, 2.5 μmol, 0.01 equiv.), pentane-1,5-diyl bis(3-mercaptopropanoate) (67 mg, 0.25 mmol, 1 equiv.), DMF (5 mL), *N,N*-diisopropylethylamine (49 mg, 0.38 mmol, 1.5 equiv.) and deionized water (100 μL). Purification by column chromatography (Eluent: petroleum ether/EtOAc = 8/1) afforded the title compound as a white solid (29 mg, 38% yield).

^{1}H NMR (500 MHz, $CDCl_3$) δ 4.10 (t, J = 5.0 Hz, 4H), 2.78 (t, J = 7.4 Hz, 4H), 2.69 (s, 4H), 2.57 (t, J = 7.4 Hz, 4H), 1.70 – 1.58 (m, 4H), 1.51 – 1.41 (m, 2H); **^{13}C NMR** (126 MHz, $CDCl_3$) δ 171.6, 64.5, 35.5, 32.4, 28.2, 27.6, 23.2; **IR** (KBr, cm^{-1}) 3158, 2951, 1720, 1400, 1253, 1203, 1166; **Mp**: 83.8 – 86.2 °C; **HRMS** (ESI) calcd. For $C_{13}H_{22}NaO_4S_2^+$ $[M+Na]^+$ m/z 329.0852, found: 329.0849.

1,12-Dioxa-5,8-dithiacyclooctadecane-2,11-dione (12)

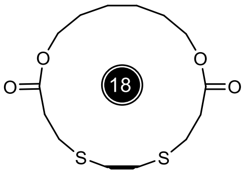

Prepared following the general procedure outlined above using [Ir[d(*t*Bu)(CF_3)ppy]$_2$(dtbbpy)[Cl] (2.7 mg, 2.5 μmol, 0.01 equiv.), hexane-1,6-diyl bis(3-mercaptopropanoate) (74 mg, 0.25 mmol, 1 equiv.), DMF (5 mL), *N,N*-diisopropylethylamine (49 mg, 0.38 mmol, 1.5 equiv.) and deionized water (100 μL). Purification by column chromatography (Eluent: petroleum ether:EtOAc = 8/1) afforded the title compound as a white solid (19 mg, 23% yield).

^{1}H NMR (500 MHz, $CDCl_3$) δ 4.17 (t, J = 5.6 Hz, 4H), 2.85 (t, J = 7.0 Hz, 4H), 2.76 (s, 4H), 2.64 (t, J = 7.0 Hz, 4H), 1.68 (t, J = 5.0 Hz, 4H), 1.48 (t, J = 3.0 Hz, 4H); **^{13}C NMR** (126 MHz, $CDCl_3$) δ 171.9, 64.6, 35.4, 31.8, 28.3, 27.1, 25.8; **IR** (KBr, cm^{-1}) 3148, 2926, 1729, 1583, 1396, 1125; **Mp**: 58.2 – 59.7 °C; **HRMS** (ESI) calcd. For $C_{14}H_{24}NaO_4S_2^+$ $[M+Na]^+$ m/z 343.1008, found: 343.1007.

1,10-Dioxa-4,7-dithiacyclooctadecane-11,18-dione (13)

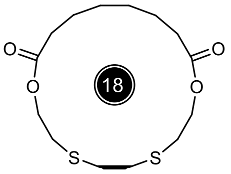

Prepared following the general procedure outlined above using [Ir[d(*t*Bu)(CF_3)ppy]$_2$(dtbbpy)[Cl] (2.7 mg, 2.5 μmol, 0.01 equiv.), bis(2-mercaptoethyl) octanedioate (74 mg, 0.25 mmol, 1 equiv.), DMF (5 mL), *N,N*-diisopropylethylamine (49 mg, 0.38 mmol, 1.5 equiv.) and deionized water (100 μL). Purification by column chromatography (Eluent: petroleum ether/EtOAc = 8/1) afforded the title compound as a white solid (22 mg, 28% yield).

^{1}H NMR (500 MHz, $CDCl_3$) δ 4.29 (t, *J* = 6.2 Hz, 4H), 2.85 (s, 4H), 2.81 (t, *J* = 6.2 Hz, 4H), 2.35 (t, *J* = 6.9 Hz, 4H), 1.73 – 1.64 (m, 4H), 1.39 (dd, *J* = 8.1, 5.4 Hz, 4H); **^{13}C NMR** (126 MHz, CDCl3) δ 173.4, 64.3, 33.9, 32.3, 30.5, 27.6, 24.3; **IR** (KBr, cm^{-1}) 3177, 2957, 1747, 1399, 1135; **Mp**: 84.6 – 85.6 °C; **HRMS** (ESI) calcd. For $C_{14}H_{24}NaO_4S_2^+$ [M+Na]$^+$ m/z 343.1008, found: 343.1003.

1,12-Dioxa-5,8-dithiacyclononadecane-2,11-dione (14)

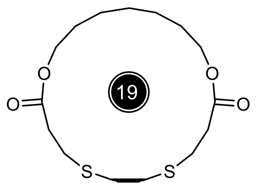

Prepared following the general procedure outlined above using [Ir[d(*t*Bu)(CF_3)ppy]$_2$(dtbbpy)[Cl] (2.7 mg, 2.5 μmol, 0.01 equiv.), heptane-1,7-diyl bis(3-mercaptopropanoate) (77 mg, 0.25 mmol, 1 equiv.), DMF (5 mL), *N,N*-diisopropylethylamine (49 mg, 0.38 mmol, 1.5 equiv.) and deionized water (100 μL). Purification by column chromatography (Eluent: petroleum ether/EtOAc = 8/1) afforded the title compound as a white solid (21 mg, 26% yield).

^{1}H NMR (500 MHz, $CDCl_3$) δ 4.29 (t, *J* = 6.2 Hz, 4H), 2.85 (s, 4H), 2.81 (t, *J* = 6.2 Hz, 4H), 2.35 (t, *J* = 6.9 Hz, 4H), 1.73 – 1.64 (m, 4H), 1.39 (dd, *J* = 8.1, 5.4 Hz, 4H); **^{13}C NMR** (126 MHz, $CDCl_3$) δ 171.8, 64.5, 35.4, 32.5, 28.2, 27.9, 27.7, 25.5; **IR** (KBr, cm^{-1}) 2946, 1731, 1506, 1149; **Mp**: 65.1 – 66.2 °C; **HRMS** (ESI) calcd. For $C_{15}H_{26}NaO_4S_2^+$ [M+Na]$^+$ m/z 357.1165, found: 357.1164.

1,12-Dioxa-5,8-dithiacycloicosane-2,11-dione (15)

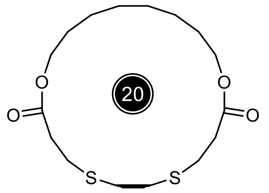

Prepared following the general procedure outlined above using [Ir[d(*t*Bu)(CF_3)ppy]$_2$(dtbbpy)[Cl] (2.7 mg, 2.5 μmol, 0.01 equiv.), octane-1,8-diyl bis(3-mercaptopropanoate) (80 mg, 0.25 mmol, 1 equiv.), DMF (5 mL), *N,N*-diisopropylethylamine (49 mg, 0.38 mmol, 1.5 equiv.) and deionized water (100 μL). Purification by column chromatography (Eluent: petroleum ether/EtOAc = 8/1) afforded the title compound as a white solid (19 mg, 22% yield).

^{1}H NMR (500 MHz, $CDCl_3$) δ 4.15 (t, *J* = 5.6 Hz, 4H), 2.85 (t, *J* = 7.1 Hz, 4H), 2.75 (s, 4H), 2.63 (t, *J* = 7.1 Hz, 4H), 1.69 – 1.60 (m, 4H), 1.45 – 1.32 (m, 8H); **^{13}C NMR** (126 MHz, $CDCl_3$) δ 166.5, 59.6, 29.9, 26.9, 23.5, 23.1, 22.2, 20.5; **IR** (KBr, cm^{-1}) 2921, 1733, 1581, 1389, 1142, 1004; **Mp**: 72.8 – 74.3 °C; **HRMS** (ESI) calcd. For $C_{16}H_{28}NaO_4S_2^+$ [M+Na]$^+$ m/z 371.1321, found: 371.1322.

1,12-Dioxa-5,8-dithiacyclohenicosane-2,11-dione (16)

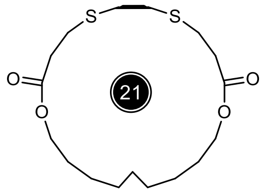

Prepared following the general procedure outlined above using [Ir[d(*t*Bu)(CF_3)ppy]$_2$(dtbbpy)[Cl] (2.7 mg, 2.5 μmol, 0.01 equiv.), nonane-1,9-diyl bis(3-mercaptopropanoate) (84 mg, 0.25 mmol, 1 equiv.), DMF (5 mL), *N,N*-diisopropylethylamine (49 mg, 0.38 mmol, 1.5 equiv.) and deionized

water (100 μL). Purification by column chromatography (Eluent: petroleum ether/EtOAc = 8/1) afforded the title compound as a white solid (22 mg, 24% yield).
^{1}H NMR (500 MHz, $CDCl_3$) δ 4.15 (t, *J* = 6.0 Hz, 4H), 2.85 (t, *J* = 6.9 Hz, 4H), 2.75 (s, 4H), 2.62 (t, *J* = 6.9 Hz, 4H), 1.67 – 1.59 (m, 4H), 1.43 – 1.30 (m, 10H); **^{13}C NMR** (126 MHz, $CDCl_3$) δ 171.8, 64.6, 35.4, 32.4, 28.7, 28.2, 28.0, 27.7, 25.5; **IR** (KBr, cm^{-1}) 3145, 2928, 1732, 1514, 1395, 1241; **Mp**: 45.9 – 46.7 °C; **HRMS** (ESI) calcd. For $C_{17}H_{30}NaO_4S_2^+$ $[M+Na]^+$ m/z 385.1478, found: 385.1477.

1,12-Dioxa-5,8-dithiacyclodocosane-2,11-dione (17)

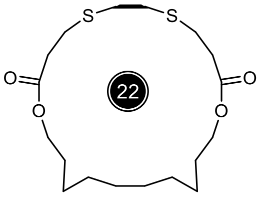

Prepared following the general procedure outlined above using [Ir[d(*t*Bu)(CF_3)ppy]$_2$(dtbbpy)[Cl] (2.7 mg, 2.5 μmol, 0.01 equiv.), decane-1,10-diyl bis(3-mercaptopropanoate) (88 mg, 0.25 mmol, 1 equiv.), DMF (5 mL), *N,N*-diisopropylethylamine (49 mg, 0.38 mmol, 1.5 equiv.) and deionized water (100 μL). Purification by column chromatography (Eluent: petroleum ether/EtOAc = 8/1) afforded the title compound as a white solid (19 mg, 20% yield).
^{1}H NMR (500 MHz, $CDCl_3$) δ 4.15 (t, *J* = 5.9 Hz, 4H), 2.84 (t, *J* = 7.1 Hz, 4H), 2.75 (s, 4H), 2.62 (t, *J* = 7.1 Hz, 4H), 1.69 – 1.58 (m, 4H), 1.43 – 1.36 (m, 4H), 1.35 – 1.26 (m, 8H); **^{13}C NMR** (126 MHz, $CDCl_3$) δ 171.8, 64.8, 35.2, 32.1, 28.7, 28.7, 28.4, 27.4, 25.8; **IR** (KBr, cm^{-1}) 3145, 2928, 1732, 1514, 1395, 1241; **Mp**: 45.9 – 46.7 °C; **HRMS** (ESI) calcd. For $C_{17}H_{30}NaO_4S_2^+$ $[M+Na]^+$ m/z 385.1478, found: 385.1477.

1,12-Dioxa-5,8-dithiacyclotetracosane-2,11-dione (18)

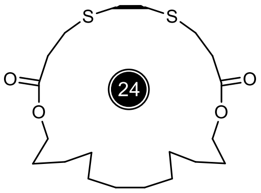

Prepared following the general procedure outlined above using [Ir[d(*t*Bu)(CF_3)ppy]$_2$(dtbbpy)[Cl] (2.7 mg, 2.5 μmol, 0.01 equiv.), dodecane-1,12-diyl bis(3-mercaptopropanoate) (95 mg, 0.25 mmol, 1 equiv.), DMF (5 mL), *N,N*-diisopropylethylamine (49 mg, 0.38 mmol, 1.5 equiv.) and deionized water (100 μL). Purification by column chromatography (Eluent: petroleum ether/EtOAc = 8/1) afforded the title compound as a white solid (25 mg, 25% yield).
^{1}H NMR (500 MHz, $CDCl_3$) δ 4.14 (t, *J* = 6.0 Hz, 4H), 2.84 (t, *J* = 7.1 Hz, 4H), 2.75 (s, 4H), 2.62 (t, *J* = 7.1 Hz, 4H), 1.72 – 1.57 (m, 4H), 1.38 (dd, *J* = 14.1, 6.7 Hz, 4H), 1.31 (d, *J* = 20.3 Hz, 12H); **^{13}C NMR** (126 MHz, $CDCl_3$) δ 171.8, 64.8, 35.2, 32.0, 28.7, 28.7, 28.5, 28.4, 27.4, 25.7; **IR** (KBr, cm^{-1}) 2932, 1733, 1452, 1188; **Mp**: 58.7 – 60.9 °C; **HRMS** (ESI) calcd. For $C_{20}H_{36}NaO_4S_2^+$ $[M+Na]^+$ m/z 427.1947, found: 427.1946.

1,12-Dioxa-5,8-dithiacyclohexacosane-2,11-dione (19)

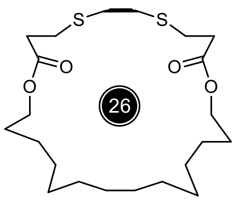

Prepared following the general procedure outlined above using [Ir[d(*t*Bu)(CF_3)ppy]$_2$(dtbbpy)[Cl] (2.7 mg, 2.5 μmol, 0.01 equiv.), tetradecane-1,14-diyl bis(3-mercaptopropanoate) (101 mg, 0.25 mmol, 1 equiv.), DMF (5 mL), *N,N*-diisopropylethylamine (49 mg, 0.38 mmol, 1.5 equiv.) and deionized water (100 μL). Purification by column chromatography (Eluent: petroleum ether/EtOAc = 8/1) afforded the title compound as a white solid (23 mg, 21% yield).

^{1}H NMR (500 MHz, $CDCl_3$) δ 4.13 (dd, *J* = 5.9, 5.4 Hz, 4H), 2.83 (t, *J* = 6.8 Hz, 4H), 2.75 (d, *J* = 1.3 Hz, 4H), 2.62 (t, *J* = 6.8 Hz, 4H), 1.68 – 1.60 (m, 4H), 1.41 – 1.35 (m, 4H), 1.33 – 1.25 (m, 16H); **^{13}C NMR** (126 MHz, $CDCl_3$) δ 171.8, 64.7, 35.1, 32.1, 28.8, 28.7, 28.6, 28.5, 28.3, 27.3, 25.6; **IR** (KBr, cm^{-1}) 2894, 1747, 1506, 1338, 1266; **Mp**: 61.6 – 62.8 °C; **HRMS** (ESI) calcd. For $C_{20}H_{36}NaO_4S_2^+$ [M+Na]$^+$ m/z 427.1947, found: 427.1946.

3,3,4,4-Tetrafluoro-1,6-dioxa-13,16-dithiacyclodocosane-2,5-dione (20)

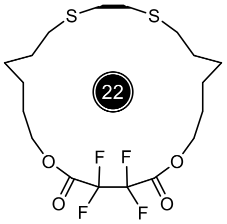

Prepared following the general procedure outlined above using [Ir[d(*t*Bu)(CF_3)ppy]$_2$(dtbbpy)[Cl] (2.7 mg, 2.5 μmol, 0.01 equiv.), bis(6-mercaptohexyl) 2,2,3,3-tetrafluorosuccinate (106 mg, 0.25 mmol, 1 equiv.), DMF (5 mL), *N,N*-diisopropylethylamine (49 mg, 0.38 mmol, 1.5 equiv.) and deionized water (100 μL). Purification by column chromatography (Eluent: petroleum ether/EtOAc = 10/1) afforded the title compound as a yellowish liquid (40 mg, 33% yield).

^{1}H NMR (500 MHz, $CDCl_3$) δ 4.38 (t, *J* = 6.1 Hz, 4H), 2.71 (s, 4H), 2.56 (t, *J* = 7.4 Hz, 4H), 1.79 – 1.72 (m, 4H), 1.67 – 1.59 (m, 4H), 1.51 – 1.39 (m, 8H); **^{13}C NMR** (126 MHz, $CDCl_3$) δ 159.3 (t, $^2J_{CF}$ = 30.2Hz), 110.2 (t, $^2J_{CF}$ = 31.5 Hz), 108.1 (tt, $^1J_{CF}$ = 265.9 Hz, $^2J_{CF}$ = 26.5 Hz), 106.0 (t, $^2J_{CF}$ = 32.8 Hz), 68.0, 32.1, 31.7, 29.4, 28.0, 27.8, 25.1; **^{19}F NMR** (471 MHz, $CDCl_3$) δ -119.8; **IR** (KBr, cm^{-1}) 2944, 1541, 1272, 899; **HRMS** (ESI) calcd. For $C_{18}H_{28}F_4NaO_4S_2^+$ [M+Na]$^+$ m/z 471.1257, found: 471.1256.

3,3,4,4,5,5,6,6-Octafluoro-1,8-dioxa-15,18-dithiacyclotetracosane-2,7-dione (21)

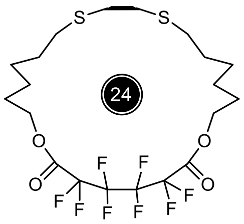

Prepared following the general procedure outlined above using [Ir[d(*t*Bu)(CF_3)ppy]$_2$(dtbbpy)[Cl] (2.7 mg, 2.5 μmol, 0.01 equiv.), bis(6-mercaptohexyl) 2,2,3,3,4,4,5,5-octafluorohexanedioate (130 mg, 0.25 mmol, 1 equiv.), DMF (5 mL), *N,N*-diisopropylethylamine (49 mg, 0.38 mmol, 1.5 equiv.)

and deionized water (100 μL). Purification by column chromatography (Eluent: petroleum ether/EtOAc = 10/1) afforded the title compound as a yellowish liquid (42 mg, 31% yield).
^{1}H NMR (500 MHz, $CDCl_3$) δ 4.33 (t, *J* = 6.0 Hz, 4H), 2.64 (s, 4H), 2.49 (t, *J* = 7.3 Hz, 4H), 1.72 – 1.64 (m, 4H), 1.59 – 1.51 (m, 4H), 1.42 – 1.32 (m, 8H); **^{13}C NMR** (126 MHz, $CDCl_3$) δ 157.5 (t, $^2J_{CF}$ = 29.0 Hz), 111.6 (t, $^2J_{CF}$ = 34.0 Hz), 109.4 (m),107.0 (m), 104.7 (t, $^2J_{CF}$ = 34.0 Hz), 104.5, 67.4, 31.1, 30.7, 28.2, 26.8, 24.0; **^{19}F NMR** (471 MHz, $CDCl_3$) δ -118.7, -118.7, -118.8, -122.8, -122.8, -122.8; **IR** (KBr, cm^{-1}) 2945, 1778, 1506, 1489, 1198; **HRMS** (ESI) calcd. For $C_{20}H_{28}F_8NaO_4S_2^+$ $[M+Na]^+$ m/z 571.1193, found: 571.1192.

1,4,7-Trioxa-14,17-dithiacyclotricosane-2,6-dione (22)

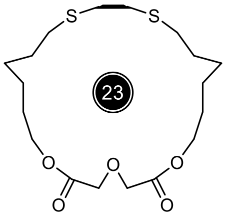

Prepared following the general procedure outlined above using [Ir[d(*t*Bu)(CF_3)ppy]$_2$(dtbbpy)[Cl] (2.7 mg, 2.5 μmol, 0.01 equiv.), bis(6-mercaptohexyl) 2,2'-oxydiacetate (92 mg, 0.25 mmol, 1 equiv.), DMF (5 mL), *N,N*-diisopropylethylamine (49 mg, 0.38 mmol, 1.5 equiv.) and deionized water (100 μL). Purification by column chromatography (Eluent: petroleum ether/EtOAc = 8/1) afforded the title compound as a yellowish liquid (23 mg, 23% yield).
^{1}H NMR (500 MHz, $CDCl_3$) δ 4.29 – 4.17 (m, 8H), 2.72 (s, 4H), 2.56 (t, *J* = 7.3 Hz, 4H), 1.72 – 1.65 (m, 4H), 1.65 – 1.59 (m, 4H), 1.50 – 1.38 (m, 8H); **^{13}C NMR** (126 MHz, $CDCl_3$) δ 169.8, 68.4, 65.0, 32.3, 31.9, 29.6, 28.3, 28.1, 25.6; **IR** (KBr, cm^{-1}) 2897, 1747, 1489, 1338, 1210; **HRMS** (ESI) calcd. For $C_{18}H_{32}NaO_5S_2^+$ $[M+Na]^+$ m/z 415.1583, found: 415.1578.

1,4,7-Trioxa-11,14-dithiacycloheptadecane-8,17-dione (23)

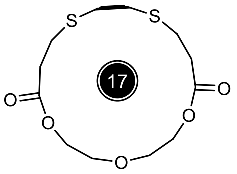

Prepared following the general procedure outlined above using [Ir[d(*t*Bu)(CF_3)ppy]$_2$(dtbbpy)[Cl] (2.7 mg, 2.5 μmol, 0.01 equiv.), oxybis(ethane-2,1-diyl) bis(3-mercaptopropanoate) (71 mg, 0.25 mmol, 1 equiv.), DMF (5 mL), *N,N*-diisopropylethylamine (49 mg, 0.38 mmol, 1.5 equiv.) and deionized water (100 μL). Purification by column chromatography (Eluent: petroleum ether/EtOAc = 4/1) afforded the title compound as a white solid (22 mg, 29% yield).
^{1}H NMR (500 MHz, $CDCl_3$) δ 4.29 (m, 4H), 3.71 (m, 4H), 2.85 (t, *J* = 7.5 Hz, 4H), 2.75 (s, 4H), 2.67 (t, *J* = 7.6 Hz, 4H); **^{13}C NMR** (126 MHz, $CDCl_3$) δ171.5, 68.9, 63.6, 35.3, 31.8, 27.1; **IR** (KBr, cm^{-1}) 3126, 2917, 1747, 1506, 1399, 1129; **Mp**: 77.5 – 79.1 °C; **HRMS** (ESI) calcd. For $C_{12}H_{20}NaO_5S_2^+$ $[M+Na]^+$ m/z 331.0644, found: 331.0638.

1,4,7,10-Tetraoxa-14,17-dithiacycloicosane-11,20-dione (24)

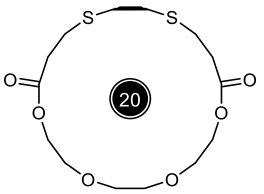

Prepared following the general procedure outlined above using [Ir[d(*t*Bu)(CF_3)ppy]$_2$(dtbbpy)[Cl] (2.7 mg, 2.5 μmol, 0.01 equiv.), (ethane-1,2-diylbis(oxy))bis(ethane-2,1-diyl) bis(3-mercaptopropanoate) (82 mg, 0.25 mmol, 1 equiv.), DMF (5 mL), *N,N*-diisopropylethylamine (49 mg, 0.38 mmol, 1.5 equiv.) and deionized water (100 μL). Purification by column chromatography (Eluent: petroleum ether/EtOAc = 3/1) afforded the title compound as a colorless oil (25 mg, 28% yield).

^{1}H NMR (500 MHz, $CDCl_3$) δ 4.29 (m, 4H), 3.72 (m, 4H), 3.67 (s, 4H), 2.86 (t, *J* = 7.3 Hz, 4H), 2.75 (s, 4H), 2.68 (t, *J* = 7.2 Hz, 4H); **^{13}C NMR** (126 MHz, $CDCl_3$) δ 171.7, 70.7, 69.1, 64.0, 35.3, 32.0, 27.1; **IR** (KBr, cm^{-1}) 3301, 2949, 1731, 1339, 1242, 1112; **Mp**: 51.8 – 54.5 °C; **HRMS** (ESI) calcd. For $C_{14}H_{24}NaO_6S_2^+$ [M+Na]$^+$ m/z 375.0907, found: 375.0909.

1,4,7,10,13-Pentaoxa-17,20-dithiacyclotricosane-14,23-dione (25)

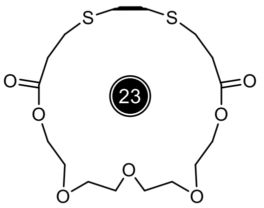

Prepared following the general procedure outlined above using [Ir[d(*t*Bu)(CF_3)ppy]$_2$(dtbbpy)[Cl] (2.7 mg, 2.5 μmol, 0.01 equiv.), (ethane-1,2-diylbis(oxy))bis(ethane-2,1-diyl) bis(3-mercaptopropanoate) (82 mg, 0.25 mmol, 1 equiv.), DMF (5 mL), *N,N*-diisopropylethylamine (49 mg, 0.38 mmol, 1.5 equiv.) and deionized water (100 μL). Purification by column chromatography (Eluent: petroleum ether/EtOAc = 3/1) afforded the title compound as a colorless oil (25 mg, 28% yield).

^{1}H NMR (500 MHz, $CDCl_3$) δ 4.28 (dd, *J* = 4.2, 2.6 Hz, 4H), 3.73 (dd, *J* = 4.4, 2.8 Hz, 4H), 3.67 (s, 8H), 2.85 (t, *J* = 7.0 Hz, 4H), 2.75 (s, 4H), 2.67 (t, *J* = 7.1 Hz, 4H); **^{13}C NMR** (126 MHz, $CDCl_3$) δ 171.8, 70.8, 70.8, 69.1, 64.0, 35.3, 32.1, 27.1; **IR** (KBr, cm^{-1}) 3186, 2918, 1730, 1456, 1377, 1108; **HRMS** (ESI) calcd. For $C_{16}H_{29}O_7S_2^+$ [M+H]$^+$ m/z 397.1349, found: 397.1351.

1,4,7,10,13,16,19,22,25-Nonaoxa-28,31-dithiacyclotetratriacontane-26,34-dione (26)

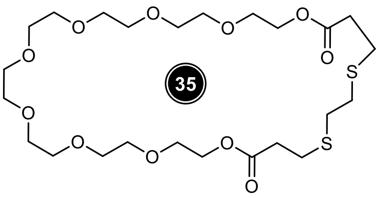

Prepared following the general procedure outlined above using [Ir[d(*t*Bu)(CF_3)ppy]$_2$(dtbbpy)[Cl] (2.7 mg, 2.5 μmol, 0.01 equiv.), 26-mercapto-25-oxo-3,6,9,12,15,18,21,24-octaoxahexacosyl 3-mercaptopropanoate (133 mg, 0.25 mmol, 1 equiv.), DMF (5 mL), *N,N*-diisopropylethylamine (49 mg, 0.38 mmol, 1.5 equiv.) and deionized water (100 μL). Purification by column chromatography (Eluent: EtOAc) afforded the title compound as a colorless oil (26 mg, 18% yield).

^{1}H NMR (500 MHz, $CDCl_3$) δ 4.20 (m, 4H), 3.65 (m, 4H), 3.59 (s, 24H), 2.77 (t, *J* = 7.3 Hz, 4H),

2.69 (s, 4H), 2.60 (t, *J* = 7.3 Hz, 4H); **^{13}C NMR** (126 MHz, $CDCl_3$) δ 171.8, 70.7, 70.6, 70.6, 70.5, 69.0, 63.9, 35.0, 32.0, 27.0; **IR** (KBr, cm^{-1}) 3304, 2946, 1440, 1240, 1100; **HRMS** (ESI) calcd. For $C_{24}H_{45}O_{11}S_2^+$ $[M+H]^+$ m/z 573.2398, found: 573.2395.

4-Phenyl-1,7-dioxa-11,14-dithia-4-azacycloheptadecane-8,17-dione (27)

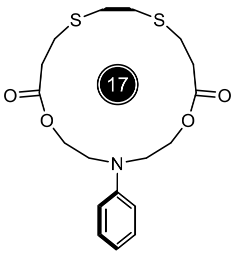

Prepared following the general procedure outlined above using $[Ir[d(tBu)(CF_3)ppy]_2(dtbbpy)[Cl]$ (2.7 mg, 2.5 μmol, 0.01 equiv.), (phenylazanediyl)bis(ethane-2,1-diyl) bis(3-mercaptopropanoate) (89 mg, 0.25 mmol, 1 equiv.), DMF (5 mL), *N,N*-diisopropylethylamine (49 mg, 0.38 mmol, 1.5 equiv.) and deionized water (100 μL). Purification by column chromatography (Eluent: petroleum ether/EtOAc = 4/1) afforded the title compound as a white solid (36 mg, 38% yield).
^{1}H NMR (500 MHz, $CDCl_3$) δ 7.24 (t, *J* = 7.8 Hz, 2H), 6.75 (d, *J* = 7.3 Hz, 1H), 6.71 (d, *J* = 8.4 Hz, 2H), 4.32 (t, *J* = 6.0 Hz, 4H), 3.70 (t, *J* = 6.0 Hz, 4H), 2.86 (t, *J* = 6.8 Hz, 4H), 2.77 (s, 4H), 2.69 (t, *J* = 6.8 Hz, 4H); **^{13}C NMR** (126 MHz, $CDCl_3$) δ 172.1, 146.9, 129.6, 117.2, 112.0, 62.5, 50.5, 35.8, 32.3, 27.0; **IR** (KBr, cm^{-1}) 3117, 1747, 1396, 1130; **Mp**: 100.3 – 101.8 °C; **HRMS** (ESI) calcd. For $C_{18}H_{26}NO_4S_2^+$ $[M+H]^+$ m/z 384.1298, found: 384.1295.

4-Methyl-1,7-dioxa-14,17-dithia-4-azacyclotricosane-2,6-dione (28)

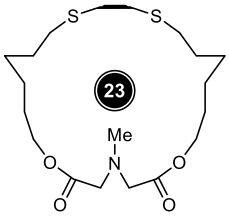

Prepared following the general procedure outlined above using $[Ir[d(tBu)(CF_3)ppy]_2(dtbbpy)[Cl]$ (2.7 mg, 2.5 μmol, 0.01 equiv.), bis(6-mercaptohexyl) 2,2'-(methylazanediyl)diacetate (95 mg, 0.25 mmol, 1 equiv.), DMF (5 mL), *N,N*-diisopropylethylamine (49 mg, 0.38 mmol, 1.5 equiv.) and deionized water (100 μL). Purification by column chromatography (Eluent: petroleum ether/EtOAc = 4/1) afforded the title compound as a colourless oil (24 mg, 24% yield).
^{1}H NMR (500 MHz, $CDCl_3$) δ 4.10 (t, *J* = 6.0 Hz, 4H), 3.42 (s, 4H), 2.65 (s, 4H), 2.49 (t, *J* = 7.5 Hz, 7H), 1.66 – 1.50 (m, 8H), 1.44 – 1.29 (m, 8H); **^{13}C NMR** (126 MHz, $CDCl_3$) δ 1170.9, 64.5, 57.4, 42.5, 32.2, 32.0, 29.7, 28.4, 28.1, 25.7; **IR** (KBr, cm^{-1}) 2922, 2855, 1747, 1731, 1454, 1180; **HRMS** (ESI) calcd. For $C_{19}H_{35}NNaO_4S_2^+$ $[M+Na]^+$ m/z 428.1900, found: 428.1903

***trans*-3,20-Dioxa-10,13-dithiabicyclo[20.2.2]hexacosane-2,21-dione (29)**

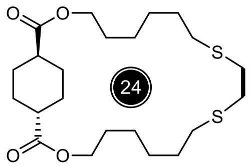

Prepared following the general procedure outlined above using [Ir[d(*t*Bu)(CF_3)ppy]$_2$(dtbbpy)[Cl] (2.7 mg, 2.5 μmol, 0.01 equiv.), bis(6-mercaptohexyl) cyclohexane-1,4-dicarboxylate (101 mg, 0.25 mmol, 1 equiv.), DMF (5 mL), *N,N*-diisopropylethylamine (49 mg, 0.38 mmol, 1.5 equiv.) and deionized water (100 μL). Purification by column chromatography (Eluent: petroleum ether/EtOAc = 10/1) afforded the title compound as a white solid (28 mg, 26% yield).

^{1}H NMR (500 MHz, $CDCl_3$) δ 4.18 – 4.05 (m, 4H), 2.70 (s, 4H), 2.58 (t, *J* = 7.2 Hz, 4H), 2.31 (s, 2H), 2.09 (d, *J* = 7.5 Hz, 4H), 1.68 – 1.58 (m, 8H), 1.55 – 1.41 (m, 12H); **^{13}C NMR** (126 MHz, $CDCl_3$) δ 175.3, 64.3, 42.7, 32.7, 31.9, 28.8, 28.1, 28.0, 27.7, 25.7; **IR** (KBr, cm^{-1}) 3296, 2942, 1721, 1265, 741; **Mp**: 99.0 – 102.2 °C; **HRMS** (ESI) calcd. For $C_{22}H_{38}NaO_4S_2^+$ $[M+Na]^+$ m/z 453.2104, found: 453.2096.

3,20-Dioxa-10,13-dithiatricyclo[20.2.2.2^{1,22}]octacosane-2,21-dione (30)

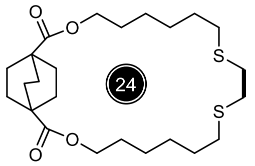

Prepared following the general procedure outlined above using [Ir[d(*t*Bu)(CF_3)ppy]$_2$(dtbbpy)[Cl] (2.7 mg, 2.5 μmol, 0.01 equiv.), bis(6-mercaptohexyl) bicyclo[2.2.2]octane-1,4-dicarboxylate (108 mg, 0.25 mmol, 1 equiv.), DMF (5 mL), *N,N*-diisopropylethylamine (49 mg, 0.38 mmol, 1.5 equiv.) and deionized water (100 μL). Purification by column chromatography (Eluent: petroleum ether/EtOAc = 10/1) afforded the title compound as a white solid (32 mg, 28% yield).

^{1}H NMR (500 MHz, $CDCl_3$) δ 4.08 – 3.98 (m, 4H), 2.64 (s, 4H), 2.52 (t, J = 7.2 Hz, 4H), 1.78 (s, 12H), 1.60 – 1.51 (m, 8H), 1.43 – 1.31 (m, 8H); **^{13}C NMR** (126 MHz, $CDCl_3$) δ 177.2, 64.3, 38.8, 32.8, 32.0, 28.7, 27.9, 27.9, 27.6, 25.6; **IR** (KBr, cm^{-1}) 2946, 1747, 1514, 1462, 1368, 1256; **Mp**: 78.9 – 81.6 °C; **HRMS** (ESI) calcd. For $C_{24}H_{41}O_4S_2^+$ $[M+H]^+$ m/z 457.2441, found: 457.2431.

3,20-Dioxa-10,13-dithiatetracyclo[20.5.1.1^{1,24}.12^{2,26}]triacontane-2,21-dione (31)

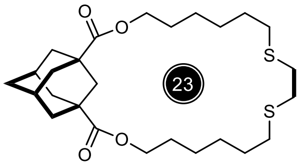

Prepared following the general procedure outlined above using [Ir[d(*t*Bu)(CF_3)ppy]$_2$(dtbbpy)[Cl] (2.7 mg, 2.5 μmol, 0.01 equiv.), bis(6-mercaptohexyl)-adamantane-1,3-dicarboxylate (114 mg, 0.25 mmol, 1 equiv.), DMF (5 mL), *N,N*-diisopropylethylamine (49 mg, 0.38 mmol, 1.5 equiv.) and deionized water (100 μL). Purification by column chromatography (Eluent: petroleum ether/EtOAc = 10/1) afforded the title compound as a colorless oil (39 mg, 32% yield).

^{1}H NMR (500 MHz, $CDCl_3$) δ 4.09 (t, *J* = 6.0 Hz, 4H), 2.72 (s, 4H), 2.55 (t, *J* = 7.3 Hz, 4H), 2.17 (s, 2H), 2.02 (s, 2H), 1.87 (s, 8H), 1.72 – 1.56 (m, 10H), 1.42 (dq, *J* = 17.4, 6.8, 6.0 Hz, 8H); **^{13}C NMR** (126 MHz, $CDCl_3$) δ176.8, 64.2, 41.0, 40.0, 38.0, 35.3, 32.3, 32.1, 29.8, 28.4, 28.2, 27.8, 25.5; **IR** (KBr, cm^{-1}) 2951, 1747, 1513, 1396, 1094; **HRMS** (ESI) calcd. For $C_{26}H_{42}NaO_4S_2^+$ $[M+Na]^+$ m/z 505.2417, found: 505.2418.

4,15-Dioxa-8,11-dithia-1,18-diazabicyclo[16.2.2]docosane-5,14-dione (32)

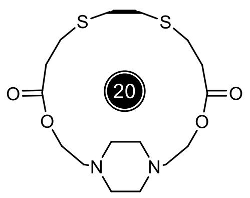

Prepared following the general procedure outlined above using [Ir[d(*t*Bu)(CF$_3$)ppy]$_2$(dtbbpy)[Cl] (2.7 mg, 2.5 μmol, 0.01 equiv.), piperazine-1,4-diylbis(ethane-2,1-diyl) bis(3-mercaptopropanoate) (88 mg, 0.25 mmol, 1 equiv.), DMF (5 mL), *N,N*-diisopropylethylamine (49 mg, 0.38 mmol, 1.5 equiv.) and deionized water (100 μL). Purification by column chromatography (Eluent: DCM/CH$_3$OH = 35/1) afforded the title compound as a yellowish solid (24 mg, 21% yield).
^{1}H NMR (500 MHz, CDCl$_3$) δ 4.22 (m, 4H), 2.79 (t, *J* = 7.0 Hz, 4H), 2.68 (d, *J* = 5.6 Hz, 8H), 2.60 – 2.50 (m, 12H); **^{13}C NMR** (126 MHz, CDCl$_3$) δ 171.5, 62.5, 56.0, 52.9, 35.0, 32.3, 28.2; **IR** (KBr, cm^{-1}) 3444, 3159, 2933, 1732, 1634, 1400, 1385, 1228, 1147; **Mp**: 105.0 – 108.2 °C; **HRMS** (ESI) calcd. For $C_{16}H_{29}N_2O_4S_2^+$ [M+H]$^+$ m/z 377.1563, found: 377.1559.

2,3,5,6,7,8,9,10-Octahydro-12*H*-benzo[*c*][1]oxa[5,8]dithiacyclotetradecin-12-one (33)

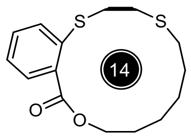

Prepared following the general procedure outlined above using [Ir[d(*t*Bu)(CF$_3$)ppy]$_2$(dtbbpy)[Cl] (2.7 mg, 2.5 μmol, 0.01 equiv.), 6-mercaptohexyl 2-mercaptobenzoate (68 mg, 0.25 mmol, 1 equiv.), DMF (5 mL), *N,N*-diisopropylethylamine (49 mg, 0.38 mmol, 1.5 equiv.) and deionized water (100 μL). Purification by column chromatography (Eluent: petroleum ether/EtOAc = 15/1) afforded the title compound as a yellowish solid (19 mg, 25% yield).
^{1}H NMR (500 MHz, CDCl$_3$) δ 7.70 (d, *J* = 7.5 Hz, 1H), 7.32 (d, *J* = 5.6 Hz, 2H), 7.19 – 7.13 (m, 1H), 4.46 – 4.23 (m, 2H), 3.16 (t, *J* = 6.2 Hz, 2H), 2.58 (t, *J* = 10.0, 2H), 2.74 (t, *J* = 5.0 Hz, 2H), 1.74 (dq, *J* = 10.7, 6.1, 5.6 Hz, 2H), 1.58 (dp, *J* = 25.7, 7.0 Hz, 4H), 1.46 (q, *J* = 6.3 Hz, 2H); **^{13}C NMR** (126 MHz, CDCl$_3$) δ 167.8, 137.0, 132.9, 131.6, 130.6, 129.7, 125.7, 65.6, 36.5, 31.7, 30.4, 27.6, 27.3, 25.9, 23.6; **IR** (KBr, cm^{-1}) 3176, 2925, 1699, 1657, 1586, 1392, 1278, 1116, 745; **Mp**: 75.7 – 78.0 °C; **HRMS** (ESI) calcd. For $C_{15}H_{20}NaO_2S_2^+$ [M+Na]$^+$ m/z 319.0797, found: 319.0793.

1,4,5,7,8,10,11,14-Octahydro-3*H*,12*H*benzo[*n*][1,12]dioxa[5,8]dithiacyclohexadecine-3,12-dione (34)

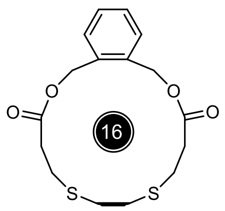

Prepared following the general procedure outlined above using [Ir[d(*t*Bu)(CF$_3$)ppy]$_2$(dtbbpy)[Cl] (2.7 mg, 2.5 μmol, 0.01 equiv.), 1,2-phenylenebis(methylene) bis(3-mercaptopropanoate) (79 mg, 0.25 mmol, 1 equiv.), DMF (5 mL), *N,N*-diisopropylethylamine (49 mg, 0.38 mmol, 1.5 equiv.) and deionized water (100 μL). Purification by column chromatography (Eluent: petroleum ether/EtOAc = 15/1) afforded the title compound as a white solid (33 mg, 39% yield).

^{1}H NMR (500 MHz, $CDCl_3$) δ 7.44 – 7.40 (m, 2H), 7.40 – 7.37 (m, 2H), 5.23 (s, 4H), 2.84 (t, *J* = 7.2 Hz, 4H), 2.71 (s, 4H), 2.68 (d, *J* = 7.3 Hz, 4H); **^{13}C NMR** (126 MHz, $CDCl_3$) δ 171.7, 134.6, 131.3, 129.3, 64.6, 35.7, 31.9, 26.9; **IR** (KBr, cm^{-1}) 3131, 2921, 1747, 1397, 1124; **Mp**: 91.2 – 93.5 °C; **HRMS** (ESI) calcd. For $C_{16}H_{20}NaO_4S_2^+$ $[M+Na]^+$ m/z 363.0695, found: 363.0689.

5,6,7,8,9,10,12,13,15,16,17,18,19,20-Tetradecahydrobenzo[*e*][1,4,7,10]tetraoxa[17,20]dithiacyclohexacosine-3,22(2*H*,23*H*)-dione (35)

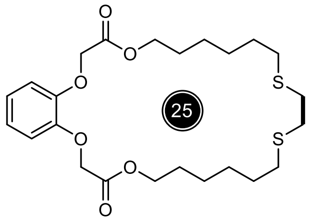

Prepared following the general procedure outlined above using [Ir[d(*t*Bu)(CF_3)ppy]$_2$(dtbbpy)[Cl] (2.7 mg, 2.5 μmol, 0.01 equiv.), bis(6-mercaptohexyl) 2,2'-(1,2-phenylenebis(oxy))diacetate (115 mg, 0.25 mmol, 1 equiv.), DMF (5 mL), *N,N*-diisopropylethylamine (49 mg, 0.38 mmol, 1.5 equiv.) and deionized water (100 μL). Purification by column chromatography (Eluent: petroleum ether/EtOAc = 5/1) afforded the title compound as a white solid (29 mg, 24% yield).

^{1}H NMR (500 MHz, $CDCl_3$) δ 6.99 – 6.94 (m, 2H), 6.93 (m, 2H), 4.74 (s, 4H), 4.21 (t, *J* = 5.9 Hz, 4H), 2.71 (s, 4H), 2.52 (t, *J* = 7.2 Hz, 4H), 1.71 – 1.60 (m, 4H), 1.60 – 1.52 (m, 4H), 1.40 (dd, *J* = 14.3, 7.3 Hz, 4H), 1.35 – 1.24 (m, 4H); **^{13}C NMR** (126 MHz, $CDCl_3$) 169.2, 148.0, 122.6, 115.7, 66.8, 65.1, 32.1, 31.9, 29.5, 28.3, 28.1, 25.3; **IR** (KBr, cm^{-1}) 3448, 3161, 2923, 1635, 1399, 1120; **Mp**: 67.9 – 68.4 °C; **HRMS** (ESI) calcd. For $C_{24}H_{36}NaO_6S_2^+$ $[M+Na]^+$ m/z 507.1846, found: 507.1843.

3,14-Dioxa-7,10-dithia-1(1,3)-benzenacyclopentadecaphane-4,13-dione (36)

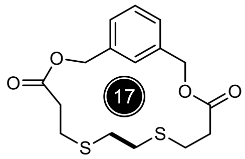

Prepared following the general procedure outlined above using [Ir[d(*t*Bu)(CF_3)ppy]$_2$(dtbbpy)[Cl] (2.7 mg, 2.5 μmol, 0.01 equiv.), 1,3-phenylenebis(methylene) bis(3-mercaptopropanoate) (79 mg, 0.25 mmol, 1 equiv.), DMF (5 mL), *N,N*-diisopropylethylamine (49 mg, 0.38 mmol, 1.5 equiv.) and deionized water (100 μL). Purification by column chromatography (Eluent: petroleum ether/EtOAc = 15/1) afforded the title compound as a white solid (31 mg, 37% yield).

^{1}H NMR (500 MHz, $CDCl_3$) δ 7.52 (s, 1H), 7.33 (m, 1H), 7.25 (d, *J* = 7.9 Hz, 2H), 5.18 (s, 4H), 2.86 (t, *J* = 7.1 Hz, 4H), 2.71 (s, 4H), 2.69 (t, *J* = 7.2 Hz, 4H); **^{13}C NMR** (126 MHz, $CDCl_3$) δ 171.4, 136.5, 128.5, 127.8, 126.9, 66.0, 35.4, 32.1, 27.4; **IR** (KBr, cm^{-1}) 3140, 2923, 1617, 1400, 1120; **Mp**: 66.7 – 71.1 °C; **HRMS** (ESI) calcd. For $C_{16}H_{20}NaO_4S_2^+$ $[M+Na]^+$ m/z 363.0695, found: 363.0687.

3,14-Dioxa-7,10-dithia-1(1,4)-benzenacyclopentadecaphane-4,13-dione (37)

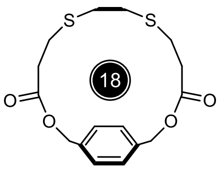

Prepared following the general procedure outlined above using [Ir[d(*t*Bu)(CF_3)ppy]$_2$(dtbbpy)[Cl] (5.4 mg, 5 μmol, 0.01 equiv.), 1,4-phenylenebis(methylene) bis(3-mercaptopropanoate) (158 mg, 0.5 mmol, 1 equiv.), DMF (10 mL), *N,N*-diisopropylethylamine (98 mg, 0.75 mmol, 1.5 equiv.) and deionized water (200 μL). Purification by column chromatography (Eluent: petroleum ether/EtOAc = 10/1) afforded the title compound as a yellowish solid (14 mg, 8% yield).

^{1}H NMR (500 MHz, $CDCl_3$) δ 7.45 (s, 4H), 5.16 (s, 4H), 2.69 (dd, *J* = 7.3, 4.6 Hz, 4H), 2.57 (dd, *J* = 7.2, 4.7 Hz, 4H), 2.07 (s, 4H); **^{13}C NMR** (126 MHz, $CDCl_3$) δ 171.8, 136.4, 130.1, 66.2, 36.0, 32.0, 27.8; **IR** (KBr, cm^{-1}) 3128, 2923, 1747, 1399, 1126; **Mp**: 74.2 – 76.8 °C; **HRMS** (ESI) calcd. For $C_{16}H_{20}NaO_4S_2^+$ [M+Na]$^+$ m/z 363.0695, found: 363.0693.

2,5,16,19-Tetraoxa-9,12-dithia-1(1,4)-benzenacyclononadecaphane-6,15-dione (38)

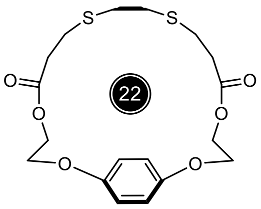

Prepared following the general procedure outlined above using [Ir[d(*t*Bu)(CF_3)ppy]$_2$(dtbbpy)[Cl] (2.7 mg, 2.5 μmol, 0.01 equiv.), (1,4-phenylenebis(oxy))bis(ethane-2,1-diyl) bis(3-mercaptopropanoate) (94 mg, 0.25 mmol, 1 equiv.), DMF (5 mL), *N,N*-diisopropylethylamine (49 mg, 0.38 mmol, 1.5 equiv.) and deionized water (100 μL). Purification by column chromatography (Eluent: petroleum ether/EtOAc = 1/1) afforded the title compound as a yellowish solid (22 mg, 22% yield).

^{1}H NMR (500 MHz, $CDCl_3$) δ 6.99 – 6.94 (m, 2H), 6.93 (m, 2H), 4.74 (s, 4H), 4.21 (t, *J* = 5.9 Hz, 4H), 2.71 (s, 4H), 2.52 (t, *J* = 7.2 Hz, 4H), 1.71 – 1.60 (m, 4H), 1.60 – 1.52 (m, 4H), 1.40 (dd, *J* = 14.3, 7.3 Hz, 4H), 1.35 – 1.24 (m, 4H); **^{13}C NMR** (126 MHz, $CDCl_3$) δ 169.2, 148.0, 122.6, 115.7, 66.8, 65.1, 32.1, 31.9, 29.5, 28.3, 28.1, 25.3; **IR** (KBr, cm^{-1}) 3448, 3161, 2923, 1635, 1399, 1120; **Mp**: 67.9 – 68.4 °C; **HRMS** (ESI) calcd. For $C_{24}H_{36}NaO_6S_2^+$ [M+Na]$^+$ m/z 507.1846, found: 507.1843.

2,6,17,21-Tetraoxa-10,13-dithia-1(2,7)-naphthalenacyclohenicosaphane-7,16-dione (39)

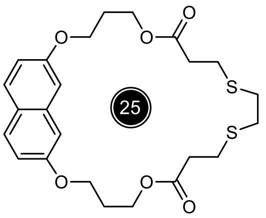

Prepared following the general procedure outlined above using [Ir[d(*t*Bu)(CF_3)ppy]$_2$(dtbbpy)[Cl] (2.7 mg, 2.5 μmol, 0.01 equiv.), (naphthalene-2,7-diylbis(oxy))bis(propane-3,1-diyl) bis(3-mercaptopropanoate) (113 mg, 0.25 mmol, 1 equiv.), DMF (5 mL), *N,N*-diisopropylethylamine (49 mg, 0.38 mmol, 1.5 equiv.) and deionized water (100 μL). Purification by column chromatography

(Eluent: petroleum ether/EtOAc = 8/1) afforded the title compound as a white solid (37 mg, 31% yield).
^{1}H NMR (500 MHz, $CDCl_3$) δ 7.63 (d, *J* = 8.9 Hz, 2H), 7.04 (d, *J* = 1.6 Hz, 2H), 6.97 (dd, *J* = 8.8, 2.0 Hz, 2H), 4.36 (t, *J* = 5.0 Hz, 4H), 4.17 (t, *J* = 6.5 Hz, 4H), 2.76 (t, *J* = 7.0 Hz, 4H), 2.62 (s, 4H), 2.59 (t, *J* = 6.9 Hz, 4H), 2.24 – 2.15 (m, 4H); **^{13}C NMR** (126 MHz, $CDCl_3$) δ 171.5, 157.6, 135.9, 129.1, 124.3, 116.7, 105.7, 64.3, 61.2, 34.8, 31.9, 28.5, 27.5; **IR** (KBr, cm^{-1}) 3135, 2922, 1737, 1515, 1457, 1396, 1125, 1047, 830; **Mp**: 103.7 – 105.6 °C; **HRMS** (ESI) calcd. For $C_{24}H_{30}O_6S_2^+$ $[M+H]^+$ m/z 479.1557, found: 479.1547.

3,14-Dioxa-7,10-dithia-1(2,6)-pyridinacyclopentadecaphane-4,13-dione (40)

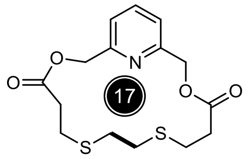

Prepared following the general procedure outlined above using [Ir[d(*t*Bu)(CF_3)ppy]$_2$(dtbbpy)[Cl] (2.7 mg, 2.5 μmol, 0.01 equiv.), pyridine-2,6-diylbis(methylene) bis(3-mercaptopropanoate) (79 mg, 0.25 mmol, 1 equiv.), DMF (5 mL), *N,N*-diisopropylethylamine (49 mg, 0.38 mmol, 1.5 equiv.) and deionized water (100 μL). Purification by column chromatography (Eluent: petroleum ether/EtOAc/TEA = 1/1/0.001) afforded the title compound as a white solid (27 mg, 32% yield).
^{1}H NMR (500 MHz, $CDCl_3$) δ 7.63 (t, *J* = 7.7 Hz, 1H), 7.23 – 7.19 (m, 2H), 5.15 (s, 4H), 2.76 (t, *J* = 7.5 Hz, 4H), 2.63 (t, *J* = 7.6 Hz, 4H), 2.58 (s, 4H); **^{13}C NMR** (126 MHz, $CDCl_3$) δ 171.4, 155.1, 137.4, 122.6, 66.6, 35.2, 31.6, 26.9; **IR** (KBr, cm^{-1}) 3164, 2954, 1734, 1637, 1399, 1139; **Mp**: 70.8 – 72.8 °C; **HRMS** (ESI) calcd. For $C_{15}H_{20}NO_4S_2^+$ $[M+H]^+$ m/z 342.0828, found: 342.0829.

3,14-Dioxa-7,10-dithia-1(2,5)-furanacyclopentadecaphane-4,13-dione (41)

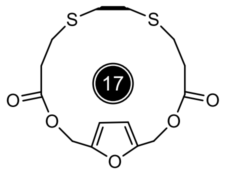

Prepared following the general procedure outlined above using [Ir[d(*t*Bu)(CF_3)ppy]$_2$(dtbbpy)[Cl] (2.7 mg, 2.5 μmol, 0.01 equiv.), furan-2,5-diylbis(methylene) bis(3-mercaptopropanoate) (76 mg, 0.25 mmol, 1 equiv.), DMF (5 mL), *N,N*-diisopropylethylamine (49 mg, 0.38 mmol, 1.5 equiv.) and deionized water (100 μL). Purification by column chromatography (Eluent: petroleum ether/EtOAc = 6/1) afforded the title compound as a yellowish solid (12 mg, 15% yield).
^{1}H NMR (500 MHz, $CDCl_3$) δ 6.31 (s, 2H), 5.03 (s, 4H), 2.74 (t, *J* = 7.0 Hz, 4H), 2.61 – 2.53 (m, 8H); **^{13}C NMR** (126 MHz, $CDCl_3$) δ 171.1, 150.1, 111.6, 58.0, 35.7, 32.5, 27.6; **IR** (KBr, cm^{-1}) 3165, 2967, 1734, 1582, 1383, 1119; **Mp**: 84.0 – 85.1 °C; **HRMS** (ESI) calcd. For $C_{14}H_{18}NaO_5S_2^+$ $[M+Na]^+$ m/z 353.0488, found: 353.0482.

3,20-Dioxa-10,13-dithiatricyclo phthalate-2,21-dione (42)

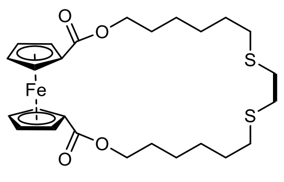

Prepared following the general procedure outlined above using [Ir[d(*t*Bu)(CF$_3$)ppy]$_2$(dtbbpy)[Cl] (2.7 mg, 2.5 μmol, 0.01 equiv.), bis(6-mercaptohexyl) 1,1′-ferrocenedicarboxylate (127 mg, 0.25 mmol, 1 equiv.), DMF (5 mL), *N,N*-diisopropylethylamine (49 mg, 0.38 mmol, 1.5 equiv.) and deionized water (100 μL). Purification by column chromatography (Eluent: petroleum ether/EtOAc = 8/1) afforded the title compound as a red liquid (11 mg, 8% yield).
^{1}H NMR (500 MHz, CDCl$_3$) δ 4.77 (s, 4H), 4.43 (s, 4H), 4.26 (t, J = 6.5 Hz, 4H), 2.75 (t, *J* = 7.2 Hz, 4H), 1.79 (q, *J* = 6.7 Hz, 8H), 1.57 (m, 4H), 1.56 – 1.51 (m, 8H); **^{13}C NMR** (126 MHz, CDCl$_3$) δ 170.5, 73.2, 72.6, 71.6, 64.5, 39.5, 29.1, 28.6, 28.0, 25.7; **IR** (KBr, cm^{-1}) 3314, 2906, 1736, 1560, 1460, 1278, 1198, 920; **HRMS** (ESI) calcd. For $C_{26}H_{37}FeO_4S_2^+$ [M+H]$^+$ m/z 533.1477, found: 533.1479.

9,10,12,13,16,17-Hexahydro-8*H*,15*H*-dinaphtho[2,1-m:1',2'o][1,12]dioxa[5,8]dithiacyclohexadecine (60)

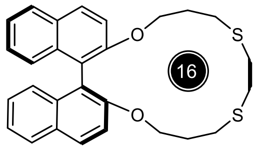

Prepared following the general procedure outlined above using [Ir[d(*t*Bu)(CF$_3$)ppy]$_2$(dtbbpy)[Cl] (2.7 mg, 2.5 μmol, 0.01 equiv.), (*R*)-(+)-3,3'-([1,1'-binaphthalene]-2,2'-diylbis(oxy))bis(propane-1-thiol) (109 mg, 0.25 mmol, 1 equiv.), DMF (5 mL), *N,N*-diisopropylethylamine (49 mg, 0.38 mmol, 1.5 equiv.) and deionized water (100 μL). Purification by column chromatography (Eluent: petroleum ether/EtOAc = 20/1) afforded the title compound as a colourless oil (43 mg, 40% yield).
^{1}H NMR (500 MHz, CDCl$_3$) δ 7.93 (d, *J* = 9.0 Hz, 2H), 7.83 (d, *J* = 8.2 Hz, 2H), 7.42 (d, *J* = 8.8 Hz, 2H), 7.29 (t, *J* = 7.4 Hz, 2H), 7.17 (t, *J* = 7.6 Hz, 2H), 7.11 (d, *J* = 8.5 Hz, 2H), 4.45 – 4.24 (m, 4H), 3.81 (dt, *J* = 9.8, 4.9 Hz, 4H), 2.72 – 2.42 (m, 4H), 2.35 – 2.04 (m, 4H), 1.61 (q, *J* = 5.1 Hz, 4H); **^{13}C NMR** (126 MHz, CDCl$_3$) δ 154.1, 134.2, 132.4, 132.3, 129.4, 129.4, 128.6, 128.5, 128.0, 126.3, 125.4, 123.7, 120.6, 115.6, 66.8, 32.6, 30.7, 27.2; **IR** (KBr, cm^{-1}) 3308, 2823, 1735, 1560, 1440, 1239, 917; **HRMS** (ESI) calcd. For $C_{28}H_{28}O_2S_2^+$ [M+H]$^+$ m/z 461.1603, found: 461.1596.

12,13,14,15,16,17,19,20,22,23,24,25,26,27-Tetradecahydrodinaphtho[2,1-b:1',2'-d][1,6]dioxa[13,16]dithiacyclodocosine (61)

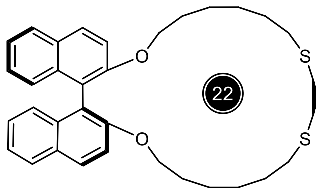

Prepared following the general procedure outlined above using [Ir[d(*t*Bu)(CF$_3$)ppy]$_2$(dtbbpy)[Cl] (2.7 mg, 2.5 μmol, 0.01 equiv.), (*R*)-(+)-6,6'-([1,1'-binaphthalene]-2,2'-diylbis(oxy))bis(hexane-1-thiol) (130 mg, 0.25 mmol, 1 equiv.), DMF (5 mL), *N,N*-diisopropylethylamine (49 mg, 0.38 mmol, 1.5 equiv.) and deionized water (100 μL). Purification by column chromatography (Eluent: petroleum ether/EtOAc = 20/1) afforded the title compound as a colourless oil (48 mg, 35% yield).
^{1}H NMR (500 MHz, CDCl$_3$) δ 7.91 (d, *J* = 9.0 Hz, 2H), 7.83 (d, *J* = 8.1 Hz, 2H), 7.39 (d, *J* = 9.0 Hz, 2H), 7.29 (t, *J* = 7.4 Hz, 2H), 7.18 (t, *J* = 7.6 Hz, 2H), 7.12 (d, *J* = 8.5 Hz, 2H), 4.00 – 3.90 (m, 2H), 3.86 (dt, *J* = 9.5, 6.2 Hz, 2H), 2.69 (s, 4H), 2.46 (t, *J* = 7.4 Hz, 4H), 1.47 (dp, *J* = 20.4, 6.7 Hz, 8H), 1.26 (ddq, *J* = 27.9, 13.3, 6.8 Hz, 4H), 1.15 (h, *J* = 7.5, 6.5 Hz, 4H); **^{13}C NMR** (126 MHz,

$CDCl_3$) δ 154.7, 134.3, 129.4, 129.2, 127.9, 126.2, 125.5, 123.6, 120.9, 116.3, 69.9, 32.1, 31.8, 29.7, 29.3, 28.1, 25.2; **IR** (KBr, cm^{-1}) 3302, 2873, 1760, 1582, 1560, 1439, 1271, 916; **HRMS** (ESI) calcd. For $C_{34}H_{40}O_2S_2Na^+$ $[M+Na]^+$ m/z 567.2362, found: 567.2367.

9,10,13,14,16,17,19,20,24,25-Decahydro-8H,12H,21H,23H-dinaphtho[2,1-u:1',2'w][1,5,16,20] tetraoxa[9,12]dithiacyclotetracosine-12,21-dione (62)

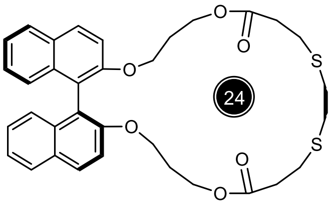

Prepared following the general procedure outlined above using $[Ir[d(tBu)(CF_3)ppy]_2(dtbbpy)[Cl]$ (2.7 mg, 2.5 μmol, 0.01 equiv.), (*R*)-(+)-([1,1'-binaphthalene]-2,2'-diylbis(oxy))bis(propane-3,1-diyl) bis(3-mercaptopropanoate) (130 mg, 0.25 mmol, 1 equiv.), DMF (5 mL), *N,N*-diisopropylethylamine (49 mg, 0.38 mmol, 1.5 equiv.) and deionized water (100 μL). Purification by column chromatography (Eluent: petroleum ether/EtOAc = 20/1) afforded the title compound as a colourless oil (45 mg, 30% yield).

^{1}H NMR (500 MHz, $CDCl_3$) δ 7.94 (d, J = 9.0 Hz, 2H), 7.85 (d, J = 8.2 Hz, 2H), 7.41 (d, J = 9.0 Hz, 2H), 7.31 (t, J = 7.4 Hz, 2H), 7.20 (t, J = 7.6 Hz, 2H), 7.12 (d, J = 8.5 Hz, 2H), 4.11 – 3.99 (m, 4H), 3.87 – 3.82 (m, 2H), 3.81 – 3.75 (m, 2H), 2.84 – 2.75 (m, 4H), 2.74 – 2.66 (m, 4H), 2.63 – 2.52 (m, 4H), 1.85 – 1.75 (m, 2H), 1.75 – 1.65 (m, 2H); **^{13}C NMR** (126 MHz, $CDCl_3$) δ 171.9, 154.0, 134.1, 129.4, 129.4, 127.9, 126.3, 125.4, 123.7, 120.4, 115.4, 65.8, 61.5, 35.5, 32.1, 28.7, 26.8; **IR** (KBr, cm^{-1}) 3302, 2873, 1731, 1583, 1441, 1233, 913; **HRMS** (ESI) calcd. For $C_{34}H_{36}NaO_6S_2^+$ $[M+Na]^+$ m/z 627.1846, found: 627.1839.

8,9,10,11,12,13,14,15,17,18,20,21,22,23,24,25,26,27-Octadecahydrodinaphtho[2,1-b:1',2'-d][1,6]dioxa[15,18]dithiacyclohexacosine (63)

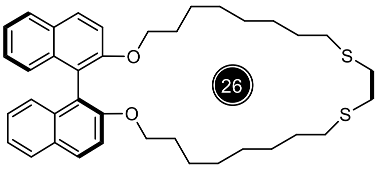

Prepared following the general procedure outlined above using $[Ir[d(tBu)(CF_3)ppy]_2(dtbbpy)[Cl]$ (2.7 mg, 2.5 μmol, 0.01 equiv.), (*R*)-(+)-8,8'-([1,1'-binaphthalene]-2,2'-diylbis(oxy))bis(octane-1-thiol) (144 mg, 0.25 mmol, 1 equiv.), DMF (5 mL), *N,N*-diisopropylethylamine (49 mg, 0.38 mmol, 1.5 equiv.) and deionized water (100 μL). Purification by column chromatography (Eluent: petroleum ether/EtOAc = 20/1) afforded the title compound as a colourless oil (42 mg, 28% yield).

^{1}H NMR (500 MHz, $CDCl_3$) δ 7.83 (d, J = 9.0 Hz, 2H), 7.75 (d, J = 8.2 Hz, 2H), 7.31 (d, J = 9.0 Hz, 2H), 7.20 (t, J = 7.4 Hz, 2H), 7.10 (t, J = 7.6 Hz, 2H), 7.03 (d, J = 8.5 Hz, 2H), 3.94 – 3.69 (m, 4H), 2.63 (d, J = 1.4 Hz, 4H), 2.53 – 2.35 (m, 4H), 1.49 (p, J = 6.9 Hz, 4H), 1.36 (dq, J = 12.5, 6.6 Hz, 4H), 1.25 (p, J = 7.0 Hz, 4H), 1.16 – 0.94 (m, 12H); **^{13}C NMR** (126 MHz, $CDCl_3$) δ 154.6, 134.3, 129.3, 129.1, 127.8, 126.1, 125.5, 123.5, 120.7, 116.0, 69.8, 32.1, 32.0, 29.9, 29.4, 29.0, 29.0, 28.6, 25.7; **IR** (KBr, cm^{-1}) 3286, 2923, 1728, 1587, 1439, 1262, 1087, 807; **HRMS** (ESI) calcd. For $C_{38}H_{48}O_2S_2^+$ $[M+Na]^+$ m/z 623.2988, found: 623.2990.

9,10,11,12,15,16,18,19,21,22,26,27,28,29-Tetradecahydro-8*H*,14*H*,23*H*,25*H*-dinaphtho[2,1-*s*:1',2'-*u*][1,12,18,23]tetraoxa[5,8]dithiacyclooctacosine-14,23-dione (64)

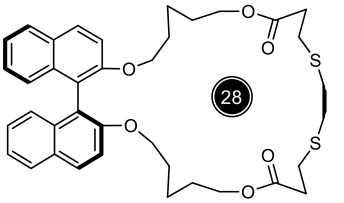

Prepared following the general procedure outlined above using [Ir[d(*t*Bu)(CF_3)ppy]$_2$(dtbbpy)[Cl] (2.7 mg, 2.5 μmol, 0.01 equiv.), (*R*)-(+)-([1,1'-binaphthalene]-2,2'-diylbis(oxy))bis(pentane-5,1-diyl) bis(3-mercaptopropanoate) (159 mg, 0.25 mmol, 1 equiv.), DMF (5 mL), *N,N*-diisopropylethylamine (49 mg, 0.38 mmol, 1.5 equiv.) and deionized water (100 μL). Purification by column chromatography (Eluent: petroleum ether/EtOAc = 4/1) afforded the title compound as a colourless oil (48 mg, 29% yield).

^{1}H NMR (500 MHz, $CDCl_3$) δ 7.85 (d, *J* = 9.0 Hz, 2H), 7.77 (d, *J* = 8.2 Hz, 2H), 7.32 (d, *J* = 9.0 Hz, 2H), 7.27 – 7.19 (m, 2H), 7.11 (td, *J* = 7.3, 6.6, 1.2 Hz, 2H), 7.05 (d, *J* = 8.5 Hz, 2H), 4.01 – 3.66 (m, 8H), 2.73 (t, *J* = 7.4 Hz, 4H), 2.66 (s, 4H), 2.51 (t, *J* = 7.1 Hz, 4H), 1.38 (ddt, *J* = 21.6, 14.0, 6.5 Hz, 4H), 1.33 – 1.22 (m, 4H), 1.11 – 0.93 (m, 4H); **^{13}C NMR** (126 MHz, $CDCl_3$) δ 171.8, 154.4, 134.3, 129.3, 129.2, 127.8, 126.2, 125.5, 123.5, 120.7, 115.8, 69.4, 64.7, 35.3, 32.2, 28.8, 28.0, 27.3, 22.2; **HRMS** (ESI) calcd. For $C_{38}H_{44}NaO_6S_2^+$ [M+Na]$^+$ m/z 683.2472, found: 683.2485.

8,9,10,11,12,13,16,17,19,20,22,23,26,27,28,29,30,31-Octadecahydro-15*H*,24*H*-dinaphtho[2,1-t:1',2'-*v*][1,12,19,24]tetraoxa[5,8]dithiacyclotriacontine-15,24-dione (65)

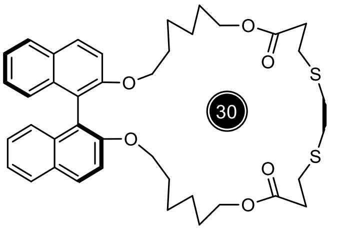

Prepared following the general procedure outlined above using [Ir[d(*t*Bu)(CF_3)ppy]$_2$(dtbbpy)[Cl] (2.7 mg, 2.5 μmol, 0.01 equiv.), (*R*)-(+)-([1,1'-binaphthalene]-2,2'-diylbis(oxy))bis(hexane-6,1-diyl) bis(3-mercaptopropanoate) (166 mg, 0.25 mmol, 1 equiv.), DMF (5 mL), *N,N*-diisopropylethylamine (49 mg, 0.38 mmol, 1.5 equiv.) and deionized water (100 μl). Purification by column chromatography (Eluent: petroleum ether/EtOAc = 4/1) afforded the title compound as a colourless oil (45 mg, 26% yield).

^{1}H NMR (500 MHz, $CDCl_3$) δ 7.92 (d, *J* = 9.0 Hz, 2H), 7.84 (d, *J* = 8.2 Hz, 2H), 7.40 (d, *J* = 9.0 Hz, 2H), 7.29 (t, *J* = 7.3 Hz, 2H), 7.18 (t, *J* = 7.5 Hz, 2H), 7.13 (d, *J* = 8.5 Hz, 2H), 4.02 – 3.94 (m, 6H), 3.91 – 3.85 (m, 2H), 2.81 (t, *J* = 7.1 Hz, 4H), 2.73 (s, 4H), 2.60 (t, *J* = 7.0 Hz, 4H), 1.49 – 1.37 (m, 8H), 1.19 – 1.09 (m, 4H), 1.07 – 0.99 (m, 4H); **^{13}C NMR** (126 MHz, $CDCl_3$) δ 171.9, 154.5, 134.3, 129.3, 129.2, 127.8, 126.1, 125.5, 123.5, 120.8, 115.9, 69.5, 64.7, 35.3, 32.2, 29.2, 28.4, 27.3, 25.4, 25.2; **IR** (KBr, cm^{-1}) 3310, 2934, 2857, 1732, 1586, 1464, 1345, 1263, 1080, 773; **HRMS** (ESI) calcd. For $C_{35}H_{48}NaO_6S_2^+$ [M+Na]$^+$ m/z 677.2941, found: 677.2939.

Dodecahydro-2*H*,11*H*-benzo[*b*][1,4]dioxa[8,11]dithiacyclotetradecine-2,11-dione (58)

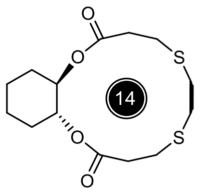

Prepared following the general procedure outlined above using [Ir[d(*t*Bu)(CF_3)ppy]$_2$(dtbbpy)[Cl] (2.7 mg, 2.5 μmol, 0.01 equiv.), (1*R*,2*R*)-cyclohexane-1,2-diyl bis(3-mercaptopropanoate) (73 mg, 0.25 mmol, 1 equiv.), DMF (5 mL), *N*,*N*-diisopropylethylamine (49 mg, 0.38 mmol, 1.5 equiv.) and deionized water (100 μL). Purification by column chromatography (Eluent: petroleum ether/EtOAc = 10/1) afforded the title compound as a white solid (30 mg, 38% yield).

^{1}H NMR (500 MHz, $CDCl_3$) δ 4.96 – 4.81 (m, 2H), 2.97 – 2.87 (m, 4H), 2.84 – 2.77 (m, 2H), 2.75 – 2.60 (m, 6H), 2.08 (d, *J* = 10.8 Hz, 2H), 1.76 (d, *J* = 7.5 Hz, 2H), 1.46 – 1.26 (m, 4H); **^{13}C NMR** (126 MHz, $CDCl_3$) δ 171.6, 74.4, 35.2, 32.4, 30.6, 27.4, 23.7; **IR** (KBr, cm^{-1}) 3130, 2920, 1742, 1399, 1127; **Mp**: 105.6 – 110.9; **HRMS** (ESI) calcd. For $C_{12}H_{20}NaO_4S_2^+$ [M+Na]$^+$ m/z 341.0852, found: 341.0845.

6-Methyl-1-oxa-7,10-dithia-4-azacyclohexadecane-2,5-dione (59)

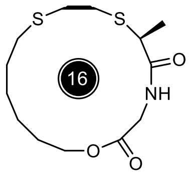

Prepared following the general procedure outlined above using [Ir[d(*t*Bu)(CF_3)ppy]$_2$(dtbbpy)[Cl] (2.7 mg, 2.5 μmol, 0.01 equiv.), 6-mercaptohexyl (*S*)-(2-mercaptopropanoyl)glycinate (70 mg, 0.25 mmol, 1 equiv.), DMF (5 mL), *N*,*N*-diisopropylethylamine (49 mg, 0.38 mmol, 1.5 equiv.) and deionized water (100 μL). Purification by column chromatography (Eluent: petroleum ether/EtOAc = 4/1) afforded the title compound as a yellowish solid (15 mg, 19% yield).

^{1}H NMR (500 MHz, $CDCl_3$) δ 7.23 (d, *J* = 4.8 Hz, 1H), 4.22 (t, *J* = 6.5 Hz, 2H), 4.14 (dd, *J* = 17.7, 6.4 Hz, 1H), 3.86 (dd, *J* = 17.7, 5.0 Hz, 1H), 3.45 (q, *J* = 7.3 Hz, 1H), 2.83 – 2.58 (m, 4H), 2.56 – 2.49 (m, 1H), 2.49 – 2.41 (m, 1H), 1.63 (d, *J* = 5.8 Hz, 2H), 1.55 – 1.49 (m, 2H), 1.48 – 1.31 (m, 7H); **^{13}C NMR** (126 MHz, $CDCl_3$) δ 172.7, 169.5, 64.9, 44.1, 42.2, 31.7, 31.3, 30.6, 28.0, 27.8, 25.7, 23.9, 18.6; **IR** (KBr, cm^{-1}) 2904, 1747, 1506, 1489, 1188, 892; **Mp**: 66.2 – 67.2; **HRMS** (ESI) calcd. For $C_{13}H_{23}NNaO_3S_2^+$ [M+Na]$^+$ m/z 328.1012, found: 328.1011.

1,2,7,8,9,10,11,12,15,16,18,19,21,22,25,26,27,28,29,30,35,36-Docosahydro-14*H*,23*H*-diindeno[7,1-*tu*:1',7'-*vw*][1,12,19,25]tetraoxa[5,8]dithiacyclohentriacontine-14,23-dione (66)

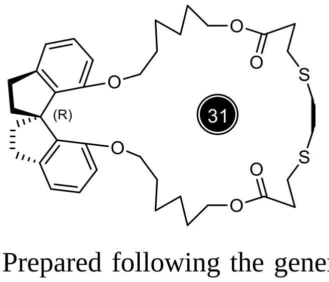

Prepared following the general procedure outlined above using [Ir[d(*t*Bu)(CF_3)ppy]$_2$(dtbbpy)[Cl] (2.7 mg, 2.5 μmol, 0.01 equiv.), (*R*)-(+)-((2,2',3,3'-tetrahydro-1,1'-spirobi[indene]-7,7'-diyl)bis(oxy))bis(hexane-6,1-diyl) bis(3-mercaptopropanoate) (157 mg, 0.25 mmol, 1 equiv.), DMF

(5 mL), *N,N*-diisopropylethylamine (49 mg, 0.38 mmol, 1.5 equiv.) and deionized water (100 μL). Purification by column chromatography (Eluent: petroleum ether/EtOAc = 6/1) afforded the title compound as a yellowish solid (34mg, 21% yield).

^{1}H NMR (500 MHz, $CDCl_3$) δ 7.00 (t, *J* = 7.7 Hz, 2H), 6.73 (d, *J* = 7.4 Hz, 2H), 6.49 (d, *J* = 8.0 Hz, 2H), 4.06 – 3.92 (m, *J* = 6.3 Hz, 4H), 3.70 (dt, *J* = 11.0, 5.7 Hz, 2H), 3.57 (q, *J* = 7.5, 7.1 Hz, 2H), 2.98 – 2.87 (m, 4H), 2.75 (t, *J* = 7.0 Hz, 4H), 2.67 (s, 4H), 2.53 (t, *J* = 7.0 Hz, 4H), 2.33 – 2.20 (m, 2H), 2.07 (dt, *J* = 11.7, 5.4 Hz, 2H), 1.43 (q, *J* = 6.4 Hz, 4H), 1.25 (q, *J* = 13.4 Hz, 4H), 1.09 (dq, *J* = 13.8, 8.0 Hz, 4H), 0.84 (tp, *J* = 13.8, 7.1 Hz, 4H); **^{13}C NMR** (126 MHz, $CDCl_3$) δ 171.9, 155.7, 145.1, 137.2, 127.4, 116.5, 108.7, 66.4, 64.8, 59.3, 38.3, 35.2, 32.1, 31.6, 28.7, 28.4, 27.3, 25.3, 24.7; **IR** (KBr, cm^{-1}) 3310, 2934, 2857, 1732, 1586, 1464, 1345, 1263, 1080, 773; **HRMS** (ESI) calcd. For $C_{35}H_{48}NaO_6S_2^+$ $[M+Na]^+$ m/z 677.2941, found: 677.2939.

Supplementary Table 9. The hydrothiolation of the substituted alkynes enabled by currently protocol.

We sincerely thank the anonymous reviewer´s curiosity in whether other simple alkynes outside of acetylene are amenable in this macrocyclization protocol. Consequently, we subjected substrates ethynylbenzene and hex-1-yne with bis(6-mercaptohexyl) 2,2'-(1,2-phenylenebis(oxy))diacetate to the identified as optimal for the macrocyclization. Unfortunately, the desired macrocyclic products were not detected in both cases, but the substantial amounts of linear vinyl thioesters could be obtained. Those experiments revealed that the diterminal acetylene as linker is important to the current macrocyclization protocol.

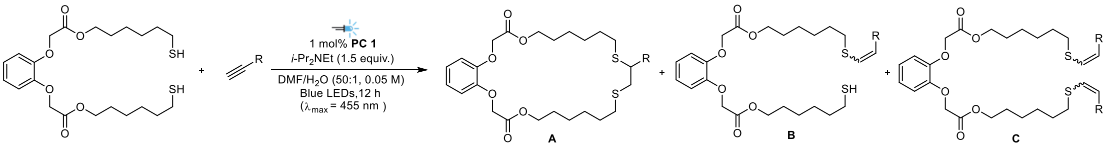

Entry	R =	Alkyne (x equiv.)	A yield (%)[a]	B yield (%)[a]	C yield (%)[a]
1	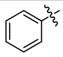	1.0	ND	73	trace
2	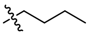	1.0	ND	55	12

[a]Isolated yield is shown in parentheses

6-Mercaptohexyl 2-(2-(2-oxo-2-((6-(styrylthio)hexyl)oxy)ethoxy)phenoxy)acetate (b1)

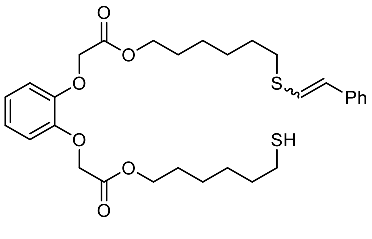

Prepared following the general procedure outlined above using $[Ir(d(tBu)(CF_3)ppy)_2(dtbbpy)][Cl]$ (2.7 mg, 0.0025 mmol, 0.5 mol%), bis(6-mercaptohexyl) 2,2'-(1,2-phenylenebis(oxy))diacetate (115 mg, 0.25 mmol, 1.0 equiv.), ethynylbenzene (26 mg, 0.25 mmol, 1.0 equiv), *N,N*-diisopropylethylamine (48 mg, 0.375 mmol, 1.5 equiv.), deionized water (100 uL), and DMF (5 mL). Purification by column chromatography (Eluent: petroleum ether/EtOAc = 8/1) yielded the pure product as a colourless oil (102 mg, 0.18 mmol, 73% yield).

^{1}H NMR (400 MHz, $CDCl_3$) δ 7.54 – 7.32 (m, 1H), 7.29 (d, J = 4.3 Hz, 3H), 7.18 (dq, J = 9.3, 5.8, 4.5 Hz, 1H), 7.00 – 6.83 (m, 4H), 6.76 – 6.06 (m, 2H), 4.71 (d, J = 2.4 Hz, 4H), 4.18 (td, J = 6.7, 3.5 Hz, 4H), 2.77 (t, J = 7.7 Hz, 2H), 2.49 (q, J = 7.4 Hz, 2H), 1.85 – 1.51 (m, 8H), 1.50 – 1.19 (m, 10H); **^{13}C NMR** (101 MHz, $CDCl_3$) δ 169.1, 148.0, 137.1, 128.7, 128.6, 128.2, 127.6, 126.8, 126.6, 125.5, 125.4, 125.2, 122.5, 115.4, 66.6, 65.1, 35.7, 33.8, 32.6, 32.4, 30.0, 29.3, 28.4, 28.3, 28.1, 28.1, 27.9, 25.4, 25.4, 25.3, 24.6, 24.5; **IR** (KBr, cm^{-1}) 3463, 2820, 1613, 1475, 1360, 1127, 943, 778, 723; **HRMS** (ESI) calcd. For $C_{30}H_{40}O_6S_2Na$ $[M+Na]^+$ m/z 583.2159, found 583.2142.

6-(hex-1-en-1-ylthio)Hexyl 2-(2-(2-((6-mercaptohexyl)oxy)-2-oxoethoxy)phenoxy)acetate (b2)

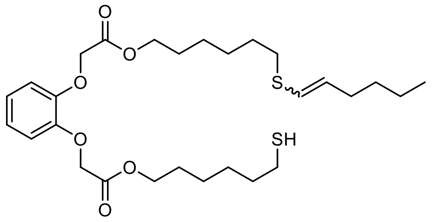

Prepared following the general procedure outlined above using [Ir(d(*t*Bu)(CF_3)ppy)$_2$(dtbbpy)][Cl^-] (2.7 mg, 0.0025 mmol, 0.5 mol%), bis(6-mercaptohexyl) 2,2'-(1,2-phenylenebis(oxy))diacetate (115 mg, 0.25 mmol, 1.0 equiv.), hex-1-yne (21 mg, 0.25 mmol, 1.0 equiv), *N*,*N*-diisopropylethylamine (48 mg, 0.375 mmol, 1.5 equiv.), deionized water (100 uL), and DMF (5 mL). Purification by column chromatography (Eluent: petroleum ether/EtOAc = 8/1) yielded the pure product as a colourless oil (74 mg, 0.13 mmol, 55% yield).

^{1}H NMR (400 MHz, $CDCl_3$) δ 7.11 – 6.76 (m, 4H), 6.09 – 5.77 (m, 1H), 5.56 (d, *J* = 9.4 Hz, 1H), 4.74 (s, 4H), 4.21 (td, *J* = 6.4, 1.8 Hz, 4H), 3.65 (t, *J* = 6.6 Hz, 2H), 2.75 – 2.60 (m, 2H), 2.58 – 2.39 (m, 2H), 1.71 – 1.52 (m, 11H), 1.50 – 1.28 (m, 10H), 1.03 – 0.77 (m, 3H); **^{13}C NMR** (101 MHz, $CDCl_3$) δ 169.2, 169.2, 148.0, 131.0, 129.7, 124.8, 122.7, 122.7, 122.6, 115.9, 115.8, 66.9, 66.8, 65.1, 62.9, 45.8, 38.2, 33.8, 33.6, 32.6, 32.5, 31.2, 30.9, 30.2, 29.7, 29.7, 29.5, 29.0, 28.9, 28.3, 28.3, 28.2, 25.4, 25.3, 25.3, 22.6, 22.3, 14.0, 13.9. **IR** (KBr, cm^{-1}) 3080, 1486, 1223, 1081, 1025, 899, 776; **HRMS** (ESI) calcd. For $C_{28}H_{44}O_6S_2Na$ $[M+Na]^+$ m/z 563.2472, found 563.2477.

Bis(6-(hex-1-en-1-ylthio)hexyl) 2,2'-(1,2-phenylenebis(oxy))diacetate (c1)

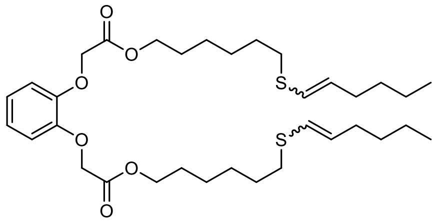

Prepared following the general procedure outlined above using [Ir(d(*t*Bu)(CF_3)ppy)$_2$(dtbbpy)][Cl^-] (2.7 mg, 0.0025 mmol, 0.5 mol%), bis(6-mercaptohexyl) 2,2'-(1,2-phenylenebis(oxy))diacetate (115 mg, 0.25 mmol, 1.0 equiv.), hex-1-yne (21 mg, 0.25 mmol, 1.0 equiv), *N*,*N*-diisopropylethylamine (48 mg, 0.375 mmol, 1.5 equiv.), deionized water (100 uL), and DMF (5 mL). Purification by column chromatography (Eluent: petroleum ether/EtOAc = 8/1) yielded the pure product as a colourless oil (19 mg, 0.03 mmol, 12% yield).

^{1}H NMR (400 MHz, CDCl3) δ 6.98 – 6.93 (m, 2H), 6.93 – 6.86 (m, 2H), 5.98 – 5.81 (m, 2H), 5.71 – 5.44 (m, 2H), 4.73 (s, 4H), 4.20 (t, *J* = 6.6 Hz, 4H), 2.63 (q, *J* = 7.1 Hz, 4H), 2.25 – 1.97 (m, 4H), 1.75 – 1.52 (m, 10H), 1.47 – 1.29 (m, 24H), 0.91 (tdd, *J* = 7.1, 4.2, 2.5 Hz, 8H); **^{13}C NMR** (101

MHz, $CDCl_3$) δ 169.1, 148.1, 131.1, 129.7, 124.8, 122.5, 115.4, 66.6, 65.2, 33.7, 32.9, 32.6, 31.5, 31.2, 30.1, 29.7, 29.3, 28.9, 28.4, 28.3, 28.1, 25.5, 25.4, 22.3, 22.1, 13.9, 13.9, 13.7; **IR** (KBr, cm^{-1}) 3561, 3087, 1615, 1475, 1365, 1130, 941, 776, 726, 669; **HRMS** (ESI) calcd. For $C_{34}H_{54}O_6S_2Na$ $[M+Na]^+$ m/z 645.3254, found 645.3244.

1,4,7,10-Tetrathiacyclododecane (52)

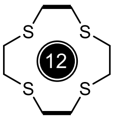

Prepared following the general procedure outlined above using [Ir[d(*t*Bu)(CF_3)ppy]$_2$(dtbbpy)[Cl] (10.8 mg, 10 μmol, 0.01 equiv.), 2,2'-(ethane-1,2-diylbis(oxy))bis(ethane-1-thiol) (93 mg, 1.0 mmol, 1 equiv.), DMF (20 mL), *N,N*-diisopropylethylamine (194 mg, 1.5 mmol, 1.5 equiv.) and deionized water (400 μL). The resulting in a yellow precipitate mixture was filtered through a small pad of silica gel, washed with EtOAc (10 mL), and evaporated. Purification by column chromatography (Eluent: petroleum ether/EtOAc = 120/1) afforded the title compound as a white solid (30 mg, 25% yield).

^{1}H NMR (400 MHz, $CDCl_3$) δ 2.66 (s, 16H); **^{13}C NMR** (101 MHz, $CDCl_3$) δ 28.8; **IR** (KBr, cm^{-1}) 740, 814, 1372, 1514, 2831, 2947, 3178, 3650, 3757, 3831; **Mp**: 222.7 – 224.5 °C; **HRMS** (ESI) calcd. For $C_8H_{16}S_4Na$ $[M+Na]^+$ m/z 263.0027, found 263.0022.

1,4,9,12-Tetrathiacyclohexadecane-6,7,14,15-tetraol (53)

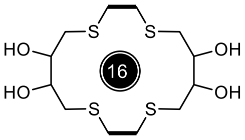

Prepared following the general procedure outlined above using [Ir[d(*t*Bu)(CF_3)ppy]$_2$(dtbbpy)[Cl] (5.4 mg, 5.0 μmol, 0.01 equiv.), 1,4-dimercaptobutane-2,3-diol (91 mg, 0.5 mmol, 1 equiv.), DMF (10 mL), *N,N*-diisopropylethylamine (97 mg, 0.75 mmol, 1.5 equiv.) and deionized water (200 μL). The resulting in a yellow precipitate mixture was filtered through a small pad of silica gel, washed with CH_3OH (10 mL), and evaporated. Purification by column chromatography (Eluent: DCM/CH_3OH = 25/1) afforded the title compound as a white solid (9 mg, 10% yield).

^{1}H NMR (500 MHz, Methanol-d_4) δ 3.77 – 3.69 (m, 4H), 3.21 (s, 2H), 3.18 (d, J = 1.2 Hz, 2H), 2.87 (s, 2H), 2.84 (d, J = 7.4 Hz, 2H), 2.81 (s, 8H).; **^{13}C NMR** (126 MHz, Methanol-d_4) δ 31.9, 31.7, 31.5, 30.2, 29.5; **IR** (KBr, cm^{-1}) 3306, 2927, 2860, 1100, 1055, 743.

1,4,8,11-Tetrathiacyclotetradecane (54)

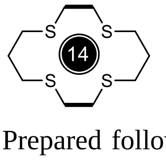

Prepared following the general procedure outlined above using [Ir[d(*t*Bu)(CF_3)ppy]$_2$(dtbbpy)[Cl] (10.8 mg, 10 μmol, 0.01 equiv.), propane-1,3-dithiol (108 mg, 1.0 mmol, 1 equiv.), DMF (20 mL), *N,N*-diisopropylethylamine (194 mg, 1.5 mmol, 1.5 equiv.) and deionized water (400 μL). The resulting in a yellow precipitate mixture was filtered through a small pad of silica gel, washed with EtOAc (10 mL), and evaporated. Purification by column chromatography (Eluent: petroleum ether/EtOAc = 120/1) afforded the title compound as a white solid (44 mg, 33% yield).

^{1}H NMR (400 MHz, $CDCl_3$) δ 2.71 (s, 8H), 2.60 (t, J = 7.3 Hz, 8H), 1.95 – 1.71 (m, 4H); **^{13}C NMR**

(101 MHz, $CDCl_3$) δ 31.6, 30.3, 30.1; **IR** (KBr, cm^{-1}) 2922, 1577, 1028, 743; **Mp**: 115.7 – 117.7 °C; **HRMS** (ESI) calcd. For $C_{10}H_{20}S_4Na$ $[M+Na]^+$ m/z 291.0340, found 291.0336.

1,4,9,12-Tetrathiacyclohexadecane (55)

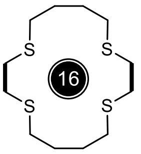

Prepared following the general procedure outlined above using [Ir[d(*t*Bu)(CF_3)ppy]$_2$(dtbbpy)[Cl] (10.8 mg, 10 μmol, 0.01 equiv.), butane-1,4-dithiol (122 mg, 1.0 mmol, 1 equiv.), DMF (20 mL), *N,N*-diisopropylethylamine (194 mg, 1.5 mmol, 1.5 equiv.) and deionized water (400 μL). The resulting in a yellow precipitate mixture was filtered through a small pad of silica gel, washed with EtOAc (10 mL), and evaporated. Purification by column chromatography (Eluent: petroleum ether/EtOAc = 120/1) afforded the title compound as a white solid (44 mg, 30% yield).

^{1}H NMR (400 MHz, $CDCl_3$) δ 2.77 (s, 8H), 2.62 (t, J = 6.3 Hz, 8H), 1.76 (t, J = 6.4 Hz, 8H); **^{13}C NMR** (101 MHz, $CDCl_3$) δ 30.7, 30.2, 27.3; **IR** (KBr, cm^{-1}) 2939, 1654, 1308, 688; **Mp**: 73.5 – 74.8 °C; **HRMS** (ESI) calcd. For $C_{12}H_{24}S_4Na$ $[M+Na]^+$ m/z 319.0653, found 319.0648.

1,4,10,13-Tetrathiacyclooctadecane (56)

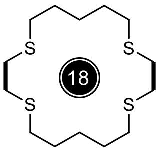

Prepared following the general procedure outlined above using [Ir[d(*t*Bu)(CF_3)ppy]$_2$(dtbbpy)[Cl] (5.4 mg, 5.0 μmol, 0.01 equiv.), pentane-1,5-dithiol (70 mg, 0.5 mmol, 1 equiv.), DMF (10 mL), *N,N*-diisopropylethylamine (97 mg, 0.75 mmol, 1.5 equiv.) and deionized water (200 μL). The resulting in a yellow precipitate mixture was filtered through a small pad of silica gel, washed with EtOAc (10 mL), and evaporated. Purification by column chromatography (Eluent: petroleum ether/EtOAc = 120/1) afforded the title compound as a white solid (20 mg, 25% yield).

^{1}H NMR (400 MHz, $CDCl_3$) δ 2.73 (s, 8H), 2.67 – 2.44 (m, 8H), 1.73 – 1.60 (m, 8H), 1.54 (dt, J = 13.8, 7.0 Hz, 4H); **^{13}C NMR** (101 MHz, $CDCl_3$) δ 77.4, 77.1, 76.8, 31.7, 31.5, 29.7, 29.4, 27.8, 1.0; **IR** (KBr, cm^{-1}) 2924, 1461, 1096, 801; **Mp**: 73.2 – 74.8°C; **HRMS** (ESI) calcd. For $C_{14}H_{28}S_4Na$ $[M+Na]^+$ m/z 347.0966, found 347.0960.

1,4,11,14-Tetrathiacycloicosane (57)

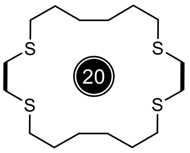

Prepared following the general procedure outlined above using [Ir[d(*t*Bu)(CF_3)ppy]$_2$(dtbbpy)[Cl] (5.4 mg, 5.0 μmol, 0.01 equiv.), hexane-1,6-dithiol (75 mg, 0.5 mmol, 1 equiv.), DMF (10 mL), *N,N*-diisopropylethylamine (97 mg, 0.75 mmol, 1.5 equiv.) and deionized water (200 μL). The resulting in a yellow precipitate mixture was filtered through a small pad of silica gel, washed with EtOAc (10 mL), and evaporated. Purification by column chromatography (Eluent: petroleum ether/EtOAc = 120/1) afforded the title compound as a white solid (20 mg, 23% yield).

^{1}H NMR (400 MHz, $CDCl_3$) δ 2.66 (s, 8H), 2.50 (t, J = 7.3 Hz, 8H), 1.62 – 1.49 (m, 8H), 1.45 –

1.34 (m, 8H); **^{13}C NMR** (101 MHz, $CDCl_3$) δ 30.9, 30.8, 28.5, 27.1; **IR** (KBr, cm^{-1}) 1412, 1186, 691; **Mp**: 73.2 – 75.6 °C; **HRMS** (ESI) calcd. For $C_{16}H_{32}S_4Na$ $[M+Na]^+$ m/z 375.1279, found 375.1274.

1,4,13,16-Tetrathiacyclotetracosane (58)

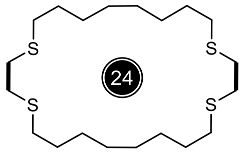

Prepared following the general procedure outlined above using [Ir[d(*t*Bu)(CF_3)ppy]$_2$(dtbbpy)[Cl] (5.4 mg, 5.0 μmol, 0.01 equiv.), octane-1,8-dithiol (89 mg, 0.5 mmol, 1 equiv.), DMF (10 mL), *N,N*-diisopropylethylamine (97 mg, 0.75 mmol, 1.5 equiv.) and deionized water (200 μL). The resulting in a yellow precipitate mixture was filtered through a small pad of silica gel, washed with EtOAc (10 mL), and evaporated. Purification by column chromatography (Eluent: petroleum ether/EtOAc = 120/1) afforded the title compound as a white solid (19 mg, 19% yield).
^{1}H NMR (400 MHz, $CDCl_3$) δ 2.74 (s, 8H), 2.57 (t, J = 7.4 Hz, 8H), 1.71 – 1.53 (m, 8H), 1.47 – 1.38 (m, 8H), 1.35 (s, 8H); **^{13}C NMR** (101 MHz, $CDCl_3$) δ 30.9, 28.6, 27.9, 27.6; **IR** (KBr, cm^{-1}) 3429, 2962, 862; **Mp**: 64.5 – 65.7 °C; **HRMS** (ESI) calcd. For $C_{20}H_{40}S_4Na$ $[M+Na]^+$ m/z 431.1905, found 431.1921.

1,4,14,17-Tetrathiacyclohexacosane (59)

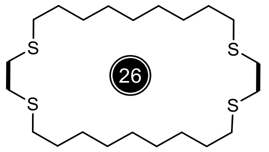

Prepared following the general procedure outlined above using [Ir[d(*t*Bu)(CF_3)ppy]$_2$(dtbbpy)[Cl] (5.4 mg, 5.0 μmol, 0.01 equiv.), nonane-1,9-dithiol (96 mg, 0.5 mmol, 1 equiv.), DMF (10 mL), *N,N*-diisopropylethylamine (97 mg, 0.75 mmol, 1.5 equiv.) and deionized water (200 μL). The resulting in a yellow precipitate mixture was filtered through a small pad of silica gel, washed with EtOAc (10 mL), and evaporated. Purification by column chromatography (Eluent: petroleum ether/EtOAc = 120/1) afforded the title compound as a white solid (26 mg, 24% yield).
^{1}H NMR (400 MHz, $CDCl_3$) δ 2.72 (s, 8H), 2.62 – 2.44 (m, 8H), 1.66 – 1.53 (m, 8H), 1.49 – 1.36 (m, 10H), 1.32 (s, 10H); **^{13}C NMR** (101 MHz, $CDCl_3$) δ 31.9, 29.7, 29.2, 29.0, 28.7; **IR** (KBr, cm^{-1}) 699, 802, 1022, 1095., 2852, 2925; **Mp**: 76.6 – 77.3 °C; **HRMS** (ESI) calcd. For $C_{22}H_{44}S_4Na$ $[M+Na]^+$ m/z 459.2218, found 459.2215.

1,4,15,18-Tetrathiacyclooctacosane (60)

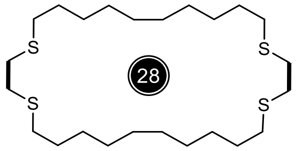

Prepared following the general procedure outlined above using [Ir[d(*t*Bu)(CF_3)ppy]$_2$(dtbbpy)[Cl] (5.4 mg, 5.0 μmol, 0.01 equiv.), decane-1,10-dithiol (103 mg, 0.5 mmol, 1 equiv.), DMF (10 mL), *N,N*-diisopropylethylamine (97 mg, 0.75 mmol, 1.5 equiv.) and deionized water (200 μL). The resulting in a yellow precipitate mixture was filtered through a small pad of silica gel, washed with EtOAc (10 mL), and evaporated. Purification by column chromatography (Eluent: petroleum

ether/EtOAc = 120/1) afforded the title compound as a white solid (21 mg, 18% yield).
^{1}H NMR (400 MHz, $CDCl_3$) δ 2.65 (s, 8H), 2.48 (t, J = 7.3 Hz, 8H), 1.67 – 1.45 (m, 8H), 1.35 (s, 8H), 1.24 (s, 16H); **^{13}C NMR** (101 MHz, $CDCl_3$) δ 31.1, 28.7, 28.2, 28.0, 27.7; **IR** (KBr, cm^{-1}) 2919, 2849, 1461, 1095, 1024, 802; **Mp**: 74.1 – 75.6 °C; **HR-MS** (ESI) calcd. For $C_{24}H_{48}S_4Na$ $[M+Na]^+$ m/z 487.2531, found 487.2525.

1,4,13,16-Tetraoxa-7,10,19,22-tetrathiacyclotetracosane (61)

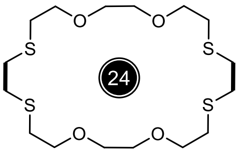

Prepared following the general procedure outlined above using [Ir[d(*t*Bu)(CF_3)ppy]$_2$(dtbbpy)[Cl] (5.4 mg, 5.0 μmol, 0.01 equiv.), 2,2'-(ethane-1,2-diylbis(oxy))bis(ethane-1-thiol) (91 mg, 0.5 mmol, 1 equiv.), DMF (10 mL), *N,N*-diisopropylethylamine (97 mg, 0.75 mmol, 1.5 equiv.) and deionized water (200 μL). Purification by column chromatography (Eluent: petroleum ether/EtOAc = 50/1) afforded the title compound as a yellowish oil (17 mg, 16% yield).
^{1}H NMR (400 MHz, $CDCl_3$) δ 3.70 (t, J = 6.3 Hz, 8H), 3.63 (s, 8H), 2.85 (s, 8H), 2.76 (t, J = 6.3 Hz, 8H); **^{13}C NMR** (101 MHz, $CDCl_3$) δ 71.7, 70.6, 32.8, 31.7; **IR** (KBr, cm^{-1}) 2911, 2853, 1062; **HRMS** (ESI) calcd. For $C_{16}H_{32}O_4S_4Na$ $[M+Na]^+$ m/z 439.1076, found 439.1073.

1,4-Tioxa-7,10-dithiacyclododecane (62)

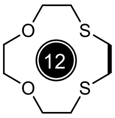

Prepared following the general procedure outlined above using [Ir[d(*t*Bu)(CF_3)ppy]$_2$(dtbbpy)[Cl] (5.4 mg, 5.0 μmol, 0.01 equiv.), 2,2'-(ethane-1,2-diylbis(oxy))bis(ethane-1-thiol) (91 mg, 0.5 mmol, 1 equiv.), DMF (10 mL), *N,N*-diisopropylethylamine (97 mg, 0.75 mmol, 1.5 equiv.) and deionized water (200 μL). Purification by column chromatography (Eluent: petroleum ether/EtOAc = 50/1) afforded the title compound as a yellowish oil (44 mg, 42% yield).
^{1}H NMR (400 MHz, $CDCl_3$) δ 3.79 – 3.70 (m, 4H), 3.54 (s, 4H), 2.93 (s, 4H), 2.70 – 2.53 (m, 4H); **^{13}C NMR** (101 MHz, $CDCl_3$) δ 74.2, 70.4, 33.0, 31.0; **IR** (KBr, cm^{-1}) 1867, 1122, 740; **HR-MS** (ESI) calcd. For $C_8H_{16}O_2S_2Na$ $[M+Na]^+$ m/z 231.0484, found 231.0479.

1,10-Dioxa-4,7,13,16-tetrathiacyclooctadecane (63)

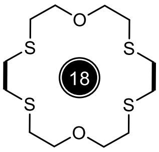

Prepared following the general procedure outlined above using [Ir[d(*t*Bu)(CF_3)ppy]$_2$(dtbbpy)[Cl] (5.4 mg, 5.0 μmol, 0.01 equiv.), 2,2'-oxybis(ethane-1-thiol) (70 mg, 0.5 mmol, 1 equiv.), DMF (10 mL), *N,N*-diisopropylethylamine (97 mg, 0.75 mmol, 1.5 equiv.) and deionized water (200 μL). The resulting in a yellow precipitate mixture was filtered through a small pad of silica gel, washed with EtOAc (10 mL), and evaporated. Purification by column chromatography (Eluent: petroleum ether/EtOAc = 80/1) afforded the title compound as a colourless solid (10 mg, 12% yield).
^{1}H NMR (500 MHz, $CDCl_3$) δ 3.69 (t, J = 6.3 Hz, 8H), 2.87 (s, 8H), 2.75 (t, J = 6.3 Hz, 8H); **^{13}C NMR** (126 MHz, $CDCl_3$) δ 72.0, 32.5, 31.3; **IR** (KBr, cm^{-1}) 3119, 2919, 1944, 1745, 1395, 1111,

1010; **Mp**: 117.7 – 119.9; **HRMS** (ESI) calcd. For $C_{12}H_{24}NaO_2S_4^+$ $[M+Na]^+$ m/z 351.0551, found: 351.0545.

1,4,7-Oxadithionane (64)

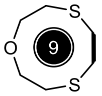

Prepared following the general procedure outlined above using [Ir[d(*t*Bu)(CF_3)ppy]$_2$(dtbbpy)[Cl] (5.4 mg, 5.0 μmol, 0.01 equiv.), 2,2'-oxybis(ethane-1-thiol) (70 mg, 0.5 mmol, 1 equiv.), DMF (10 mL), *N,N*-diisopropylethylamine (97 mg, 0.75 mmol, 1.5 equiv.) and deionized water (200 μL). The resulting in a yellow precipitate mixture was filtered through a small pad of silica gel, washed with EtOAc (10 mL), and evaporated. Purification by column chromatography (Eluent: petroleum ether/EtOAc = 80/1) afforded the title compound as a yellowish oil (18 mg, 23% yield).

^{1}H NMR (500 MHz, $CDCl_3$) δ 4.01 – 3.87 (m, 4H), 3.07 (s, 4H), 2.85 – 2.75 (m, 4H); **^{13}C NMR** (126 MHz, $CDCl_3$) δ 74.0, 35.0, 33.5; **IR** (KBr, cm^{-1}) 2896, 1747, 1506, 1488, 1338, 1266; **HRMS** (ESI) calcd. For $C_6H_{12}NaOS_2^+$ $[M+Na]^+$ m/z 187.0222, found: 187.0230.

1,4,7,10,14,17,20,23-Octathiacyclohexacosane (65)

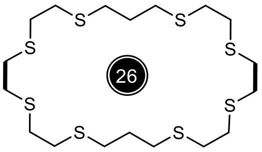

Prepared following the general procedure outlined above using [Ir[d(*t*Bu)(CF_3)ppy]$_2$(dtbbpy)[Cl] (5.4 mg, 5.0 μmol, 0.01 equiv.), 2,2'-(propane-1,3-diylbis(sulfanediyl))bis(ethane-1-thiol) (114 mg, 0.5 mmol, 1 equiv.), DMF (10 mL), *N,N*-diisopropylethylamine (97 mg, 0.75 mmol, 1.5 equiv.) and deionized water (200 μL). The resulting in a yellow precipitate mixture was filtered through a small pad of silica gel, washed with EtOAc (10 mL), and evaporated. Purification by column chromatography (Eluent: petroleum ether/EtOAc = 120/1) afforded the title compound as a white solid (10 mg, 9% yield).

^{1}H NMR (500 MHz, $CDCl_3$) δ 2.83 – 2.74 (m, 24H), 2.70 (t, *J* = 7.0 Hz, 8H), 1.90 (p, *J* = 7.0 Hz, 4H); **^{13}C NMR** (126 MHz, $CDCl_3$) δ 32.5, 32.5, 32.4, 30.9, 29.5; **IR** (KBr, cm^{-1}) 2880, 1734, 1561, 1438, 1349, 901; **Mp**: 90.2 – 91.0; **HRMS** (ESI) calcd. For $C_{18}H_{37}S_8^+$ $[M+H]^+$ m/z 509.0655, found: 509.0650.

1,4,7,10-Tetrathiacyclotridecane (66)

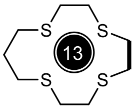

Prepared following the general procedure outlined above using [Ir[d(*t*Bu)(CF_3)ppy]$_2$(dtbbpy)[Cl] (5.4 mg, 5.0 μmol, 0.01 equiv.), 2,2'-(propane-1,3-diylbis(sulfanediyl))bis(ethane-1-thiol) (114 mg, 0.5 mmol, 1 equiv.), DMF (10 mL), *N,N*-diisopropylethylamine (97 mg, 0.75 mmol, 1.5 equiv.) and deionized water (200 μL). The resulting in a yellow precipitate mixture was filtered through a small pad of silica gel, washed with EtOAc (10 mL), and evaporated. Purification by column chromatography (Eluent: petroleum ether/EtOAc = 120/1) afforded the title compound as a white solid (22 mg, 17% yield).

^{1}H NMR (500 MHz, $CDCl_3$) δ 2.81 (s, 4H), 2.80 – 2.73 (m, 8H), 2.65 (t, *J* = 7.5 Hz, 4H), 1.97 –

1.92 (m, 2H); **^{13}C NMR** (126 MHz, $CDCl_3$) δ 31.9, 31.7, 31.5, 30.2, 29.5; **IR** (KBr, cm^{-1}) 2909, 1747, 1506, 1471, 1338; **Mp**: 125.5 – 128.2; **HRMS** (ESI) calcd. For $C_9H_{18}NaS_4^+$ $[M+Na]^+$ m/z 277.0184, found: 277.0178.

1,2-Bis(phenylthio)ethane (67)

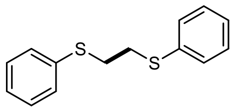

Prepared following the general procedure outlined above using [Ir(d(*t*Bu)(CF_3)ppy)$_2$(dtbbpy)][Cl] (2.7 mg, 0.0025 mmol, 0.5 mol%), benzenethiol (55 mg, 0.5 mmol, 1.0 equiv.), *N,N*-diisopropylethylamine (97 mg, 0.75 mmol, 1.5 equiv.), deionized water (0.25 ml), and DMF (5 mL). Purification by column chromatography (Eluent: petroleum ether/EtOAc = 100/1) yielded the pure product as a light-yellow solid (43 mg, 0.18 mmol, 70% yield).

^{1}H NMR (400 MHz, $CDCl_3$) δ 7.40 – 7.05 (m, 10H), 3.07 (s, 4H); **^{13}C NMR** (101 MHz, $CDCl_3$) δ 135.1, 130.1, 129.1, 126.6, 33.4; **IR** (KBr, cm^{-1}) 3651, 2827, 1747, 1514, 741; **HRMS** (ESI) calcd. for $C_{14}H_{14}S_2Na$ $[M+Na]^+$ *m/z* 269.0429, found 269.0430. The analytical datas are in agreement with the literature.[8]

1,2-Bis(*p*-tolylthio)ethane (68)

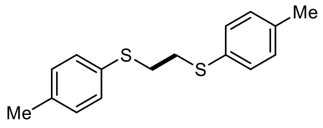

Prepared following the general procedure outlined above using [Ir(d(*t*Bu)(CF_3)ppy)$_2$(dtbbpy)][Cl] (2.7 mg, 0.0025 mmol, 0.5 mol%), 4-methylbenzenethiol (62 mg, 0.5 mmol, 1.0 equiv.), *N,N*-diisopropylethylamine (97 mg, 0.75 mmol, 1.5 equiv.), deionized water (0.25 mL), and DMF (5 mL). Purification by column chromatography (Eluent: petroleum ether/EtOAc = 100/1) yielded the pure product as a white solid (51 mg, 0.19 mmol, 76% yield).

^{1}H NMR (400 MHz, $CDCl_3$) δ 7.25 (d, *J* = 7.3 Hz, 4H), 7.12 (d, *J* = 7.1 Hz, 4H), 3.05 (s, 4H), 2.36 (s, 6H); **^{13}C NMR** (101 MHz, $CDCl_3$) δ 136.8, 131.3, 130.8, 129.8, 34.0, 21.1; **Mp** 74-76 °C; **IR** (KBr, cm^{-1}) 3731, 2825, 1371, 1024, 461, 424; **HRMS** (ESI) calcd. for $C_{16}H_{18}S_2Na$ $[M+Na]^+$ m/z 297.0742, found 297.0745. The analytical datas are in agreement with the literature.[8]

1,2-Bis((4-(*tert*-butyl)phenyl)thio)ethane (69)

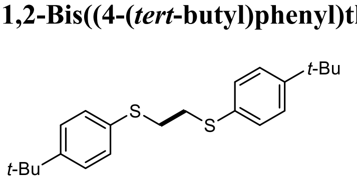

Prepared following the general procedure outlined above using [Ir(d(*t*Bu)(CF_3)ppy)$_2$(dtbbpy)][Cl] (2.7 mg, 0.0025 mmol, 0.5 mol%), 4-(*tert*-butyl)benzenethiol (83 mg, 0.5 mmol, 1.0 equiv.), *N,N*-diisopropylethylamine (97 mg, 0.75 mmol, 1.5 equiv.), deionized water (0.25 mL), and DMF (5 mL). Purification by column chromatography (Eluent: petroleum ether/EtOAc = 100/1) yielded the pure product as a yellow solid (64 mg, 0.18 mmol, 72% yield).

^{1}H NMR (400 MHz, $CDCl_3$) δ 7.34 (d, *J* = 8.5 Hz, 4H), 7.28 (d, *J* = 8.5 Hz, 4H), 3.09 (s, 4H), 1.35 (s, 18H); **^{13}C NMR** (101 MHz, $CDCl_3$) δ 149.9, 131.5, 130.3, 126.1, 34.5, 33.8, 31.3; **Mp**: 104.2 – 105.4 °C; **IR** (KBr, cm^{-1}) 3730, 2893, 2316, 1430, 1371, 1060, 736, 696; **HRMS** (ESI) calcd. for

$C_{22}H_{30}S_2Na$ [M+Na]$^+$ m/z 381.1681, found 381.1675. The analytical datas are in agreement with the literature.[8]

1,2-Bis((4-fluorophenyl)thio)ethane (70)

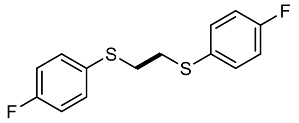

Prepared following the general procedure outlined above using [Ir(d(*t*Bu)(CF_3)ppy)$_2$(dtbbpy)][Cl] (2.7 mg, 0.0025 mmol, 0.5 mol%), 4-fluorobenzenethiol (64 mg, 0.5 mmol, 1.0 equiv.), *N*,*N*-diisopropylethylamine (97 mg, 0.75 mmol, 1.5 equiv.), deionized water (0.25 mL), and DMF (5 mL). Purification by column chromatography (Eluent: petroleum ether/EtOAc = 80/1) yielded the pure product as a white solid (54 mg, 0.19 mmol, 76% yield).
^{1}H NMR (400 MHz, $CDCl_3$) δ 7.30 (t, *J* = 5.1 Hz, 4H), 6.98 (t, *J* = 7.9 Hz, 4H), 2.98 (s, 4H); **^{13}C NMR** (101 MHz, $CDCl_3$) δ 162.1 (d, *J* = 980 Hz, $^1J_{CF}$), 133.2 (d, *J* = 32 Hz, $^3J_{CF}$), 129.8 (d, *J* = 12 Hz, $^3J_{CF}$), 116.2 (d, *J* = 88 Hz, $^2J_{CF}$), 34.6; **^{19}F NMR** (376 MHz, $CDCl_3$) δ -114.51; **IR** (KBr, cm^{-1}) 3728, 3053, 2320, 1401, 1377, 1101, 560; **Mp**: 73.5 – 74.2 °C; **HRMS** (ESI) calcd. for $C_{14}H_{12}F_2S_2Na$ [M+Na]$^+$ m/z 305.0241, found 305.0246. The analytical datas are in agreement with the literature.[9]

1,2-Bis((4-chlorophenyl)thio)ethane (71)

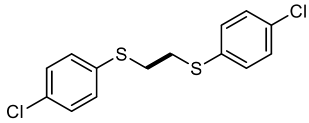

Prepared following the general procedure outlined above using [Ir(d(*t*Bu)(CF_3)ppy)$_2$(dtbbpy)][Cl] (2.7 mg, 0.0025 mmol, 0.5 mol%), 4-chlorobenzenethiol (72 mg, 0.5 mmol, 1.0 equiv.), *N*,*N*-diisopropylethylamine (97 mg, 0.75 mmol, 1.5 equiv.), deionized water (0.25 mL), and DMF (5 mL). Purification by column chromatography (Eluent: petroleum ether/EtOAc = 80/1) yielded the pure product as a colorless solid (55 mg, 0.175 mmol, 70% yield).
^{1}H NMR (400 MHz, CDCl3) δ 7.30 – 7.16 (m, 8H), 3.03 (s, 4H); **^{13}C NMR** (101 MHz, $CDCl_3$) δ 133.4, 132.9, 131.6, 129.2, 33.7; **IR** (KBr, cm^{-1}) 3727, 3208, 2823, 1430, 466; **Mp** 93 – 95 °C; **HRMS** (ESI) calcd. for $C_{14}H_{12}Cl_2S_2Na$ [M+Na]$^+$ m/z 336.9650, found 336.9660. The analytical datas are in agreement with the literature.[8]

1,2-Bis((4-bromophenyl)thio)ethane (72)

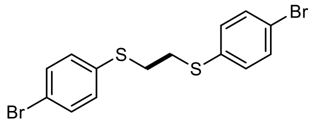

Prepared following the general procedure outlined above using [Ir(d(*t*Bu)(CF_3)ppy)$_2$(dtbbpy)][Cl] (2.7 mg, 0.0025 mmol, 0.5 mol%), 4-bromobenzenethiol (94 mg, 0.5 mmol, 1.0 equiv.), *N*,*N*-diisopropylethylamine (97 mg, 0.75 mmol, 1.5 equiv.), deionized water (0.25 mL), and DMF (5 mL). Purification by column chromatography (Eluent: petroleum ether/EtOAc = 80/1) yielded the pure product as a white solid (67 mg, 0.17 mmol, 67% yield).
^{1}H NMR (400 MHz, $CDCl_3$) δ 7.43 (d, *J* = 8.2 Hz, 4H), 7.19 (d, *J* = 8.2 Hz, 4H), 3.06 (s, 4H); **^{13}C**

NMR (101 MHz, $CDCl_3$) δ 134.1, 132.2, 131.7, 120.7, 33.5; **IR** (KBr, cm^{-1}) 3684, 3152, 1397, 1106, 500, 459; **Mp** 106.6 – 108.2 °C; **HRMS** (ESI) calcd. For $C_{14}H_{12}Br_2S_2Na$ $[M+Na]^+$ m/z 424.8639, found 424.8644.

1,2-Bis((furan-2-ylmethyl)thio)ethane (73)

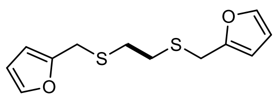

Prepared following the general procedure outlined above using [Ir(d(*t*Bu)(CF_3)ppy)$_2$(dtbbpy)][Cl] (2.7 mg, 0.0025 mmol, 0.5 mol%), furan-2-ylmethanethiol (57 mg, 0.5 mmol, 1.0 equiv.), *N*,*N*-diisopropylethylamine (97 mg, 0.75 mmol, 1.5 equiv.), deionized water (0.25 mL), and DMF (5 mL). Purification by column chromatography (Eluent: petroleum ether/EtOAc = 90/1) yielded the pure product as a clear liquid (34 mg, 0.133 mmol, 53% yield).
^{1}H NMR (400 MHz, $CDCl_3$) δ 7.42 – 7.33 (m, 2H), 6.36 – 6.30 (m, 2H), 6.20 (d, *J* = 3.2 Hz, 2H), 3.75 (s, 4H), 2.69 (s, 4H); **^{13}C NMR** (101 MHz, $CDCl_3$) δ 151.5, 142.2, 110.5, 107.6, 31.4, 28.3; **HRMS** (ESI) calcd. For $C_{14}H_{14}O_2S_2Na$ $[M+Na]^+$ m/z 277.0327, found 277.0342.

1,2-Bis((2-(pyrazin-2-yl)ethyl)thio)ethane (74)

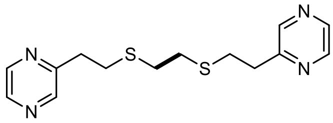

Prepared following the general procedure outlined above using [Ir(d(*t*Bu)(CF_3)ppy)$_2$(dtbbpy)][Cl] (2.7 mg, 0.0025 mmol, 0.5 mol%), 2-(pyrazin-2-yl)ethane-1-thiol (70 mg, 0.5 mmol, 1.0 equiv.), *N*,*N*-diisopropylethylamine (97 mg, 0.75 mmol, 1.5 equiv.), deionized water (0.25 mL), and DMF (5 mL). Purification by column chromatography (Eluent: petroleum ether/EtOAc = 1/1) yielded the pure product as a red-brown liquid (64 mg, 0.21 mmol, 84% yield).
^{1}H NMR (400 MHz, CDCl3) δ 8.50 (d, *J* = 6.2 Hz, 4H), 8.44 (s, 2H), 3.10 (t, *J* = 7.3 Hz, 4H), 2.98 (t, *J* = 7.3 Hz, 4H), 2.73 (s, 4H); **^{13}C NMR** (101 MHz, $CDCl_3$) δ 155.4, 144.8, 144.2, 142.8, 35.5, 32.3, 31.2; **IR** (KBr, cm^{-1}) 3733, 3191, 2321, 1371, 1024, 498, 461; **HRMS** (ESI) calcd. For $C_{14}H_{18}N_4S_2H$ $[M+H]^+$ m/z 307.1046, found 307.1047.

1,2-Bis((4-(4,4,5,5-tetramethyl-1,3,2-dioxaborolan-2-yl)benzyl)thio)ethane (75)

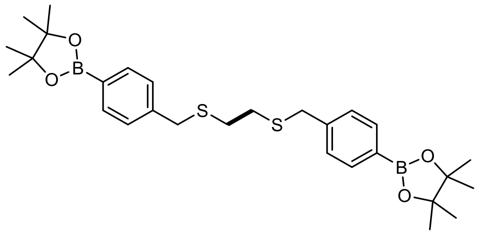

Prepared following the general procedure outlined above using [Ir(d(*t*Bu)(CF_3)ppy)$_2$(dtbbpy)][Cl] (2.7 mg, 0.0025 mmol, 0.5 mol%), (4-(4,4,5,5-tetramethyl-1,3,2-dioxaborolan-2-yl)phenyl)methanethiol (125 mg, 0.5 mmol, 1.0 equiv.), *N*,*N*-diisopropylethylamine (97 mg, 0.75 mmol, 1.5 equiv.), deionized water (0.25 mL), and DMF (5 mL). Purification by column chromatography (Eluent: petroleum ether/EtOAc = 10/1) yielded the pure product as white solid (89 mg, 0.20 mmol, 68% yield).

^{1}**H NMR** (400 MHz, $CDCl_3$) δ 7.80 (d, *J* = 7.8 Hz, 4H), 7.29 (d, *J* = 8.0 Hz, 4H), 3.69 (s, 4H), 2.55 (s, 4H), 1.36 (s, 24H); ^{13}**C NMR** (101 MHz, CDCl3) δ 141.5, 135.1, 135.0, 128.5, 128.4, 128.3, 83.8, 36.4, 31.0, 25.0; **Mp**: 142.5–146.2 °C; **HRMS** (ESI) calcd. For $C_{28}H_{40}B_2O_4S_2Na$ $[M+Na]^+$ m/z 549.2446, found 549.2441.

4,4,13,13-Tetraethoxy-3,14-dioxa-7,10-dithia-4,13-disilahexadecane (76)

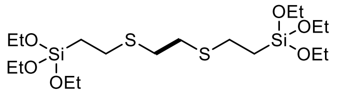

Prepared following the general procedure outlined above using [Ir(d(*t*Bu)(CF_3)ppy)$_2$(dtbbpy)][Cl] (2.7 mg, 0.0025 mmol, 0.5 mol%), 2-(triethoxysilyl)ethane-1-thiol (112 mg, 0.5 mmol, 1.0 equiv.), *N*,*N*-diisopropylethylamine (97 mg, 0.75 mmol, 1.5 equiv.), deionized water (0.25 mL), and DMF (5 mL). Purification by column chromatography (Eluent: petroleum ether/EtOAc = 25/1) yielded the pure product as a clear liquid (19 mg, 0.038 mmol, 15% yield). *Note*: The reaction has been independently three times repeated with similar results. If the reaction time is too long, it will produce an insoluble yellow colloid.
^{1}**H NMR** (500 MHz, $CDCl_3$) δ 3.83 (q, *J* = 7.0 Hz, 12H), 2.72 (s, 4H), 2.63 – 2.52 (m, 4H), 1.24 (t, *J* = 7.0 Hz, 18H), 0.83 – 0.67 (m, 4H); ^{13}**C NMR** (126 MHz, $CDCl_3$) δ 58.4, 35.2, 32.1, 23.3, 18.3, 9.9; **IR** (KBr, cm^{-1}) 3746, 3646, 1681, 1539, 1517; **HRMS** (ESI) calcd. For $C_{18}H_{42}O_6S_2Si_2Na$ $[M+Na]^+$ m/z 497.1854, found 497.1848.

Diethyl 2,2'-(ethane-1,2-diylbis(sulfanediyl))diacetate (77)

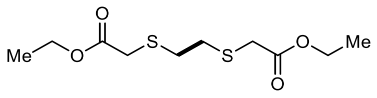

Prepared following the general procedure outlined above using [Ir(d(*t*Bu)(CF_3)ppy)$_2$(dtbbpy)][Cl] (2.7 mg, 0.0025 mmol, 0.5 mol%), ethyl 2-mercaptoacetate (60 mg, 0.5 mmol, 1.0 equiv.), *N*,*N*-diisopropylethylamine (97 mg, 0.75 mmol, 1.5 equiv.), deionized water (0.25 mL), and DMF (5 mL). Purification by column chromatography (Eluent: petroleum ether/EtOAc = 80/1) yielded the pure product as a colorless liquid (55 mg, 0.205 mmol, 82% yield).
^{1}**H NMR** (400 MHz, $CDCl_3$) δ 4.13 (q, *J* = 7.0 Hz, 4H), 3.19 (s, 4H), 2.83 (s, 4H), 1.22 (t, *J* = 7.1 Hz, 6H); ^{13}**C NMR** (101 MHz, $CDCl_3$) δ 170.2, 61.4, 33.4, 31.9, 14.1; **IR** (KBr, cm^{-1}) 3731, 3630, 2939, 2869, 1734, 1263, 729, 501, 460; **HRMS** (ESI) calcd. For $C_{10}H_{18}O_4S_2Na$ $[M+Na]^+$ m/z 289.0544, found 289.0536.

***S*,*S'*-(Ethane-1,2-diyl) diethanethioate (78)**

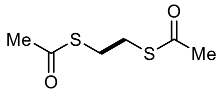

Prepared following the general procedure outlined above using [Ir(d(*t*Bu)(CF_3)ppy)$_2$(dtbbpy)][Cl] (5.4 mg, 0.005 mmol, 0.5 mol%), ethanethioic *S*-acid (76 mg, 1 mmol, 1.0 equiv.), *N*,*N*-diisopropylethylamine (194 mg, 1.5 mmol, 1.5 equiv.), deionized water (0.5 mL), and DMF (10 mL). Purification by column chromatography (Eluent: petroleum ether/EtOAc = 80/1) yielded the pure product as a white solid (70 mg, 0.4 mmol, 79% yield).
^{1}**H NMR** (400 MHz, $CDCl_3$) δ 3.05 (s, 4H), 2.34 (s, 6H); ^{13}**C NMR** (101 MHz, $CDCl_3$) δ 195.1, 30.6, 29.0; **IR** (KBr,cm^{-1}) 3732, 2976, 2826, 2317, 670; **HRMS** (ESI) calcd. For $C_6H_{10}O_4S_2H$

[M+H]$^+$ m/z 179.0195, found 197.0194. The analytical datas are in agreement with the literature.[10]

(Ethane-1,2-diylbis(sulfanediyl))bis(3-methylbutane-3,1-diyl) diformate (79)

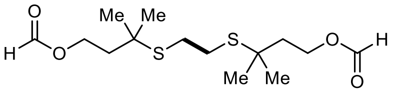

Prepared following the general procedure outlined above using [Ir(d(*t*Bu)(CF$_3$)ppy)$_2$(dtbbpy)][Cl] (2.7 mg, 0.0025 mmol, 0.5 mol%), 3-mercapto-3-methylbutyl formate (74 mg, 0.5 mmol, 1.0 equiv.), *N*,*N*-diisopropylethylamine (97 mg, 0.75 mmol, 1.5 equiv.), deionized water (0.25 mL), and DMF (5 mL). Purification by column chromatography (Eluent: petroleum ether/EtOAc = 25/1) yielded the pure product as a yellow liquid (42 mg, 0.13 mmol, 52% yield).

^{1}H NMR (400 MHz, CDCl$_3$) δ 8.04 (s, 2H), 4.34 (t, *J* = 7.3 Hz, 4H), 2.68 (s, 4H), 1.90 (t, *J* = 7.3 Hz, 4H), 1.34 (s, 12H); ^{13}C NMR (101 MHz, CDCl$_3$) δ 161.0, 61.2, 44.3, 40.1, 29.2, 28.1; **IR** (KBr, cm^{-1}) 3731, 3186, 1655, 1560, 1399, 1063, 619, 501; **HR-MS** (ESI) calcd. For $C_{14}H_{26}O_4S_2Na$ [M+Na]$^+$ m/z 345.1165, found 345.1167.

4,4'-(Ethane-1,2-diylbis(sulfanediyl))bis(4-methylpentan-2-one) (80)

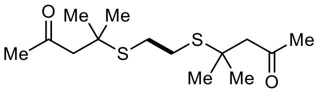

Prepared following the general procedure outlined above using [Ir(d(*t*Bu)(CF$_3$)ppy)$_2$(dtbbpy)][Cl] (2.7 mg, 0.0025 mmol, 0.5 mol%), 4-mercapto-4-methylpentan-2-one (66 mg, 0.5 mmol, 1.0 equiv.), *N*,*N*-diisopropylethylamine (97 mg, 0.75 mmol, 1.5 equiv.), deionized water (0.25 mL), and DMF (5 mL). Purification by column chromatography (Eluent: petroleum ether/EtOAc = 75/1) yielded the pure product as dark-brown liquid (60 mg, 0.205 mmol, 82% yield).

^{1}H NMR (400 MHz, CDCl$_3$) δ 2.69 (s, 4H), 2.66 (s, 4H), 2.14 (s, 6H), 1.39 (s, 12H); **^{13}C NMR** (101 MHz, CDCl$_3$) δ 206.4, 54.6, 44.1, 32.2, 28.5, 28.3; **IR** (KBr, cm^{-1}) 3619, 3176, 1712, 1397, 1108, 572, 501; **HRMS** (ESI) calcd. For $C_{14}H_{26}O_2S_2Na$ [M+Na]$^+$ m/z 313.1266, found 313.1257.

2,2'-(Ethane-1,2-diylbis(sulfanediyl))bis(ethan-1-ol) (81)

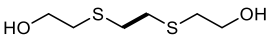

Prepared following the general procedure outlined above using [Ir(d(*t*Bu)(CF$_3$)ppy)$_2$(dtbbpy)][Cl] (5.4 mg, 0.005 mmol, 0.5 mol%), 2-mercaptoethan-1-ol (78 mg, 1 mmol, 1.0 equiv.), *N*,*N*-diisopropylethylamine (194 mg, 1.5 mmol, 1.5 equiv.), deionized water (0.5 mL), and DMF (10 mL). Purification by column chromatography (Eluent: EtOAc) yielded the pure product as a light-brown solid (37 mg, 0.185 mmol, 37% yield).

^{1}H NMR (400 MHz, CDCl$_3$) δ 3.69 (t, *J* = 5.5 Hz, 4H), 2.72 (s, 4H), 2.79 – 2.62 (t, 4H), 2.57 (s, 2H); **^{13}C NMR** (101 MHz, CDCl$_3$) δ 60.8, 35.3, 32.1; **IR** (KBr, cm^{-1}) 3651, 1681, 1563, 1034; **HRMS** (ESI) calcd. For $C_6H_{14}O_2S_2H$ [M+H]$^+$ m/z 183.0508, found 183.0507.

3,3'-(Ethane-1,2-diylbis(sulfanediyl))dipropionic acid (82)

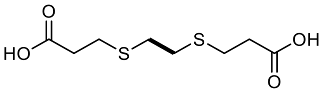

Prepared following the general procedure outlined above using [Ir(d(*t*Bu)(CF$_3$)ppy)$_2$(dtbbpy)][Cl] (2.7 mg, 0.0025 mmol, 0.5 mol%), 3-mercaptopropanoic acid (53 mg, 0.5 mmol, 1.0 equiv.), *N*,*N*-

diisopropylethylamine (97 mg, 0.75 mmol, 1.5 equiv.), deionized water (0.25 mL), and DMF (5 mL). Purification by column chromatography (Eluent: DCM/CH_3OH = 10/1) yielded the pure product as a yellow solid (33 mg, 0.137 mmol, 55% yield).

^{1}H NMR (400 MHz, *d*$_6$-DMSO) δ 12.23 (s, 2H), 2.78 – 2.63 (m, 8H), 2.50 (t, *J* = 7.1 Hz, 4H); **^{13}C NMR** (101 MHz, *d*$_6$-DMSO) δ 173.5, 35.1, 31.8, 26.8; **IR** (KBr, cm^{-1}) 3728, 3163, 1397, 1107, 563, 497; **HRMS** (ESI) calcd. For $C_8H_{14}O_4S_2Na$ [M+Na]$^+$ m/z 261.0226, found 261.0231.

1,2-Bis((adamantan-1-yl)thio)ethane (83)

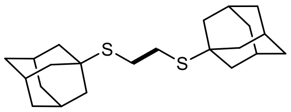

Prepared following the general procedure outlined above using [Ir(d(*t*Bu)(CF_3)ppy)$_2$(dtbbpy)][Cl] (2.7 mg, 0.0025 mmol, 0.5 mol%), 3-mercaptopentan-2-one (84 mg, 0.5 mmol, 1.0 equiv.), *N*,*N*-diisopropylethylamine (97 mg, 0.75 mmol, 1.5 equiv.), deionized water (0.25 mL), and DMF (5 mL). Purification by column chromatography (Eluent: petroleum ether/EtOAc = 70/1) yielded the pure product as a yellow solid (69 mg, 0.19 mmol, 76% yield).

^{1}H NMR (400 MHz, $CDCl_3$) δ 2.65 (s, 4H), 2.02 (broad s, 6H), 1.85 (s, 12H), 1.73 – 1.59 (m, 12H); ^{13}C NMR (101 MHz, $CDCl_3$) δ 44.7, 43.7, 36.3, 29.7, 26.9, 1.0; **IR** (KBr, cm^{-1}) 3731, 3230, 2897, 1707, 1371, 1026, 802, 701, 562; **Mp**: 115.7 – 117.7 °C; **HRMS** (ESI) calcd. For $C_{22}H_{34}S_2Na$ [M+Na]$^+$ m/z 385.1994, found 385.1990. The analytical datas are in agreement with the literature.[8]

(1*S*,2*S*,4*R*)-1,3,3-Trimethylbicyclo[2.2.1]heptan-2-yl2-((2-((2-oxo-2-(((1R,2R)-1,3,3-trimethyl bicyclo[2.2.1]heptan-2-yl)oxy)ethyl)thio)ethyl)thio)acetate (84)

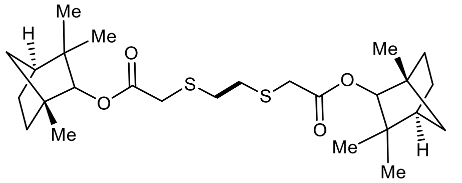

Prepared following the general procedure outlined above using [Ir(d(*t*Bu)(CF_3)ppy)$_2$(dtbbpy)][Cl] (2.7 mg, 0.0025 mmol, 0.5 mol%), 1,3,3-trimethylbicyclo[2.2.1]heptan-2-yl 2-mercaptoacetate (114 mg, 0.5 mmol, 1.0 equiv.), *N*,*N*-diisopropylethylamine (97 mg, 0.75 mmol, 1.5 equiv.), deionized water (0.25 mL), and DMF (5 mL). Purification by column chromatography (Eluent: petroleum ether/EtOAc = 95/1) yielded the pure product as a colorless liquid (94 mg, 0.195 mmol, 78% yield).

^{1}H NMR (400 MHz, $CDCl_3$) δ 4.40 (d, *J* = 1.4 Hz, 2H), 3.30 (s, 4H), 2.92 (s, 4H), 1.81 – 1.65 (m, 6H), 1.59 (d, *J* = 10.4 Hz, 2H), 1.55 – 1.41 (m, 2H), 1.21 (d, *J* = 10.4 Hz, 2H), 1.17 – 1.03 (m, 14H), 0.82 (s, 6H); **^{13}C NMR** (101 MHz, $CDCl_3$) δ 170.7, 87.4, 48.3, 48.3, 41.3, 39.5, 33.6, 32.0, 29.7, 26.6, 25.8, 20.2, 19.4; **IR** (KBr, cm^{-1}) 3731, 3202, 2954, 2875, 2320, 1729, 1453, 1371, 1278, 1036, 463; **HRMS** (ESI) calcd. For $C_{26}H_{42}O_4S_2H$ [M+H]$^+$ m/z 483.2597, found 483.2595.

1,2-Bis(((3*R*,8*S*,9*S*,10*R*,13*R*,14*S*,17*R*)-10,13-dimethyl-17-((*R*)-6-methylheptan-2-yl)-2,3,4,7,8,9,10,11,12,13,14,15,16,17-tetradecahydro-1*H*-cyclopenta[*a*]phenanthren-3-yl)thio)ethane (85)

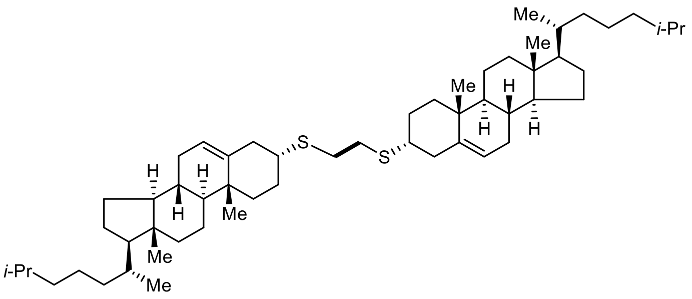

Prepared following the general procedure outlined above using [Ir(d(*t*Bu)(CF_3)ppy)$_2$(dtbbpy)][Cl] (2.7 mg, 0.0025 mmol, 0.5 mol%), thiocholesterol (201 mg, 0.5 mmol, 1.0 equiv.), *N*,*N*-diisopropylethylamine (97 mg, 0.75 mmol, 1.5 equiv.), deionized water (0.25 mL), and DMF (5 mL). Purification by column chromatography (Eluent: petroleum ether/EtOAc = 100/1) yielded the pure product as a yellow solid (106 mg, 0.127 mmol, 51% yield)

^{1}H NMR (400 MHz, $CDCl_3$) δ 5.34 (s, 2H), 3.23 (s, 2H), 2.74 (d, *J* = 15.5 Hz, 2H), 2.68 – 2.62 (m, 4H), 2.12 (d, *J* = 14.6 Hz, 2H), 2.08 – 1.92 (m, 6H), 1.91 – 1.79 (m, 2H), 1.77 – 1.50 (m, 15H), 1.49 – 1.24 (m, 13H), 1.23 – 0.98 (m, 24H), 0.94 (d, *J* = 6.4 Hz, 6H), 0.89 (dd, *J* = 6.6, 1.6 Hz, 12H), 0.69 (s, 6H); **^{13}C NMR** (101 MHz, $CDCl_3$) δ 138.9, 122.4, 56.7, 56.2, 49.9, 43.8, 42.3, 39.8, 39.5, 37.7, 37.2, 36.2, 35.8, 33.8, 31.8, 31.8, 31.3, 28.3, 28.0, 26.9, 24.3, 23.9, 22.9, 22.6, 20.8, 19.3, 18.7, 11.9; **IR** (KBr, cm^{-1}) 3730, 3291, 2948, 2704, 1507, 1295, 821, 567, 458; **Mp**: 203.8 – 205.8 °C; **HRMS** (ESI) calcd. For $C_{56}H_{94}S_2Na$ [M+Na]$^+$ m/z 853.6689, found 853.6697.

2,4,6-Trimethylbenzenethiol (86)

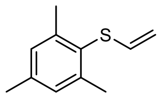

Prepared following the general procedure outlined above using [Ir(d(*t*Bu)(CF_3)ppy)$_2$(dtbbpy)][Cl] (2.7 mg, 0.0025 mmol, 0.5 mol%), 2,4,6-trimethylbenzenethiol (76 mg, 0.5 mmol, 1.0 equiv.), *N*,*N*-diisopropylethylamine (97 mg, 0.75 mmol, 1.5 equiv.), deionized water (0.25 mL), and DMF (5 mL). Purification by column chromatography (Eluent: petroleum ether/EtOAc = 150/1) yielded the pure product as a yellow solid (60 mg, 0.34 mmol, 68% yield).

^{1}H NMR (400 MHz, $CDCl_3$) δ 7.07 (s, 2H), 6.38 (dd, *J* = 16.3, 9.7 Hz, 1H), 5.13 (d, *J* = 9.7 Hz, 1H), 4.68 (d, *J* = 16.3 Hz, 1H), 2.53 (s, 6H), 2.39 (s, 3H); **^{13}C NMR** (101 MHz, $CDCl_3$) δ 143.3, 139.1, 131.7, 129.3, 125.9, 108.9, 21.4, 21.1; **HRMS** (ESI) calcd. For $C_{11}H_{14}SH$ [M+H]$^+$ m/z 1179.0889, found 179.0886.

(4-Nitrophenyl)(vinyl)sulfane (87)

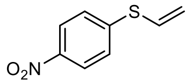

Following the general procedure **A**, using [Ir(d(*t*Bu)(CF_3)ppy)$_2$(dtbbpy)][Cl] (2.7 mg, 0.0025 mmol, 0.5 mol%), 4-nitrobenzenethiol (76 mg, 0.5 mmol, 1.0 equiv.), *N*,*N*-diisopropylethylamine (97 mg, 0.75 mmol, 1.5 equiv.), deionized water (0.25 mL), and DMF (5 mL). Purification by column chromatography (Eluent: petroleum ether/EtOAc = 15/1) yielded the pure product as yellow oil (40 mg, 0.225 mmol, 45% yield).

^{1}H NMR (400 MHz, CDCl3) δ 8.15 (d, *J* = 8.5 Hz, 2H), 7.40 (d, *J* = 8.5 Hz, 2H), 6.59 (dd, *J* = 16.6, 9.4 Hz, 1H), 5.70 (m, *J* = 12.5 Hz, 2H); **^{13}C NMR** (101 MHz, $CDCl_3$) δ 145.9, 145.6, 127.8, 127.5, 124.1, 122.1; **IR** (KBr, cm^{-1}) 3732, 3157, 1397, 1107, 497, 460; **HRMS** (ESI) calcd. For $C_8H_7NO_2SH$ $[M+H]^+$ m/z 182.0270, found 182.0264.

Methyl *N*-(4-chlorobenzyl)-*S*-vinylcysteinate (88)

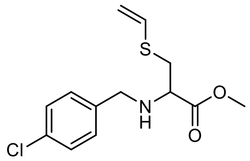

Prepared following the general procedure outlined above using [Ir(d(*t*Bu)(CF_3)ppy)$_2$(dtbbpy)][Cl] (2.7 mg, 0.0025 mmol, 0.5 mol%), L-Cysteine methyl ester hydrochloride (86 mg, 0.5 mmol, 1.0 equiv.), *N*,*N*-diisopropylethylamine (97 mg, 0.75 mmol, 1.5 equiv.), deionized water (0.25 mL), and DMF (5 mL) and the mixture was stirred for 20 h at room temperature. After completion, the resulting free amino were not isolated but instead were directly subjected to workup to provide the corresponding protected products. Potassium acetate (54 mg, 0.55 mmol, 1.1 equive) 1-(bromomethyl)-4-chlorobenzene (102 mg, 0.5 mmol, 1 equive)), were added to the same tube under air. The tube was then sealed and was stirred at 80 °C. After completion, the reaction mixture was diluted with AcOEt (10 mL), quenched with water (5 mL) and stirred for 5 min. The mixture was then extracted with AcOEt (3 x 10 mL) and the combined organic layers were washed with a saturated aqueous $NaHCO_3$ solution (5 mL), water (5 mL) and brine (10 mL), and dried over Na_2SO_4. The combined organic layers were concentrated in vacuo and purification by column chromatography yielded the pure product as a light-yellow liquid (28 mg, 0.10 mmol, 20% yield).
^{1}H NMR (400 MHz, $CDCl_3$) δ 7.34 – 7.24 (m, 4H), 6.32 (dd, *J* = 16.7, 10.0 Hz, 1H), 5.21 (dd, *J* = 13.3, 10.3 Hz, 2H), 3.86 (d, *J* = 13.4 Hz, 1H), 3.76 (s, 3H), 3.69 (d, *J* = 13.4 Hz, 1H), 3.52 (t, *J* = 6.2 Hz, 1H), 3.02 (qd, *J* = 13.5, 6.2 Hz, 2H); **^{13}C NMR** (101 MHz, $CDCl_3$) δ 173.6, 137.9, 132.9, 131.8, 129.6, 128.5, 112.4, 59.8, 52.2, 51.2, 35.1; **IR** (KBr, cm^{-1}) 3730, 3298, 2894, 1737, 1371, 1164, 1028, 501; **HRMS** (ESI) calcd. For $C_{13}H_{16}ClNO_2SNa$ $[M+Na]^+$ m/z 308.0482, found 308.0478.

Adamantan-1-yl)(2-((2,4,6-triisopropylphenyl)thio)ethyl)sulfane (89)

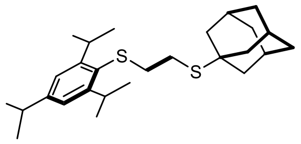

Prepared following the general procedure outlined above using [Ir(d(*t*Bu)(CF_3)ppy)$_2$(dtbbpy)][Cl] (2.7 mg, 0.0025 mmol, 1 mol%), 2,4,6-triisopropylbenzenethiol (59 mg, 0.25 mmol, 1.0 equiv.), adamantane-1-thiol (46 mg, 0.28 mmol, 1.1 equiv.), *N*,*N*-diisopropylethylamine (97 mg, 0.75 mmol, 1.5 equiv.), deionized water (0.25 mL), and DMF (5 mL). Purification by column chromatography (Eluent: petroleum ether/EtOAc = 85/1) yielded the pure product as a white solid (69 mg, 0.16 mmol, 64% yield).
^{1}H NMR (400 MHz, $CDCl_3$) δ 7.05 (s, 2H), 3.98 (hept, *J* = 6.8 Hz, 2H), 2.91 (dt, *J* = 13.8, 6.9 Hz, 1H), 2.84 – 2.74 (m, 2H), 2.74 – 2.58 (m, 2H), 2.01 (s, 3H), 1.78 (d, *J* = 2.2 Hz, 5H), 1.66 (dd, *J* = 27.6, 12.6 Hz, 7H), 1.27 (dd, *J* = 6.9, 4.2 Hz, 18H); **^{13}C NMR** (101 MHz, $CDCl_3$) δ 153.1, 149.7, 127.9, 121.7, 45.0, 43.7, 43.7, 38.9, 36.3, 36.3, 34.3, 31.6, 29.8, 29.7, 25.6, 24.5, 23.9; **IR** (KBr, cm$^-$

^{1}) 3855, 3744, 3673, 2906, 1681, 1518, 1365, 677; **Mp**: 59.1 – 62.2 °C; **HRMS** (ESI) calcd. For $C_{27}H_{42}S_2H$ $[M+H]^+$ m/z 431.2801, found 431.2805.

Ethyl 2-((2-((2,4,6-triisopropylphenyl)thio)ethyl)thio)acetate (90)

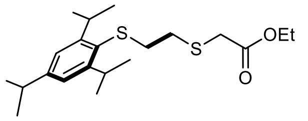

Prepared following the general procedure outlined above using [Ir(d(*t*Bu)(CF_3)ppy)$_2$(dtbbpy)][Cl] (2.7 mg, 0.0025 mmol, 1 mol%), 2,4,6-triisopropylbenzenethiol (59 mg, 0.25 mmol, 1.0 equiv.), ethyl 2-mercaptoacetate (33 mg, 0.28 mmol, 1.1 equiv.), *N*,*N*-diisopropylethylamine (97 mg, 0.75 mmol, 1.5 equiv.), deionized water (0.25 mL), and DMF (5 mL). Purification by column chromatography (Eluent: petroleum ether/EtOAc = 50/1) yielded the pure product as a yellow liquid (70 mg, 0.18 mmol, 73% yield).

^{1}H NMR (400 MHz, $CDCl_3$) δ 7.01 (s, 2H), 4.14 (q, *J* = 7.1 Hz, 2H), 4.02 – 3.79 (m, 2H), 3.19 (s, 2H), 2.97 – 2.63 (m, 5H), 1.28 – 1.18 (m, 21H); **^{13}C NMR** (101 MHz, $CDCl_3$) δ 170.2, 153.1, 149.8, 127.5, 121.9, 61.4, 36.9, 34.3, 33.6, 32.20, 31.5, 24.5, 23.9, 14.2; **IR** (KBr, cm^{-1}) 3705, 3627, 3171, 2962, 1658, 1569, 1392, 1269, 1096; **HRMS** (ESI) calcd. For $C_{21}H_{34}S_2O_2Na$ $[M+Na]^+$ m/z 405.1892, found 405.1891.

***tert*-Butyl (2-((2-((2,4,6-triisopropylphenyl)thio)ethyl)thio)ethyl)carbamate (91)**

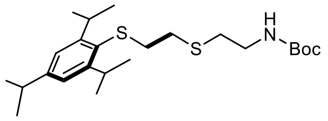

Prepared following the general procedure outlined above using [Ir(d(*t*Bu)(CF_3)ppy)$_2$(dtbbpy)][Cl] (2.7 mg, 0.0025 mmol, 1 mol%), 2,4,6-triisopropylbenzenethiol (59 mg, 0.25 mmol, 1.0 equiv.), *tert*-butyl (2-mercaptoethyl)carbamate (48 mg, 0.28 mmol, 1.1 equiv.), *N*,*N*-diisopropylethylamine (97 mg, 0.75 mmol, 1.5 equiv.), deionized water (0.25 mL), and DMF (5 mL). Purification by column chromatography (Eluent: petroleum ether/EtOAc = 50/1) yielded the pure product as a white solid (84 mg, 0.19 mmol, 75% yield).

^{1}H NMR (400 MHz, $CDCl_3$) δ 7.04 (s, 2H), 4.93 (s, 1H), 3.94 (hept, *J* = 6.8 Hz, 2H), 3.29 (d, *J* = 6.0 Hz, 2H), 2.90 (dt, *J* = 13.8, 6.9 Hz, 1H), 2.83 (dd, *J* = 9.7, 5.7 Hz, 2H), 2.75 – 2.59 (m, 4H), 1.46 (s, 9H), 1.27 (t, *J* = 6.9 Hz, 18H); **^{13}C NMR** (101 MHz, $CDCl_3$) δ 155.7, 153.0, 149.8, 127.6, 121.9, 79.5, 39.7, 37.6, 34.3, 32.4, 31.5, 31.4, 28.4, 24.5, 23.9; **IR** (KBr, cm^{-1}) 3851, 3745, 3672, 3129, 2961, 2921, 1398, 1136; **Mp**: 66.7 – 69.2 °C; **HRMS** (ESI) calcd. For $C_{24}H_{41}NO_2S_2Na$ $[M+Na]^+$ m/z 462.2471, found 462.2469.

Methyl *N*-(2,2,2-trifluoroacetyl)-*S*-(2-((2,4,6-triisopropylphenyl)**thio)ethyl)-*L*-cysteinate (92)**

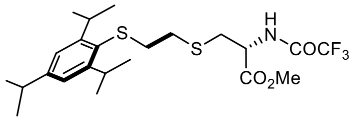

Prepared following the general procedure outlined above using [Ir(d(*t*Bu)(CF_3)ppy)$_2$(dtbbpy)][Cl] (2.7 mg, 0.0025 mmol, 1 mol%), 2,4,6-triisopropylbenzenethiol (59 mg, 0.25 mmol, 1.0 equiv.), methyl (2,2,2-trifluoroacetyl)-*L*-cysteinate (63 mg, 0.28 mmol, 1.1 equiv.), *N*,*N*-diisopropylethylamine (97 mg, 0.75 mmol, 1.5 equiv.), deionized water (0.25 mL), and DMF (5 mL). Purification by column chromatography (Eluent: petroleum ether/EtOAc = 100/1) yielded the pure product as a faint yellow solid (64 mg, 0.13 mmol, 52% yield).

^{1}H NMR (400 MHz, $CDCl_3$) δ 7.19 (d, *J* = 6.7 Hz, 1H), 7.04 (s, 2H), 4.82 (dt, *J* = 7.5, 4.9 Hz, 1H), 3.99 – 3.86 (m, 2H), 3.78 (s, 3H), 3.17 – 3.02 (m, 2H), 2.90 (dt, *J* = 13.8, 6.9 Hz, 1H), 2.85 – 2.77 (m, 2H), 2.72 – 2.60 (m, 2H), 1.27 (t, *J* = 6.3 Hz, 18H); **^{13}C NMR** (101 MHz, $CDCl_3$) δ 169.5, 157.4, 157.1, 156.7, 156.3, 153.1, 149.9, 127.3, 121.9, 117.0, 114.1, 53.1, 53.1, 52.2, 37.2, 34.3, 33.5, 32.3, 31.6, 31.6, 24.5, 23.9; **^{19}F NMR** (471 MHz, $CDCl_3$) δ -75.78; **IR** (KBr, cm^{-1}) 3855, 3745, 3672, 3131, 1655, 1395, 1072; **Mp**: 74.8 – 76.1 °C; **HRMS** (ESI) calcd. For $C_{23}H_{34}F_3NO_3S_2Na$ $[M+Na]^+$ m/z 516.1824, found 516.2815.

Diphenyl(2-((2,4,6-triisopropylphenyl)thio)ethyl)phosphine oxide (93)

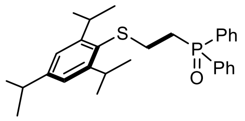

Prepared following the general procedure outlined above using [Ir(d(*t*Bu)(CF_3)ppy)$_2$(dtbbpy)][Cl] (2.7 mg, 0.0025 mmol, 1 mol%), 2,4,6-triisopropylbenzenethiol (55 mg, 0.25 mmol, 1.0 equiv.), methyl diphenylphosphine oxide (55 mg, 0.28 mmol, 1.1 equiv.), *N*,*N*-diisopropylethylamine (97 mg, 0.75 mmol, 1.5 equiv.), deionized water (0.25 mL), and DMF (5 mL). Purification by column chromatography (Eluent: petroleum ether/EtOAc = 1/1) yielded the pure product as a yellow solid (37 mg, 0.08 mmol, 32% yield).

^{1}H NMR (400 MHz, $CDCl_3$) δ 7.69 – 7.60 (m, 4H), 7.52 (td, *J* = 7.4, 1.3 Hz, 2H), 7.44 (ddd, *J* = 8.2, 5.2, 2.1 Hz, 4H), 7.01 (s, 2H), 3.83 (dt, *J* = 13.7, 6.9 Hz, 2H), 2.90 (dt, *J* = 13.8, 6.9 Hz, 1H), 2.86 – 2.76 (m, 2H), 2.64 – 2.43 (m, 2H), 1.28 (d, *J* = 6.9 Hz, 8H), 1.16 (d, *J* = 6.9 Hz, 10H); **^{13}C NMR** (101 MHz, $CDCl_3$) δ 153.2, 150.0, 132.8, 131.9, 131.9, 131.9, 130.7, 130.6, 128.8, 128.7, 127.1, 121.9, 34.3, 31.5, 30.3, 30.3, 29.7, 29.7, 24.4, 24.0; **^{31}P NMR** (202 MHz, $CDCl_3$) δ 30.64; **IR** (KBr, cm^{-1}) 3779, 3694, 3662, 3137, 2922, 1655, 1400, 1129, 727; **Mp**: 253.6 – 257.1 °C; **HRMS** (ESI) calcd. For $C_{29}H_{37}OPSNa$ $[M+Na]^+$ m/z 487.2195, found 487.2197.

6. Gram-Scale Reaction and Synthetic Transformation

6.1 Gram-Scale synthesis

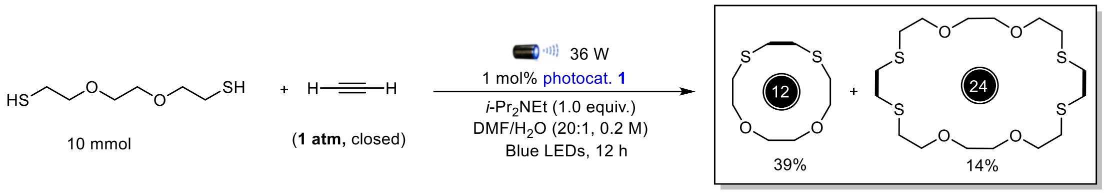

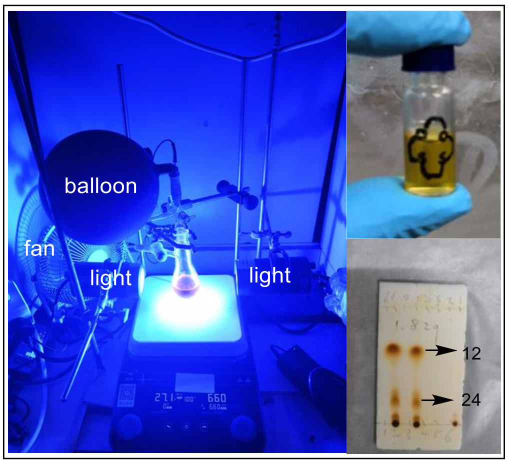

Supplementary Figure 3. Experimental set-up for the scale-up synthesis of oxo-thiocrown ethers, (in the bottom right corner) TLC of the reaction mixture after staining with Iodine (ethyl acetate/Petroleum ether = 1:5) and (in the top right corner) the desired product

To a 100 mL Schlenk round bottom flask equipped with a magnetic stir bar was charged with [Ir(d(*t*Bu)(CF$_3$)ppy)$_2$(dtbbpy)][Cl] (54 mg, 0.005 mmol, 0.5 mol %) and 2,2'-(ethane-1,2-diylbis(oxy))bis(ethane-1-thiol) (1.82 g, 10 mmol, 1.0 equiv.). The vessel was evacuated with three-way valve and refilled with acetylene gas. Subsequently, DMF (50 mL), deionized water (2.5 mL) and *N*,*N*-diisopropylethylamine (1.3 g, 10 mmol, 1.0 equiv.) were added to the tube. Finally, a balloon was filled with acetylene gas through three-way valve until its size was roughly 30 cm in diameter. The reaction mixture was stirred in front of two 420 nm Kessil lamps at room temperature for 12 hours (flask about 10 cm away from lights, fan for cooling) and then quenched with H_2O (25 mL), extracted with EtOAc (3 × 50 mL). The combined organic layer was washed with brine (50 mL), dried over Na_2SO_4, filtered and evaporated. Purification by flash column chromatography on silica gel gave 12 membered-ring (811 mg, 39%) as a pale yellow oil, 24 membered ring (291 mg, 14%) as a deeply yellow oil.

6.2 Synthetic Transformation

6.3 The oxidation of 1,4-dioxa-7,10-dithiacyclododecane to the sulfoxide

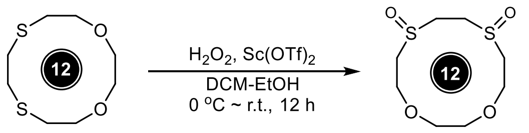

94, 81%

Following reported procedure[11], to a round-bottom flask equipped with a stirring bar was added scandium(III) triflate (68.4 mg, 0.2 mmol, 0.4 equiv.), DCM/EtOH mixture (5 mL, 9:1) and Hydrogen peroxide (5 mmol, 10.0 equiv., 30%). After stirring for 2 minutes at ambient environment, a solution of 1,4-dioxa-7,10-dithiacyclododecane (104 g, 0.5 mmol, 1.00 equiv.), previously dissolved in 5 mL of DCM/EtOH (9:1) was added. Subsequently, additional 1.5 mL solvent mixture was used to rinse the flask. The reaction was stirred under room temperature and monitored by GC-MS. The reaction was completed and H_2O (10 mL) and DCM (15 mL) were added. The aqueous layer was extracted with DCM (3 × 15 mL) and washed with H_2O (10 mL). The organic layers were combined, dried with $MgSO_4$, and filtered, and the volatiles were removed under reduced pressure. The 1,4-dioxa-7,10-dithiacyclododecane 7,10-dioxide was isolated after flash chromatography (Eluent: DCM/CH_3OH = 20/1) as white solid (97 mg, 0.40 mmol, 81%).

1,4-Dioxa-7,10-dithiacyclododecane 7,10-dioxide (94)

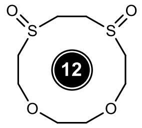

^{1}H NMR (500 MHz, $CDCl_3$) δ 4.12 – 4.01 (m, 2H), 3.76 (dddd, *J* = 25.7, 11.6, 7.4, 1.8 Hz, 2H), 3.68 – 3.51 (m, 4H), 3.45 – 3.32 (m, 4H), 3.19 (dddd, *J* = 12.6, 10.8, 7.4, 1.7 Hz, 2H), 2.93 (dddd, *J* = 26.6, 14.7, 7.8, 1.8 Hz, 2H); **^{13}C NMR** (126 MHz, $CDCl_3$) δ 70.1, 69.9, 62.0, 61.1, 50.4, 49.4, 42.8, 41.9; **IR** (KBr, cm^{-1}) 3747, 3647, 3164, 1993, 1384, 1112, 422; **Mp**: 89.8 – 91.1; **HRMS** (ESI) calcd. For $C_8H_{16}O_4NaS_2^+$ $[M+Na]^+$ m/z 263.0382, found: 263.0380.

6.4 The oxidation of 1,4-dioxa-7,10-dithiacyclododecane to the sulfone

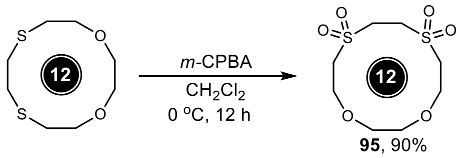

To a round-bottom flask equipped with a stirring bar was added 1,4-dioxa-7,10-dithiacyclododecane (208 g, 1.0 mmol, 1.00 equiv.) and 6 mL of dry DCM. The solution was cooled down to 0 °C and 3-chloroperbenzoic acid (85%) (896 mg, 4.4 mmol, 4.4 equiv.) in DCM (12 mL) was added dropwise. The resultant mixture was stirred at 0 °C for 30 minutes, warmed up to room temperature and stirred for additional 12 h. The reaction was monitored by TLC (staining with Iodine). Next, saturated $NaHCO_3$ solution (10 mL) and water (10 mL) were added carefully, and the product was extracted into DCM (3 × 15 mL). The organic layers were combined, dried with $MgSO_4$, and filtered, and the volatiles were removed under reduced pressure. The 1,4-dioxa-7,10-dithiacyclododecane 7,7,10,10-tetraoxide was isolated after flash chromatography (Eluent: DCM/CH_3OH = 20/1) as white solid (245 mg, 0.90 mmol, 90%).

1,4-Dioxa-7,10-dithiacyclododecane 7,7,10,10-tetraoxide (95)

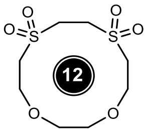

^{1}H NMR (500 MHz, DMSO-d_6) δ 3.82 – 3.74 (m, 4H), 3.58 (d, J = 10.7 Hz, 8H), 3.44 – 3.35 (m, 4H); **^{13}C NMR** (126 MHz, DMSO-d_6) δ 69.9 64.9, 54.0, 49.5; **IR** (KBr, cm^{-1}) 3175, 1646, 1398, 1384, 1111, 421; **Mp**: 146.9 – 157.8; **HRMS** (ESI) calcd. For $C_8H_{16}O_6NaS_2^+$ $[M+Na]^+$ m/z 295.0281, found: 295.0280.

6.5 Transition metal salts coordinate with the *oxo*-thiocrown ether

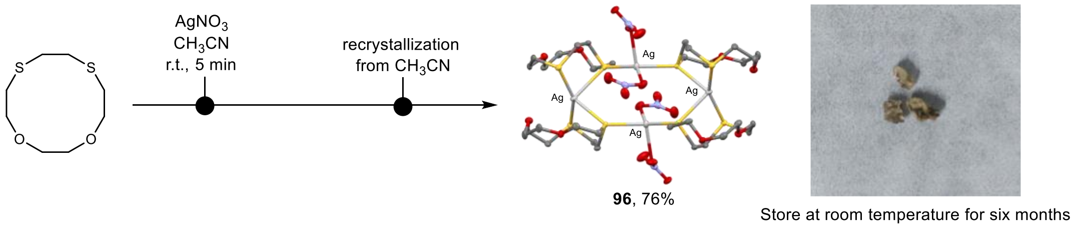

Supplementary Figure 4. synthesis of metal complexes and crystal structure of **96** with hydrogens omitted for clarity

Following reported procedure with slight modification[12], to a 10 mL Schlenk tube equipped with a magnetic stir bar was added 1,4-dioxa-7,10-dithiacyclododecane (104 mg, 0.5 mmol) and 5 mL of CH_3CN. After then, $AgNO_3$ (85 mg, 0.5 mmol) in anhydrous methanol (3.0 mL) was added in one portion. After 5 minutes a colorless clear liquid formed, which was crystallized from CH_3CN.

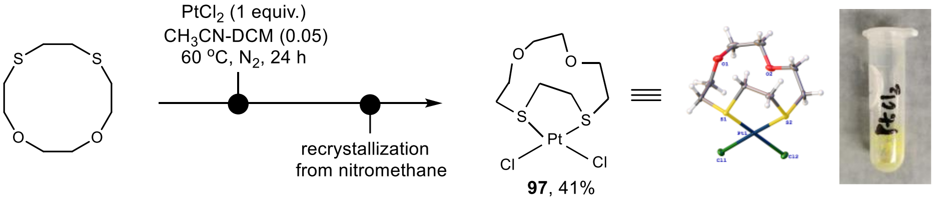

Supplementary Figure 5. synthesis of metal complexes and crystal structure of **97**

Following reported procedure with slight modification[13], to a 10 mL Schlenk tube equipped with a magnetic stir bar was added $PtCl_2$ (132 mg, 0.5 mmol), and the tube was evacuated and backfilled with N_2 (three times). 1,4-dioxa-7,10-dithiacyclododecane (104 mg, 0.5 mmol) in acetonitrile/dichloromethane (2.0 mL, v/v = 3/1) were added by syringe under a nitrogen stream. The tube was then sealed and was refluxed for 24 h. After completion, the yellow solution was filtered hot and allow to cool. Addition was diethyl ether afforded complex as a yellow solid. Recrystallisation from nitromethane yielded orange crystals at ambient temperature. HRMS (ESI) calcd. For $C_8H_{18}Cl_2O_2NaPtS_2^+$ $[M+Na]^+$ m/z 497.9665, found: 497.9664

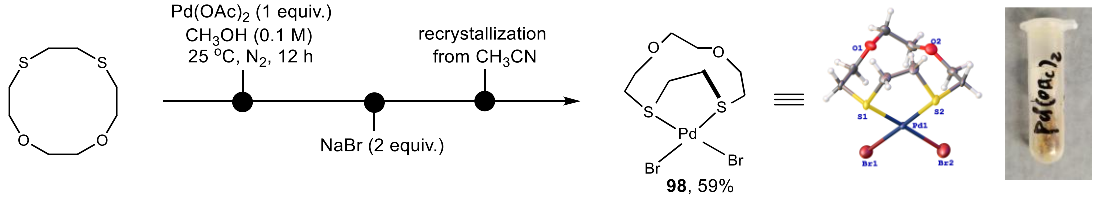

Supplementary Figure 6. synthesis of metal complexes and crystal structure of **98**

Following reported procedure[13,14], to a 10 mL Schlenk tube equipped with a magnetic stir bar was evacuated and backfilled with N_2 (three times). After then, 1,4-dioxa-7,10-dithiacyclododecane (41.6 mg, 0.2 mmol) in anhydrous methanol (1.0 mL) was added by microsyringe under a nitrogen stream. Subsequently, $Pd(OAc)_2$ (22.4 mg, 0.1 mmol) was added at ambient environment, then the reaction mixture was added NaBr (20 mg, 0.2 mmol) in methanol (0.5 mL). Orange-brown crystals immediately separated out; these were recrystallized from acetonitrile. HRMS (ESI) calcd. For $C_8H_{18}Cl_2O_2NaPdS_2^+$ $[M+Na]^+$ m/z 496.8042, found: 496.8031.

6.6 Gram-Scale synthesis of bis-Sulfoxide

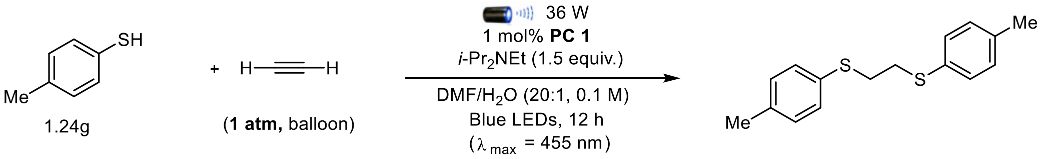

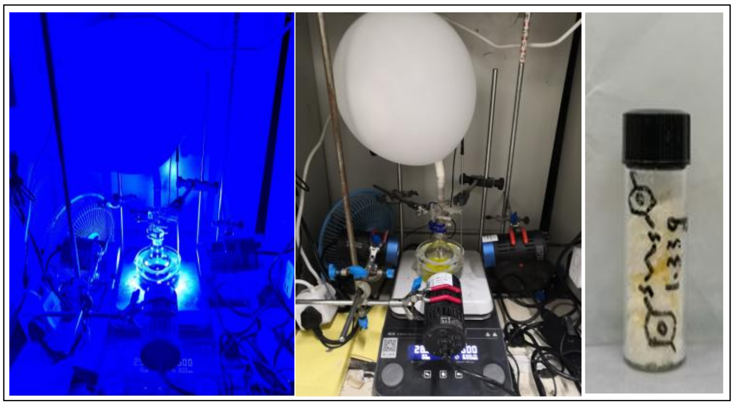

Supplementary Figure 2: Experimental set-up for the scale-up reaction of the reaction-condition-based sensitivity assessment. (right) the desired product 1,2-bis(*p*-tolylthio)ethane.

A 100 mL round bottom flask equipped with a stirring bar was charged with $[Ir(d(tBu)(CF_3)ppy)_2(dtbbpy)][Cl]$ (1 mol%) and 4-methylbenzenethiol (1.24 g, 10 mmol, 1.0 equiv.). The vessel was evacuated with three-way valve and refilled with acetylene gas three times,

N,N-Dimethylformamide and *N,N*-diisopropylethylamine (15 mmol, 1.5 equiv.) were added followed by deionized water. In the end a balloon was filled with acetylene gas through three-away valve until its size was roughly 30 cm in diameter. The mixture was stirred in front of two 420 nm and one 430 nm Kessil lamps at room temperature for 12 hours (flask about 8 cm away from lights, fan and water bath for cooling, 25 ~ 30 °C) and then diluted with H_2O (25 mL) and EtOAc (50 mL). The layers were separated and the aqueous layer was extracted with EtOAc (50 mL × 3). The combined organic layers are washed with brine (50 mL), dried ($NaSO_4$), filtered and evaporated under reduced pressure. Purification by flash column chromatography on silica gel (Eluent: petroleum ether/EtOAc = 50:1) gave 1,2-bis(*p*-tolylthio)ethane (1.33 g, 97%) as an snowflake solid.

1,2-Bis(*p*-tolylsulfinyl)ethane gram-scale synthesis by oxidation with H_2O_2

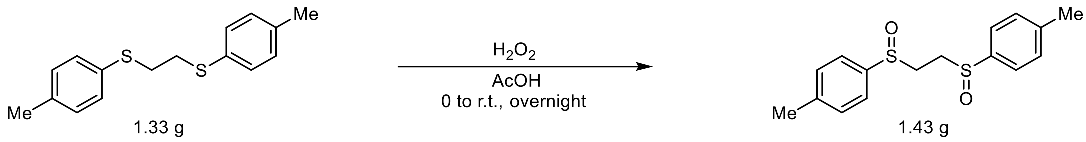

The following procedure is adapted from the literature[15]. A 100 mL round bottom flask was charged with a stir bar, 1.33g (4.9 mmol) of 1,2-bis(*p*-tolylthio)ethane, and 10 mL of glacial acetic acid. The mixture was allowed to stir at room temperature until it became homogeneous at which time it was cooled to 0 °C and hydrogen peroxide 30% aqueous solution H_2O_2 (1.23 g, 10.8 mmol) was added dropwise. The reaction was allowed to room temperature and was stirred overnight. The acetic acid was removed with mild heating (50 °C) under high vacuum and the white solid was washed with EtOH until the washings were clear (3 × 20 mL). The white solid was dried under high vacuum to give (1.43 g, 4.7 mmol) of 1,2-bis(*p*-tolylsulfinyl)ethane in 96% yield as a white solid, which was employed without further purification for the next step.

^{1}H NMR (400 MHz, $CDCl_3$) δ 7.42 – 7.31 (m, 4H), 7.31 – 7.19 (m, 4H), 3.28 (dd, *J* = 17.1, 7.2 Hz, 1H), 2.96 (d, *J* = 2.4 Hz, 2H), 2.68 (dd, *J* = 16.8, 7.5 Hz, 1H), 2.34 (d, *J* = 6.1 Hz, 6H); **^{13}C NMR** (101 MHz, $CDCl_3$) δ 141.8, 141.8, 139.1, 138.9, 130.1, 130.1, 124.0, 123.9, 47.7, 46.9, 21.4; **IR** (KBr, cm^{-1}) 3730, 3629, 3298, 2739, 1498, 1296, 1046, 498; **HRMS** (ESI) calcd. For $C_{16}H_{18}O_2S_2Na$ $[M+Na]^+$ m/z 329.0646, found 329.0641. The analytical datas are in agreement with the literature.[8]

6.7 Synthesis of Chiral 2-Sulfinyl-Phosphonate

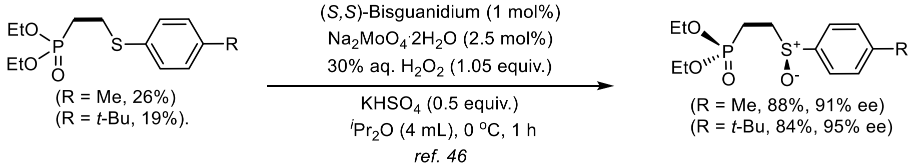

The following procedure is adapted from the literature[16], an oven-dried Schlenk tube (10 mL) containing a stirrer bar was charged with with a solution of sulfide (0.2 mmol) and bisguanidinium phase-transfer catalyst (*S,S*)-Bisguanidium (developed by *Choon-Hong Tan Groups, Nanyang Technological University, 21 Nanyang Link, Singapore 637371, Singapore*) (2.8 mg, 0.002 mmol) in *i*-Pr_2O (4mL). Then $Na_2MoO_4·2H_2O$ (1.2 mg, 0.005 mmol) and $KHSO_4$ (13.6 mg, 0.1 mmol) were added. The reaction mixture was stirred for 5min in an ice bath, and then hydrogen peroxide 30% aqueous solution H_2O_2 (21 uL, 0.21 mmol) was added in one portion. The resulting mixture was stirred vigorously at 0 °C and monitored by thin-layer chromatography until thioether was completely consumed. Purification by flash column chromatography on silica gel (Eluent: EtOAc)

gave chiral 2-sulfinyl-phosphonate (53.6 mg, 88% and 58.2 mg, 84%) as a colorless oil.

Diethyl (2-(*p*-tolylthio)ethyl)phosphonate (101)

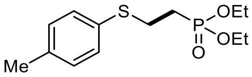

Prepared following the general procedure outlined above using [Ir(d(*t*Bu)(CF_3)ppy)$_2$(dtbbpy)][Cl] (2.7 mg, 0.0025 mmol, 1 mol%), diethyl phosphonate (35 mg, 0.25 mmol, 1.0 equiv.), 4-methylbenzenethiol (35 mg, 0.28 mmol, 1.1 equiv.), *N*,*N*-diisopropylethylamine (97 mg, 0.75 mmol, 1.5 equiv.), deionized water (0.25 mL), and DMF (5 mL). Purification by column chromatography (Eluent: petroleum ether/EtOAc = 3/1) yielded the pure product as a colorless liquid (19 mg, 0.07 mmol, 26% yield).

^{1}H NMR (400 MHz, $CDCl_3$) δ 7.28 (d, *J* = 7.9 Hz, 1H), 7.12 (d, *J* = 7.7 Hz, 1H), 4.21 – 3.84 (m, 2H), 3.07 (dd, *J* = 16.6, 8.1 Hz, 1H), 2.33 (s, 1H), 2.17 – 1.81 (m, 1H), 1.31 (t, *J* = 7.0 Hz, 3H); **^{13}C NMR** (101 MHz, $CDCl_3$) δ 137.0, 131.0, 130.9, 129.9, 61.8, 61.8, 27.7, 27.2, 25.8, 21.1, 16.5, 16.4; **^{31}P NMR** (162 MHz, $CDCl_3$) δ 28.73, 28.68, 28.63, 28.57, 28.52, 28.47, 28.41, 28.36, 28.31; **IR** (KBr, cm^{-1}) 3732, 3174, 1397, 1029, 674, 492; **HRMS** (ESI) calcd. For $C_{13}H_{21}O_3PSNa$ $[M+Na]^+$ m/z 311.0841, found 311.0840.

Diethyl (*S*)-(2-(p-tolylsulfinyl)ethyl)phosphonate (103)

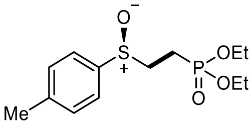

^{1}H NMR (500 MHz, $CDCl_3$) δ 7.47 (d, *J* = 7.9 Hz, 2H), 7.34 (d, *J* = 7.9 Hz, 2H), 4.17 – 3.94 (m, 4H), 3.13 (tdd, *J* = 12.8, 8.6, 4.0 Hz, 1H), 2.85 (tdd, *J* = 12.9, 8.0, 4.5 Hz, 1H), 2.42 (s, 3H), 2.29 – 2.11 (m, 1H), 1.81 (ddd, *J* = 27.7, 18.7, 4.4 Hz, 1H), 1.29 (dd, *J* = 13.1, 6.7 Hz, 6H); **^{13}C NMR** (126 MHz, $CDCl_3$) δ 141.8, 139.1, 130.0, 124.1, 62.1, 62.0, 49.1, 49.1, 21.4, 18.0, 16.8, 16.4, 16.3; **^{31}P NMR** (202 MHz, $CDCl_3$) δ 28.82, 28.78, 28.74, 28.69, 28.65, 28.61, 28.56, 28.52, 28.47; **HRMS** (ESI) calcd. For $C_{13}H_{21}O_4PSNa$ $[M+Na]^+$ m/z 327.0796, found 327.0792; Resolution of enantiomers: **HPLC** (FLM chiral INA column, column size: 250*4.6 mmI.D., injection: 3μL, mobile phase: hexane/isopropanol = 75/25, flow rate: 0.8 mL/min, back pressure: 2.1 MPa, detection: UV254 nm) retention time = 7.4 min (minor) and 6.5 min (major), ee = 90%.

Diethyl (2-((4-(*tert*-butyl)phenyl)thio)ethyl)phosphonate (102)

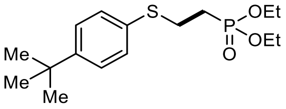

Prepared following the general procedure outlined above using [Ir(d(*t*Bu)(CF_3)ppy)$_2$(dtbbpy)][Cl] (2.7 mg, 0.0025 mmol, 1 mol%), diethyl phosphonate (35 mg, 0.25 mmol, 1.0 equiv.), 4-methylbenzenethiol (46 mg, 0.28 mmol, 1.1 equiv.), *N*,*N*-diisopropylethylamine (97 mg, 0.75 mmol, 1.5 equiv.), deionized water (0.25 mL), and DMF (5 mL). Purification by column chromatography (Eluent: petroleum ether/EtOAc = 3/1) yielded the pure product as a colorless liquid (19 mg, 0.05 mmol, 19% yield).

^{1}H NMR (400 MHz, $CDCl_3$) δ 7.38 – 7.24 (m, 4H), 4.09 (dq, *J* = 14.2, 7.2 Hz, 4H), 3.09 (dd, *J* = 16.6, 8.1 Hz, 2H), 2.27 – 1.79 (m, 2H), 1.46 – 1.05 (m, 15H); **^{13}C NMR** (101 MHz, $CDCl_3$) δ 150.1, 131.1, 130.4, 126.2, 61.8, 61.8, 34.5, 31.3, 27.5, 27.5, 27.2, 25.9, 16.5, 16.4; **^{31}P NMR** (162 MHz,

$CDCl_3$) δ 28.76, 28.71, 28.66, 28.60, 28.55, 28.50, 28.44, 28.39, 28.34; **IR** (KBr, cm^{-1}) 2962, 2905, 1244, 1056, 1029, 964, 825, 741, 530; **HRMS** (ESI) calcd. For $C_{16}H_{27}O_3PSH$ $[M+H]^+$ m/z 331.1491, found 331.1485.

Diethyl (*S*)-(2-((4-(*tert*-butyl)phenyl)sulfinyl)ethyl)phosphonate (104)

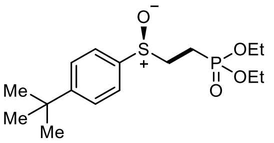

^{1}H NMR (400 MHz, $CDCl_3$) δ 7.52 (q, *J* = 8.4 Hz, 1H), 4.06 (dt, *J* = 12.1, 6.0 Hz, 1H), 3.25 – 3.01 (m, 1H), 2.86 (tdd, *J* = 12.7, 8.0, 4.5 Hz, 1H), 2.34 – 2.02 (m, 1H), 1.82 (ddd, *J* = 27.6, 18.6, 4.4 Hz, 1H), 1.33 (s, 2H), 1.28 (td, *J* = 7.0, 3.2 Hz, 2H); **^{13}C NMR** (101 MHz, $CDCl_3$) δ 155.0, 138.9, 126.4, 123.9, 62.1, 62.1, 49.0, 35.0, 31.2, 18.1, 16.7, 16.4, 16.3; **^{31}P NMR** (162 MHz, $CDCl_3$) δ 28.97, 28.92, 28.87, 28.81, 28.76, 28.71, 28.64, 28.59, 28.54; **HRMS** (ESI) calcd. For $C_{16}H_{27}O_3PSH$ $[M+H]^+$ m/z 347.1440, found 347.1443; **IR** (KBr, cm^{-1}) 2981, 2926, 1240, 1049, 1030, 965, 813, 739, 511; Resolution of enantiomers: **HPLC** (FLM chiral INA column size: 250*4.6 mmI.D., injection: 4μL, mobile phase: hexane/isopropanol = 50/50, flow rate: 0.7 mL/min, back pressure: 2.1 MPa, detection at 254 nm, retention time = 4.6 min (minor) and 4.3 min (major), ee = 95%.

6.8 Synthesis of Tiamulin (a pleuromutilin antibiotic drug)

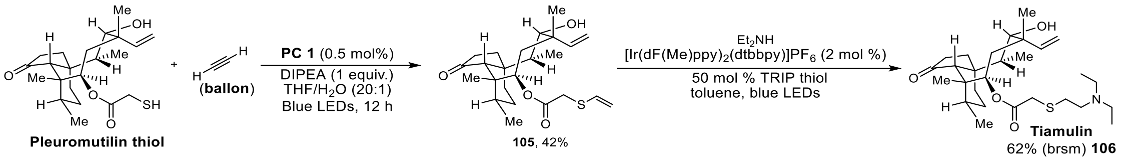

An oven-dried 25 mL Schlenk flask, equipped with a Teflon-coated magnetic stir bar, was charged with $[Ir[d(tBu)(CF_3)ppy]_2(dtbbpy)[Cl]$ (0.0025 mmol, 0.005 equiv., 2.7 mg), pleuromutilin thiol (197 mg, 0.5 mmol, 1.0 equiv.), THF (10 mL), H_2O (0.5 mL) and *N*,*N*-diisopropylethylamine (65 mg, 0.5 mmol, 1 equiv.). The reaction mixture was then cooled to -78 ºC and degassed via freeze-pump-thaw, backfilled with acetylene gas, and warmed to room temperature. A balloon, connected to a 5 mL plastic syringe head, was filled with acetylene gas until its size was roughly 10 cm in diameter. A needle was attached to the head of the syringe. Immediately after, the acetylene gas contained in the balloon was used to connected with the reaction tube by piercing the septum with the needle. The mixture was stirred in front of two 420 nm and one 430 nm Kessil lamps at room temperature for 20 hours (flask about 8 cm away from lights, fan and water bath for cooling, 25 ~ 30 ºC). Then, the solvent was removed under reduced pressure with the aid of a rotary. Purification by flash column chromatography on silica gel (petroleum ether /EtOAc = 4/1) gave vinyl thioether (88.8 mg, 0.21 mmol, 42%) as a faint yellow gooey oils. The above process is the average of three individual experiments.

(3aR,4R,5R,7S,8S,9R,9aS,12R)-8-Hydroxy-4,7,9,12-tetramethyl-3-oxo-7-vinyldecahydro-4,9a-propanocyclopenta[8]annulen-5-yl 2-(vinylthio)acetate (105)

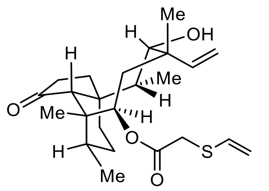

^{1}H NMR (400 MHz, $CDCl_3$) δ 6.60 – 6.46 (m, 1H), 6.46 – 6.27 (m, 1H), 5.74 (dd, J = 16.0, 8.5 Hz, 1H), 5.34 (dd, J = 11.0, 3.7 Hz, 1H), 5.28 – 5.11 (m, 2H), 3.45 – 3.22 (m, 2H), 2.35 (dq, J = 13.9, 6.9 Hz, 1H), 2.29 – 2.16 (m, 2H), 2.14 – 2.01 (m, 2H), 1.78 (d, J = 12.1 Hz, 1H), 1.66 (dd, J = 22.1, 11.3 Hz, 2H), 1.53 (d, J = 10.9 Hz, 1H), 1.49 – 1.42 (m, 4H), 1.34 (dd, J = 19.8, 10.8 Hz, 2H), 1.27 (dd, J = 12.8, 5.6 Hz, 1H), 1.22 – 1.09 (m, 4H), 0.89 (d, J = 7.0 Hz, 3H), 0.82 – 0.71 (d, 3H); **^{13}C NMR** (101 MHz, $CDCl_3$) δ 217.0, 168.0, 139.0, 130.8, 117.0, 112.7, 74.6, 69.7, 58.2, 45.5, 44.9, 43.9, 41.8, 41.7, 36.8, 36.0, 34.6, 34.4, 30.4, 26.8, 26.5, 24.8, 16.8, 14.9, 11.5; **IR** (KBr, cm^{-1}) 3555, 2934, 2882, 1735, 1458, 1413, 1281, 1150, 1116, 1016, 981, 917; **HRMS** (ESI) calcd. For $C_{24}H_{36}O_4SNa$ $[M+Na]^+$ m/z 443.2227, found 443.2229.

An oven dried 25 mL Schlenk quartz tube equipped with a stirring bar was charged with $[Ir(dF(Me)ppy)_2(dtbbpy)]PF_6$ (10.1 mg, 0.010 mmol, 2 mol%) and 2,4,6- triisopropylbezene-1-thiol (59.1 mg, 0.25 mmol, 0.5 equiv.). The reaction tube was sealed and placed under vacuum and backfilled with argon three times. Freshly degassed anhydrous toluene (10 mL, 0.05 M) was then added via syringe, followed by diethylamine (37 mg, 0.5 mmol, 1.0 equiv.) and vinyl thioether (210 mg, 0.5 mmol, 1.0 equiv.). The resulting solution was degassed using three freeze pump-thaw cycles and the tube was finally backfilled with nitrogen. The tube was then sealed and was placed upon blue LEDs lamp of Parallel Light Reactor (10 W, λ_{max} = 430 nm, tube about 2 cm away from lights). The light source parts are designed to continue flow of water entering into cooling system, and the mixture was stirred for 60 h at room temperature. The reaction has been independently three times repeated with similar results. No matter prolonging the reaction time or changing light's emission wavelengths from 410 to 530 nm, the raw materials still can't be completely consumed. The reaction mixture was purified via the specified procedure reported by the literature[7] gave the tiamulin (62%, brsm) as a colorless gooey oil.

(3a*R*,4*R*,5*R*,7*S*,8*S*,9*R*,9a*S*,12*R*)-8-Hydroxy-4,7,9,12-tetramethyl-3-oxo-7-vinyldecahydro-4,9a-propanocyclopenta[8]annulen-5-yl 2-((2-(diethylamino)ethyl)thio)acetate (106)

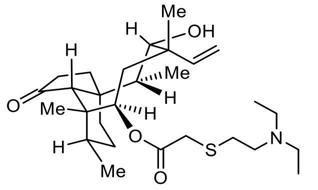

^{1}H NMR (400 MHz, $CDCl_3$) δ 6.46 (dd, J = 17.4, 11.0 Hz, 1H), 5.74 (d, J = 8.4 Hz, 1H), 5.32 (d, J = 11.0 Hz, 1H), 5.19 (d, J = 17.4 Hz, 1H), 3.44 – 3.27 (m, 1H), 3.16 (s, 2H), 2.74 – 2.58 (m, 4H), 2.52 (q, J = 7.1 Hz, 4H), 2.33 (dd, J = 13.7, 6.9 Hz, 1H), 2.22 (dt, J = 17.5, 8.6 Hz, 2H), 2.15 – 2.01 (m, 2H), 1.76 (dd, J = 14.4, 2.0 Hz, 1H), 1.65 (dd, J = 21.7, 11.8 Hz, 2H), 1.53 (dd, J = 19.3, 6.8 Hz, 2H), 1.51 – 1.40 (m, 4H), 1.35 (dd, J = 16.6, 9.2 Hz, 2H), 1.20 – 1.07 (m, 4H), 1.01 (t, J = 7.1 Hz, 6H), 0.87 (d, J = 7.0 Hz, 3H), 0.73 (d, J = 6.8 Hz, 3H); **^{13}C NMR** (101 MHz, $CDCl_3$) δ 217.0, 169.0, 139.1, 117.1, 74.6, 69.2, 58.2, 52.3, 47.0, 45.5, 44.8, 43.9, 41.8, 36.8, 36.0, 34.5, 34.5, 30.4, 30.1, 26.9, 26.4, 24.9, 16.8, 14.9, 11.9, 11.5; **IR** (KBr, cm^{-1}) 3730, 3661, 1728, 1395, 1272, 1109,

460; **HRMS** (ESI) calcd. For $C_{28}H_{47}NO_4SNa$ $[M+Na]^+$ m/z 516.3123, found 516.3125; The yield is calculated according to the conversion rate. The analytical datas are in agreement with the literature.[21]

7. Control Experiments for Mechanistic Investigation

7.1 Radical capture experiment

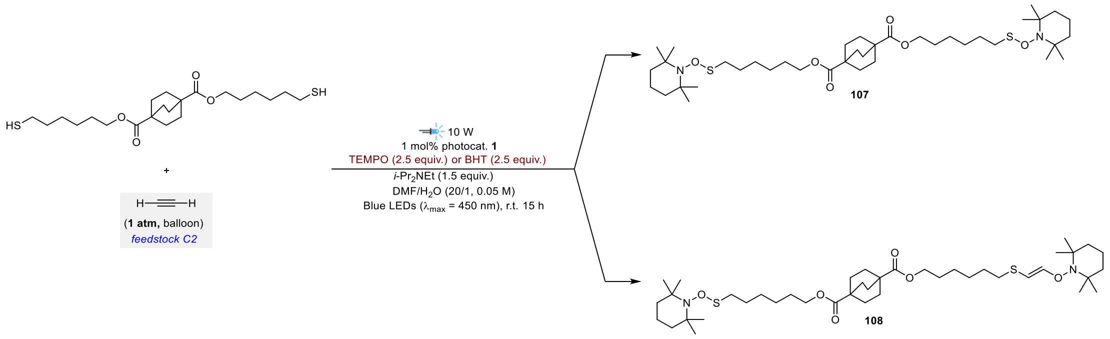

To a 10 mL Schlenk tube equipped with a magnetic stir bar was added bis(6-mercaptohexyl) bicyclo[2.2.2]octane-1,4-dicarboxylate (108 mg, 0.25 mmol, 1.0 equiv.), [Ir[d(*t*Bu)(CF_3)ppy]$_2$(dtbbpy)[Cl] (2.7 mg, 2.5 μmol, 0.01 equiv.), *N,N*-diisopropylethylamine (49 mg, 0.38 mmol, 1.5 equiv.), DMF (5 mL) and deionized water (100 μL) under acetylene gas. The tube was then sealed and was placed in the Parallel Light Reactor, which cooled with the recirculated cooling water. After 15 minutes, the radical trapper TEMPO or BHT (0.56 mmol, 2.5 equiv.) was added. The corresponding reaction mixture was detected according to HRMS analysis. It is noteworthy that BHT cannot effective suppression. However, in the presence of 2.5 eq. radical inhibitor, TEMPO, the reaction was not only inhibited but also radical coupling products detected by high-resolution mass spectrometry (**Supplementary Figure 7**), which means that radicals were involved in this transformation.

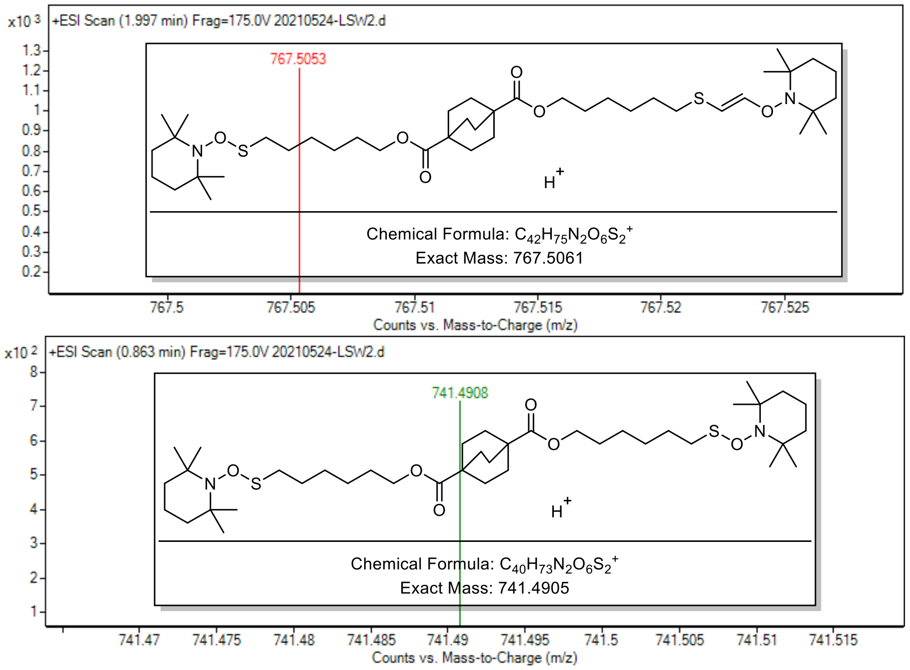

Supplementary Figure 7. High-resolution mass spectra of TEMPO adduct

2,2,6,6-Tetramethyl-1-piperinedinyloxy (TEMPO) capture DIPEA radical

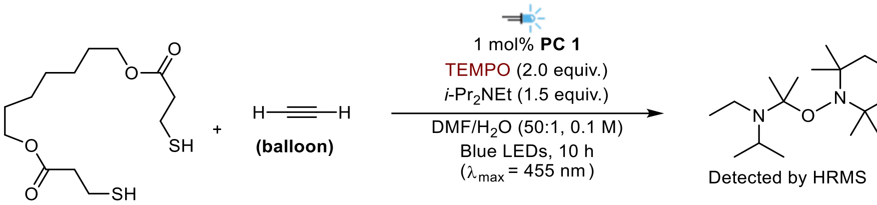

To a 10 mL Schlenk tube equipped with a magnetic stir bar was added heptane-1,7-diyl bis(3-mercaptopropanoate) (31.1 mg, 0.20 mmol, 1.0 equiv.), photocatalyst [Ir(d(*t*Bu)(CF_3)ppy)$_2$(dtbbpy)][Cl] (2.2 mg, 1 mol %), the tube was evacuated and backfilled with acetylene gas (three times). Subsequently, DMF/D_2O (2 mL, 50/1 = v/v) and *N*,*N*-diisopropylethylamine (39 mg, 0.30 mmol, 1.5 equiv.) were added by pipettor under acetylene gas. The tube was then sealed and was placed on blue LEDs lamp of Parallel Light Reactor (10 W, λ_{max} = 455 nm, tube about 2 cm away from lights). The mixture was stirred for 10 minutes at room temperature. Then, TEMPO (62.4 mg, 0.4 mmol, 2 equiv.) was added at acetylene atmosphere. After completion, the corresponding reaction mixture was detected by HRMS (**Supplementary Figure 14**). This result mean that the DIPEA as hydrogen donor involved in HAT procedure.

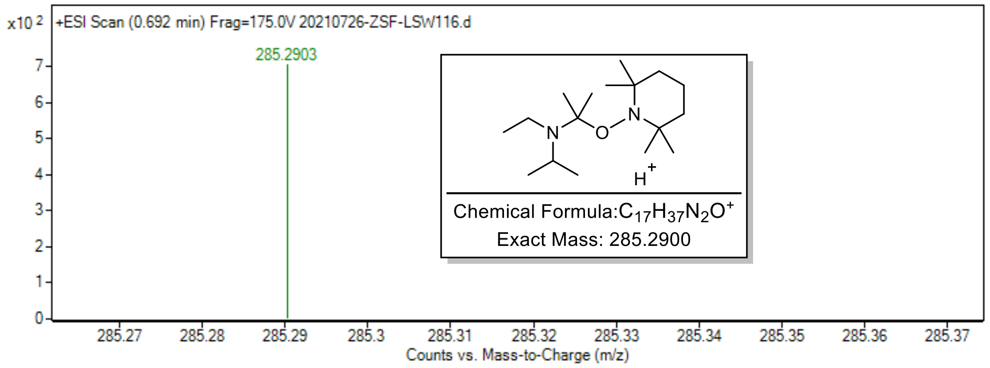

Supplementary Figure 14. High-resolution mass spectra of TEMPO adduct to DIPEA

7.2 Tracking of the reaction process

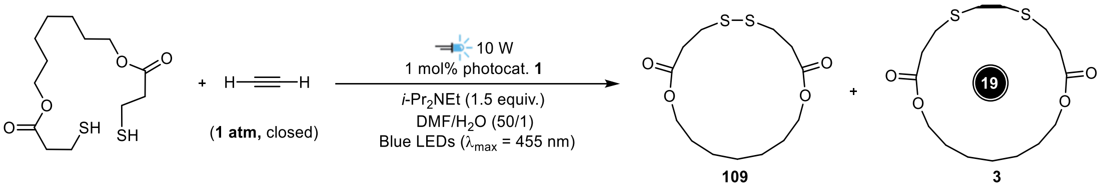

The photocatalyst [Ir[d(*t*Bu)(CF_3)ppy]$_2$(dtbbpy)[Cl] (5.4 mg, 5.0 μmol, 0.01 equiv.) was added to an oven-dried Schlenk tube containing a magnetic stirring bar. In the absence of blue light, hexane-1,6-dithiol (75mg, 0.5 mmol, 1.0 equiv.), DMF (10 mL), *N*,*N*-diisopropylethylamine (96 mg, 0.75 mmol, 1.5 equiv.) and deionized water (200 μL) were added under an argon stream. The resulting solution cooled to -78 °C was degassed using three freeze pump-thaw cycles and the tube was finally backfilled with acetylene gas. The reaction mixture was irradiated with blue LEDs (10 W, λ_{max} = 455 nm). At every point in time, the mixture was detected by GC-MS (**Supplementary Figure 8 and 9**). A time-course study showed that the cyclic disulfide intermediate was the key intermediate in our protocol.

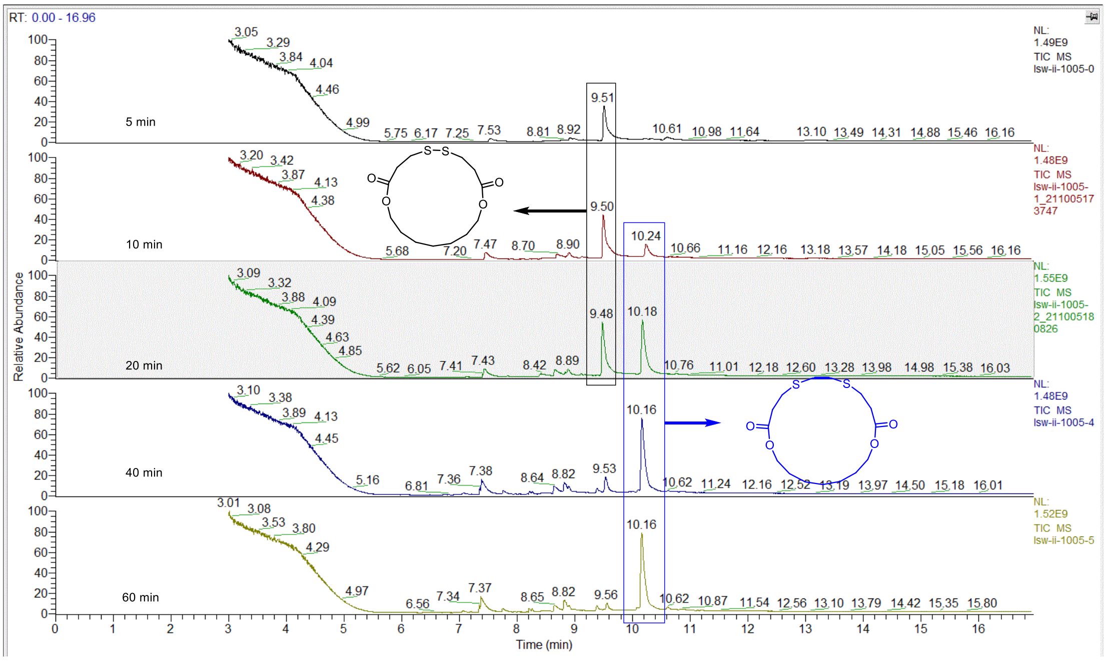

Supplementary Figure 8. Reaction progress monitoring the chemical intermediate versus reaction time via GC-MS

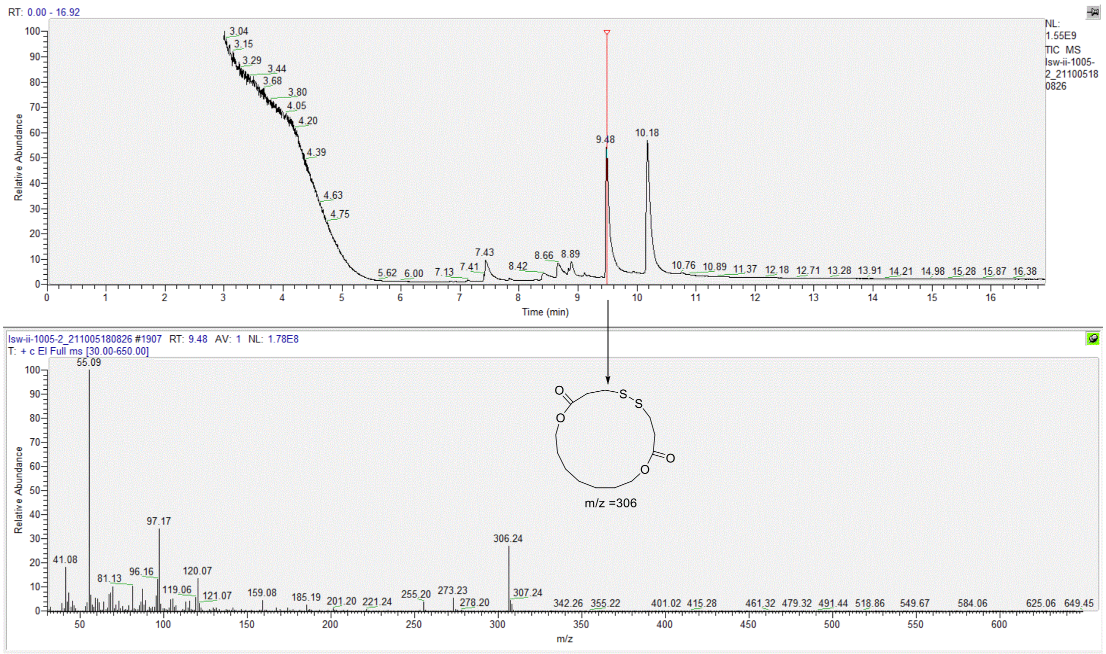

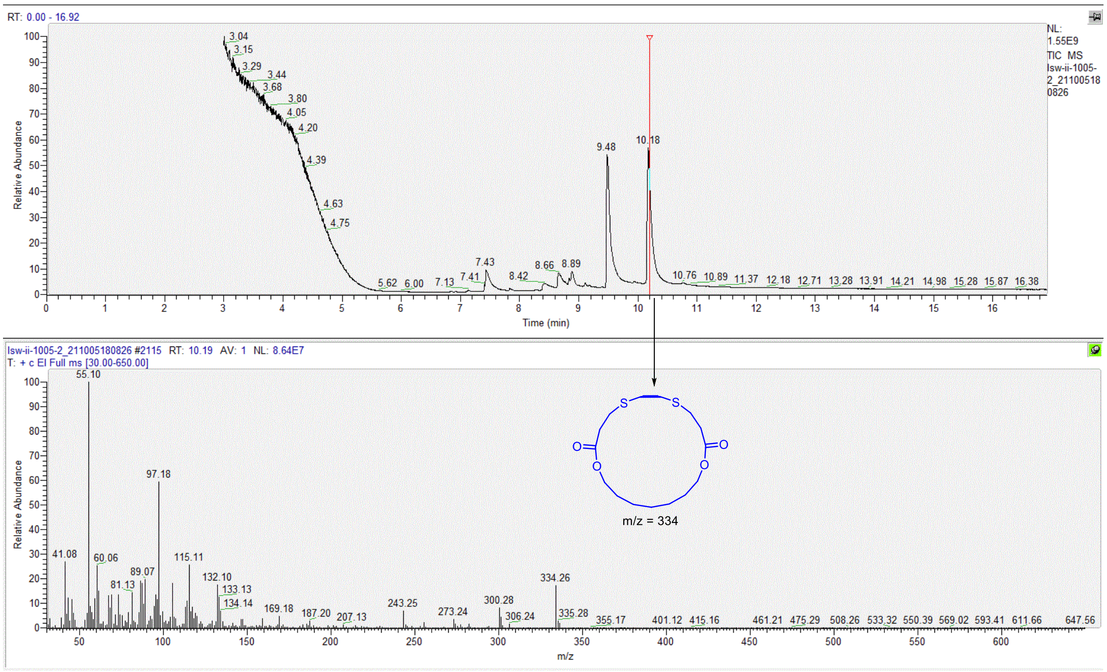

Supplementary Figure 9. The intermediate of this reaction detected by GC-MS

7.3 The preparation and transformation of reaction intermediates 109

7.3.1 The preparation of 1,10-dioxa-5,6-dithiacycloheptadecane-2,9-dione **109**

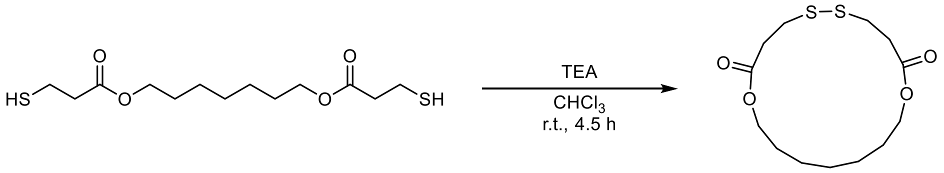

109, 62%

According to the literature[17], to a round-bottom flask equipped with a stirring bar was added triethylamine (425 mg, 4.2 mmol, 2.1 equiv.) in chloroform (15 mL). To another round-bottom flask equipped with a stirring bar was added heptane-1,7-diyl bis(3-mercaptopropanoate) (616 mg, 2.0 mmol) in chloroform (45 mL) and iodine (507 mg, 2.0 mmol, 1.0 equiv.) in chloroform (15 mL) was added separately. The resulting mixture was added to a another vigorously stirred of triethylamine solution over 4 h at room temperature. The rate of addition was adjusted so that the reaction mixture is colorless or slightly yellow so as to maintain the reaction in nearly equal concentration. There remains some iodine solution after all the dithiol is added which contains (125 mg, 0.5 mmol) of iodine. The reaction mixture was washed successively with water (25 mL) containing a few crystals of sodium thiosulfate, 0.1 normal hydrochloric acid (15 mL), and water (20 mL). The solution was dried over anhydrous sodium sulfate and evaporated to provide a faint yellow oil. The crude product was purified by column chromatography (Eluent: pentane: EtOAc = 10:1) to afford the title compound **74** as a faint yellow liquid (379 mg, 62%).

1,10-Dioxa-5,6-dithiacycloheptadecane-2,9-dione (109)

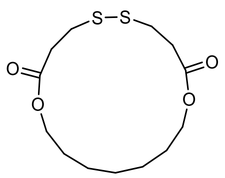

^{1}**H NMR** (500 MHz, $CDCl_3$) δ 4.16 (t, *J* = 6.0 Hz, 4H), 3.01 (t, *J* = 7.0 Hz, 4H), 2.70 (t, *J* = 7.0 Hz, 4H), 1.73 – 1.57 (m, 4H), 1.41 (ddd, J = 24.7, 12.2, 6.0 Hz, 6H); ^{13}**C NMR** (126 MHz, $CDCl_3$) δ 171.5, 64.4, 34.8, 34.4, 27.4, 26.7, 24.6; **IR** (KBr, cm^{-1}) 3168, 29618, 1734, 1401, 1129, 668; **HRMS** (ESI) calcd. For $C_{13}H_{22}NaO_4S_2^+$ $[M+Na]^+$ m/z 329.0852, found: 329.0845.

7.3.2 The reaction of 1,10-dioxa-5,6-dithiacycloheptadecane-2,9-dione **109** with acetylene under standard conditions

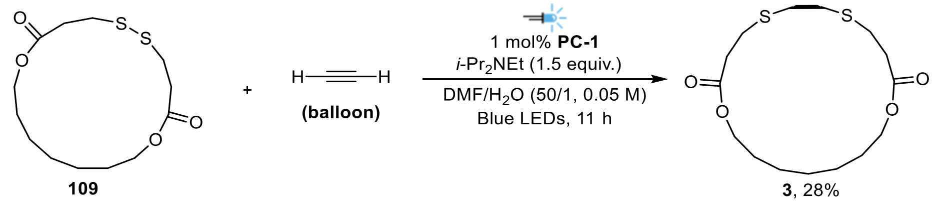

The intermediate 1,10-dioxa-5,6-dithiacycloheptadecane-2,9-dione **109** was resubjected to the standard conditions. The desired two directional hydrofunctionalization of acetylene product was detected by gas chromatography-mass spectrometry, suggesting that the disulfide is a putative intermediate in the sequential hydrofunctionalization process.

7.4 The synthesis of DIPEA analogues deuterated at different specific positions

7.4.1 The preparation of *N*-Octyl-*N*-(propan-2-yl-2-*d*)acetamide **110**

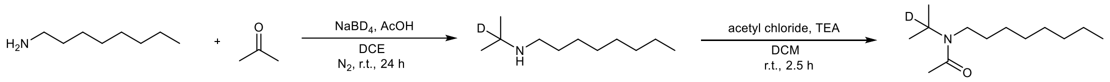

Step1: On the basis of a literature report[18], an oven-dried screw-capped Schlenk tube equipped with a Teflon-coated magnetic stir bar was added the corresponding $NaBD_4$ (628 mg, 15 mmol, 3 equiv.), 1,2-dichloroethane (15 mL) and AcOH (2.70 g, 45 mmol, 3.0 equiv.). The tube was evacuated and filled with nitrogen (three times). 1-octylamine (646 mg, 5.0 mmol, 1 equiv.), acetone (580 mg, 10 mmol, 2 equiv.) and AcOH (600 mg, 10 mmol, 2 equiv.) in 1,2-dichloroethane (5.0 mL) in one portion were added afterwards and the resulting mixture was stirred at room temperature for 5 min. (**Caution!** Rapid evolution of gas, vigorously stirred of solution and slow added were needed). The vial was then sealed, and the resulting mixture was stirred at room temperature for 24 h under a nitrogen atmosphere. After the reaction mixture was quenched by adding 1 N NaOH aqueous solution, extracted with Et_2O, washed with brine, dried over $MgSO_4$, filtered and concentrated under reduced pressure. The corresponding *N*-(propan-2-yl-2-*d*)octan-1-amine was directly used in the next step without further purification.

Step2: A oven-dried 50 mL round bottom flask was charged sequentially with a stir bar, *N*-(propan-2-yl-2-*d*)octan-1-amine, anhydrous DCM (5.0 mL) and TEA (505 mg, 5 mmol, 1 equiv.) under atmospheric environment. Acetyl chloride (392 mg, 5 mmol, 1 equiv.) was added dropwise over 2 min via syringe to the solution and the mixture was allowed to stir for 2.5 h at room temperature. The reaction mixture was diluted with DCM (15 mL), washed with 1 N HCl aqueous solution,

extracted with DCM, dried over Na_2SO_4, filtered and concentrated under reduced pressure. Purification by column chromatography (Eluent: petroleum ether/EtOAc = 2/1) afforded the title compound as a colourless oil (706 mg, 66% yield).

***N*-Octyl-*N*-(propan-2-yl-2-*d*)acetamide**

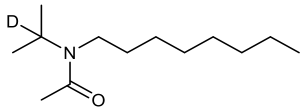

^{1}H NMR (500 MHz, Chloroform-*d*) δ 3.18 – 3.03 (m, 2H), 2.09 (d, J = 11.0 Hz, 3H), 1.64 – 1.47 (m, 2H), 1.28 (s, 10H), 1.18 (s, 3H), 1.13 (s, 1H), 0.88 (q, J = 6.9 Hz, 3H); **^{13}C NMR** (126 MHz, $CDCl_3$) δ 170.2, 169.6, 49.1, 49.0, 48.9, 48.7, 45.3, 45.1, 44.7, 44.2, 40.9, 31.8, 31.7, 31.2, 29.6, 29.3, 29.3, 29.2, 29.2, 27.4, 27.2, 22.6, 22.6, 22.1, 22.1, 21.1, 20.4, 14.1, 14.0; **IR** (KBr, cm^{-1}) 3852, 3449, 1641, 1389, 1108; **HRMS** (ESI) calcd. For $C_{13}H_{27}DNO^+$ $[M+H]^+$ m/z 215.2228, found: 215.2231.

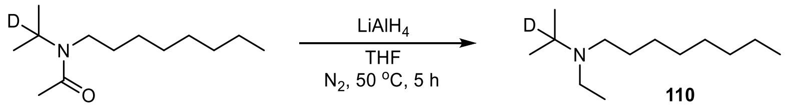

Step3: According to the reported procedure with slight modification[13], an oven-dried screw-capped Schlenk tube equipped with a Teflon-coated magnetic stir bar was added the corresponding *N*-octyl-*N*-(propan-2-yl-2-*d*) acetamide (643 mg, 3 mmol, 1.0 equiv.) and anhydrous THF (5 mL) under a N_2 atmosphere. Next, $LiAlH_4$ (136 mg, 3.6 mmol, 1.2 equiv.) was added as a solid in four portions. The mixture was heated for 5 h. With cooling and stirring, an aqueous 10% NaOH solution was added and the ether layer was extracted, dried over anhydrous Na_2SO_4, filtered and concentrated under reduced pressure. Purification by flash column chromatography provided the *N*-ethyl-*N*-(propan-2-yl-2-*d*)octan-1-amine as a yellow oil (565 mg, 94%).

***N*-Ethyl-*N*-(propan-2-yl-2-*d*)octan-1-amine (110)**

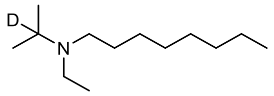

^{1}H NMR (500 MHz, Chloroform-*d*) δ 2.46 (q, J = 7.2 Hz, 2H), 2.39 – 2.31 (m, 2H), 1.42 (p, J = 7.1, 6.5 Hz, 2H), 1.28 (s, 10H), 1.02 (t, J = 7.2 Hz, 3H), 0.97 (s, 6H), 0.88 (t, J = 6.8 Hz, 3H); **^{13}C NMR** (126 MHz, $CDCl_3$) δ 49.7, 43.8, 31.9, 29.6, 29.3, 29.1, 27.7, 22.7, 18.2, 14.1, 14.0; **IR** (KBr, cm^{-1}) 3741, 3619, 3165, 1396, 1384, 1110; **HRMS** (ESI) calcd. For $C_{13}H_{29}DNO^+$ $[M+H]^+$ m/z 201.2436, found: 201.2535.

7.4.2 The preparation of *N*-ethyl-*N*-(propan-2-yl-*d*$_7$)octan-1-amine **111**

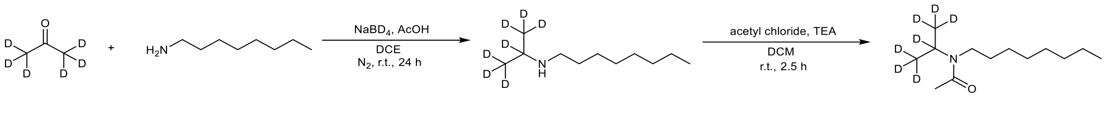

Step1: An oven-dried screw-capped Schlenk tube equipped with a Teflon-coated magnetic stir bar was added the corresponding $NaBD_4$ (628 mg, 15 mmol, 3 equiv.), 1,2-dichloroethane (15 mL) and AcOH (2.70 g, 45 mmol, 3.0 equiv.). The tube was evacuated and filled with nitrogen (three times). 1-octylamine (646 mg, 5.00 mmol, 1 equiv.), propan-2-one-*d*$_6$ (641 mg, 10 mmol, 2 equiv.) and AcOH (600 mg, 10 mmol, 2 equiv.) in 1,2-dichloroethane (5.0 mL) in one portion were added

afterwards and the resulting mixture was stirred at room temperature for 5 min. (**Caution!** Rapid evolution of gas, vigorously stirred of solution and slow added were needed). The vial was then sealed, and the resulting mixture was stirred at room temperature for 24 h under a nitrogen atmosphere. After the reaction mixture was quenched by adding 1 N NaOH aqueous solution, extracted with Et_2O, washed with brine, dried over $MgSO_4$, filtered and concentrated under reduced pressure. The corresponding *N*-(propan-2-yl-d_7)octan-1-amine was directly used in the next step without further purification.

Step2: A oven-dried 50 mL round bottom flask was charged sequentially with a stir bar, *N*-(propan-2-yl-d_7)octan-1-amine, anhydrous DCM (5.0 mL) and TEA (505 mg, 5 mmol, 1 equiv.) under atmospheric environment. Acetyl chloride (392 mg, 5 mmol, 1 equiv.) was added dropwise over 2 min via syringe to the solution and the mixture was allowed to stir for 2.5 h at room temperature. The reaction mixture was diluted with DCM (15 mL), washed with 1 N HCl aqueous solution, extracted with DCM, dried over Na_2SO_4, filtered and concentrated under reduced pressure. Purification by column chromatography (Eluent: petroleum ether/EtOAc = 2/1) afforded the title compound as a a light oil (694 mg, 63% yield).

***N*-octyl-*N*-(propan-2-yl-d_7)acetamide**

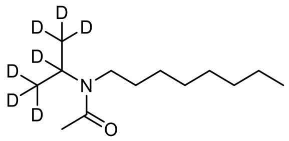

^{1}H NMR (500 MHz, Chloroform-*d*) δ 3.12 (dt, *J* = 14.8, 8.5 Hz, 2H), 2.28 – 1.93 (m, 3H), 1.55 (td, *J* = 9.4, 8.9, 4.8 Hz, 2H), 1.29 (s, 10H), 0.88 (dt, *J* = 7.0, 3.3 Hz, 3H); **^{13}C NMR** (126 MHz, $CDCl_3$) δ 170.2, 169.7, 44.2, 40.9, 31.8, 31.7, 31.2, 29.5, 29.3, 29.2, 29.2, 29.1, 27.4, 27.2, 22.6, 22.5, 22.0, 22.0, 14.0, 14.0; **IR** (KBr, cm^{-1}) 3748, 3445, 2924, 1647, 1397, 1384, 1110; **HRMS** (ESI) calcd. For $C_{13}H_{20}NaD_7NO^+$ $[M+Na]^+$ m/z 243.2424, found: 243.2416.

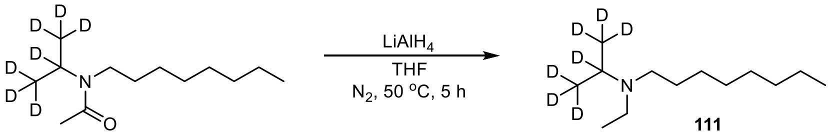

Step3: An oven-dried screw-capped Schlenk tube equipped with a Teflon-coated magnetic stir bar was added the corresponding *N*-octyl-*N*-(propan-2-yl-d_7)acetamid*e* (330 mg, 3.0 mmol, 1.0 equiv.) and anhydrous THF (6 mL) under a N_2 atmosphere. Next, $LiAlH_4$ (148 mg, 3.9 mmol, 1.3 equiv.) was added as a solid in four portions. The mixture was heated for 5 h. With cooling and stirring, an aqueous 10% NaOH solution was added and the ether layer was extracted, dried over anhydrous Na_2SO_4, filtered and concentrated under reduced pressure. Purification by column chromatography (Eluent: $CHCl_3$/MeOH = 4/1) afforded the title compound as a pale-yellow oil (594 mg, 96% yield).

***N*-ethyl-*N*-(propan-2-yl-d_7)octan-1-amine (111)**

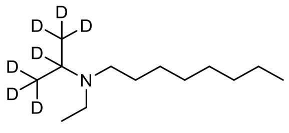

^{1}H NMR (500 MHz, Chloroform-*d*) δ 2.46 (q, *J* = 7.2 Hz, 2H), 2.35 (dd, *J* = 8.9, 6.8 Hz, 2H), 1.42 (p, *J* = 7.1 Hz, 2H), 1.28 (s, 10H), 1.02 (t, *J* = 7.3 Hz, 3H), 0.88 (t, *J* = 6.8 Hz, 3H); **^{13}C NMR** (126

MHz, $CDCl_3$) δ 49.9, 49.7, 49.6, 49.3, 49.2, 43.8 31.8, 29.6, 29.3, 29.0, 27.7, 22.7, 18.2, 18.0, 17.8, 17.6, 17.54, 17.1, 14.1, 14.0; **IR** (KBr, cm^{-1}) 3749, 3168, 2926, 1647, 1397, 1384, 1110; **HRMS** (ESI) calcd. For $C_{13}H_{23}D_7N^+$ $[M+H]^+$ m/z 207.2812, found: 207.2810.

7.4.3 The preparation of *N*,*N*-Diisopropylnonan-1-amine-1,1-d_2 **112**

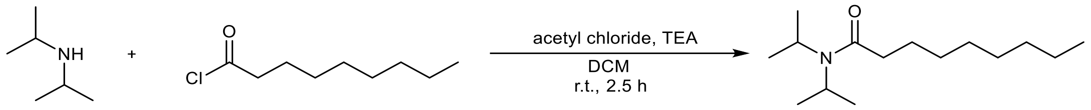

Step1: An oven-dried 50 mL round bottom flask was charged sequentially with a stir bar, diisopropylamine (501 mg, 5 mmol, 1.0 equiv.), anhydrous DCM (5.0 mL) and TEA (606 mg, 6 mmol, 1.2 equiv.) under atmospheric environment. Nonanoyl chloride (501 mg, 5 mmol, 1.0 equiv.) was added dropwise over 2 min via syringe to the solution and the mixture was allowed to stir for 2.5 h at room temperature. The reaction mixture was diluted with DCM (15 mL), washed with 1 N HCl aqueous solution, extracted with DCM, dried over Na_2SO_4, filtered and concentrated under reduced pressure. Purification by flash column chromatography (Eluent: petroleum ether/EtOAc = 10/1) provided the *N*,*N*-diisopropylnonanamide as a faint yellow oil (1.05 g, 87%).

***N*,*N*-Diisopropylnonanamide**

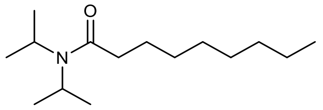

^{1}H NMR (500 MHz, Chloroform-*d*) δ 3.96 (q, *J* = 6.8 Hz, 1H), 3.48 (s, 1H), 2.38 – 2.19 (m, 2H), 1.61 (p, *J* = 7.4 Hz, 2H), 1.37 (d, *J* = 6.8 Hz, 6H), 1.29 (dq, *J* = 17.4, 5.6, 5.2 Hz, 10H), 1.20 (d, *J* = 6.8 Hz, 6H), 0.88 (t, *J* = 6.8 Hz, 3H); **^{13}C NMR** (126 MHz, $CDCl_3$) δ 172.1, 48.2, 45.5, 35.4, 31.8, 29.5, 29.4, 29.2, 25.5, 22.6, 21.0, 20.7, 14.1; **IR** (KBr, cm^{-1}) 3730, 3622, 3166, 1653, 139. 1384, 1111, 422; **HRMS** (ESI) calcd. For $C_{15}H_{32}NO^+$ $[M+H]^+$ m/z 242.2478, found: 242.2472.

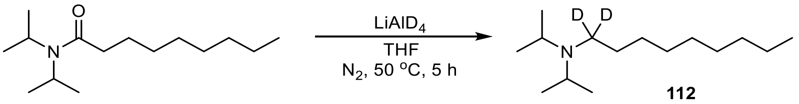

112

Step2: An oven-dried screw-capped Schlenk tube equipped with a Teflon-coated magnetic stir bar was added the corresponding *N*,*N*-diisopropylnonan-1-amine-1,1-d_2 (966 mg, 4 mmol, 1.0 equiv.) and anhydrous THF (5 mL) under a N_2 atmosphere. Next, $LiAlD_4$ (200 mg, 4.8 mmol, 1.2 equiv.) was added as a solid in four portions. The mixture was heated for 5 h. With cooling and stirring, an aqueous 10% NaOH solution was added and the ether layer was extracted, dried over anhydrous Na_2SO_4, filtered and concentrated under reduced pressure. Purification by flash column chromatography provided the *N*,*N*-diisopropylnonan-1-amine-1,1-d_2 as a yellow oil (870 mg, 95%).

***N*,*N*-Diisopropylnonan-1-amine-1,1-d_2 (112)**

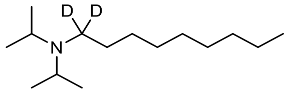

^{1}H NMR (500 MHz, Chloroform-*d*) δ 3.00 (p, *J* = 6.5 Hz, 2H), 1.33 – 1.18 (m, 12H), 1.00 (d, *J* = 6.6 Hz, 12H), 0.88 (t, *J* = 6.9 Hz, 3H); **^{13}C NMR** (126 MHz, $CDCl_3$) δ 48.6, 31.9, 31.5, 29.7, 29.7, 29.3, 27.5, 22.7, 20.6, 14.1; **HRMS** (ESI) calcd. For $C_{13}H_{32}D_7N^+$ $[M+H]^+$ m/z 230.2811, found: 2302837.

7.5 Thiol-yne click chemistry of acetylene-enabled macrocyclization using DIPEA analogues deuterated

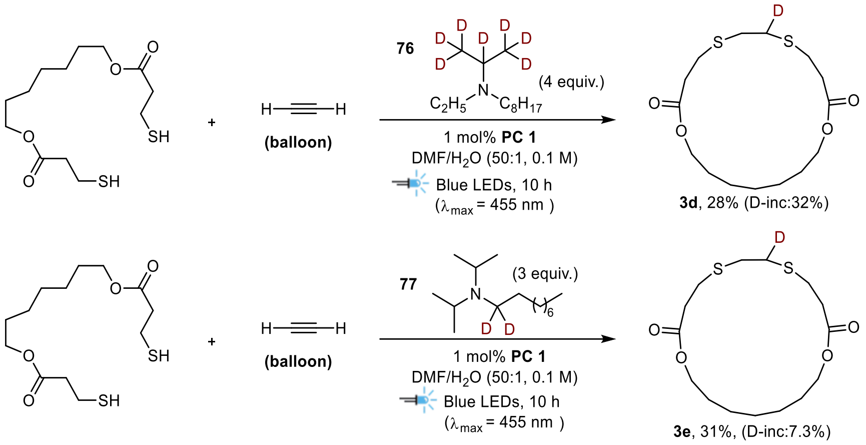

Following the general procedure **A** and similar to the preparation of **3i** while using deuterated bases (*N*-ethyl-*N*-(propan-2-yl-d_7)octan-1-amine **76** and *N*,*N'*-diisopropylnonan-1-amine-1,1-d_2 **77** as organic base, respectively).

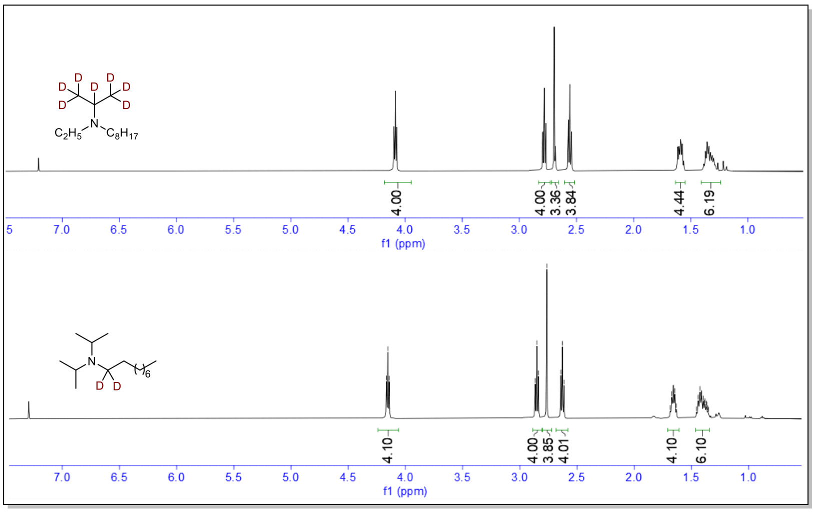

Supplementary Figure 12. ^{1}H NMR spectrum of the corresponding product **3d** and **3e**

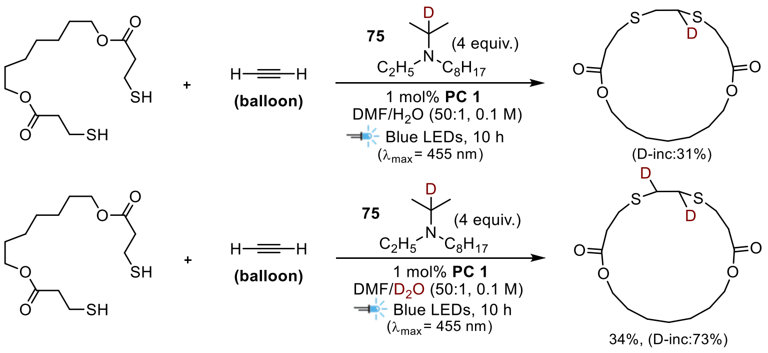

When *N*-ethyl-*N*-(propan-2-yl-d_7)octan-1-amine was used, ^{1}H NMR analysis of the 19-membered ring product revealed that 32% deuterium scrambling and deuterium incorporated into the two directional hydrofunctionalized product. In contrast, if *N*,*N*-diisopropylnonan-1-amine-1,1-d_2 are used for the reaction, ^{1}H NMR analysis of the desired 19-membered ring product revealed that only

a small amount of deuterium scrambling and deuterium incorporated at S-CH_2-CH_2-S setting. Whereas 31% total deuterium transfer was observed in the initial deuterium labeling experiment, 73% total deuterium transfer was observed in a double labeling crossover experiment, which is mean that hydrogen source of two directional hydrofunctionalization of acetylene mainly from DIPEA and H_2O.

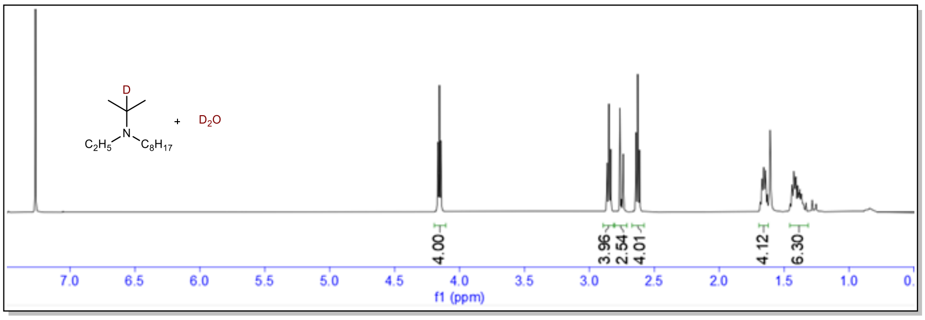

Supplementary Figure 13. ^{1}H NMR spectrum of the corresponding product **3f**

7.6 Isotope labeling experiment

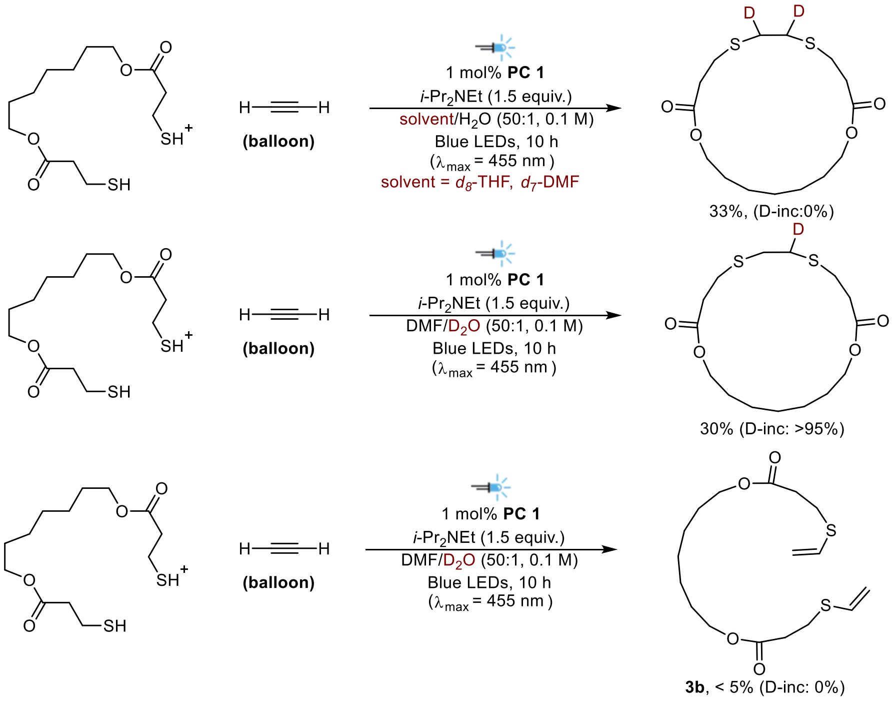

Following the general procedure **A** and similar to the preparation of **3i** while using deuterated reagents (d_8-THF, d_7-DMFand D_2O) as solvents. The results indicate that When d_8-THF or 1.0 mL d_7-DMF was used, respectively, ^{1}H NMR analysis revealed that no deuterium incorporation in the desired 19-membered ring product. When D_2O was used, ^{1}H NMR analysis of the 19-membered ring product revealed that >95% deuterium scrambling and deuterium incorporated into the two directional hydrofunctionalized product. The non-deuterium linear vinyl thioether **3b** was formed in low yield (< 5%), so we only obtained the hydrogen spectrum. The results of **Supplementary Figure 10** and **11** unambiguously depicted that polar protic solvent water was responsible for one hydrogen atom of the two new forming C-H bonds instead of organic solvent (d_7-DMF and d_8-THF).

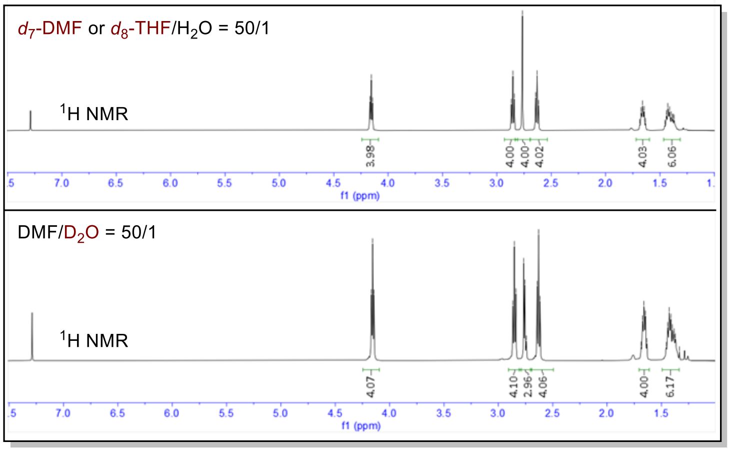

Supplementary Figure 10. ^{1}H NMR spectrums of **3** and **3a**

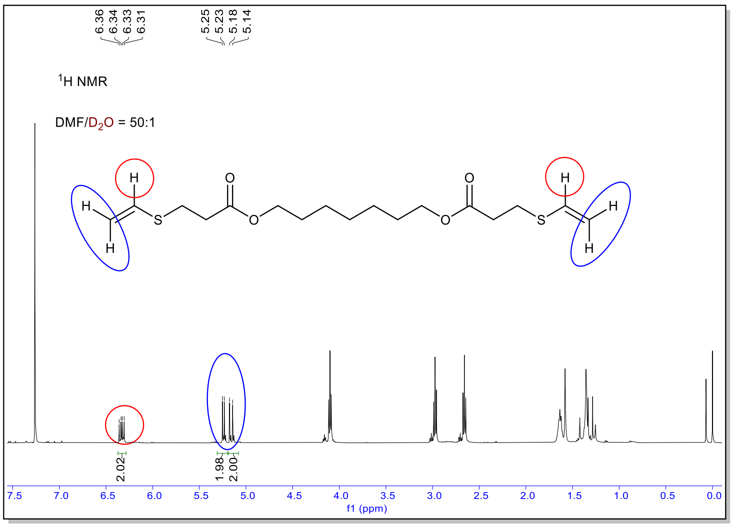

Supplementary Figure 11. ^{1}H NMR spectrum of the **3b**

7.7 The Source of α-Olefinic Hydrogen for vinyl thioethers

Noteworthy, in the process of evaluate the scope of this protocol, the electron-deficient aryl thiols linear precursors, the reaction selected stop at the stage of semihydrogenation, did not produce desired macrocyclization. Presumably because of the mismatch of electronic polarity for the reaction of electrophilic R-S• radicals with the electron-deficient vinyl thiolether[19]. This result provides favorable conditions for our follow-up mechanism research. To identify the source of α-olefinic hydrogen of vinyl thioether, two parallel reactions were conducted. Respectively, no incorporation of deuterium was found for the semihydrothiolation, which strongly supported the mechanism that the D-incorporation exclusively occurred during the second hydrothiolation process.

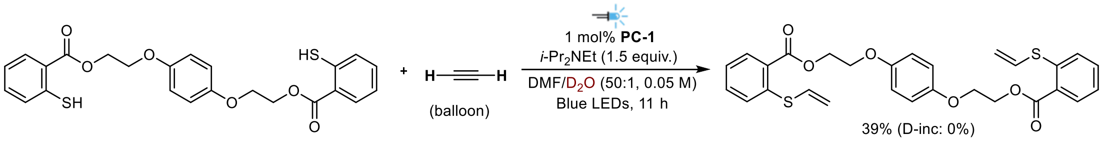

Prepared following the general procedure outlined above using [Ir[d(*t*Bu)(CF_3)ppy]$_2$(dtbbpy)[Cl] (2.7 mg, 2.5 μmol, 0.01 equiv.), (1,4-phenylenebis(oxy))bis(ethane-2,1-diyl) bis(2-mercaptobenzoate) (118 mg, 0.25 mmol, 1 equiv.), DMF (5 mL), *N,N*-diisopropylethylamine (49

mg, 0.38 mmol, 1.5 equiv.) and deionized water (100 μL). Purification by column chromatography (Eluent: petroleum ether/EtOAc = 3/1) afforded the title compound as a white solid (51 mg, 39% yield).

(1,4-Phenylenebis(oxy))bis(ethane-2,1-diyl) bis(2-((-vinyl-2-d)thio)benzoate) (52b)

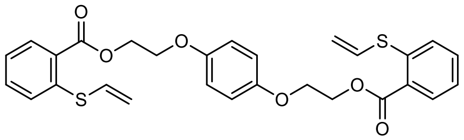

^{1}H NMR (500 MHz, $CDCl_3$) δ 8.10 – 7.89 (m, 2H), 7.50 – 7.40 (m, 2H), 7.37 (d, *J* = 7.9 Hz, 2H), 7.20 (td, *J* = 7.6, 1.3 Hz, 2H), 6.88 (s, 4H), 6.58 (dd, *J* = 16.7, 9.4 Hz, 2H), 5.72 (d, *J* = 16.7 Hz, 2H), 5.61 (d, *J* = 9.4 Hz, 2H), 4.80 – 4.56 (m, 4H), 4.43 – 4.21 (m, 4H); **^{13}C NMR** (126 MHz, $CDCl_3$) δ 166.1, 153.1, 140.5, 132.6, 131.4, 130.2, 127.6, 127.5, 125.0, 121.5, 115.9, 66.7, 63.7; **Mp**: 83.1 – 95.7 °C; **IR** (KBr, cm^{-1}) 3296, 2874, 1728, 1586; **HRMS** (ESI) calcd. For $C_{28}H_{26}NaO_6S_2^+$ $[M+Na]^+$ m/z 545.1063, found: 545.1063.

7.8 Trapping of carbanion experiments

7.8.1 Using 2,3,4,5,6-pentafluorobenzaldehyde to capture carbanion intermediate

Trapped with others electrophilic reagents. To support the formation of carbanion intermediate, electrophilic reagent, 2,3,4,5,6-pentafluorobenzaldehyde, was added instead of H_2O under standard conditions. The addition product detected by high-resolution mass spectrometry, which means that the reaction was mediated by a carbon anion intermediate.

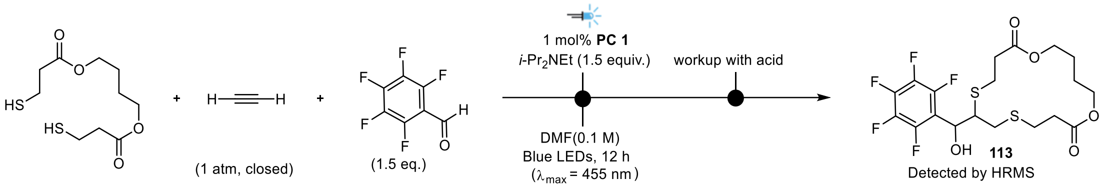

To a 10 mL Schlenk tube equipped with a magnetic stir bar was added heptane-1,7-diyl bis(3-mercaptopropanoate) (31.1 mg, 0.20 mmol, 1.0 equiv.), photocatalyst [Ir(d(*t*Bu)(CF_3)ppy)$_2$(dtbbpy)][Cl] (2.2 mg, 1 mol %), the tube was evacuated and backfilled with acetylene gas (three times). Subsequently, DMF (2 mL), *N*,*N*-diisopropylethylamine (39 mg, 0.30 mmol, 1.5 equiv.) and 2,3,4,5,6-pentafluorobenzaldehyde (59 mg, 0.30 mmol, 1.5 equiv.) were added by pipettor under acetylene gas. The tube was then sealed and was placed in Parallel Light Reactor The tube was placed in the Parallel Light Reactor, which was cooled with the recirculated cooling water. After completion, the resulting mixture was diluted with ethyl acetate (2 mL) and quenched by 1.5 N HCl (2 mL), then stirred for 10 min. Subsequently, the corresponding reaction mixture was directly detected by HRMS without further purification (**Supplementary Figure 15**).

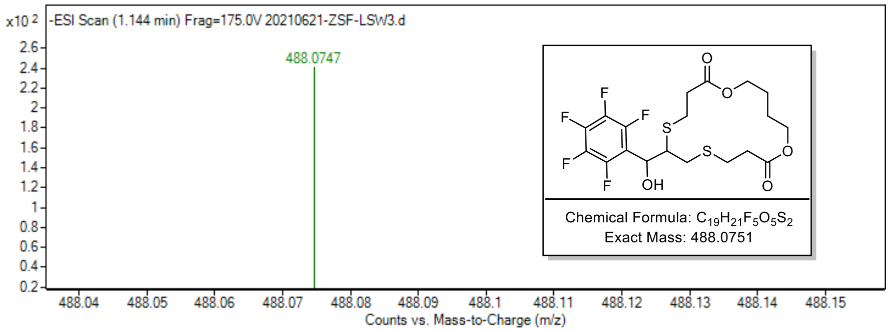

Supplementary Figure 15. High-resolution mass spectra of carbon anion intermediate trapped by 2,3,4,5,6-pentafluorobenzaldehyde

7.8.2 The capture of carbanion intermediate using CO_2

On the basis of this success, we further considered whether other electrophiles, especially thermodynamic stability and kinetic inertness more stable carbon dioxide, could be captured in such catalytic system[20]. The photocatalyst [Ir(d(*t*Bu)(CF$_3$)ppy)$_2$(dtbbpy)][Cl] (2.2 mg, 0.005 mmol, 1 mol %) was added to an oven-dried Schlenk tube containing a magnetic stirring bar. In the absence of blue light, butane-1,4-diyl bis(3-mercaptopropanoate) (53 mg, 0.20 mmol, 1.0 equiv.), *N*,*N*-dimethylformamide (2 mL), *N*,*N*-diisopropylethylamine (0.30 mmol, 1.5 equiv.) were added via pipette (10 ~ 100 μL) under an argon stream. The resulting solution was degassed using three freeze pump-thaw cycles and the tube was finally backfilled with nitrogen. Subsequently, two balloons, connected to 5 mL plastic syringe head, were filled with carbon dioxide and acetylene gas until its size was roughly 10 cm in diameter, respectively. Two balloons equipped with needles were inserted concurrent through a rubber plug to the reaction system. The solution was then bubbled gases for 3 minutes, after which the tube was sealed to maintain the acetylene and carbon dioxide atmosphere throughout the tube. Finally, the reaction tube was placed in the Parallel Light Reactor cooled with the recirculated cooling water for 12 hours. The resulting mixture was diluted with ethyl acetate (2 mL) and quenched by 1.5 N HCl (2 mL), then stirred for 10 min. Subsequently, the corresponding reaction mixture was directly detected by HRMS (**Supplementary Figure 16**) without further workup. The carboxylation product detected by high-resolution mass spectrometry, which further elucidated that this transformation involved a carbon anion intermediate.

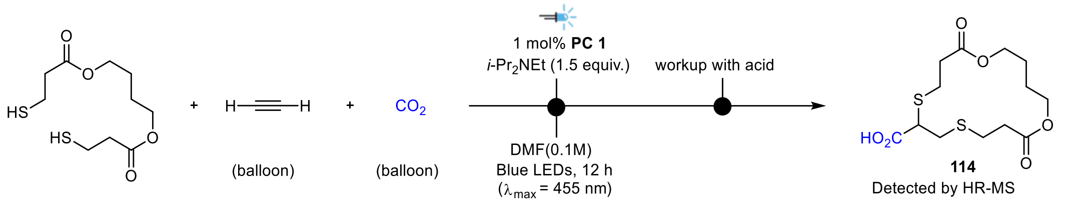

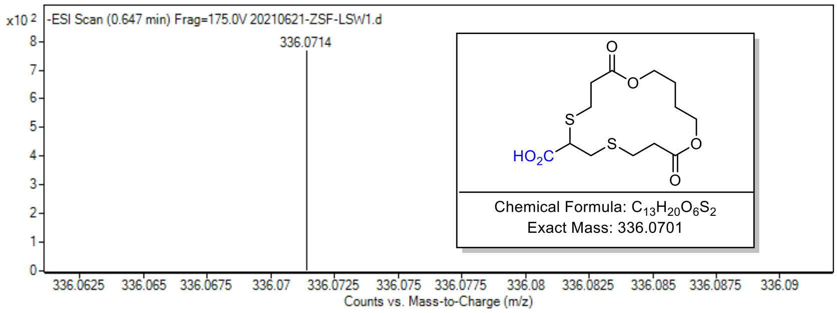

Supplementary Figure 16. High-resolution mass spectra of carbon anion intermediate trapped by CO_2

7.9 UV/Vis Absorption Spectroscopy

UV–Vis absorption spectra were recorded on a Jasco V-730 spectrophotometer at 25 °C. The samples were measured in Starna® fluorescence quartz cuvettes (type: 29-F, chamber volume = 1.400 mL, H × W × D = 48 mm × 12.5 mm × 12.5 mm, path length = 10 mm).

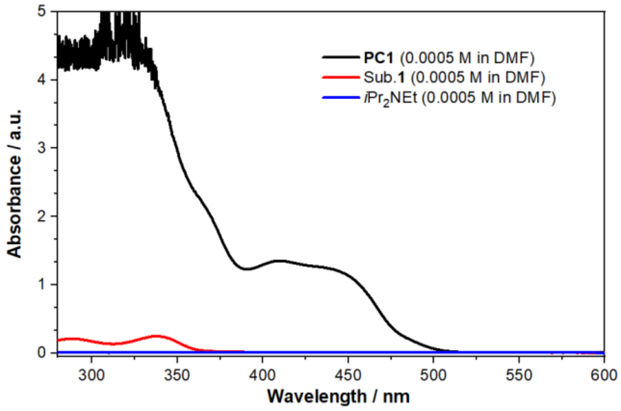

Supplementary Figure 17. UV–Vis absorption spectra of the starting materials.

Supplementary Figure 17 reveal that the Ir[*t*Bu(CF$_3$)ppy]$_2$(dtbbpy)Cl is the only species, which absorbs light at around λ = 450 nm (intensity maximum of the used blue LEDs λ_{max} = 455 nm), implying that no other species is directly excited by the irradiation under the reaction conditions.

7.10 Stern–Volmer Luminescence Quenching Analysis

Steady-state emission spectra were acquired using a Hitachi F-4500 fluorescence spectrophotometers, equipped with a temperature control unit at 25 °C. In a typical experiment, a solution of photocatalyst Ir[*t*Bu(CF$_3$)ppy]$_2$(dtbbpy)[Cl] (6.5 mg) in degassed DMF (5 mL) was added with an appropriate amount of quencher in a quartz cuvette. Then the emission of the sample was collected. The results are listed below:

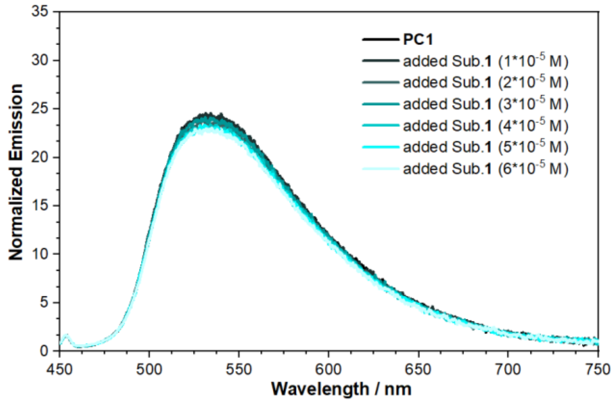

Supplementary Figure 18. Luminescence quenching of **PC1** with various concentrations of Sub.**1**.

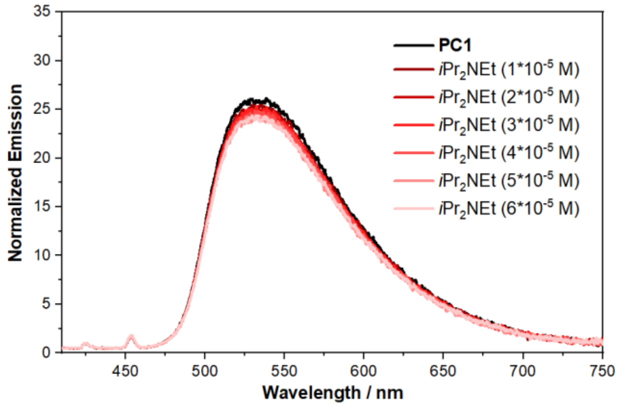

Supplementary Figure 19. Luminescence quenching of **PC1** with various concentrations of *i*Pr$_2$NEt.

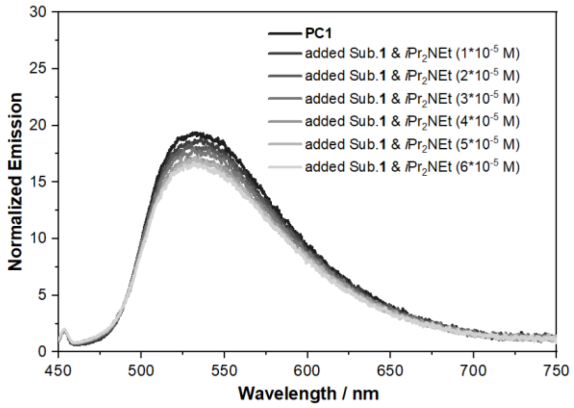

Supplementary Figure 20. Luminescence quenching of **PC1** with various concentrations of iPr$_2$NEt & Sub.**1**.

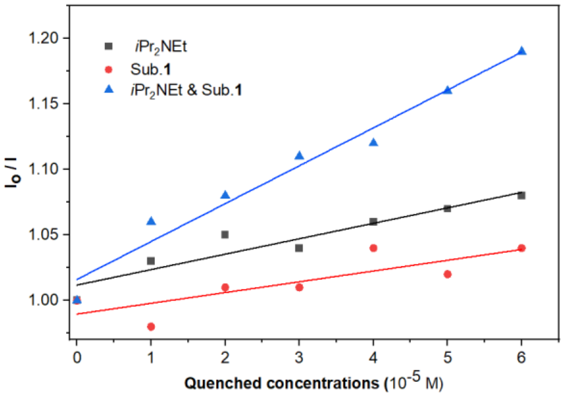

Supplementary Figure 21. Overlay of the quenching experiments.

Stern–Volmer fluorescence quenching analysis suggested that photoexcited *Ir(II) was quenched by Sub.1, iPr$_2$NEt and the solution of Sub.**1** with iPr$_2$NEt. However, consideration of the Stern–Volmer constants suggests that quenching of the excited photocatalyst by the complex of Sub.1 and iPr$_2$NEt is most efficient quenching agent compared with others. Based on this experiment and the quenching data, the solution of Sub.**1** and iPr$_2$NEt is the most likely quencher of *Ir[tBu(CF$_3$)ppy]$_2$(dtbbpy)[Cl]. Similar mechanism has also been proposed by MacMillan and coworkers[21].

8. X-ray structure analysis

Single crystals for X-ray studies were grown by slow evaporation of a solution of cyclic compound in a mixture of ethyl acetate and hexane at room temperature. Compounds were collected at 100 K on a Rigaku Oxford Diffraction Supernova Dual Source, Cu at Zero equipped with an AtlasS2 CCD using Cu Kα radiation.

The X-ray data of **4** is deposited in the Cambridge Crystallographic Data Centre with a number of CCDC 2107557.

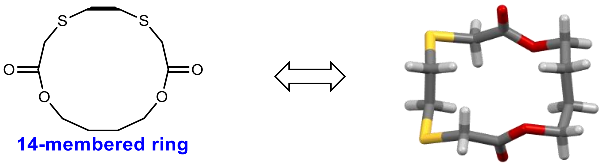

Supplementary Figure 22. ORTEP view of the crystal structure of 1,10-dioxa-4,7-dithiacyclotetradecane-2,9-dione (The ellipsoids are shown at 30% probability levels)

Supplementary Table 10. Crystal data and structure refinement for **4**

Identification code	LSW-10
Empirical formula	$C_{10}H_{16}O_4S_2$
Formula weight	264.35
Temperature/K	150.00(10)
Crystal system	monoclinic
Space group	$P2_1/n$
a/Å	8.1673(5)
b/Å	9.6459(9)
c/Å	15.6056(11)
α/°	90
β/°	91.608(5)
γ/°	90
Volume/Å^3	1228.94(16)
Z	4
ρ_{calc}g/cm^3	1.429
μ/mm^{-1}	0.429
F(000)	560.0
Crystal size/mm^3	0.14 × 0.13 × 0.12
Radiation	Mo Kα (λ = 0.71073)
2Θ range for data collection/°	4.966 to 49.962
Index ranges	$-8 \leq h \leq 9$, $-11 \leq k \leq 8$, $-17 \leq l \leq 18$
Reflections collected	5187
Independent reflections	2163 [R_{int} = 0.0404, R_{sigma} = 0.0531]
Data/restraints/parameters	2163/0/145
Goodness-of-fit on F^2	1.098
Final R indexes [I>=2σ (I)]	R_1 = 0.0436, wR_2 = 0.1112
Final R indexes [all data]	R_1 = 0.0520, wR_2 = 0.1188
Largest diff. peak/hole / e Å^{-3}	0.47/-0.41

The X-ray data of **7** is deposited in the Cambridge Crystallographic Data Centre with a number of CCDC 2107582.

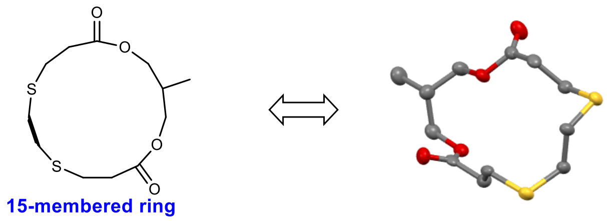

Supplementary Figure 23. ORTEP view of the crystal structure of 3-methyl-1,5-dioxa-9,12-dithiacyclopentadecane-6,15-dione (The ellipsoids are shown at 30% probability levels)

Supplementary Table 11. Crystal data and structure refinement for **7**

Identification code	w01
Empirical formula	$C_{12}H_{20}O_4S_2$
Formula weight	292.40
Temperature/K	150.00(10)
Crystal system	triclinic
Space group	P-1
a/Å	8.1221(10)
b/Å	9.0279(9)
c/Å	19.7667(16)
α/°	91.670(8)
β/°	101.485(9)
γ/°	90.925(9)
Volume/Å^3	1419.4(3)
Z	4
ρ_{calc}g/cm^3	1.368
μ/mm^{-1}	0.379
F(000)	624.0
Crystal size/mm^3	0.15 × 0.12 × 0.1
Radiation	Mo Kα (λ = 0.71073)
2Θ range for data collection/°	4.208 to 49.99
Index ranges	$-9 \leq h \leq 9$, $-10 \leq k \leq 10$, $-23 \leq l \leq 23$
Reflections collected	11720
Independent reflections	5012 [R_{int} = 0.0536, R_{sigma} = 0.0768]
Data/restraints/parameters	5012/0/346
Goodness-of-fit on F^2	1.056
Final R indexes [I>=2σ (I)]	R_1 = 0.0901, wR_2 = 0.2208
Final R indexes [all data]	R_1 = 0.1108, wR_2 = 0.2329
Largest diff. peak/hole / e Å^{-3}	0.78/-0.47

The X-ray data of **9** is deposited in the Cambridge Crystallographic Data Centre with a number of CCDC 2107579.

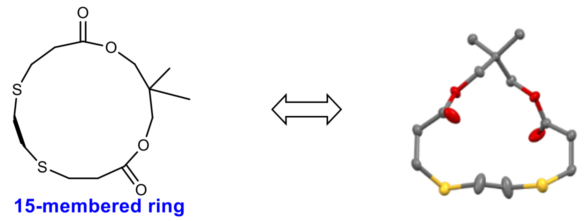

Supplementary Figure 24. ORTEP view of the crystal structure of 3,3-dimethyl-1,5-dioxa-9,12-dithiacyclopentadecane-6,15-dione (The ellipsoids are shown at 30% probability levels)

Supplementary Table 12. Crystal data and structure refinement for **9**

Identification code	5
Empirical formula	$C_{13}H_{22}O_4S_2$
Formula weight	306.42
Temperature/K	149.99(10)
Crystal system	monoclinic
Space group	C2/c
a/Å	12.4573(14)
b/Å	10.4357(11)
c/Å	11.8965(12)
α/°	90
β/°	98.983(10)
γ/°	90
Volume/Å^3	1527.6(3)
Z	4
ρ_{calc}g/cm^3	1.332
μ/mm^{-1}	0.355
F(000)	656.0
Crystal size/mm^3	0.13 × 0.11 × 0.09
Radiation	Mo Kα (λ = 0.71073)
2Θ range for data collection/°	5.118 to 49.93
Index ranges	$-12 \leq h \leq 14$, $-8 \leq k \leq 12$, $-14 \leq l \leq 13$
Reflections collected	3054
Independent reflections	1343 [R_{int} = 0.0337, R_{sigma} = 0.0394]
Data/restraints/parameters	1343/0/88
Goodness-of-fit on F^2	1.063
Final R indexes [I>=2σ (I)]	R_1 = 0.0436, wR_2 = 0.1131
Final R indexes [all data]	R_1 = 0.0490, wR_2 = 0.1179
Largest diff. peak/hole / e Å^{-3}	0.42/-0.35

The X-ray data of **13** is deposited in the Cambridge Crystallographic Data Centre with a number of CCDC 2107584.

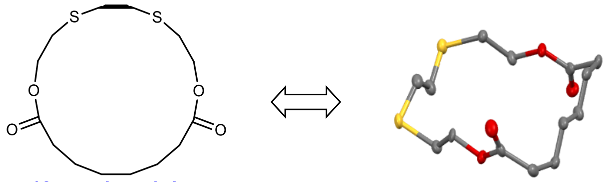

Supplementary Figure 25. ORTEP view of the crystal structure of 1,10-dioxa-4,7-dithiacyclooctadecane-11,18-dione (The ellipsoids are shown at 30% probability levels).

Supplementary Table 13. Crystal data and structure refinement for **13**

Identification code	w04
Empirical formula	$C_{14}H_{24}O_4S_2$
Formula weight	320.45
Temperature/K	150.00(10)
Crystal system	triclinic
Space group	P-1
a/Å	7.7216(9)
b/Å	10.1764(16)
c/Å	11.1812(10)
α/°	92.321(10)
β/°	93.139(8)
γ/°	112.140(13)
Volume/Å^3	810.82(19)
Z	2
ρ_{calc}g/cm^3	1.313
μ/mm^{-1}	0.338
F(000)	344.0
Crystal size/mm^3	0.15 × 0.13 × 0.12
Radiation	Mo Kα (λ = 0.71073)
2Θ range for data collection/°	4.33 to 49.99
Index ranges	$-8 \le h \le 9$, $-12 \le k \le 11$, $-13 \le l \le 12$
Reflections collected	5310
Independent reflections	2811 [R_{int} = 0.1120, R_{sigma} = 0.1508]
Data/restraints/parameters	2811/0/182
Goodness-of-fit on F^2	1.002
Final R indexes [I>=2σ (I)]	R_1 = 0.0876, wR_2 = 0.2008
Final R indexes [all data]	R_1 = 0.1147, wR_2 = 0.2408
Largest diff. peak/hole / e Å^{-3}	0.69/-1.02

The X-ray data of **15** is deposited in the Cambridge Crystallographic Data Centre with a number of CCDC 2107578.

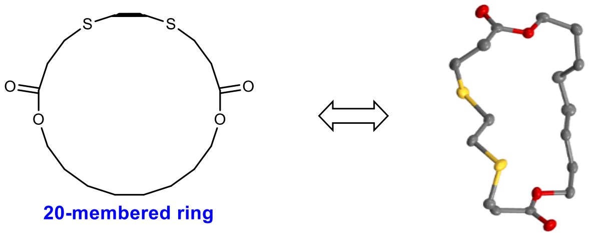

Supplementary Figure 26. ORTEP view of the crystal structure of 1,12-dioxa-5,8-dithiacycloicosane-2,11-dione (The ellipsoids are shown at 30% probability levels).

Supplementary Table 14. Crystal data and structure refinement for **15**

Identification code	04
Empirical formula	$C_{16}H_{28}O_4S_2$
Formula weight	348.50
Temperature/K	149.99(10)
Crystal system	monoclinic
Space group	$P2_1/c$
a/Å	16.7363(12)
b/Å	5.2768(3)
c/Å	21.9744(16)
α/°	90
β/°	111.390(8)
γ/°	90
Volume/Å^3	1807.0(2)
Z	4
ρ_{calc}g/cm^3	1.281
μ/mm^{-1}	0.309
F(000)	752.0
Crystal size/mm^3	0.15 × 0.13 × 0.12
Radiation	Mo Kα (λ = 0.71073)
2Θ range for data collection/°	3.982 to 50
Index ranges	$-12 \leq h \leq 19$, $-6 \leq k \leq 4$, $-26 \leq l \leq 23$
Reflections collected	8081
Independent reflections	3186 [R_{int} = 0.0260, R_{sigma} = 0.0330]
Data/restraints/parameters	3186/0/199
Goodness-of-fit on F^2	1.029
Final R indexes [I>=2σ (I)]	R_1 = 0.0352, wR_2 = 0.0808
Final R indexes [all data]	R_1 = 0.0410, wR_2 = 0.0846
Largest diff. peak/hole / e Å^{-3}	0.19/-0.30

The X-ray data of **19** is deposited in the Cambridge Crystallographic Data Centre with a number of CCDC 2107586.

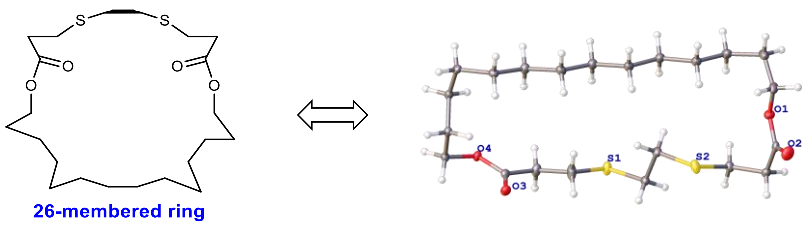

Supplementary Figure 27. ORTEP view of the crystal structure of 1,12-dioxa-5,8-dithiacyclohexacosane-2,11-dione (The ellipsoids are shown at 30% probability levels).

Supplementary Table 15. Crystal data and structure refinement for **19**

Identification code	LSW-011
Empirical formula	$C_{22}H_{40}O_4S_2$
Formula weight	432.66
Temperature/K	149.99(10)
Crystal system	triclinic
Space group	P-1
a/Å	5.0707(4)
b/Å	15.8154(13)
c/Å	16.4252(14)
α/°	110.718(8)
β/°	96.633(8)
γ/°	95.381(7)
Volume/Å^3	1210.87(18)
Z	2
ρ_{calc}g/cm^3	1.187
μ/mm^{-1}	0.243
F(000)	472.0
Crystal size/mm^3	0.15 × 0.13 × 0.12
Radiation	Mo Kα (λ = 0.71073)
2Θ range for data collection/°	4.524 to 58.894
Index ranges	$-7 \le h \le 6$, $-21 \le k \le 17$, $-15 \le l \le 20$
Reflections collected	9280
Independent reflections	5597 [R_{int} = 0.0262, R_{sigma} = 0.0555]
Data/restraints/parameters	5597/0/272
Goodness-of-fit on F^2	1.050
Final R indexes [I>=2σ (I)]	R_1 = 0.0534, wR_2 = 0.1204
Final R indexes [all data]	R_1 = 0.0739, wR_2 = 0.1352
Largest diff. peak/hole / e Å^{-3}	0.52/-0.31

The X-ray data of **23** is deposited in the Cambridge Crystallographic Data Centre with a number of CCDC 2107580.

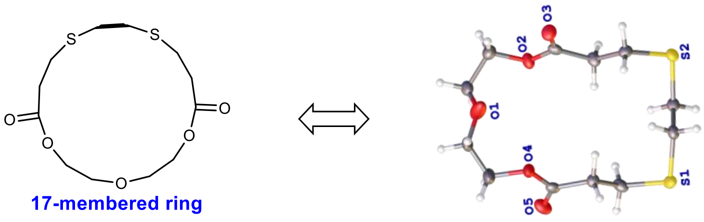

Supplementary Figure 28. ORTEP view of the crystal structure of 1,4,7-trioxa-11,14-dithiacycloheptadecane-8,17-dione (The ellipsoids are shown at 30% probability levels).

Supplementary Table 16. Crystal data and structure refinement for **23**

Identification code	6
Empirical formula	$C_{12}H_{20}O_5S_2$
Formula weight	308.40
Temperature/K	150.00(10)
Crystal system	triclinic
Space group	P-1
a/Å	8.2937(5)
b/Å	8.9887(7)
c/Å	10.6686(9)
α/°	73.070(7)
β/°	88.524(6)
γ/°	73.584(6)
Volume/Å^3	728.47(10)
Z	2
ρ_{calc}g/cm^3	1.406
μ/mm^{-1}	0.378
F(000)	328.0
Crystal size/mm^3	0.14 × 0.12 × 0.11
Radiation	Mo Kα (λ = 0.71073)
2Θ range for data collection/°	4.946 to 49.996
Index ranges	$-9 \le h \le 8$, $-10 \le k \le 10$, $-12 \le l \le 12$
Reflections collected	4683
Independent reflections	2566 [R_{int} = 0.0257, R_{sigma} = 0.0450]
Data/restraints/parameters	2566/30/182
Goodness-of-fit on F^2	1.056
Final R indexes [I>=2σ (I)]	R_1 = 0.0599, wR_2 = 0.1305
Final R indexes [all data]	R_1 = 0.0691, wR_2 = 0.1376
Largest diff. peak/hole / e Å^{-3}	1.09/-1.32

The X-ray data of **32** is deposited in the Cambridge Crystallographic Data Centre with a number of CCDC 2107590.

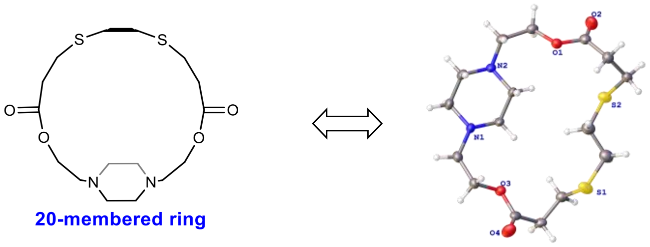

Supplementary Figure 29. ORTEP view of the crystal structure of 4,15-dioxa-8,11-dithia-1,18-diazabicyclo [16.2.2]docosane-5,14-dione (The ellipsoids are shown at 30% probability levels).

Supplementary Table 17. Crystal data and structure refinement for **32**

Identification code	zhu02
Empirical formula	$C_{16}H_{28}N_2O_4S_2$
Formula weight	376.52
Temperature/K	150.00(10)
Crystal system	orthorhombic
Space group	Pbca
a/Å	41.224(3)
b/Å	8.8322(6)
c/Å	10.1634(9)
α/°	90
β/°	90
γ/°	90
Volume/Å^3	3700.4(5)
Z	8
ρ_{calc}g/cm^3	1.352
μ/mm^{-1}	0.310
F(000)	1616.0
Crystal size/mm^3	0.15 × 0.13 × 0.12
Radiation	Mo Kα (λ = 0.71073)
2Θ range for data collection/°	5.018 to 49.998
Index ranges	$-37 \leq h \leq 49$, $-9 \leq k \leq 10$, $-7 \leq l \leq 12$
Reflections collected	11451
Independent reflections	3253 [R_{int} = 0.0459, R_{sigma} = 0.0521]
Data/restraints/parameters	3253/23/217
Goodness-of-fit on F^2	1.056
Final R indexes [I>=2σ (I)]	R_1 = 0.0626, wR_2 = 0.1375
Final R indexes [all data]	R_1 = 0.0803, wR_2 = 0.1485
Largest diff. peak/hole / e Å^{-3}	0.88/-0.53

The X-ray data of **33** is deposited in the Cambridge Crystallographic Data Centre with a number of CCDC 2107581.

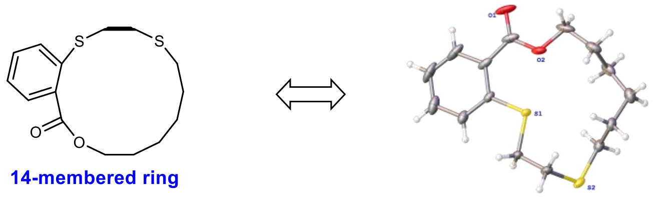

Supplementary Figure 30. ORTEP view of the crystal structure of 2,3,5,6,7,8,9,10-octahydro-12*H*-benzo[*c*][1]oxa[5,8]dithiacyclotetradecin-12-one (The ellipsoids are shown at 30% probability levels)

Supplementary Table 18. Crystal data and structure refinement for **33**

Identification code	1-B
Empirical formula	$C_{15}H_{20}O_2S_2$
Formula weight	296.43
Temperature/K	150.00(10)
Crystal system	monoclinic
Space group	$P2_1/c$
a/Å	9.1300(8)
b/Å	11.1901(11)
c/Å	14.3824(11)
α/°	90
β/°	96.548(8)
γ/°	90
Volume/$Å^3$	1459.8(2)
Z	4
ρ_{calc}g/cm^3	1.349
μ/mm^{-1}	0.360
F(000)	632.0
Crystal size/mm^3	0.14 × 0.12 × 0.11
Radiation	Mo Kα (λ = 0.71073)
2Θ range for data collection/°	4.49 to 49.994
Index ranges	$-10 \le h \le 10$, $-13 \le k \le 12$, $-17 \le l \le 14$
Reflections collected	6333
Independent reflections	2571 [R_{int} = 0.0237, R_{sigma} = 0.0320]
Data/restraints/parameters	2571/36/172
Goodness-of-fit on F^2	1.059
Final R indexes [I>=2σ (I)]	R_1 = 0.0418, wR_2 = 0.1019
Final R indexes [all data]	R_1 = 0.0491, wR_2 = 0.1077
Largest diff. peak/hole / e $Å^{-3}$	0.36/-0.39

The X-ray data of **36** is deposited in the Cambridge Crystallographic Data Centre with a number of CCDC 2107575.

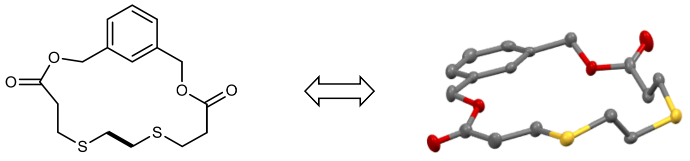

Supplementary Figure 31. ORTEP view of the crystal structure of 3,14-dioxa-7,10-dithia-1(1,3)-benzenacyclop entadecaphane-4,13-dione (The ellipsoids are shown at 30% probability levels).

Supplementary Table 19. Crystal data and structure refinement for **36**

Identification code	02_0401
Empirical formula	$C_{16}H_{20}O_4S_2$
Formula weight	340.44
Temperature/K	149.99(10)
Crystal system	monoclinic
Space group	$P2_1/n$
a/Å	5.7308(5)
b/Å	17.8889(17)
c/Å	16.1481(14)
α/°	90
β/°	95.643(8)
γ/°	90
Volume/Å^3	1647.4(3)
Z	4
ρ_{calc}g/cm^3	1.373
μ/mm^{-1}	0.338
F(000)	720.0
Crystal size/mm^3	0.14 × 0.13 × 0.11
Radiation	Mo Kα (λ = 0.71073)
2Θ range for data collection/°	4.554 to 49.996
Index ranges	$-6 \leq h \leq 6, -21 \leq k \leq 21, -2 \leq l \leq 19$
Reflections collected	2885
Independent reflections	2885 [R_{int} = 0.0720, R_{sigma} = 0.0754]
Data/restraints/parameters	2885/0/200
Goodness-of-fit on F^2	1.043
Final R indexes [I>=2σ (I)]	R_1 = 0.0926, wR_2 = 0.2418
Final R indexes [all data]	R_1 = 0.1074, wR_2 = 0.2521
Largest diff. peak/hole / e Å^{-3}	0.76/-0.43

Note: The structure of the orthogonal crystal system cannot be resolved probably due to twinning, disorder, poor crystal quality etc, but the C2 space group of monoclinic can be resolved. Low R1 and wR2 values are typically seen in the diffraction statistics of such systems.

The X-ray data of **37** is deposited in the Cambridge Crystallographic Data Centre with a number of CCDC 2107577.

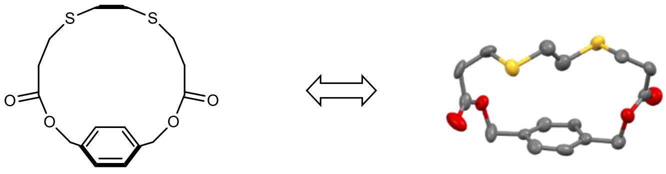

Supplementary Figure 32. ORTEP view of the crystal structure of 3,14-dioxa-7,10-dithia-1(1,4)-benzenacyclop entadecaphane-4,13-dione (The ellipsoids are shown at 30% probability levels).

Supplementary Table 20. Crystal data and structure refinement for **37**

Identification code	3_2
Empirical formula	$C_{64}H_{80}O_{16}S_8$
Formula weight	1361.76
Temperature/K	150.00(10)
Crystal system	monoclinic
Space group	C2
a/Å	18.8982(12)
b/Å	8.2098(5)
c/Å	21.5929(12)
α/°	90
β/°	90.044(4)
γ/°	90
Volume/Å^3	3350.1(3)
Z	2
ρ_{calc}g/cm^3	1.350
μ/mm^{-1}	0.332
F(000)	1440.0
Crystal size/mm^3	0.15 × 0.13 × 0.12
Radiation	Mo Kα (λ = 0.71073)
2Θ range for data collection/°	4.31 to 58.976
Index ranges	-23 ≤ h ≤ 23, -10 ≤ k ≤ 10, 0 ≤ l ≤ 27
Reflections collected	7850
Independent reflections	7850 [R_{int} = 0.0793, R_{sigma} = 0.0752]
Data/restraints/parameters	7850/35/417
Goodness-of-fit on F^2	1.042
Final R indexes [I>=2σ (I)]	R_1 = 0.0583, wR_2 = 0.1297
Final R indexes [all data]	R_1 = 0.0742, wR_2 = 0.1465
Largest diff. peak/hole / e Å^{-3}	0.35/-0.33
Flack parameter	0.02(4)

The X-ray data of **38** is deposited in the Cambridge Crystallographic Data Centre with a number of CCDC 2107583.

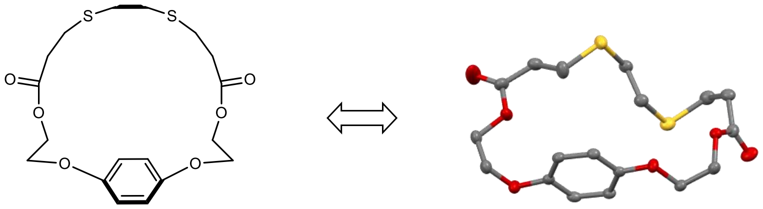

Supplementary Figure 33. ORTEP view of the crystal structure of 2,5,16,19-tetraoxa-9,12-dithia-1(1,4)-benzenacyclononadecaphane-6,15-dione (The ellipsoids are shown at 30% probability levels)

Supplementary Table 21. Crystal data and structure refinement for **38**

Identification code	w03
Empirical formula	$C_{18}H_{24}O_6S_2$
Formula weight	400.49
Temperature/K	150.00(10)
Crystal system	monoclinic
Space group	C2/c
a/Å	18.7979(12)
b/Å	8.6324(5)
c/Å	36.195(2)
α/°	90
β/°	98.334(6)
γ/°	90
Volume/Å^3	5811.5(6)
Z	12
ρ_{calc}g/cm^3	1.373
μ/mm^{-1}	0.306
F(000)	2544.0
Crystal size/mm^3	0.15 × 0.13 × 0.12
Radiation	Mo Kα (λ = 0.71073)
2Θ range for data collection/°	4.38 to 49.998
Index ranges	-16 ≤ h ≤ 22, -9 ≤ k ≤ 10, -42 ≤ l ≤ 43
Reflections collected	12817
Independent reflections	5116 [R_{int} = 0.0312, R_{sigma} = 0.0417]
Data/restraints/parameters	5116/0/361
Goodness-of-fit on F^2	1.096
Final R indexes [I>=2σ (I)]	R_1 = 0.0528, wR_2 = 0.1146
Final R indexes [all data]	R_1 = 0.0640, wR_2 = 0.1199
Largest diff. peak/hole / e Å^{-3}	0.42/-0.51
Flack parameter	0.02(4)

The X-ray data of **40** is deposited in the Cambridge Crystallographic Data Centre with a number of CCDC 2107588.

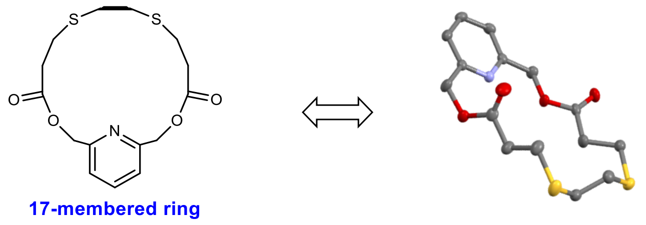

Supplementary Figure 34. ORTEP view of the crystal structure of 3,14-dioxa-7,10-dithia-1(2,6)-pyridina cyclopentadecaphane-4,13-dione (The ellipsoids are shown at 30% probability levels)

Supplementary Table 22. Crystal data and structure refinement for **40**

Identification code	lsw-012
Empirical formula	$C_{15}H_{19}NO_4S_2$
Formula weight	341.43
Temperature/K	150.00(10)
Crystal system	monoclinic
Space group	C2/c
a/Å	18.5000(15)
b/Å	10.9439(7)
c/Å	18.4444(14)
α/°	90
β/°	119.819(10)
γ/°	90
Volume/Å^3	3239.9(5)
Z	8
ρ_{calc}g/cm^3	1.400
μ/mm^{-1}	0.345
F(000)	1440.0
Crystal size/mm^3	0.15 × 0.13 × 0.12
Radiation	Mo Kα (λ = 0.71073)
2Θ range for data collection/°	4.504 to 50
Index ranges	$-22 \le h \le 16, -12 \le k \le 12, -18 \le l \le 21$
Reflections collected	7267
Independent reflections	2847 [R_{int} = 0.0248, R_{sigma} = 0.0309]
Data/restraints/parameters	2847/0/199
Goodness-of-fit on F^2	1.031
Final R indexes [I>=2σ (I)]	R_1 = 0.0333, wR_2 = 0.0771
Final R indexes [all data]	R_1 = 0.0397, wR_2 = 0.0811
Largest diff. peak/hole / e Å^{-3}	0.20/-0.22

The X-ray data of **49** is deposited in the Cambridge Crystallographic Data Centre with a number of CCDC 2107589.

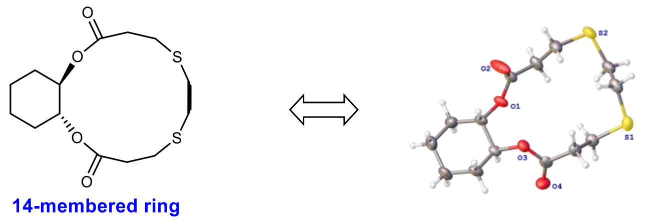

Supplementary Figure 35. ORTEP view of the crystal structure of 49 (The ellipsoids are shown at 30% probability levels)

Supplementary Table 23. Crystal data and structure refinement for **49**

Identification code	zhu01_2
Empirical formula	$C_{14}H_{22}O_4S_2$
Formula weight	318.43
Temperature/K	149.99(10)
Crystal system	triclinic
Space group	P-1
a/Å	7.1947(6)
b/Å	10.0255(8)
c/Å	11.0983(8)
α/°	79.978(6)
β/°	82.630(7)
γ/°	85.276(7)
Volume/Å^3	780.32(11)
Z	2
ρ_{calc}g/cm^3	1.355
μ/mm^{-1}	0.351
F(000)	340.0
Crystal size/mm^3	0.13 × 0.12 × 0.09
Radiation	Mo Kα (λ = 0.71073)
2Θ range for data collection/°	4.134 to 50
Index ranges	-8 ≤ h ≤ 8, -11 ≤ k ≤ 11, -12 ≤ l ≤ 13
Reflections collected	4408
Independent reflections	2682 [R_{int} = 0.0914, R_{sigma} = 0.0921]
Data/restraints/parameters	2682/0/181
Goodness-of-fit on F^2	1.049
Final R indexes [I>=2σ (I)]	R_1 = 0.0653, wR_2 = 0.1801
Final R indexes [all data]	R_1 = 0.0753, wR_2 = 0.1952
Largest diff. peak/hole / e Å^{-3}	0.52/-0.42
Flack parameter	0.02(4)

The X-ray data of **50** is deposited in the Cambridge Crystallographic Data Centre with a number of CCDC 2107585.

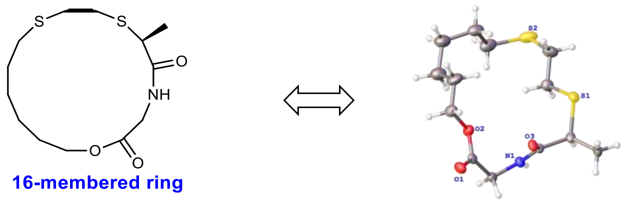

Supplementary Figure 36. ORTEP view of the crystal structure of (*S*)-6-methyl-1-oxa-7,10-dithia-4-azacyclo hexadecane-2,5-dione (The ellipsoids are shown at 30% probability levels)

Supplementary Table 24. Crystal data and structure refinement for **50**

Identification code	lsw-1-010
Empirical formula	$C_{13}H_{23}NO_3S_2$
Formula weight	305.44
Temperature/K	149.99(10)
Crystal system	monoclinic
Space group	$P2_1/c$
a/Å	5.0325(13)
b/Å	36.87(2)
c/Å	8.672(2)
α/°	90
β/°	103.80(3)
γ/°	90
Volume/Å^3	1562.7(11)
Z	4
ρ_{calc}g/cm^3	1.298
μ/mm^{-1}	0.344
F(000)	656.0
Crystal size/mm^3	0.13 × 0.1 × 0.07
Radiation	Mo Kα (λ = 0.71073)
2Θ range for data collection/°	4.418 to 49.994
Index ranges	$-5 \leq h \leq 4$, $-38 \leq k \leq 43$, $-9 \leq l \leq 10$
Reflections collected	6759
Independent reflections	2736 [R_{int} = 0.1084, R_{sigma} = 0.1433]
Data/restraints/parameters	2736/16/181
Goodness-of-fit on F^2	1.060
Final R indexes [I>=2σ (I)]	R_1 = 0.1105, wR_2 = 0.2269
Final R indexes [all data]	R_1 = 0.1735, wR_2 = 0.2746
Largest diff. peak/hole / e Å^{-3}	0.71/-0.39
Flack parameter	0.02(4)

The X-ray data of **54** is deposited in the Cambridge Crystallographic Data Centre with a number of CCDC 2107556.

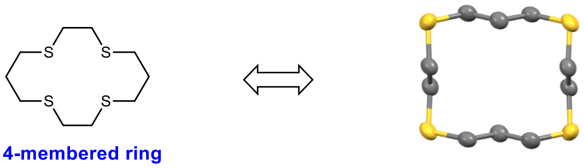

Supplementary Figure 37. ORTEP view of the crystal structure of 1,4,8,11-tetrathiacyclotetradecane (The ellipsoids are shown at 30% probability levels)

Supplementary Table 25. Crystal data and structure refinement for **54**

Identification code	7S
Empirical formula	$C_{10}H_{20}S_4$
Formula weight	268.50
Temperature/K	293(2)
Crystal system	monoclinic
Space group	$P2_1/n$
a/Å	5.3448(15)
b/Å	8.2866(19)
c/Å	15.545(4)
α/°	90
β/°	93.01(3)
γ/°	90
Volume/$Å^3$	687.5(3)
Z	2
$\rho_{calc}g/cm^3$	1.297
μ/mm^{-1}	0.656
F(000)	288.0
Crystal size/mm^3	0.14 × 0.12 × 0.11
Radiation	Mo Kα (λ = 0.71073)
2Θ range for data collection/°	5.248 to 49.998
Index ranges	$-6 \le h \le 6$, $-9 \le k \le 9$, $-18 \le l \le 14$
Reflections collected	2700
Independent reflections	1209 [R_{int} = 0.0280, R_{sigma} = 0.0397]
Data/restraints/parameters	1209/0/64
Goodness-of-fit on F^2	1.061
Final R indexes [I>=2σ (I)]	R_1 = 0.0616, wR_2 = 0.1730
Final R indexes [all data]	R_1 = 0.0762, wR_2 = 0.1859
Largest diff. peak/hole / e $Å^{-3}$	0.37/-0.34

The X-ray data of **55** is deposited in the Cambridge Crystallographic Data Centre with a number of CCDC 2107558.

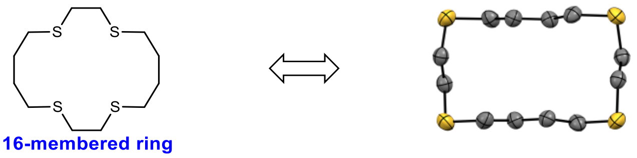

Supplementary Figure 38. ORTEP view of the crystal structure of 1,4,9,12-tetrathiacyclohexadecane (The ellipsoids are shown at 30% probability levels)

Supplementary Table 26. Crystal data and structure refinement for **55**

Identification code	16SS
Empirical formula	$C_{12}H_{24}S_4$
Formula weight	296.55
Temperature/K	293(2)
Crystal system	orthorhombic
Space group	Fddd
a/Å	8.2429(10)
b/Å	17.663(3)
c/Å	21.111(3)
α/°	90
β/°	90
γ/°	90
Volume/Å^3	3073.7(7)
Z	8
ρ_{calc}g/cm^3	1.282
μ/mm^{-1}	0.594
F(000)	1280.0
Crystal size/mm^3	0.14 × 0.13 × 0.12
Radiation	Mo Kα (λ = 0.71073)
2Θ range for data collection/°	5.786 to 49.996
Index ranges	$-9 \le h \le 9, -20 \le k \le 19, -24 \le l \le 24$
Reflections collected	3489
Independent reflections	683 [R_{int} = 0.0250, R_{sigma} = 0.0168]
Data/restraints/parameters	683/0/38
Goodness-of-fit on F^2	1.054
Final R indexes [I>=2σ (I)]	R_1 = 0.0446, wR_2 = 0.1243
Final R indexes [all data]	R_1 = 0.0489, wR_2 = 0.1320
Largest diff. peak/hole / e Å^{-3}	0.29/-0.33

The X-ray data of **56** is deposited in the Cambridge Crystallographic Data Centre with a number of CCDC 2107560.

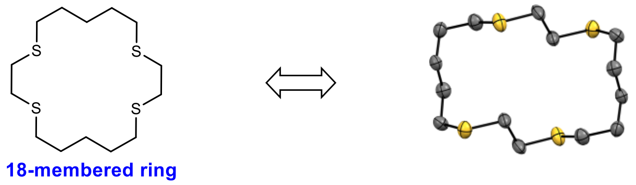

Supplementary Figure 39. ORTEP view of the crystal structure of 1,4,10,13-tetrathiacyclooctadecane (The ellipsoids are shown at 30% probability levels)

Supplementary Table 27. Crystal data and structure refinement for **56**

Identification code	ZSF-6
Empirical formula	$C_{14}H_{28}S_4$
Formula weight	324.60
Temperature/K	293(2)
Crystal system	triclinic
Space group	P-1
a/Å	5.2007(5)
b/Å	8.1496(16)
c/Å	10.679(2)
α/°	74.283(18)
β/°	79.026(13)
γ/°	87.417(12)
Volume/Å^3	427.72(13)
Z	1
ρ_{calc}g/cm^3	1.260
μ/mm^{-1}	0.539
F(000)	176.0
Crystal size/mm^3	0.14 × 0.12 × 0.11
Radiation	Mo Kα (λ = 0.71073)
2Θ range for data collection/°	4.032 to 49.982
Index ranges	$-6 \leq h \leq 6$, $-9 \leq k \leq 8$, $-9 \leq l \leq 12$
Reflections collected	2926
Independent reflections	1502 [R_{int} = 0.0287, R_{sigma} = 0.0521]
Data/restraints/parameters	1502/0/82
Goodness-of-fit on F^2	1.070
Final R indexes [I>=2σ (I)]	R_1 = 0.0575, wR_2 = 0.1729
Final R indexes [all data]	R_1 = 0.0744, wR_2 = 0.1901
Largest diff. peak/hole / e Å^{-3}	0.48/-0.34

The X-ray data of **57**is deposited in the Cambridge Crystallographic Data Centre with a number of CCDC 2107566.

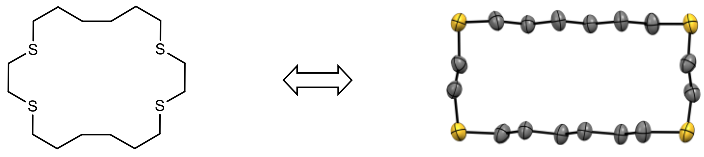

Supplementary Figure 40. ORTEP view of the crystal structure of 1,4,11,14-tetrathiacycloicosane (The ellipsoids are shown at 30% probability levels)

Supplementary Table 28. Crystal data and structure refinement for **57**

Identification code	zsf-3
Empirical formula	$C_{16}H_{32}S_4$
Formula weight	352.65
Temperature/K	293(2)
Crystal system	monoclinic
Space group	C2/c
a/Å	14.1744(12)
b/Å	13.3009(9)
c/Å	10.4879(10)
α/°	90
β/°	97.566(8)
γ/°	90
Volume/Å^3	1960.1(3)
Z	4
ρ_{calc}g/cm^3	1.195
μ/mm^{-1}	0.476
F(000)	768.0
Crystal size/mm^3	0.12 × 0.11 × 0.1
Radiation	Mo Kα (λ = 0.71073)
2Θ range for data collection/°	4.216 to 49.994
Index ranges	$-14 \leq h \leq 16$, $-15 \leq k \leq 12$, $-11 \leq l \leq 12$
Reflections collected	4109
Independent reflections	1731 [R_{int} = 0.0220, R_{sigma} = 0.0283]
Data/restraints/parameters	1731/0/92
Goodness-of-fit on F^2	1.057
Final R indexes [I>=2σ (I)]	R_1 = 0.0456, wR_2 = 0.1159
Final R indexes [all data]	R_1 = 0.0539, wR_2 = 0.1246
Largest diff. peak/hole / e Å^{-3}	0.26/-0.23

The X-ray data of **58** is deposited in the Cambridge Crystallographic Data Centre with a number of CCDC 2107567.

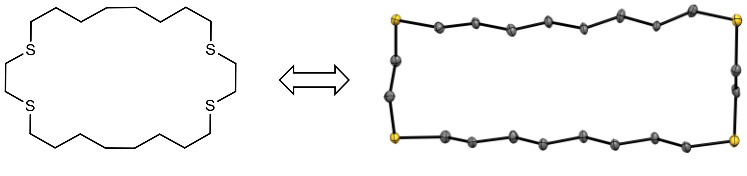

Supplementary Figure 41. ORTEP view of the crystal structure of 1,4,13,16-tetrathiacyclotetracosane (The ellipsoids are shown at 30% probability levels).

Supplementary Table 29. Crystal data and structure refinement for **58**

Identification code	lsw-18_tw
Empirical formula	$C_{20}H_{40}S_4$
Formula weight	408.76
Temperature/K	99.98(10)
Crystal system	monoclinic
Space group	$P2_1/c$
a/Å	11.3948(7)
b/Å	21.3854(12)
c/Å	9.3625(6)
α/°	90
β/°	98.762(6)
γ/°	90
Volume/Å^3	2254.9(2)
Z	4
ρ_{calc}g/cm^3	1.204
μ/mm^{-1}	0.423
F(000)	896.0
Crystal size/mm^3	0.13 × 0.12 × 0.11
Radiation	Mo Kα (λ = 0.71073)
2Θ range for data collection/°	4.088 to 50
Index ranges	$-13 \leq h \leq 13$, $-25 \leq k \leq 25$, $-9 \leq l \leq 11$
Reflections collected	4597
Independent reflections	3965 [R_{int} = 0.1092, R_{sigma} = 0.0859]
Data/restraints/parameters	3965/0/217
Goodness-of-fit on F^2	1.050
Final R indexes [I>=2σ (I)]	R_1 = 0.0717, wR_2 = 0.1148
Final R indexes [all data]	R_1 = 0.0937, wR_2 = 0.1229
Largest diff. peak/hole / e Å^{-3}	0.72/-0.51

The X-ray data of **59** is deposited in the Cambridge Crystallographic Data Centre with a number of CCDC 2107574.

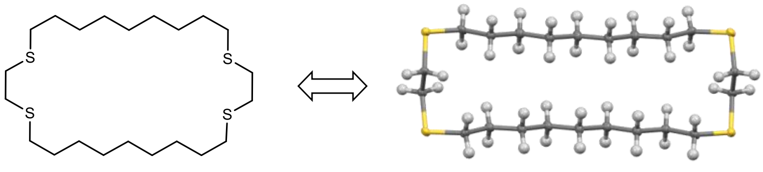

Supplementary Figure 42. ORTEP view of the crystal structure of 1,4,14,17-tetrathiacyclohexacosane (The ellipsoids are shown at 30% probability levels)

Supplementary Table 30. Crystal data and structure refinement for **59**

Identification code	lsw
Empirical formula	$C_{22}H_{44}S_4$
Formula weight	436.81
Temperature/K	149.99(10)
Crystal system	triclinic
Space group	P-1
a/Å	6.9789(8)
b/Å	8.0801(8)
c/Å	11.2415(12)
α/°	92.390(8)
β/°	94.189(9)
γ/°	104.649(9)
Volume/Å^3	610.47(12)
Z	1
ρ_{calc}g/cm^3	1.188
μ/mm^{-1}	0.395
F(000)	240.0
Crystal size/mm^3	0.12 × 0.11 × 0.1
Radiation	Mo Kα (λ = 0.71073)
2Θ range for data collection/°	5.222 to 49.99
Index ranges	$-8 \leq h \leq 8$, $-9 \leq k \leq 8$, $-13 \leq l \leq 11$
Reflections collected	3864
Independent reflections	2148 [R_{int} = 0.0283, R_{sigma} = 0.0448]
Data/restraints/parameters	2148/0/118
Goodness-of-fit on F^2	1.051
Final R indexes [I>=2σ (I)]	R_1 = 0.0464, wR_2 = 0.1231
Final R indexes [all data]	R_1 = 0.0542, wR_2 = 0.1321
Largest diff. peak/hole / e Å^{-3}	0.55/-0.48

The X-ray data of **60** is deposited in the Cambridge Crystallographic Data Centre with a number of CCDC 2107569.

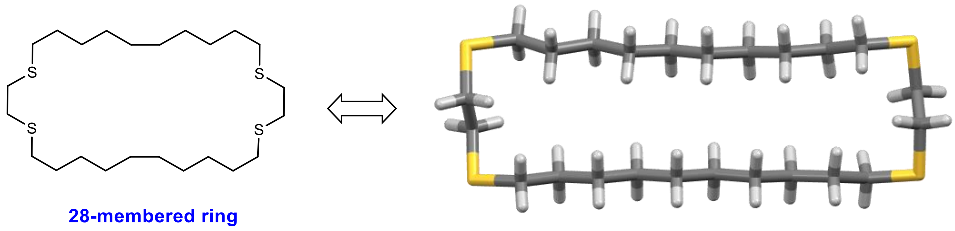

Supplementary Figure 43. ORTEP view of the crystal structure of 1,4,15,18-tetrathiacyclooctacosane (The ellipsoids are shown at 30% probability levels)

Supplementary Table 31. Crystal data and structure refinement for **60**

Identification code	lsw_2_tw_tw
Empirical formula	$C_{24}H_{48}S_4$
Formula weight	464.86
Temperature/K	100.0(2)
Crystal system	monoclinic
Space group	$P2_1/c$
a/Å	13.6074(7)
b/Å	21.3795(9)
c/Å	9.3514(5)
α/°	90
β/°	104.702(5)
γ/°	90
Volume/Å^3	2631.4(2)
Z	4
ρ_{calc}g/cm^3	1.173
μ/mm^{-1}	3.355
F(000)	1024.0
Crystal size/mm^3	0.12 × 0.11 × 0.1
Radiation	Cu Kα (λ = 1.54184)
2Θ range for data collection/°	6.716 to 146.812
Index ranges	$-16 \le h \le 16, -26 \le k \le 26, -8 \le l \le 11$
Reflections collected	5175
Independent reflections	5175 [R_{int} = ?, R_{sigma} = 0.0729]
Data/restraints/parameters	5175/0/254
Goodness-of-fit on F^2	1.175
Final R indexes [I>=2σ (I)]	R_1 = 0.0791, wR_2 = 0.2564
Final R indexes [all data]	R_1 = 0.0829, wR_2 = 0.2590
Largest diff. peak/hole / e Å^{-3}	1.03/-0.64

The X-ray data of **63**is deposited in the Cambridge Crystallographic Data Centre with a number of CCDC 2107565.

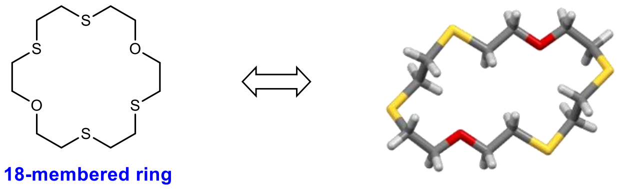

Supplementary Figure 44. ORTEP view of the crystal structure of 1,10-dioxa-4,7,13,16-tetrathiacyclooctadecane (The ellipsoids are shown at 30% probability levels)

Supplementary Table 32. Crystal data and structure refinement for **63**

Identification code	shuangS
Empirical formula	$C_{12}H_{24}O_2S_4$
Formula weight	328.55
Temperature/K	150.00(10)
Crystal system	triclinic
Space group	P-1
a/Å	5.2220(6)
b/Å	7.1063(12)
c/Å	10.7031(14)
α/°	97.484(13)
β/°	99.431(10)
γ/°	92.438(12)
Volume/Å^3	387.65(10)
Z	1
ρ_{calc}g/cm^3	1.407
μ/mm^{-1}	0.605
F(000)	176.0
Crystal size/mm^3	0.13 × 0.12 × 0.09
Radiation	Mo Kα (λ = 0.71073)
2Θ range for data collection/°	3.896 to 49.998
Index ranges	$-6 \leq h \leq 5$, $-8 \leq k \leq 6$, $-12 \leq l \leq 12$
Reflections collected	2479
Independent reflections	1374 [R_{int} = 0.0315, R_{sigma} = 0.0484]
Data/restraints/parameters	1374/0/82
Goodness-of-fit on F^2	1.060
Final R indexes [I>=2σ (I)]	R_1 = 0.0755, wR_2 = 0.2097
Final R indexes [all data]	R_1 = 0.0794, wR_2 = 0.2218
Largest diff. peak/hole / e Å^{-3}	0.73/-0.64

The X-ray data of **66** is deposited in the Cambridge Crystallographic Data Centre with a number of CCDC 2107554.

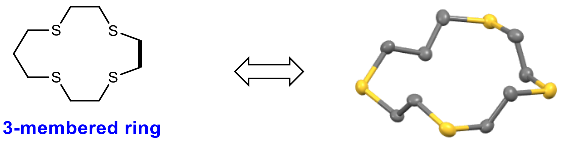

Supplementary Figure 45. ORTEP view of the crystal structure of 1,4,7,10-tetrathiacyclotridecane (The ellipsoids are shown at 30% probability levels)

Supplementary Table 33. Crystal data and structure refinement for **66**

Identification code	z04
Empirical formula	C9H18S4
Formula weight	254.47
Temperature/K	149.99(10)
Crystal system	orthorhombic
Space group	Cmc21
a/Å	29.4958(14)
b/Å	18.4454(10)
c/Å	9.1504(5)
α/°	90
β/°	90
γ/°	90
Volume/Å3	4978.4(5)
Z	16
ρcalcg/cm3	1.358
μ/mm-1	0.721
F(000)	2176.0
Crystal size/mm3	0.13 × 0.12 × 0.11
Radiation	Mo Kα (λ = 0.71073)
2Θ range for data collection/°	4.416 to 58.984
Index ranges	-40 ≤ h ≤ 29, -25 ≤ k ≤ 16, -10 ≤ l ≤ 11
Reflections collected	9361
Independent reflections	4863 [Rint = 0.0400, Rsigma = 0.0633]
Data/restraints/parameters	4863/377/352
Goodness-of-fit on F2	1.050
Final R indexes [I>=2σ (I)]	R1 = 0.0601, wR2 = 0.1414
Final R indexes [all data]	R1 = 0.0735, wR2 = 0.1520
Largest diff. peak/hole / e Å-3	0.95/-0.43
Flack parameter	0.38(11)
Identification code	shuangS
Empirical formula	$C_{12}H_{24}O_2S_4$
Formula weight	328.55
Temperature/K	150.00(10)
Crystal system	triclinic

Space group	P-1
a/Å	5.2220(6)
b/Å	7.1063(12)
c/Å	10.7031(14)
α/°	97.484(13)
β/°	99.431(10)
γ/°	92.438(12)
Volume/$Å^3$	387.65(10)
Z	1
$\rho_{calc}g/cm^3$	1.407
μ/mm^{-1}	0.605
F(000)	176.0
Crystal size/mm^3	0.13 × 0.12 × 0.09
Radiation	Mo Kα (λ = 0.71073)
2Θ range for data collection/°	3.896 to 49.998
Index ranges	-6 ≤ h ≤ 5, -8 ≤ k ≤ 6, -12 ≤ l ≤ 12
Reflections collected	2479
Independent reflections	1374 [R_{int} = 0.0315, R_{sigma} = 0.0484]
Data/restraints/parameters	1374/0/82
Goodness-of-fit on F^2	1.060
Final R indexes [I>=2σ (I)]	R_1 = 0.0755, wR_2 = 0.2097
Final R indexes [all data]	R_1 = 0.0794, wR_2 = 0.2218
Largest diff. peak/hole / e $Å^{-3}$	0.73/-0.64

The X-ray data of **69** is deposited in the Cambridge Crystallographic Data Centre with a number of CCDC 2107549.

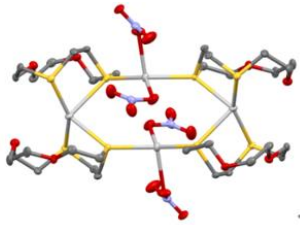

Supplementary Figure 46. ORTEP view of the crystal structure of 96 (The ellipsoids are shown at 30% probability levels)

Supplementary Table 34. Crystal data and structure refinement for **96**

Identification code	exp_2915
Empirical formula	$C_{16}H_{32}Ag_2N_2O_{10}S_4$
Formula weight	756.41
Temperature/K	150.00(10)
Crystal system	triclinic
Space group	P-1
a/Å	9.6864(4)
b/Å	11.7082(6)
c/Å	11.9652(4)
α/°	83.640(3)
β/°	78.454(3)
γ/°	74.588(4)
Volume/Å^3	1279.32(10)
Z	2
ρ_{calc}g/cm^3	1.964
μ/mm^{-1}	1.910
F(000)	760.0
Crystal size/mm^3	0.12 × 0.11 × 0.09
Radiation	Mo Kα (λ = 0.71073)
2Θ range for data collection/°	4.432 to 49.998
Index ranges	$-11 \leq h \leq 11$, $-13 \leq k \leq 12$, $-14 \leq l \leq 11$
Reflections collected	8393
Independent reflections	4510 [R_{int} = 0.0282, R_{sigma} = 0.0492]
Data/restraints/parameters	4510/17/307
Goodness-of-fit on F^2	1.058
Final R indexes [I>=2σ (I)]	R_1 = 0.0302, wR_2 = 0.0538
Final R indexes [all data]	R_1 = 0.0384, wR_2 = 0.0589
Largest diff. peak/hole / e Å^{-3}	0.80/-0.94

The X-ray data of **70** is deposited in the Cambridge Crystallographic Data Centre with a number of CCDC 2107552.

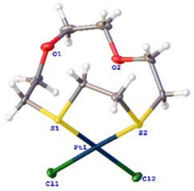

Supplementary Figure 47. ORTEP view of the crystal structure of 97 (The ellipsoids are shown at 30% probability levels)

Supplementary Table 35. Crystal data and structure refinement for **97**

Identification code	02-0320_2
Empirical formula	$C_8H_{16}Cl_2O_2PtS_2$
Formula weight	474.32
Temperature/K	150.00(10)
Crystal system	monoclinic
Space group	$P2_1/c$
a/Å	13.6034(7)
b/Å	8.4677(5)
c/Å	23.1552(15)
α/°	90
β/°	101.448(6)
γ/°	90
Volume/Å^3	2614.2(3)
Z	8
ρ_{calc}g/cm^3	2.410
μ/mm^{-1}	11.441
F(000)	1792.0
Crystal size/mm^3	0.13 × 0.12 × 0.11
Radiation	Mo Kα (λ = 0.71073)
2Θ range for data collection/°	4.226 to 49.992
Index ranges	$-16 \le h \le 13, -9 \le k \le 10, -21 \le l \le 27$
Reflections collected	11339
Independent reflections	4587 [R_{int} = 0.0325, R_{sigma} = 0.0435]
Data/restraints/parameters	4587/20/290
Goodness-of-fit on F^2	1.033
Final R indexes [I>=2σ (I)]	R_1 = 0.0347, wR_2 = 0.0705
Final R indexes [all data]	R_1 = 0.0463, wR_2 = 0.0762
Largest diff. peak/hole / e Å^{-3}	1.89/-1.18

The X-ray data of **71** is deposited in the Cambridge Crystallographic Data Centre with a number of CCDC 2107553.

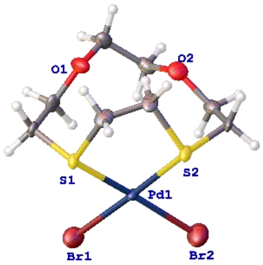

Supplementary Figure 48. ORTEP view of the crystal structure of 98 (The ellipsoids are shown at 30% probability levels)

Supplementary Table 36. Crystal data and structure refinement for **98**

Identification code	03
Empirical formula	$C_8H_{16}Br_2O_2PdS_2$
Formula weight	474.55
Temperature/K	150.00(10)
Crystal system	orthorhombic
Space group	Pbca
a/Å	8.5516(5)
b/Å	14.0523(13)
c/Å	23.0214(17)
α/°	90
β/°	90
γ/°	90
Volume/Å^3	2766.5(4)
Z	8
ρ_{calc}g/cm^3	2.279
μ/mm^{-1}	7.400
F(000)	1824.0
Crystal size/mm^3	0.13 × 0.1 × 0.08
Radiation	Mo Kα (λ = 0.71073)
2Θ range for data collection/°	5.798 to 49.976
Index ranges	$-8 \leq h \leq 10, -10 \leq k \leq 16, -27 \leq l \leq 26$
Reflections collected	7675
Independent reflections	2429 [R_{int} = 0.0372, R_{sigma} = 0.0389]
Data/restraints/parameters	2429/0/136
Goodness-of-fit on F^2	1.087
Final R indexes [I>=2σ (I)]	R_1 = 0.0714, wR_2 = 0.1906
Final R indexes [all data]	R_1 = 0.0839, wR_2 = 0.1995
Largest diff. peak/hole / e Å^{-3}	1.43/-1.21

Supplementary NMR Spectra and HPLC Spectra

Supplementary Figure 49 | ^{1}H NMR (400 MHz, 298K, $CDCl_3$) of 1,3,3-Trimethylbicyclo[2.2.1]heptan-2-yl 2-mercaptoacetate **(84a)**

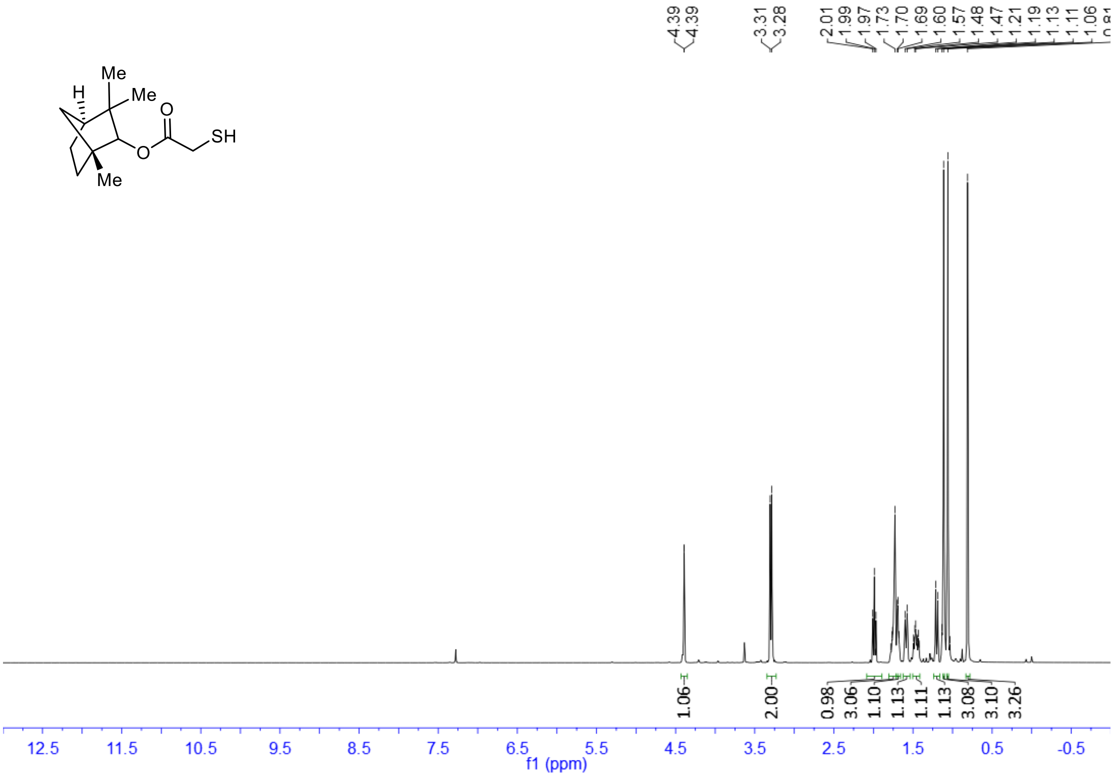

Supplementary Figure 50 | ^{13}C NMR (101MHz, 298K, $CDCl_3$) of 1,3,3-Trimethylbicyclo[2.2.1]heptan-2-yl 2-mercaptoacetate **(84a)**

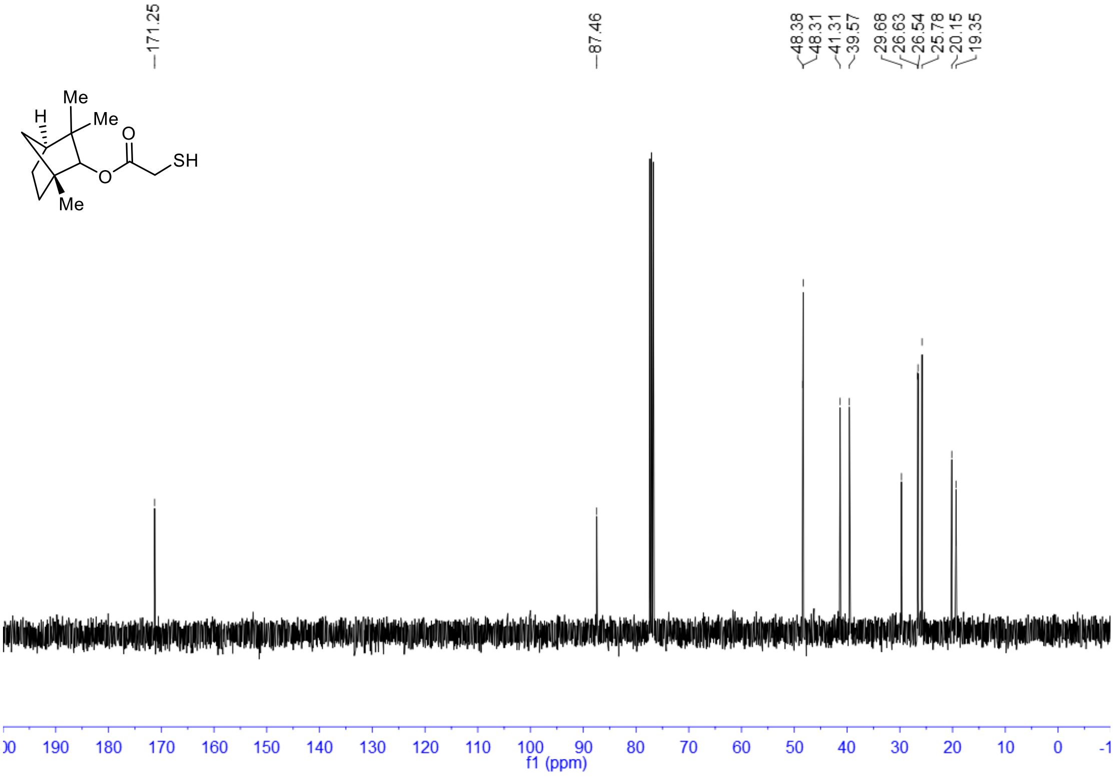

Supplementary Figure 51 | ^{1}H NMR (400 MHz, 298K, $CDCl_3$) of *S*-((3*R*,8*S*,9*S*,10*R*,13*R*,14*S*,17*R*)-10,13-Dimethyl-17-((*R*)-6-methylheptan-2-yl) 2,3,4,7,8,9,10, 11,12,13,14,15,16,17-tetradecahydro-1*H*-cyclopenta[*a*]phenanthren-3-yl) ethanethioate **(85a)**

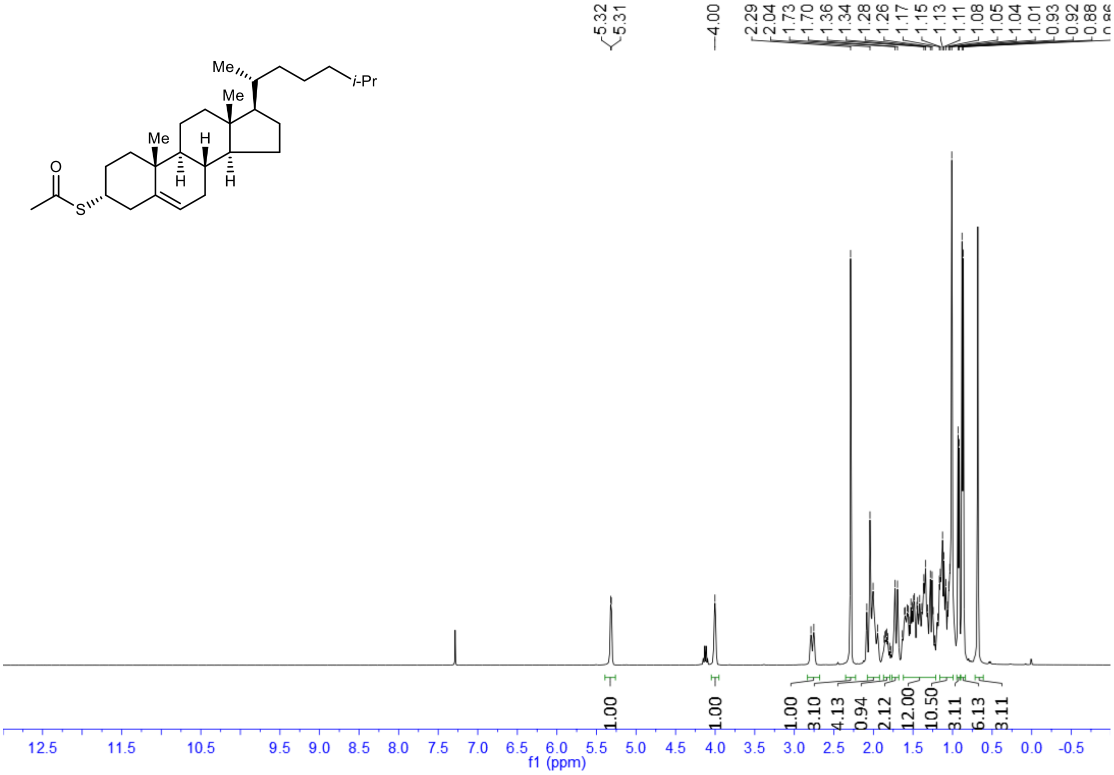

Supplementary Figure 52 | ^{13}C NMR (101 MHz, 298K, $CDCl_3$) of *S*-((3*R*,8*S*,9*S*,10*R*,13*R*,14*S*,17*R*)-10,13-Dimethyl-17-((*R*)-6-methylheptan-2-yl) 2,3,4,7,8,9,10, 11,12,13,14,15,16,17-tetradecahydro-1*H*-cyclopenta[*a*]phenanthren-3-yl) ethanethioate **(85a)**

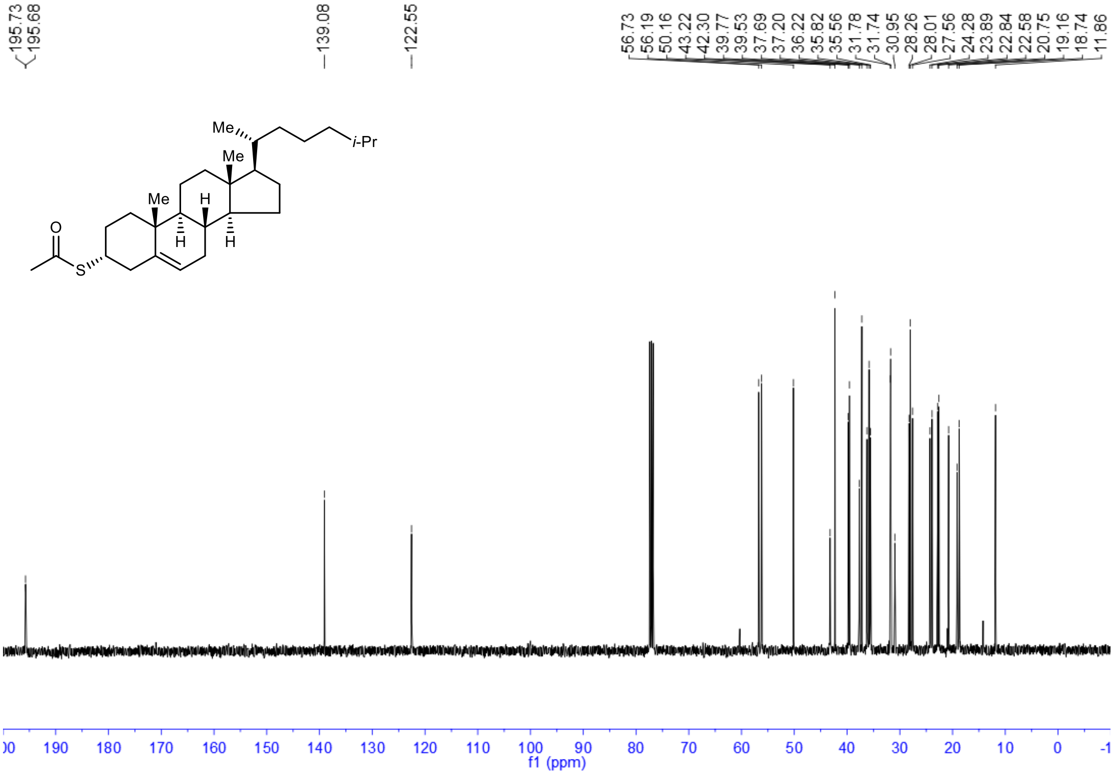

Supplementary Figure 53 | ^{1}H NMR (400 MHz, 298K, $CDCl_3$) of (3*R*)-Cholestanethiol **(85b)**

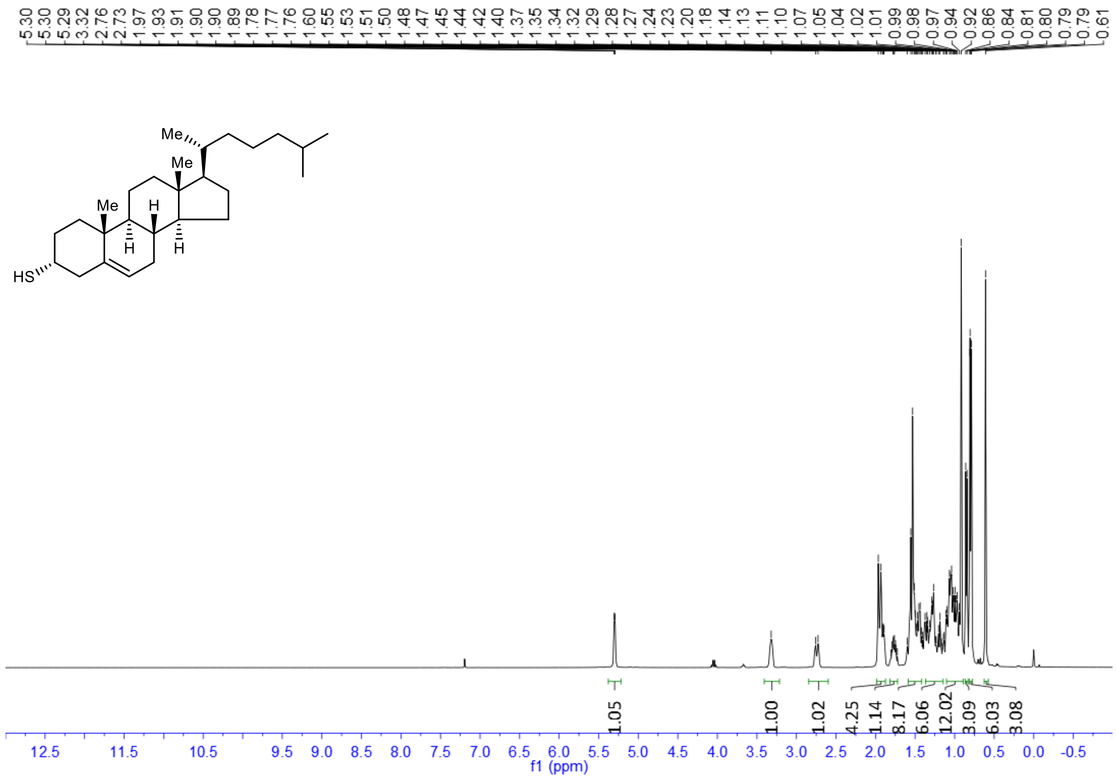

Supplementary Figure 54 | ^{13}C NMR (101 MHz, 298K, $CDCl_3$) of (3*R*)-Cholestanethiol **(85b)**

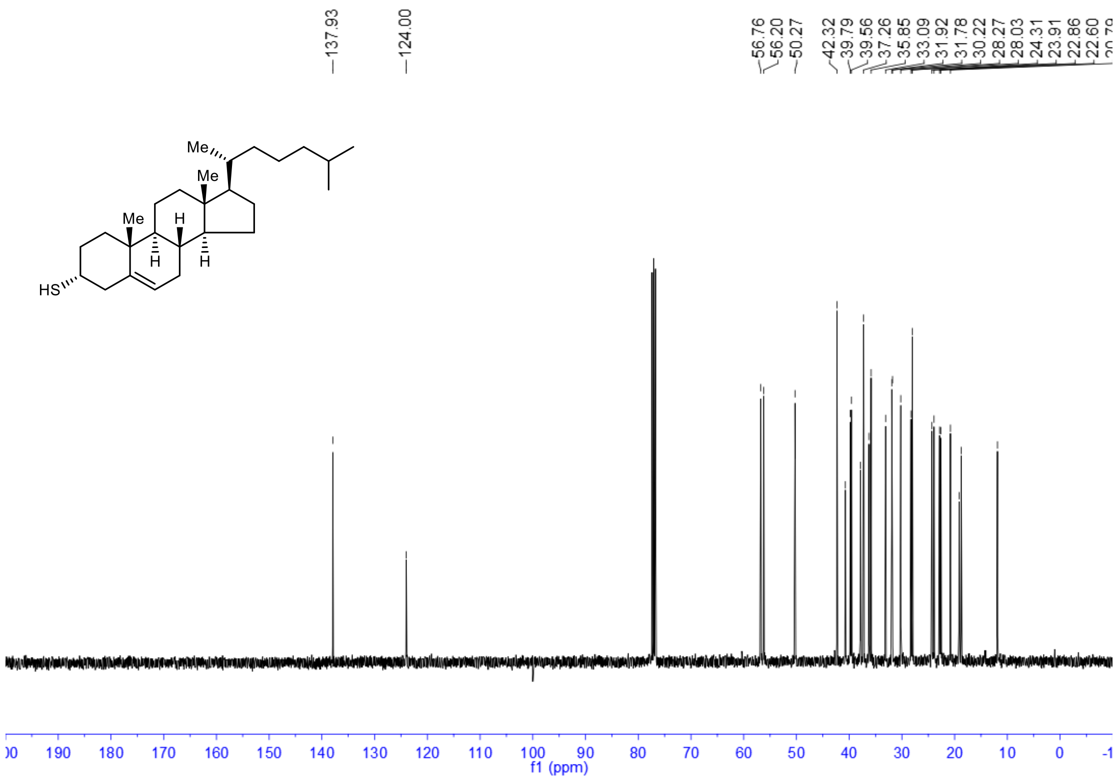

Supplementary Figure 55 | ^{1}H NMR (400 MHz, 298K, $CDCl_3$) of TRIP thiol **(87a)**

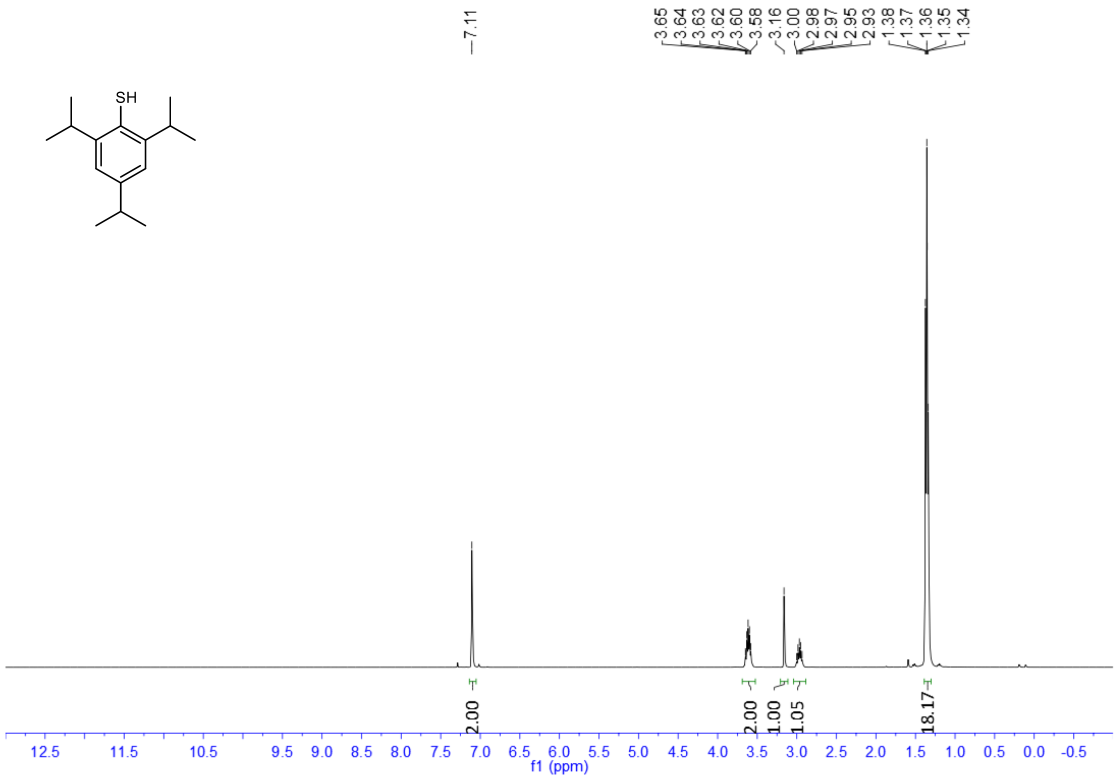

Supplementary Figure 56 | ^{13}C NMR (101 MHz, 298K, $CDCl_3$) of **TRIP** thiol **(87a)**

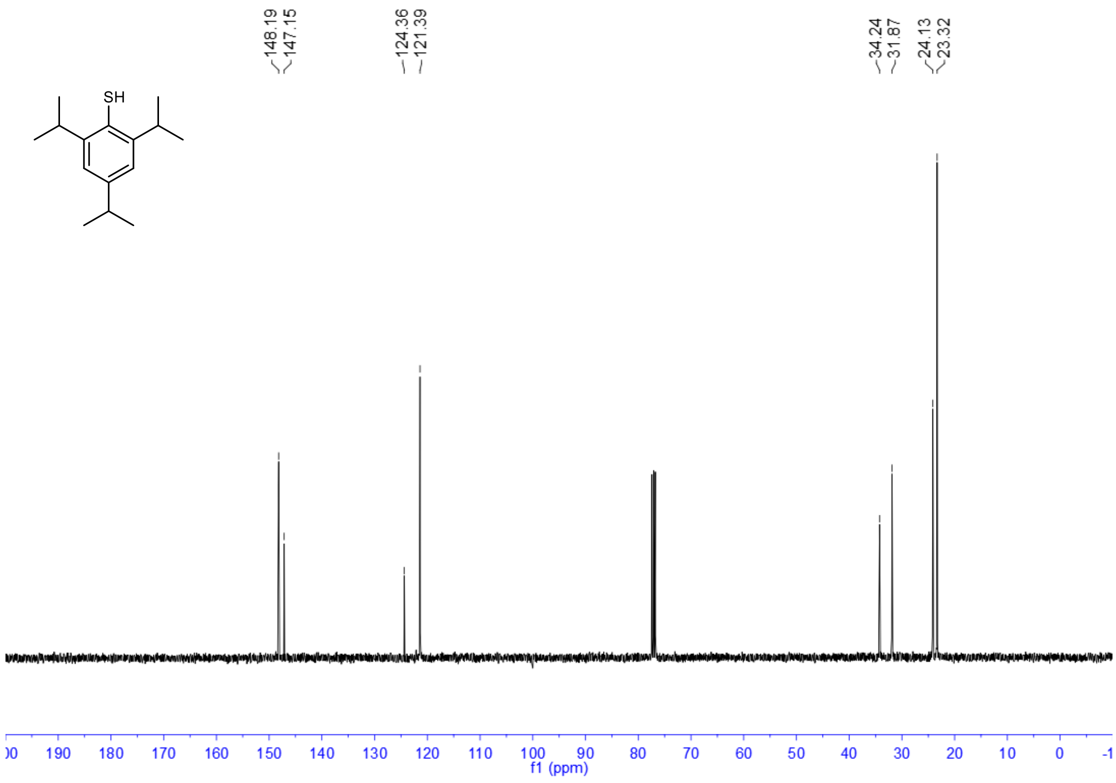

Supplementary Figure 57 | ^{1}H NMR (500 MHz, 298K, $CDCl_3$) of Ethane-1,2-diyl bis(3-mercaptopropanoate) **(5a)**

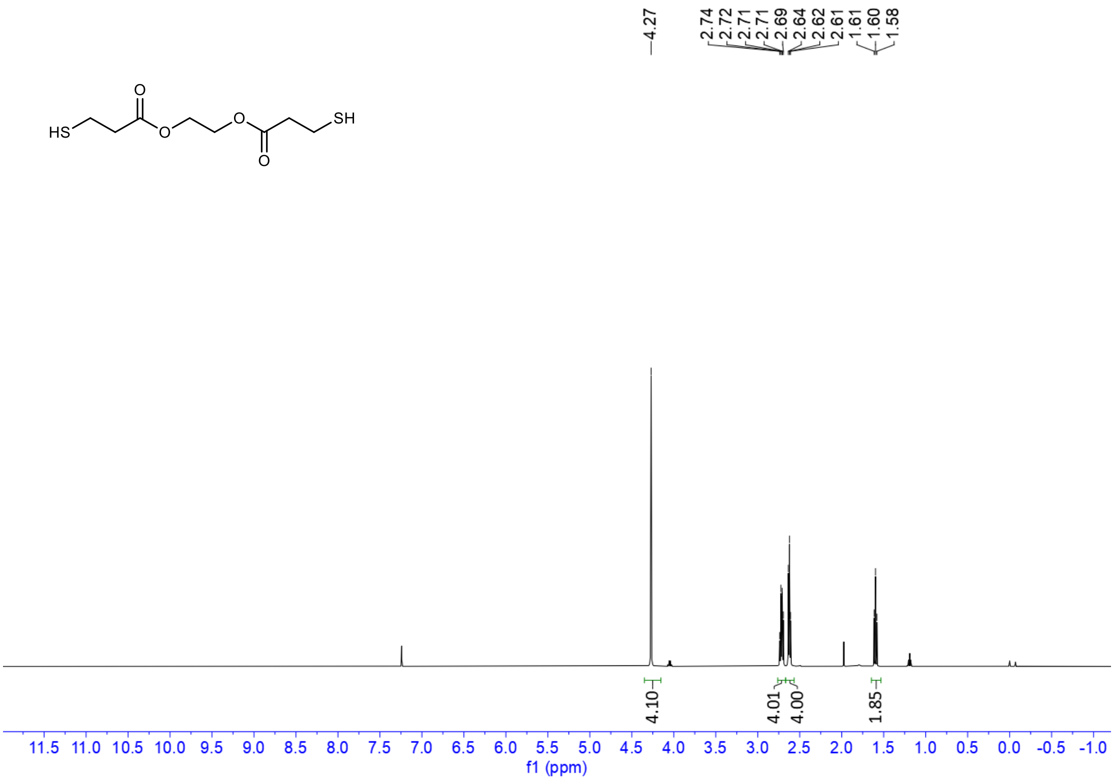

Supplementary Figure 58 | ^{13}C NMR (126 MHz, 298K, $CDCl_3$) of Ethane-1,2-diyl bis(3-mercaptopropanoate) **(5a)**

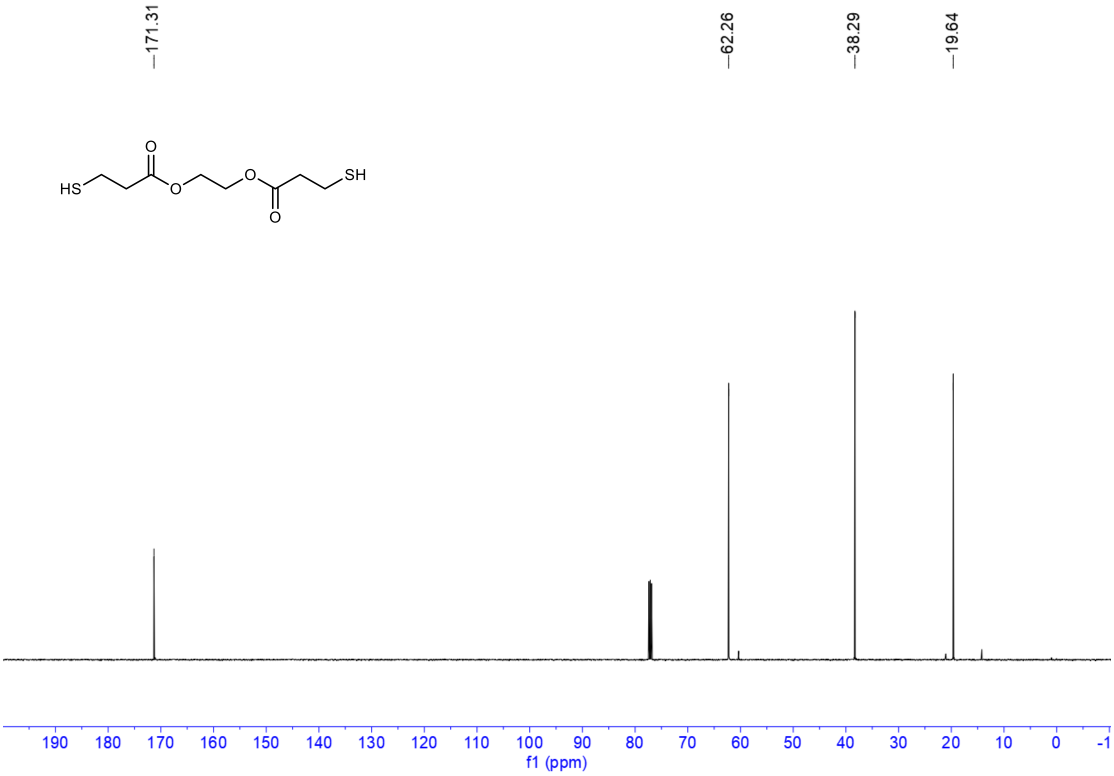

Supplementary Figure 59 | ^{1}H NMR (500 MHz, 298K, $CDCl_3$) of Propane-1,3-diyl bis(3-mercaptopropanoate) **(6a)**

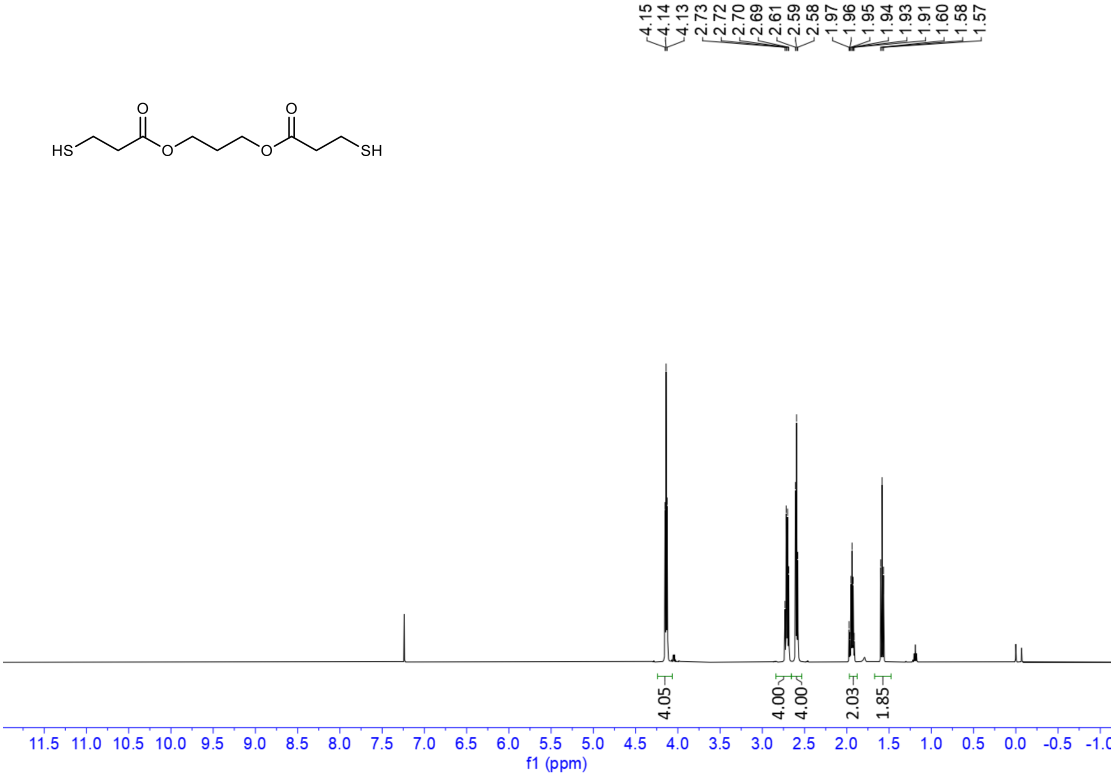

Supplementary Figure 60 | ^{13}C NMR (126 MHz, 298K, $CDCl_3$) of Propane-1,3-diyl bis(3-mercaptopropanoate) **(6a)**

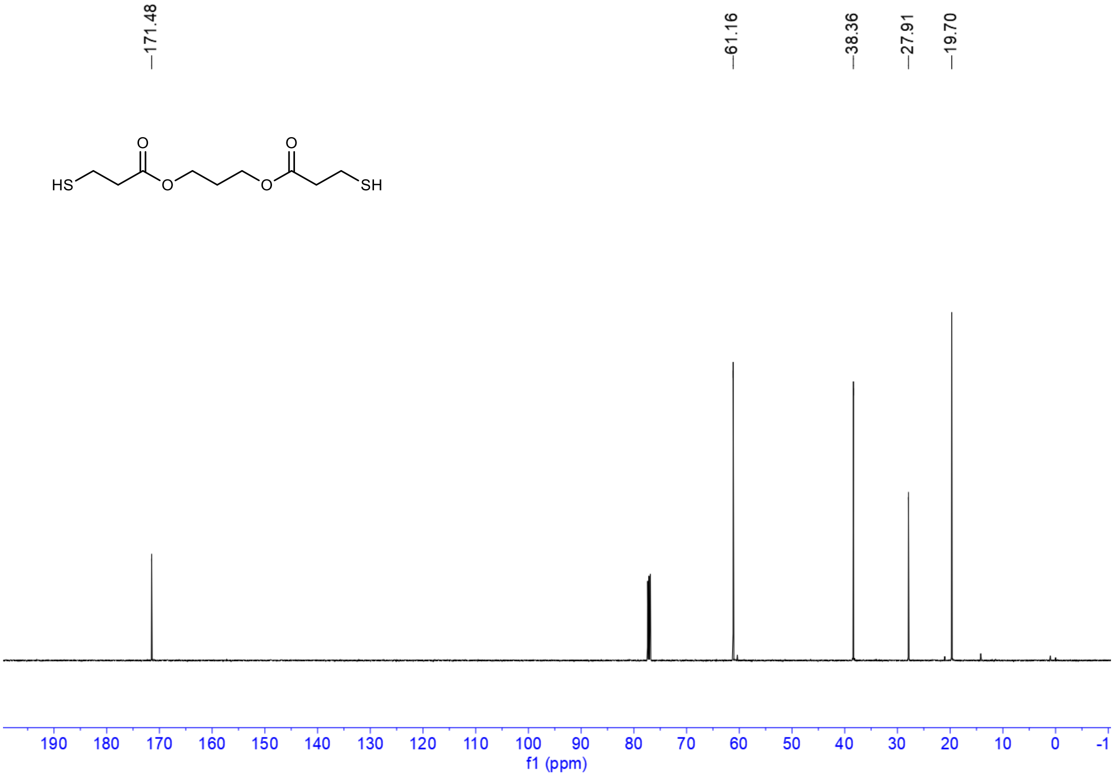

Supplementary Figure 61 | ^{1}H NMR (500 MHz, 298K, $CDCl_3$) of 2-Methylpropane-1,3-diyl bis(3-mercaptopropanoate) **(7a)**

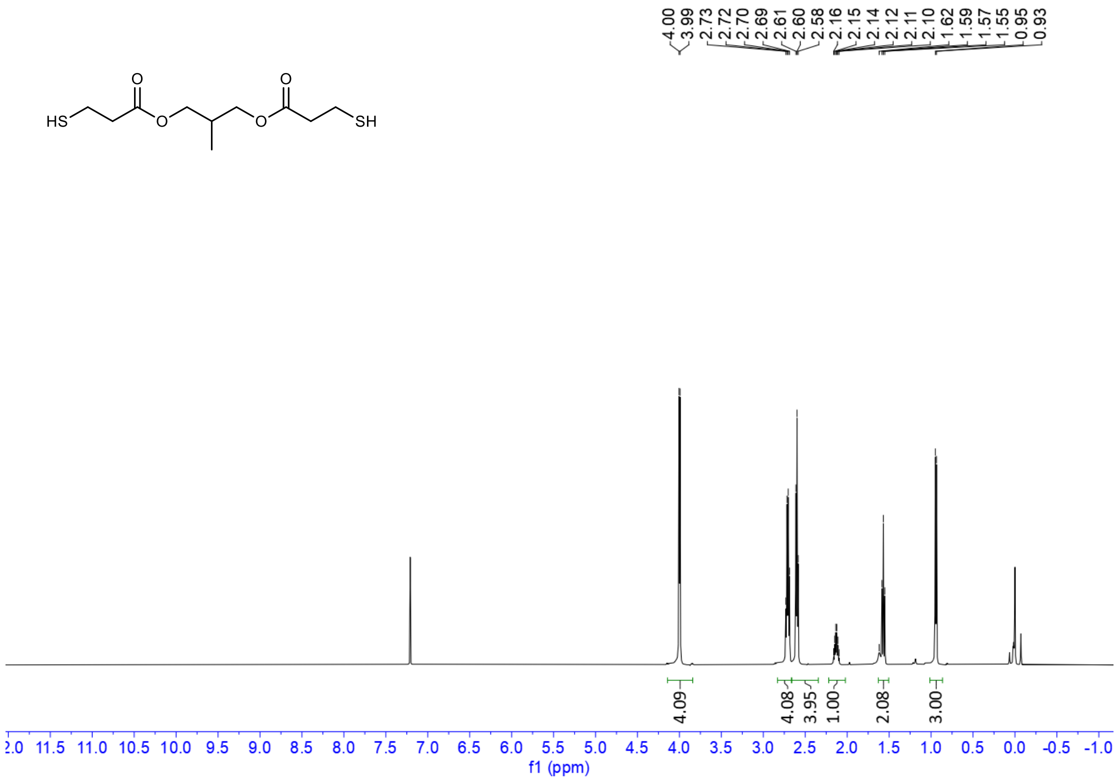

Supplementary Figure 62 | ^{13}C NMR (126 MHz, 298K, $CDCl_3$) of 2-Methylpropane-1,3-diyl bis(3-mercaptopropanoate) **(7a)**

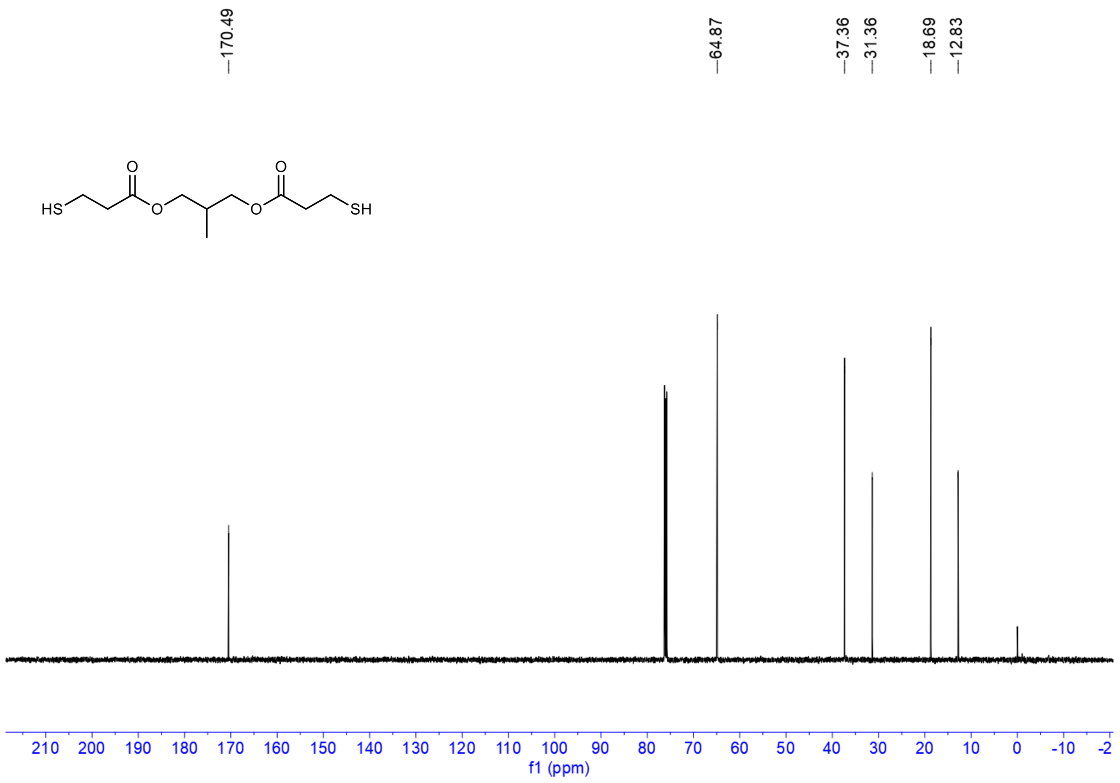

Supplementary Figure 63 | ^{1}H NMR (500 MHz, 298K, $CDCl_3$) of (±)-Pentane-2,4-diyl bis(3-mercaptopropanoate) **(8a)**

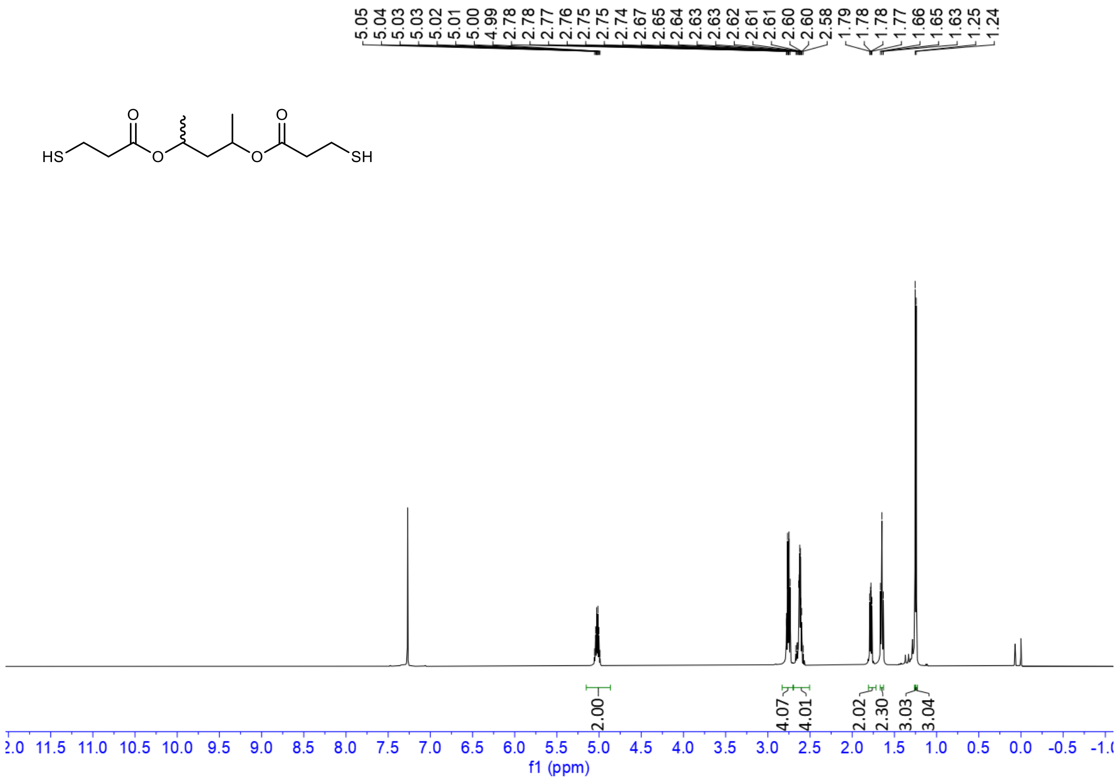

Supplementary Figure 64 | ^{13}C NMR (126 MHz, 298K, $CDCl_3$) of (±)-Pentane-2,4-diyl bis(3-mercaptopropanoate) **(8a)**

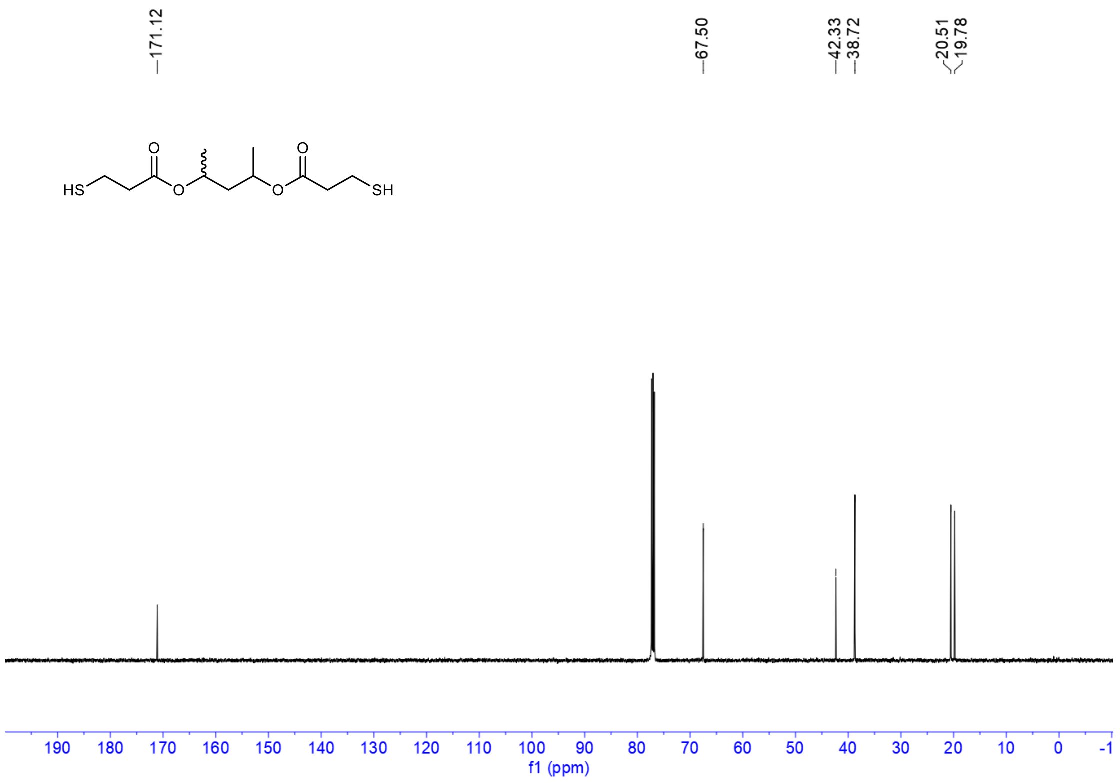

Supplementary Figure 65 | ^{1}H NMR (500 MHz, 298K, $CDCl_3$) of 2,2-Dimethylpropane-1,3-diyl bis(3-mercaptopropanoate) **(9a)**

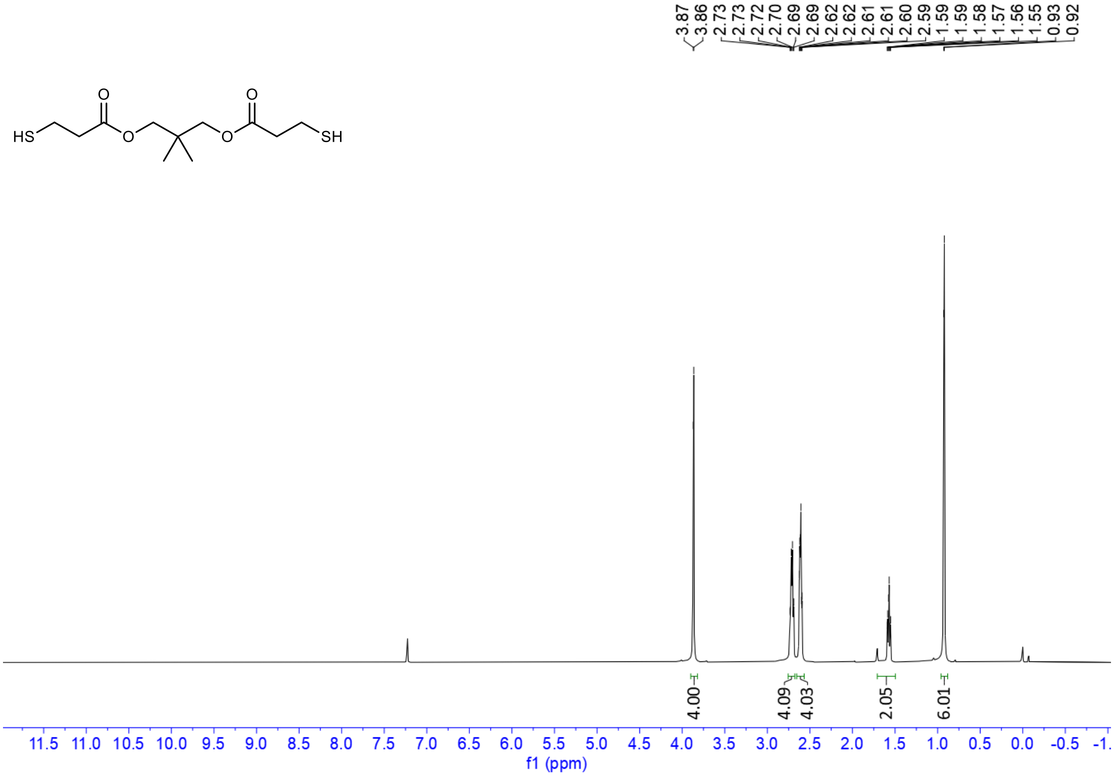

Supplementary Figure 66 | ^{13}C NMR (126 MHz, 298K, $CDCl_3$) of 2,2-Dimethylpropane-1,3-diyl bis(3-mercaptopropanoate) **(9a)**

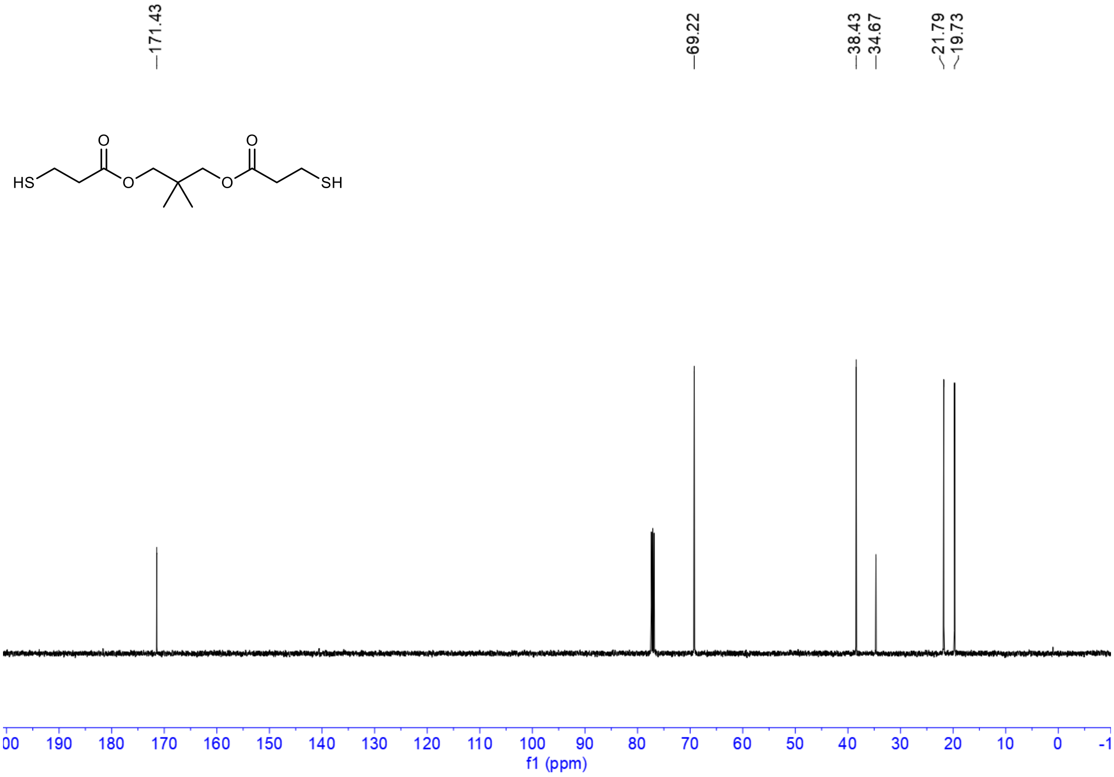

Supplementary Figure 67 | ^{1}H NMR (500 MHz, 298K, $CDCl_3$) of Butane-1,4-diyl bis(3-mercaptopropanoate) **(10a)**

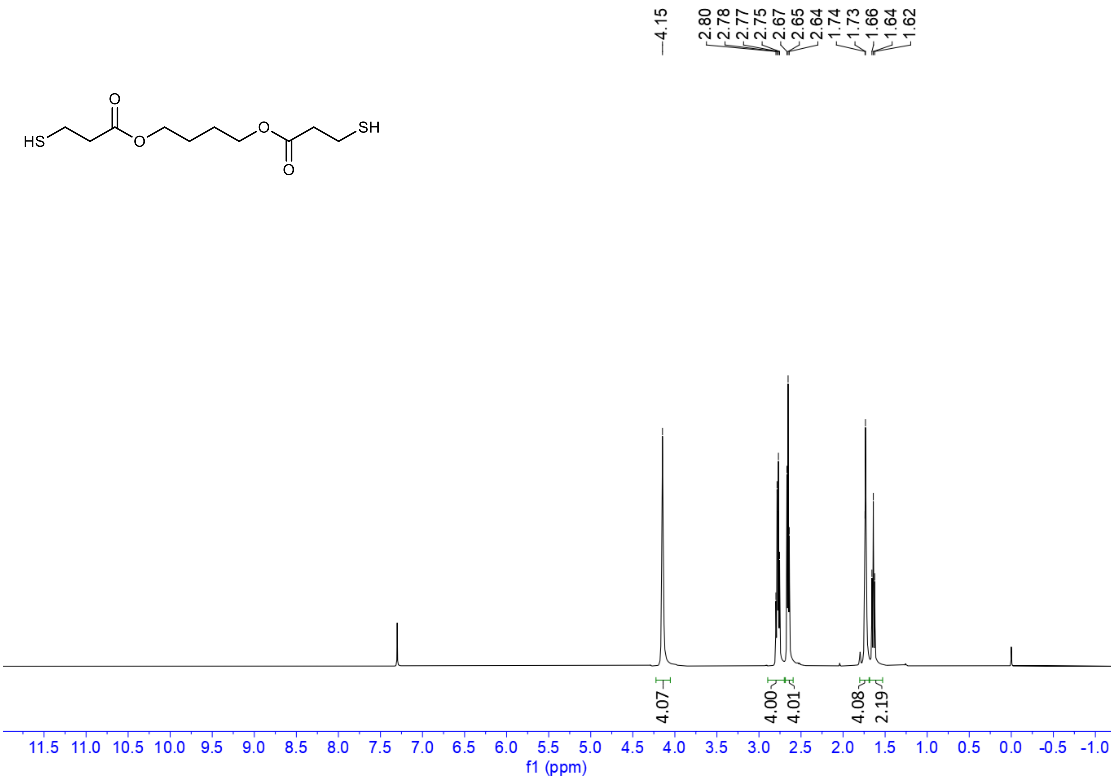

Supplementary Figure 68 | ^{13}C NMR (126 MHz, 298K, $CDCl_3$) of Butane-1,4-diyl bis(3-mercaptopropanoate) **(10a)**

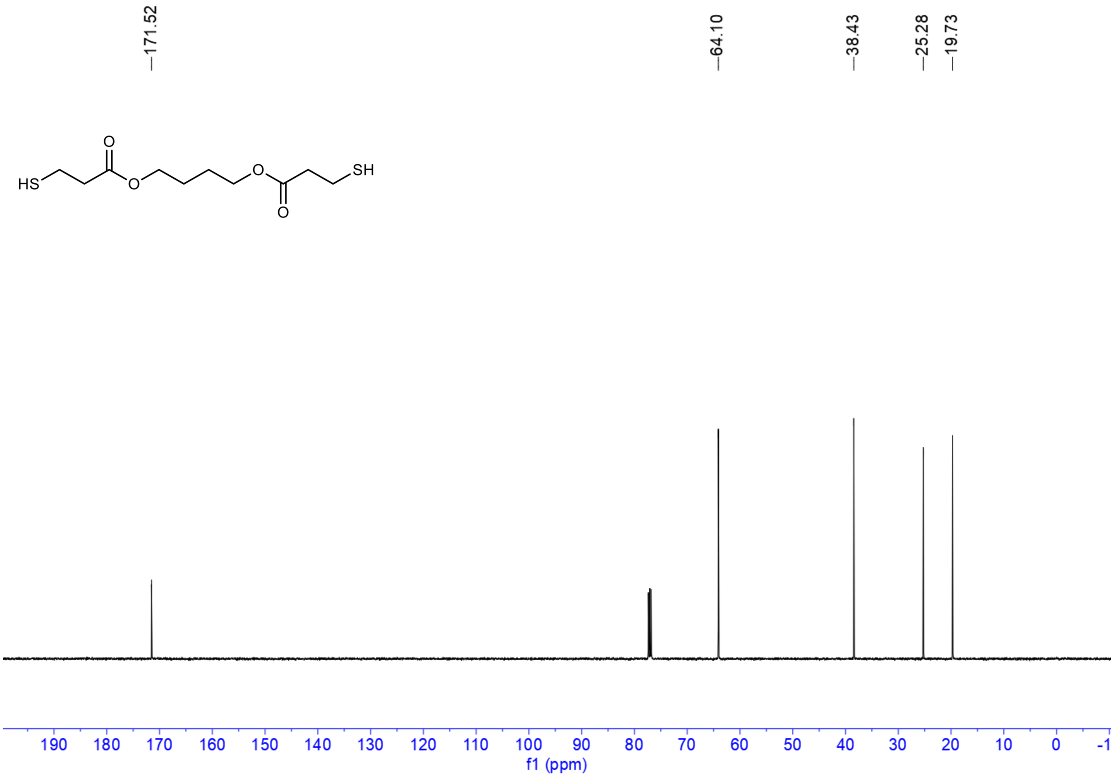

Supplementary Figure 69 | ^{1}H NMR (500 MHz, 298K, $CDCl_3$) of Pentane-1,5-diyl bis(3-mercaptopropanoate) **(11a)**

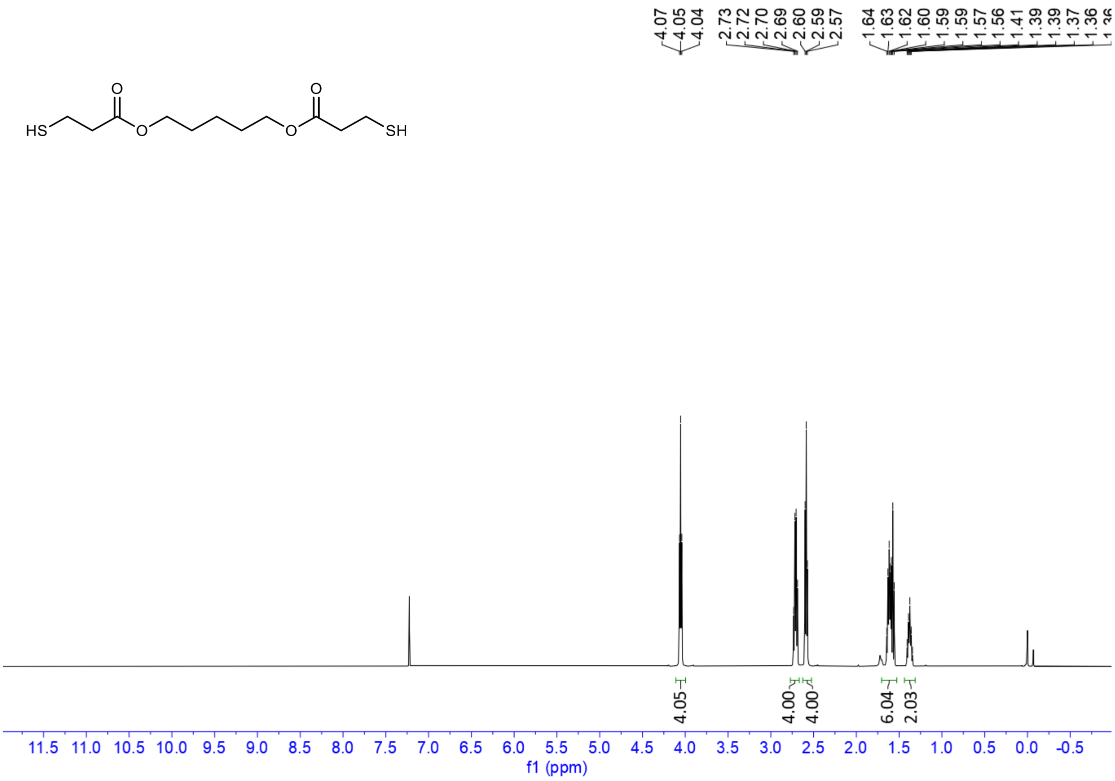

Supplementary Figure 70 | ^{13}C NMR (126 MHz, 298K, $CDCl_3$) of Pentane-1,5-diyl bis(3-mercaptopropanoate) **(11a)**

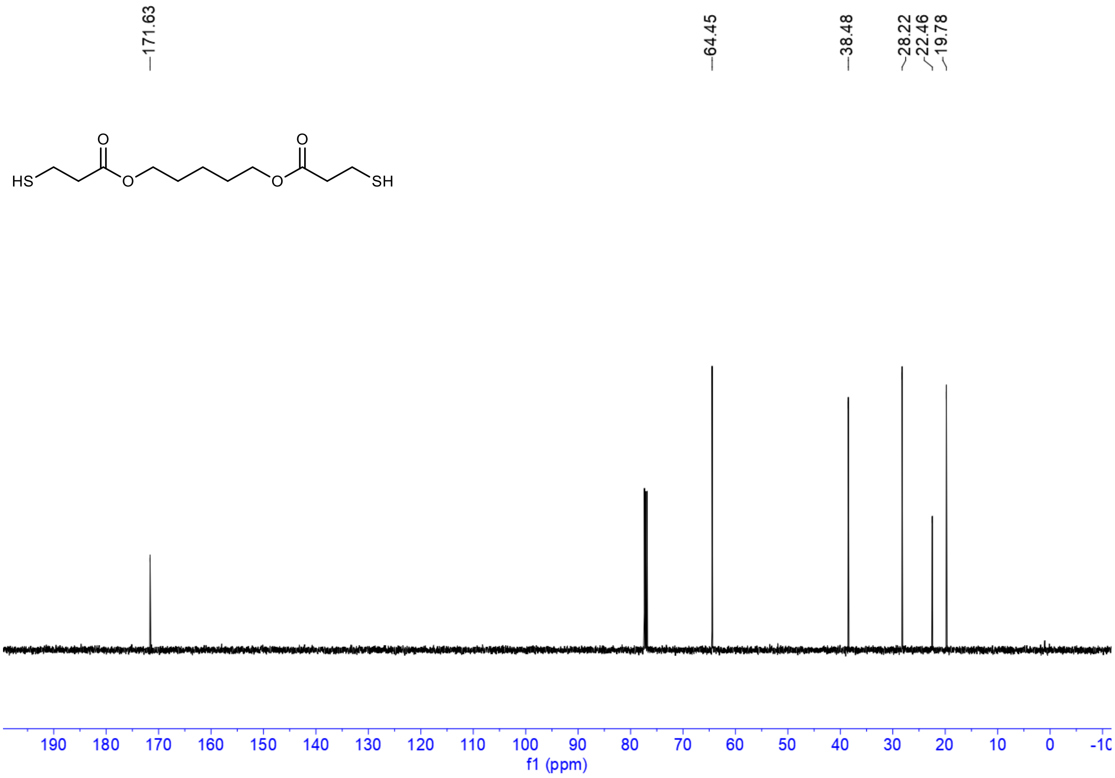

Supplementary Figure 71 | ^{1}H NMR (500 MHz, 298K, $CDCl_3$) of Hexane-1,6-diyl bis(3-mercaptopropanoate) **(12a)**

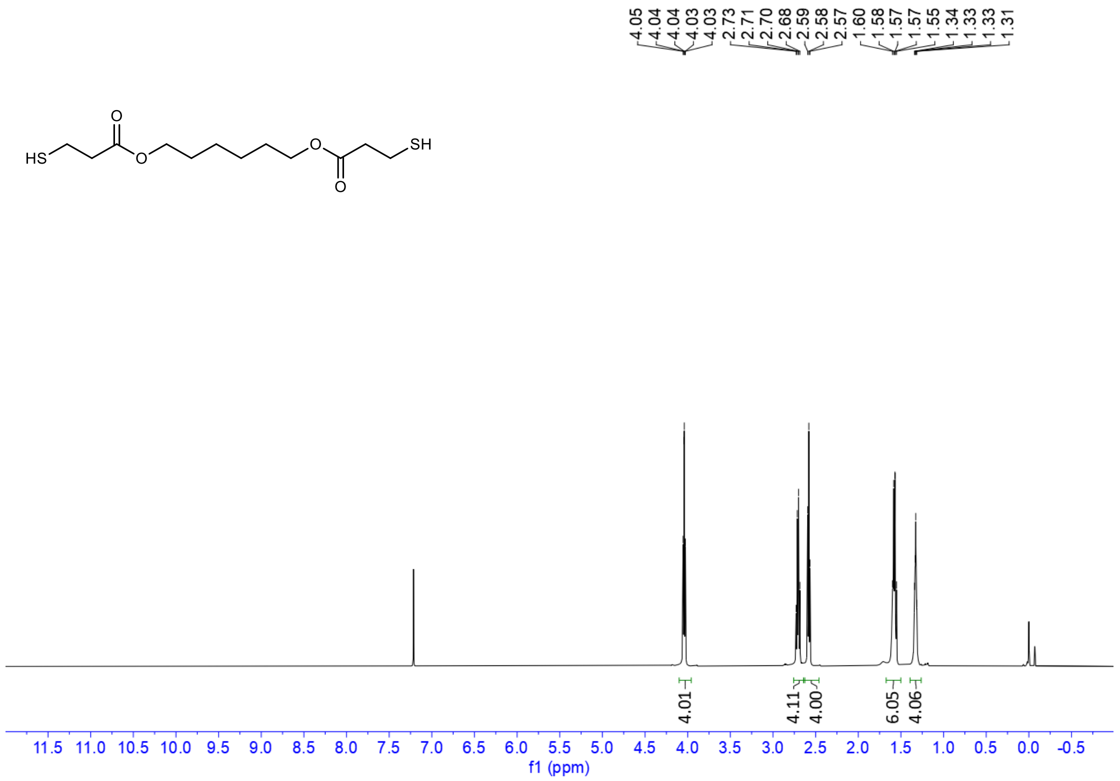

Supplementary Figure 72 | ^{13}C NMR (126 MHz, 298K, $CDCl_3$) of Hexane-1,6-diyl bis(3-mercaptopropanoate) **(12a)**

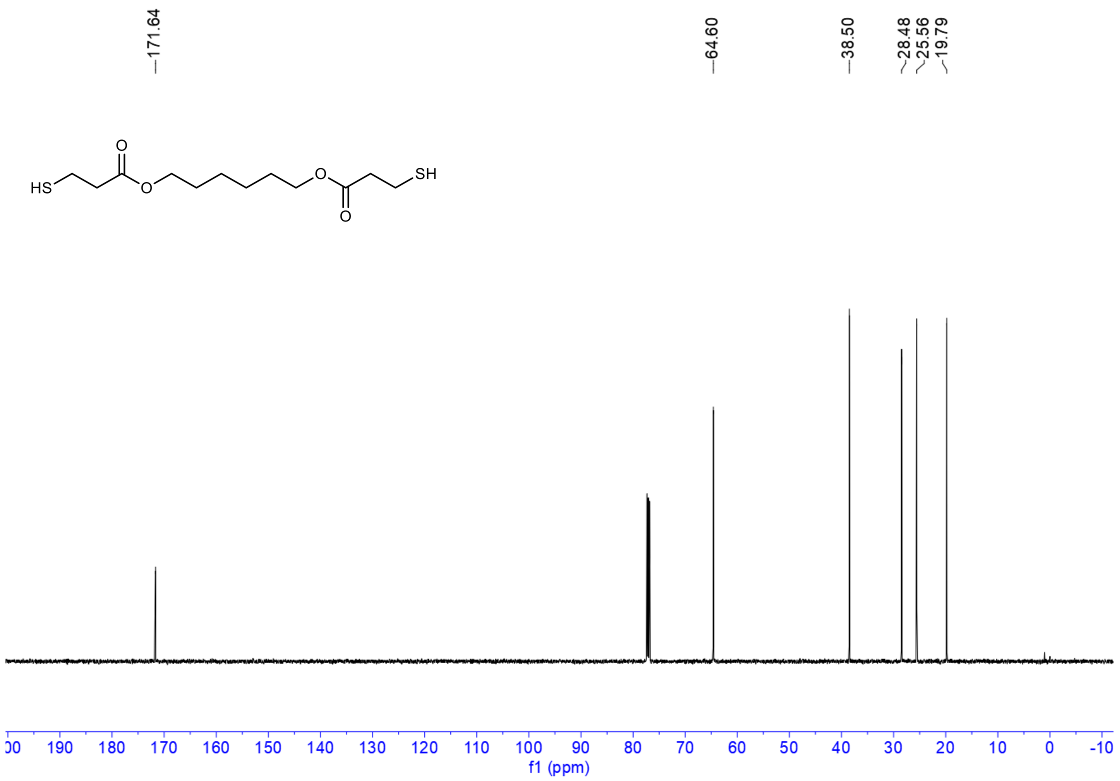

Supplementary Figure 73 | ^{1}H NMR (500 MHz, 298K, $CDCl_3$) of Heptane-1,7-diyl bis(3-mercaptopropanoate) **(14a)**

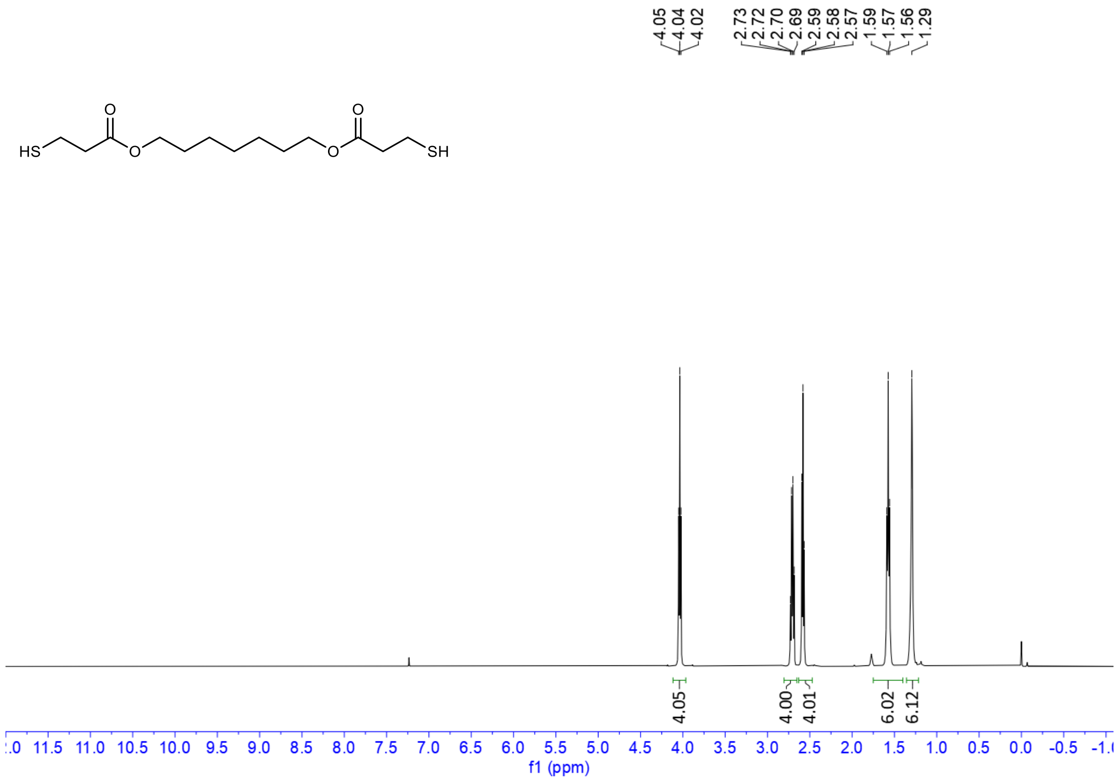

Supplementary Figure 74 | ^{13}C NMR (126 MHz, 298K, $CDCl_3$) of Heptane-1,7-diyl bis(3-mercaptopropanoate) **(14a)**

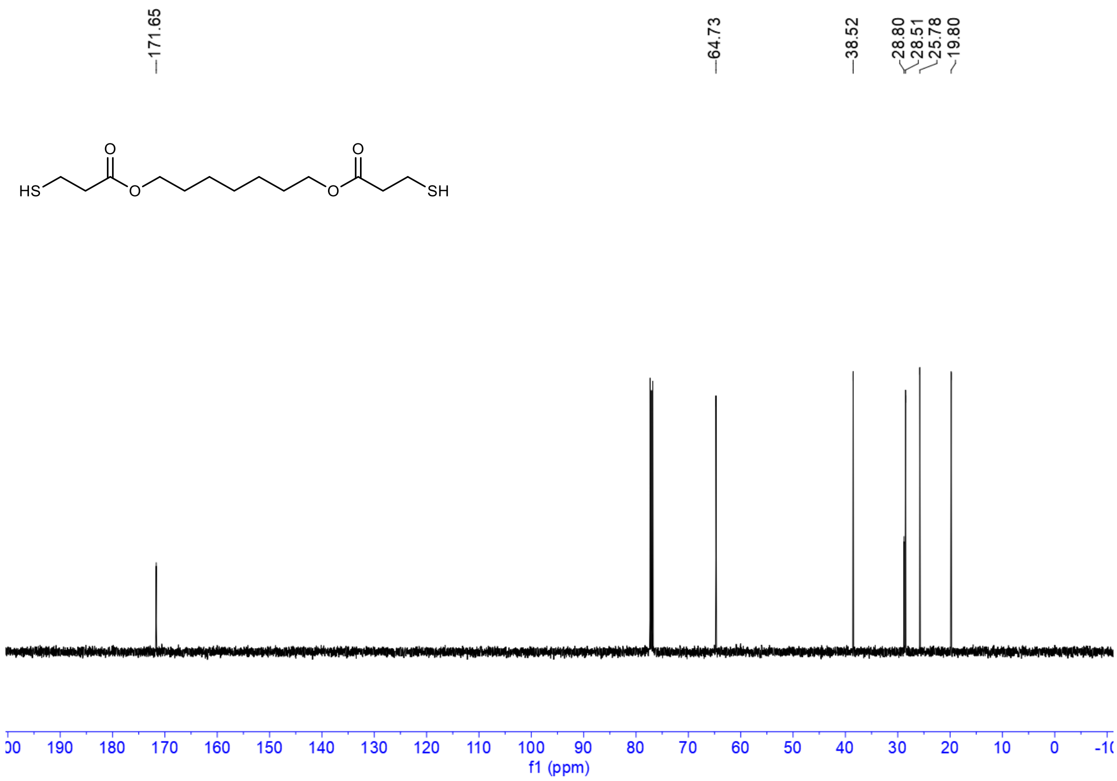

Supplementary Figure 75 | ^{1}H NMR (500 MHz, 298K, $CDCl_3$) of Octane-1,8-diyl bis(3-mercaptopropanoate) **(15a)**

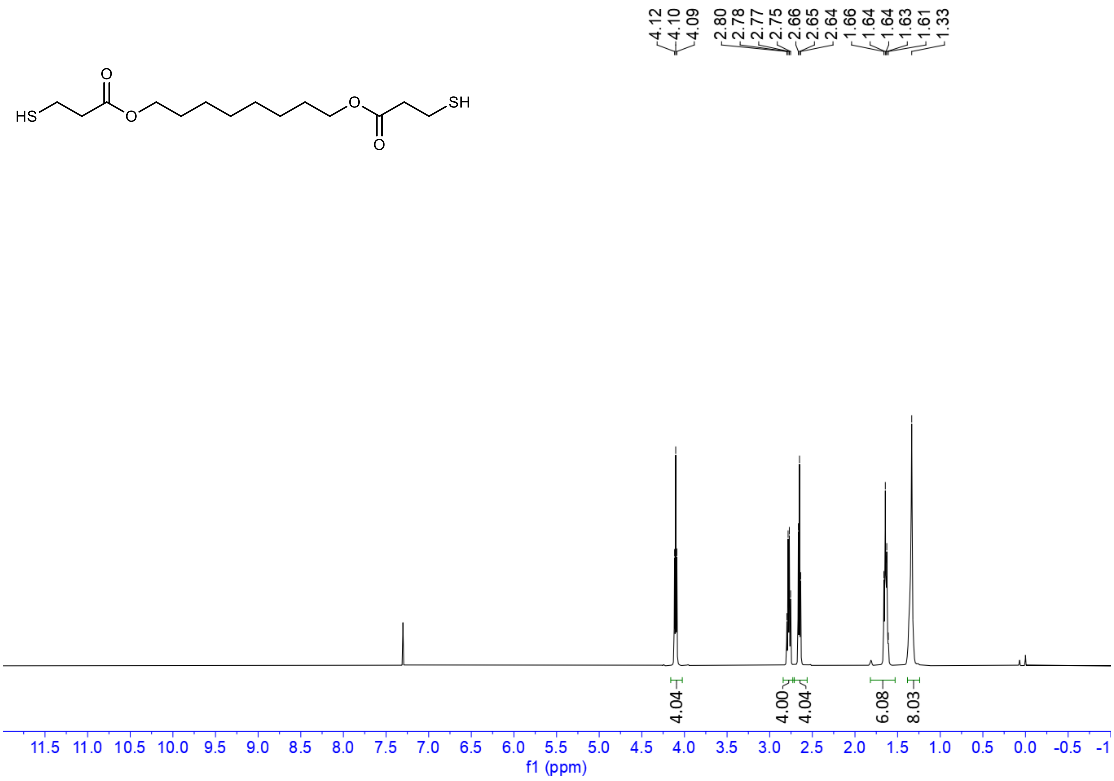

Supplementary Figure 76 | ^{13}C NMR (126 MHz, 298K, $CDCl_3$) of Octane-1,8-diyl bis(3-mercaptopropanoate) **(15a)**

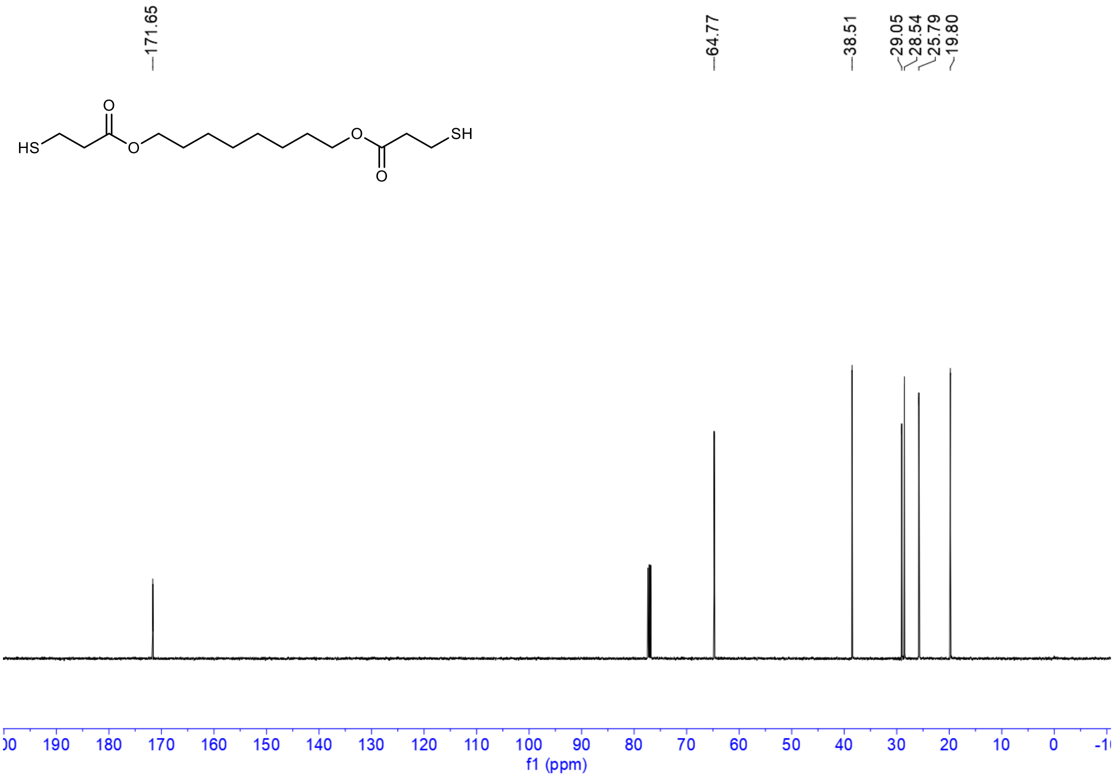

Supplementary Figure 77 | ^{1}H NMR (500 MHz, 298K, $CDCl_3$) of Nonane-1,9-diyl bis(3-mercaptopropanoate) **(16a)**

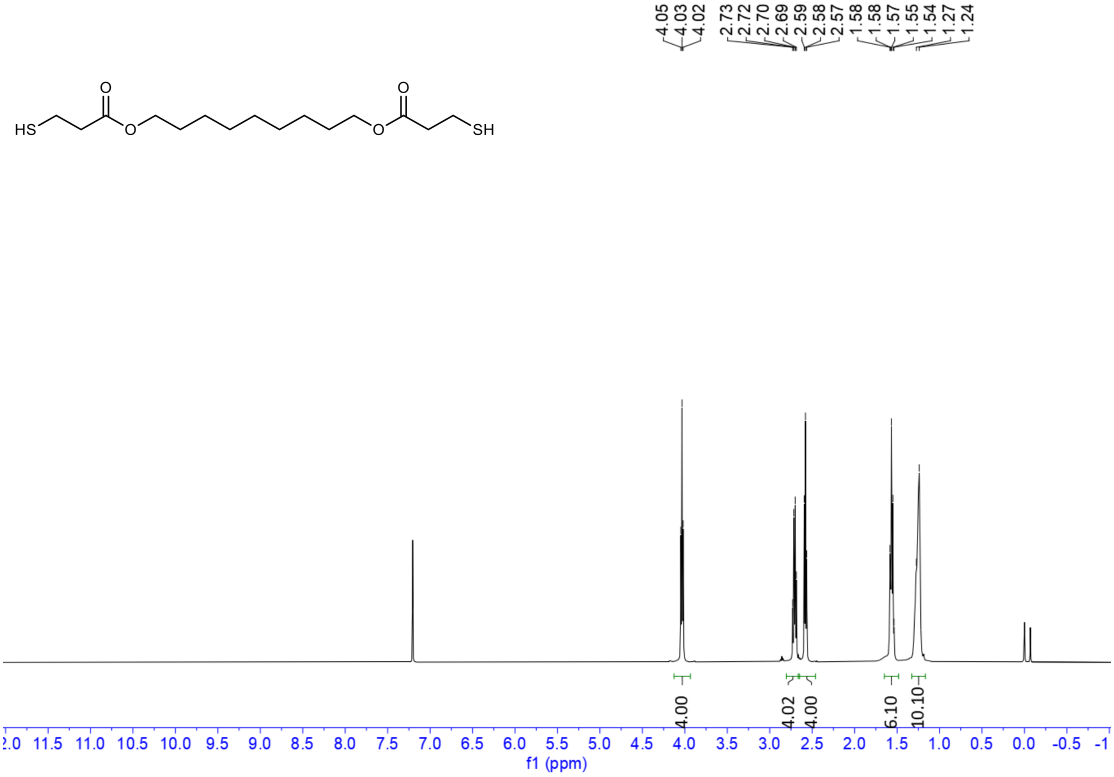

Supplementary Figure 78 | ^{13}C NMR (126 MHz, 298K, $CDCl_3$) of Nonane-1,9-diyl bis(3-mercaptopropanoate) **(16a)**

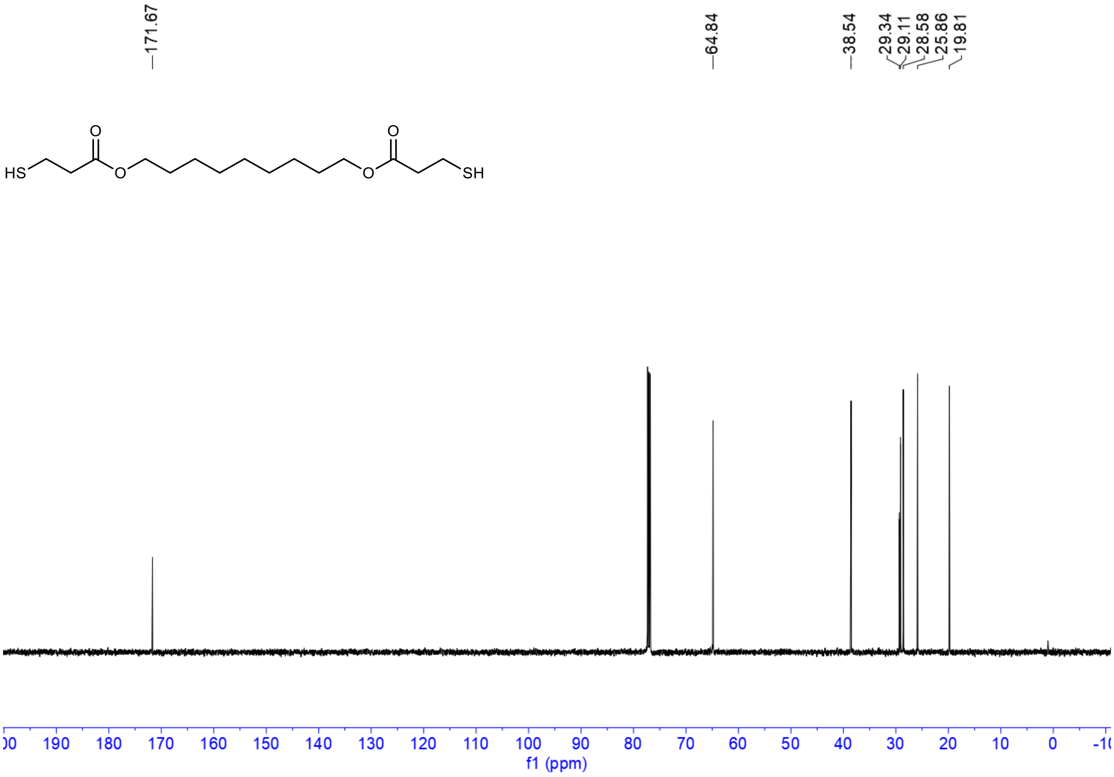

Supplementary Figure 79 | ^{1}H NMR (500 MHz, 298K, $CDCl_3$) of Decane-1,10-diyl bis(3-mercaptopropanoate) **(17a)**

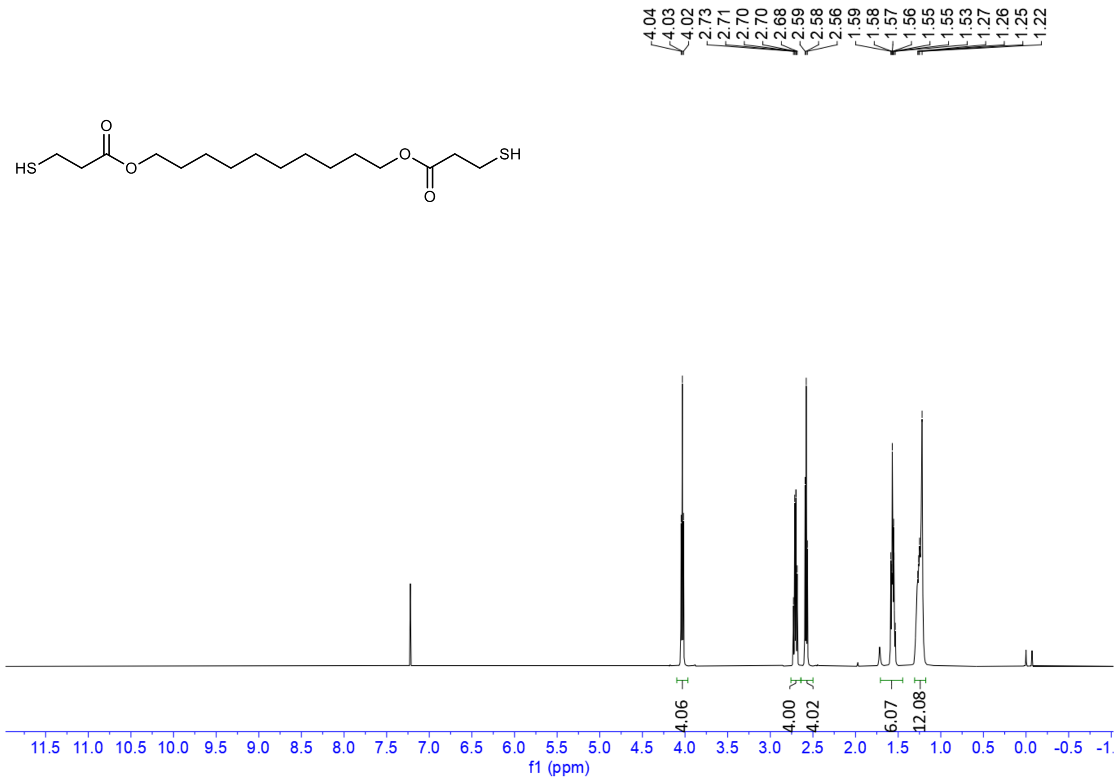

Supplementary Figure 80 | ^{13}C NMR (126 MHz, 298K, $CDCl_3$) of Decane-1,10-diyl bis(3-mercaptopropanoate) **(17a)**

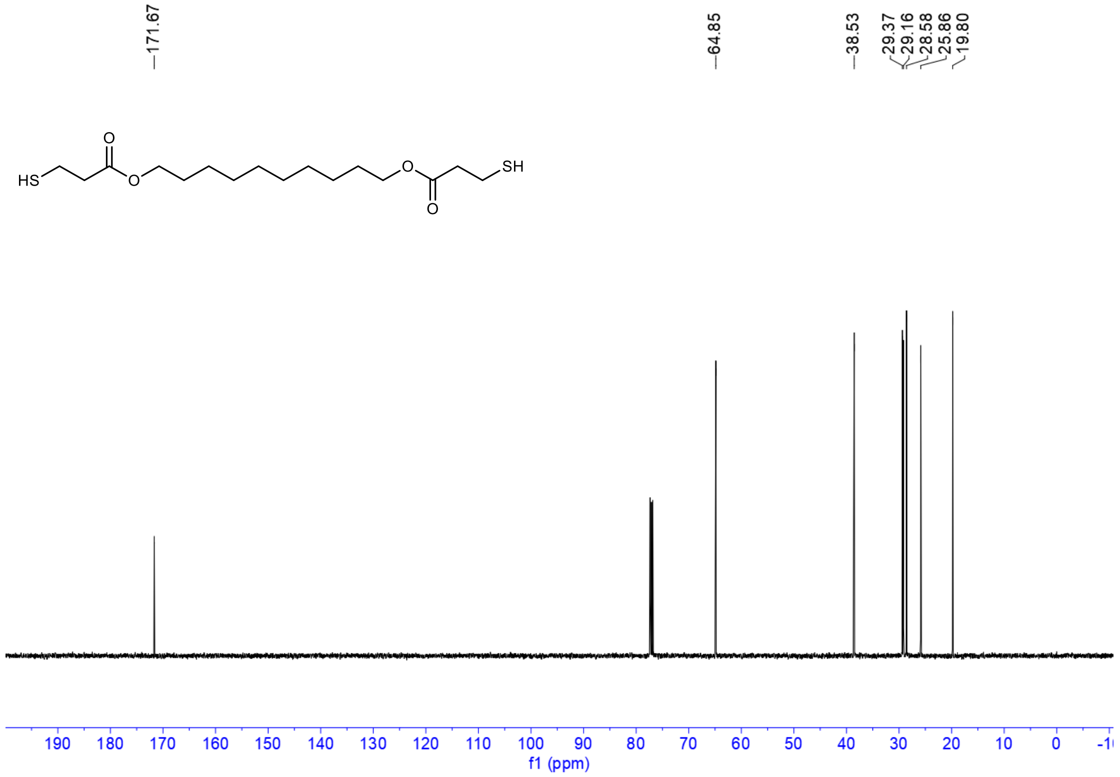

Supplementary Figure 81 | ^{1}H NMR (500 MHz, 298K, $CDCl_3$) of Dodecane-1,12-diyl bis(3-mercaptopropanoate) **(18a)**

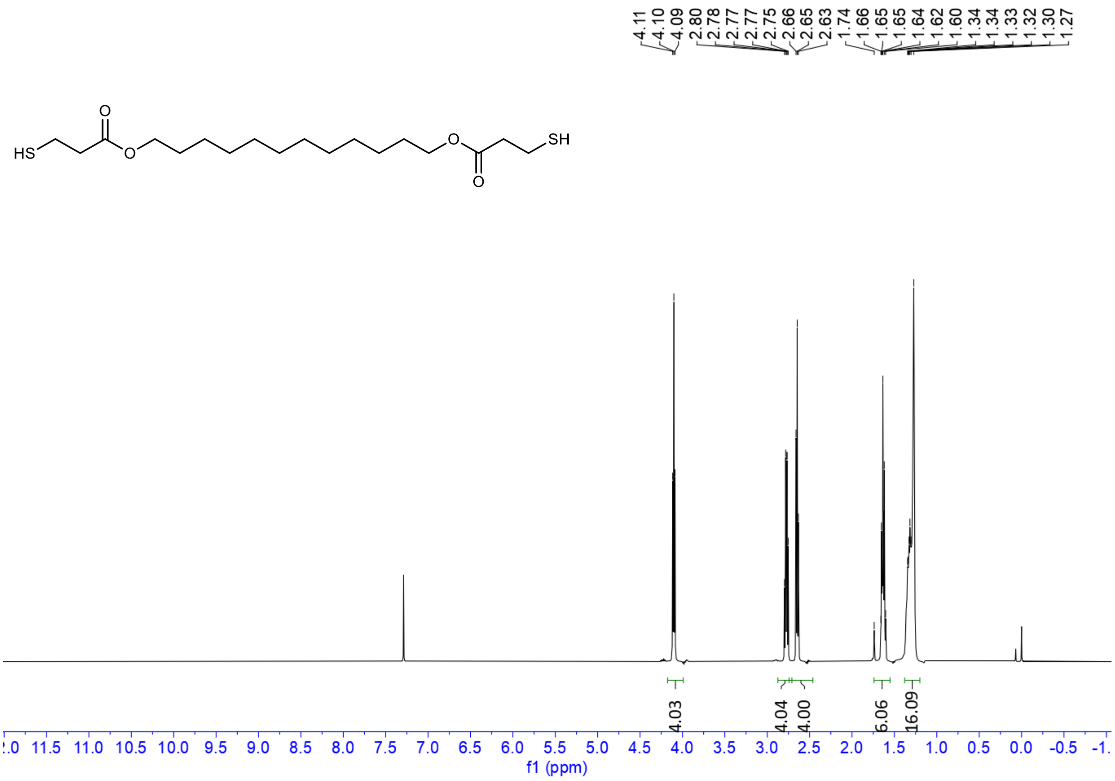

Supplementary Figure 82 | ^{13}C NMR (126 MHz, 298K, $CDCl_3$) of Dodecane-1,12-diyl bis(3-mercaptopropanoate) **(18a)**

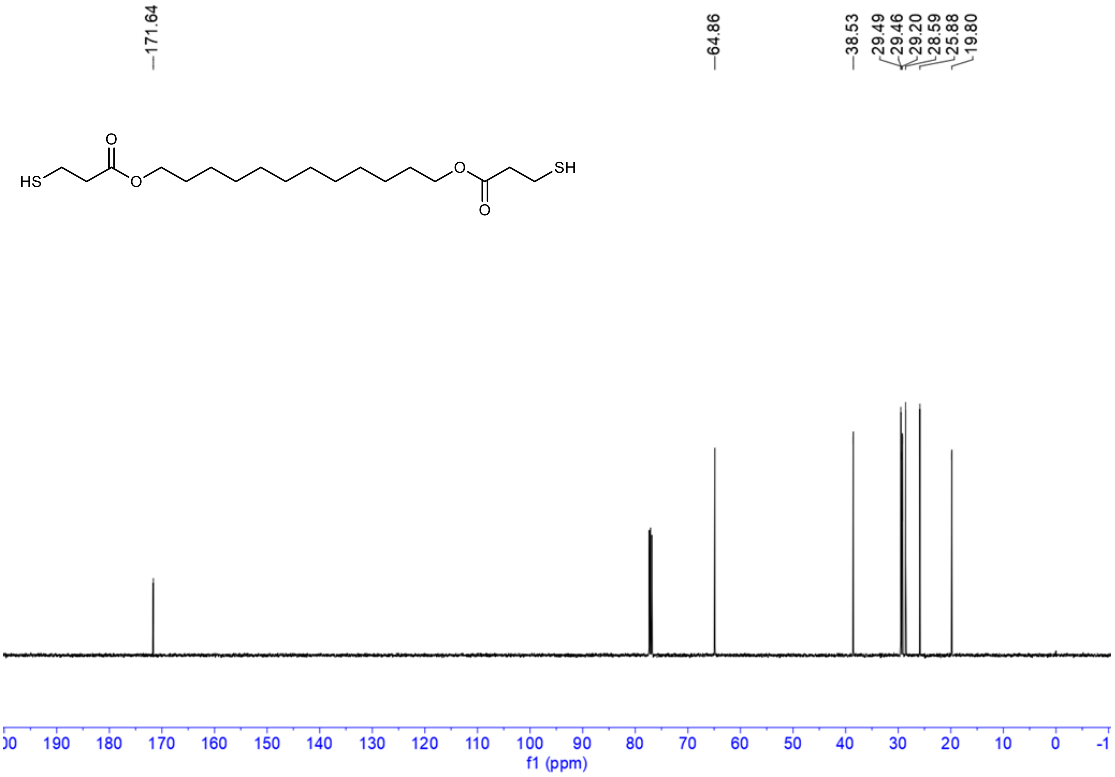

Supplementary Figure 83 | ^{1}H NMR (500 MHz, 298K, $CDCl_3$) of Bis(6-mercaptohexyl) 2,2'-oxydiacetate **(22a)**

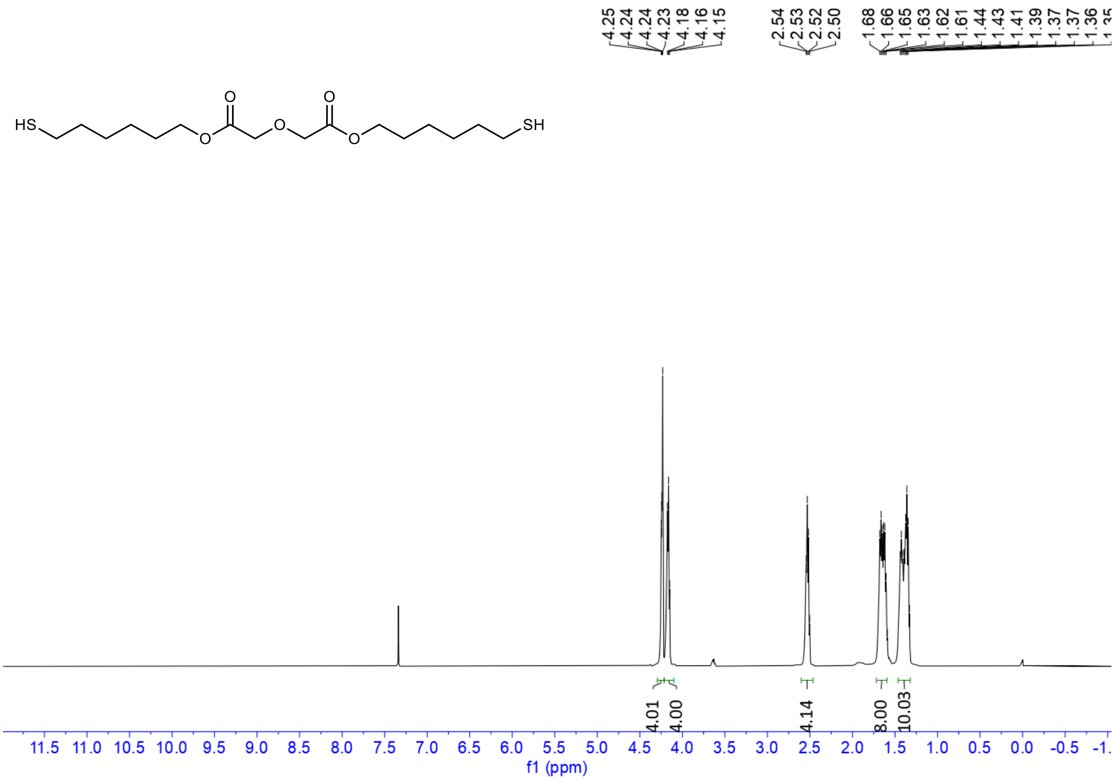

Supplementary Figure 84 | ^{13}C NMR (126 MHz, 298K, $CDCl_3$) of Bis(6-mercaptohexyl) 2,2'-oxydiacetate **(22a)**

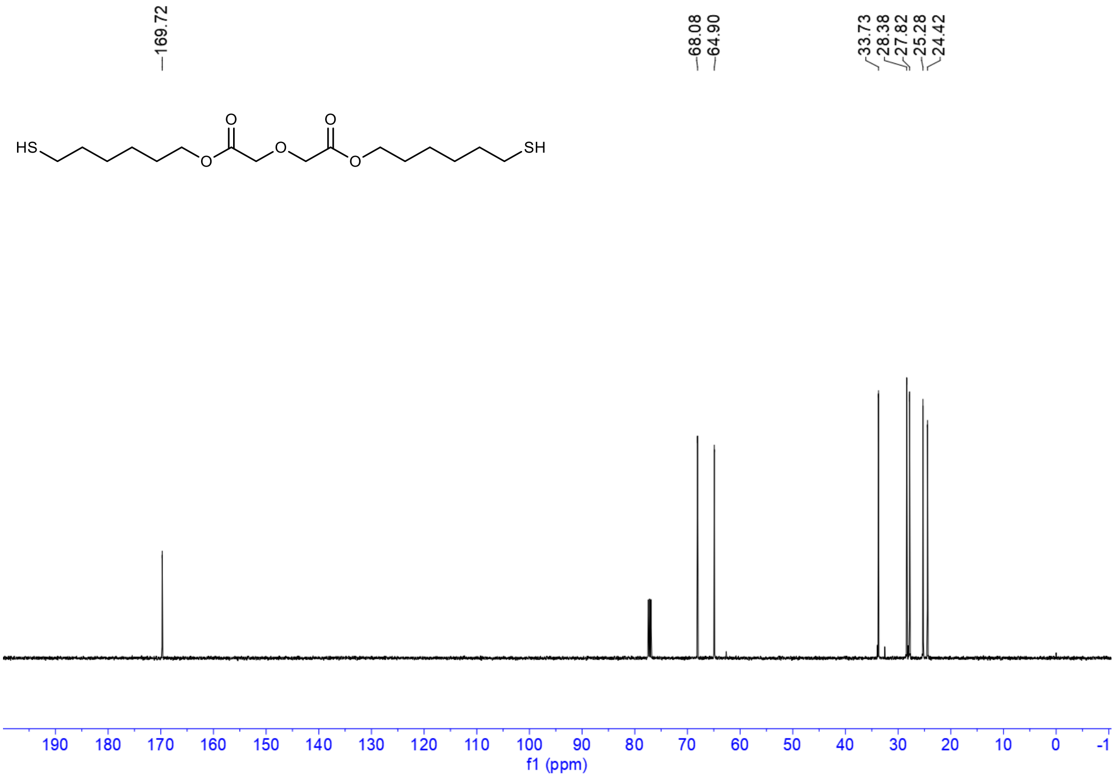

Supplementary Figure 85 | ^{1}H NMR (500 MHz, 298K, $CDCl_3$) of Bis(6-mercaptohexyl) 2,2,3,3-tetrafluorosuccinate **(20a)**

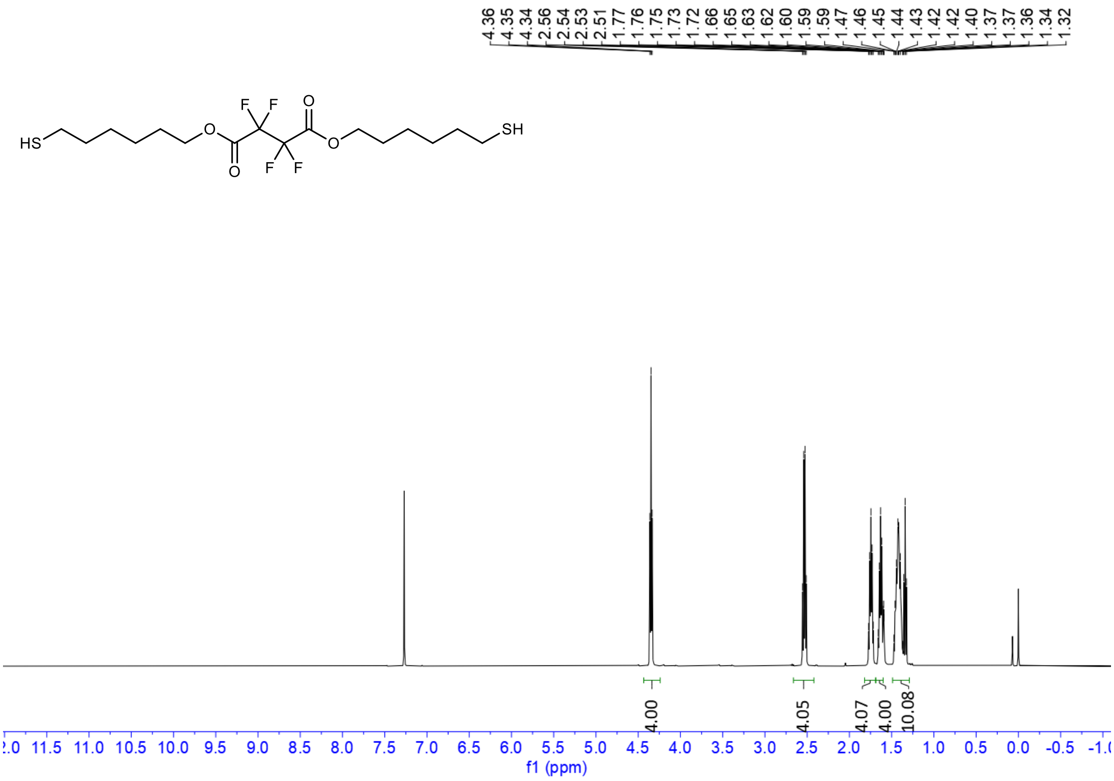

Supplementary Figure 86 | ^{13}C NMR (126 MHz, 298K, $CDCl_3$) of Bis(6-mercaptohexyl) 2,2,3,3-tetrafluorosuccinate **(20a)**

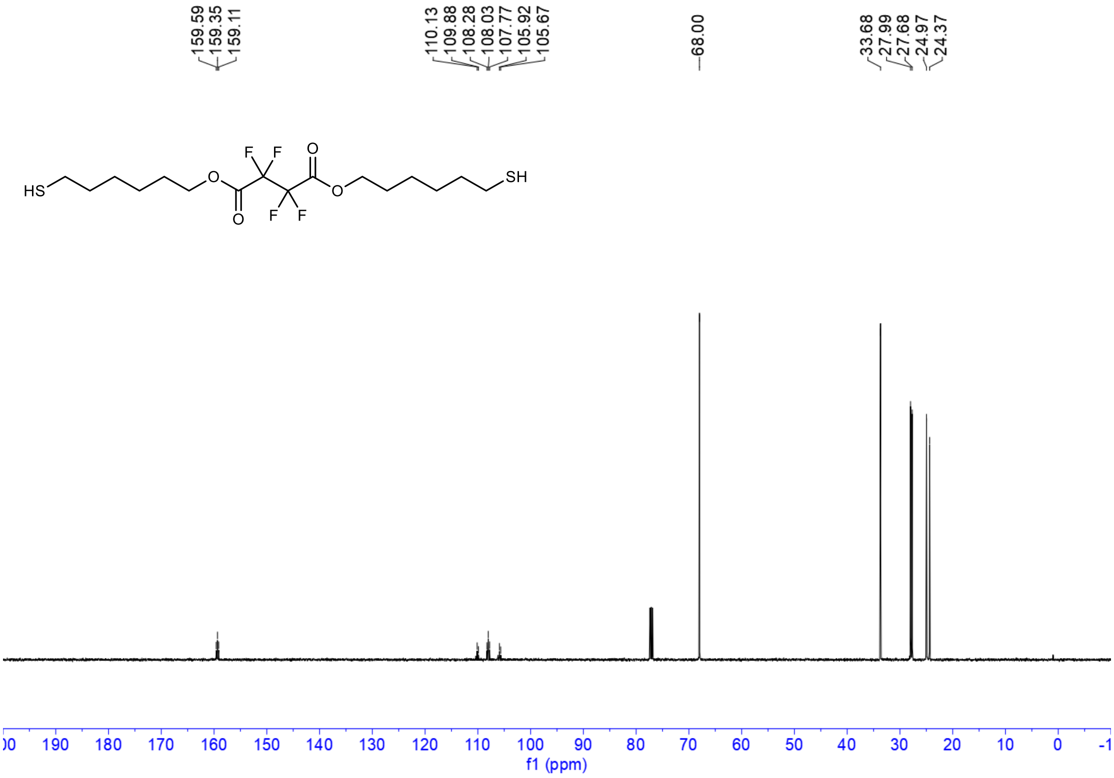

Supplementary Figure 87 | ^{19}F NMR (471 MHz, 298K, $CDCl_3$) of Bis(6-mercaptohexyl) 2,2,3,3-tetrafluorosuccinate **(20a)**

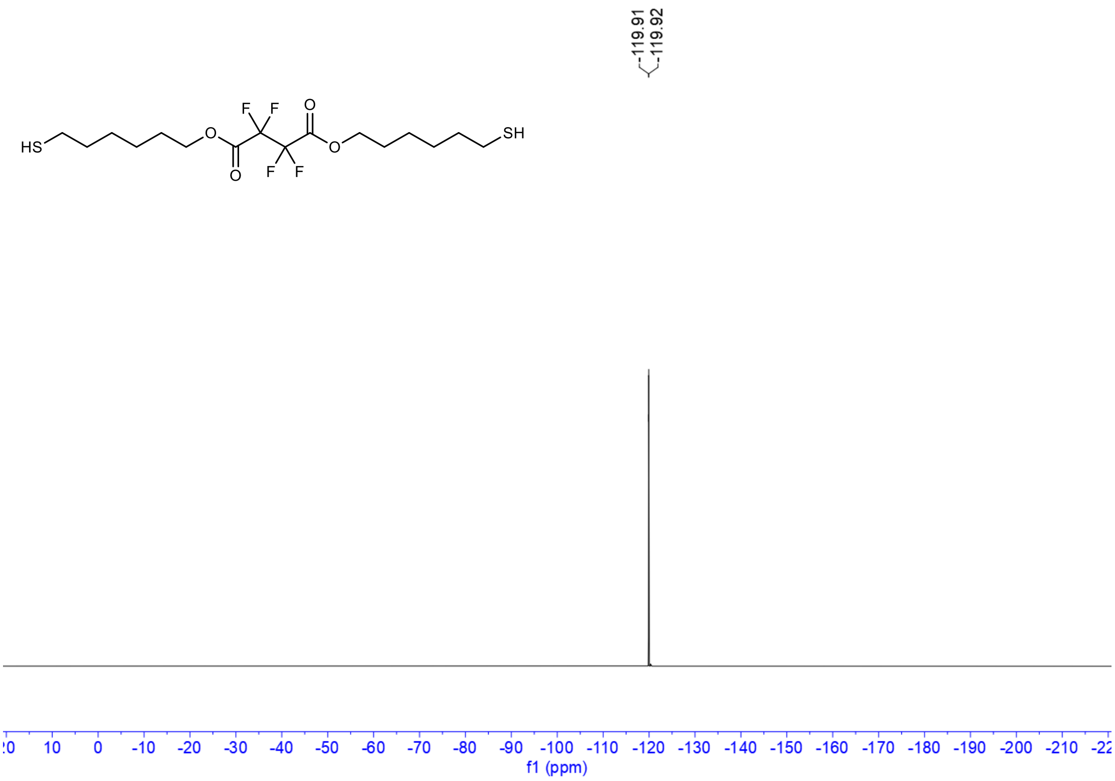

Supplementary Figure 88 | ^{1}H NMR (500 MHz, 298K, $CDCl_3$) of Bis(6-mercaptohexyl) 2,2,3,3-tetrafluorosuccinate **(21a)**

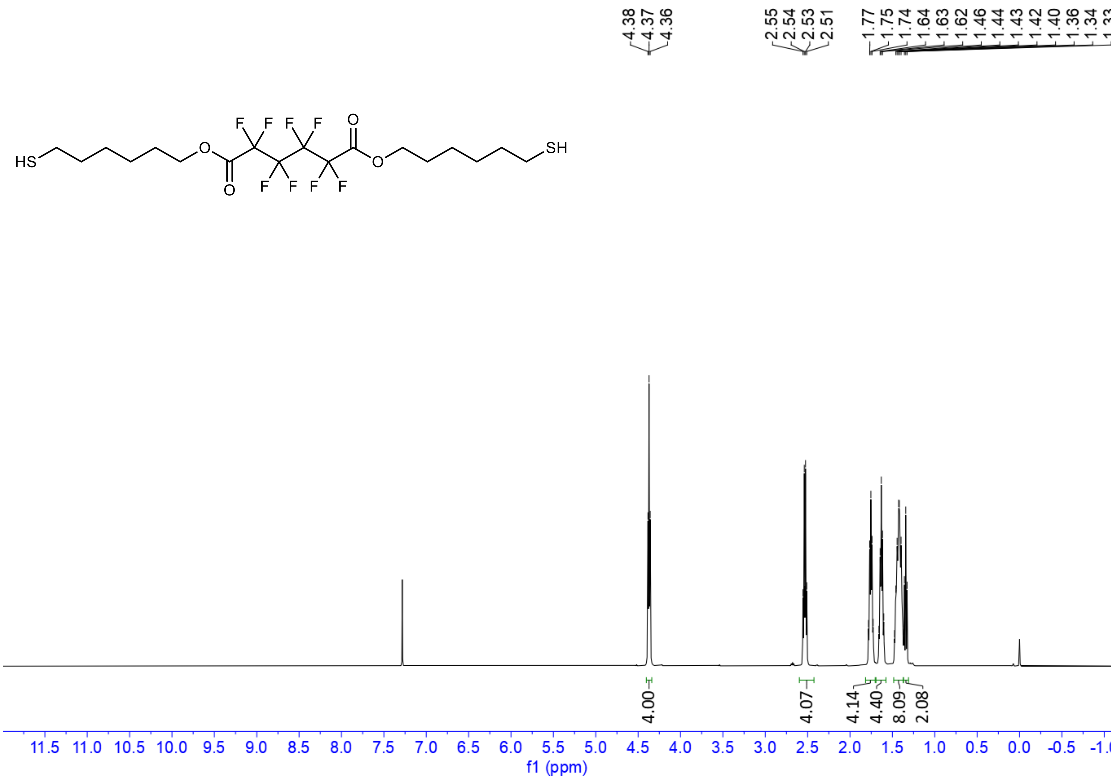

Supplementary Figure 89 | ^{13}C NMR (126 MHz, 298K, $CDCl_3$) of Bis(6-mercaptohexyl) 2,2,3,3-tetrafluorosuccinate **(21a)**

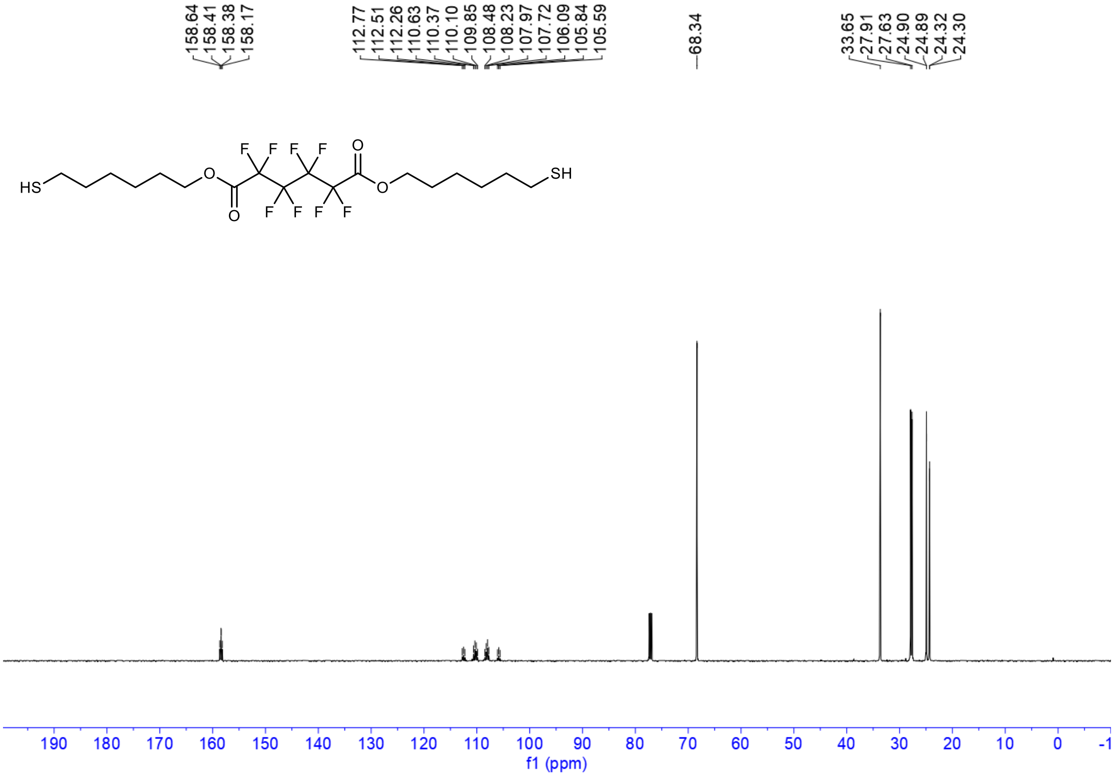

Supplementary Figure 90 | ^{19}F NMR (471 MHz, 298K, $CDCl_3$) of Bis(6-mercaptohexyl) 2,2,3,3-tetrafluorosuccinate **(21a)**

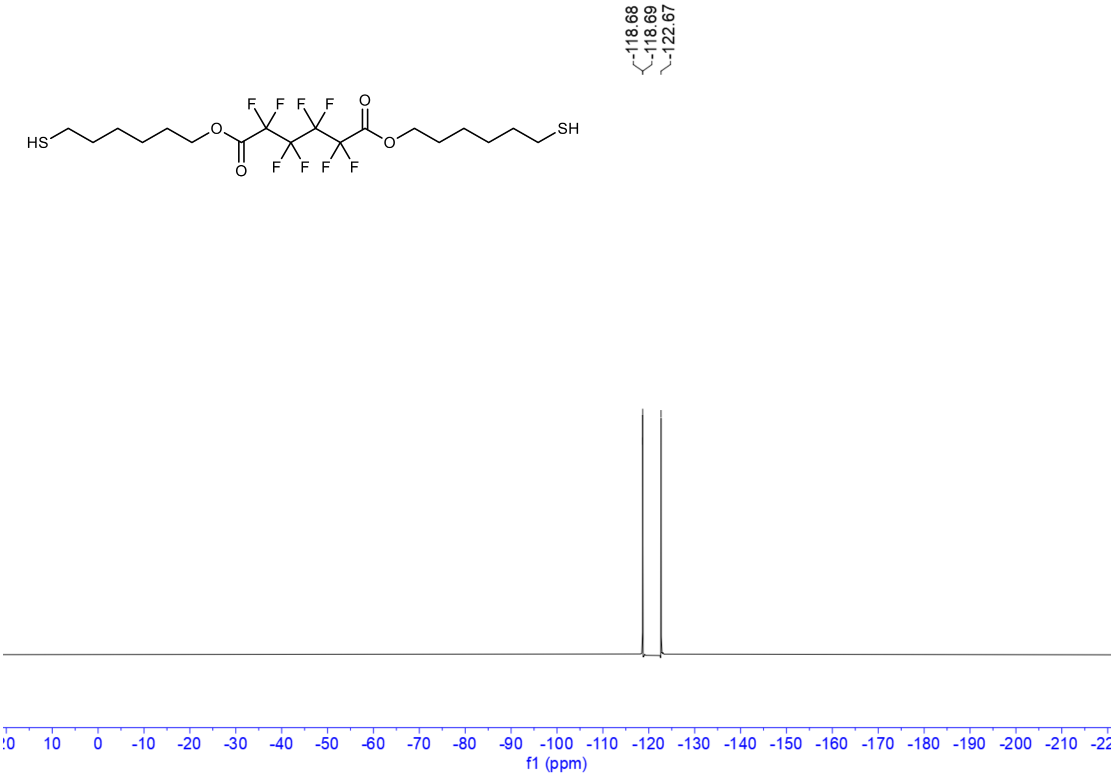

Supplementary Figure 91 | ^{1}H NMR (500 MHz, 298K, $CDCl_3$) of *trans*-Bis(6-mercaptohexyl) cyclohexane-1,4-dicarboxylate **(29a)**

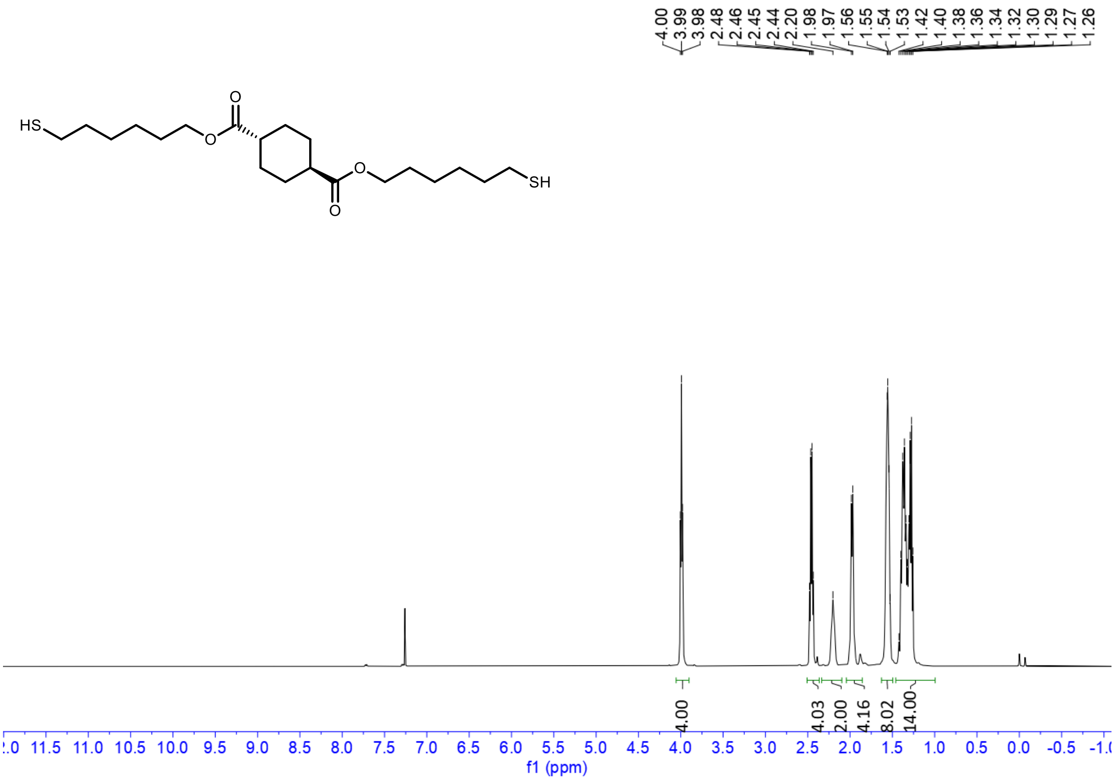

Supplementary Figure 92 | ^{13}C NMR (126 MHz, 298K, $CDCl_3$) of *trans*-Bis(6-mercaptohexyl) cyclohexane-1,4-dicarboxylate **(29a)**

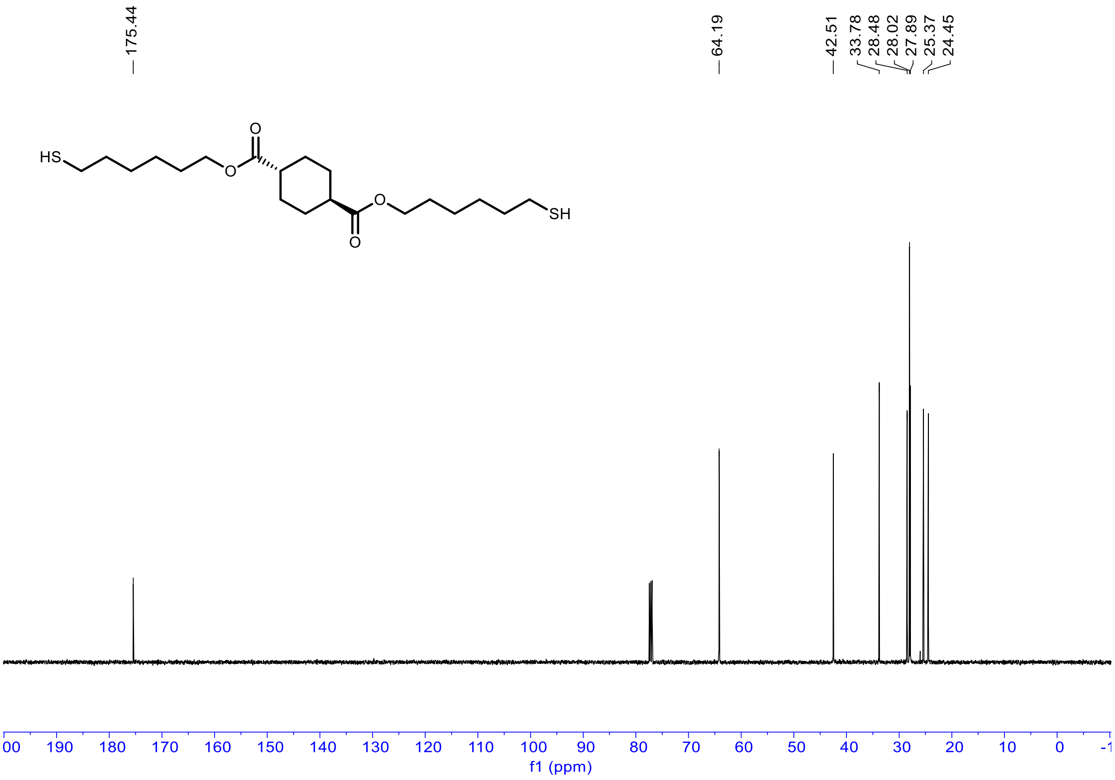

Supplementary Figure 93 | ^{1}H NMR (500 MHz, 298K, $CDCl_3$) of Bis(6-mercaptohexyl) bicyclo[2.2.2]octane-1,4-dicarboxylate **(30a)**

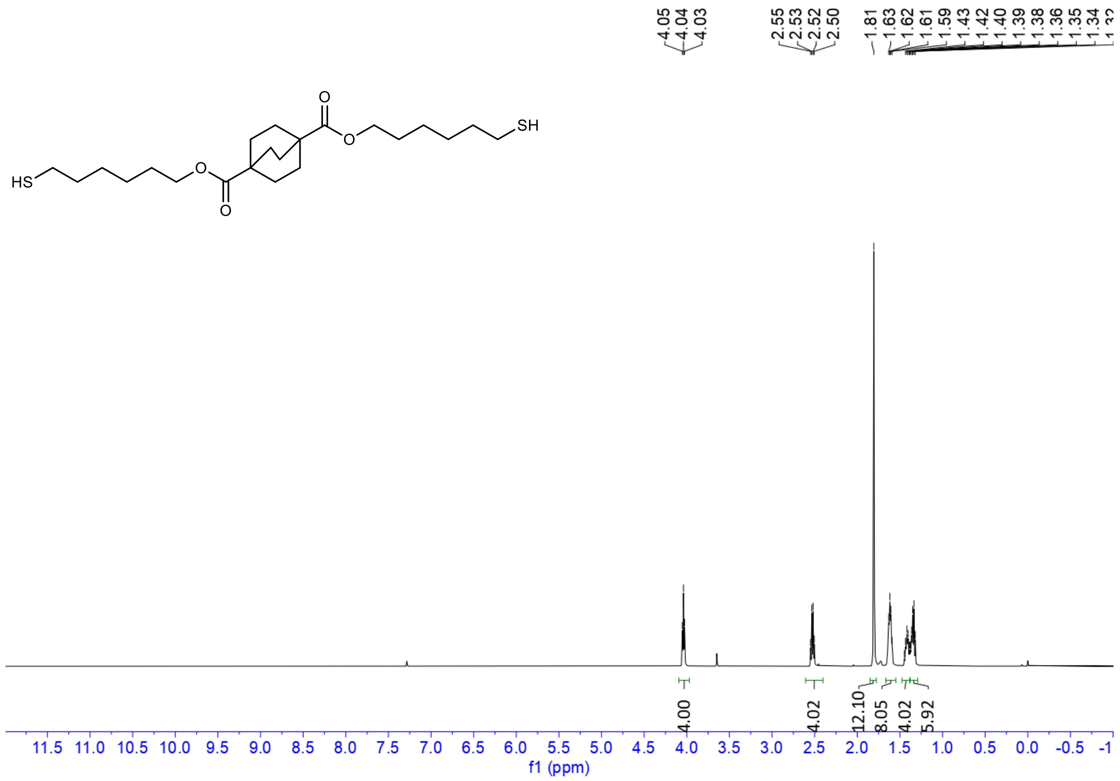

Supplementary Figure 94 | ^{13}C NMR (126 MHz, 298K, $CDCl_3$) of Bis(6-mercaptohexyl) bicyclo[2.2.2]octane-1,4-dicarboxylate **(30a)**

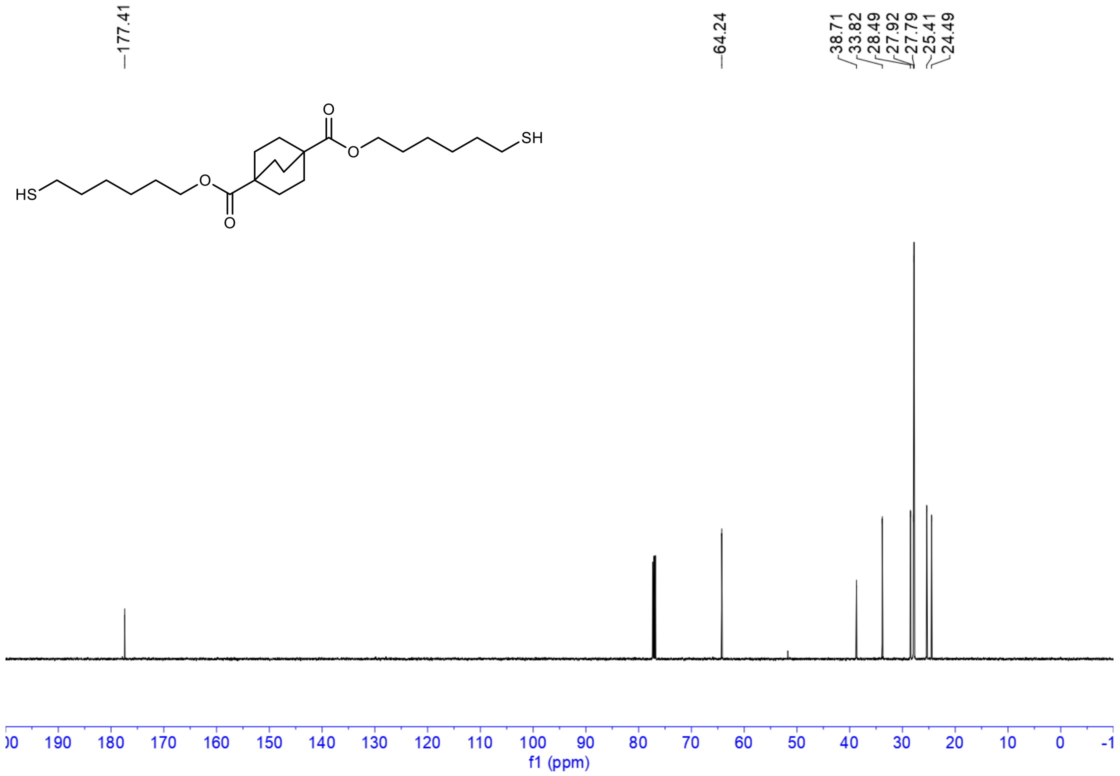

Supplementary Figure 95 | ^{1}H NMR (500 MHz, 298K, $CDCl_3$) of Bis(6-mercaptohexyl) adamantane-1,3-dicarboxylate **(31a)**

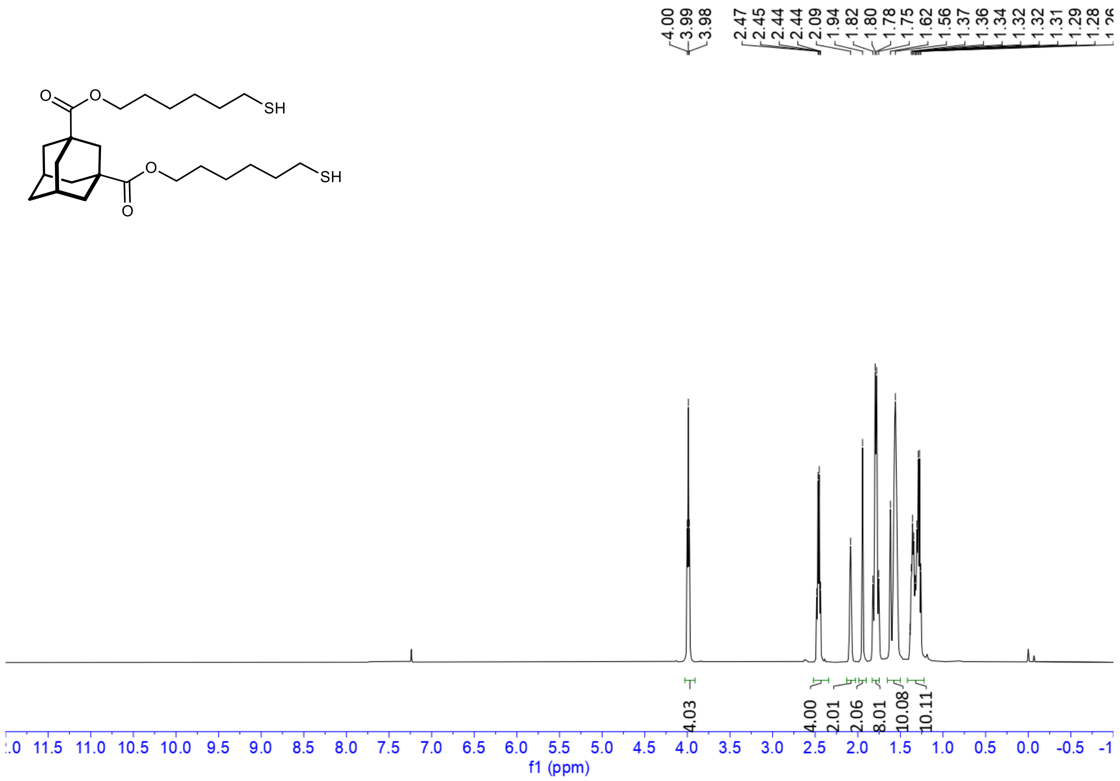

Supplementary Figure 96 | ^{13}C NMR (126 MHz, 298K, $CDCl_3$) of Bis(6-mercaptohexyl) adamantane-1,3-dicarboxylate **(31a)**

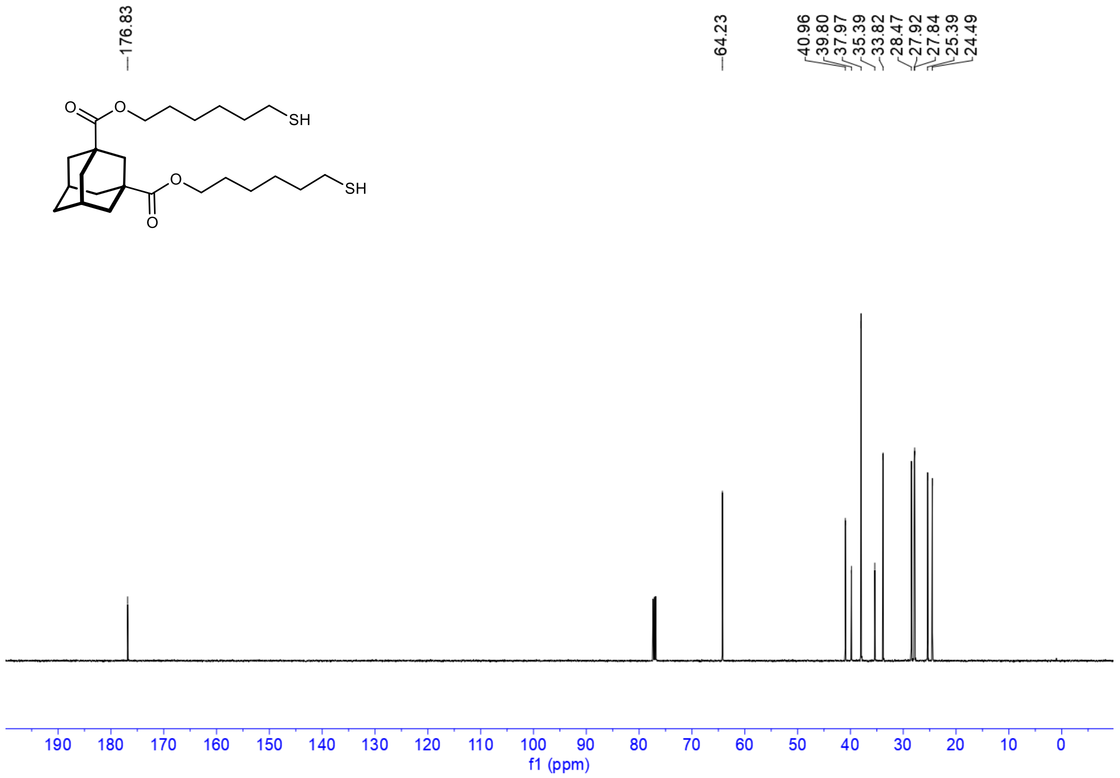

Supplementary Figure 97 | ^{1}H NMR (500 MHz, 298K, $CDCl_3$) of Oxybis (ethane-2,1-diyl) bis(3-mercaptopropanoate) **(23a)**

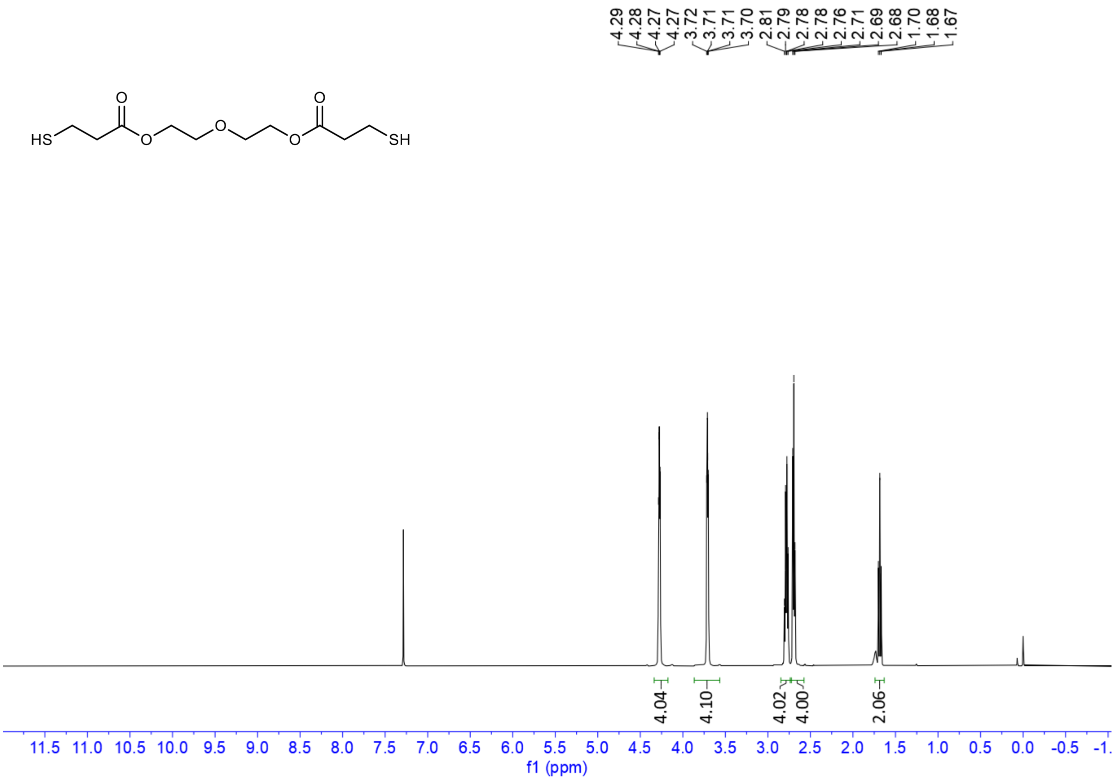

Supplementary Figure 98 | ^{13}C NMR (126 MHz, 298K, $CDCl_3$) of Oxybis (ethane-2,1-diyl) bis(3-mercaptopropanoate) **(23a)**

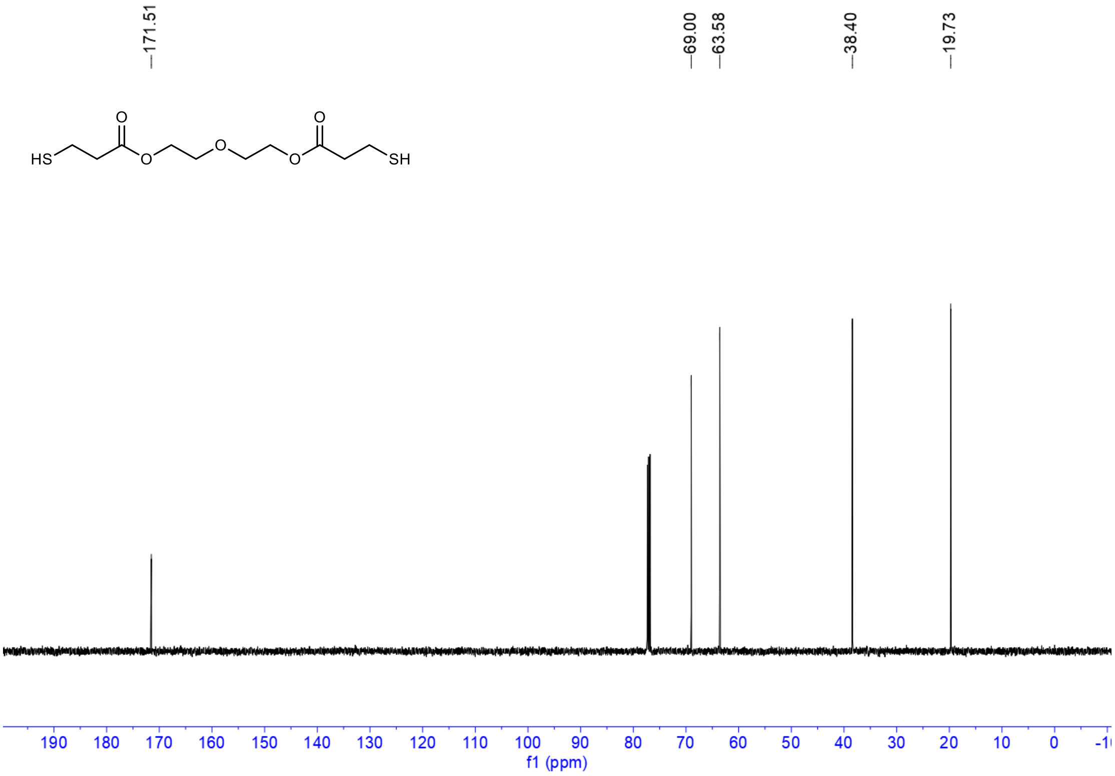

Supplementary Figure 99 | ^{1}H NMR (500 MHz, 298K, $CDCl_3$) of (Ethane-1,2-diylbis(oxy))bis(ethane-2,1-diyl) bis(3-mercaptopropanoate) **(24a)**

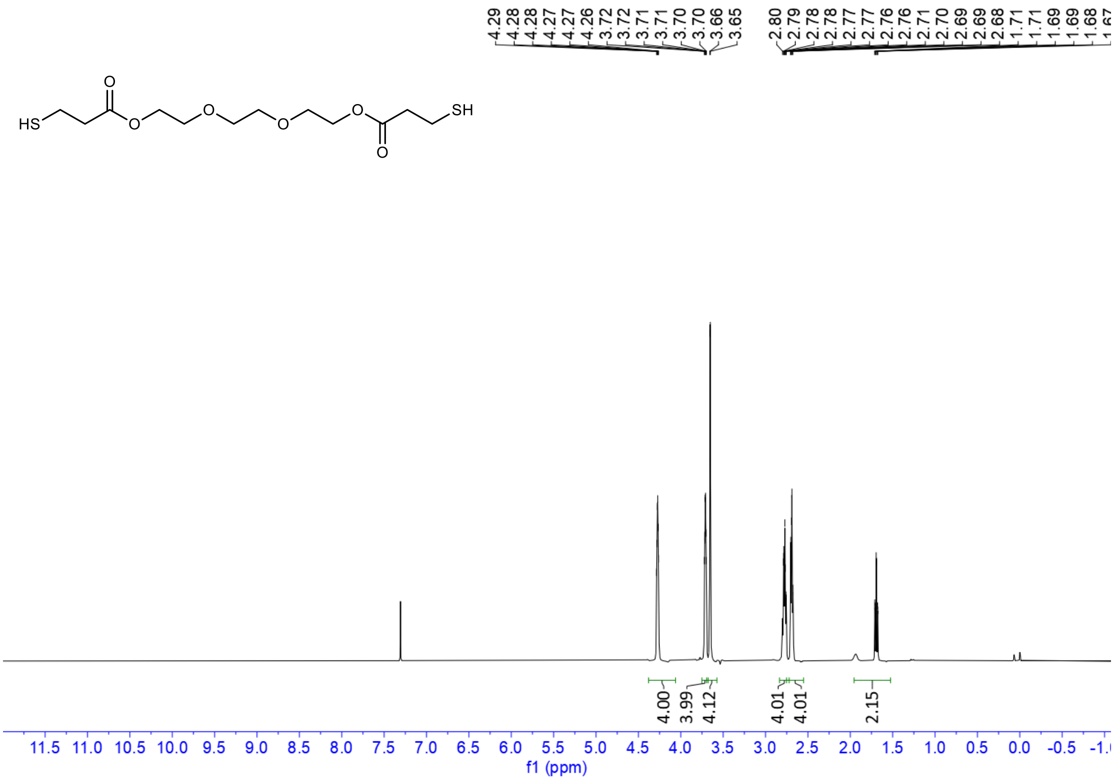

Supplementary Figure 100 | ^{13}C NMR (126 MHz, 298K, $CDCl_3$) of (Ethane-1,2-diylbis(oxy))bis(ethane-2,1-diyl) bis(3-mercaptopropanoate) **(24a)**

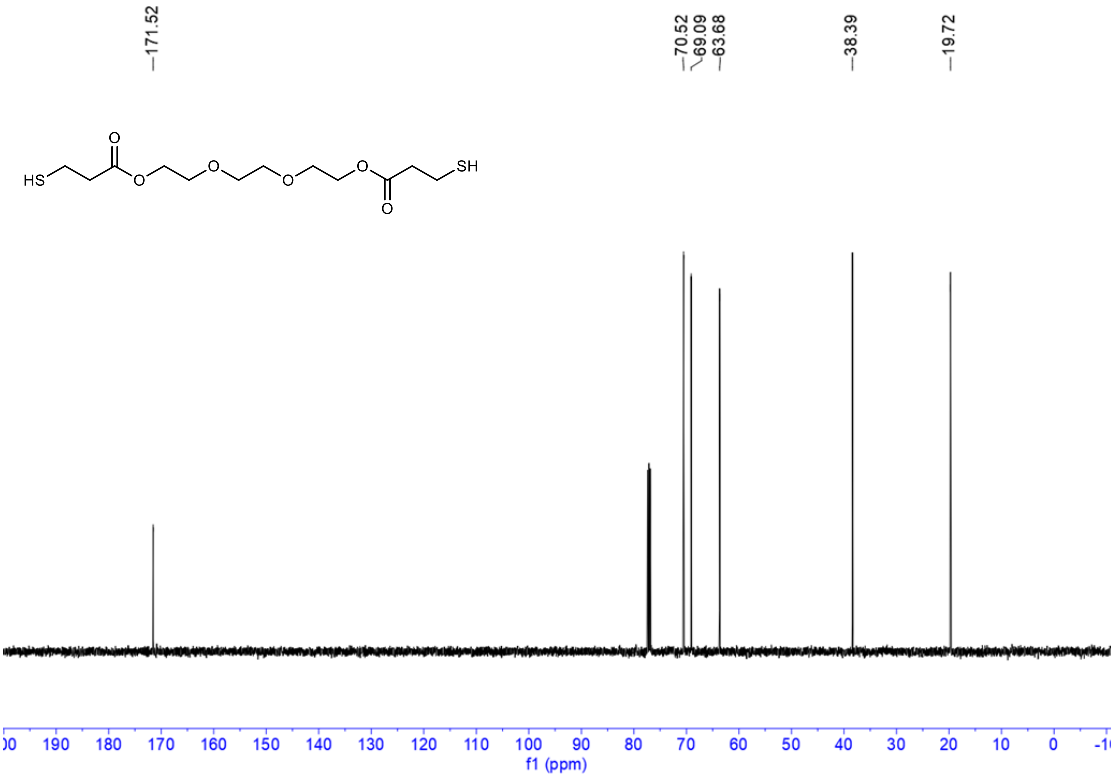

Supplementary Figure 101 | ^{1}H NMR (500 MHz, 298K, $CDCl_3$) of ((Oxybis(ethane-2,1-diyl))bis(oxy))bis(ethane-2,1-diyl) bis(3-mercaptopropanoate) **(25a)**

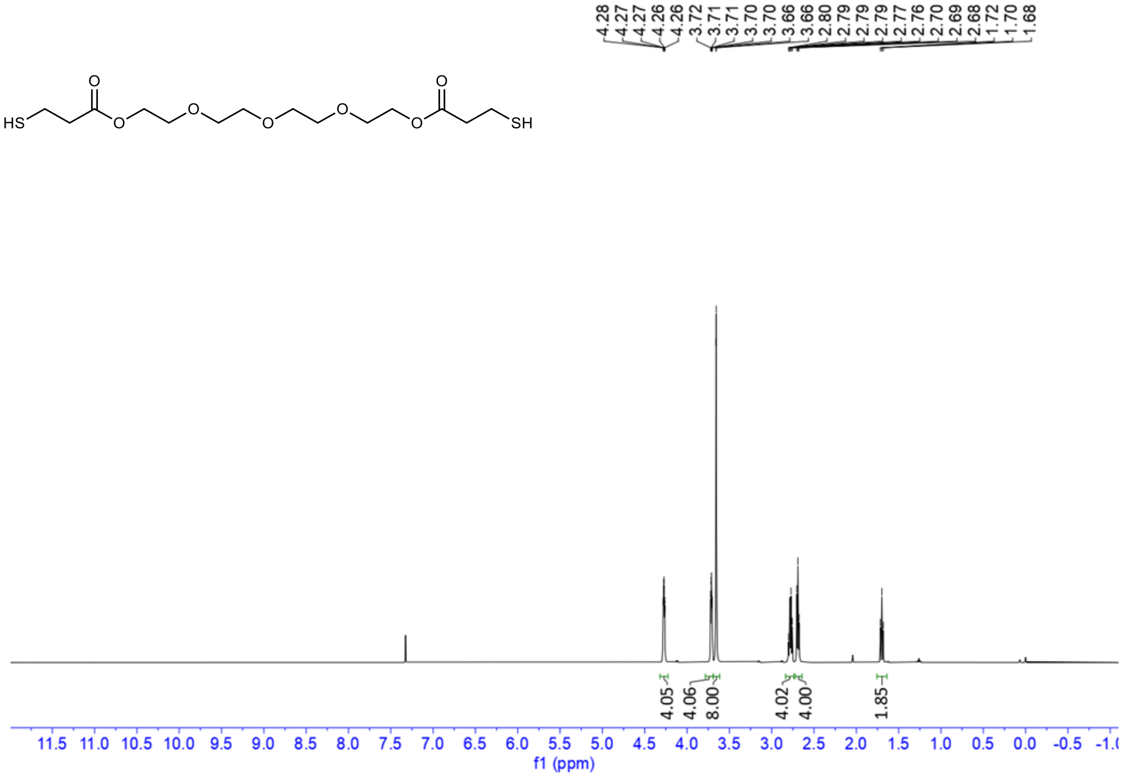

Supplementary Figure 102 | ^{13}C NMR (126 MHz, 298K, $CDCl_3$) of ((Oxybis(ethane-2,1-diyl))bis(oxy))bis(ethane-2,1-diyl) bis(3-mercaptopropanoate) **(25a)**

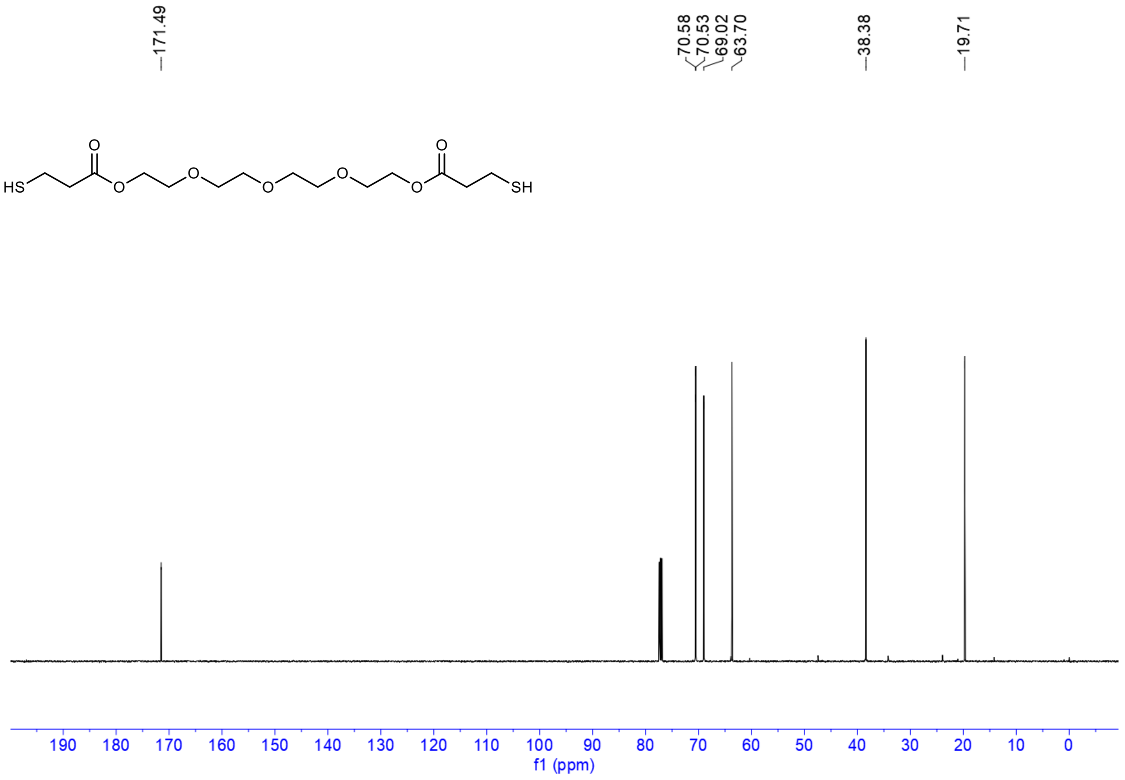

Supplementary Figure 103 | ^{1}H NMR (500 MHz, 298K, $CDCl_3$) of 3,6,9,12,15,18,21-Heptaoxatricosan-1,23-diyl bis(3 mercaptopropanoate) **(26a)**

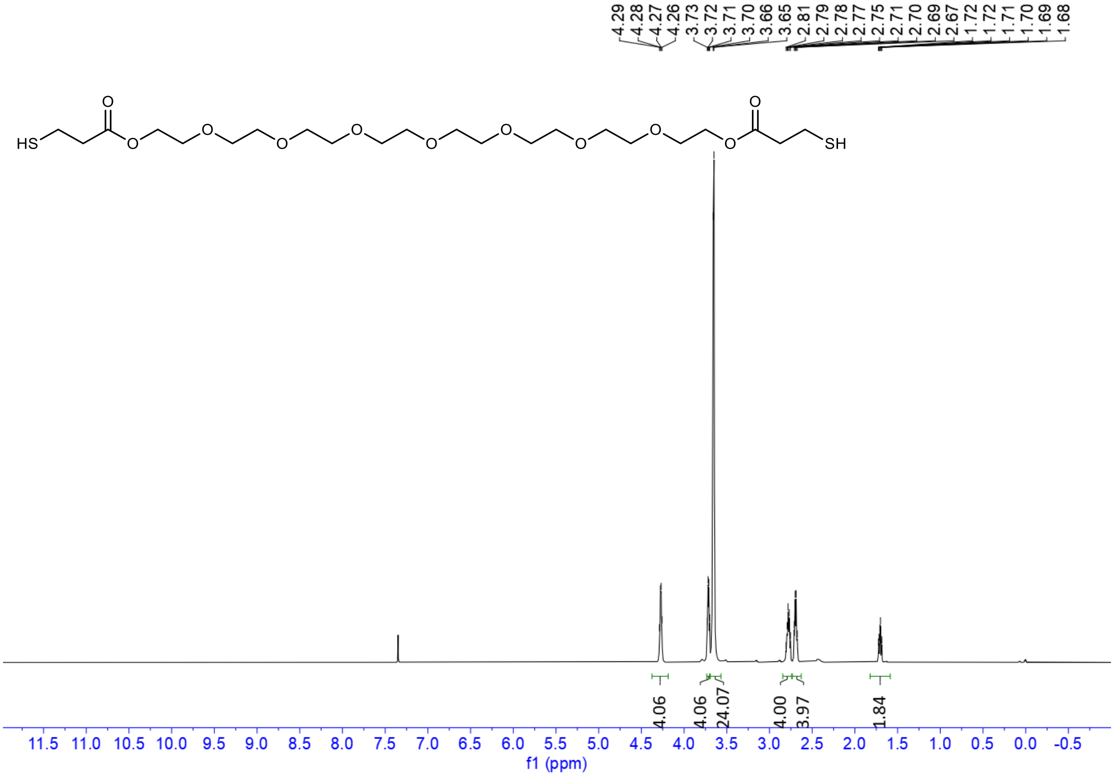

Supplementary Figure 104 | ^{13}C NMR (126 MHz, 298K, $CDCl_3$) of 3,6,9,12,15,18,21-Heptaoxatricosan-1,23-diyl bis(3 mercaptopropanoate) **(26a)**

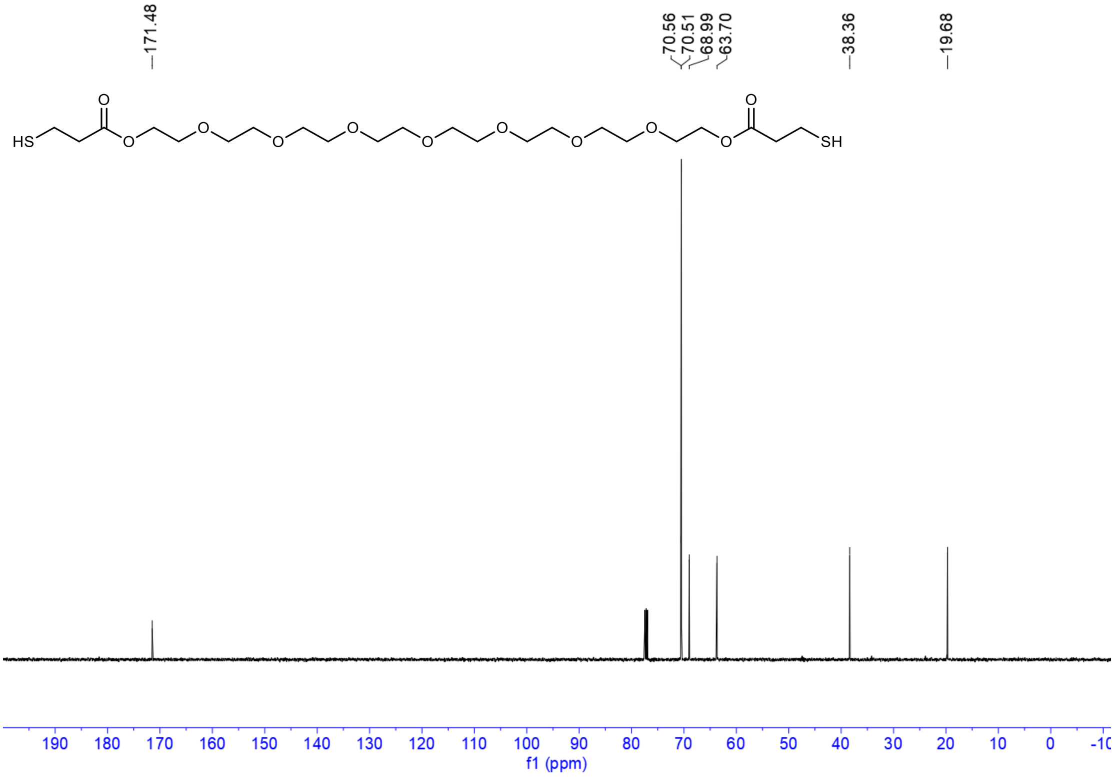

Supplementary Figure 105 | ^{1}H NMR (500 MHz, 298K, $CDCl_3$) of (1,4-Phenylenebis(oxy))bis (ethane-2,1-diyl) bis(2-mercaptobenzoate) **(52a)**

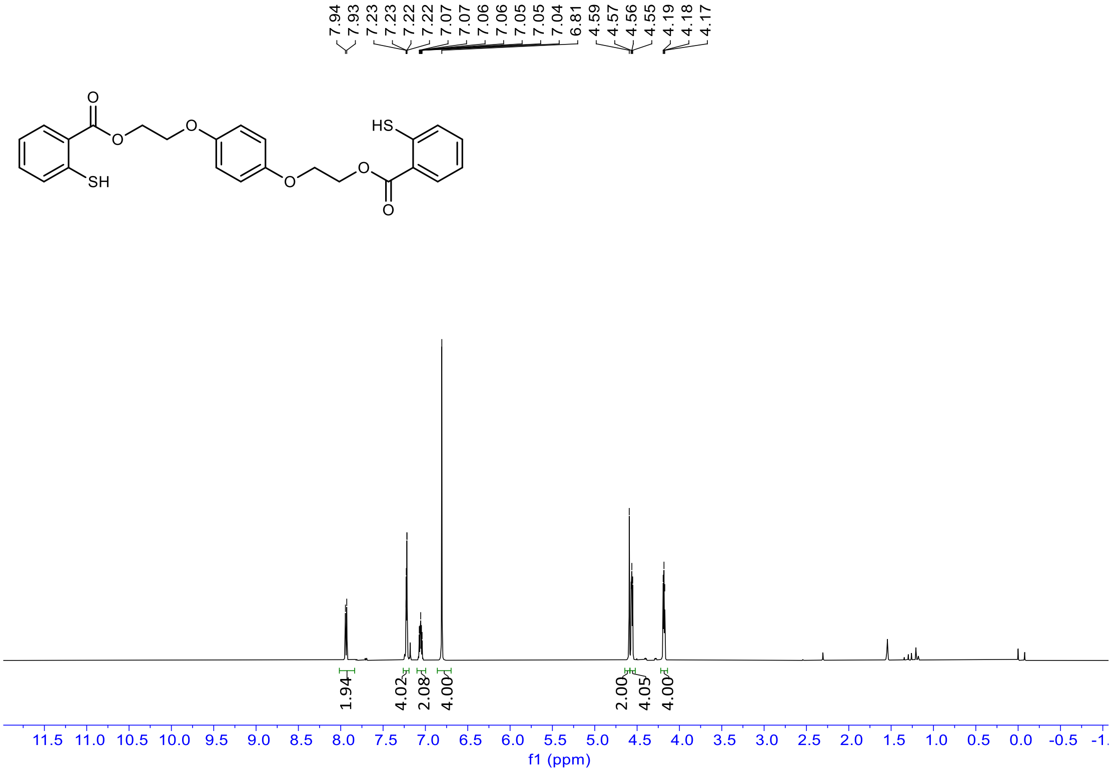

Supplementary Figure 106 | ^{13}C NMR (126 MHz, 298K, $CDCl_3$) of (1,4-Phenylenebis(oxy)) bis(ethane-2,1-diyl) bis(2-mercaptobenzoate) **(52a)**

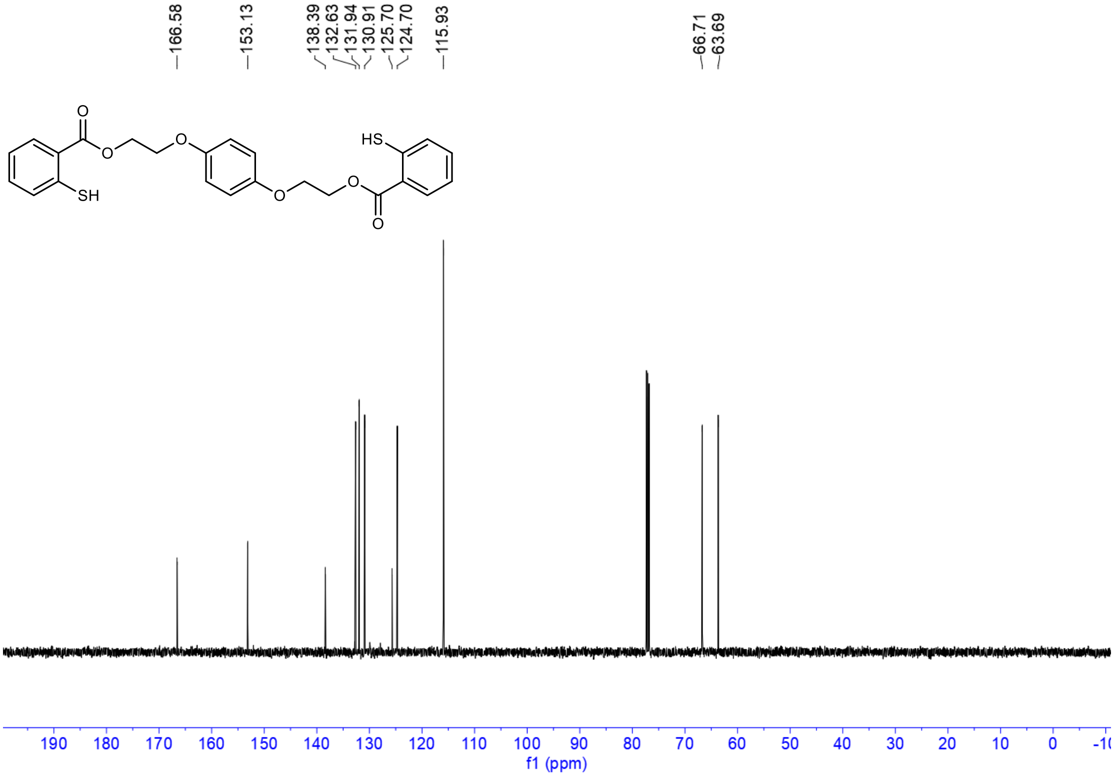

Supplementary Figure 107 | ^{1}H NMR (500 MHz, 298K, $CDCl_3$) of 6-Mercaptohexyl 2-mercaptbenzoate **(33a)**

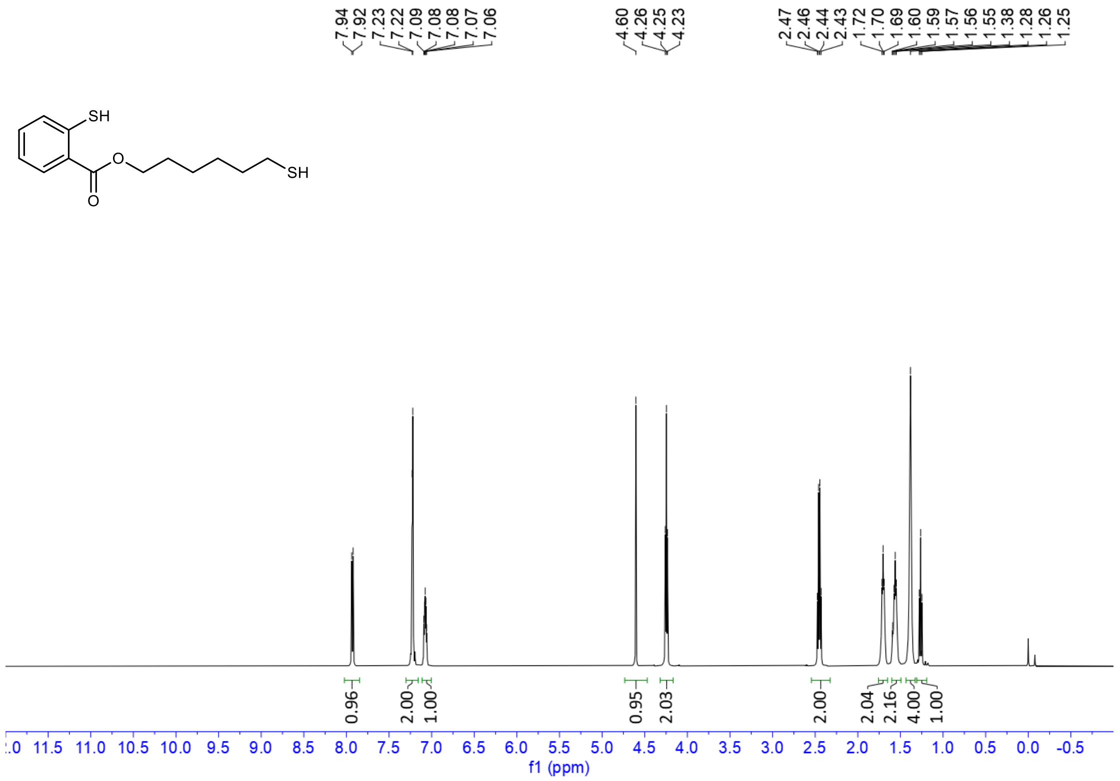

Supplementary Figure 108 | ^{13}C NMR (126 MHz, 298K, $CDCl_3$) of 6-Mercaptohexyl 2-mercaptbenzoate **(33a)**

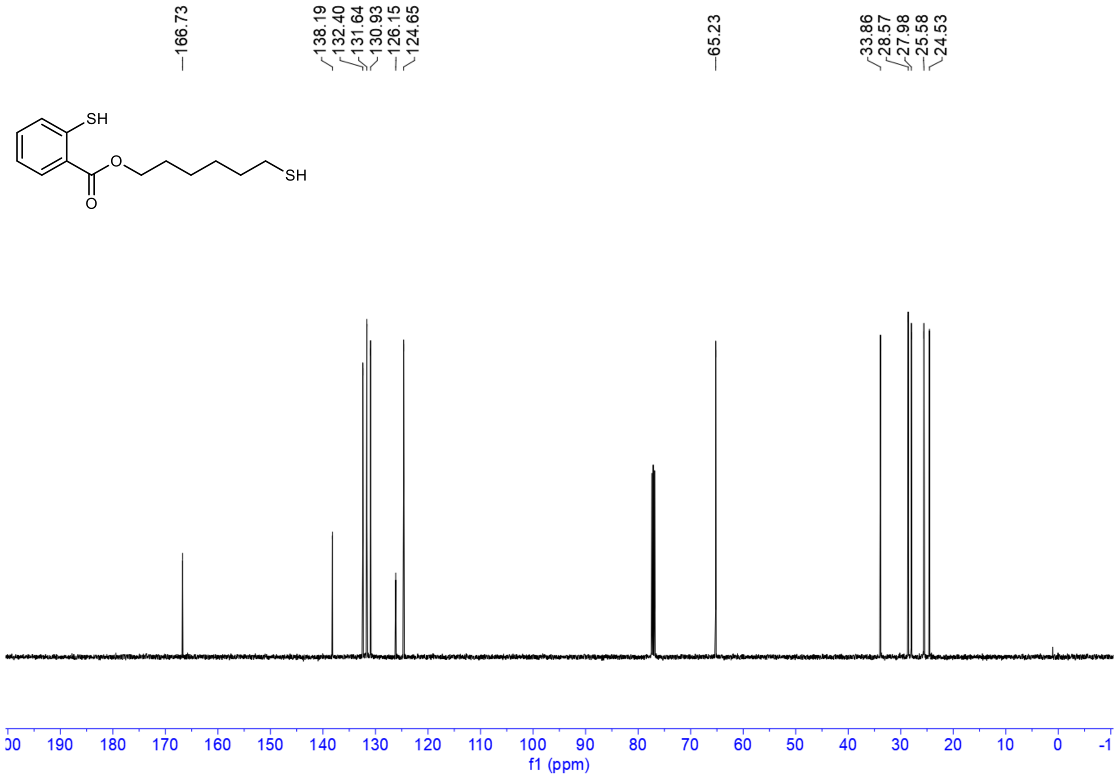

Supplementary Figure 109 | ^{1}H NMR (500 MHz, 298K, $CDCl_3$) of 1,2-Phenylenebis(methylene) bis(3-mercaptopropanoate) **(34a)**

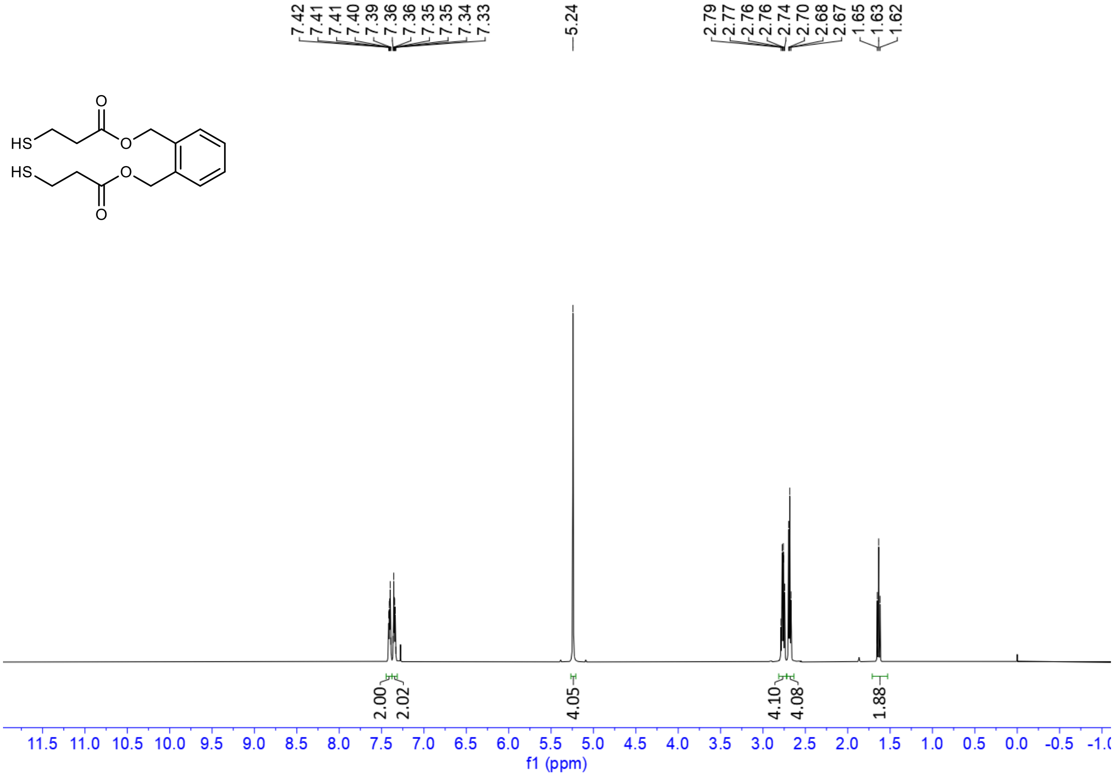

Supplementary Figure 110 | ^{13}C NMR (126 MHz, 298K, $CDCl_3$) of 1,2-Phenylenebis(methyl ene) bis(3-mercaptopropanoate) **(34a)**

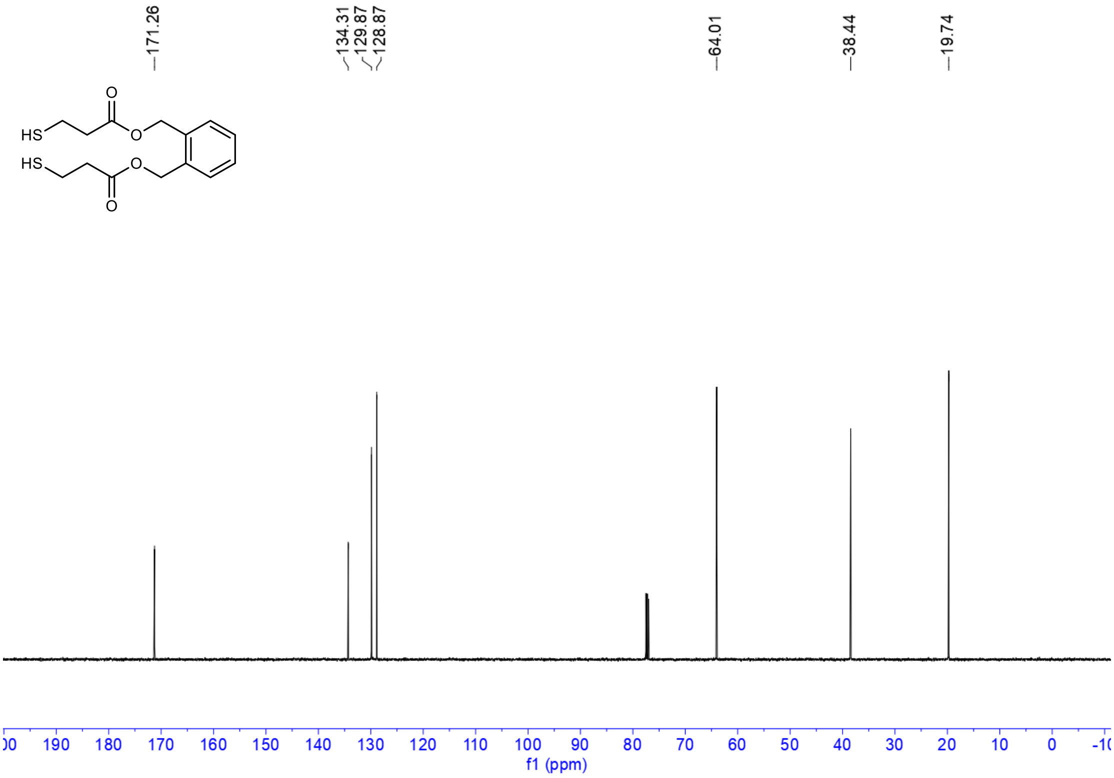

Supplementary Figure 111 | ^{1}H NMR (500 MHz, 298K, $CDCl_3$) of 1,3-Phenylenebis(methylene) bis(3-mercaptopropanoate) **(36a)**

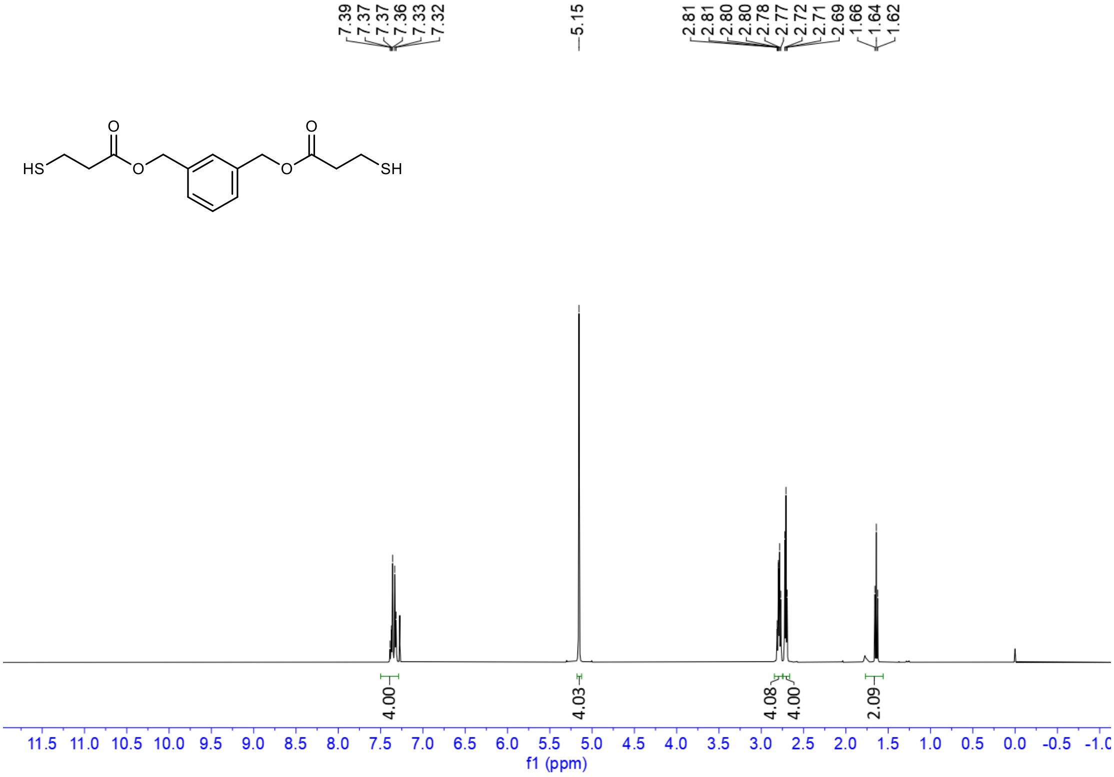

Supplementary Figure 112 | ^{13}C NMR (126 MHz, 298K, $CDCl_3$) of 1,3-Phenylenebis (methylene) bis(3-mercaptopropanoate) **(36a)**

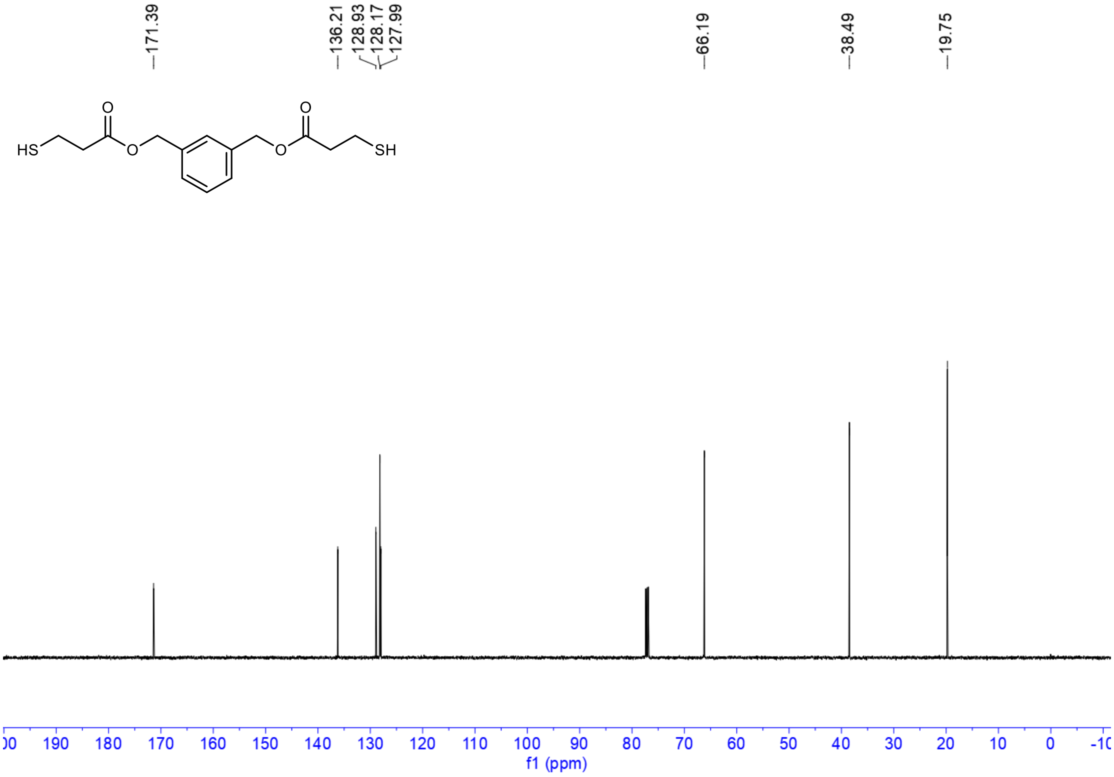

Supplementary Figure 113 | ^{1}H NMR (500 MHz, 298K, $CDCl_3$) of 1,4-Phenylenebis(methylene) bis(3-mercaptopropanoate) **(37a)**

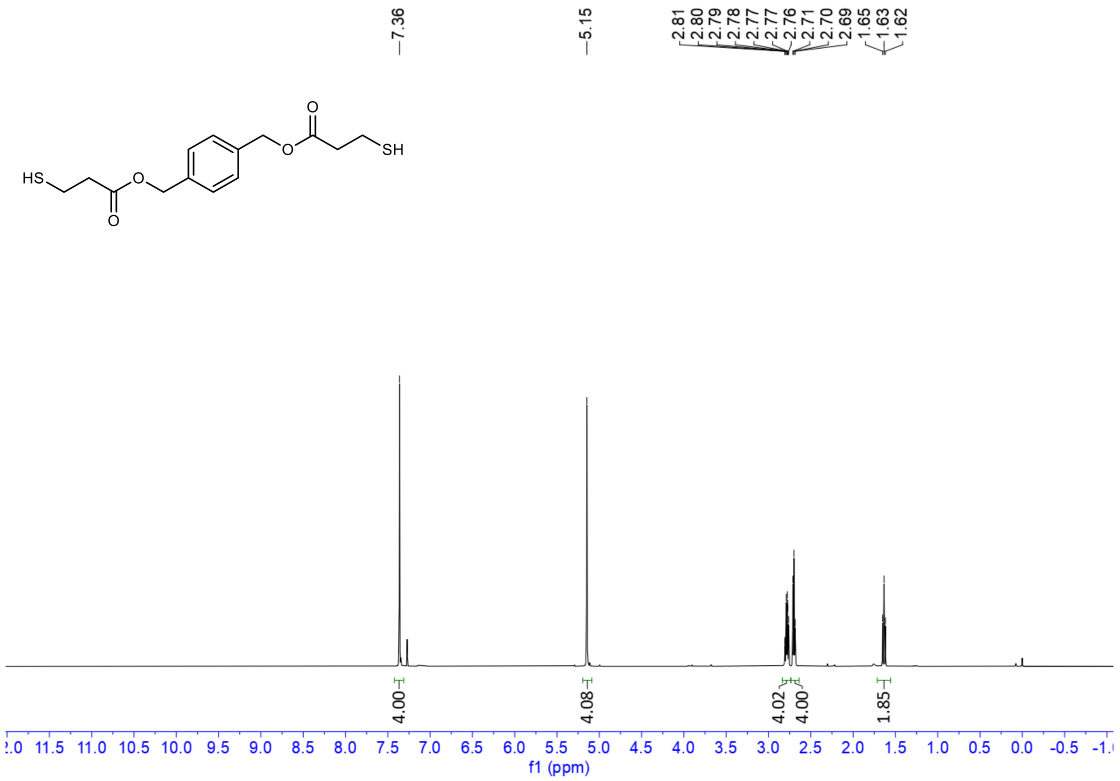

Supplementary Figure 114 | ^{13}C NMR (126 MHz, 298K, $CDCl_3$) of 1,4-Phenylenebis (methylene) bis(3-mercaptopropanoate) **(37a)**

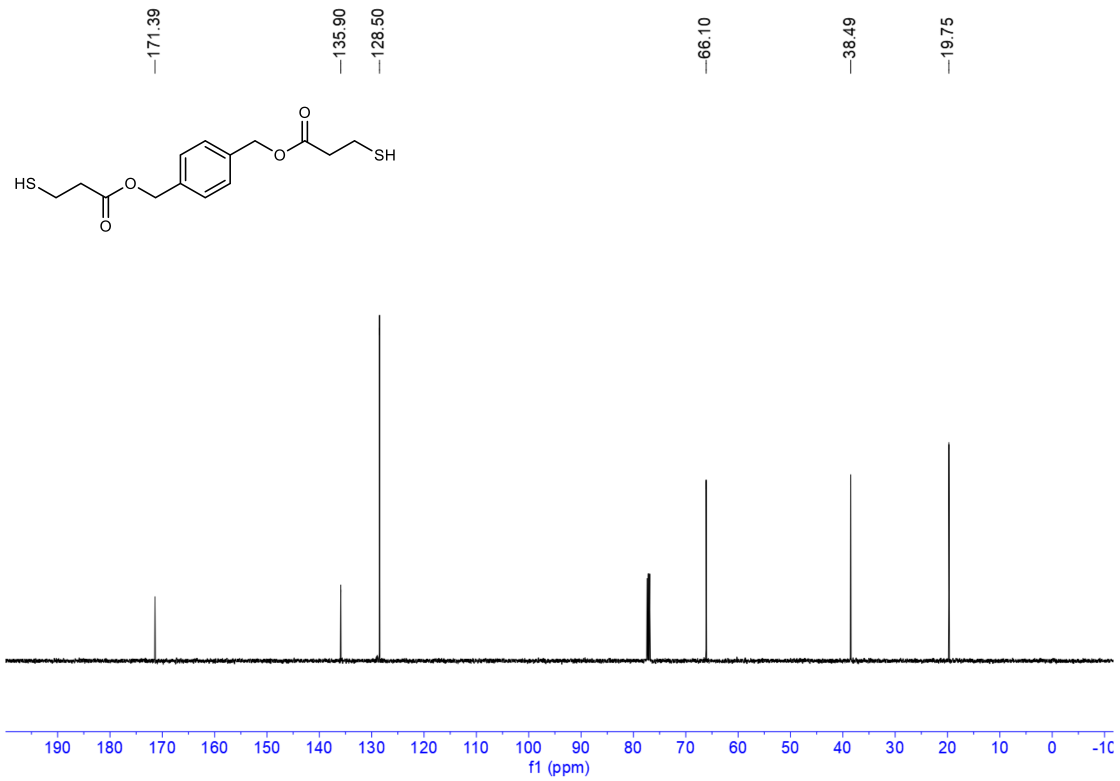

Supplementary Figure 115 | ^{1}H NMR (500 MHz, 298K, $CDCl_3$) of 1,1'-Ferrocenedicarboxylic acid bis(3-mercaptopropanoate) **(42a)**

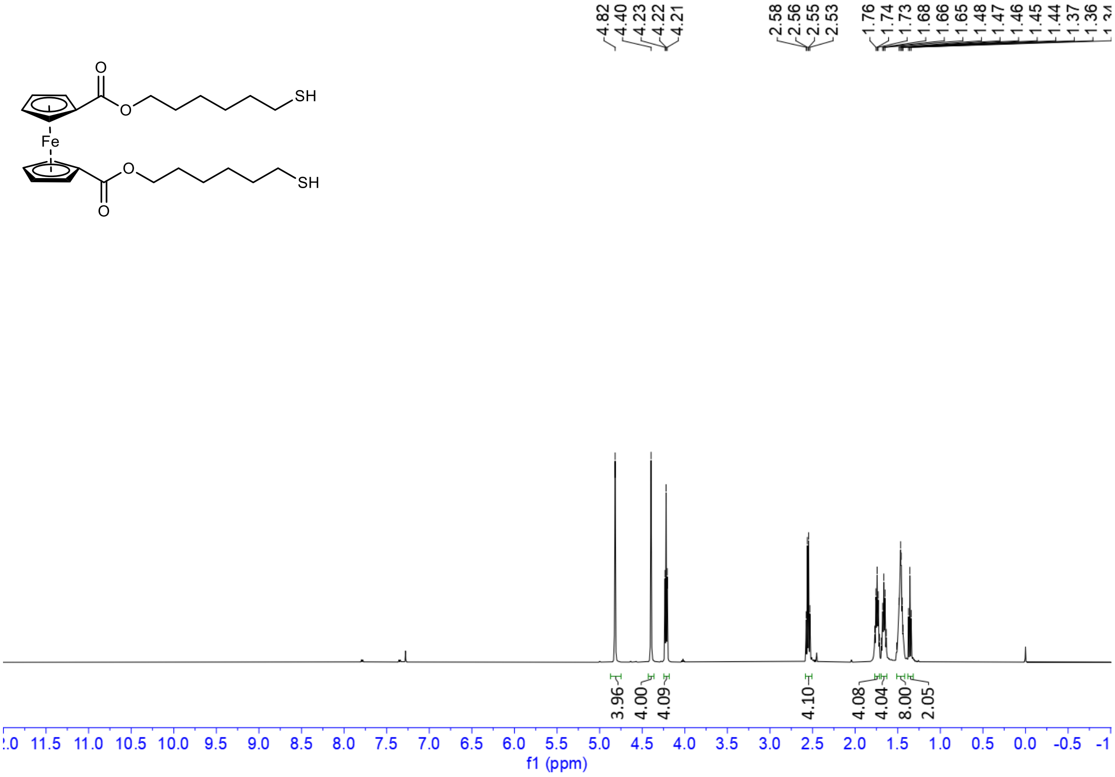

Supplementary Figure 116 | ^{13}C NMR (126 MHz, 298K, $CDCl_3$) of 1,1'-Ferrocenedicarboxylic acid bis(3-mercaptopropanoate) **(42a)**

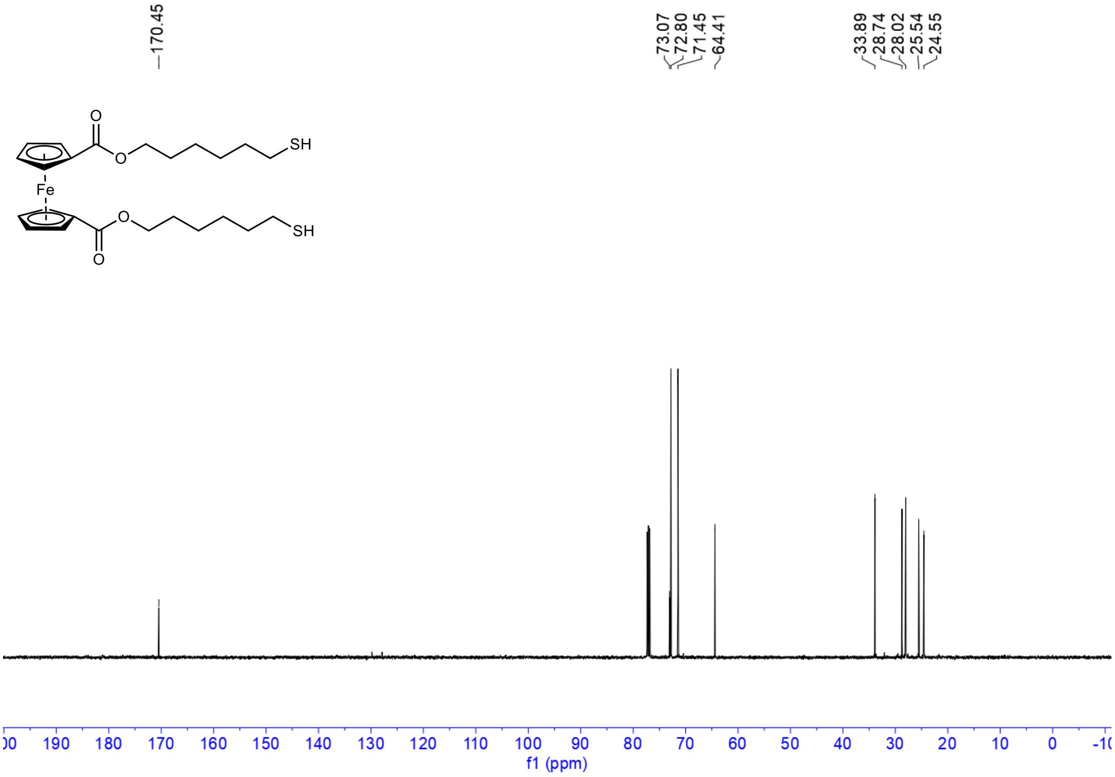

Supplementary Figure 117 | ^{1}H NMR (500 MHz, 298K, $CDCl_3$) of Pyridine-2,6-diylbis(methylene) bis(3-mercaptopropanoate) **(40a)**

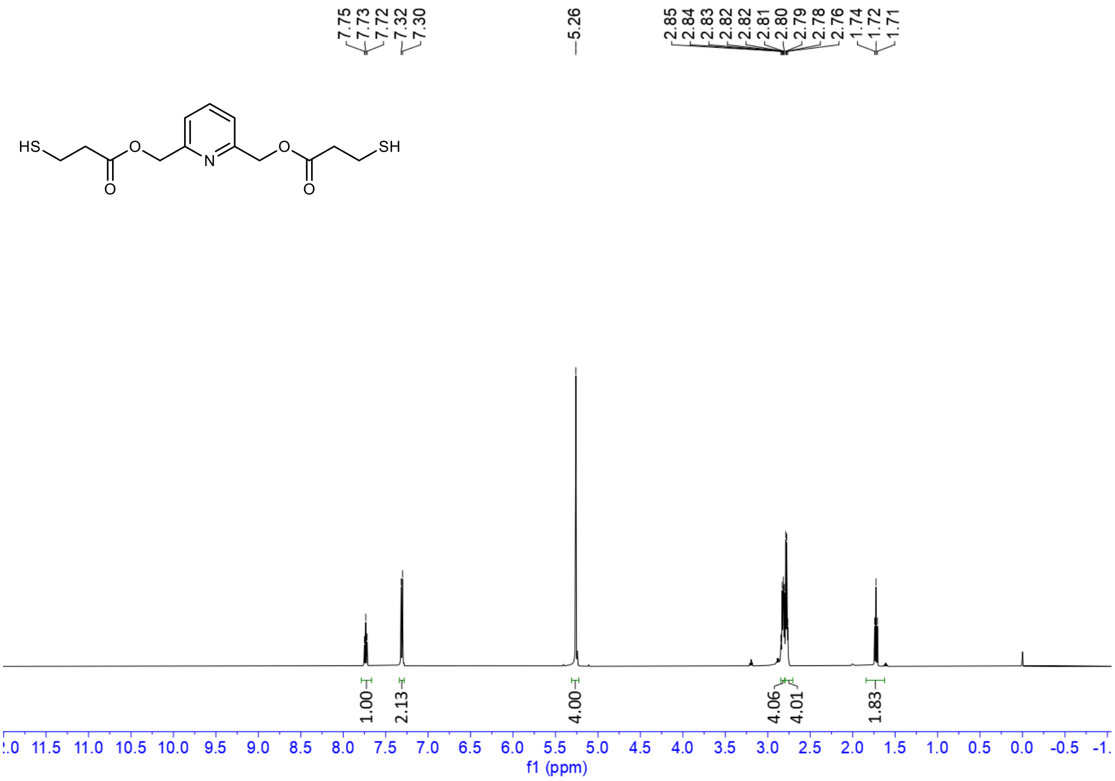

Supplementary Figure 118 | ^{13}C NMR (126 MHz, 298K, $CDCl_3$) of Pyridine-2,6-diylbis(methylene) bis(3-mercaptopropanoate) **(40a)**

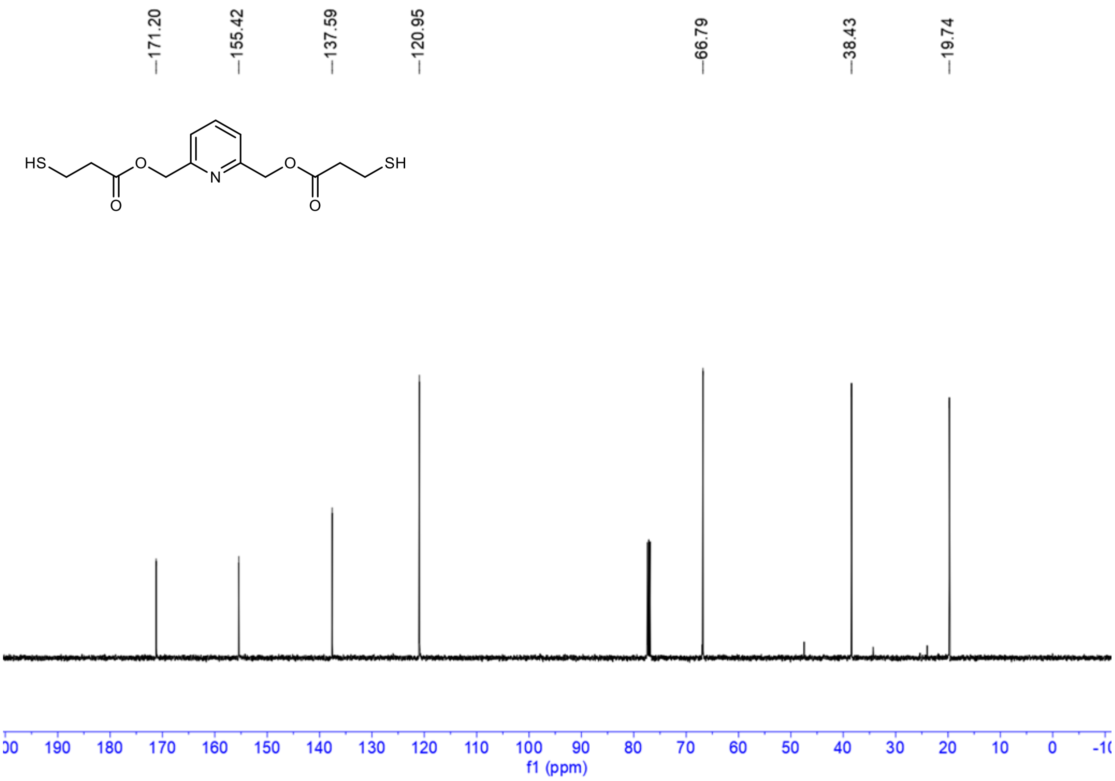

Supplementary Figure 119 | ^{1}H NMR (500 MHz, 298K, $CDCl_3$) of (Phenylazanediyl)bis(ethane-2,1-diyl) bis(3-mercaptopropanoate) **(27a)**

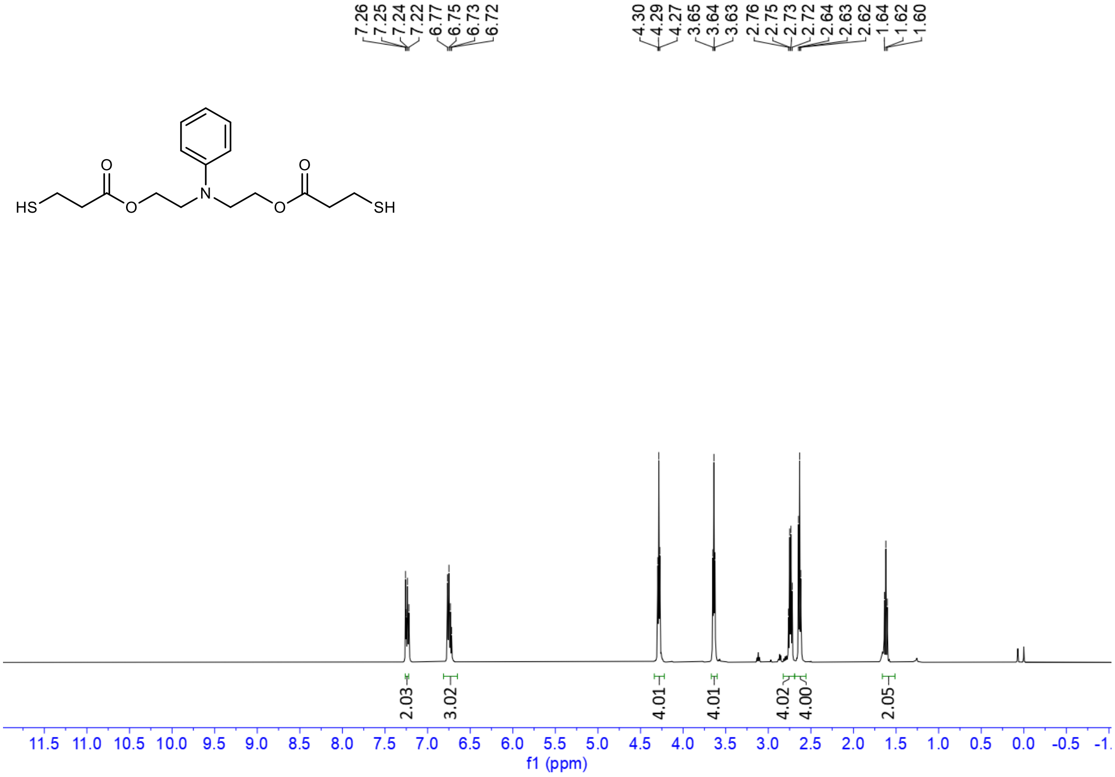

Supplementary Figure 120 | ^{13}C NMR (126 MHz, 298K, $CDCl_3$) of (Phenylazanediyl)bis(ethane-2,1-diyl) bis(3-mercaptopropanoate) **(27a)**

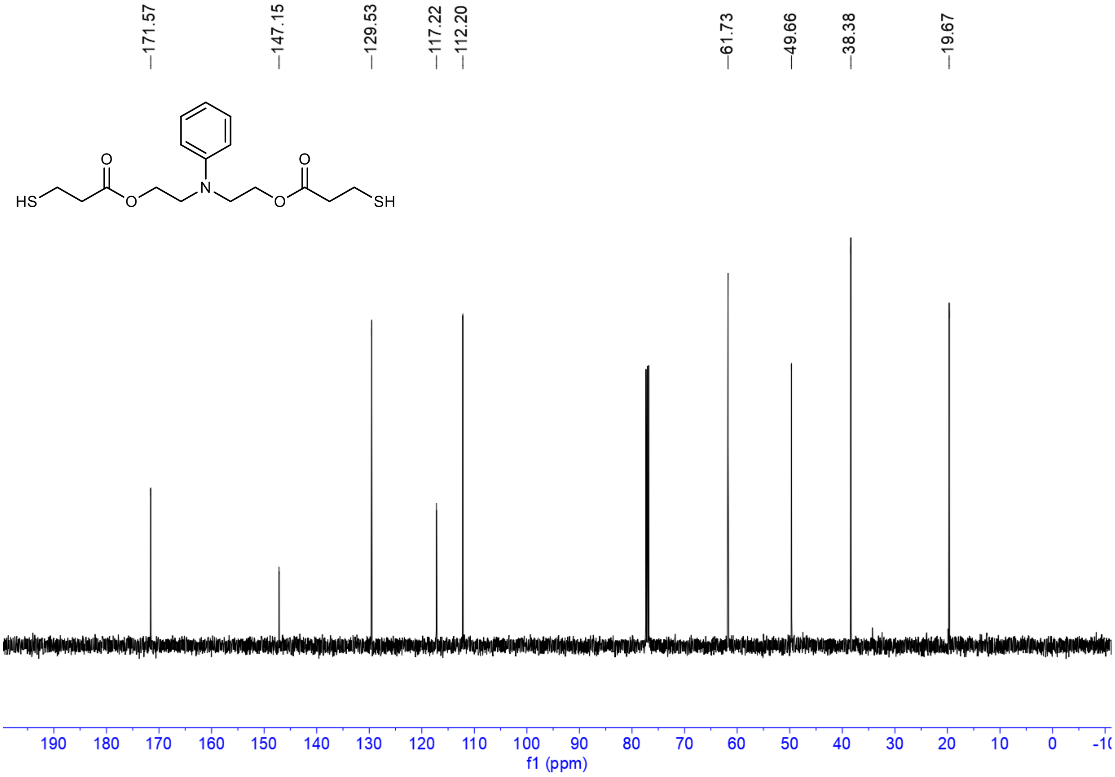

Supplementary Figure 121 | ^{1}H NMR (500 MHz, 298K, $CDCl_3$) of (Phenylazanediyl)bis(ethane-2,1-diyl) bis(3-mercaptopropanoate) **(28a)**

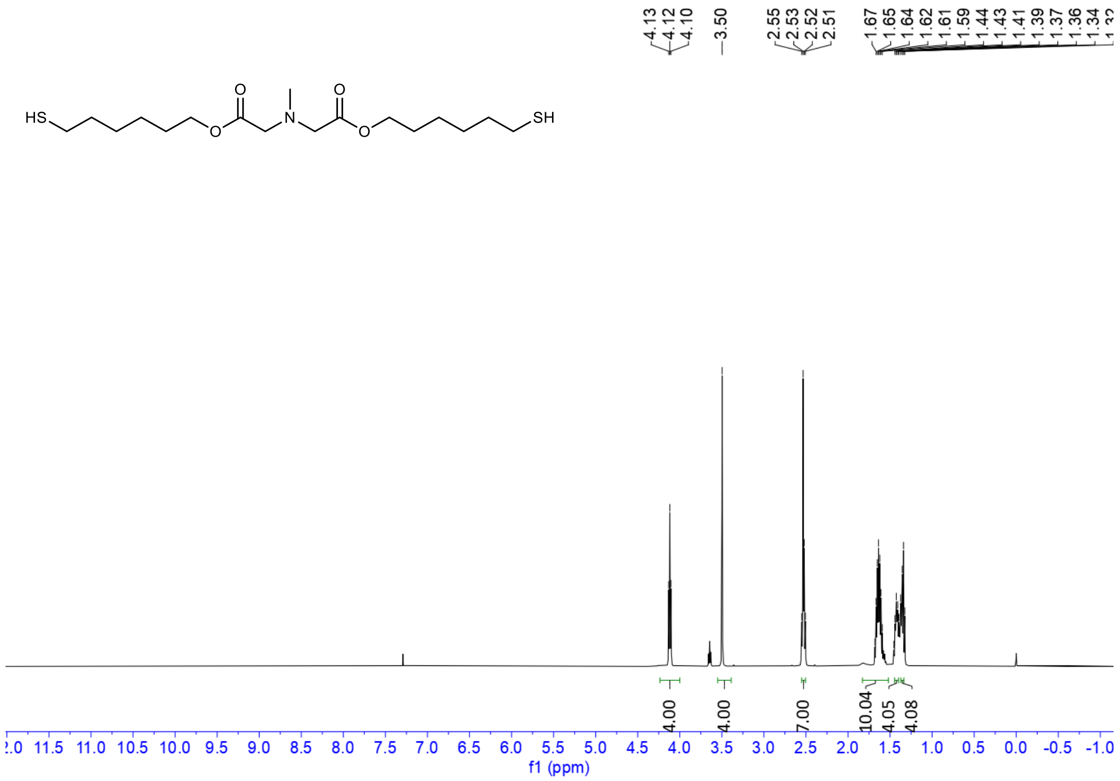

Supplementary Figure 122 | ^{13}C NMR (126 MHz, 298K, $CDCl_3$) of (Phenylazanediyl)bis (ethane-2,1-diyl) bis(3-mercaptopropanoate) **(28a)**

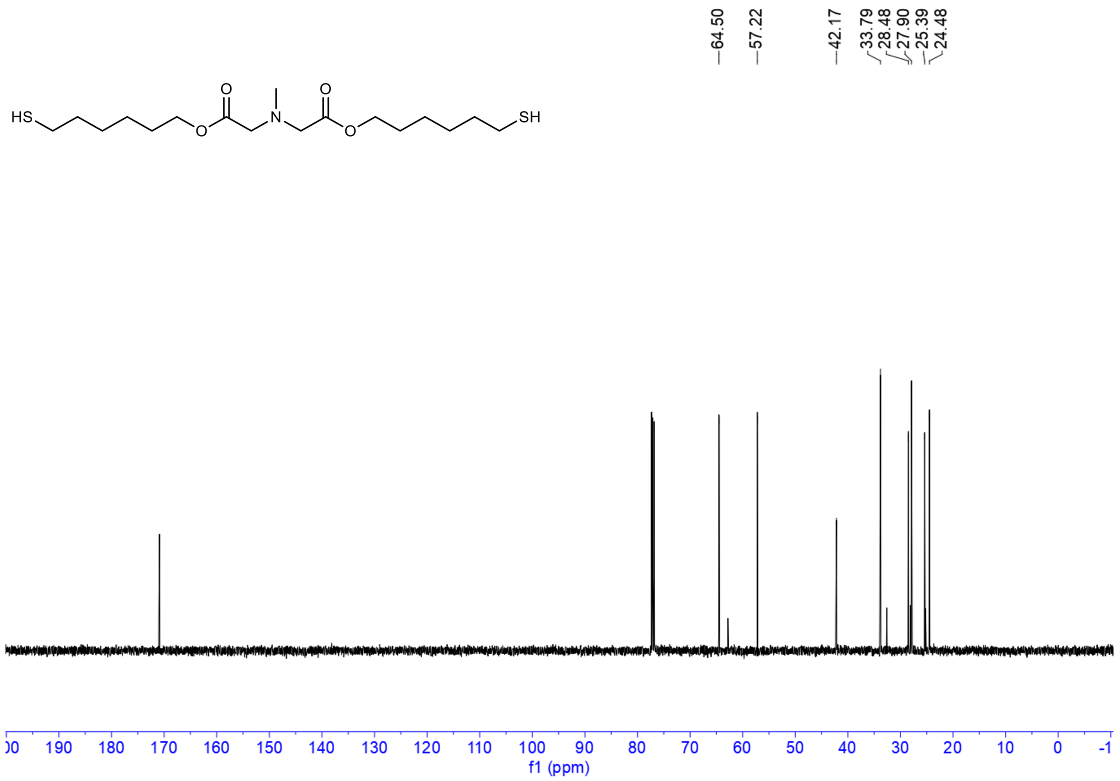

Supplementary Figure 123 | ^{1}H NMR (500 MHz, 298K, $CDCl_3$) of Piperazine-1,4-diylbis(ethane-2,1-diyl) bis(3-mercaptopropanoate) **(32a)**

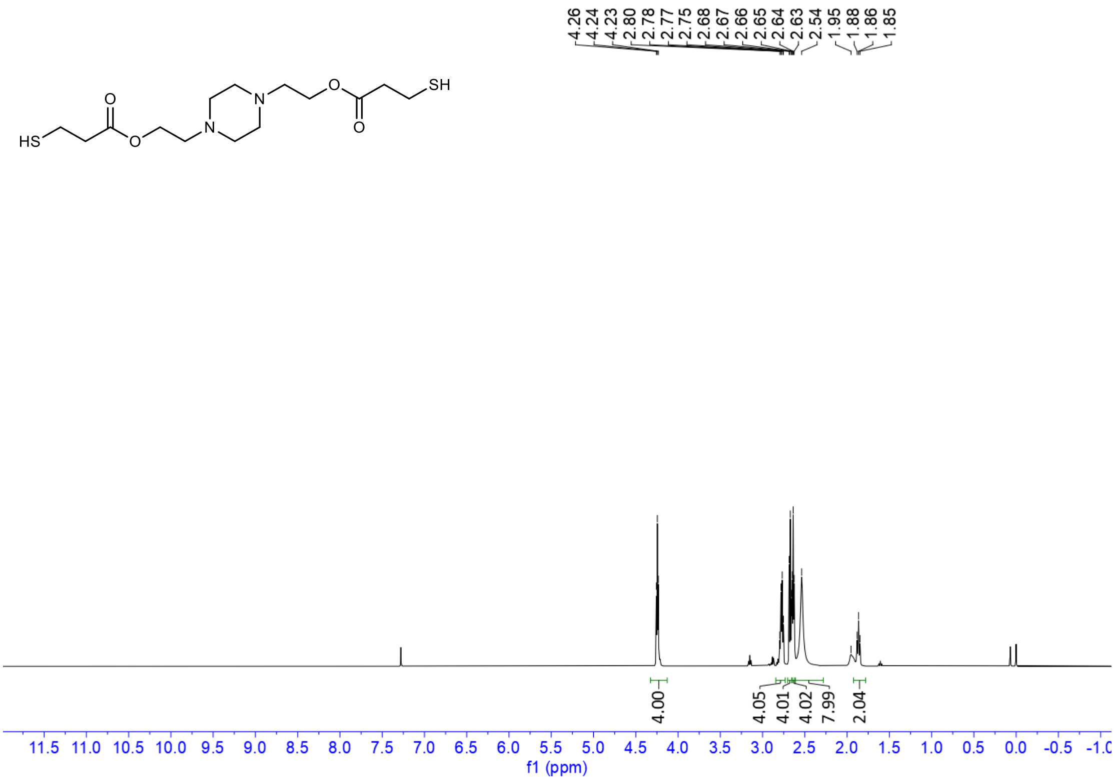

Supplementary Figure 124 | ^{13}C NMR (126 MHz, 298K, $CDCl_3$) of Piperazine-1,4-diylbis(ethane-2,1-diyl) bis(3-mercaptopropanoate) **(32a)**

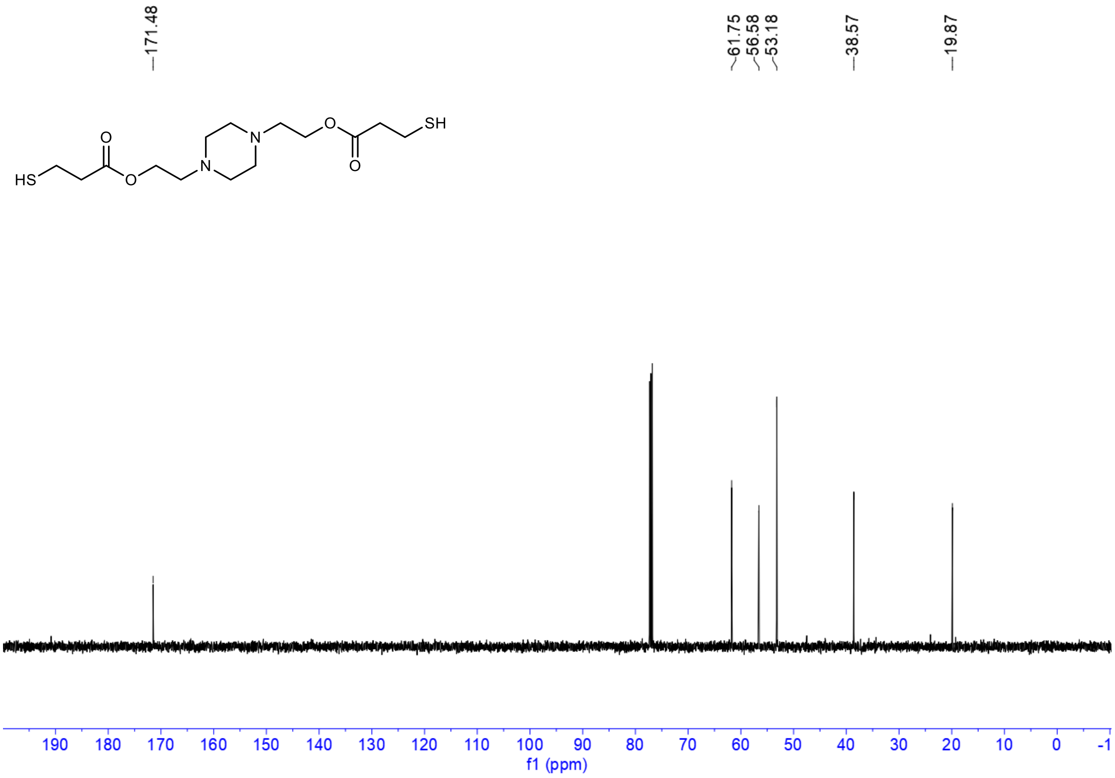

Supplementary Figure 125 | ^{1}H NMR (500 MHz, 298K, $CDCl_3$) of Furan-2,5-diylbis(methylene) bis(3-mercaptopropanoate) **(41a)**

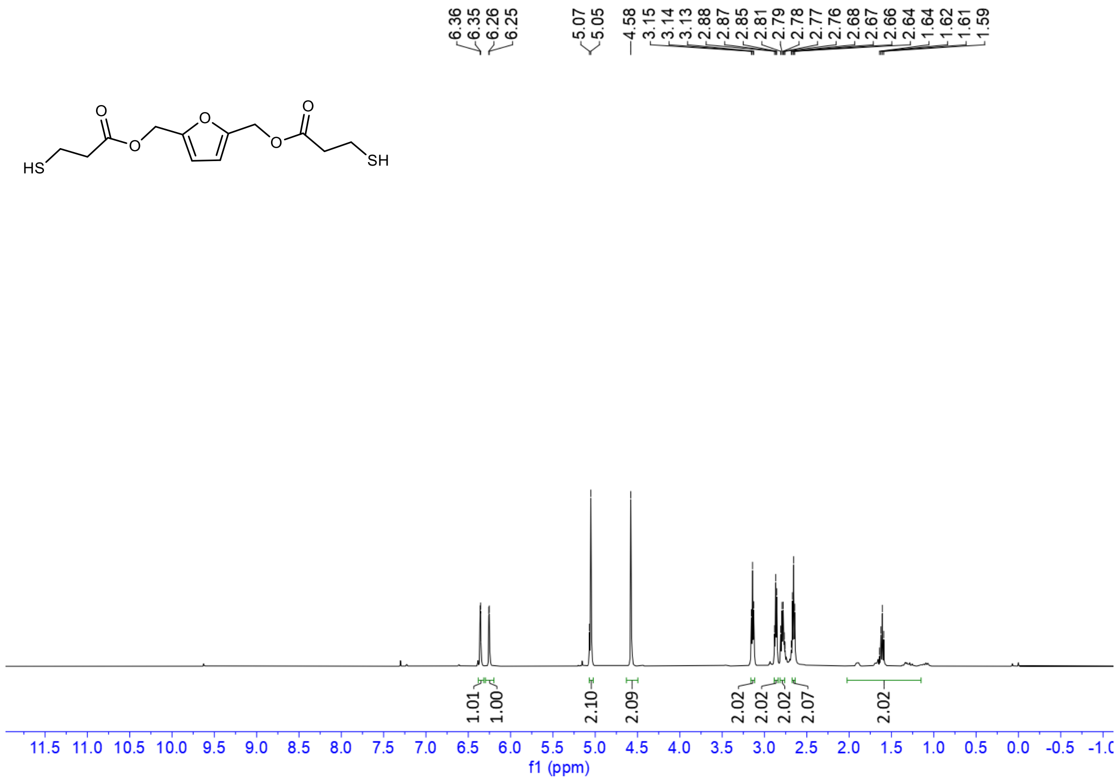

Supplementary Figure 126 | ^{13}C NMR (126 MHz, 298K, $CDCl_3$) of Furan-2,5-diylbis(methy lene) bis(3-mercaptopropanoate) **(41a)**

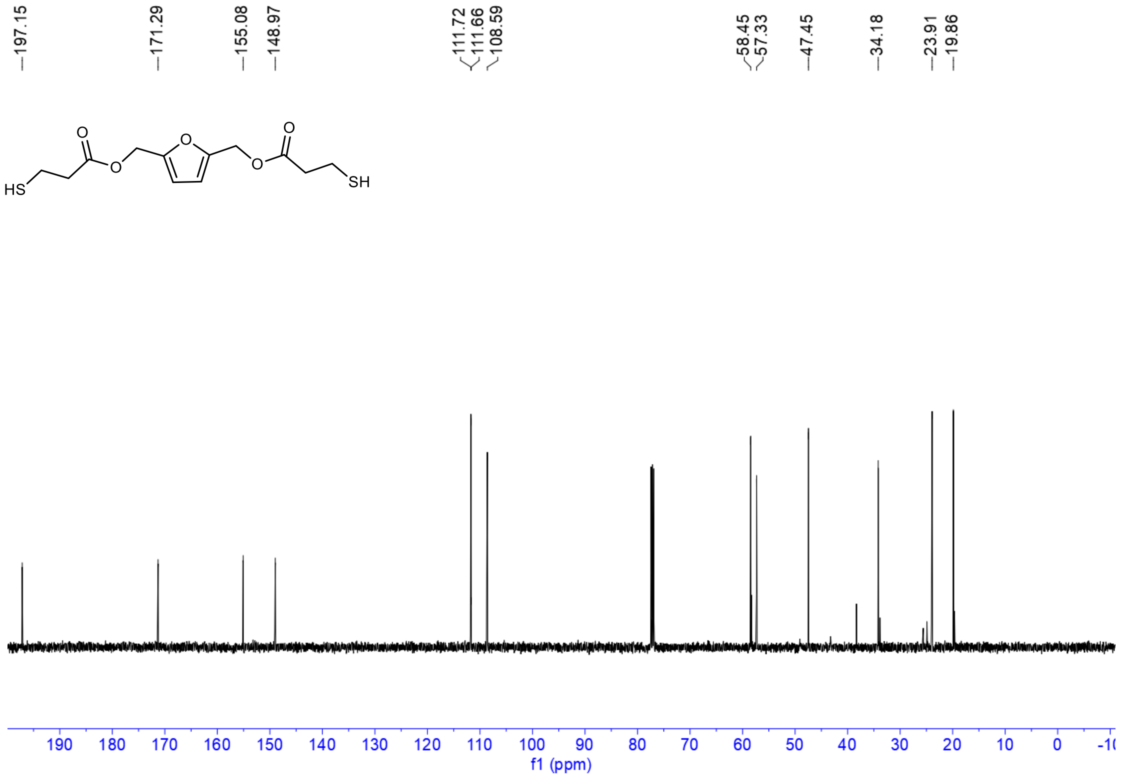

Supplementary Figure 127 | ^{1}H NMR (500 MHz, 298K, $CDCl_3$) of (Naphthalene-2,7-diylbis(oxy))bis(propane-3,1-diyl) bis(3-mercaptopropanoate) **(39a)**

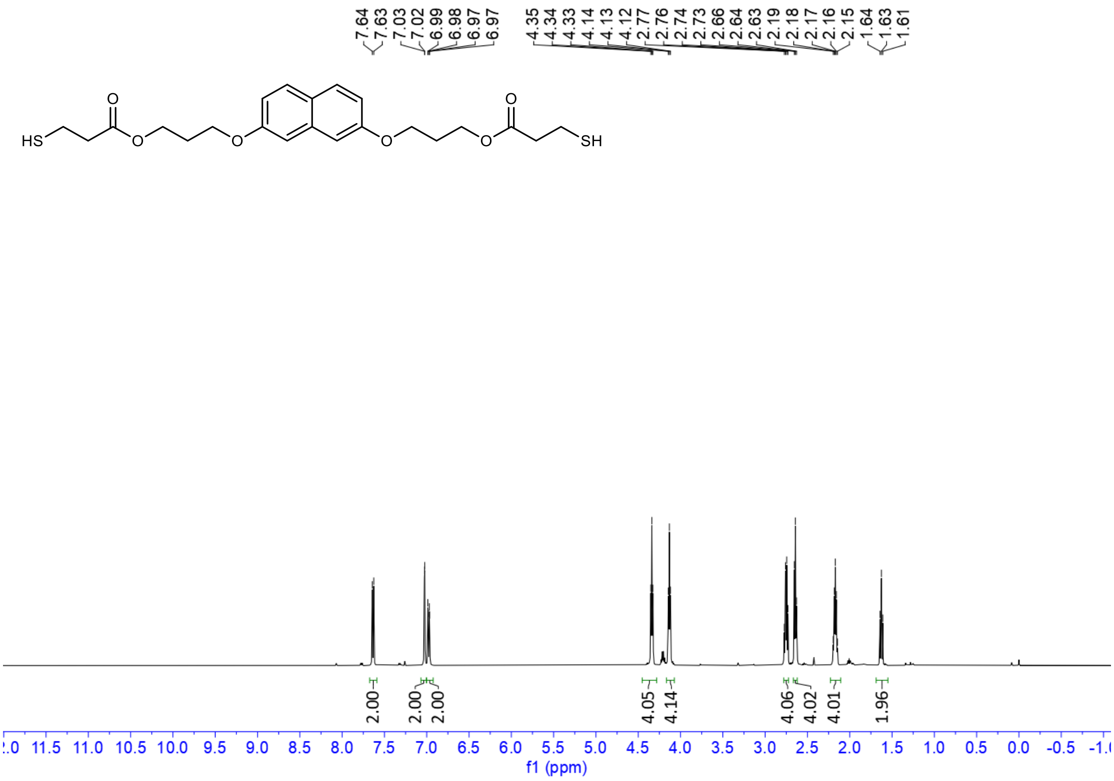

Supplementary Figure 128 | ^{13}C NMR (126 MHz, 298K, $CDCl_3$) of (Naphthalene-2,7-diylbis(oxy))bis(propane-3,1-diyl) bis(3-mercaptopropanoate) **(39a)**

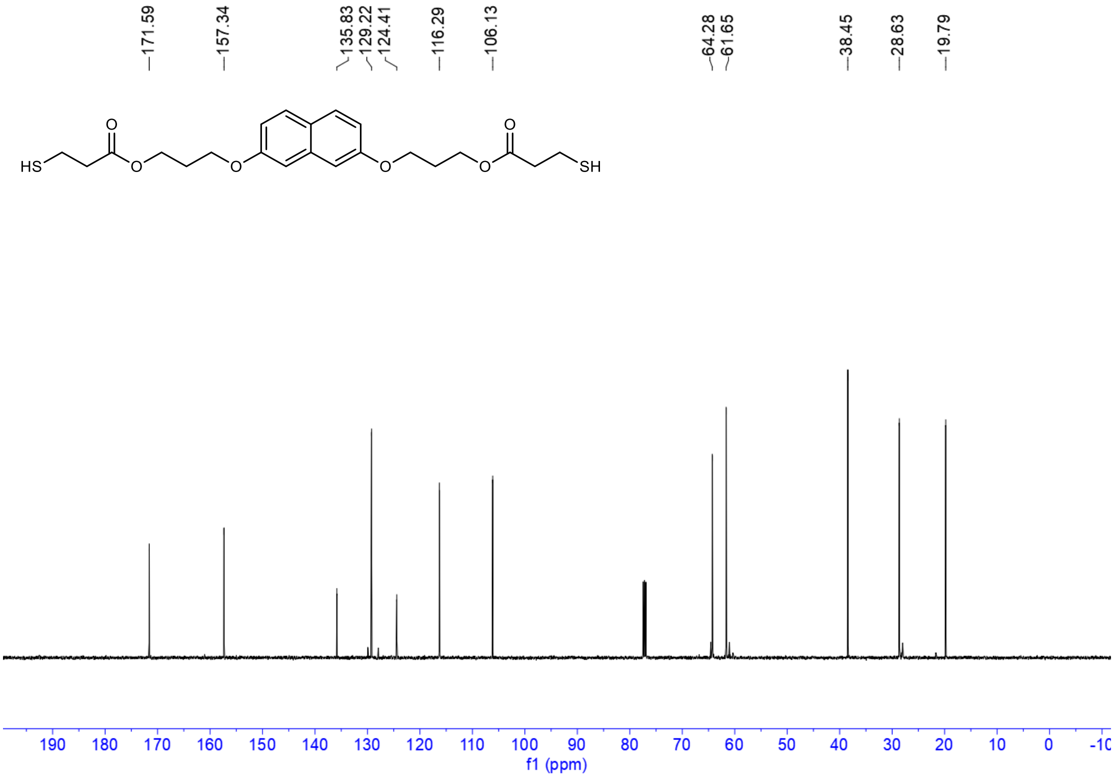

Supplementary Figure 129 | ^{1}H NMR (500 MHz, 298K, $CDCl_3$) of Bis(6-mercaptohexyl) 2,2'-(1,2-phenylenebis(oxy))diacetate **(35a)**

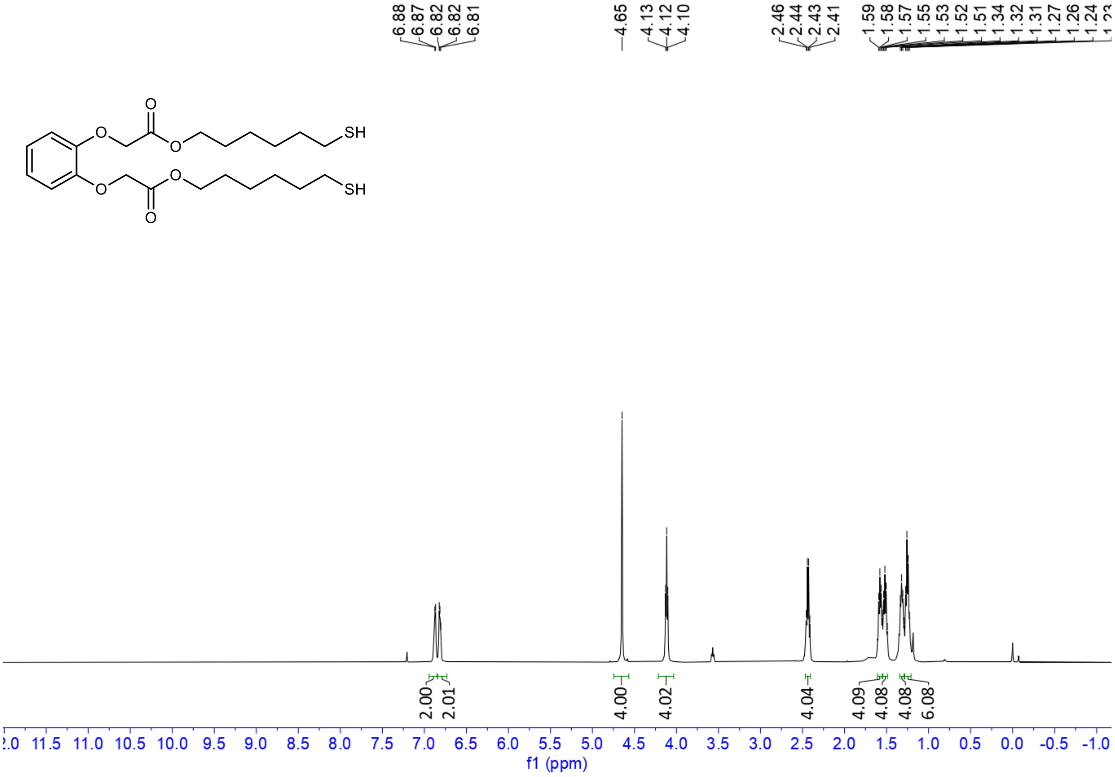

Supplementary Figure 130 | ^{13}C NMR (126 MHz, 298K, $CDCl_3$) of Bis(6-mercaptohexyl) 2,2'-(1,2-phenylenebis(oxy))diacetate **(35a)**

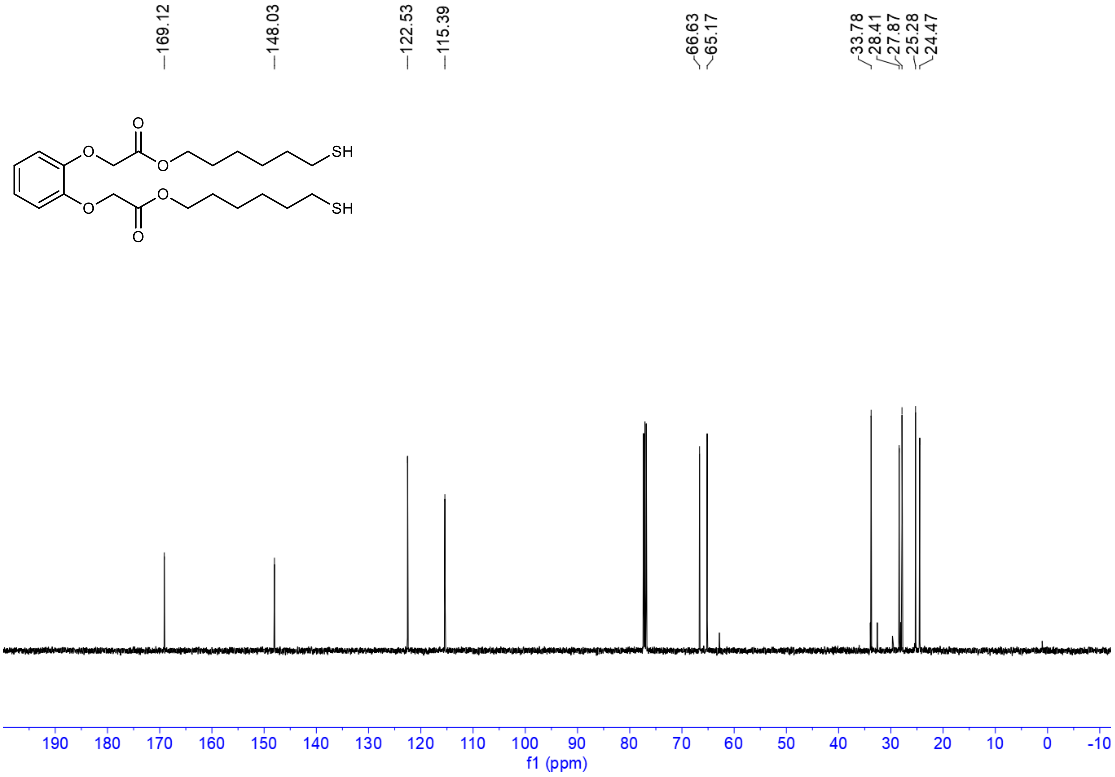

Supplementary Figure 131 | ^{1}H NMR (500 MHz, 298K, $CDCl_3$) of (1,4-Phenylenebis(oxy))bis (ethane-2,1-diyl) bis(3-mercaptopropanoate) **(38a)**

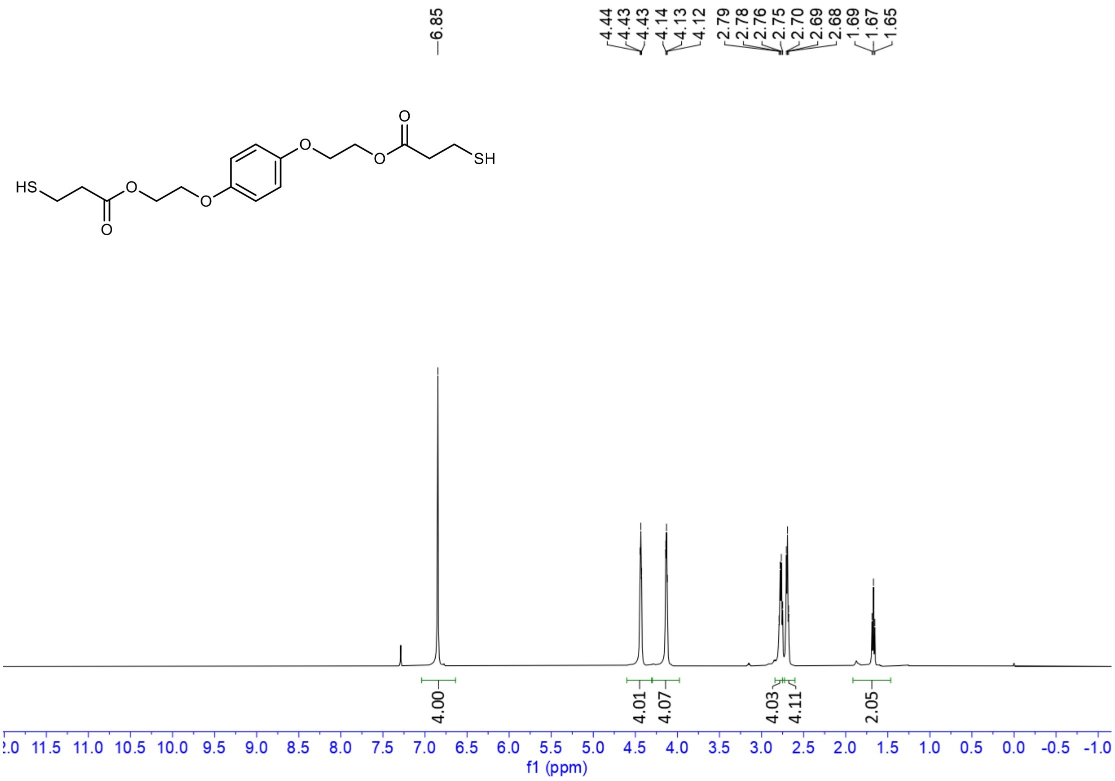

Supplementary Figure 132 | ^{13}C NMR (126 MHz, 298K, $CDCl_3$) of (1,4-Phenylenebis(oxy))bis (ethane-2,1-diyl) bis(3-mercaptopropanoate) **(38a)**

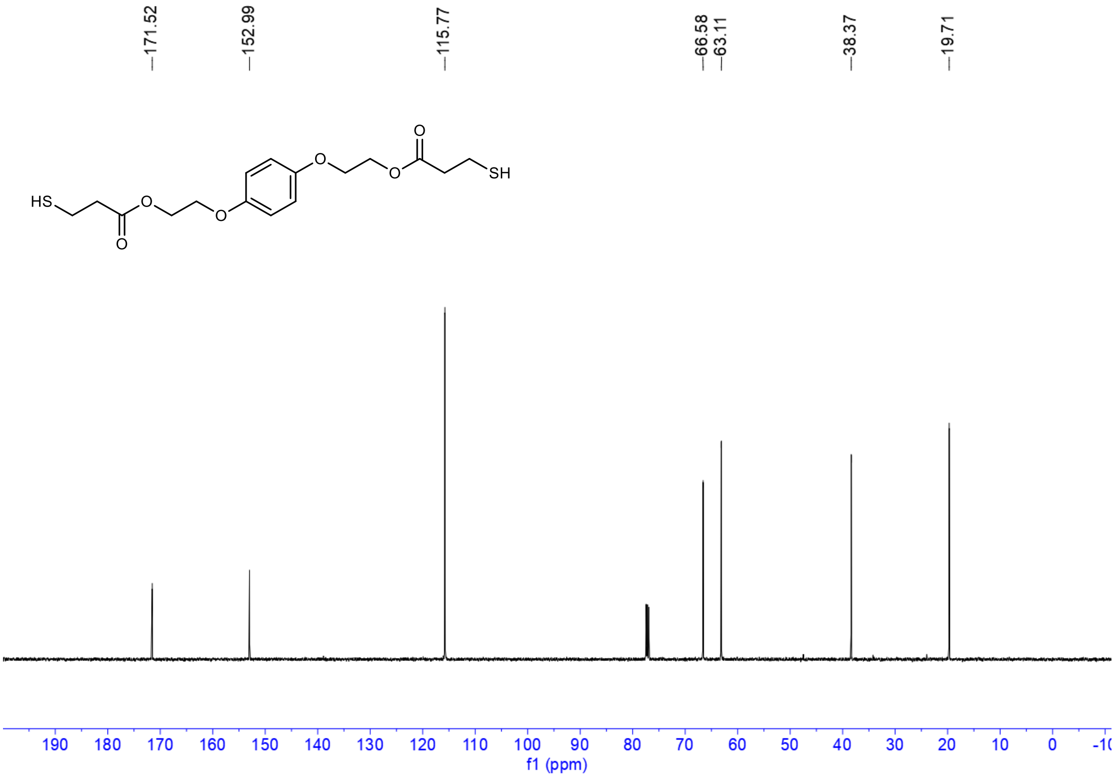

Supplementary Figure 134 | ^{1}H NMR (500 MHz, 298K, $CDCl_3$) of (*R*)-(+)-*S*,*S'*-(([1,1'-Binaphthalene]-2,2'-diylbis(oxy))bis(propane-3,1-diyl)) diethanethioate **(43a)**

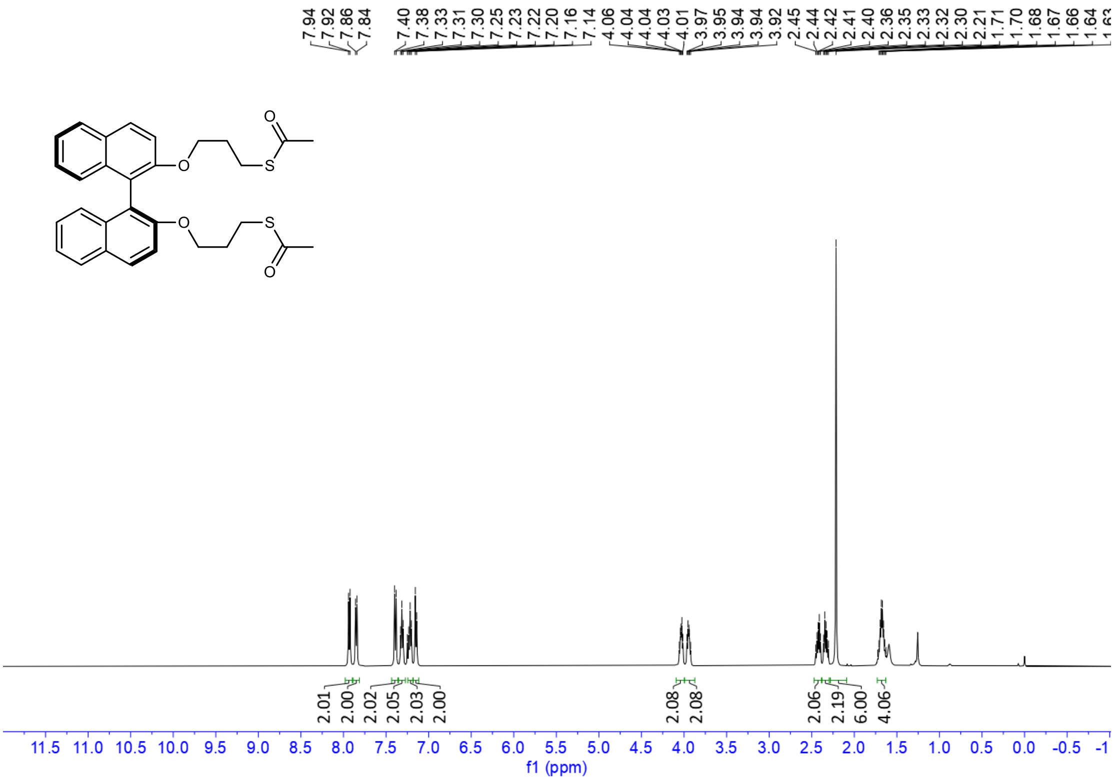

Supplementary Figure 135 | ^{13}C NMR (126 MHz, 298K, $CDCl_3$) of (*R*)-(+)-*S*,*S'*-(([1,1'-Binaphthalene]-2,2'-diylbis(oxy))bis(propane-3,1-diyl)) diethanethioate **(43a)**

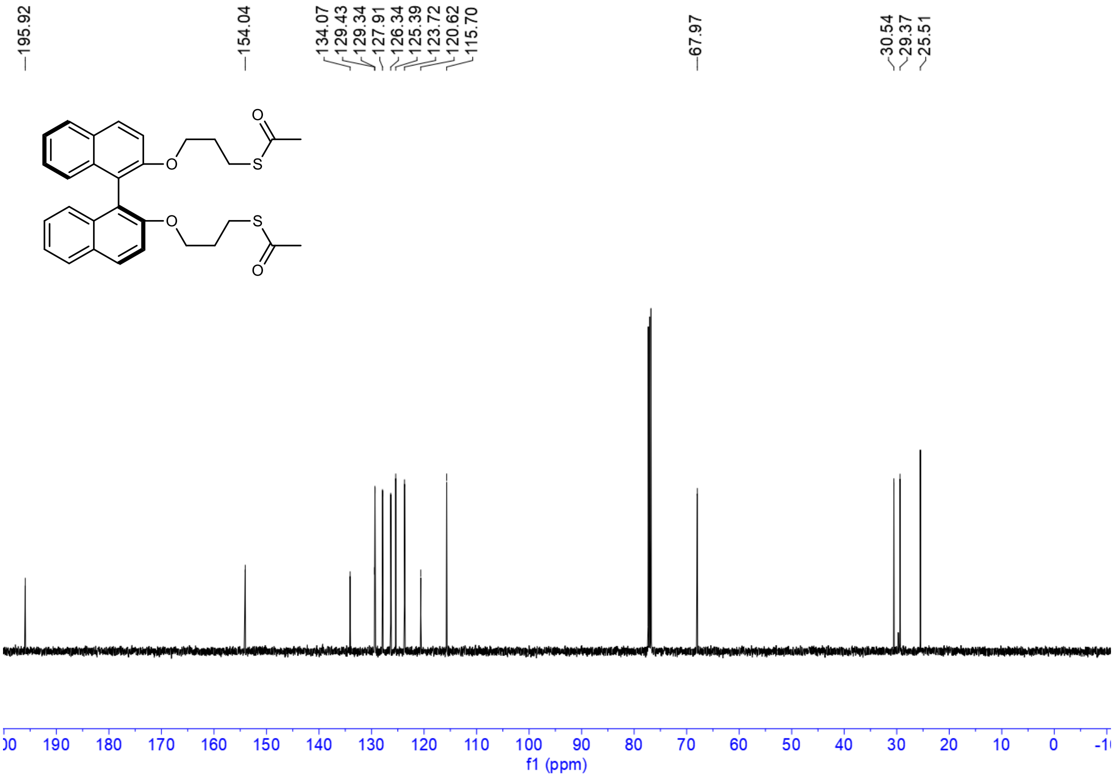

Supplementary Figure 136 | ^{1}H NMR (500 MHz, 298K, $CDCl_3$) of (*R*)-(+)-*3,3'*-([1,1'-Binaphthalene]-2,2'-diylbis(oxy))bis(propane-1-thiol) **(43b)**

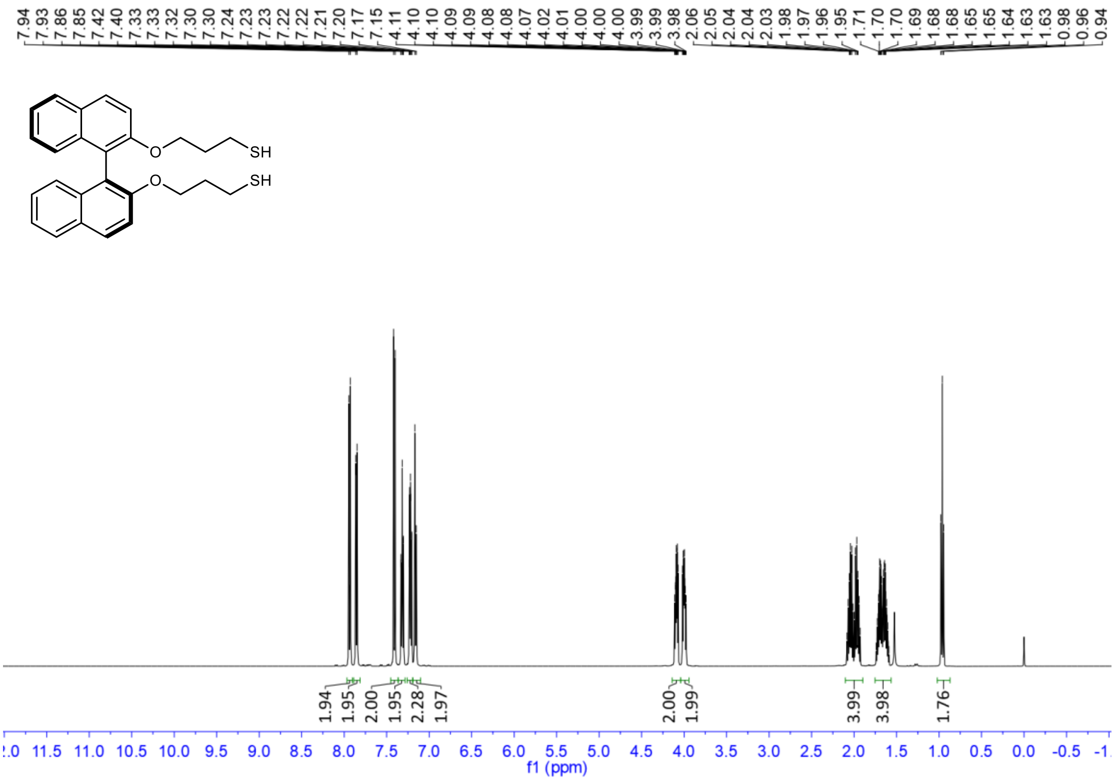

Supplementary Figure 137 | ^{13}C NMR (126 MHz, 298K, $CDCl_3$) of (*R*)-(+)-*3,3'*-([1,1'-Binaphthalene]-2,2'-diylbis(oxy))bis(propane-1-thiol) **(43b)**

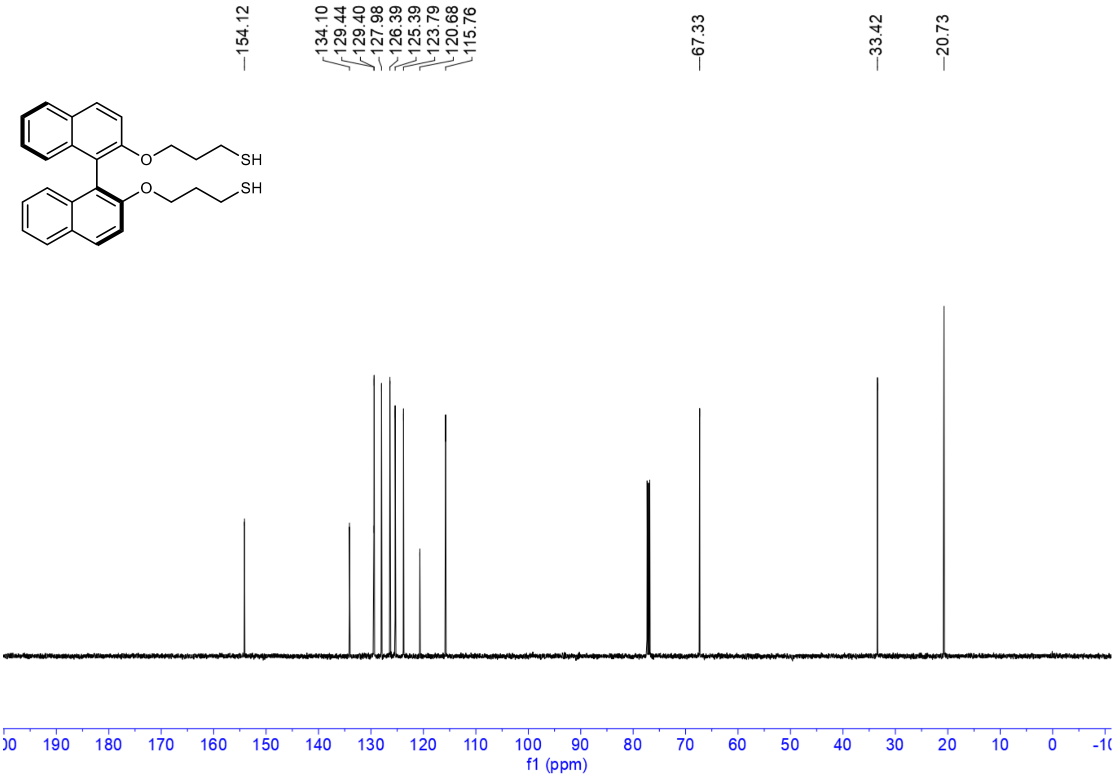

Supplementary Figure 138 | ^{1}H NMR (500 MHz, 298K, $CDCl_3$) of (*R*)-(+)-*S,S'*-(([1,1'-Binaphthalene]-2,2'-diylbis(oxy))bis(hexane-6,1-diyl)) diethanethioate **(44a)**

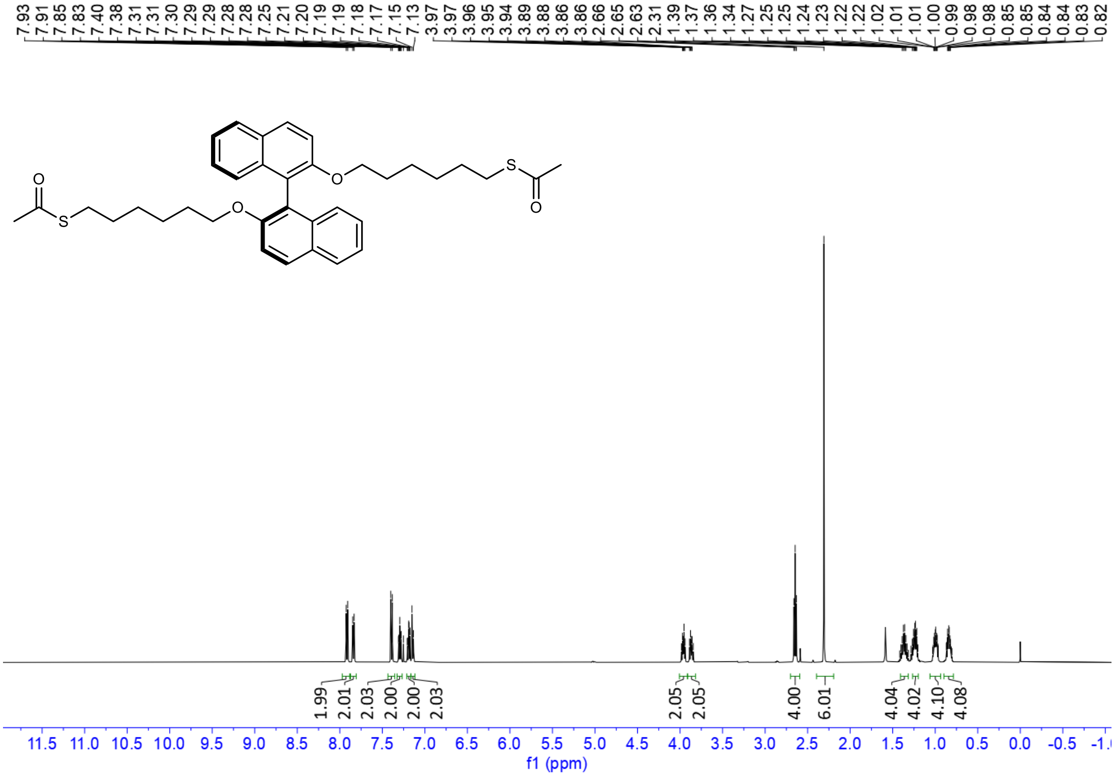

Supplementary Figure 139 | ^{13}C NMR (126 MHz, 298K, $CDCl_3$) of (*R*)-(+)-*S,S'*-(([1,1'-Binaphthalene]-2,2'-diylbis(oxy))bis(hexane-6,1-diyl)) diethanethioate **(44a)**

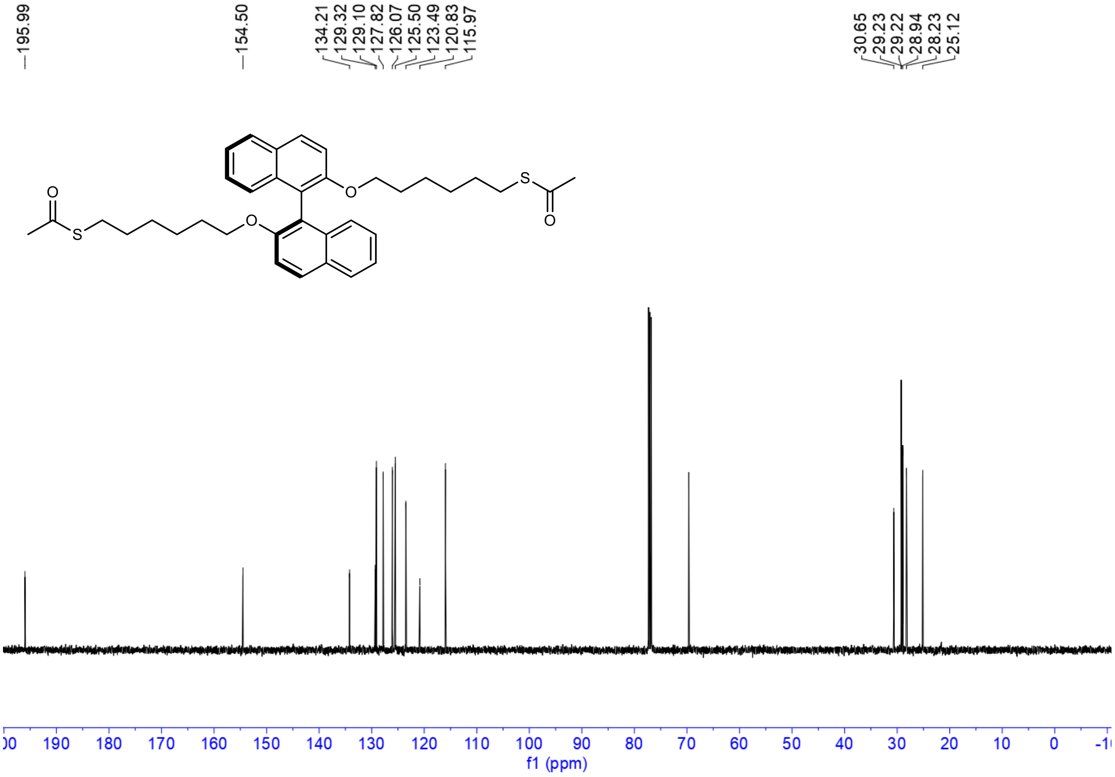

Supplementary Figure 140 | ^{1}H NMR (500 MHz, 298K, $CDCl_3$) of (*R*)-(+)-6,6'-([1,1'-Binaphthalene]-2,2'-diylbis(oxy))bis(hexane-1-thiol) **(44b)**

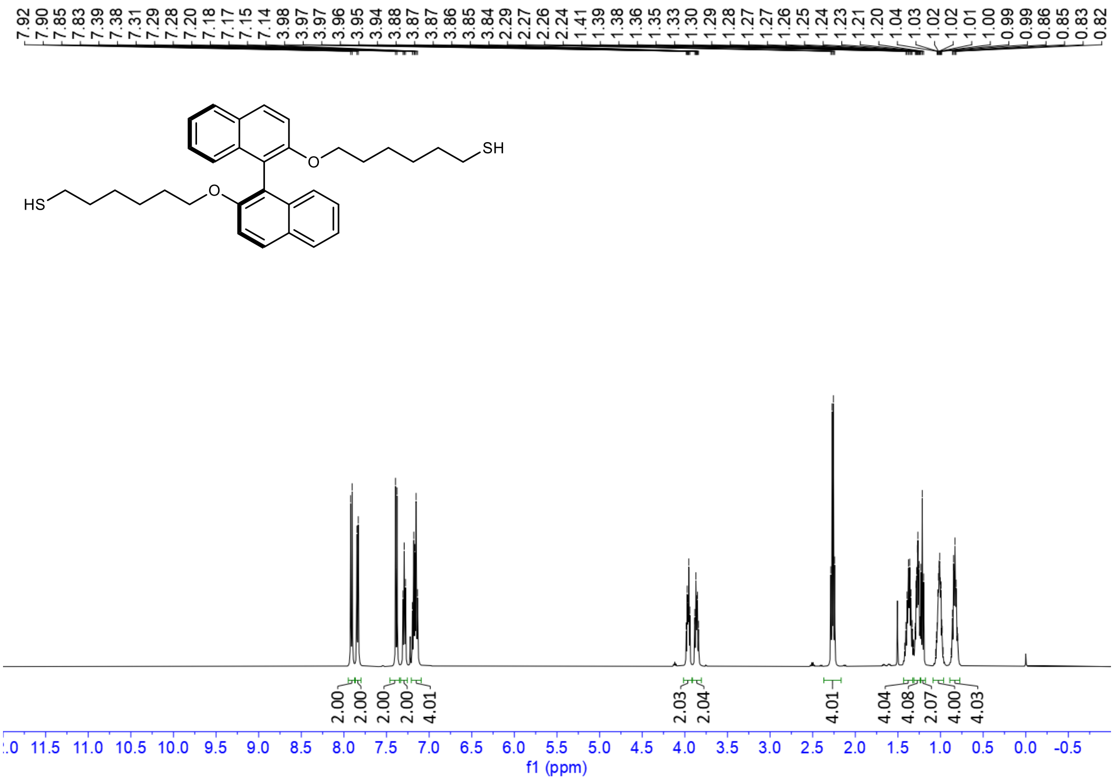

Supplementary Figure 141 | ^{13}C NMR (126 MHz, 298K, $CDCl_3$) of (*R*)-(+)-6,6'-([1,1'-Binaphthalene]-2,2'-diylbis(oxy))bis(hexane-1-thiol) **(44b)**

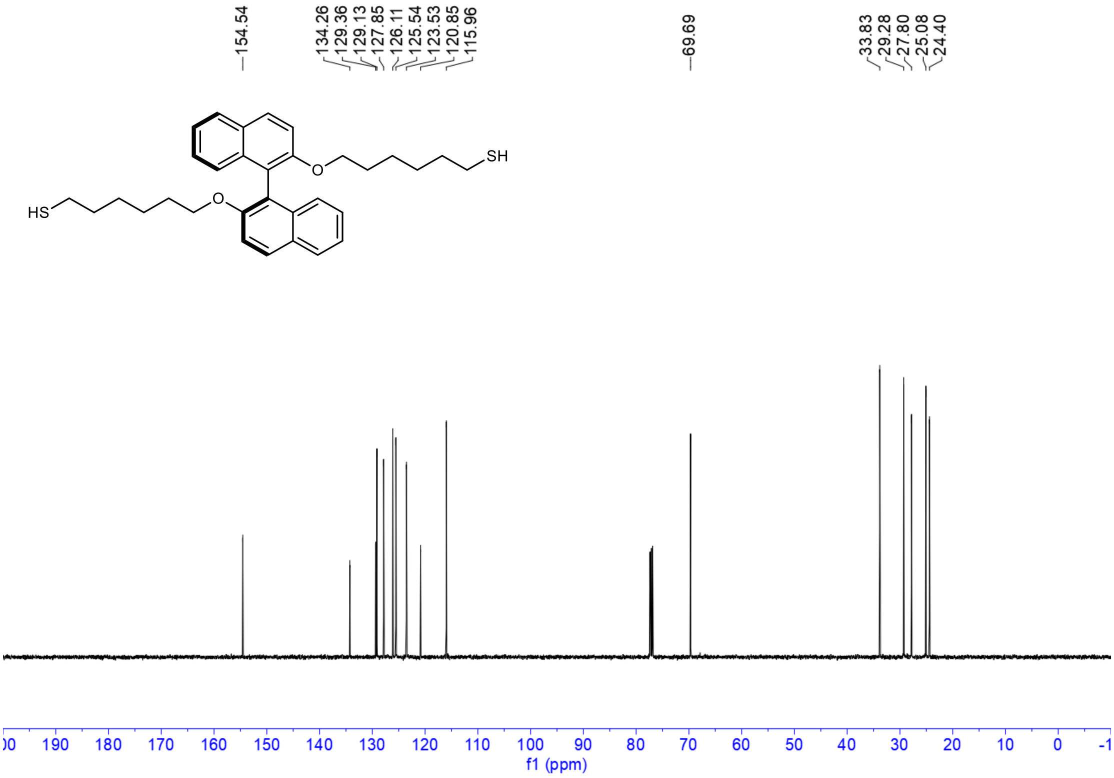

Supplementary Figure 142 | ^{1}H NMR (500 MHz, 298K, $CDCl_3$) of (*R*)-(+)-([1,1'-Binaphthalene]-2,2'-diylbis(oxy))bis(propane-3,1-diyl) bis(3-mercaptopropanoate) **(45a)**

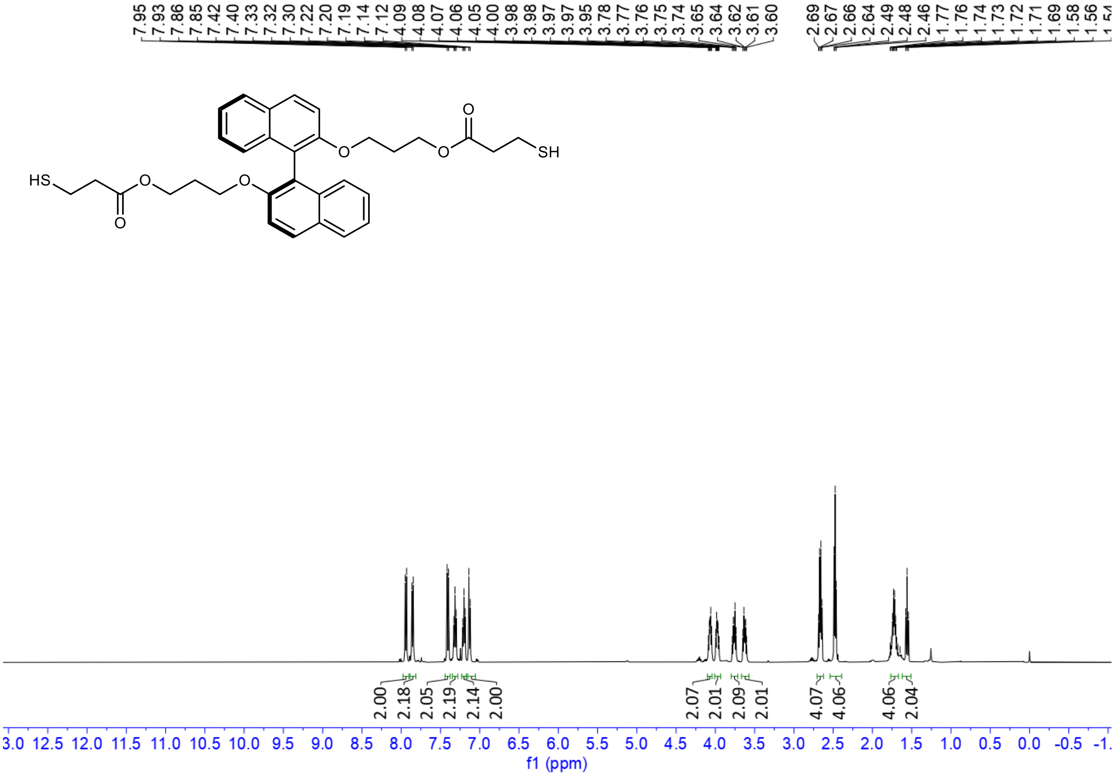

Supplementary Figure 143 | ^{13}C NMR (126 MHz, 298K, $CDCl_3$) of (*R*)-(+)-([1,1'-Binaphthalene]-2,2'-diylbis(oxy))bis(propane-3,1-diyl) bis(3-mercaptopropanoate) **(45a)**

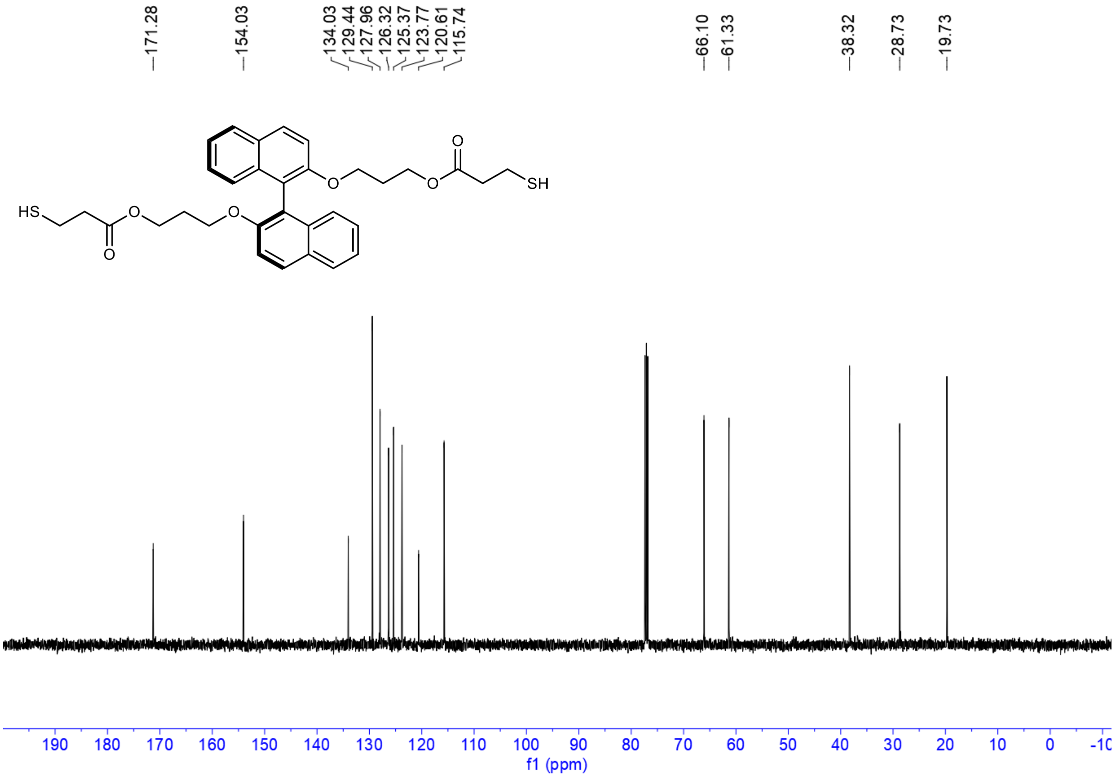

Supplementary Figure 144 | ^{1}H NMR (500 MHz, 298K, $CDCl_3$) of (*R*)-(+)-*S*,*S'*-(([1,1'-Binaphthalene]-2,2'-diylbis(oxy))bis(octane-8,1-diyl)) diethanethioate **(46a)**

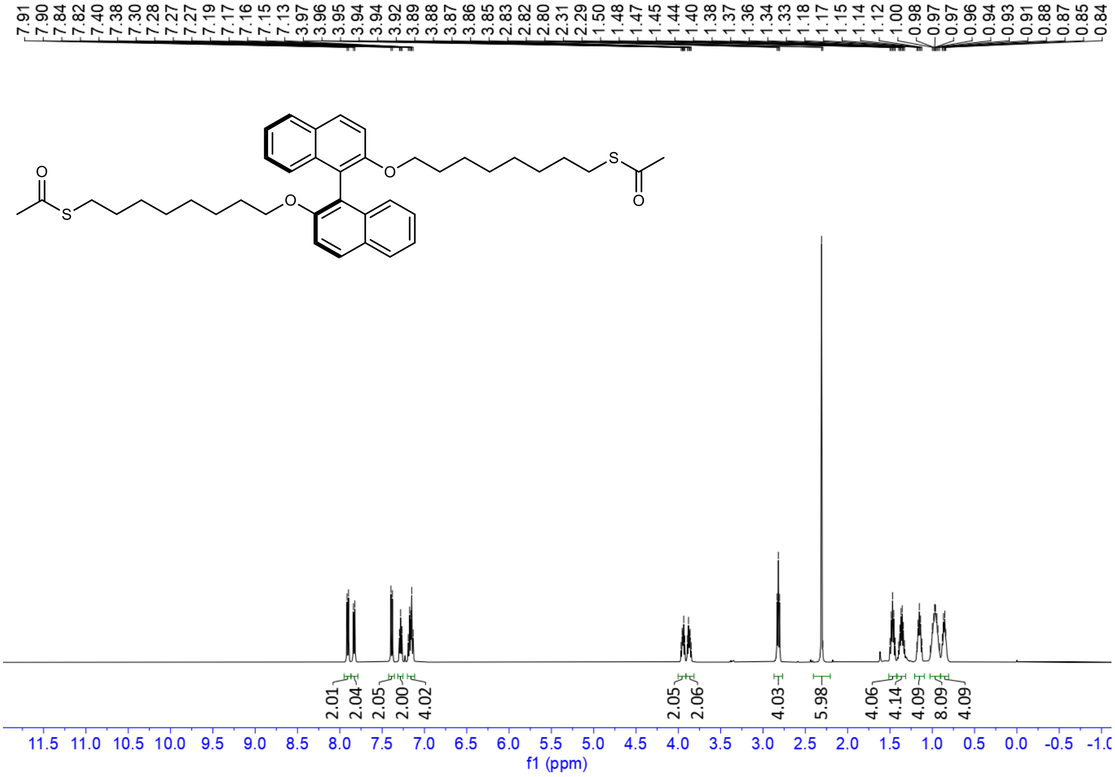

Supplementary Figure 145 | ^{13}C NMR (126 MHz, 298K, $CDCl_3$) of (*R*)-(+)-*S*,*S'*-(([1,1'-Binaphthalene]-2,2'-diylbis(oxy))bis(octane-8,1-diyl)) diethanethioate **(46a)**

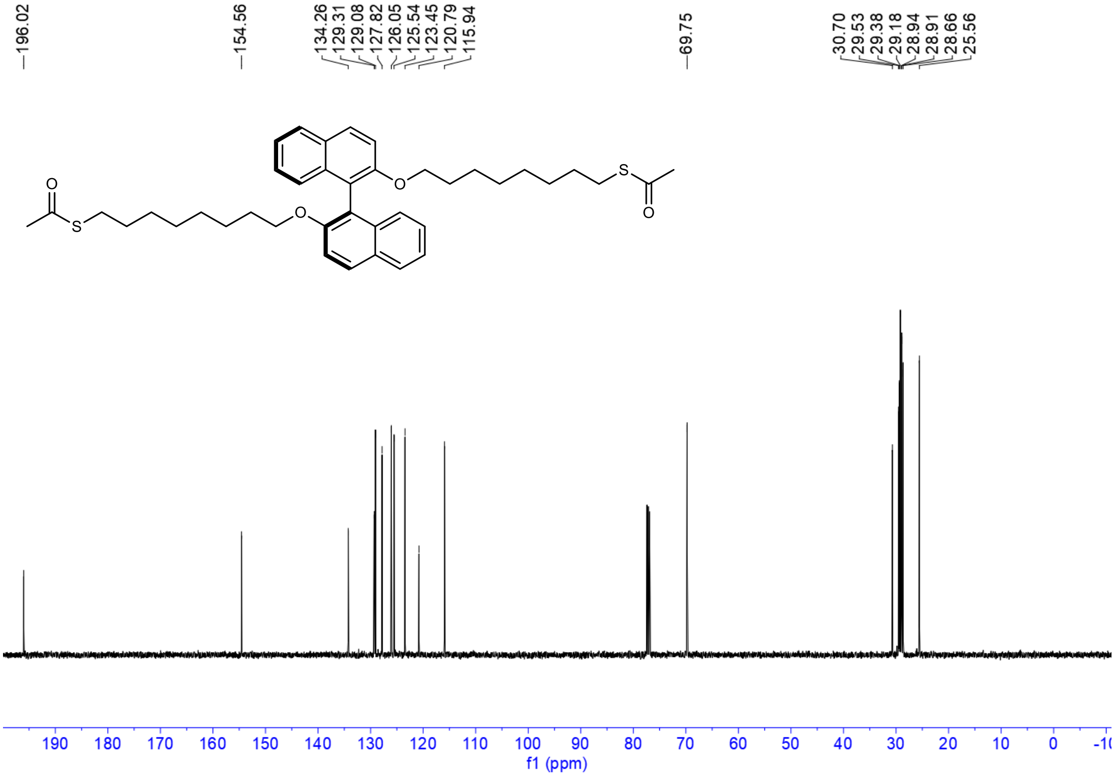

Supplementary Figure 146 | ^{1}H NMR (500 MHz, 298K, $CDCl_3$) of (*R*)-(+)-8,8'-([1,1'-Binaphthalene]-2,2'-diylbis(oxy))bis(octane-1-thiol) **(46b)**

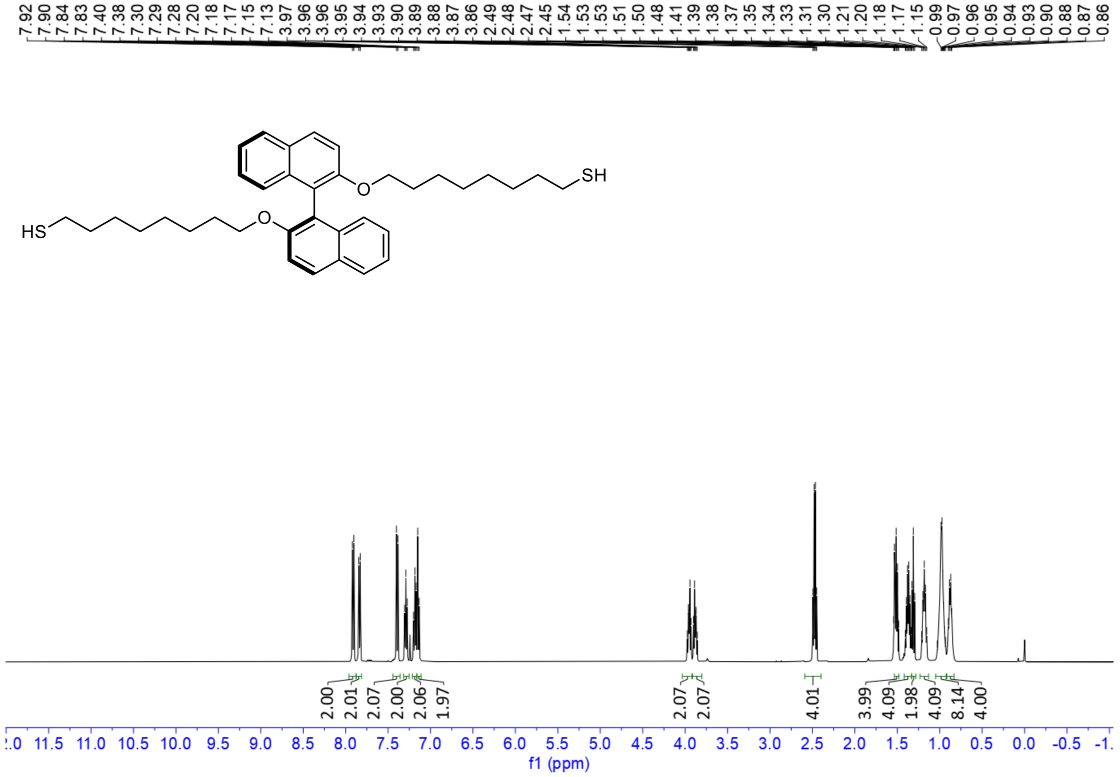

Supplementary Figure 147 | ^{13}C NMR (126 MHz, 298K, $CDCl_3$) of (*R*)-(+)-8,8'-([1,1'-Binaphthalene]-2,2'-diylbis(oxy))bis(octane-1-thiol) **(46b)**

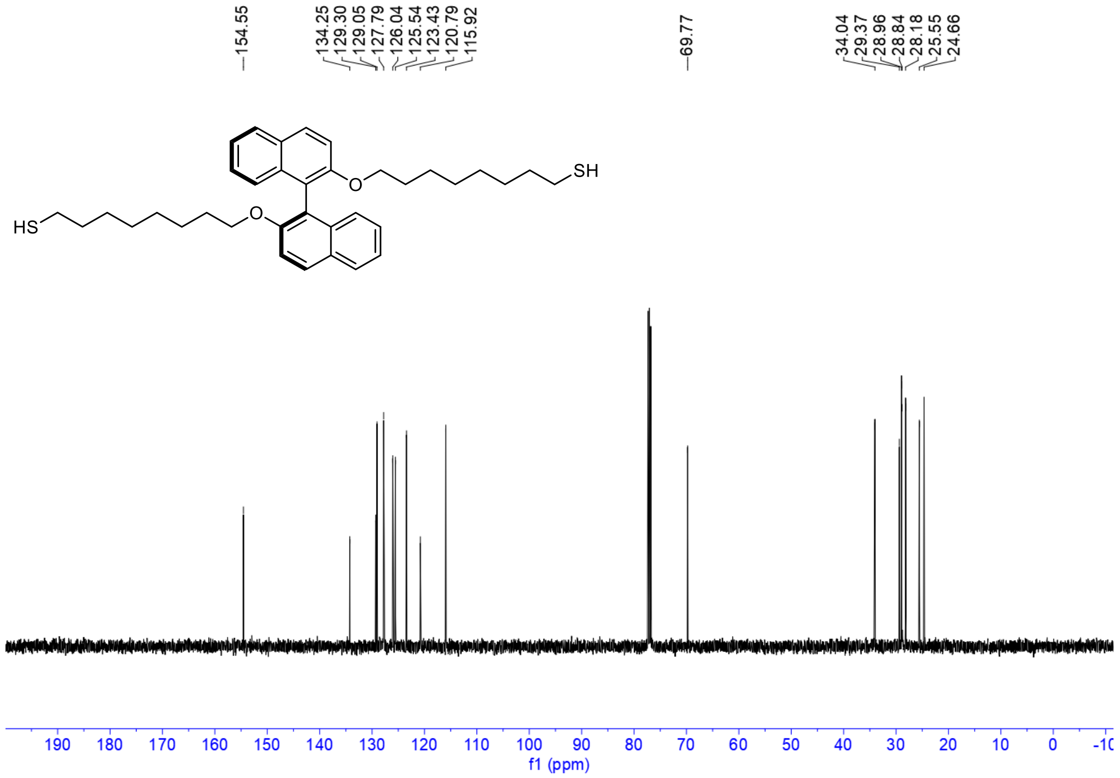

Supplementary Figure 148 | ^{1}H NMR (500 MHz, 298K, $CDCl_3$) of (*R*)-(+)-([1,1'-Binaphthalene]-2,2'-diylbis(oxy))bis(pentane-5,1-diyl) bis(3-mercaptopropanoate) **(47a)**

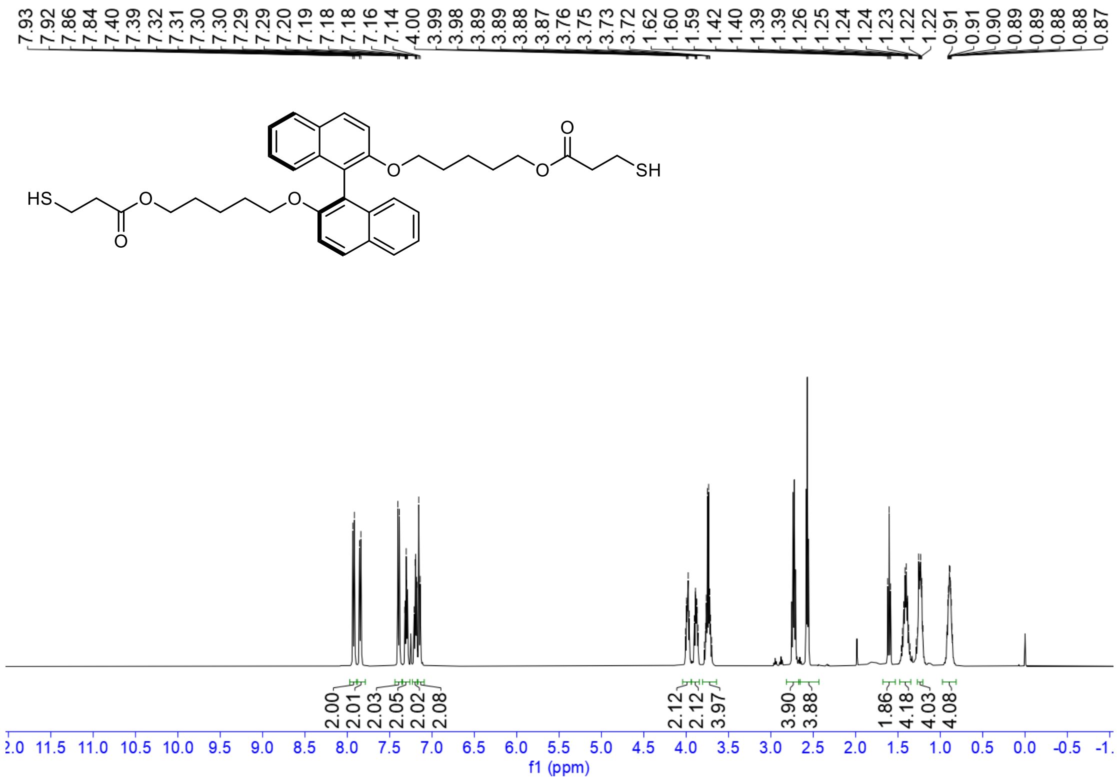

Supplementary Figure 149 | ^{13}C NMR (126 MHz, 298K, $CDCl_3$) of (*R*)-(+)-([1,1'-Binaphthalene]-2,2'-diylbis(oxy))bis(pentane-5,1-diyl) bis(3-mercaptopropanoate) **(47a)**

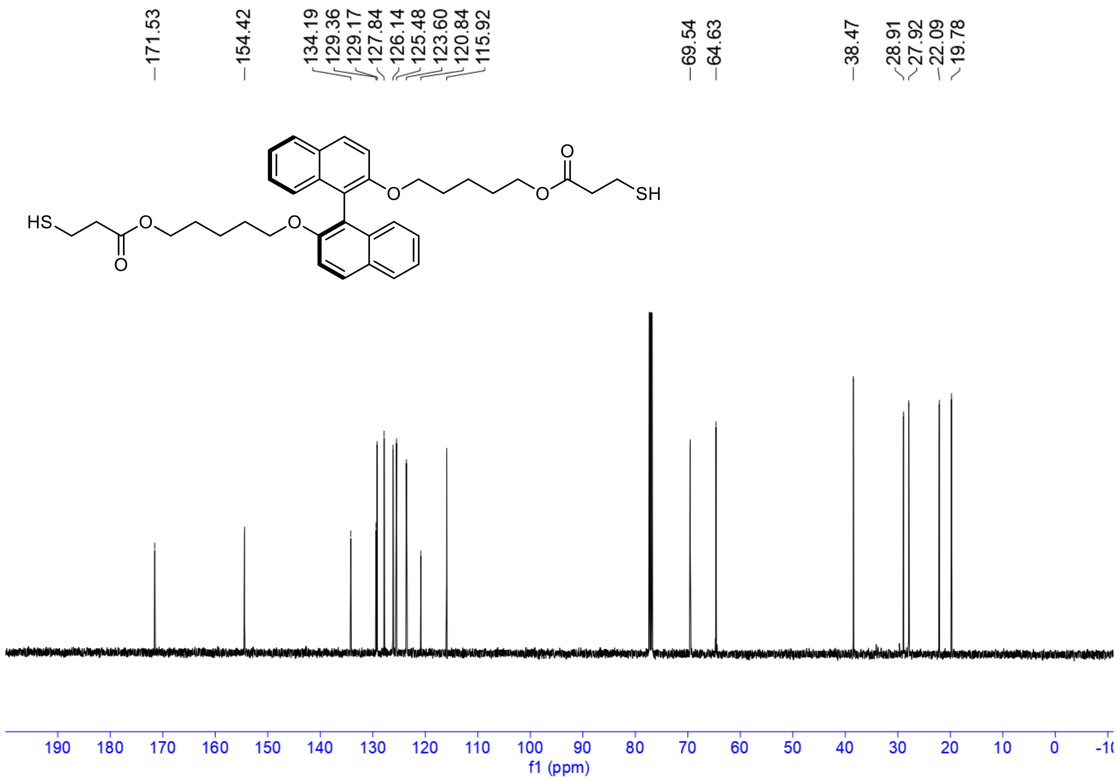

Supplementary Figure 150 | ^{1}H NMR (500 MHz, 298K, $CDCl_3$) of (*R*)-(+)-([1,1'-Binaphthalene]-2,2'-diylbis(oxy))bis(hexane-6,1-diyl) bis(3-mercaptopropanoate) **(48a)**

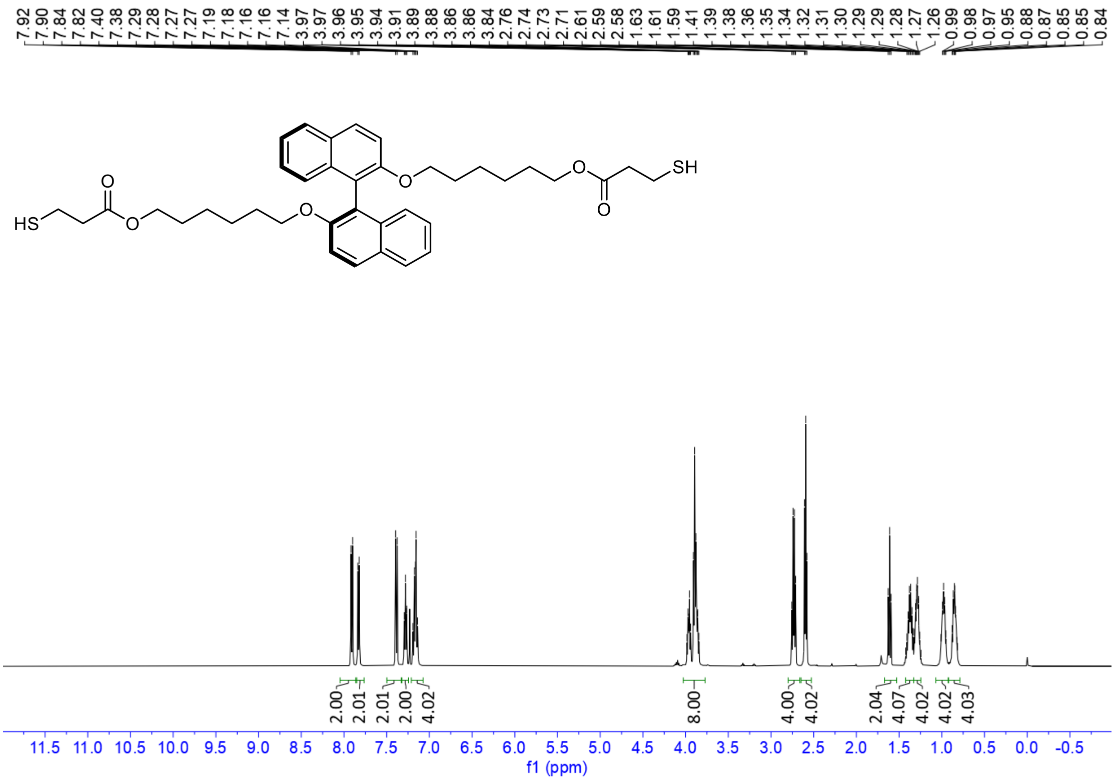

Supplementary Figure 151 | ^{13}C NMR (126 MHz, 298K, $CDCl_3$) of (*R*)-(+)-([1,1'-Binaphthalene]-2,2'-diylbis(oxy))bis(hexane-6,1-diyl) bis(3-mercaptopropanoate) **(48a)**

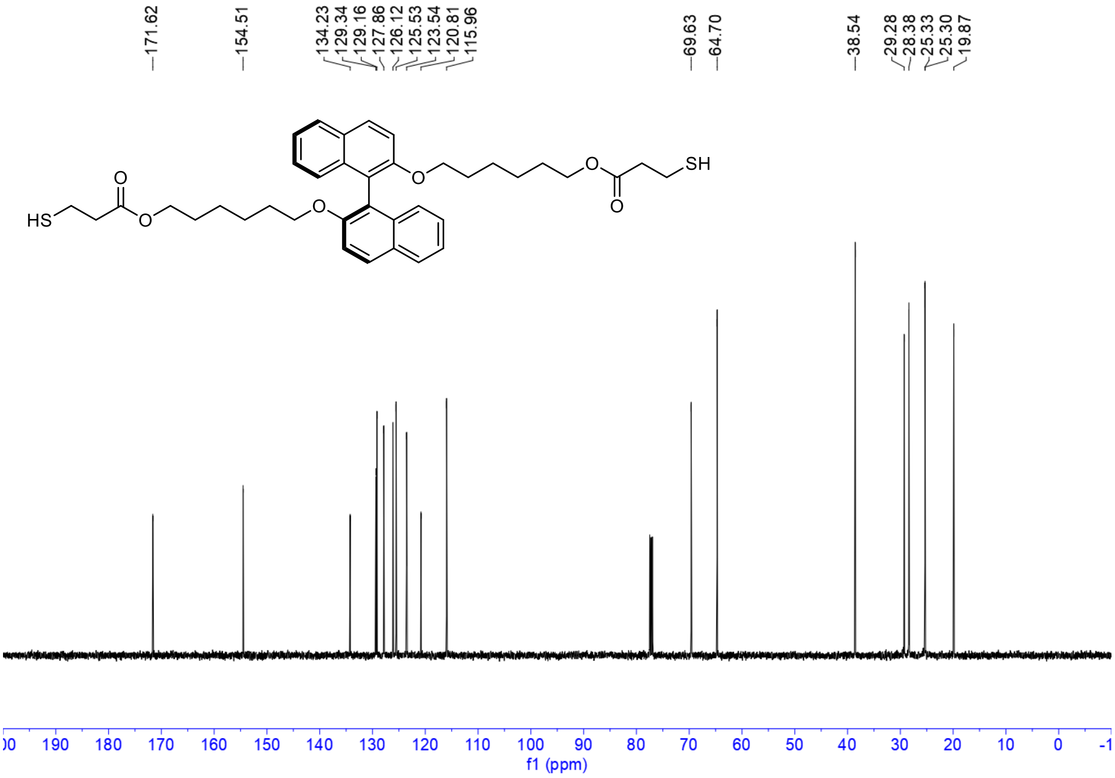

Supplementary Figure 150 | ^{1}H NMR (500 MHz, 298K, $CDCl_3$) (1*S*,2*S*)-*trans*-Cyclohexane-1,2-diyl bis(3-mercaptopropanoate) **(49a)**

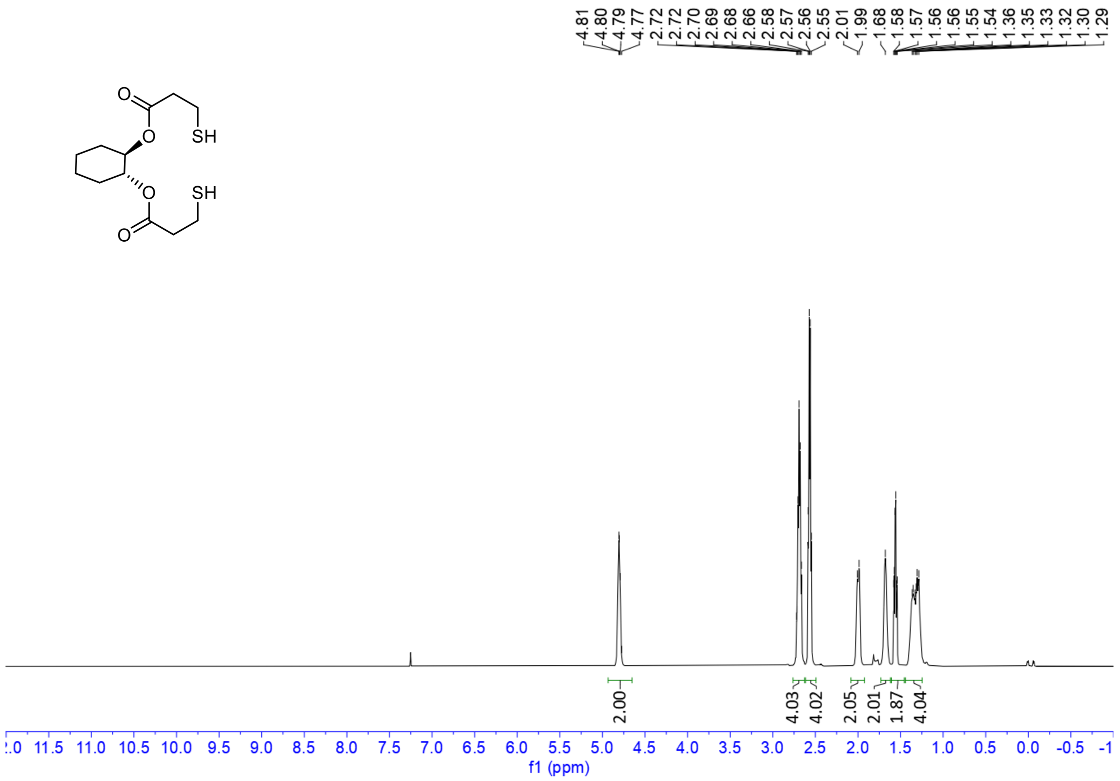

Supplementary Figure 151 | ^{13}C NMR (126 MHz, 298K, $CDCl_3$) (1*S*,2*S*)-*trans*-Cyclohexane-1,2-diyl bis(3-mercaptopropanoate) **(49a)**

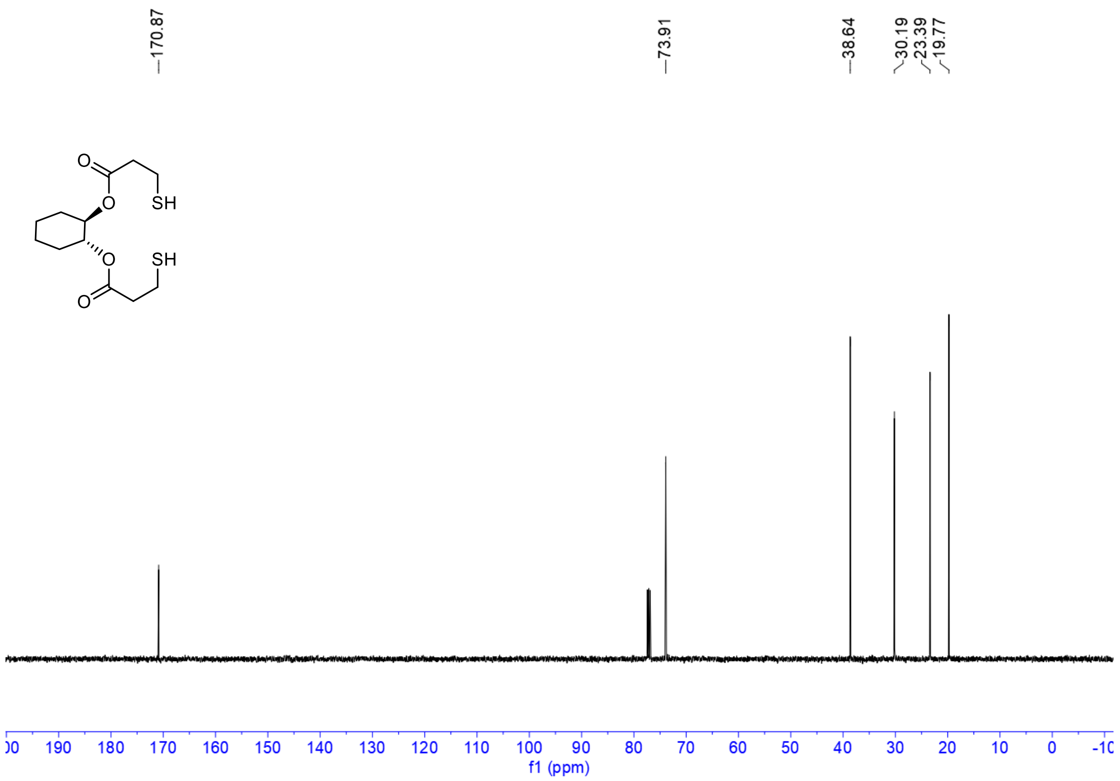

Supplementary Figure 152 | ^{1}H NMR (500 MHz, 298K, $CDCl_3$) of 6-Mercaptohexyl (*R*)-(2-mercaptopropanoyl)glycinate **(50a)**

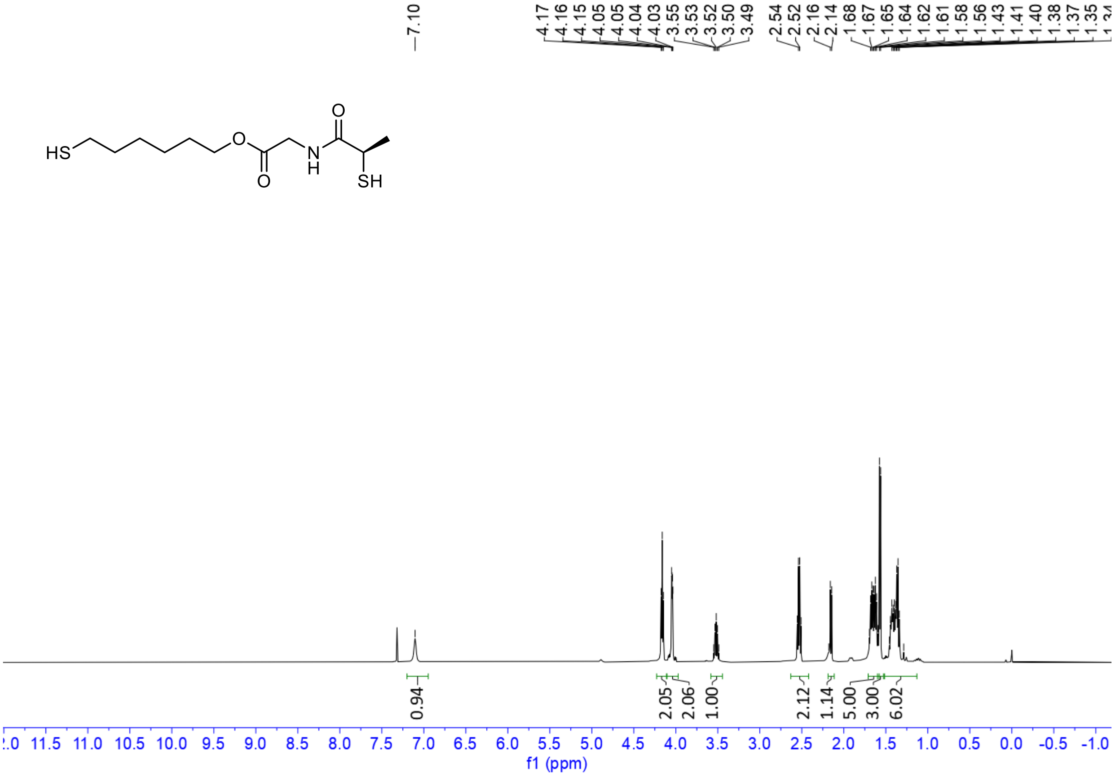

Supplementary Figure 153 | ^{13}C NMR (126 MHz, 298K, $CDCl_3$) of 6-Mercaptohexyl (*R*)-(2-mercaptopropanoyl)glycinate **(50a)**

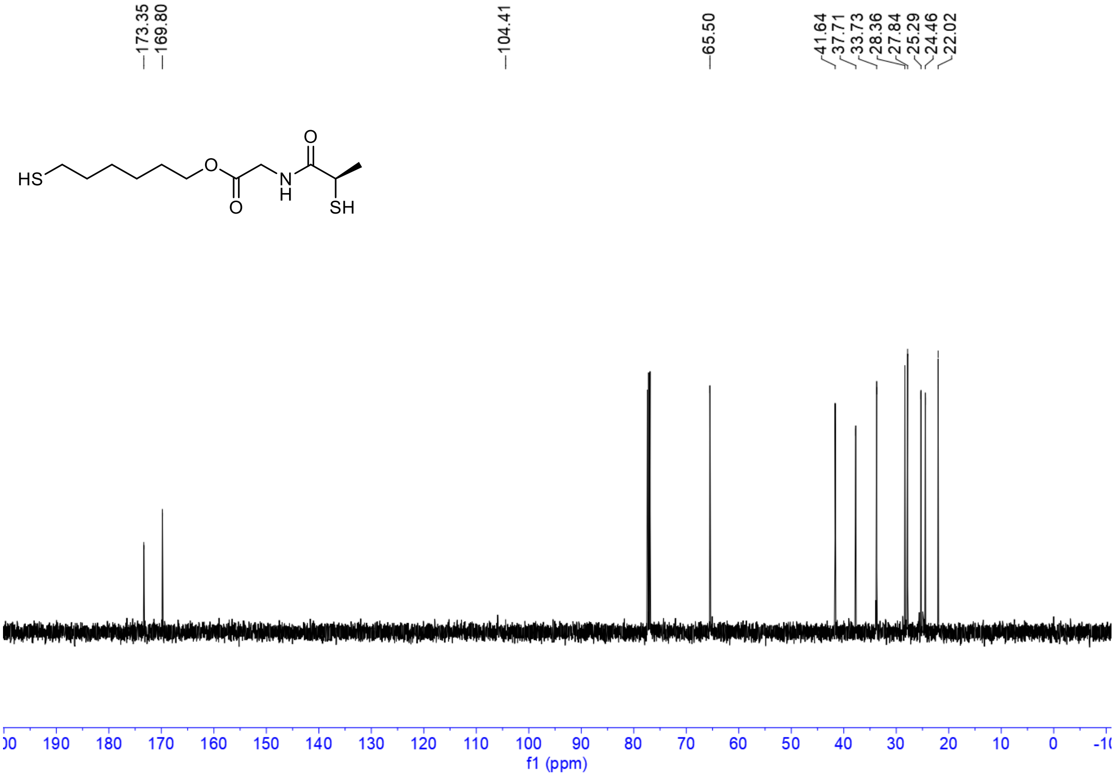

Supplementary Figure 154 | ^{1}H NMR (500 MHz, 298K, $CDCl_3$) of (*R*)-((2,2',3,3'-tetrahydro-1,1'-spirobi[indene]-7,7'-diyl)bis(oxy))bis(hexane-6,1-diyl) bis(3-mercaptopropanoate) **(51a)**

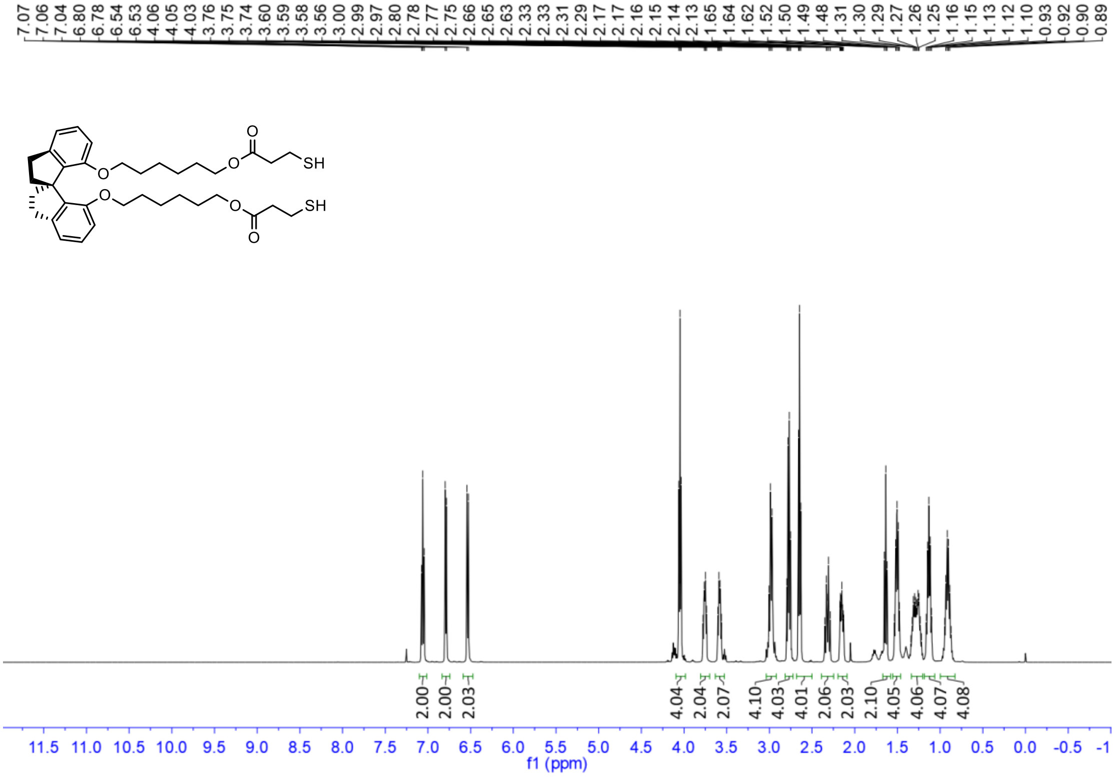

Supplementary Figure 155 | ^{13}C NMR (126 MHz, 298K, $CDCl_3$) of (*R*)-((2,2',3,3'-tetrahydro-1,1'-spirobi[indene]-7,7'-diyl)bis(oxy))bis(hexane-6,1-diyl) bis(3-mercaptopropanoate) **(51a)**

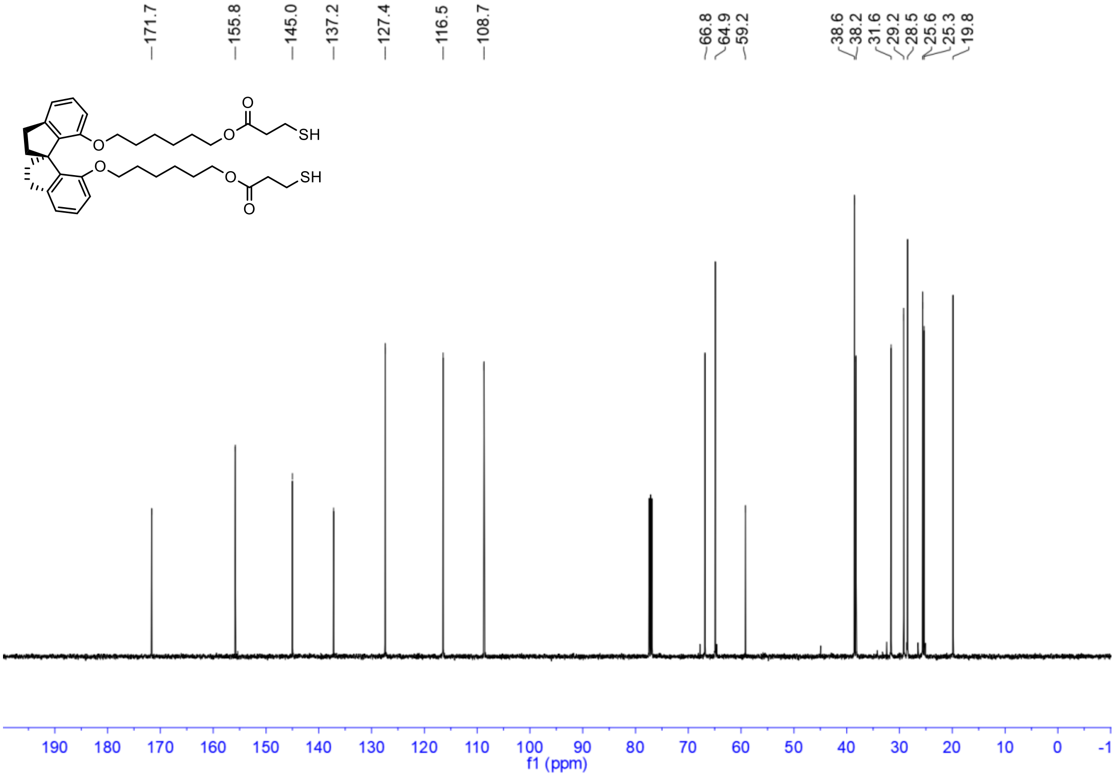

Supplementary Figure 156 | ^{1}H NMR (500 MHz, 298K, $CDCl_3$) of *N*-Octyl-*N*-(propan-2-yl-2-*d*)acetamide **(110a)**

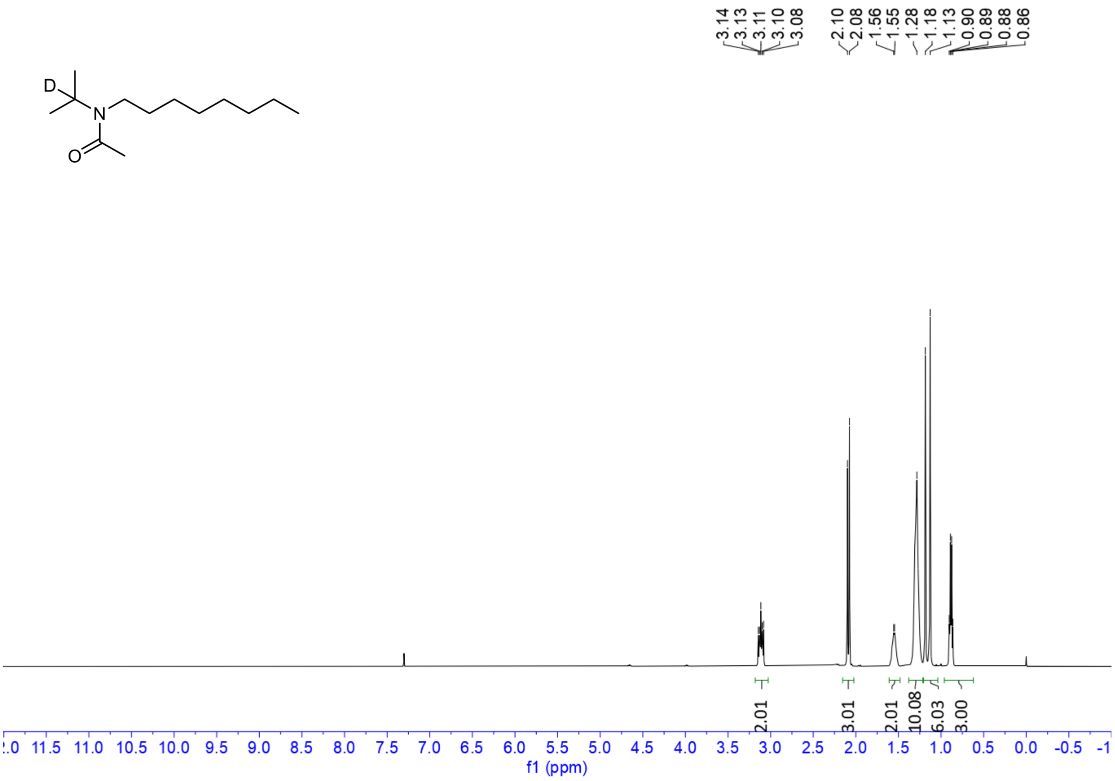

Supplementary Figure 157 | ^{13}C NMR (126 MHz, 298K, $CDCl_3$) of *N*-Octyl-*N*-(propan-2-yl-2-*d*)acetamide **(110a)**

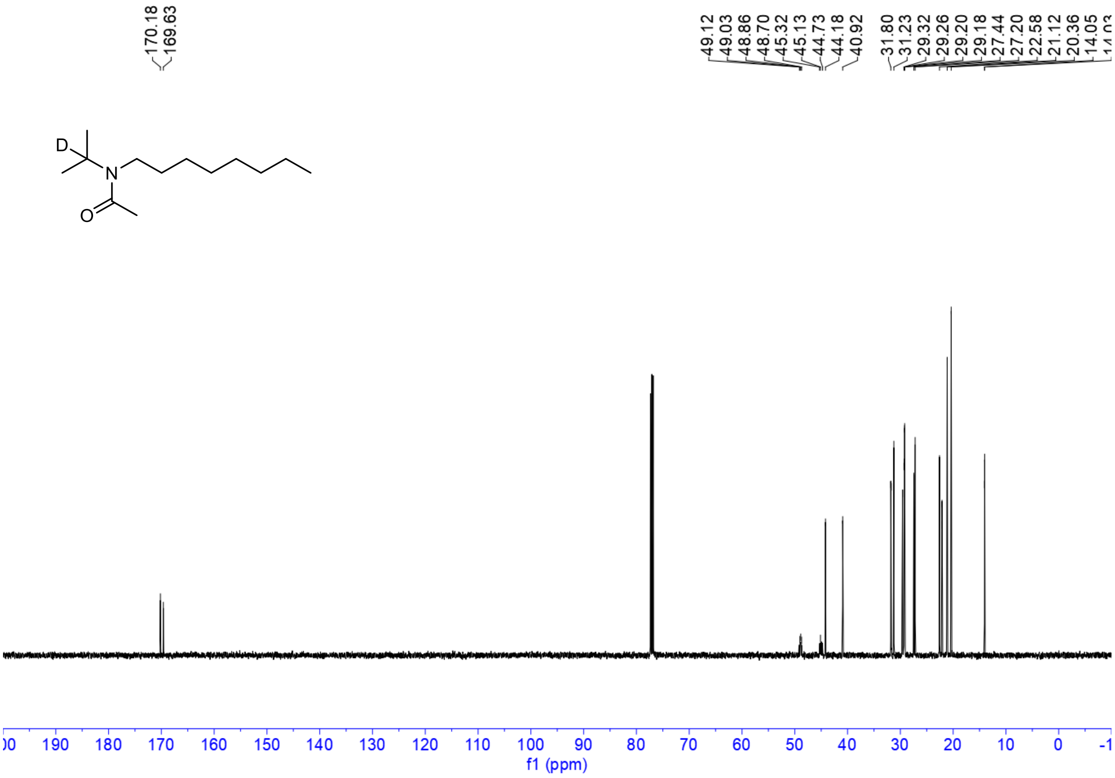

Supplementary Figure 158 | ^{1}H NMR (500 MHz, 298K, $CDCl_3$) of *N*-Ethyl-*N*-(propan-2-yl-2-*d*)octan-1-amine **(110b)**

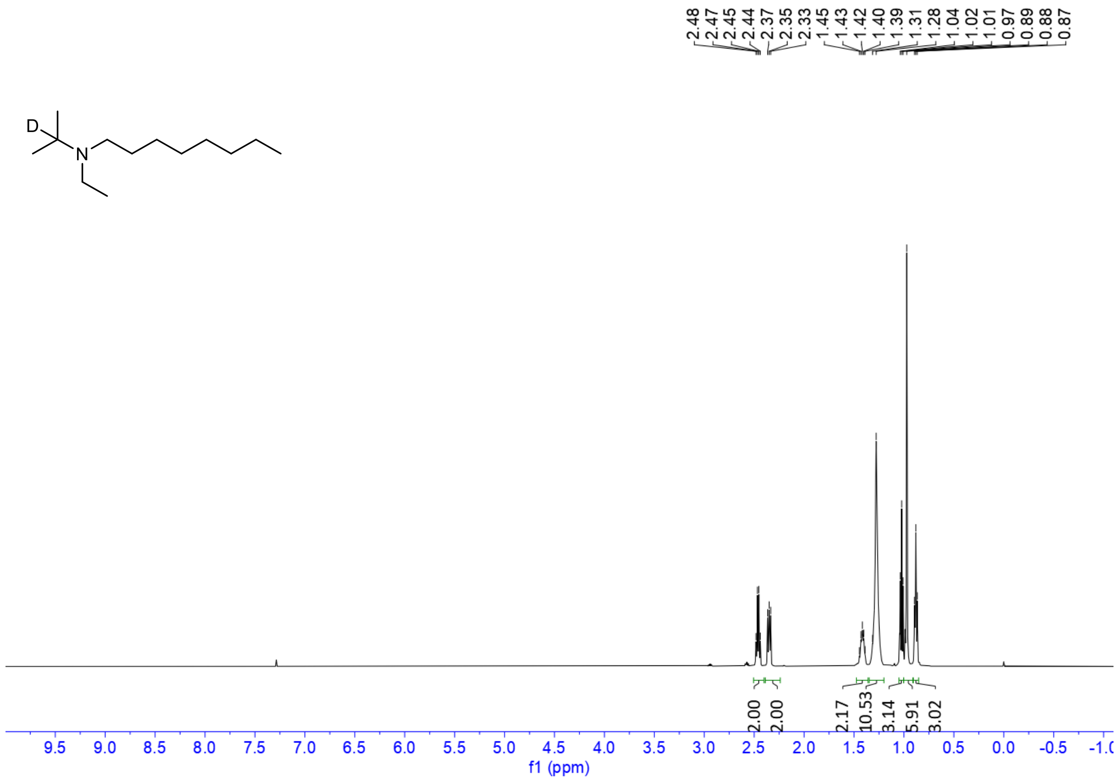

Supplementary Figure 159 | ^{13}C NMR (126 MHz, 298K, $CDCl_3$) of *N*-Ethyl-*N*-(propan-2-yl-2-*d*)octan-1-amine **(110b)**

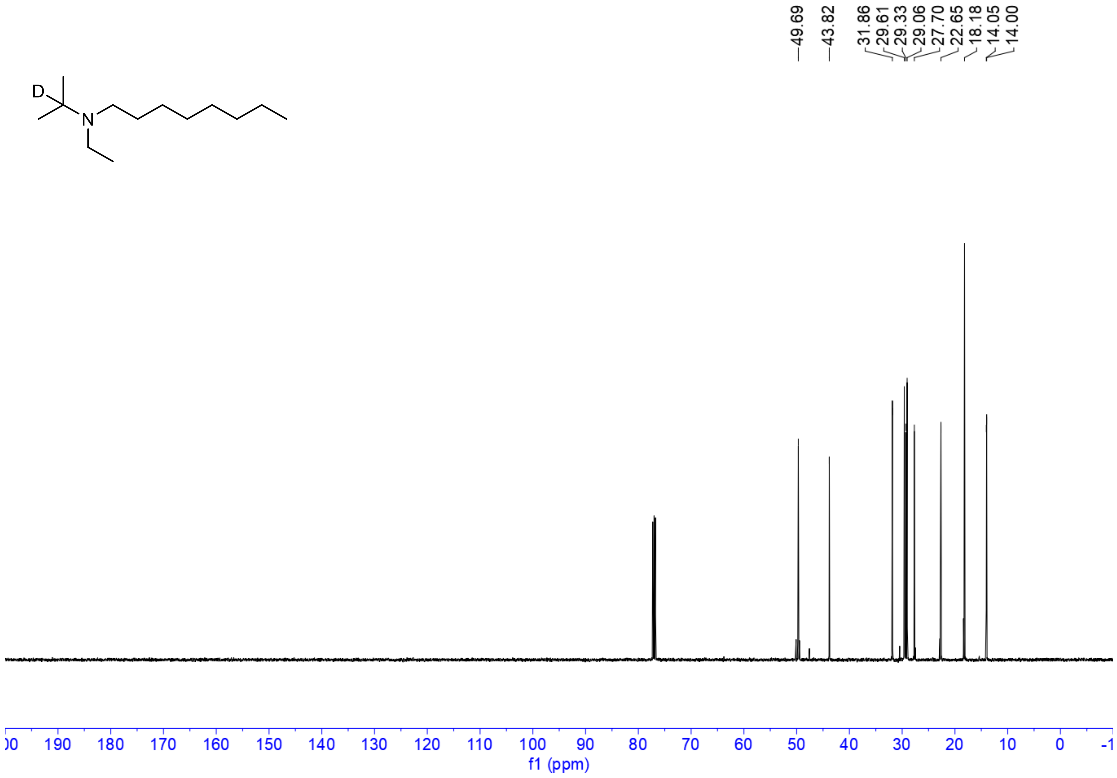

Supplementary Figure 160 | ^{1}H NMR (500 MHz, 298K, $CDCl_3$) of *N*-Octyl-*N*-(propan-2-yl-d_7)acetamide **(111a)**

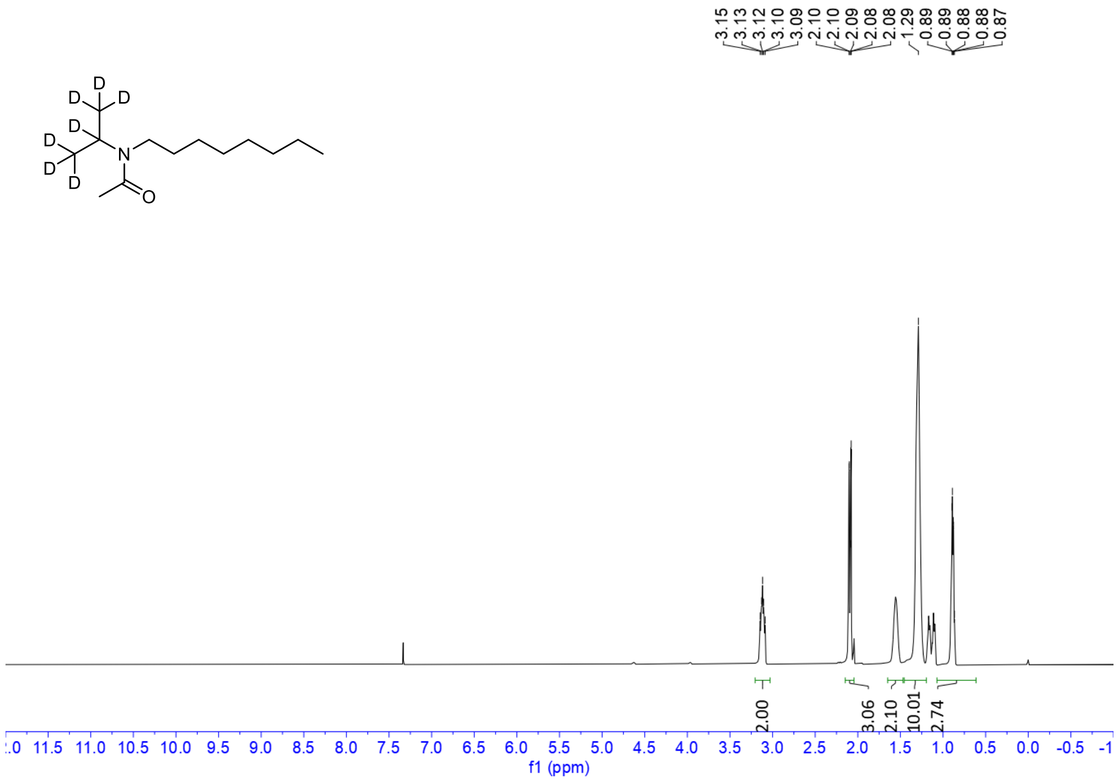

Supplementary Figure 161 | ^{13}C NMR (126 MHz, 298K, $CDCl_3$) of *N*-Octyl-*N*-(propan-2-yl-d_7)acetamide **(111a)**

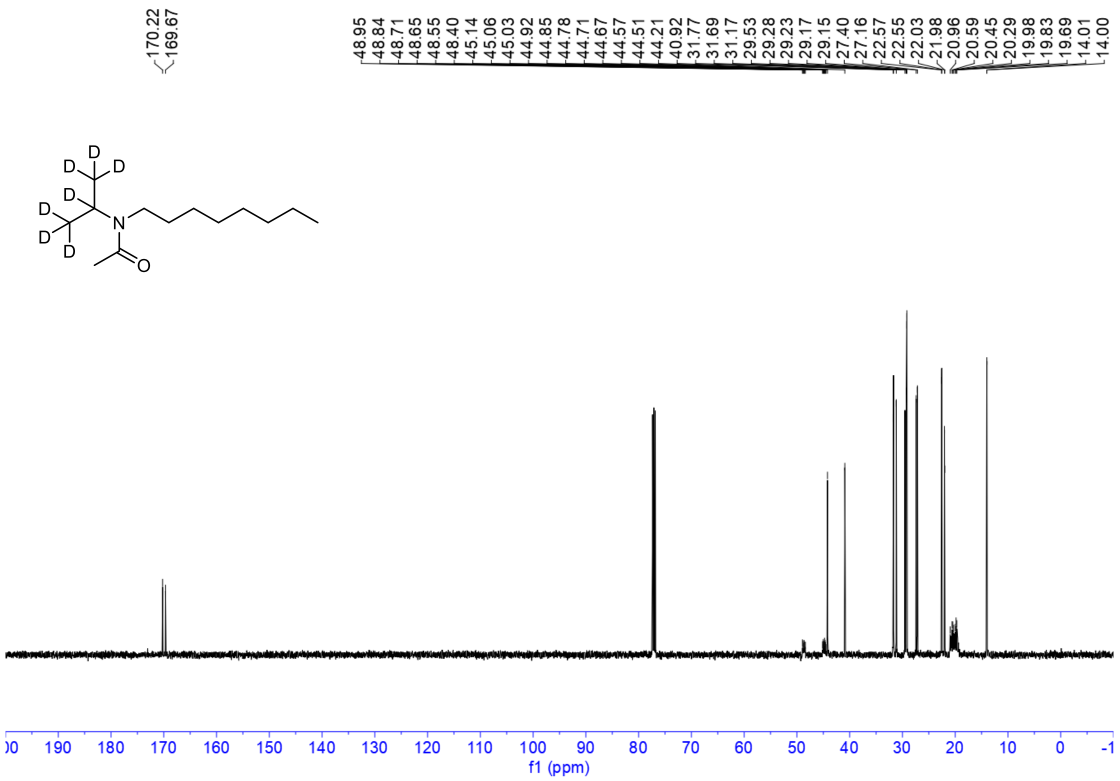

Supplementary Figure 162 | ^{1}H NMR (500 MHz, 298K, $CDCl_3$) of *N*-Ethyl-*N*-(propan-2-yl-d_7)octan-1-amine **(111b)**

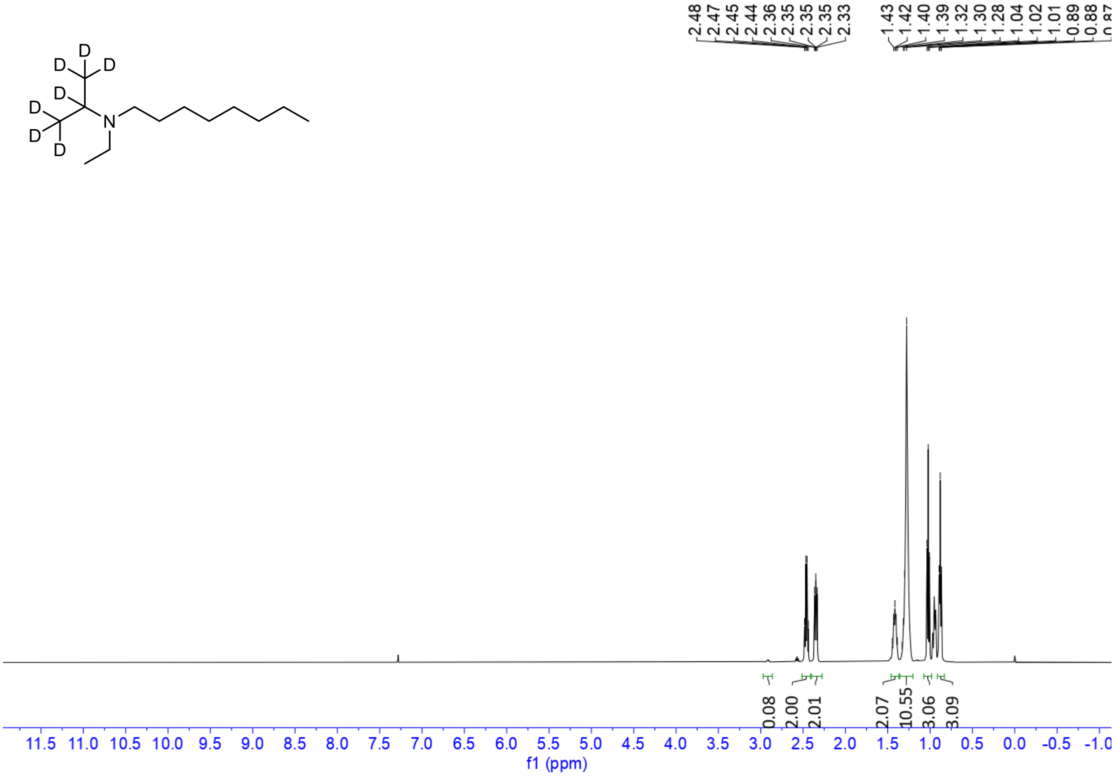

Supplementary Figure 163 | ^{13}C NMR (126 MHz, 298K, $CDCl_3$) of *N*-Ethyl-*N*-(propan-2-yl-d_7)octan-1-amine **(111b)**

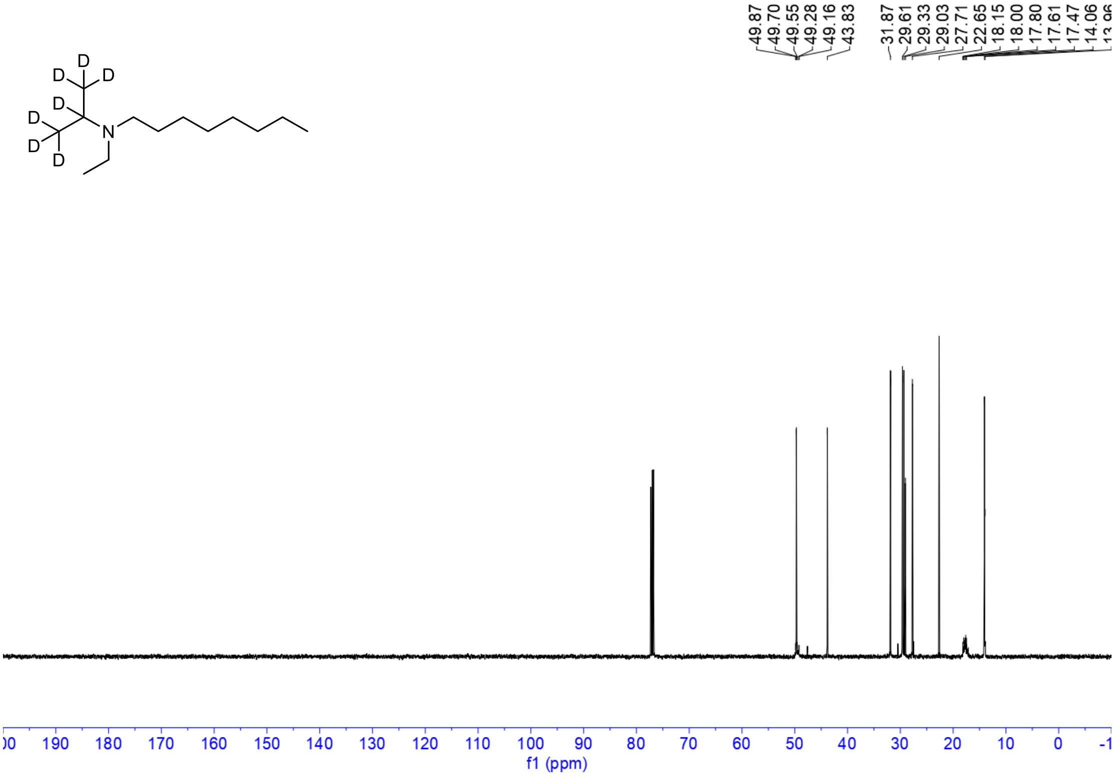

Supplementary Figure 164 | ^{1}H NMR (500 MHz, 298K, $CDCl_3$) of *N*,*N*-Diisopropylnonanamide **(112a)**

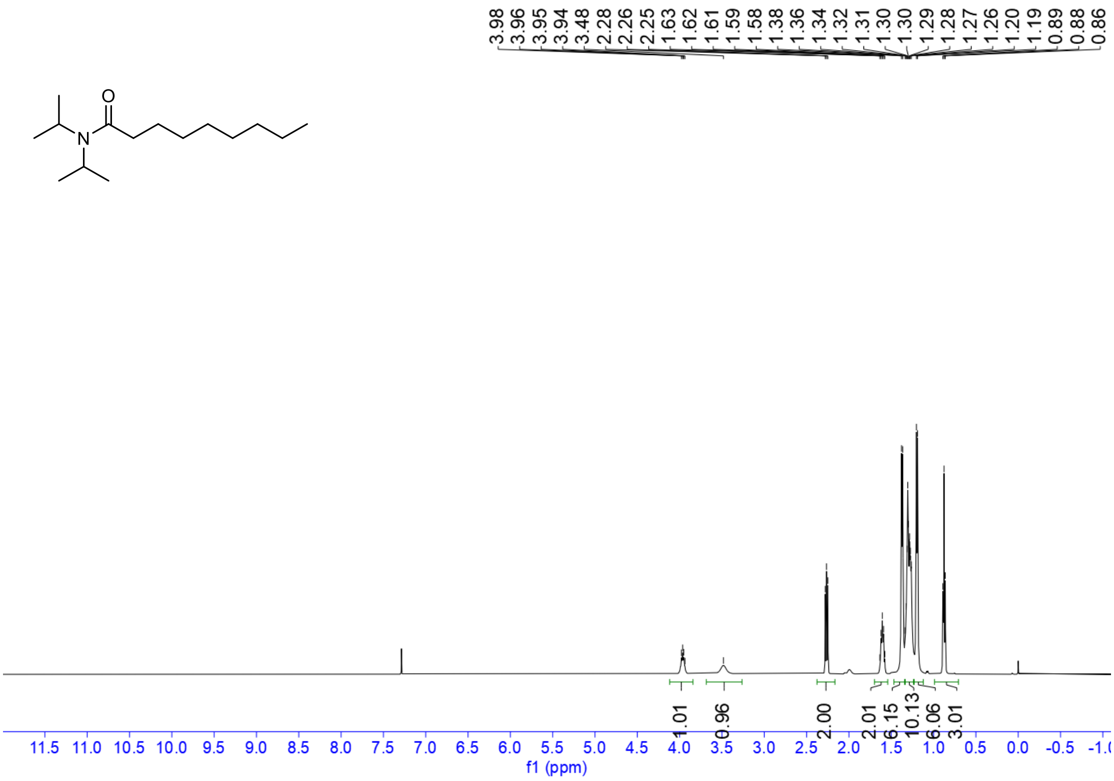

Supplementary Figure 165| ^{13}C NMR (126 MHz, 298K, $CDCl_3$) of *N*,*N*′-Diisopropylnon anamide **(112a)**

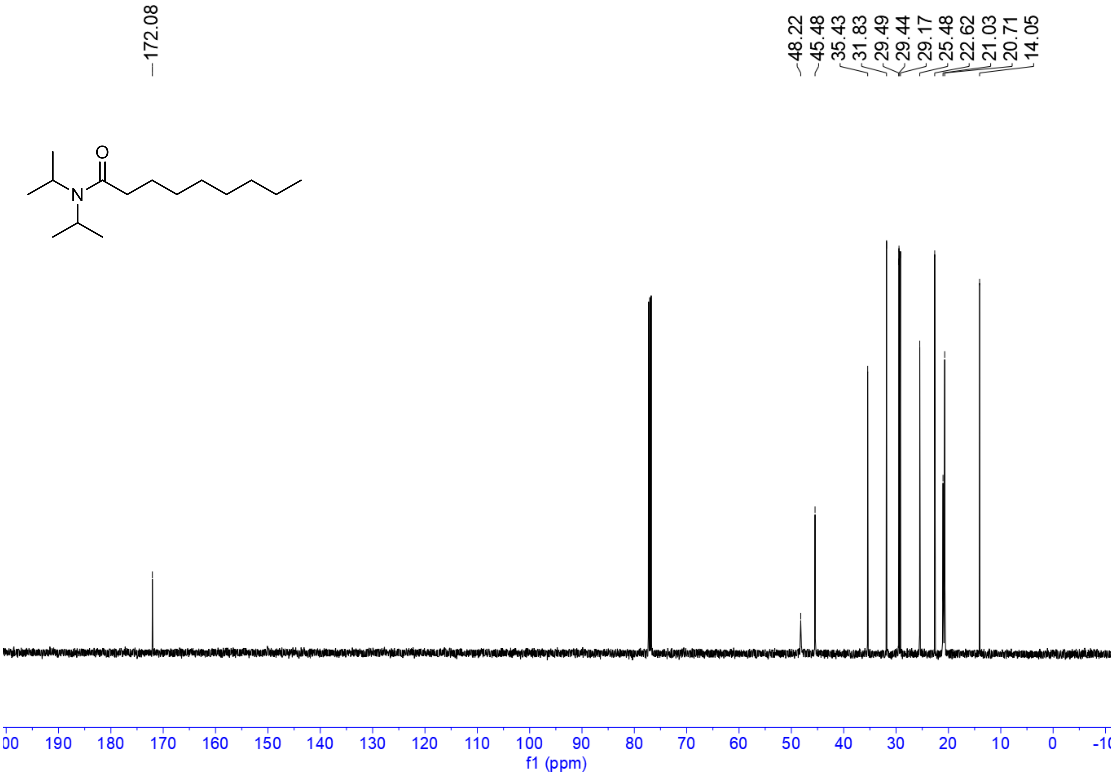

Supplementary Figure 166 | ^{1}H NMR (500 MHz, 298K, $CDCl_3$) of *N*,*N*′-Diisopropylnonanamide **(112b)**

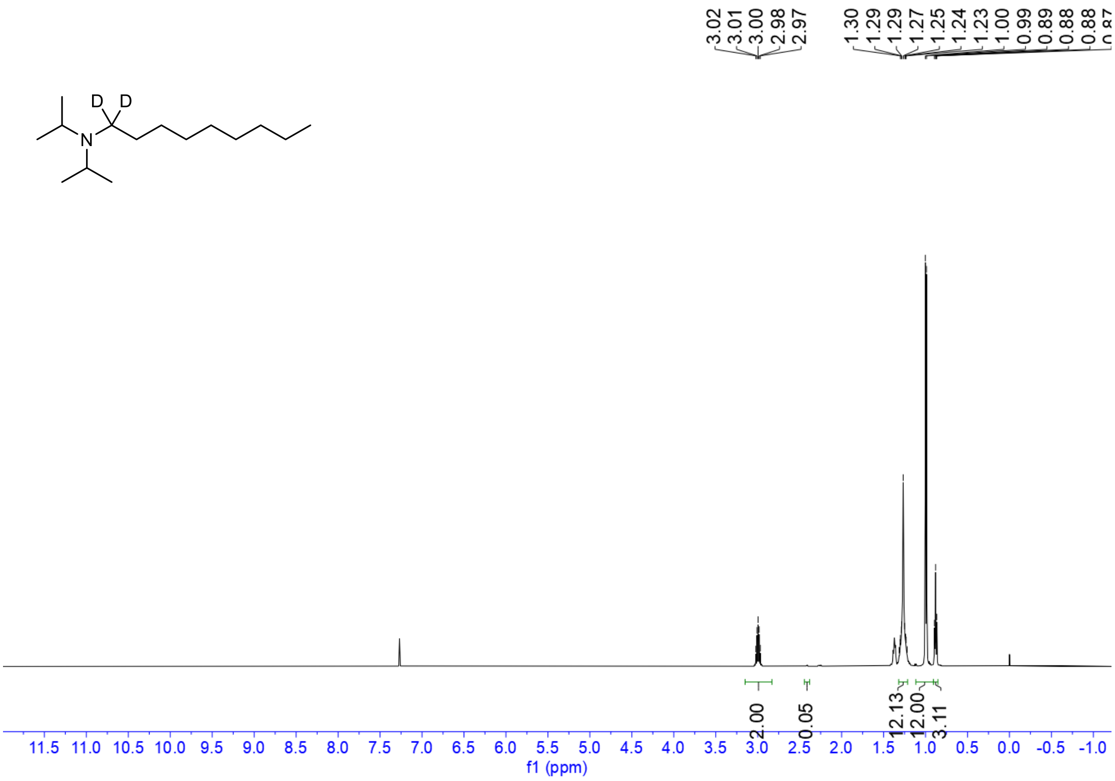

Supplementary Figure 167| ^{13}C NMR (126 MHz, 298K, $CDCl_3$) of *N*,*N*′-Diisopropylnon anamide **(112b)**

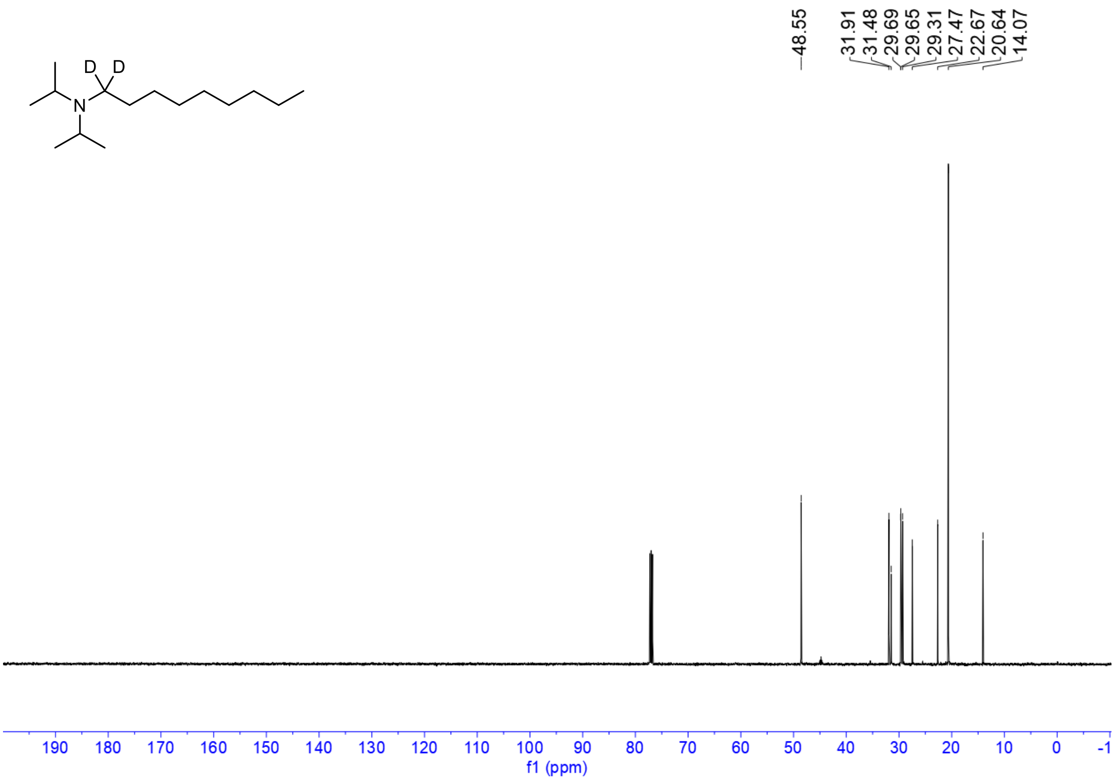

Supplementary Figure 168 | ^{1}H NMR (500 MHz, 298K, $CDCl_3$) of 1,10-Dioxa-5,6-dithiacyclohe ptadecane-2,9-dione **(109)**

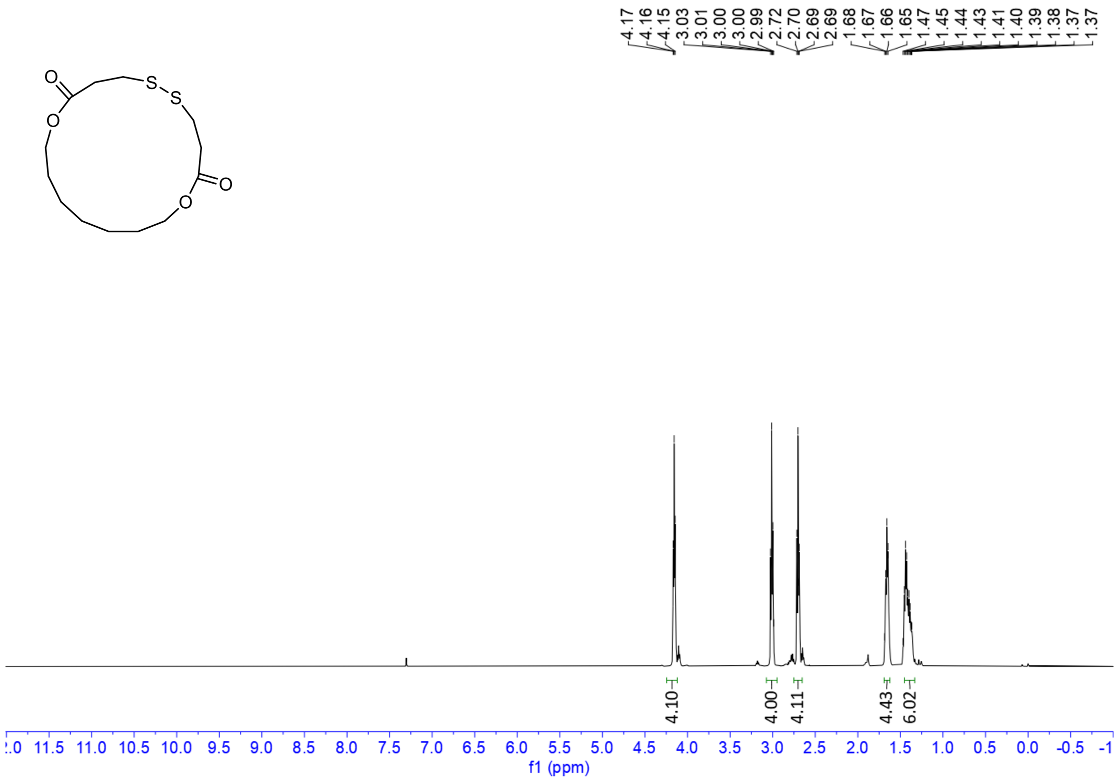

Supplementary Figure 169 | ^{13}C NMR (126 MHz, 298K, $CDCl_3$) of 1,10-Dioxa-5,6-dithiacycloheptadecane-2,9-dione **(109)**

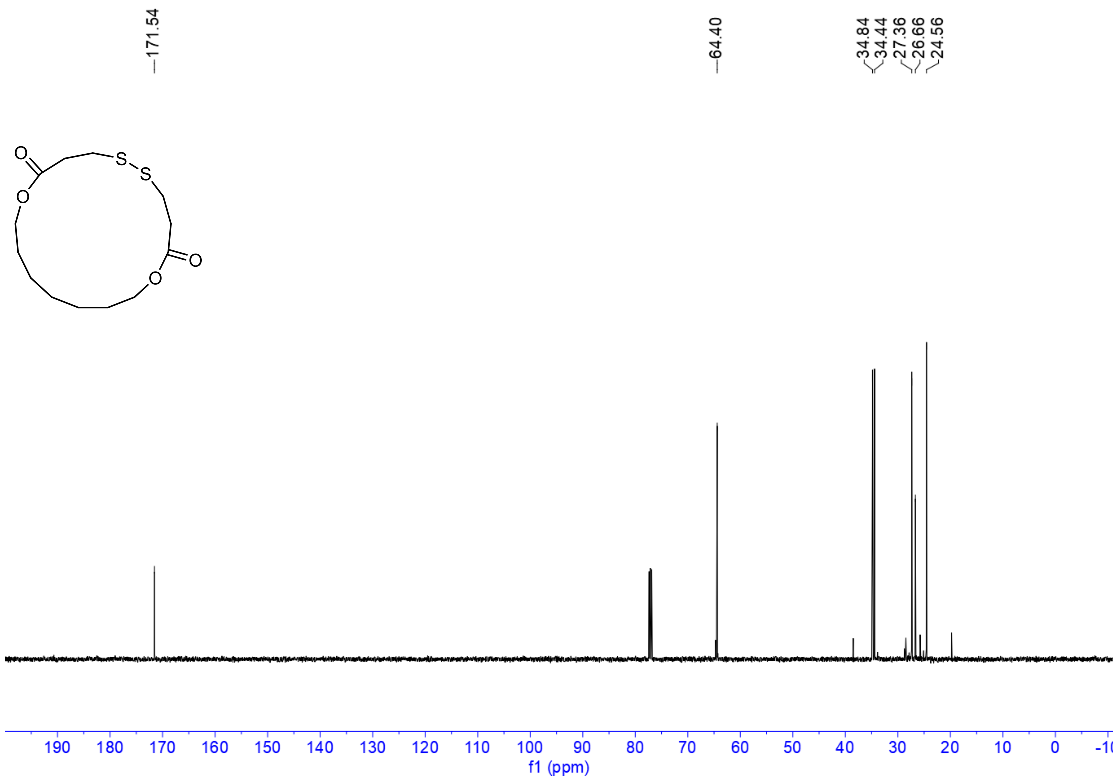

Supplementary Figure 170 | ^{1}H NMR (500 MHz, 298K, $CDCl_3$) of (1,4-Phenylenebis(oxy))bis(ethane-2,1-diyl) bis(2-(vinylthio)benzoate) **(52b)**

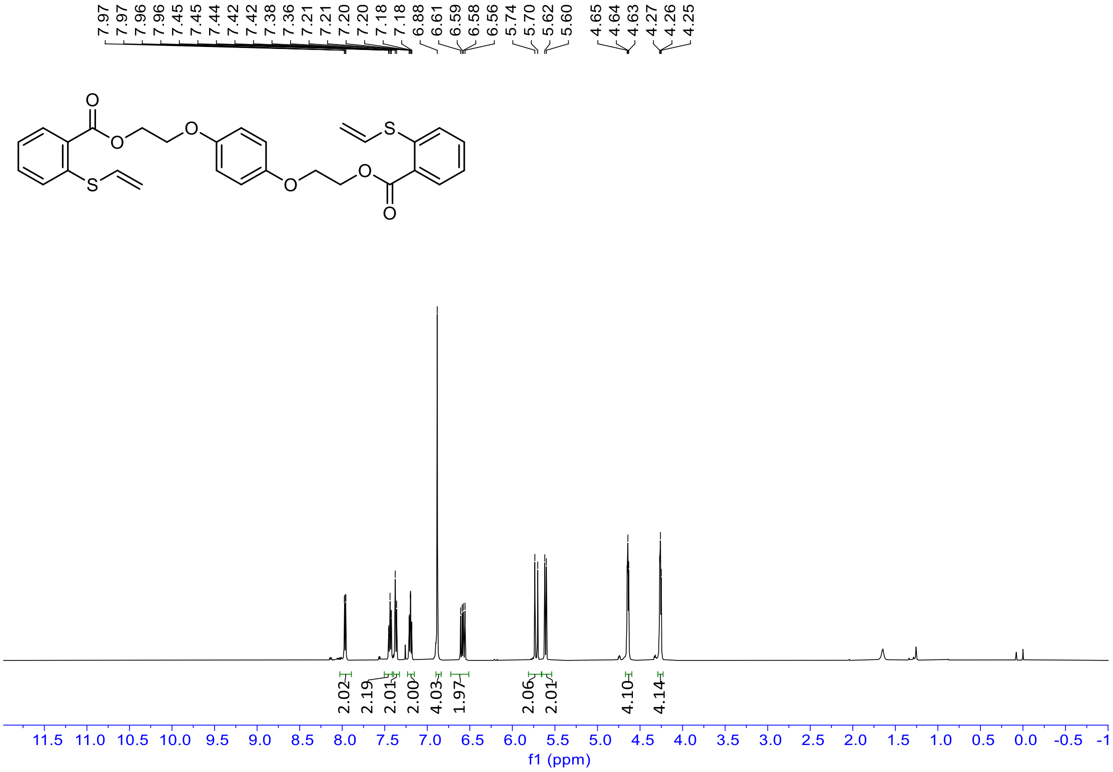

Supplementary Figure 171 | ^{13}C NMR (126 MHz, 298K, $CDCl_3$) of (1,4-Phenylenebis(oxy))bis(ethane-2,1-diyl) bis(2-(vinylthio)benzoate) **(52b)**

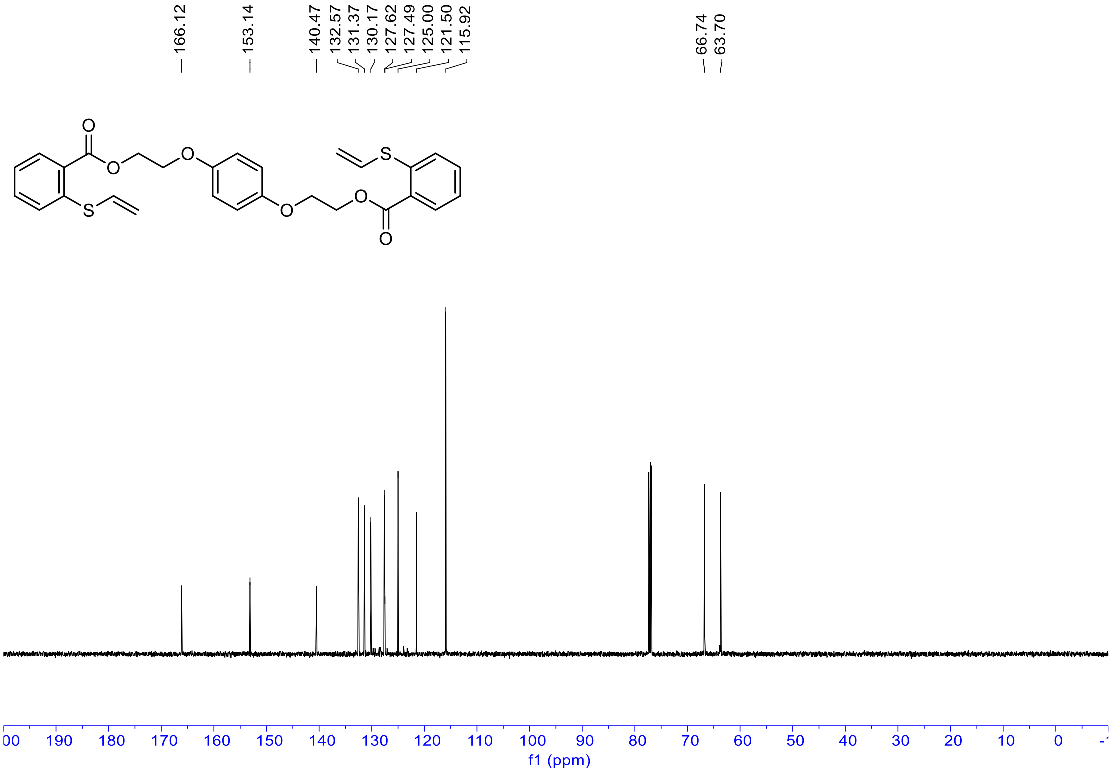

Supplementary Figure 172 | ^{1}H NMR (500 MHz, 298K, $CDCl_3$) of 1,10-Dioxa-4,7-dithiacyclotetradecane-2,9-dione **(4)**

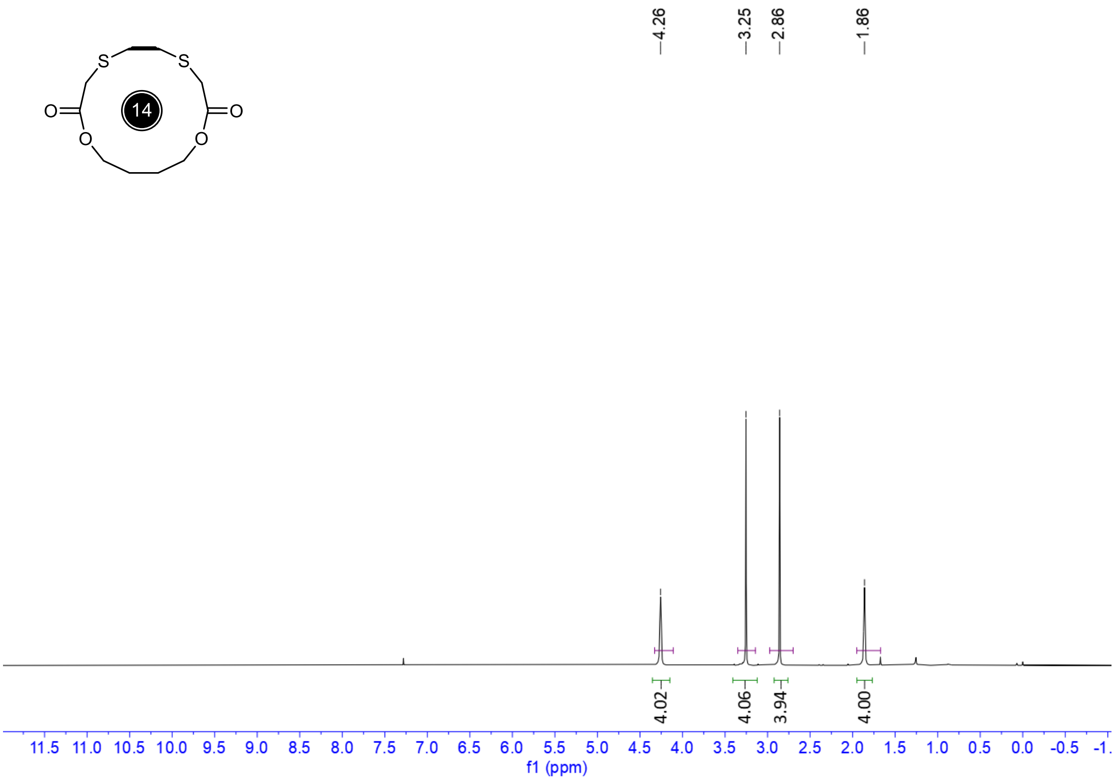

Supplementary Figure 173 | ^{13}C NMR (126 MHz, 298K, $CDCl_3$) of 1,10-Dioxa-4,7-dithiacyclotetradecane-2,9-dione **(4)**

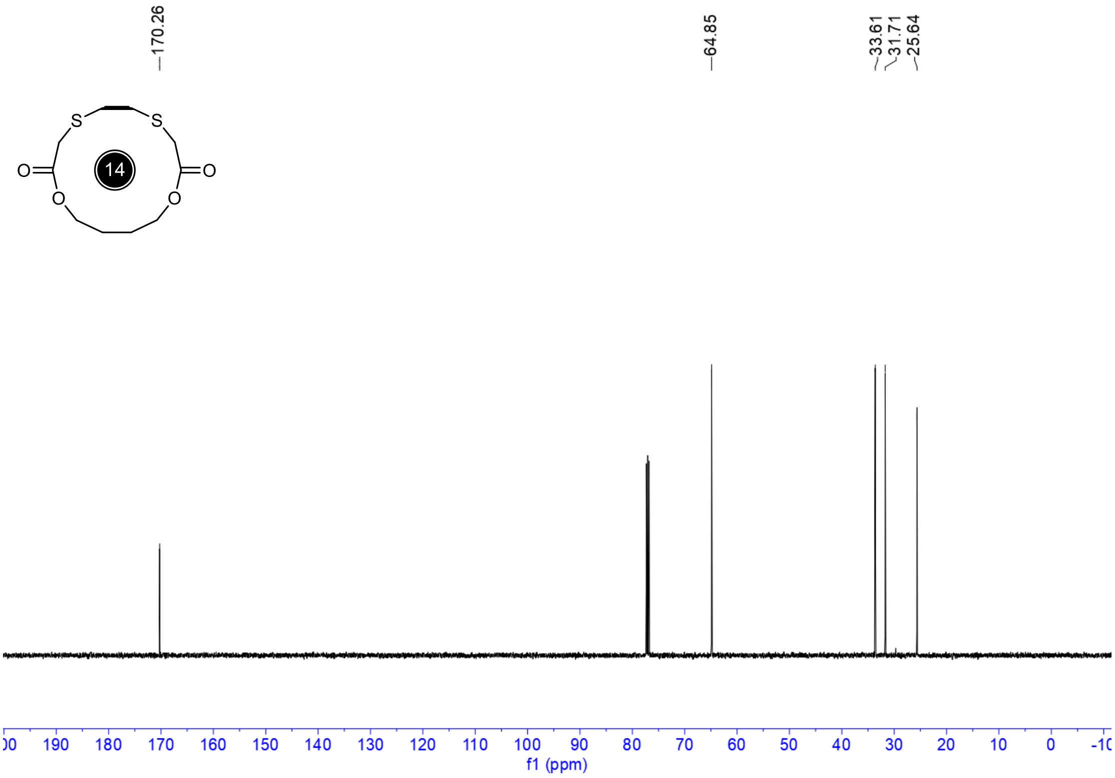

Supplementary Figure 174 | ^{1}H NMR (500 MHz, 298K, $CDCl_3$) of 1,4-Dioxa-8,11-dithiacyclotetradecane-5,14-dione **(5)**

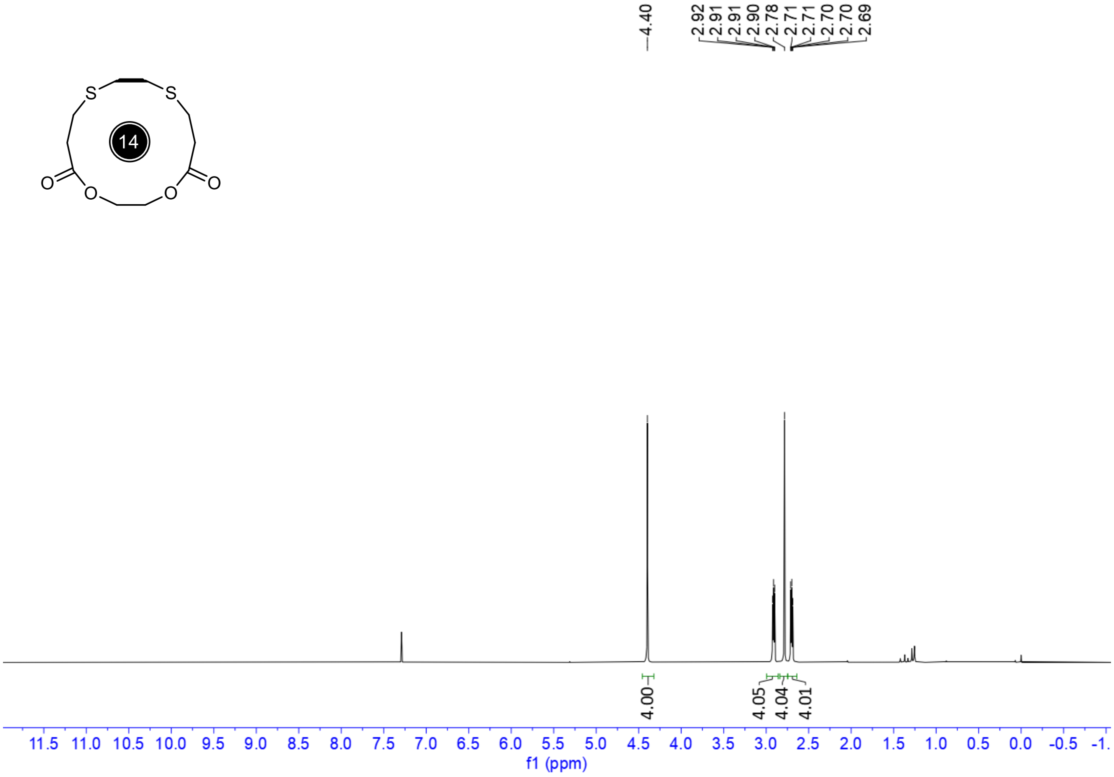

Supplementary Figure 175| ^{13}C NMR (126 MHz, 298K, $CDCl_3$) of 1,4-Dioxa-8,11-dithiacyclotetradecane-5,14-dione **(5)**

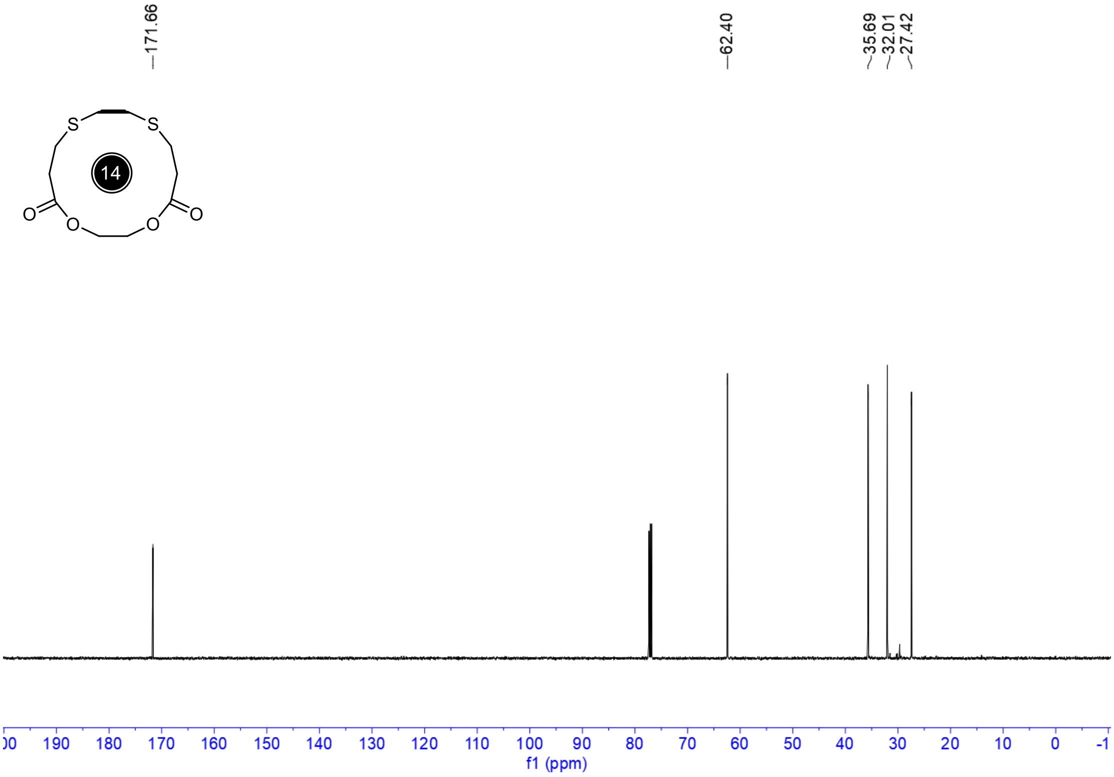

Supplementary Figure 176 | ^{1}H NMR (500 MHz, 298K, $CDCl_3$) of 1,5-Dioxa-9,12-dithiacyclopentadecane-6,15-dione **(6)**

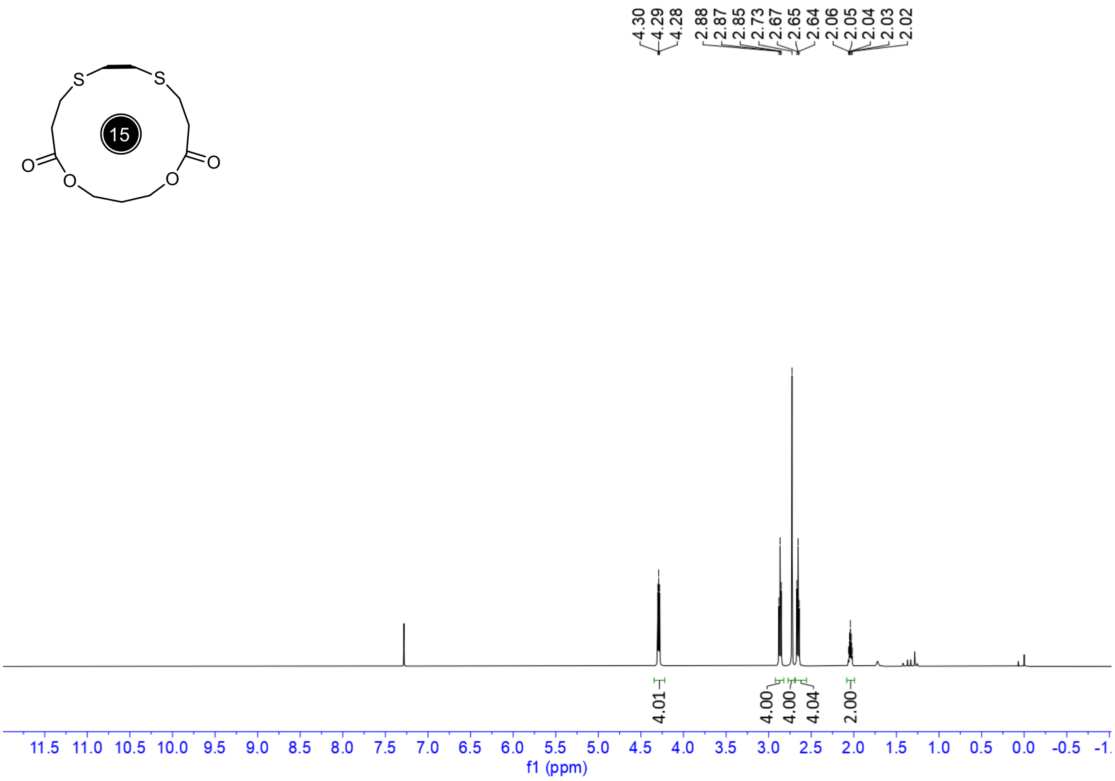

Supplementary Figure 177 | ^{13}C NMR (126 MHz, 298K, $CDCl_3$) of 1,5-Dioxa-9,12-dithiacyclopentadecane-6,15-dione **(6)**

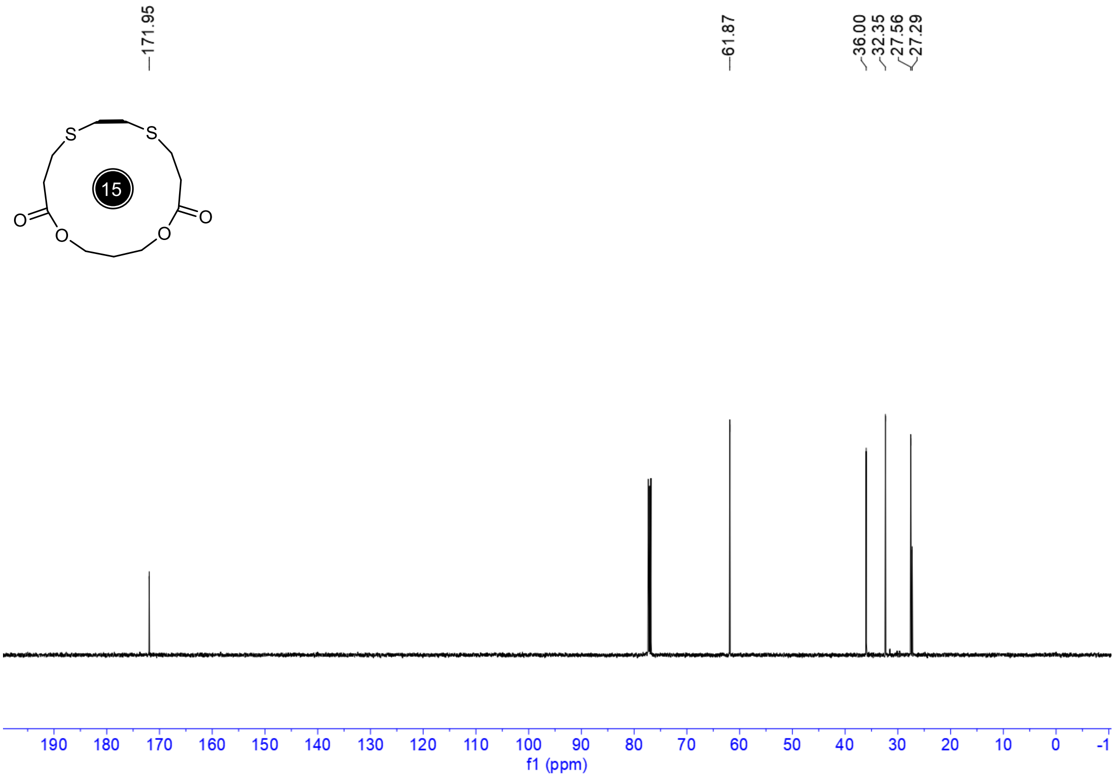

Supplementary Figure 178 | ^{1}H NMR (500 MHz, 298K, $CDCl_3$) of 3-Methyl-1,5-dioxa-9,12-dithiacyclopentadecane-6,15-dione **(7)**

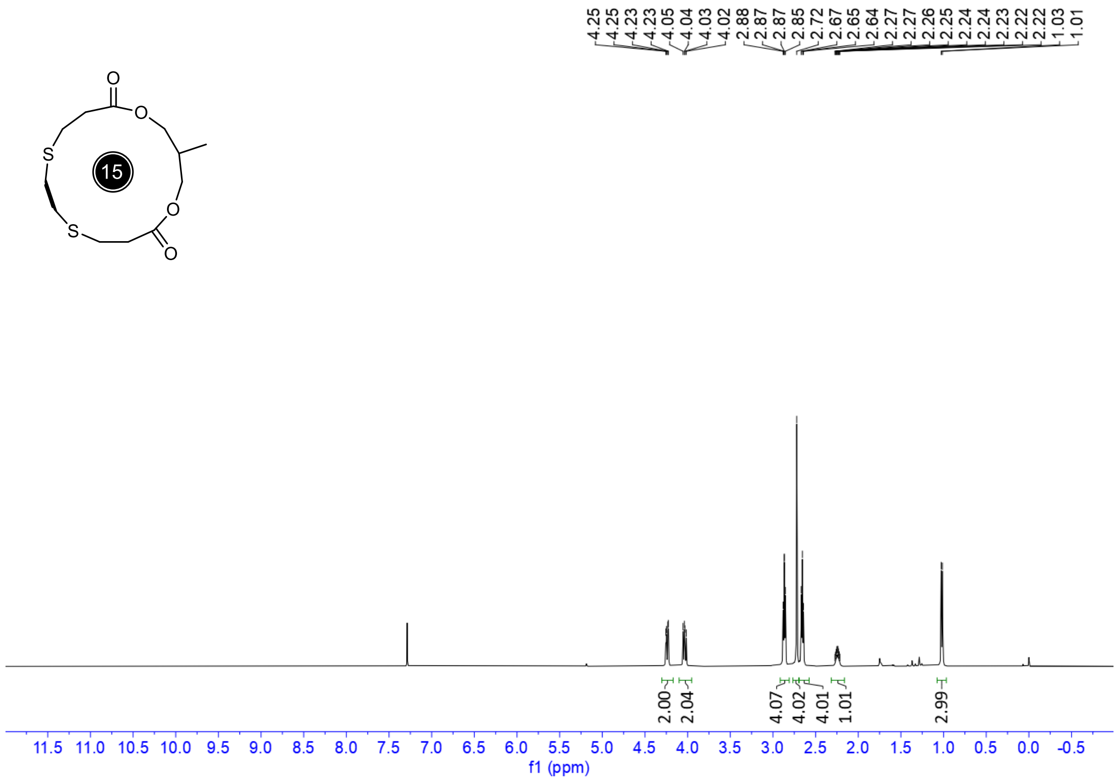

Supplementary Figure 179 | ^{13}C NMR (126 MHz, 298K, $CDCl_3$) of 3-Methyl-1,5-dioxa-9,12-dithiacyclopentadecane-6,15-dione **(7)**

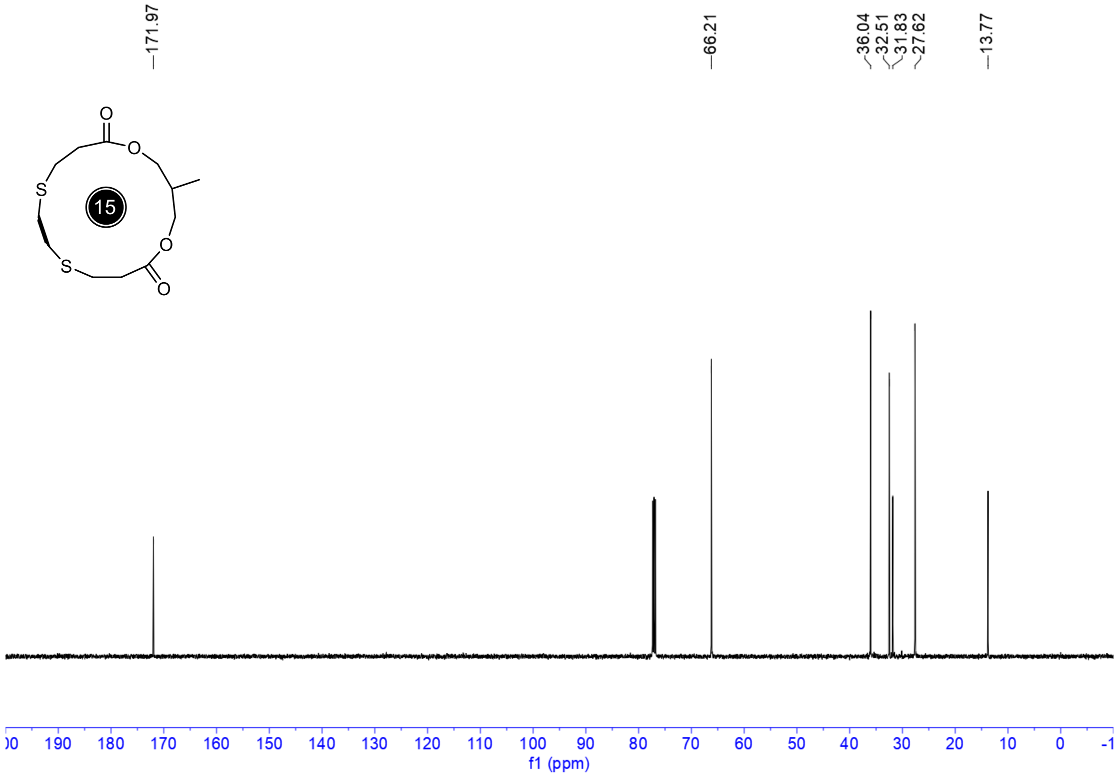

Supplementary Figure 180 | ^{1}H NMR (500 MHz, 298K, $CDCl_3$) of (±)-2,4-Dimethyl-1,5-dioxa-9,12-dithiacyclopentadecane-6,15-dione **(8)**

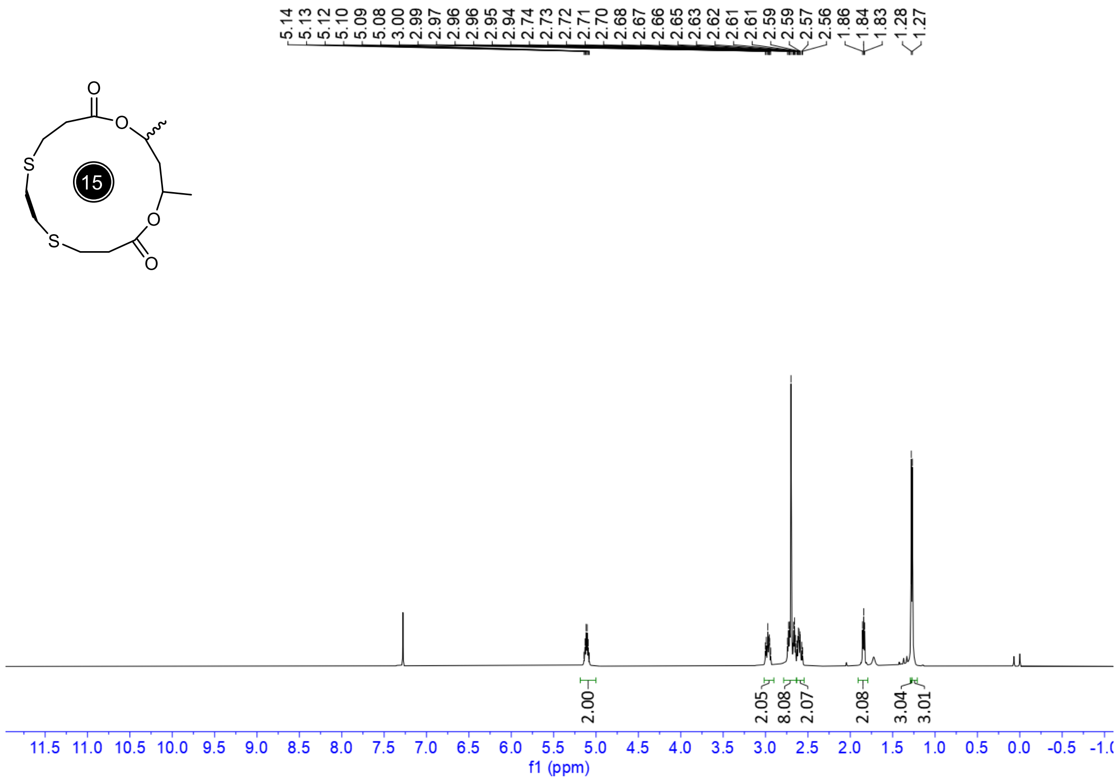

Supplementary Figure 181 | ^{13}C NMR (126 MHz, 298K, $CDCl_3$) of (±)-2,4-Dimethyl-1,5-dioxa-9,12-dithiacyclopentadecane-6,15-dione **(8)**

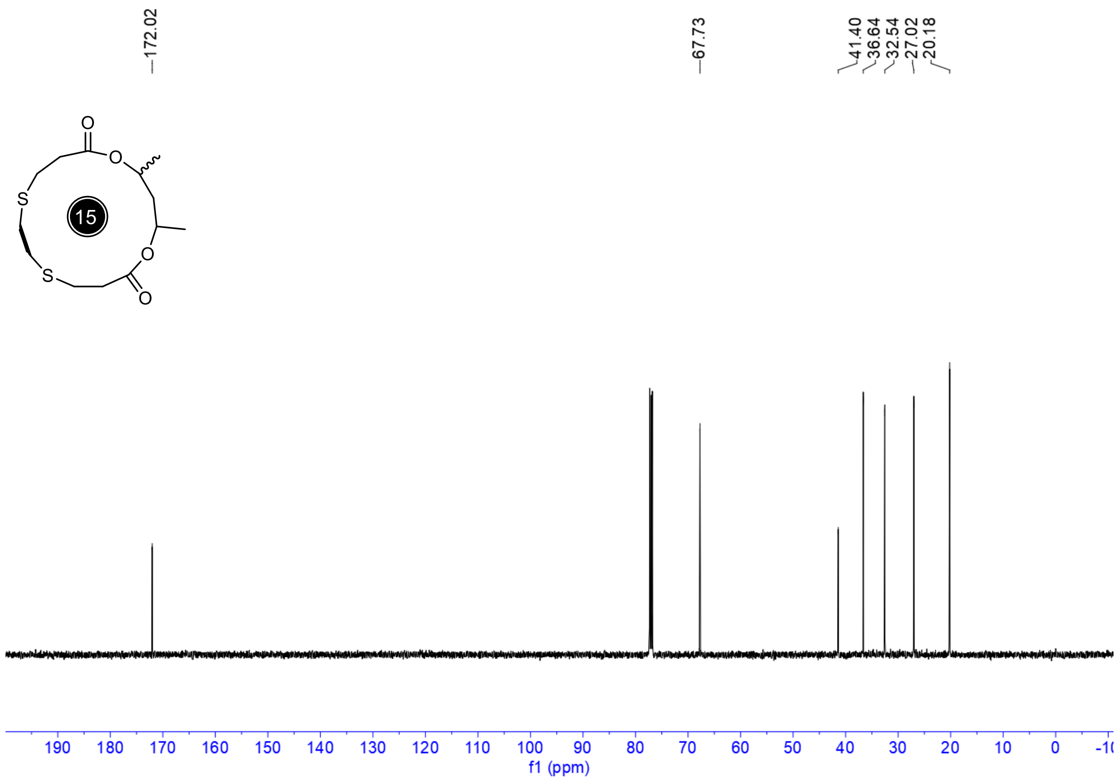

Supplementary Figure 182 | ^{1}H NMR (500 MHz, 298K, $CDCl_3$) of 3,3-Dimethyl-1,5-dioxa-9,12-dithiacyclopentadecane-6,15-dione **(9)**

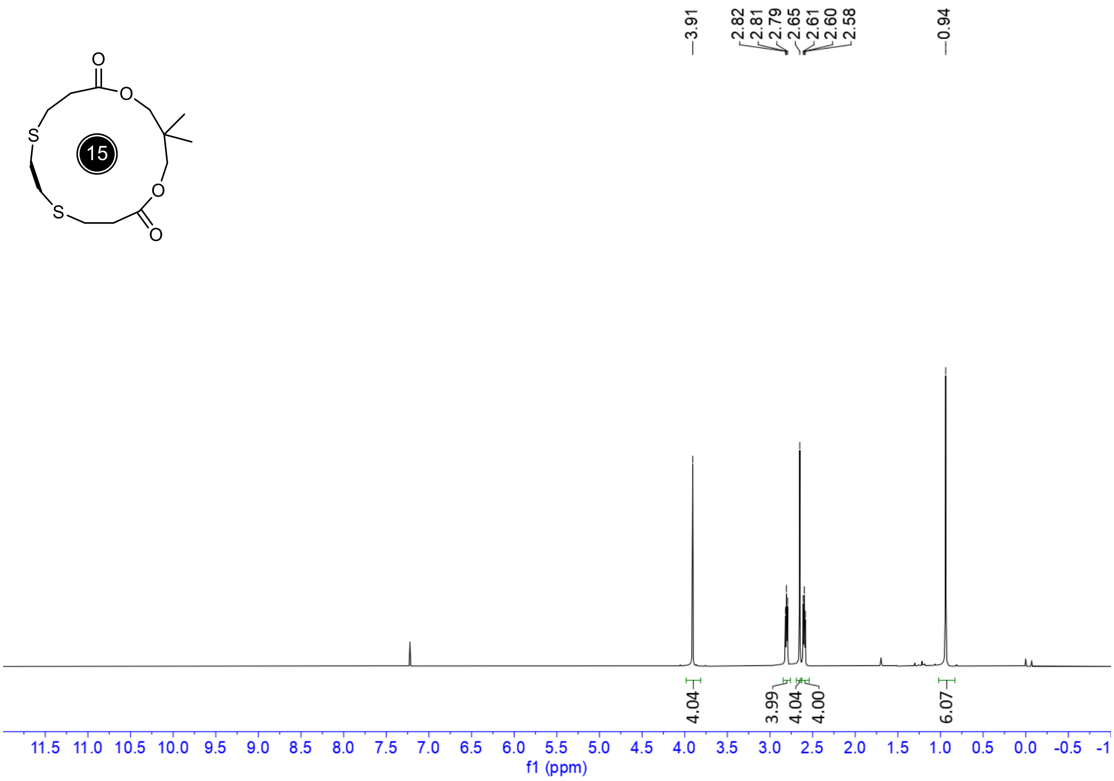

Supplementary Figure 183 | ^{13}C NMR (126 MHz, 298K, $CDCl_3$) of 3,3-Dimethyl-1,5-dioxa-9,12-dithiacyclopentadecane-6,15-dione **(9)**

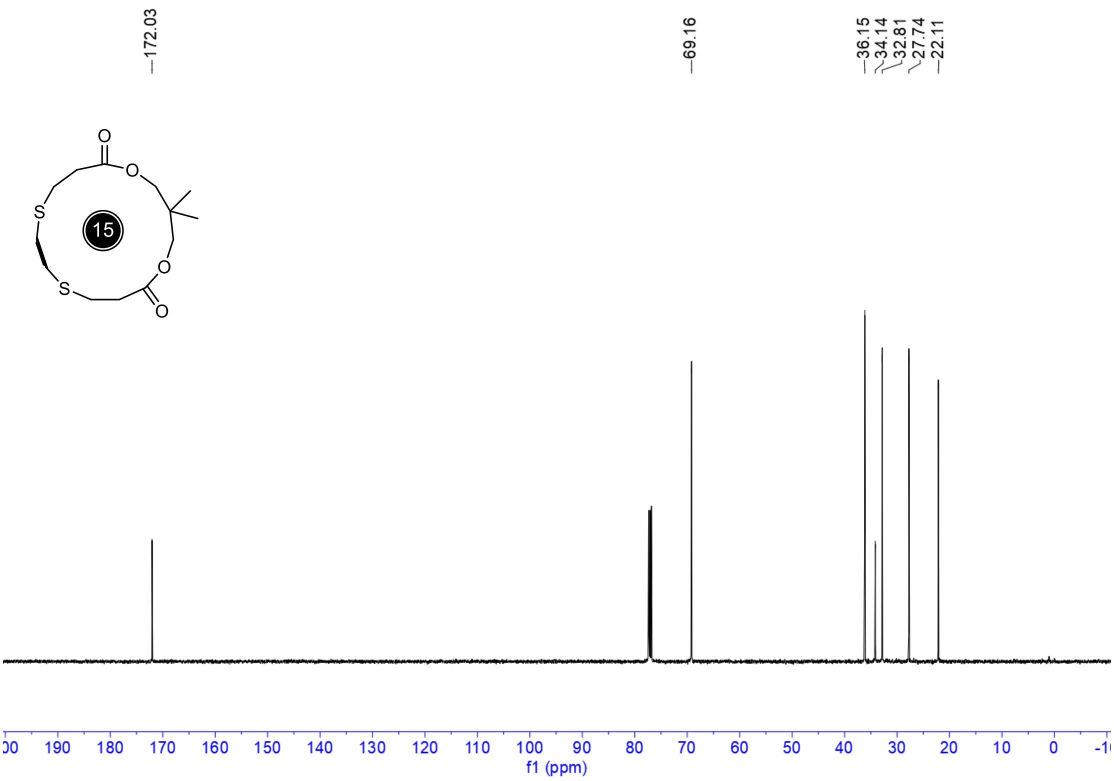

Supplementary Figure 184 | ^{1}H NMR (500 MHz, 298K, $CDCl_3$) of 1,12-Dioxa-5,8-dithiacyclohexadecane-2,11-dione **(10)**

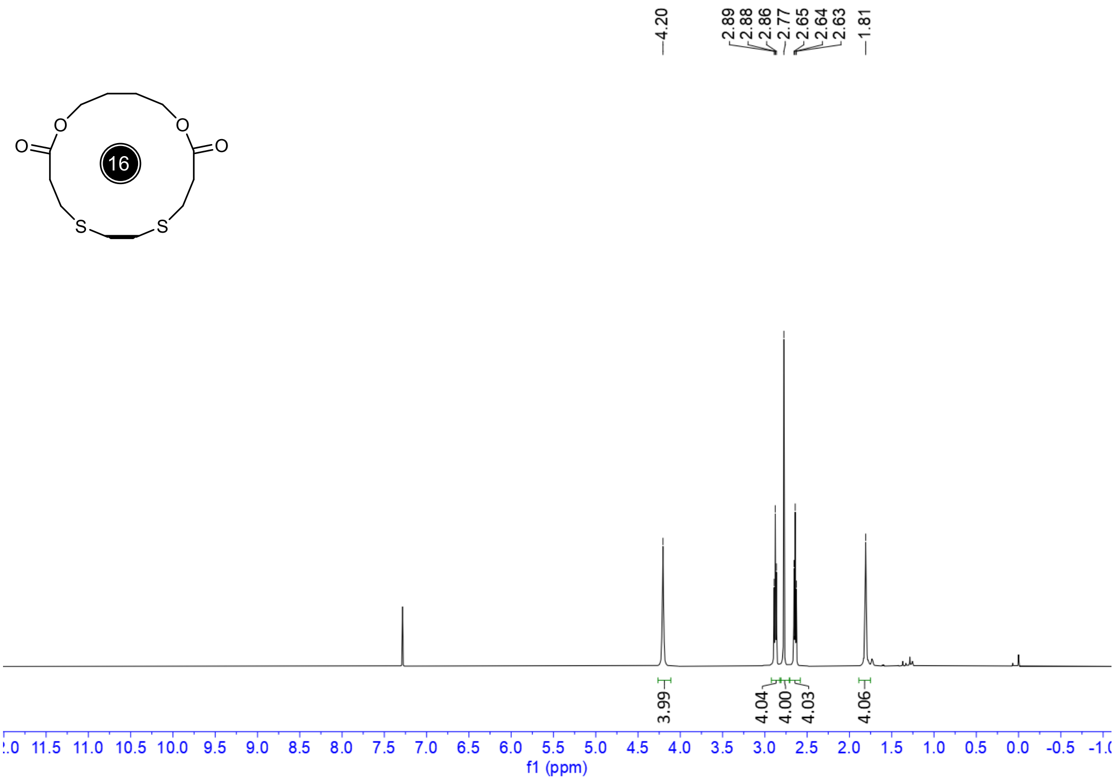

Supplementary Figure 185 | ^{13}C NMR (126 MHz, 298K, $CDCl_3$) of 1,12-Dioxa-5,8-dithiacyclohexadecane-2,11-dione **(10)**

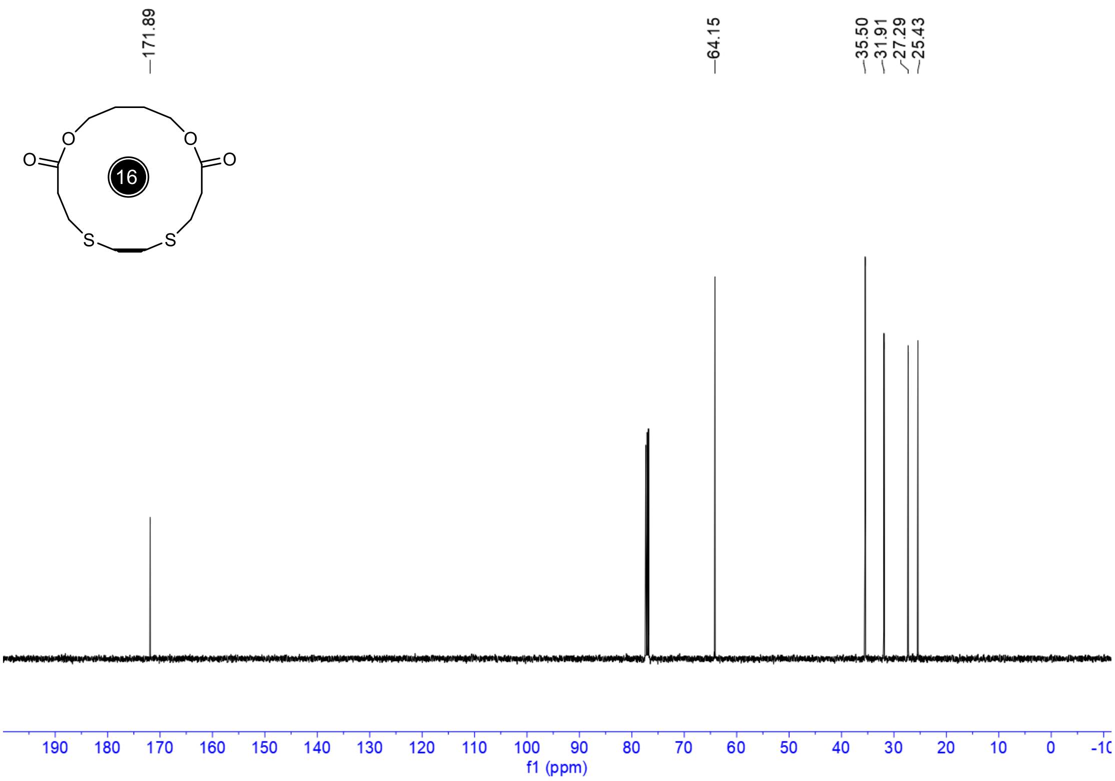

Supplementary Figure 186 | ^{1}H NMR (500 MHz, 298K, $CDCl_3$) of 1,12-Dioxa-5,8-dithiacycloheptadecane-2,11-dione **(11)**

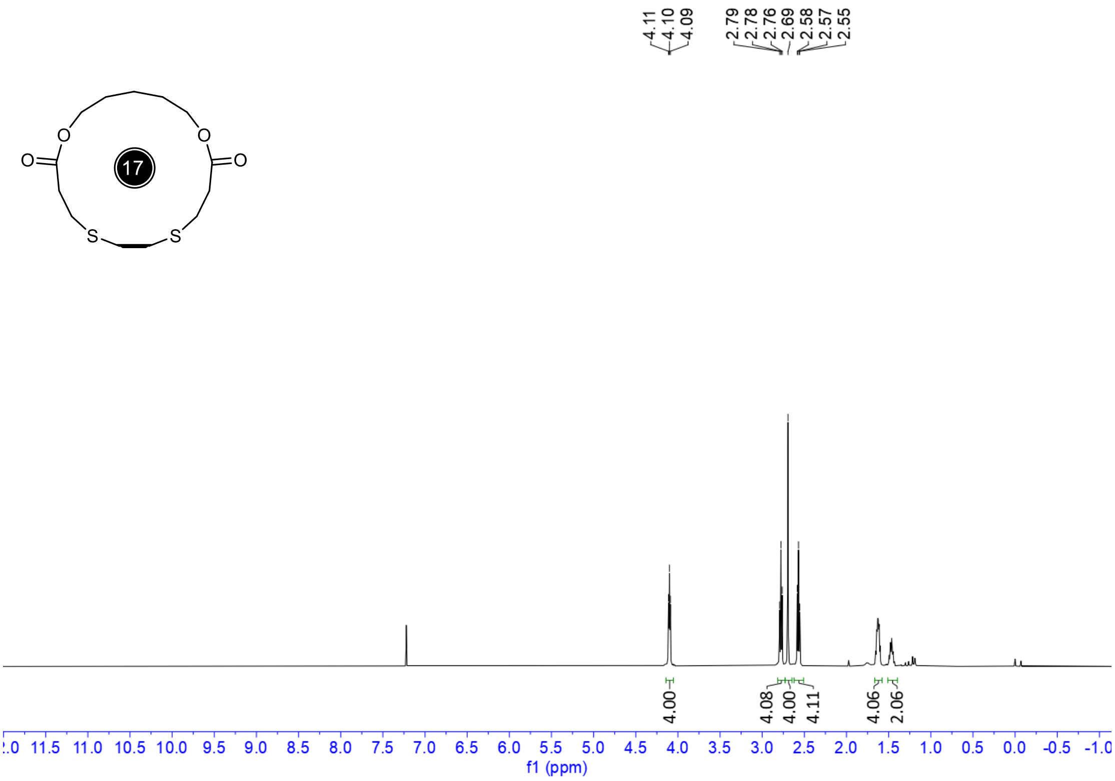

Supplementary Figure 187 | ^{13}C NMR (126 MHz, 298K, $CDCl_3$) of 1,12-Dioxa-5,8-dithiacycloheptadecane-2,11-dione **(11)**

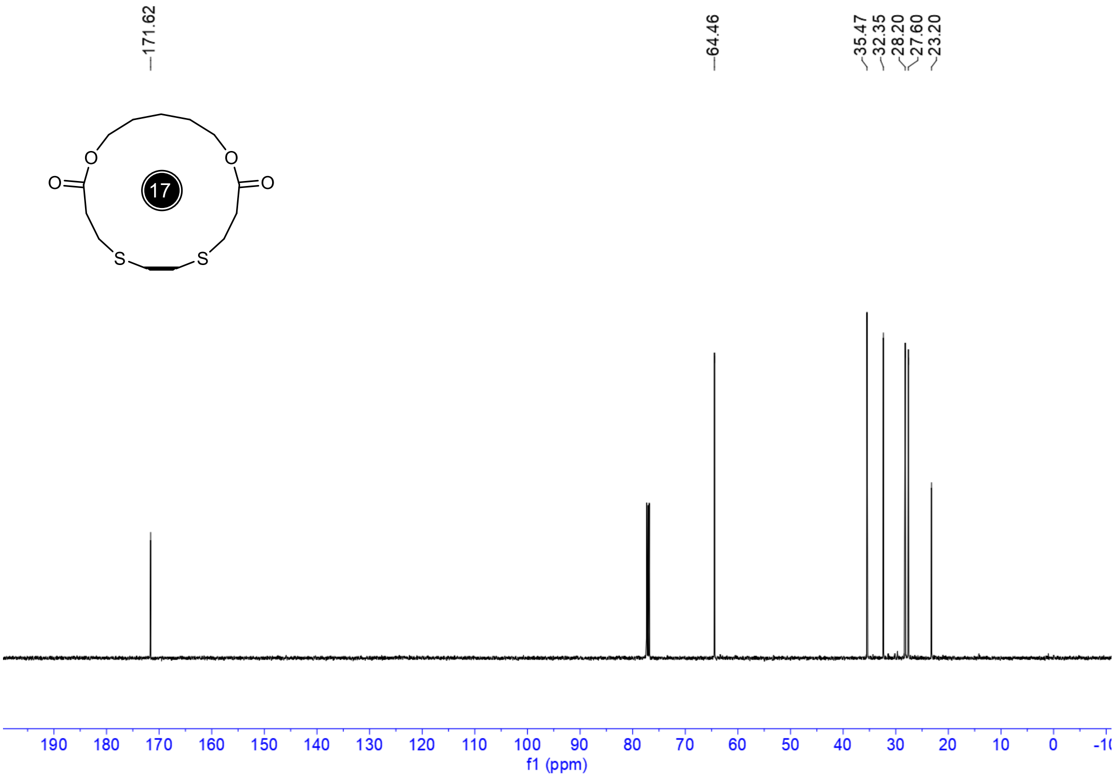

Supplementary Figure 188 | ^{1}H NMR (500 MHz, 298K, $CDCl_3$) of 1,12-Dioxa-5,8-dithiacyclooctadecane-2,11-dione **(12)**

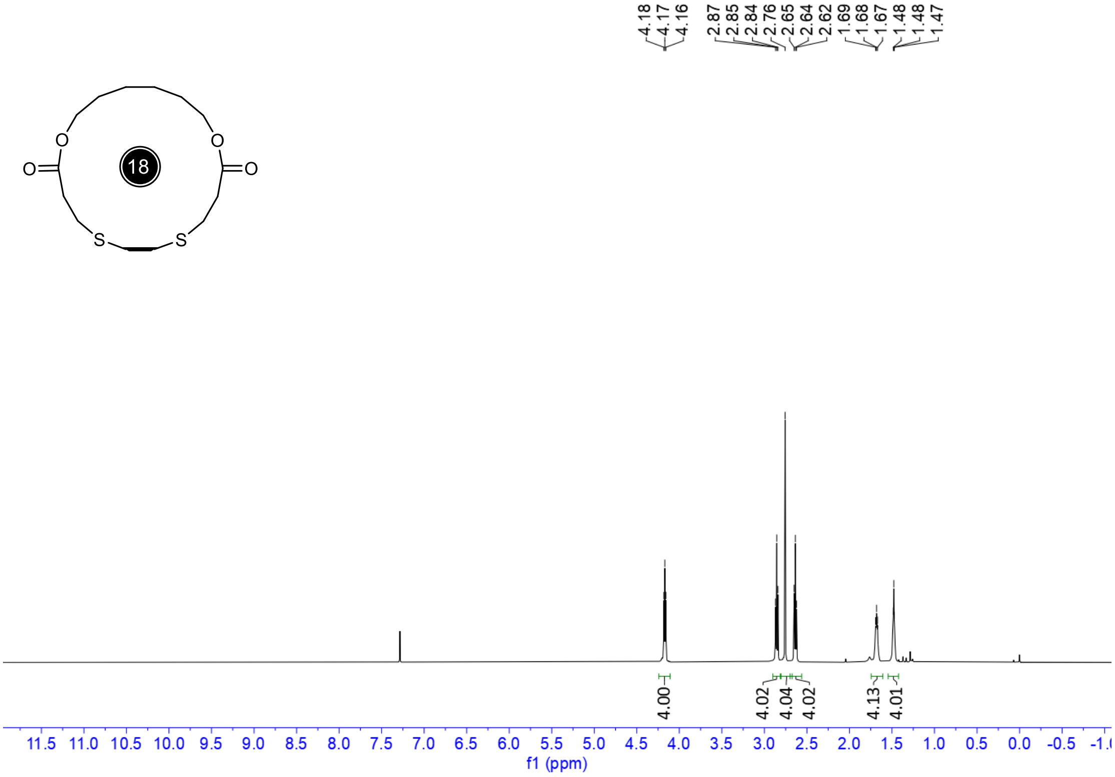

Supplementary Figure 189| ^{13}C NMR (126 MHz, 298K, $CDCl_3$) of 1,12-Dioxa-5,8-dithiacycloheptadecane-2,11-dione **(12)**

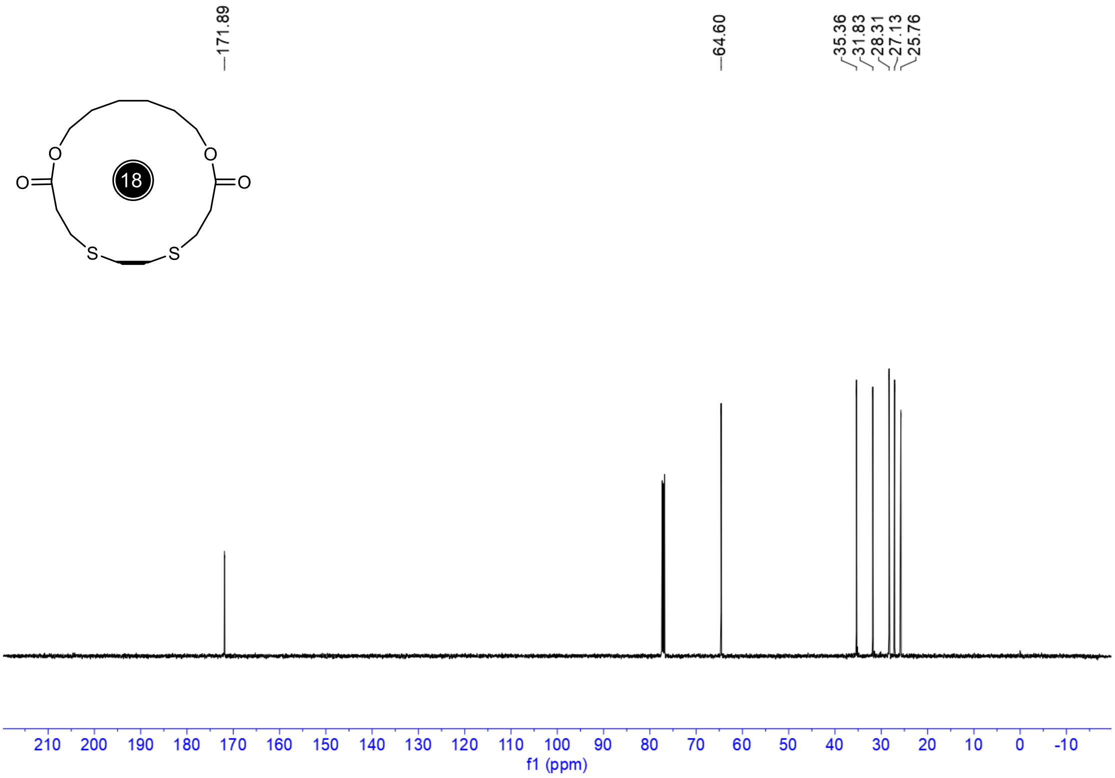

Supplementary Figure 190 | ^{1}H NMR (500 MHz, 298K, $CDCl_3$) of 1,10-Dioxa-4,7-dithiacyclooctadecane-11,18-dione **(13)**

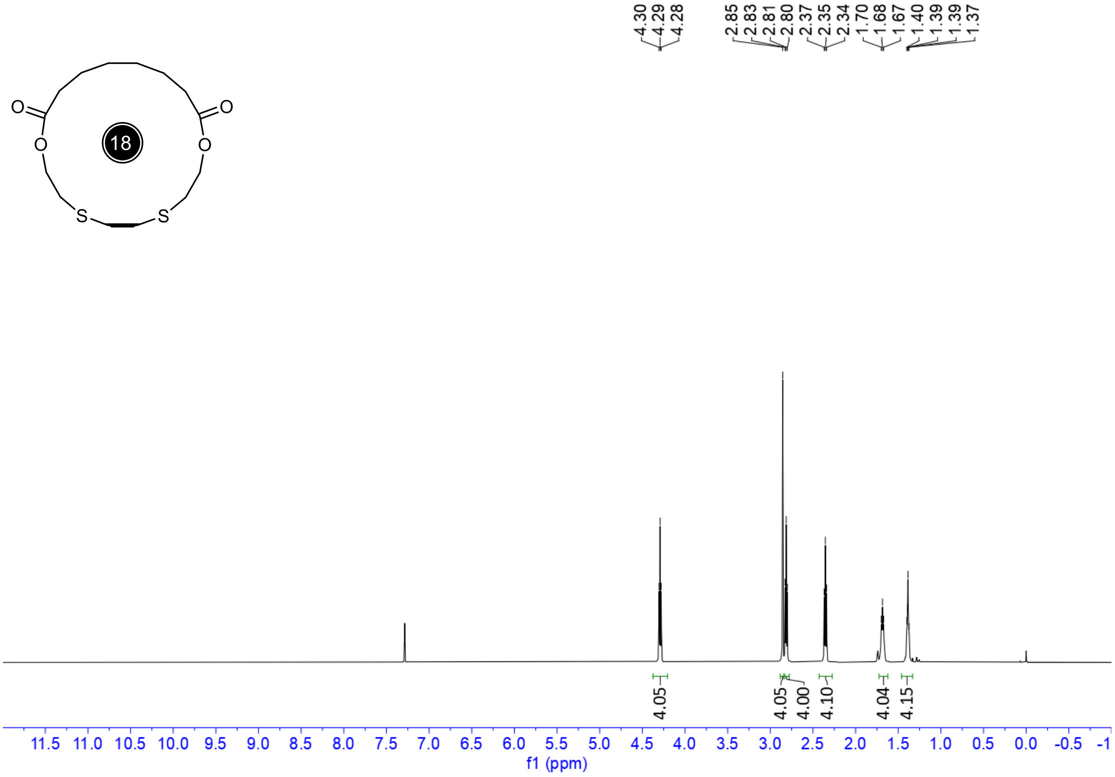

Supplementary Figure 191 | ^{13}C NMR (126 MHz, 298K, $CDCl_3$) of 1,10-Dioxa-4,7-dithiacyclooctadecane-11,18-dione **(13)**

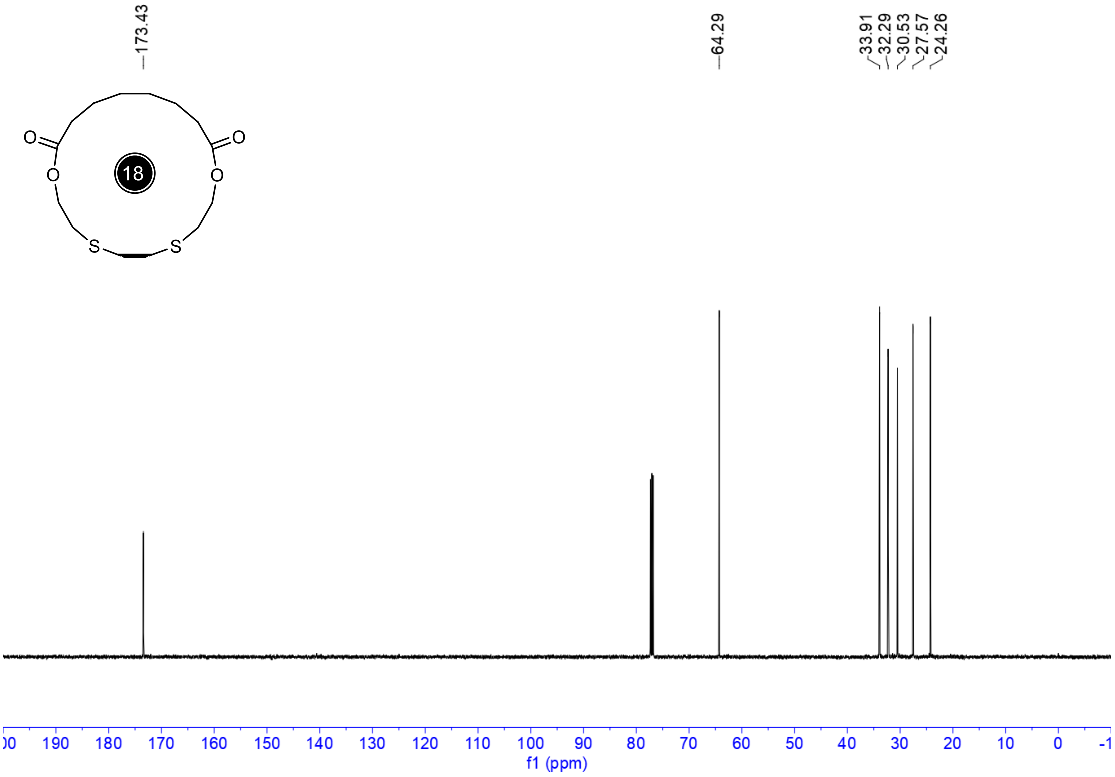

Supplementary Figure 192 | ^{1}H NMR (500 MHz, 298K, $CDCl_3$) of 1,12-Dioxa-5,8-dithiacyclononadecane-2,11-dione **(14)**

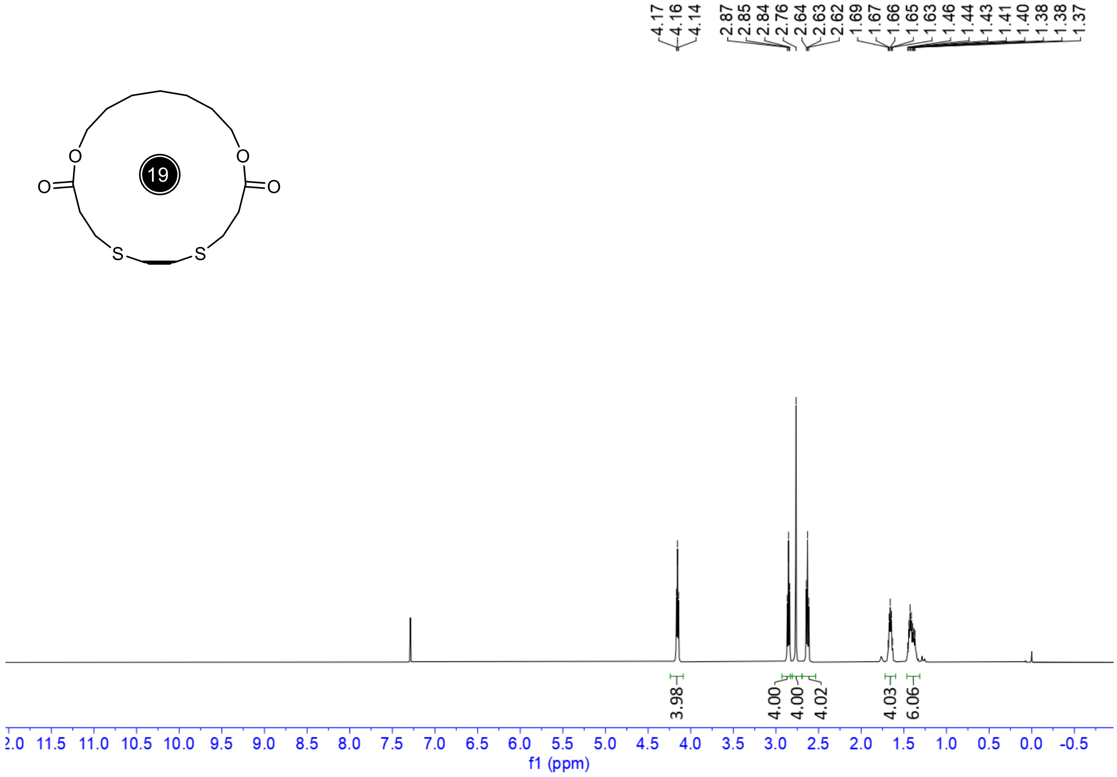

Supplementary Figure 193 | ^{13}C NMR (126 MHz, 298K, $CDCl_3$) of 1,12-Dioxa-5,8-dithiacyclononadecane-2,11-dione **(14)**

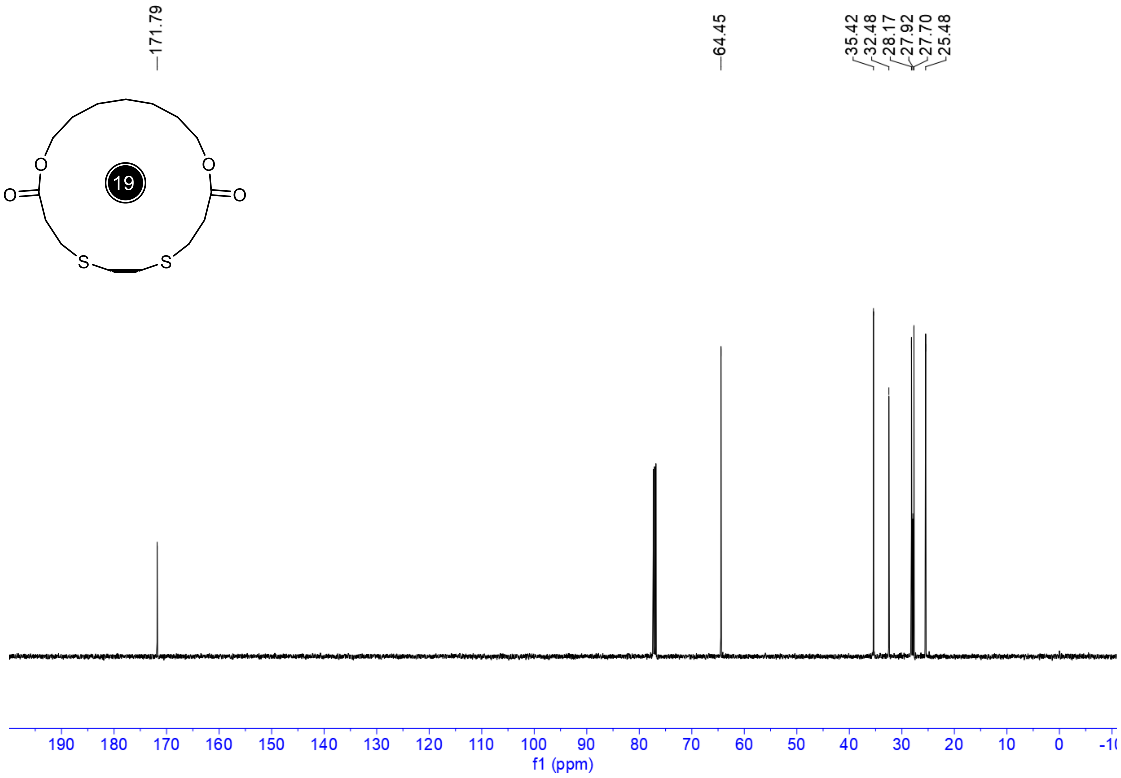

Supplementary Figure 194 | ^{1}H NMR (500 MHz, 298K, $CDCl_3$) of 1,12-Dioxa-5,8-dithiacycloicosane-2,11-dione **(15)**

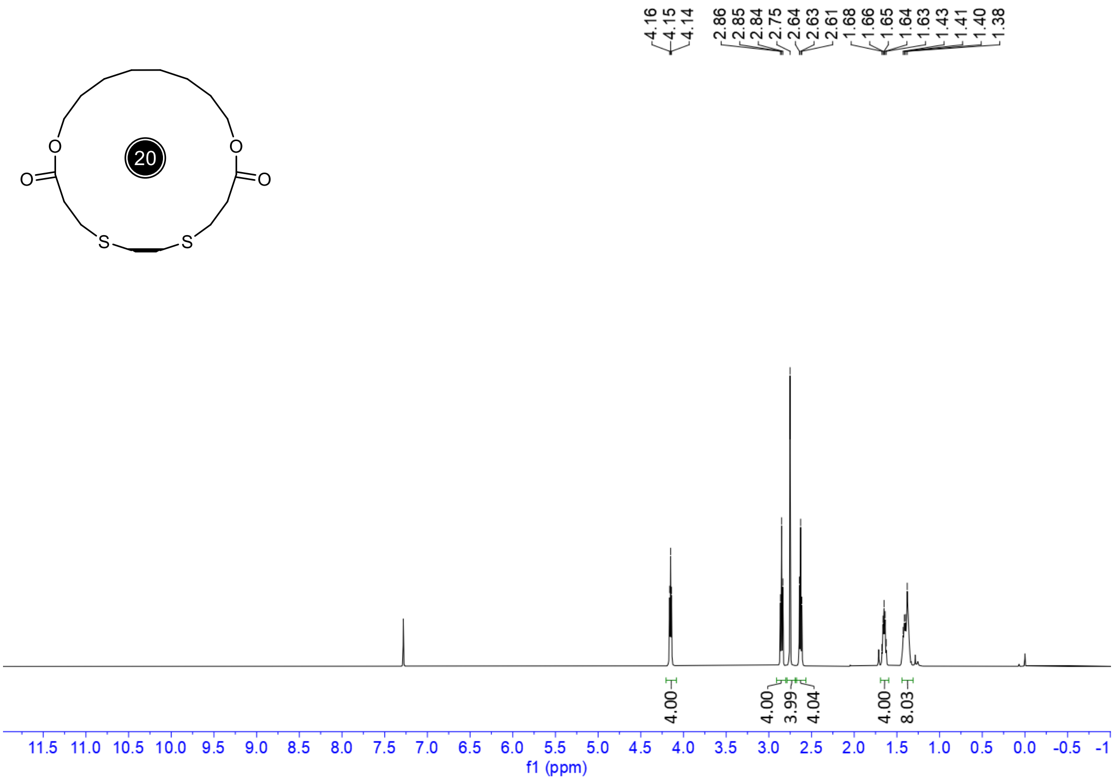

Supplementary Figure 195 | ^{13}C NMR (126 MHz, 298K, $CDCl_3$) of 1,12-Dioxa-5,8-dithiacycloicosane-2,11-dione **(15)**

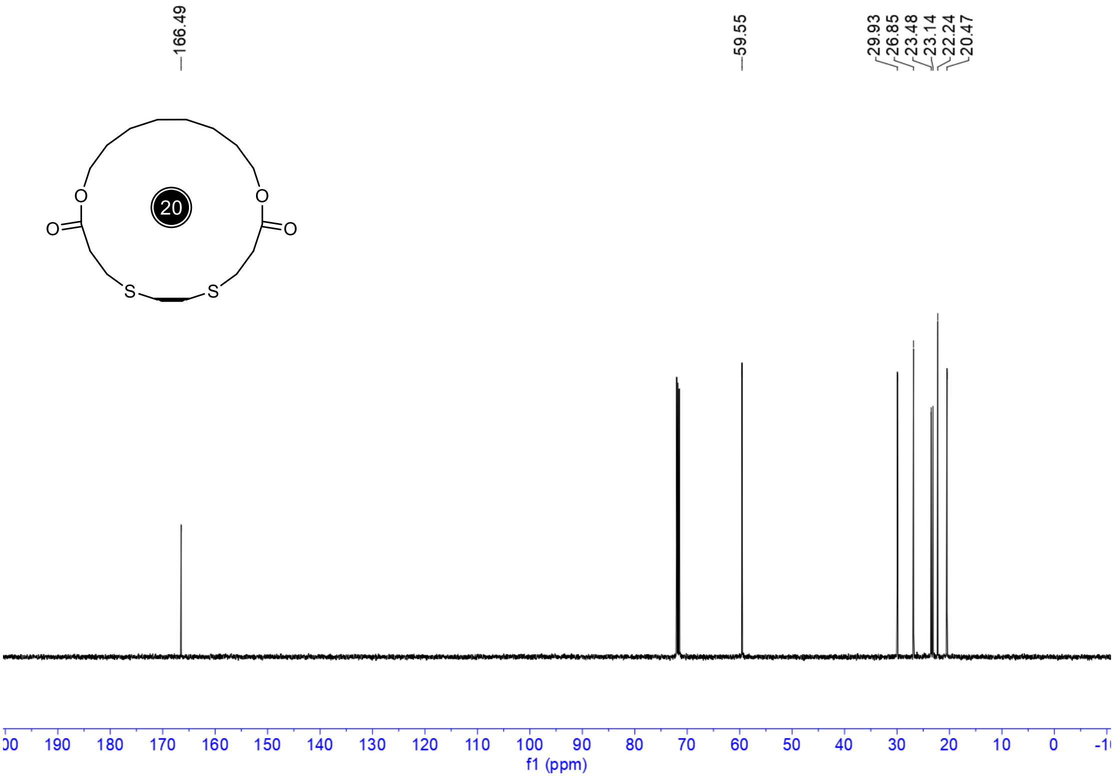

Supplementary Figure 196 | ^{1}H NMR (500 MHz, 298K, $CDCl_3$) of 1,12-Dioxa-5,8-dithiacyclohenicosane-2,11-dione **(16)**

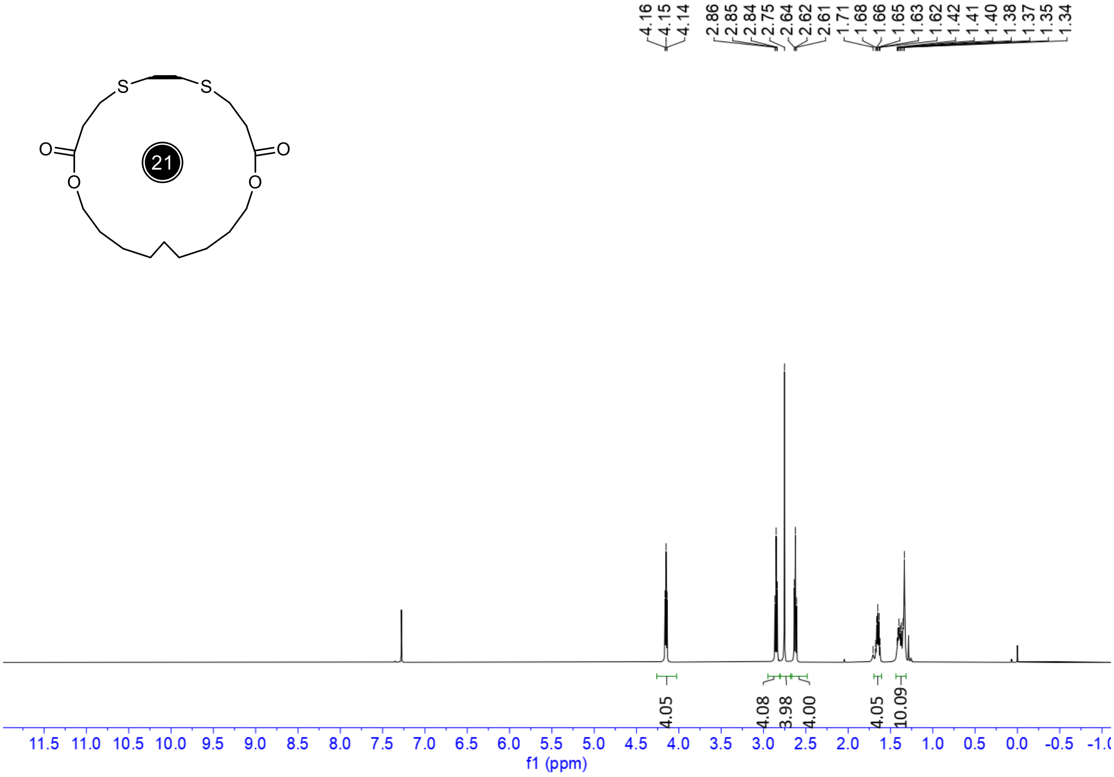

Supplementary Figure 197 | ^{13}C NMR (126 MHz, 298K, $CDCl_3$) of 1,12-Dioxa-5,8-dithiacyclohenicosane-2,11-dione (16)

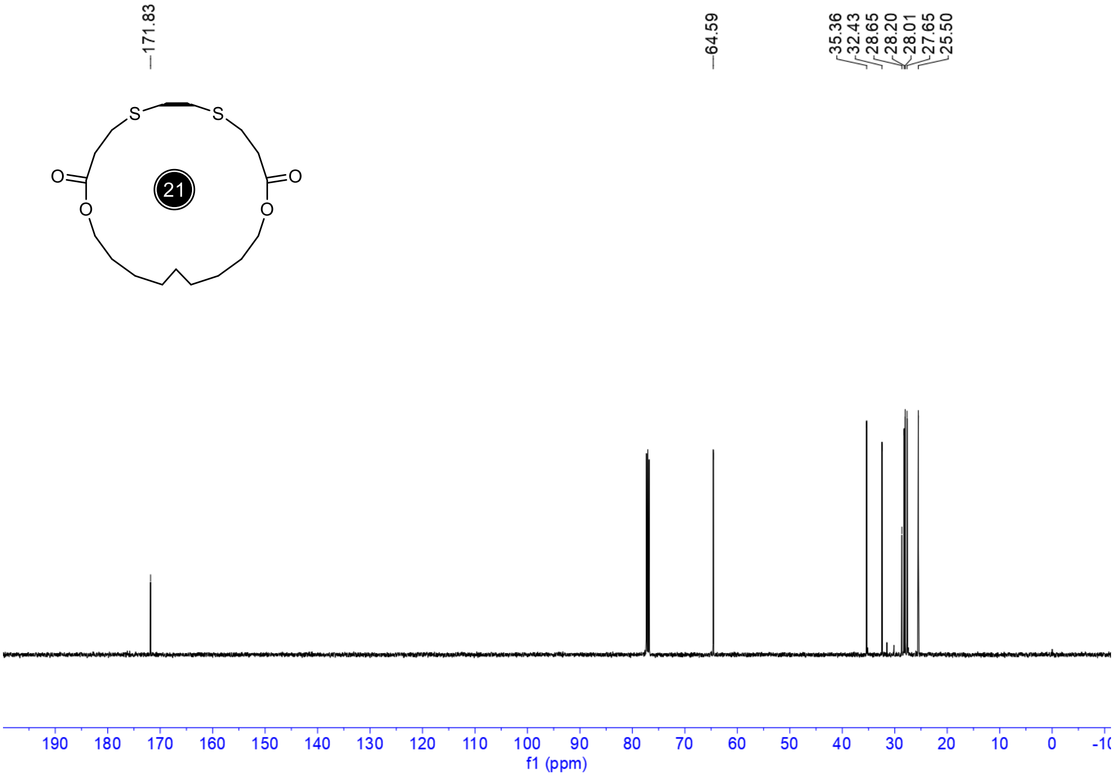

Supplementary Figure 198 | ^{1}H NMR (500 MHz, 298K, $CDCl_3$) of 1,12-Dioxa-5,8-dithiacyclodocosane-2,11-dione **(17)**

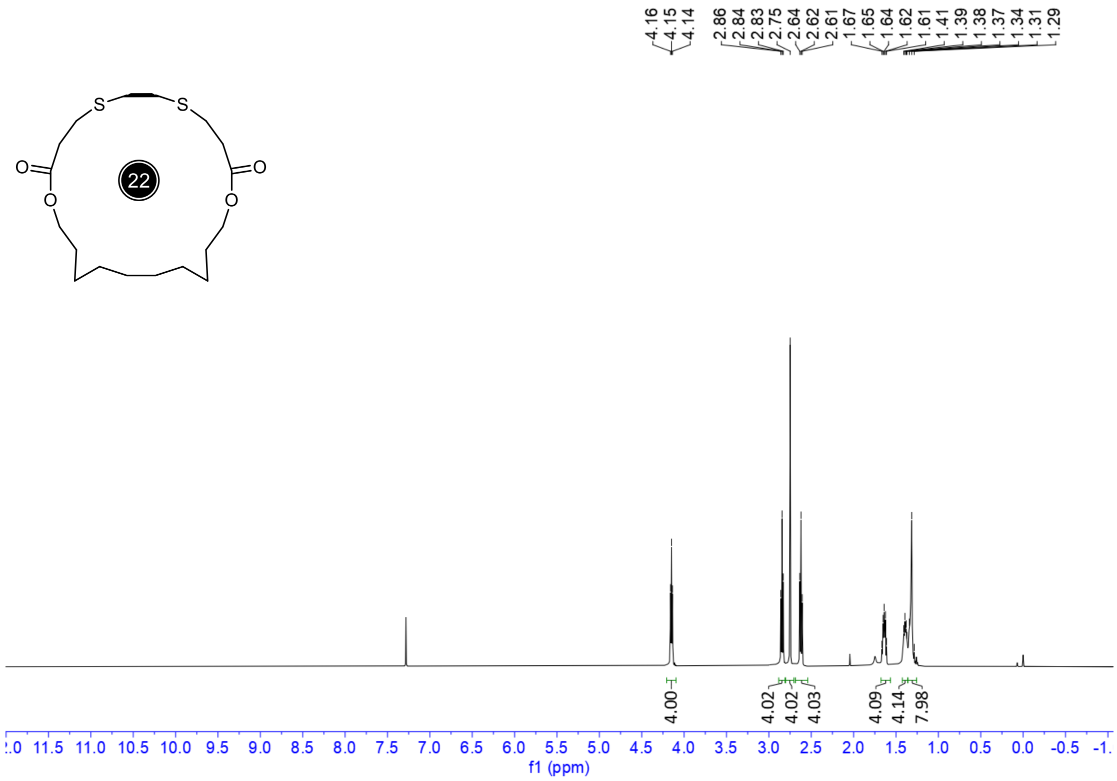

Supplementary Figure 199 | ^{13}C NMR (126 MHz, 298K, $CDCl_3$) of 1,12-Dioxa-5,8-dithiacyclodocosane-2,11-dione **(17)**

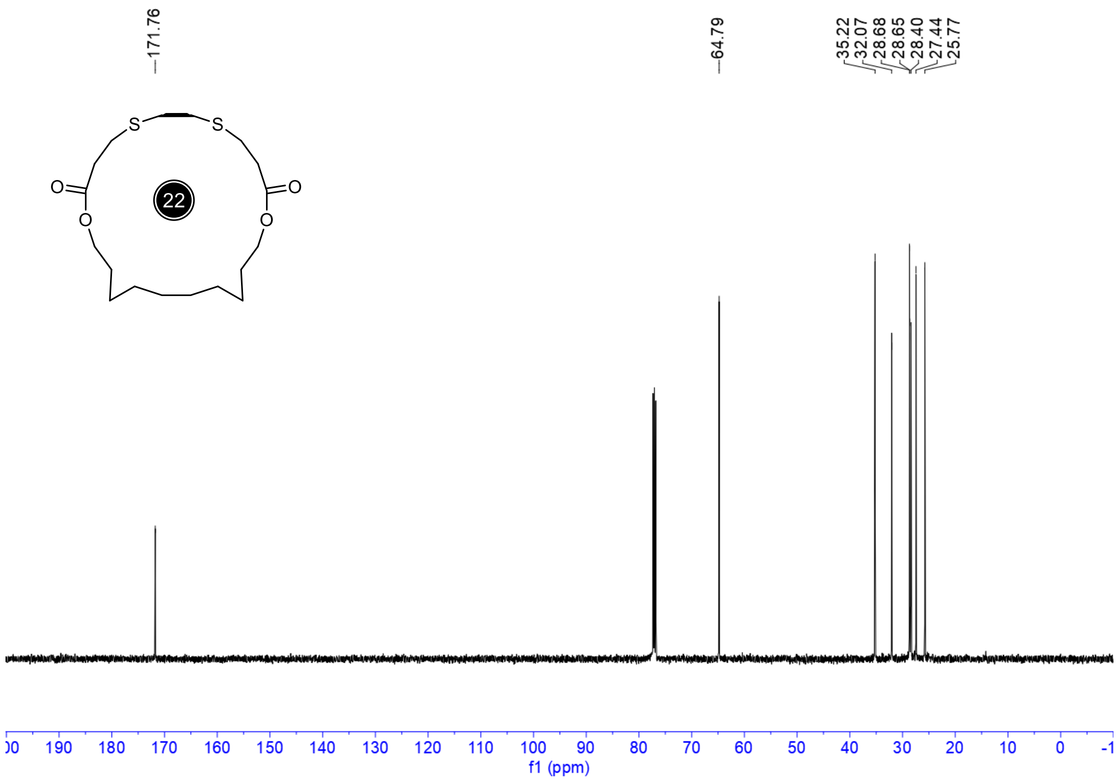

Supplementary Figure 200 | ^{1}H NMR (500 MHz, 298K, $CDCl_3$) of 1,12-Dioxa-5,8-dithiacyclotetracosane-2,11-dione **(18)**

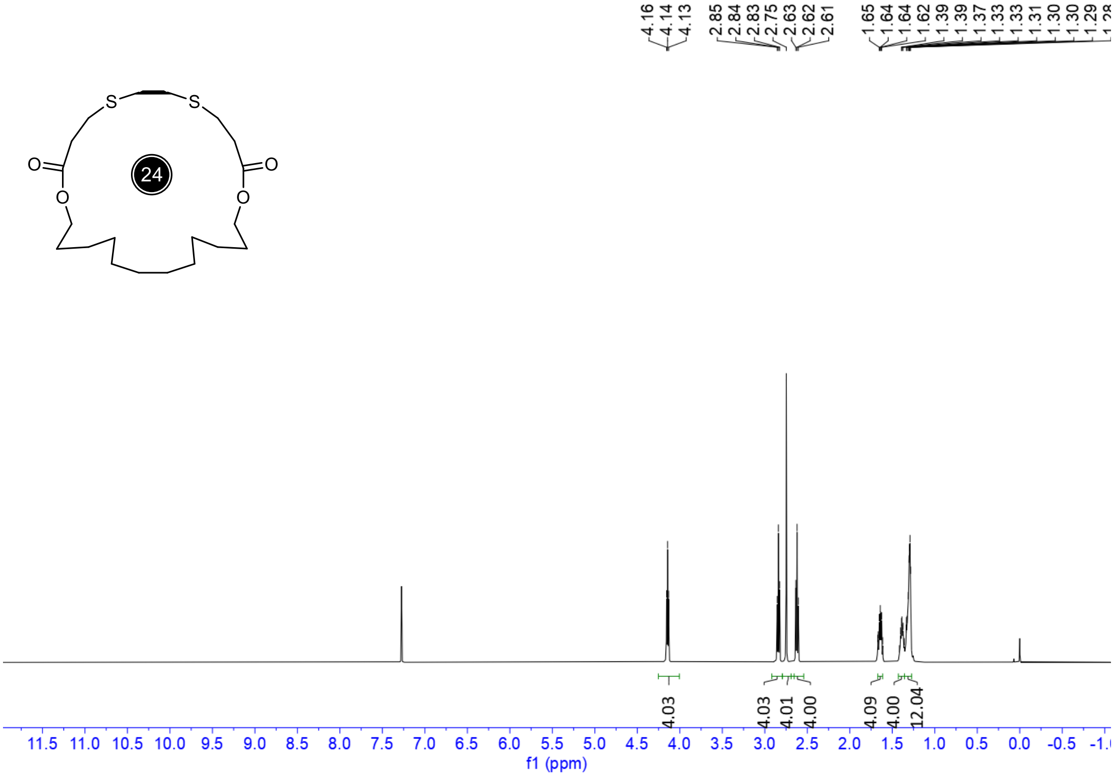

Supplementary Figure 201| ^{13}C NMR (126 MHz, 298K, $CDCl_3$) of 1,12-Dioxa-5,8-dithiacyclotetracosane-2,11-dione **(18)**

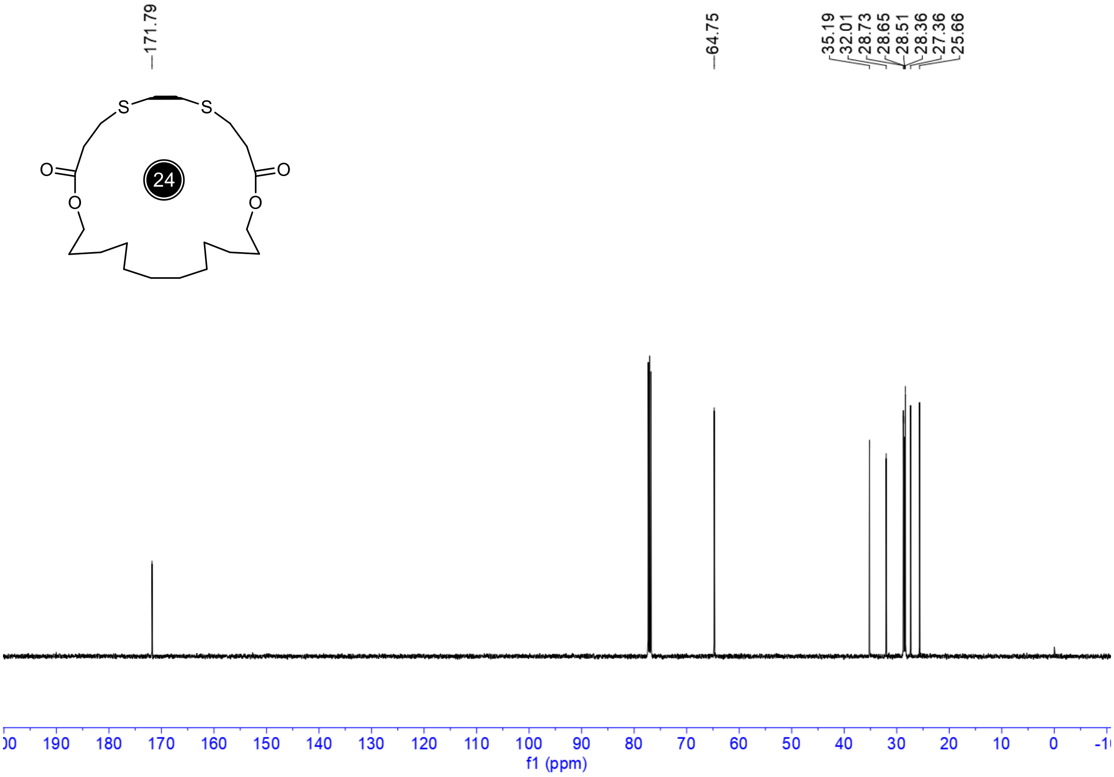

Supplementary Figure 202 | ^{1}H NMR (500 MHz, 298K, $CDCl_3$) of 1,12-Dioxa-5,8-dithiacyclohexacosane-2,11-dione **(19)**

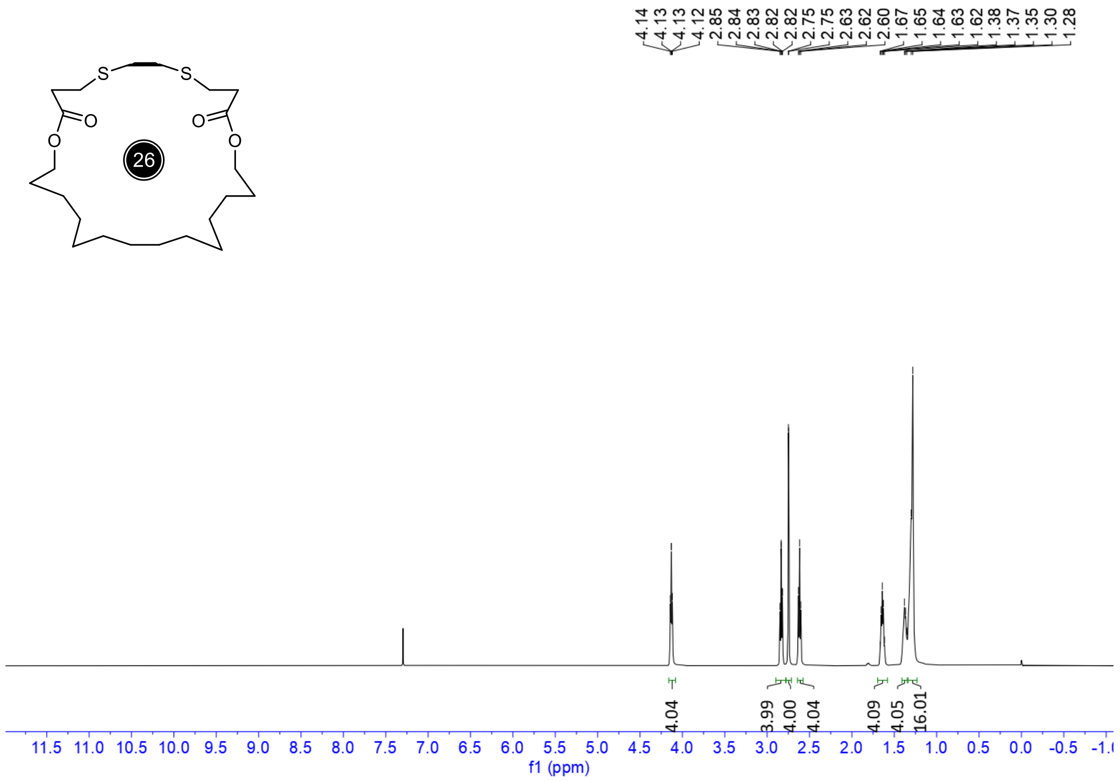

Supplementary Figure 203 | ^{13}C NMR (126 MHz, 298K, $CDCl_3$) of 1,12-Dioxa-5,8-dithiacyclohexacosane-2,11-dione **(19)**

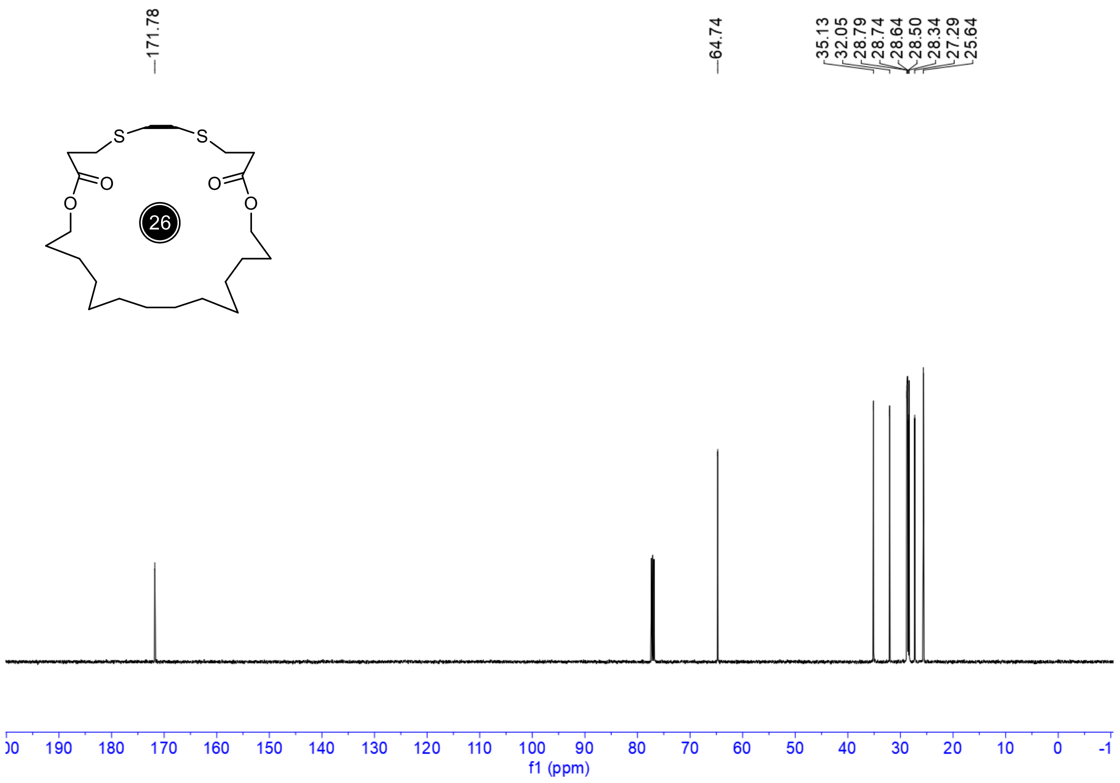

Supplementary Figure 204 | ^{1}H NMR (500 MHz, 298K, $CDCl_3$) of 3,3,4,4-Tetrafluoro-1,6-dioxa-13,16-dithiacyclodocosane-2,5-dione **(20)**

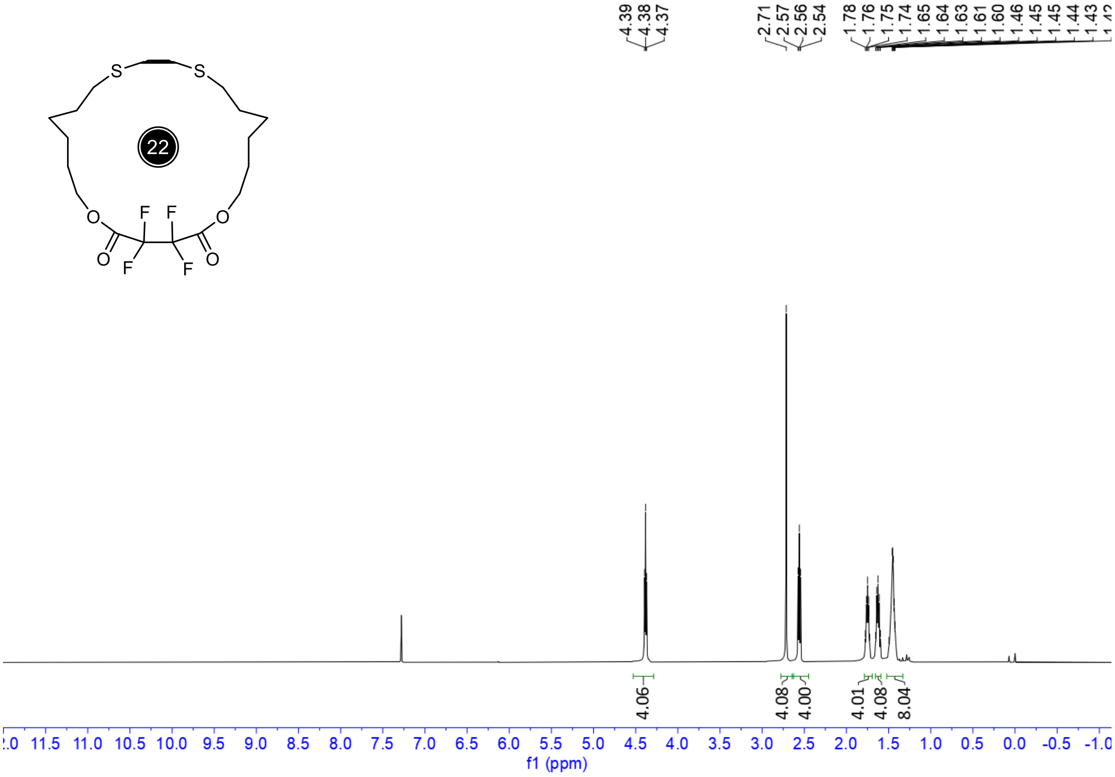

Supplementary Figure 205 | ^{13}C NMR (126 MHz, 298K, $CDCl_3$) of 3,3,4,4-Tetrafluoro-1,6-dioxa-13,16-dithiacyclodocosane-2,5-dione **(20)**

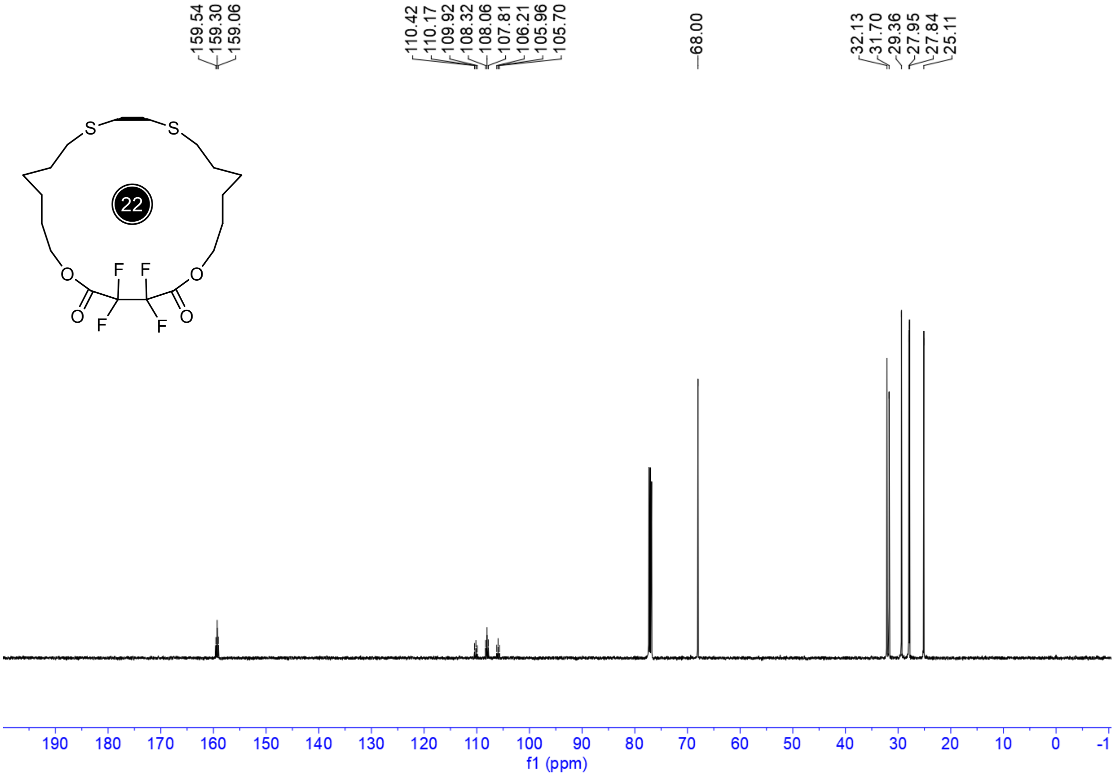

Supplementary Figure 206 | ^{19}F NMR (471 MHz, 298K, $CDCl_3$) 3,3,4,4-Tetrafluoro-1,6-dioxa-13,16-dithiacyclodocosane-2,5-dione **(20)**

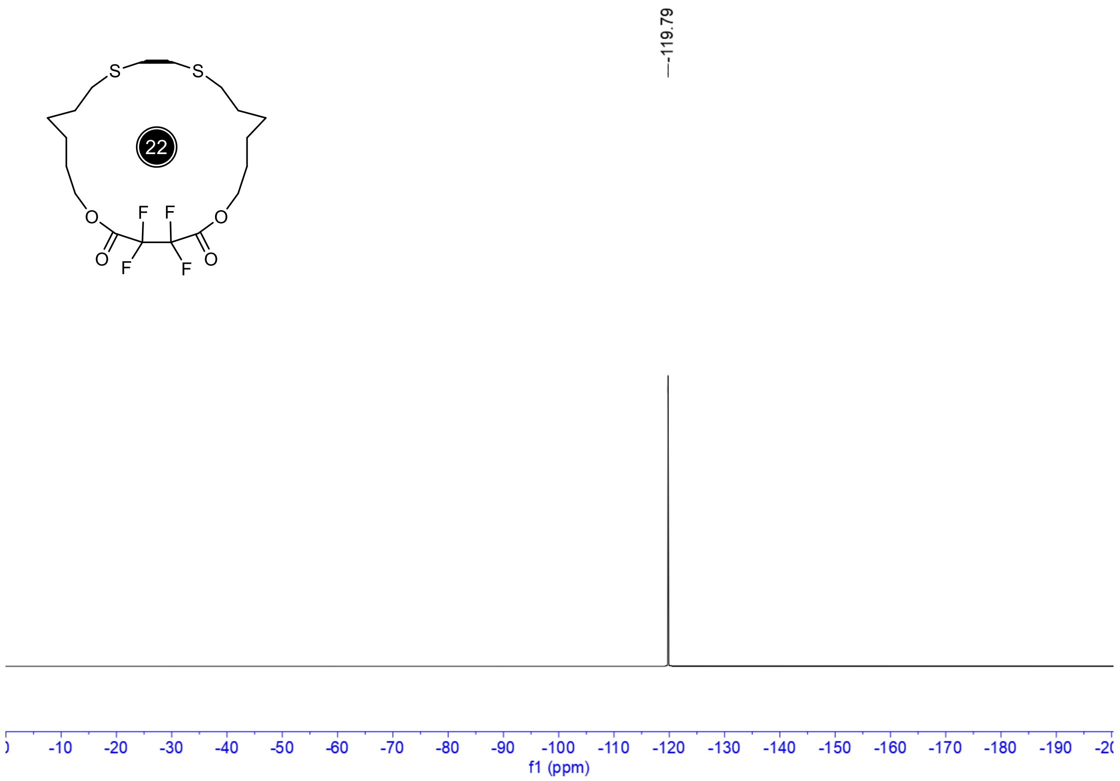

Supplementary Figure 207 | ^{1}H NMR (500 MHz, 298K, $CDCl_3$) of 3,3,4,4,5,5,6,6-Octafluoro-1,8-dioxa-15,18-dithiacyclotetracosane-2,7-dione **(21)**

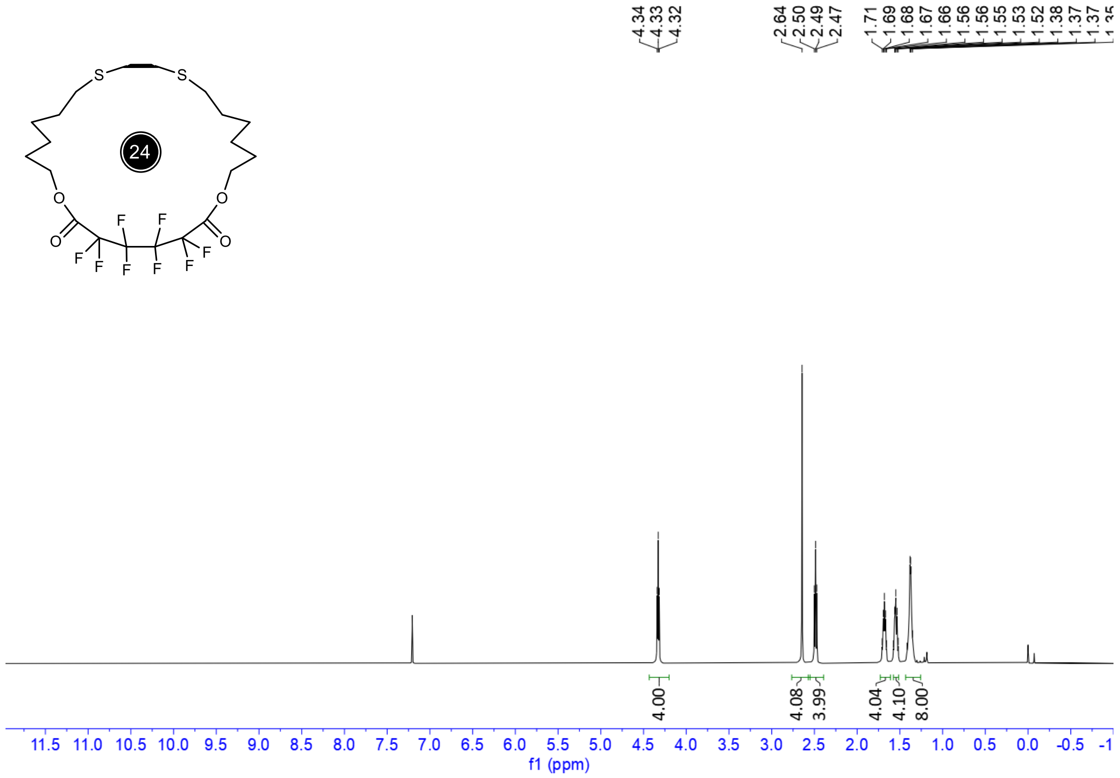

Supplementary Figure 208| ^{13}C NMR (126 MHz, 298K, $CDCl_3$) of 3,3,4,4,5,5,6,6-Octafluoro-1,8-dioxa-15,18-dithiacyclotetracosane-2,7-dione **(21)**

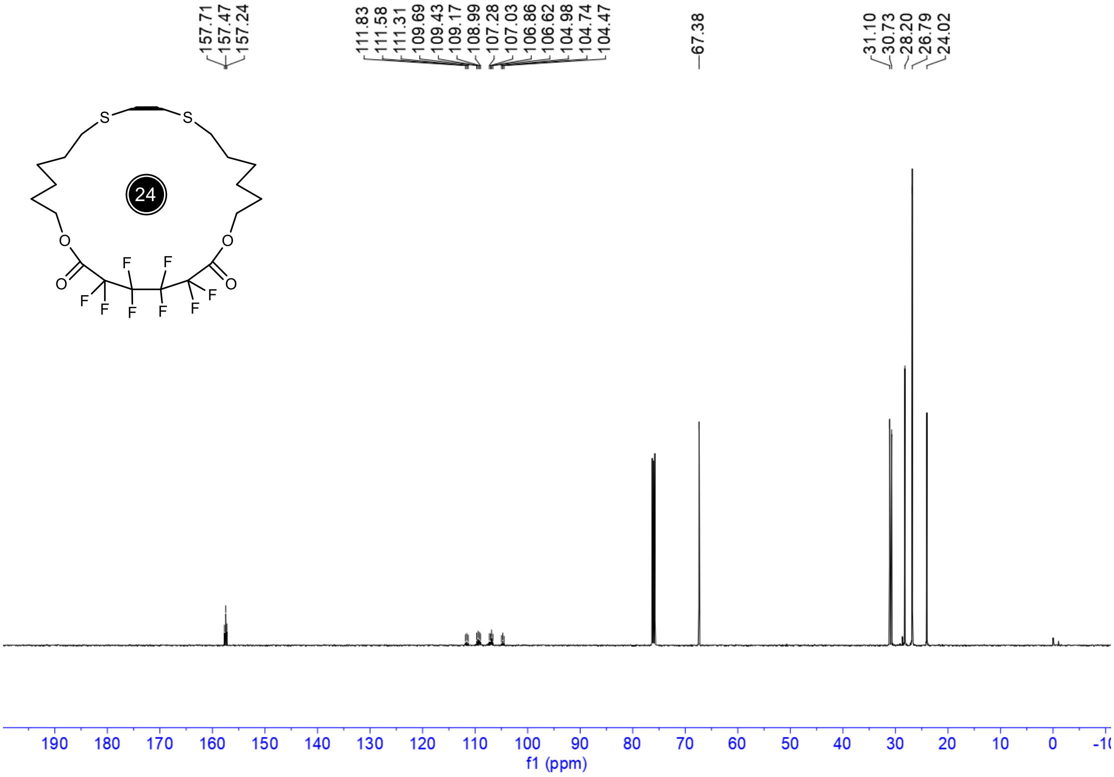

Supplementary Figure 209 | ^{19}F NMR (471 MHz, 298K, $CDCl_3$) 3,3,4,4,5,5,6,6-Octafluoro-1,8-dioxa-15,18-dithiacyclotetracosane-2,7-dione **(21)**

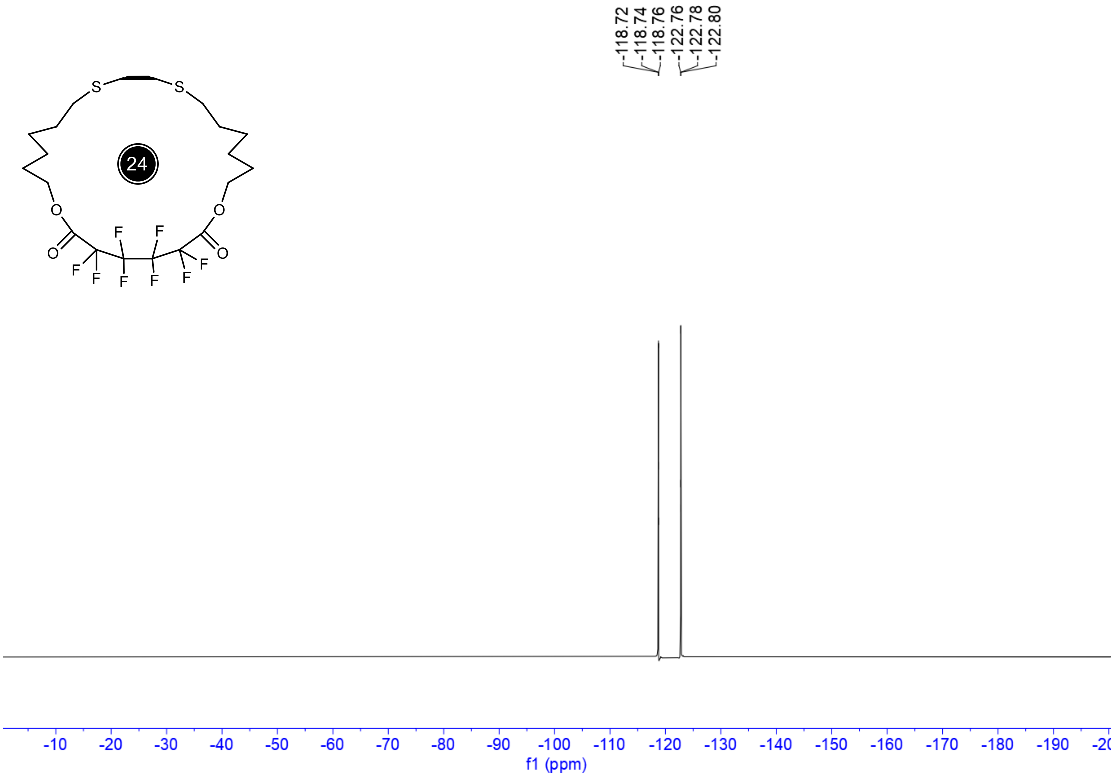

Supplementary Figure 210 | ^{1}H NMR (500 MHz, 298K, $CDCl_3$) of 1,4,7-Trioxa-14,17-dithiacyclotricosane-2,6-dione **(22)**

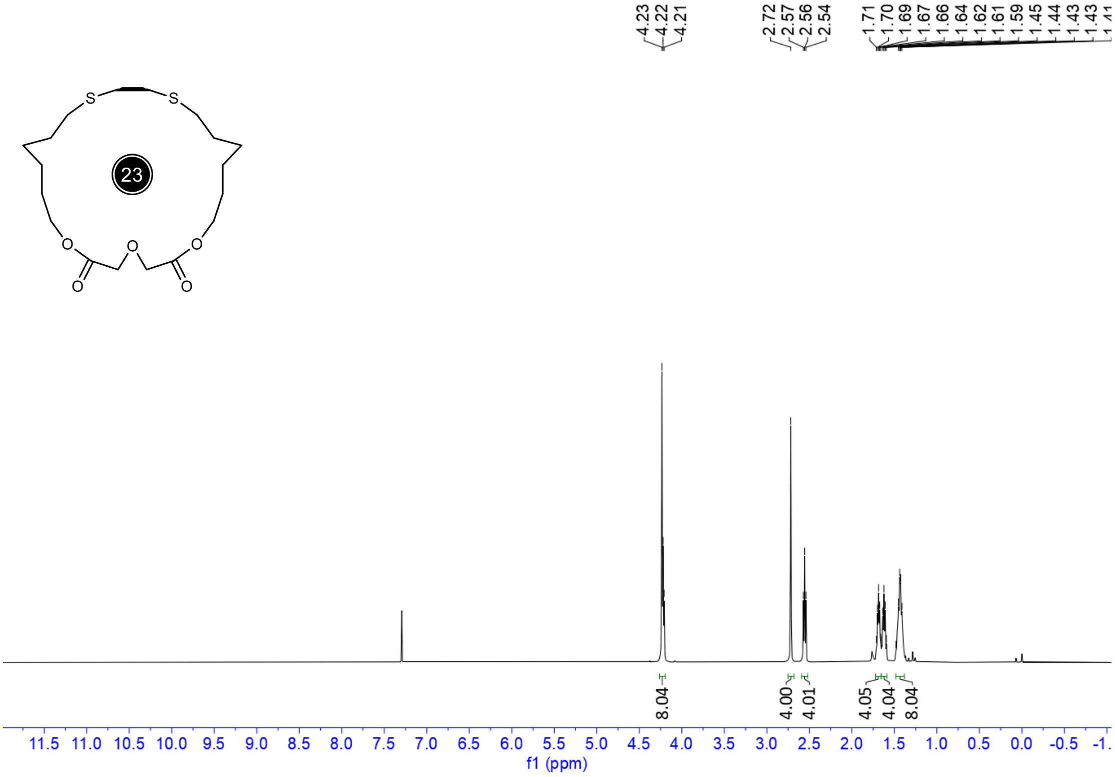

Supplementary Figure 211 | ^{13}C NMR (126 MHz, 298K, $CDCl_3$) of **1**,4,7-Trioxa-14,17-dithiacyclotricosane-2,6-dione **(22)**

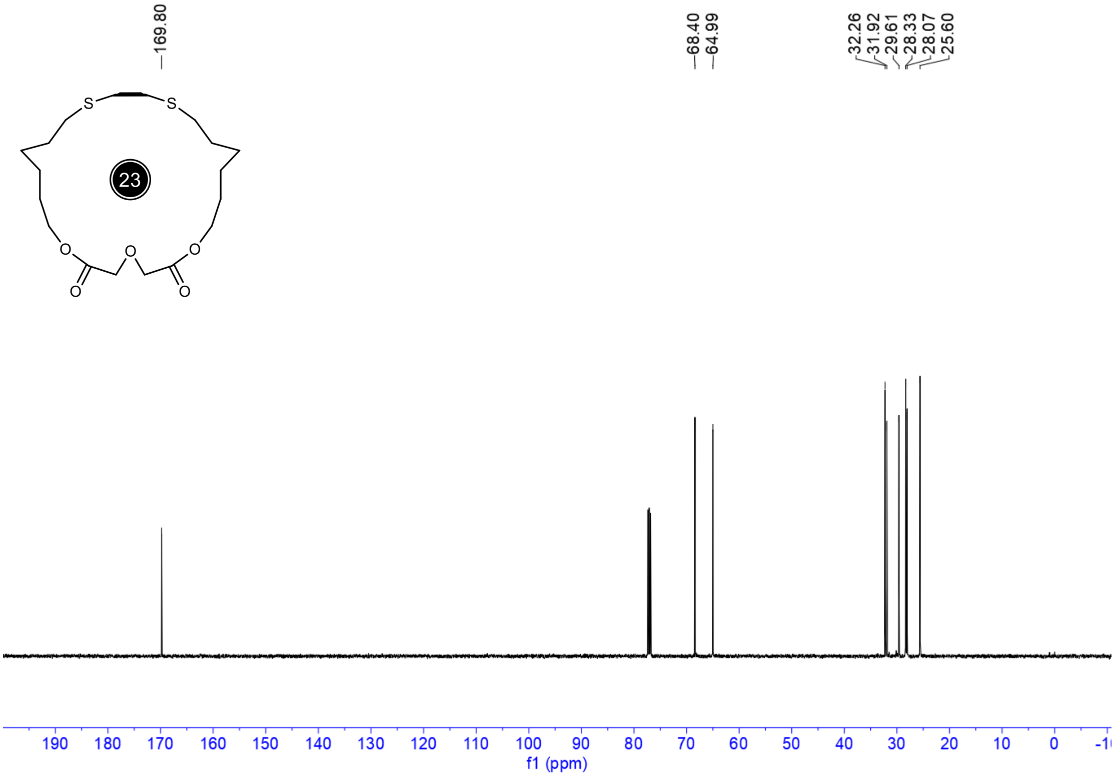

Supplementary Figure 212 | ^{1}H NMR (500 MHz, 298K, $CDCl_3$) of 1,4,7-Trioxa-11,14-dithiacycloheptadecane-8,17-dione **(23)**

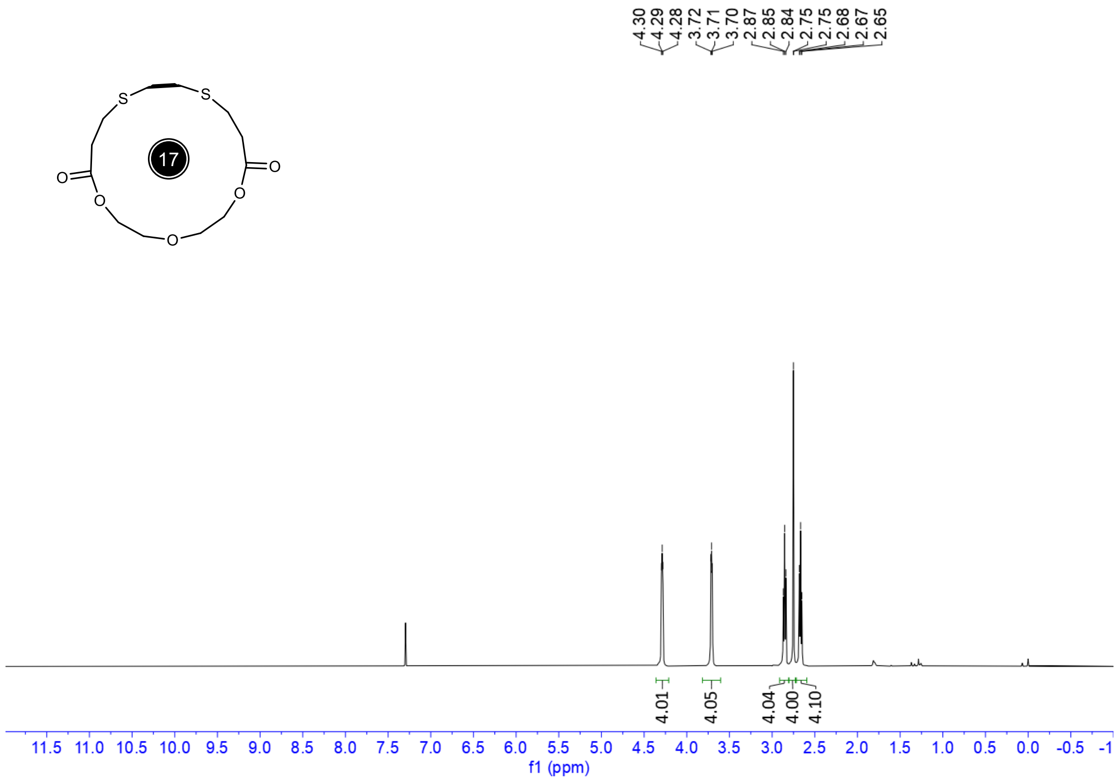

Supplementary Figure 213 | ^{13}C NMR (126 MHz, 298K, $CDCl_3$) of 1,4,7-Trioxa-11,14-dithiacycloheptadecane-8,17-dione **(23)**

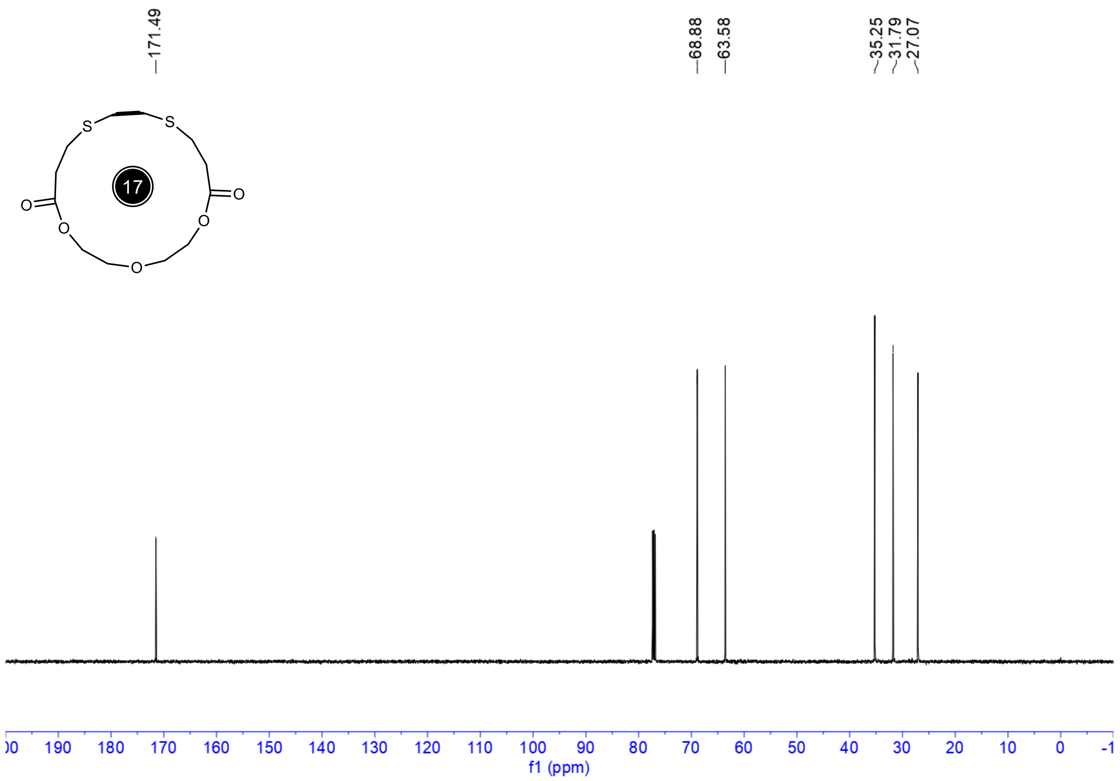

Supplementary Figure 214 | ^{1}H NMR (500 MHz, 298K, $CDCl_3$) of 1,4,7,10-Tetraoxa-14,17-dithiacycloicosane-11,20-dione **(24)**

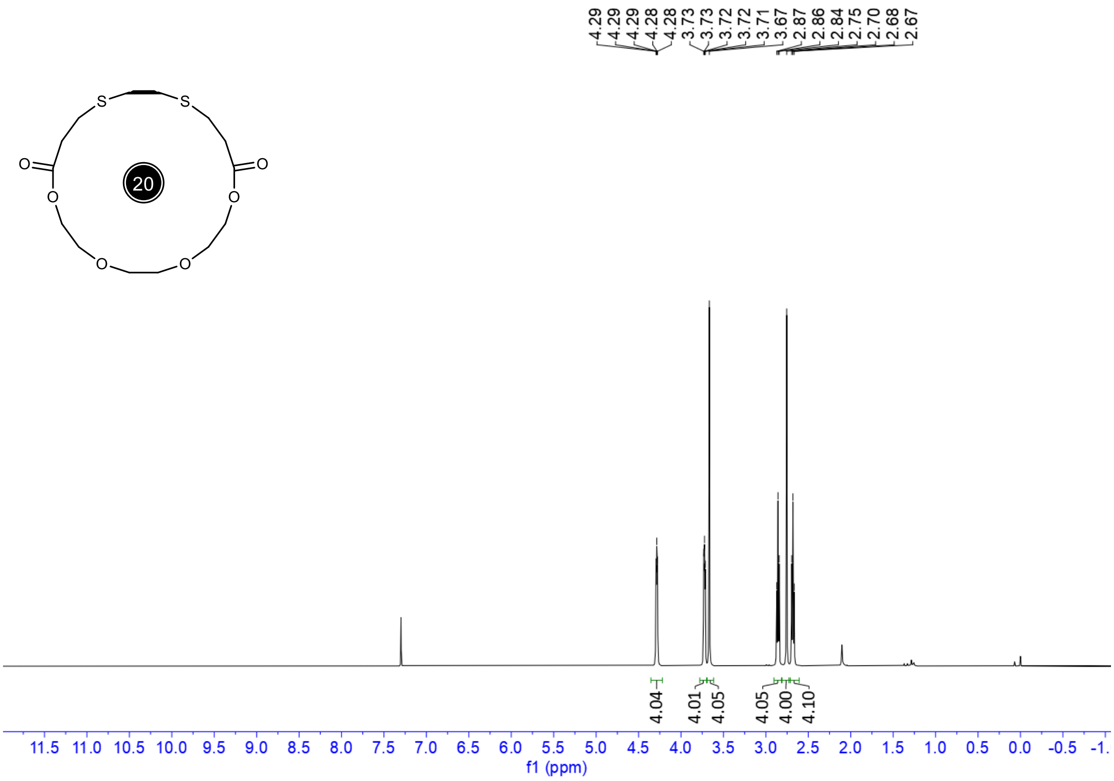

Supplementary Figure 215 | ^{13}C NMR (126 MHz, 298K, $CDCl_3$) of 1,4,7,10-Tetraoxa-14,17-dithiacycloicosane-11,20-dione **(24)**

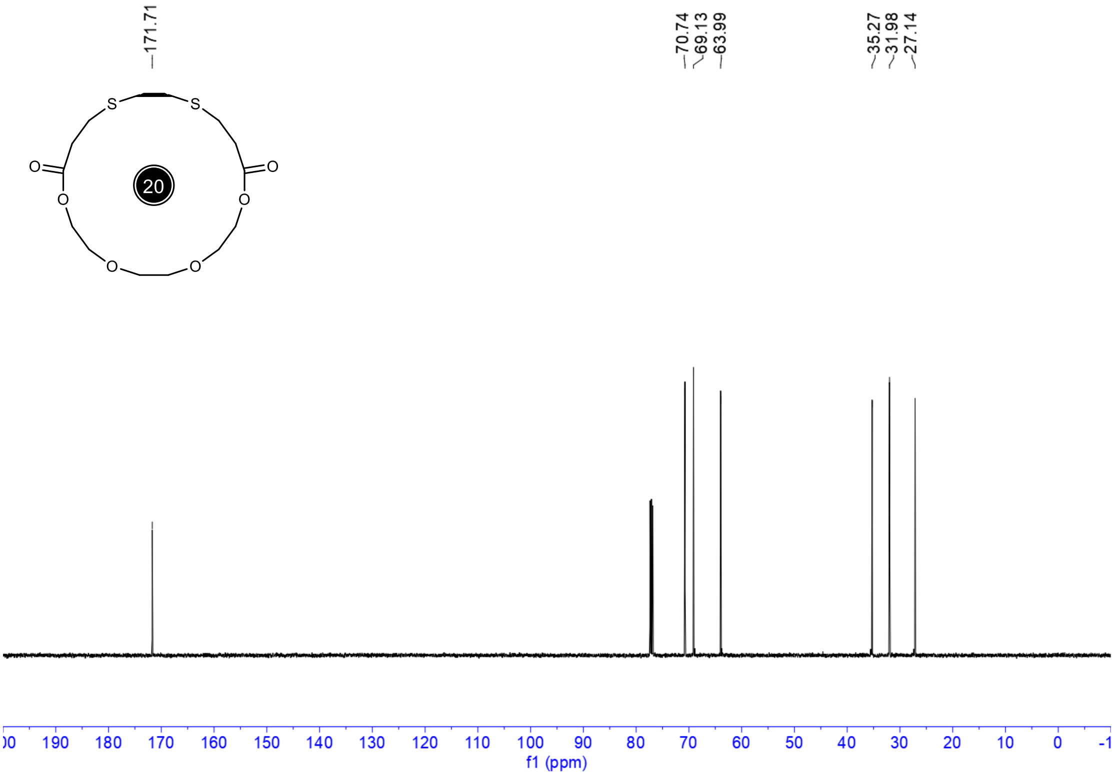

Supplementary Figure 216 | ^{1}H NMR (500 MHz, 298K, $CDCl_3$) of 1,4,7,10,13-Pentaoxa-17,20-dithiacyclotricosane-14,23-dione **(25)**

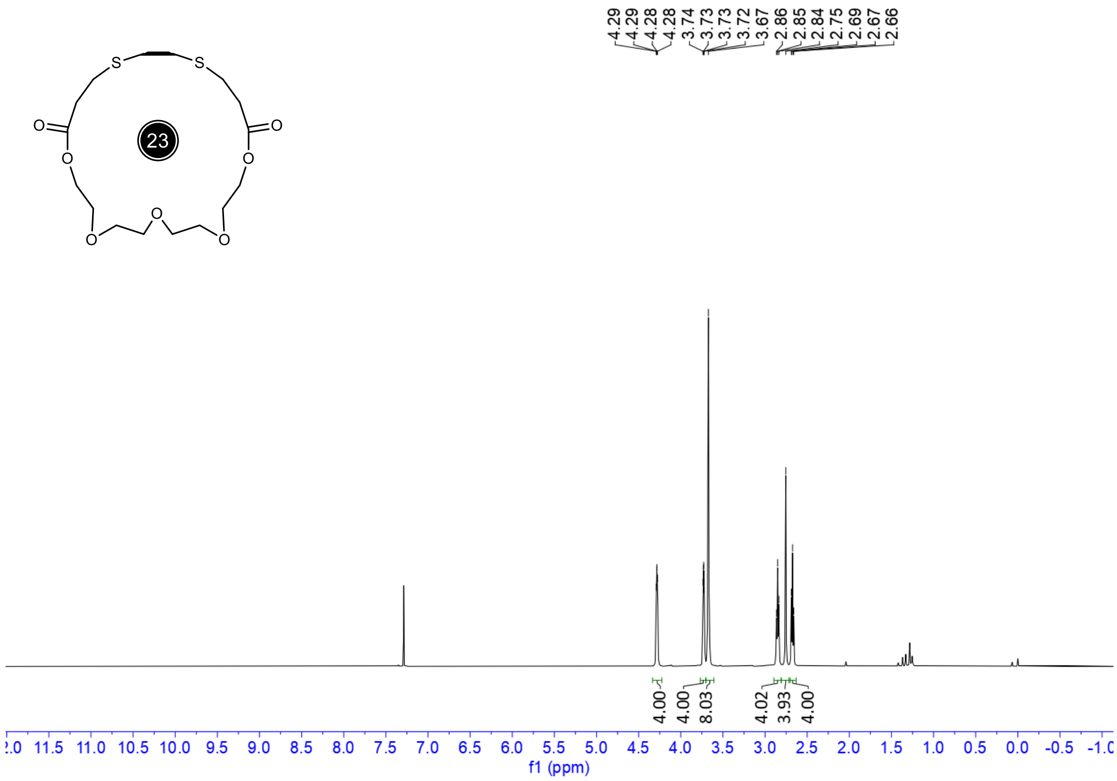

Supplementary Figure 217 | ^{13}C NMR (126 MHz, 298K, $CDCl_3$) of 1,4,7,10,13-Pentaoxa-17,20-dithiacyclotricosane-14,23-dione **(25)**

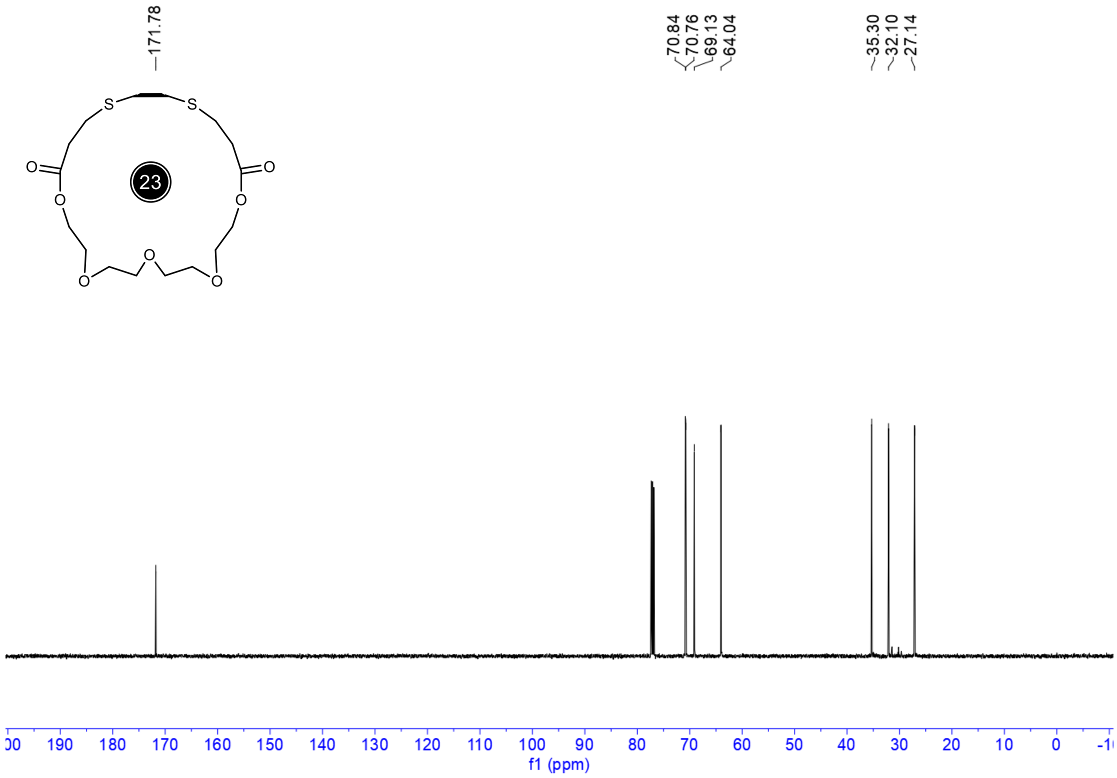

Supplementary Figure 218 | ^{1}H NMR (500 MHz, 298K, $CDCl_3$) of 1,4,7,10,13,16,19,22,25-nonaoxa-29,32-dithiacyclopentatriacontane-26,35-dione **(26)**

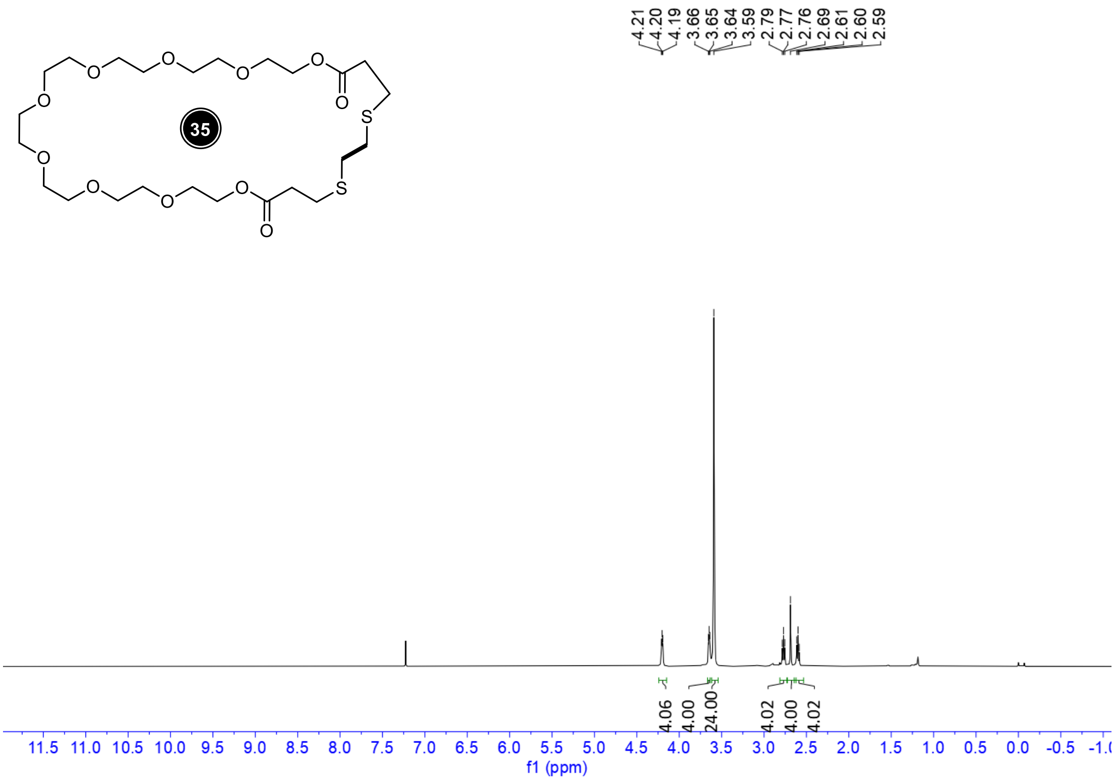

Supplementary Figure 219 | ^{13}C NMR (126 MHz, 298K, $CDCl_3$) of 1,4,7,10,13,16,19,22,25-nonaoxa-29,32-dithiacyclopentatriacontane-26,35-dione **(26)**

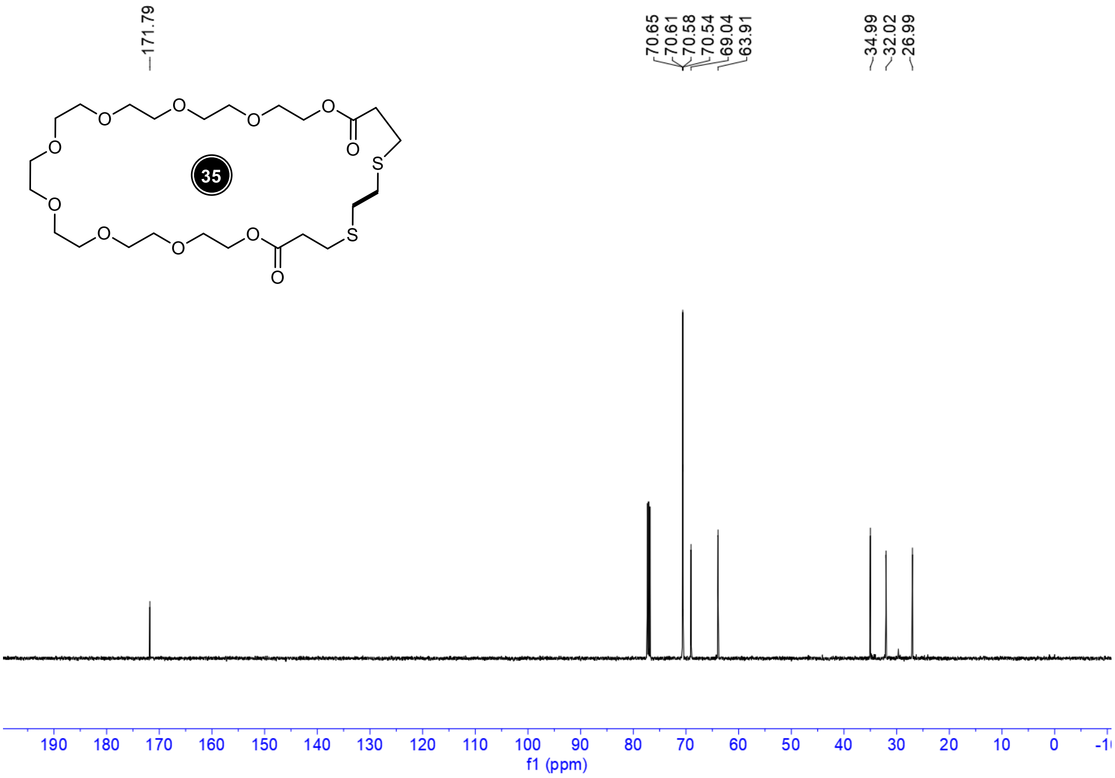

Supplementary Figure 220 | ^{1}H NMR (500 MHz, 298K, $CDCl_3$) of 4-Phenyl-1,7-dioxa-11,14-dithia-4-azacycloheptadecane-8,17-dione **(27)**

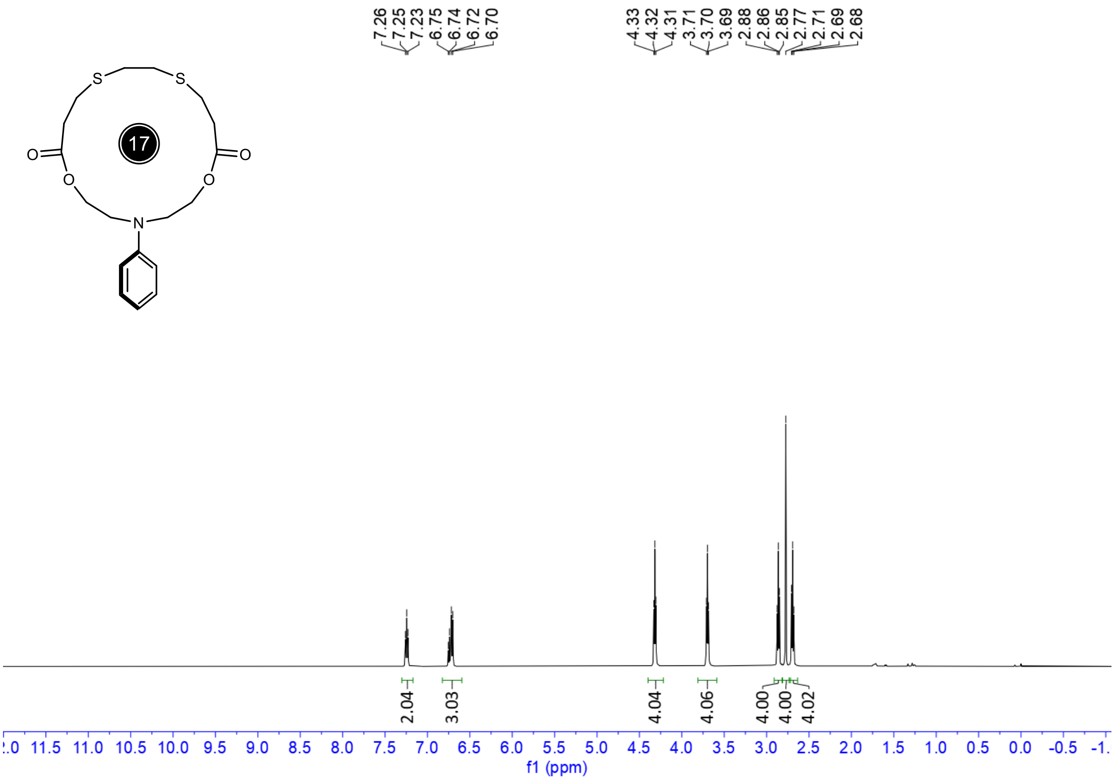

Supplementary Figure 221 | ^{13}C NMR (126 MHz, 298K, $CDCl_3$) of 4-Phenyl-1,7-dioxa-11,14-dithia-4-azacycloheptadecane-8,17-dione **(27)**

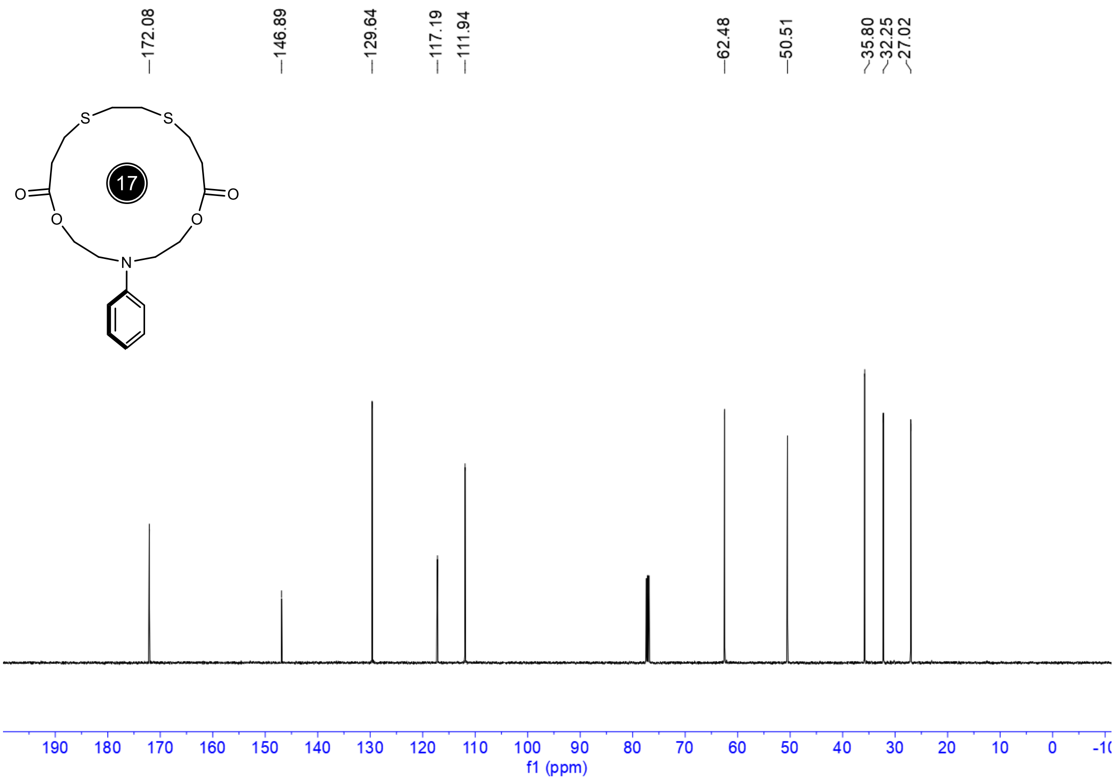

Supplementary Figure 222 | ^{1}H NMR (500 MHz, 298K, $CDCl_3$) of 4-Methyl-1,7-dioxa-14,17-dithia-4-azacyclotricosane-2,6-dione **(28)**

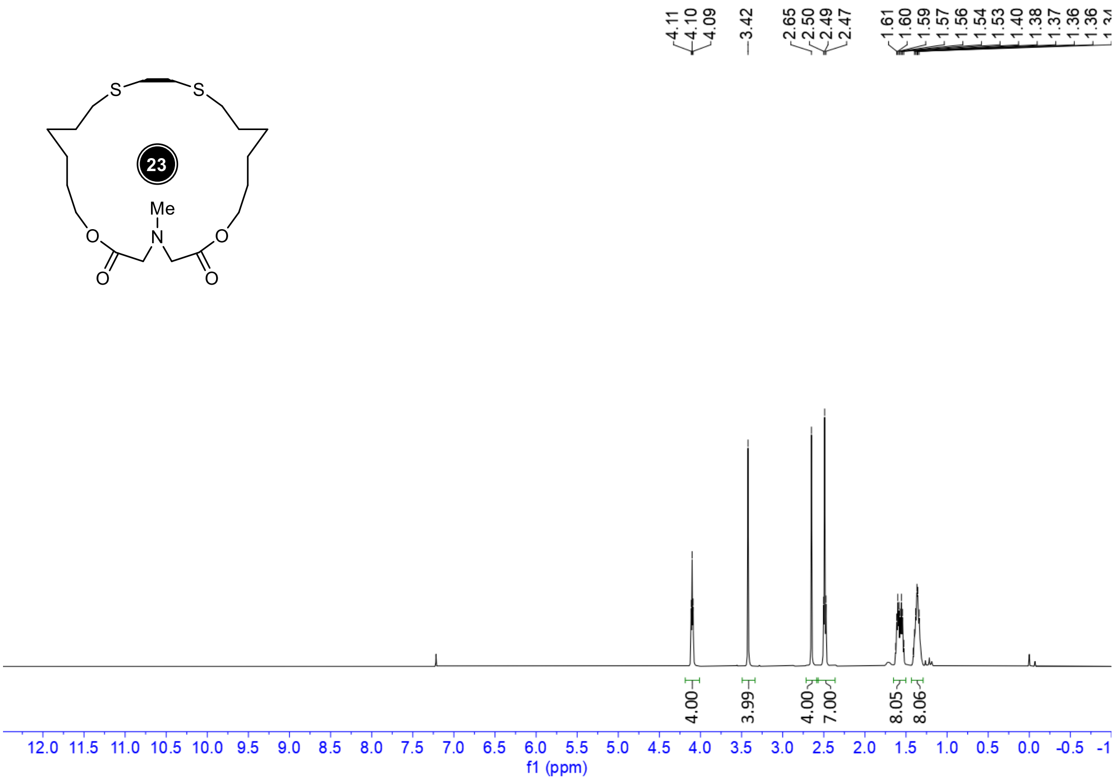

Supplementary Figure 223 | ^{13}C NMR (126 MHz, 298K, $CDCl_3$) of 4-Methyl-1,7-dioxa-14,17-dithia-4-azacyclotricosane-2,6-dione **(28)**

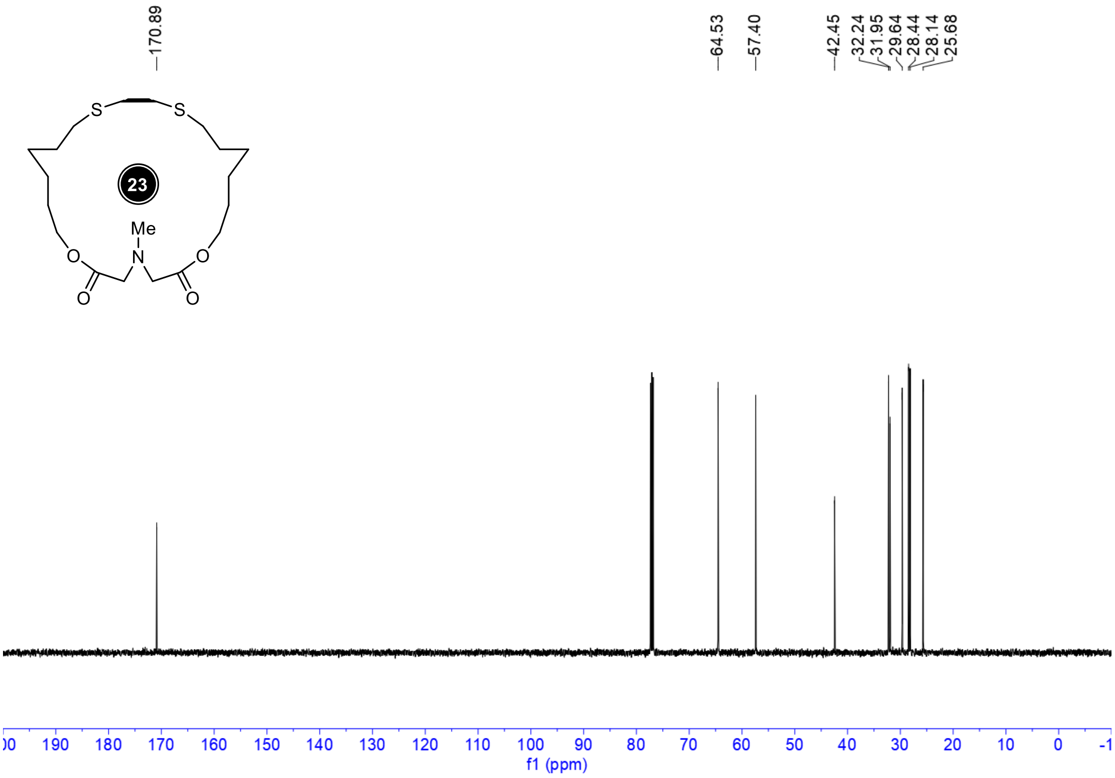

Supplementary Figure 224 | ^{1}H NMR (500 MHz, 298K, $CDCl_3$) of *Trans*-3,20-dioxa-10,13-dithiabicyclo[20.2.2]hexacosane-2,21-dione **(29)**

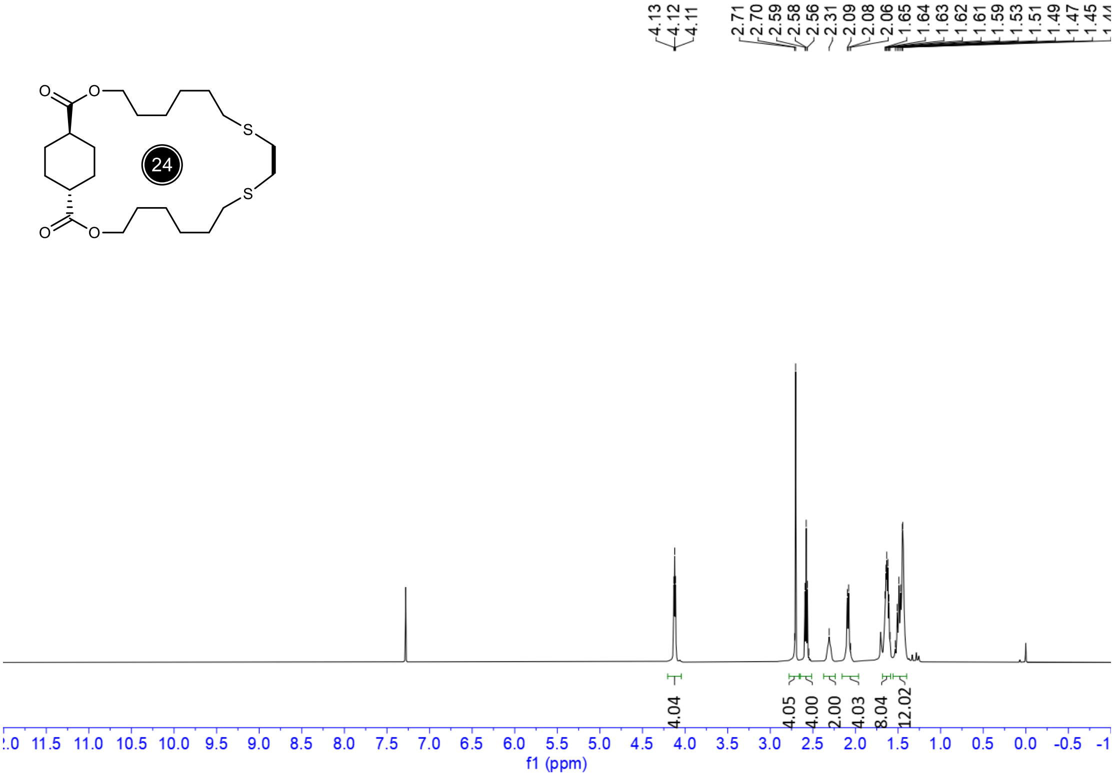

Supplementary Figure 225 | ^{13}C NMR (126 MHz, 298K, $CDCl_3$) of *Trans*-3,20-dioxa-10,13-dithiabicyclo[20.2.2]hexacosane-2,21-dione **(29)**

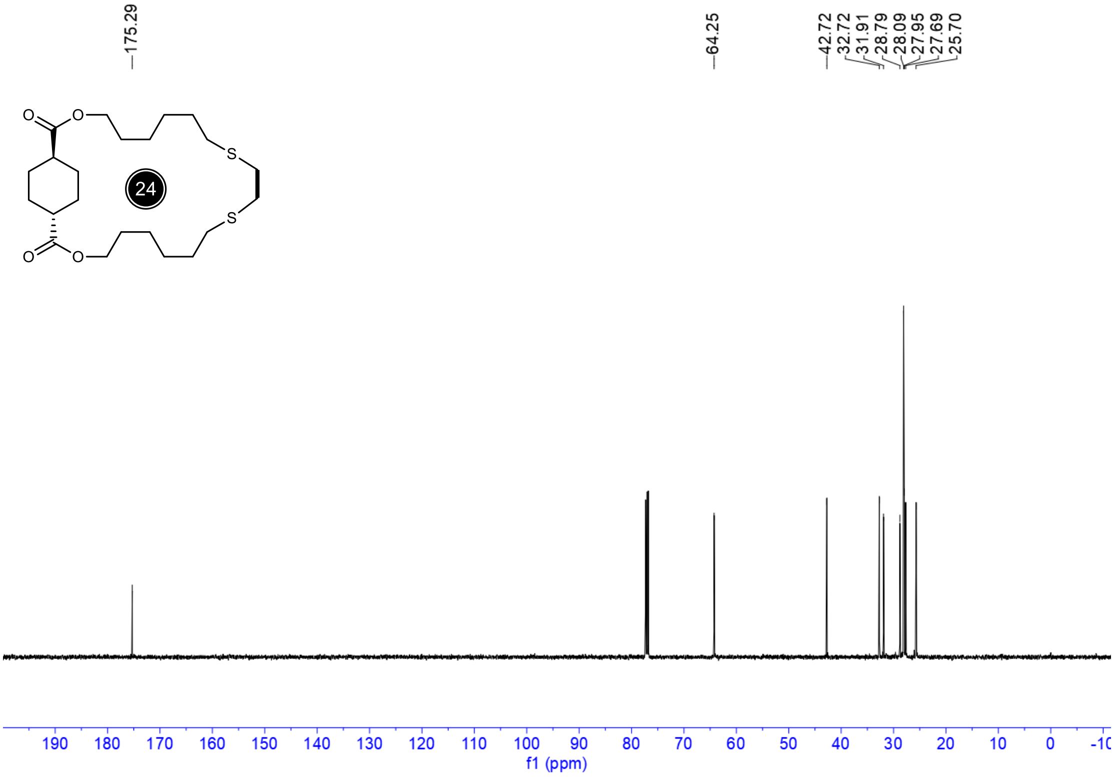

Supplementary Figure 226 | ^{1}H NMR (500 MHz, 298K, $CDCl_3$) of 3,20-Dioxa-10,13-dithiatricyclo[20.2.2.21,22]octacosane-2,21-dione **(30)**

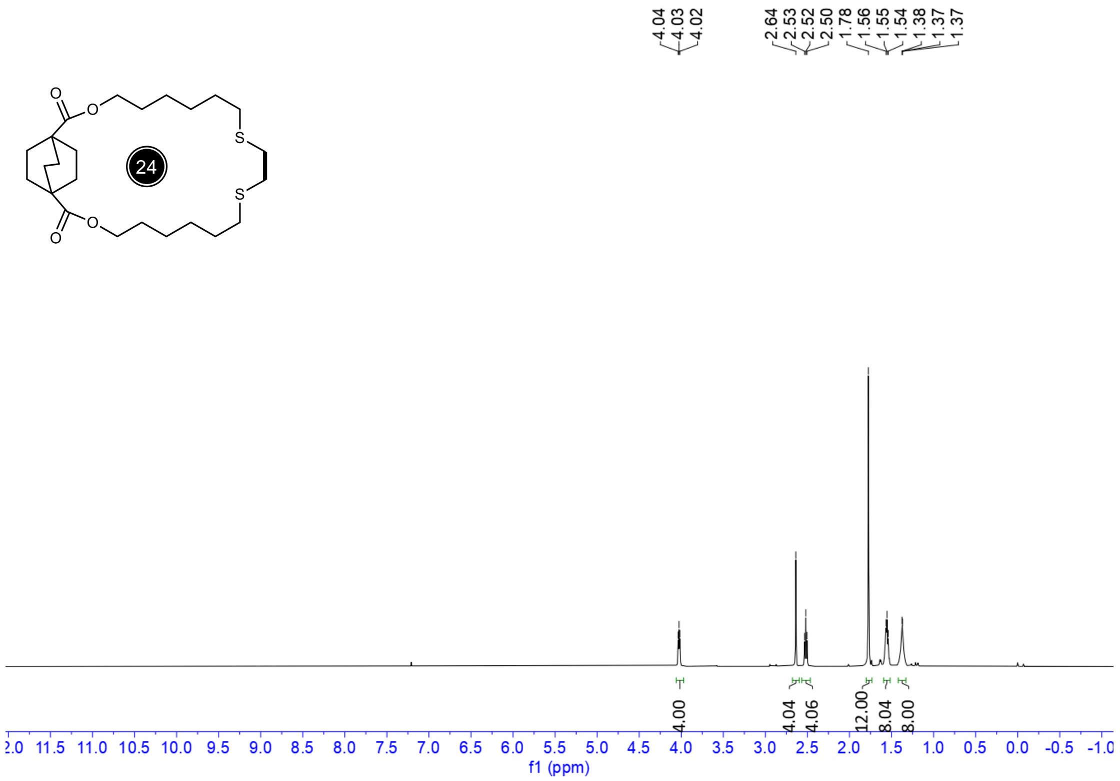

Supplementary Figure 227 | ^{13}C NMR (126 MHz, 298K, $CDCl_3$) of 3,20-Dioxa-10,13-dithiatricyclo[20.2.2.21,22]octacosane-2,21-dione **(30)**

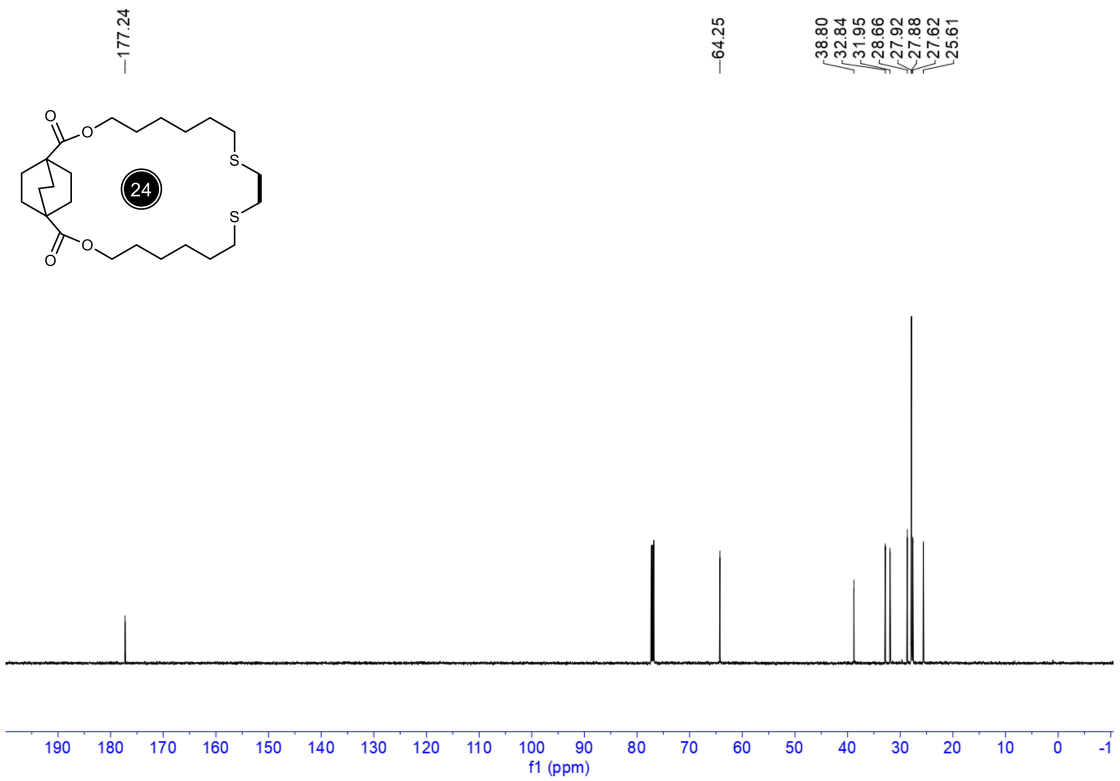

Supplementary Figure 228 | ^{1}H NMR (500 MHz, 298K, $CDCl_3$) of 3,20-Dioxa-10,13-dithiatetracyclo[20.5.1.1^{1,24}.1^{22,26}]triacontane-2,21-dione **(31)**

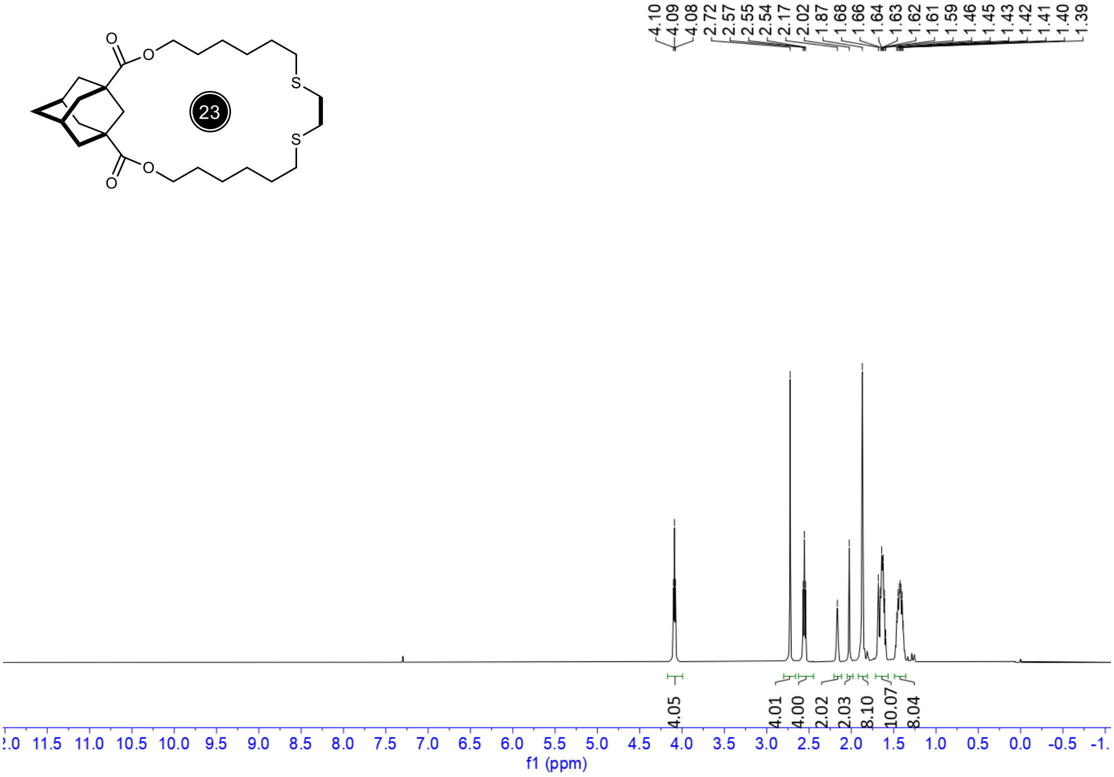

Supplementary Figure 229 | ^{13}C NMR (126 MHz, 298K, $CDCl_3$) of 3,20-Dioxa-10,13-dithiatetracyclo[20.5.1.1^{1,24}.1^{22,26}]triacontane-2,21-dione **(31)**

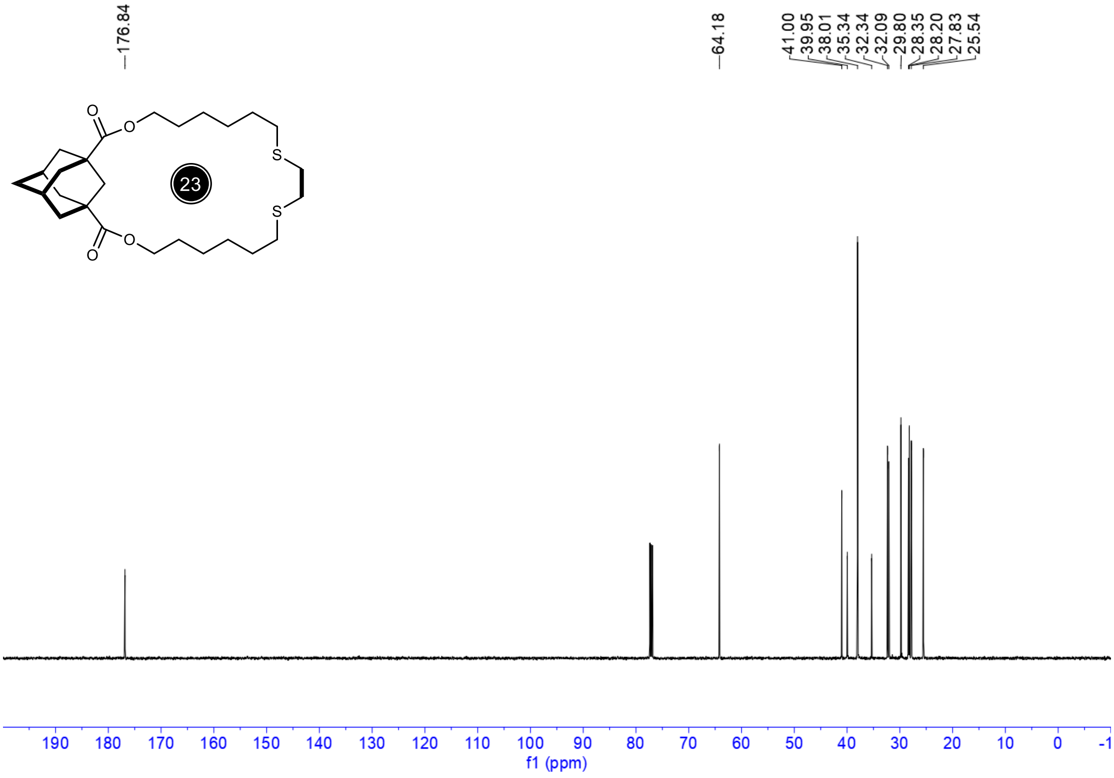

Supplementary Figure 230 | ^{1}H NMR (500 MHz, 298K, $CDCl_3$) of 4,15-Dioxa-8,11-dithia-1,18-diazabicyclo[16.2.2]docosane-5,14-dione **(32)**

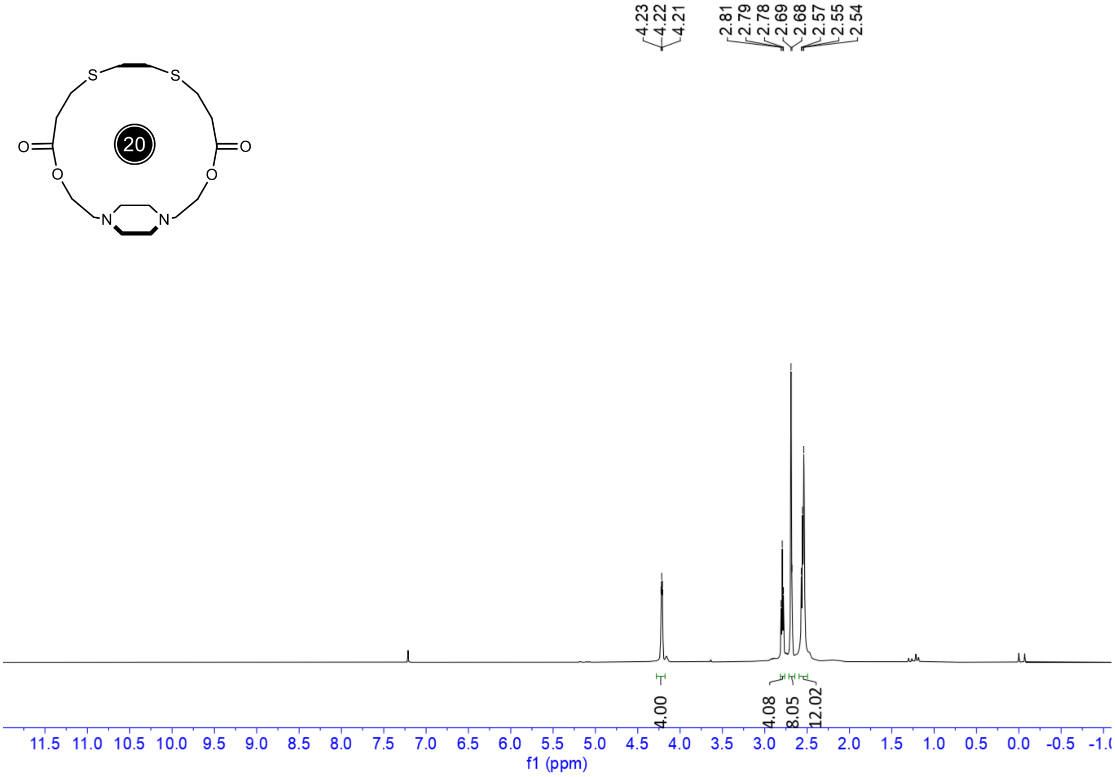

Supplementary Figure 231 | ^{13}C NMR (126 MHz, 298K, $CDCl_3$) of 4,15-Dioxa-8,11-dithia-1,18-diazabicyclo[16.2.2]docosane-5,14-dione **(32)**

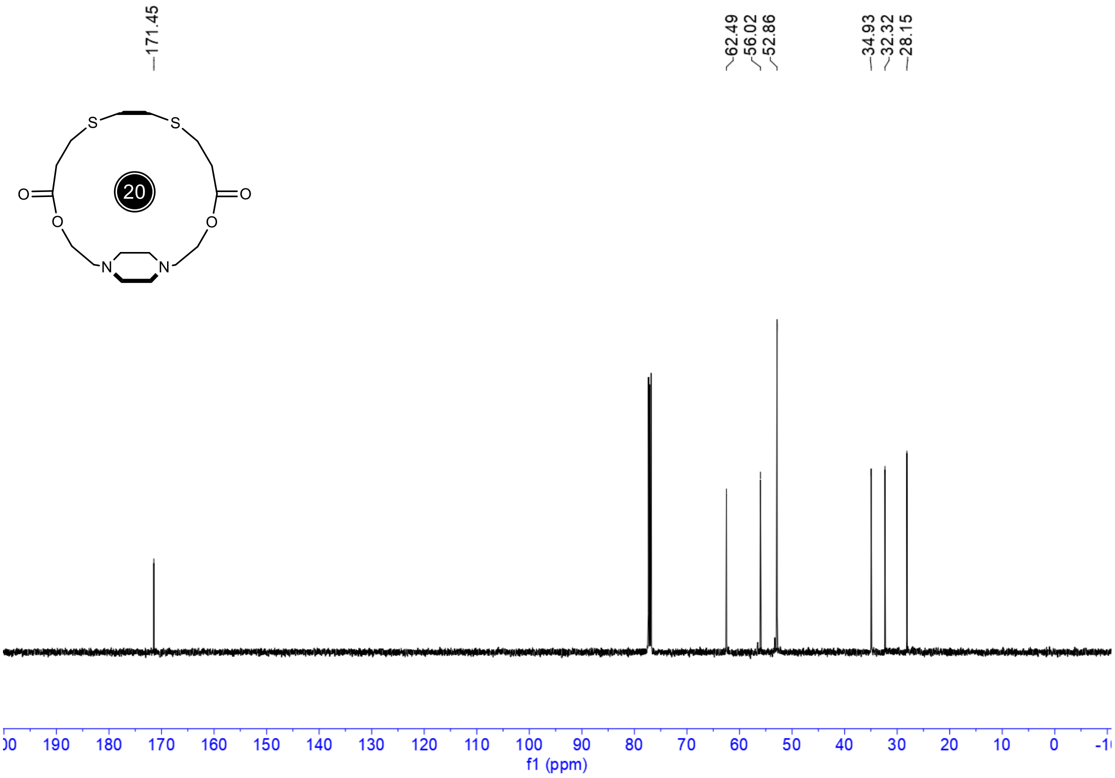

Supplementary Figure 232 | ^{1}H NMR (500 MHz, 298K, $CDCl_3$) of 2,3,5,6,7,8,9,10-Octahydro-12H-benzo[c][1]oxa[5,8]dithiacyclotetradecin-12-one **(33)**

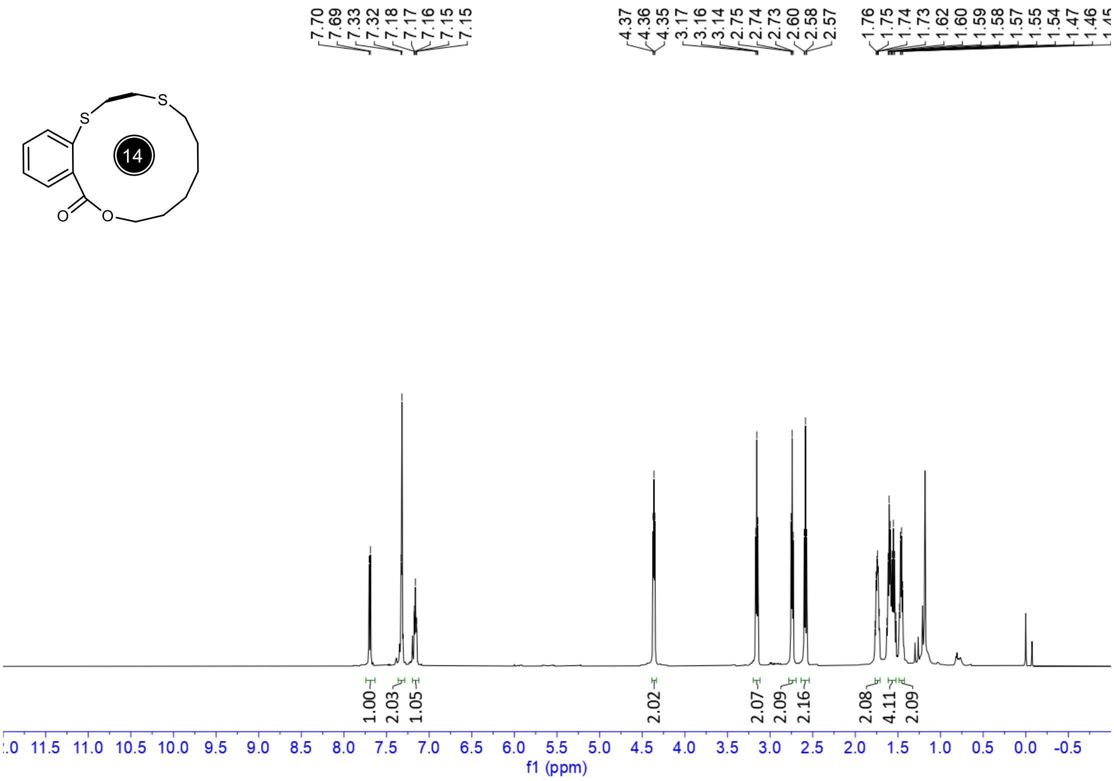

Supplementary Figure 233 | ^{13}C NMR (126 MHz, 298K, $CDCl_3$) of 2,3,5,6,7,8,9,10-Octahydro-12H-benzo[c][1]oxa[5,8]dithiacyclotetradecin-12-one **(33)**

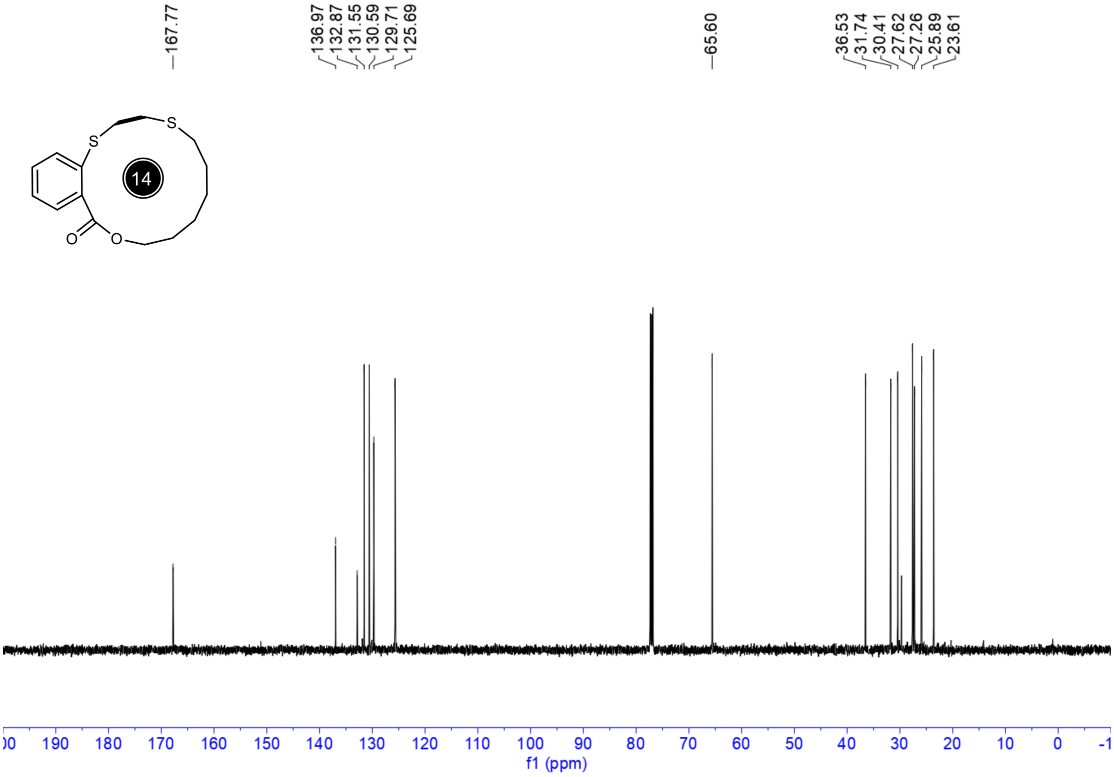

Supplementary Figure 234 | ^{1}H NMR (500 MHz, 298K, $CDCl_3$) of 1,4,5,7,8,10,11,14-Octahydro-3*H*,12*H*-benzo[*n*][1,12]dioxa[5,8]dithiacyclohexadecine-3,12-dione **(34)**

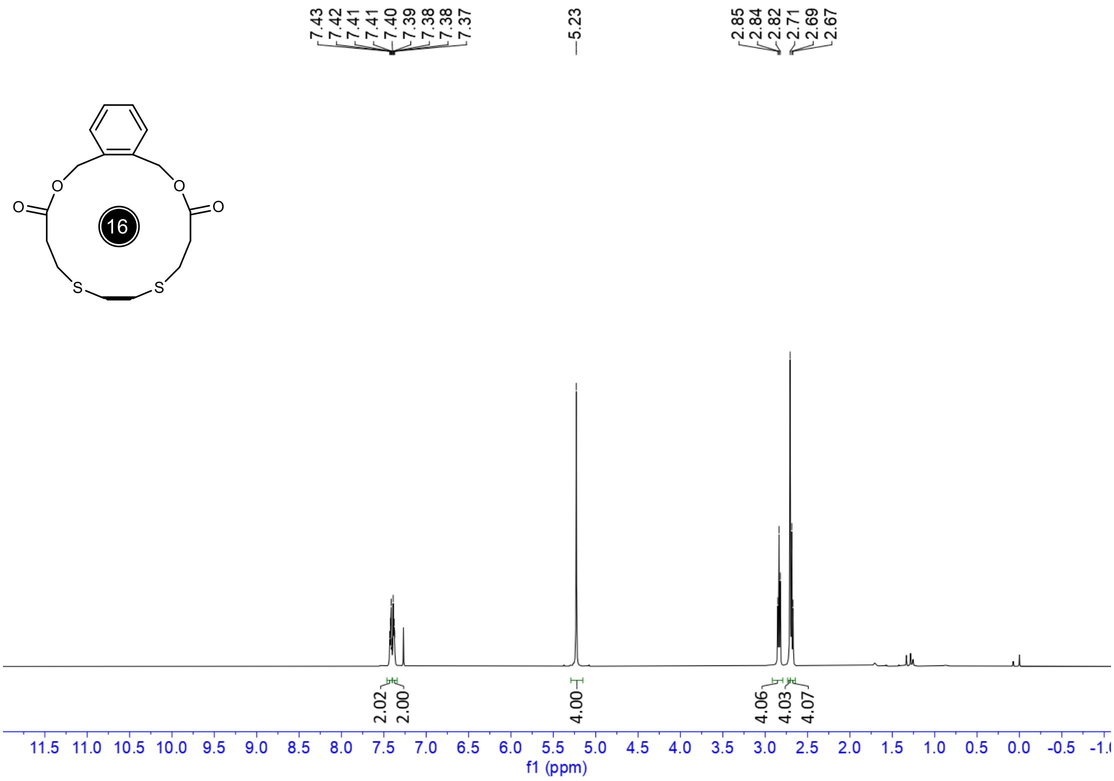

Supplementary Figure 235 | ^{13}C NMR (126 MHz, 298K, $CDCl_3$) of 1,4,5,7,8,10,11,14-Octahydro-3*H*,12*H*-benzo[*n*][1,12]dioxa[5,8]dithiacyclohexadecine-3,12-dione **(34)**

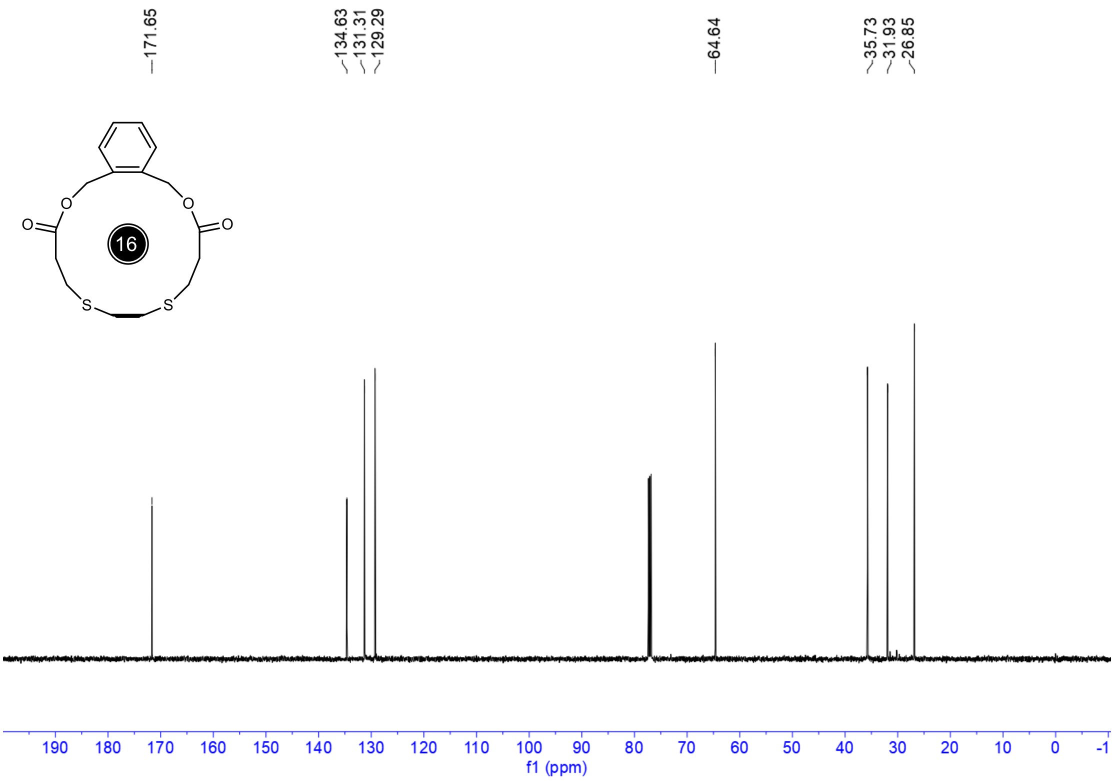

Supplementary Figure 236| ^{1}H NMR (500 MHz, 298K, $CDCl_3$) of 5,6,7,8,9,10,12,13,15,16,17,18,19,20Tetradecahydrobenzo[*e*][1,4,7,10]tetraoxa[17,20]dithiacyclohexacosine-3,22(2H,23H)-dione **(35)**

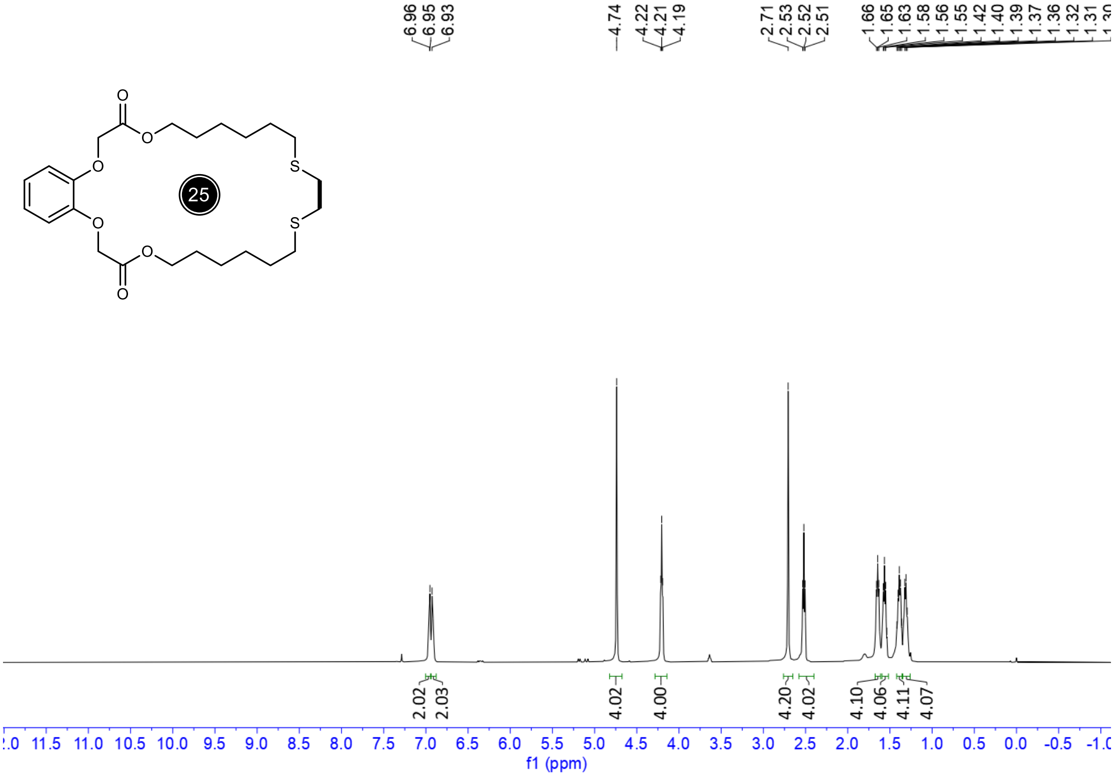

Supplementary Figure 237 | ^{13}C NMR (126 MHz, 298K, $CDCl_3$) of 5,6,7,8,9,10,12,13,15,16,17,18,19,20-Tetradecahydrobenzo[*e*][1,4,7,10]tetraoxa [17,20]dithiacyclohexacosine-3,22(2H,23H)-dione **(35)**

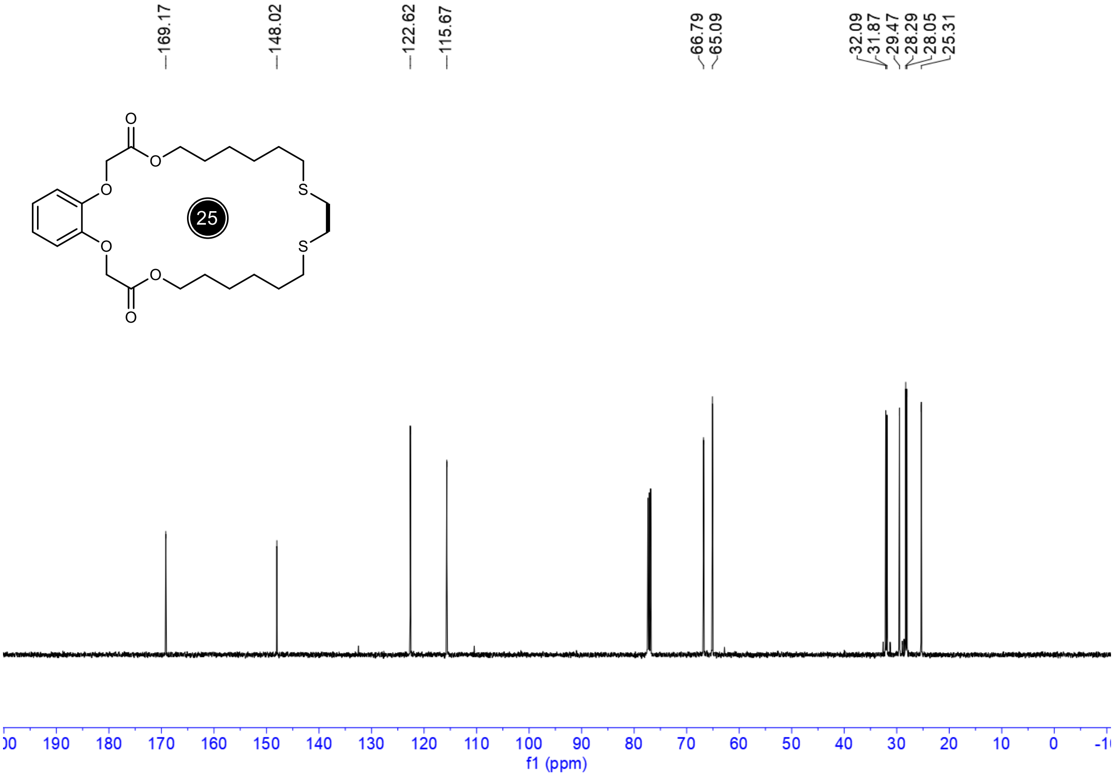

Supplementary Figure 238 | ^{1}H NMR (500 MHz, 298K, $CDCl_3$) of 3,14-Dioxa-7,10-dithia-1(1,3)-benzenacyclopentadecaphane-4,13-dione **(36)**

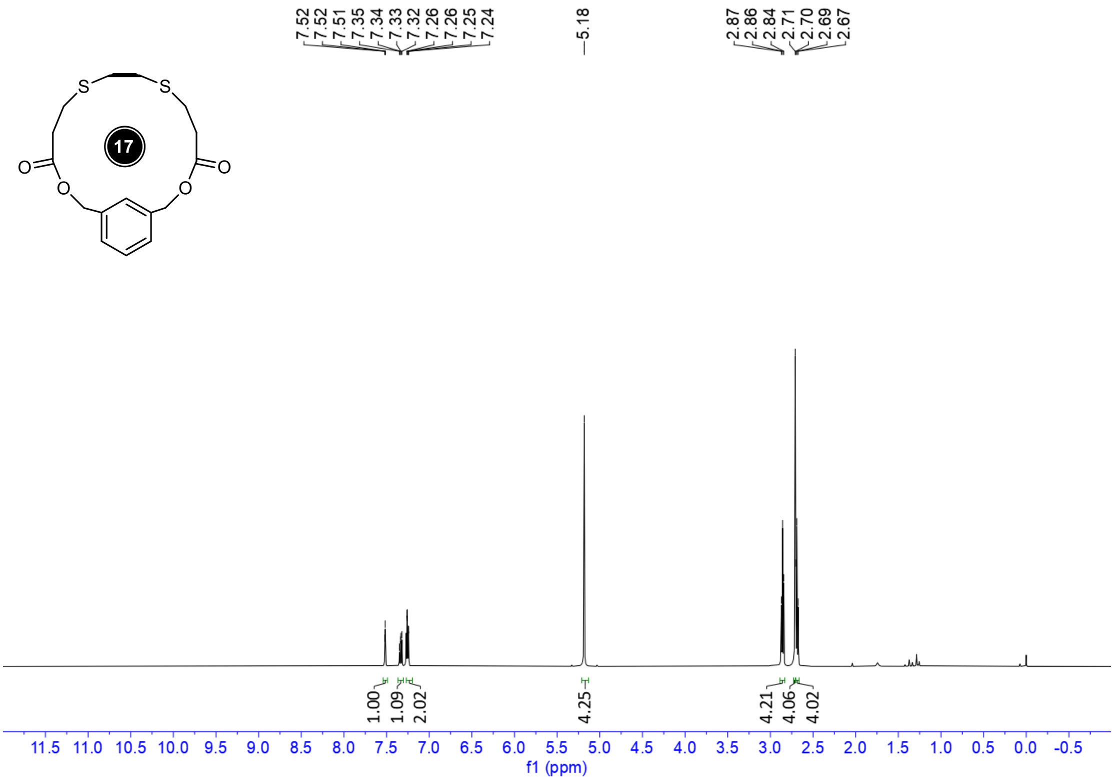

Supplementary Figure 239 | ^{13}C NMR (126 MHz, 298K, $CDCl_3$) of 3,14-Dioxa-7,10-dithia-1(1,3)-benzenacyclopentadecaphane-4,13-dione **(36)**

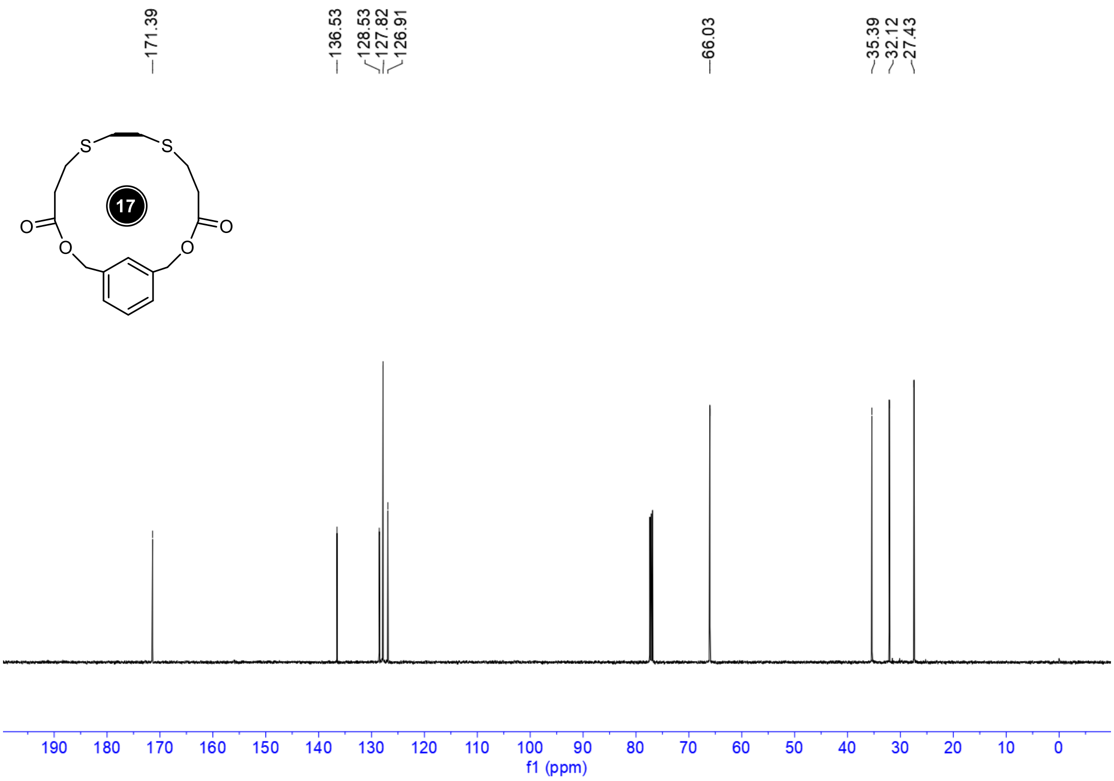

Supplementary Figure 240 | ^{1}H NMR (500 MHz, 298K, $CDCl_3$) of 3,14-Dioxa-7,10-dithia-1(1,4)-benzenacyclopentadecaphane-4,13-dione **(37)**

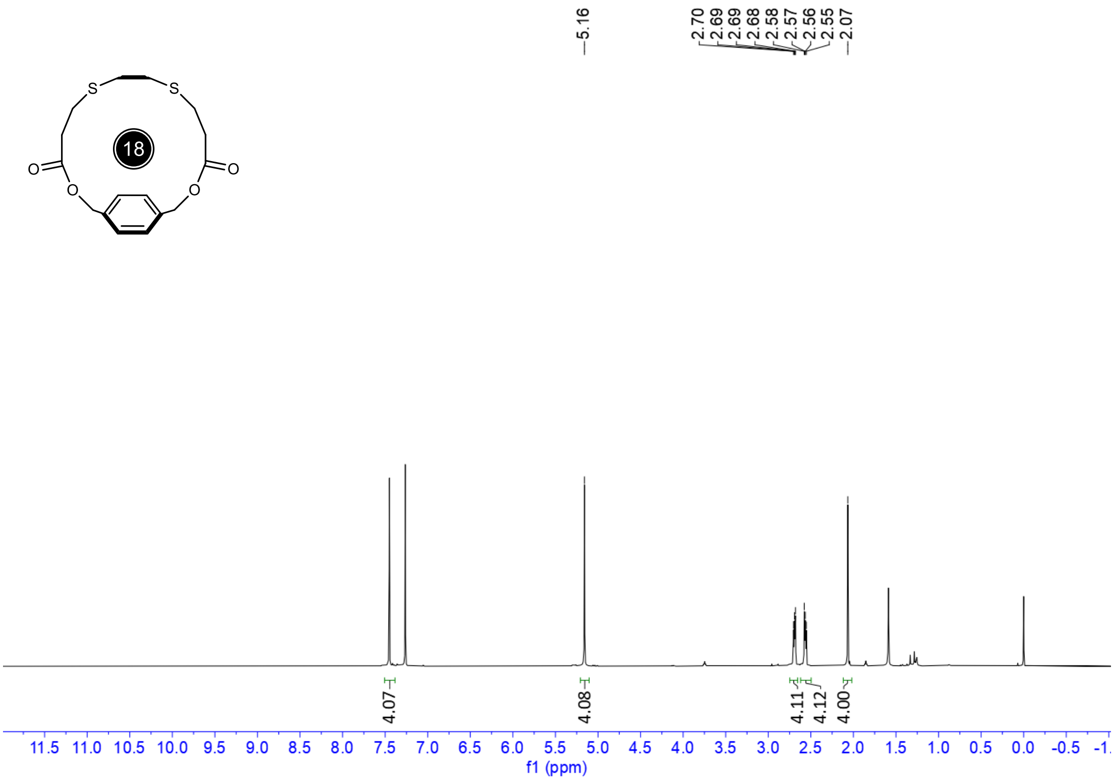

Supplementary Figure 241 | ^{13}C NMR (126 MHz, 298K, $CDCl_3$) of 3,14-Dioxa-7,10-dithia-1(1,4)-benzenacyclopentadecaphane-4,13-dione **(37)**

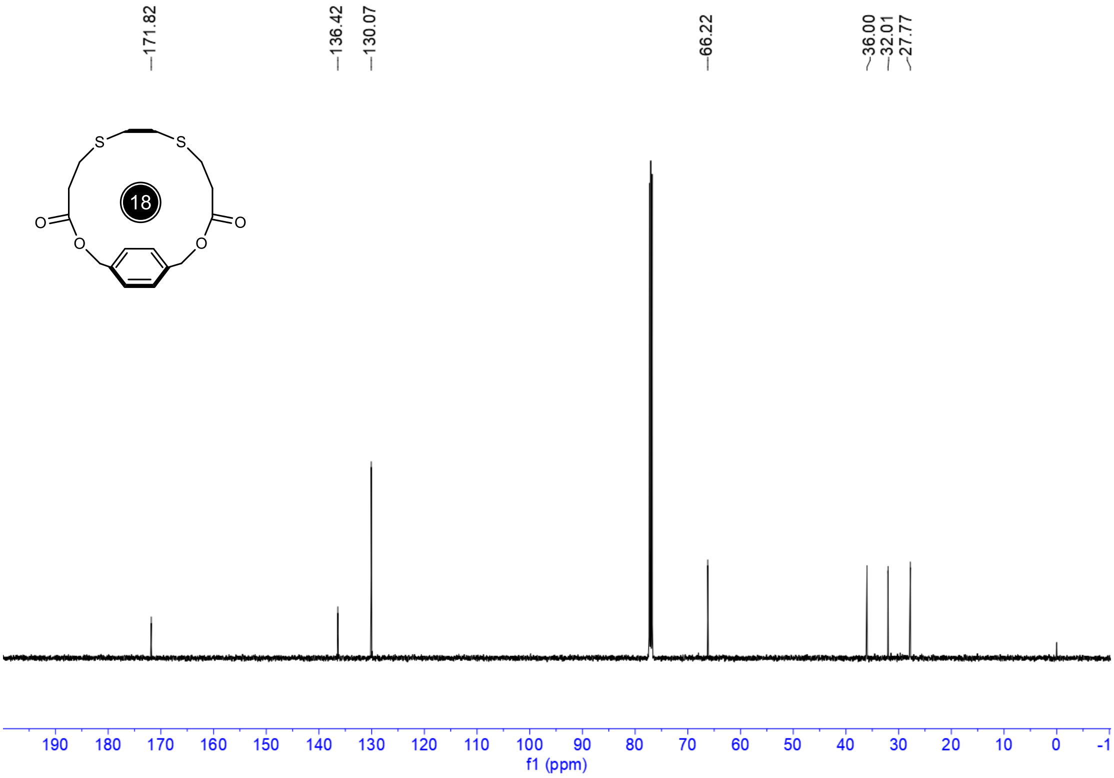

Supplementary Figure 242 | ^{1}H NMR (500 MHz, 298K, $CDCl_3$) of 2,5,16,19-Tetraoxa-9,12-dithia-1(1,4)-benzenacyclononadecaphane-6,15-dione **(38)**

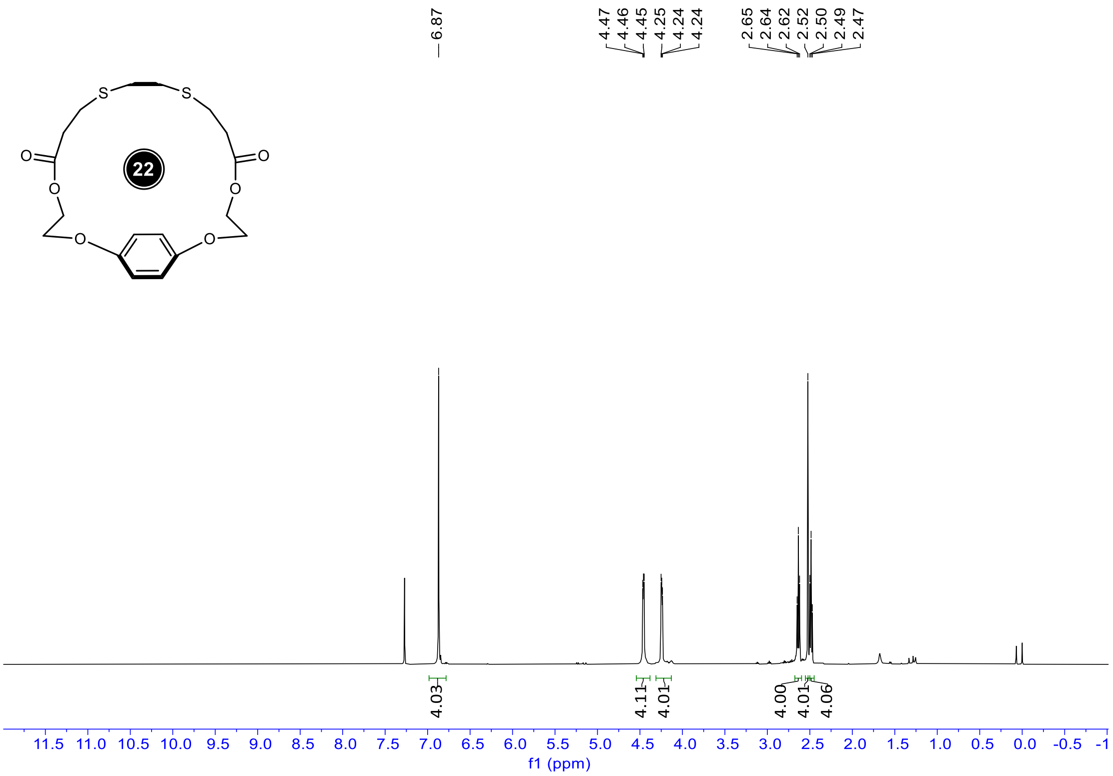

Supplementary Figure 243 | ^{13}C NMR (126 MHz, 298K, $CDCl_3$) of 2,5,16,19-Tetraoxa-9,12-dithia-1(1,4)-benzenacyclononadecaphane-6,15-dione **(38)**

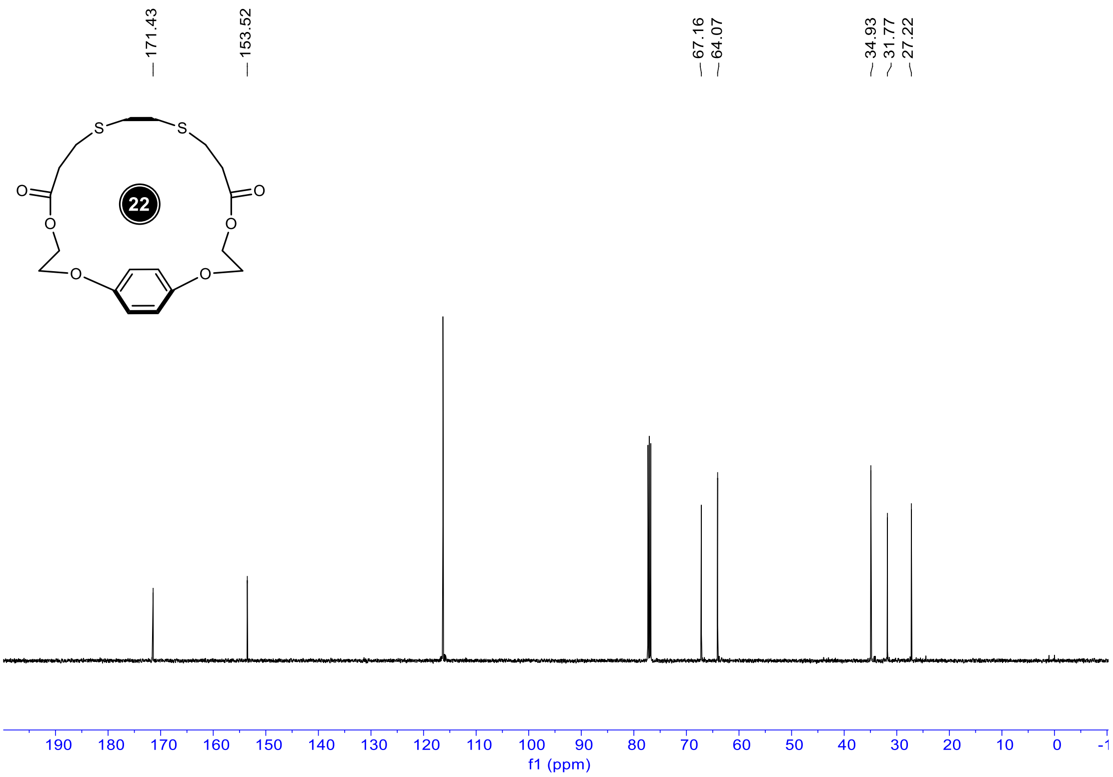

Supplementary Figure 244 | ^{1}H NMR (500 MHz, 298K, $CDCl_3$) of 2,6,17,21-Tetraoxa-10,13-dithia-1(2,7)-naphthalenacyclohenicosaphane-7,16-dione **(39)**

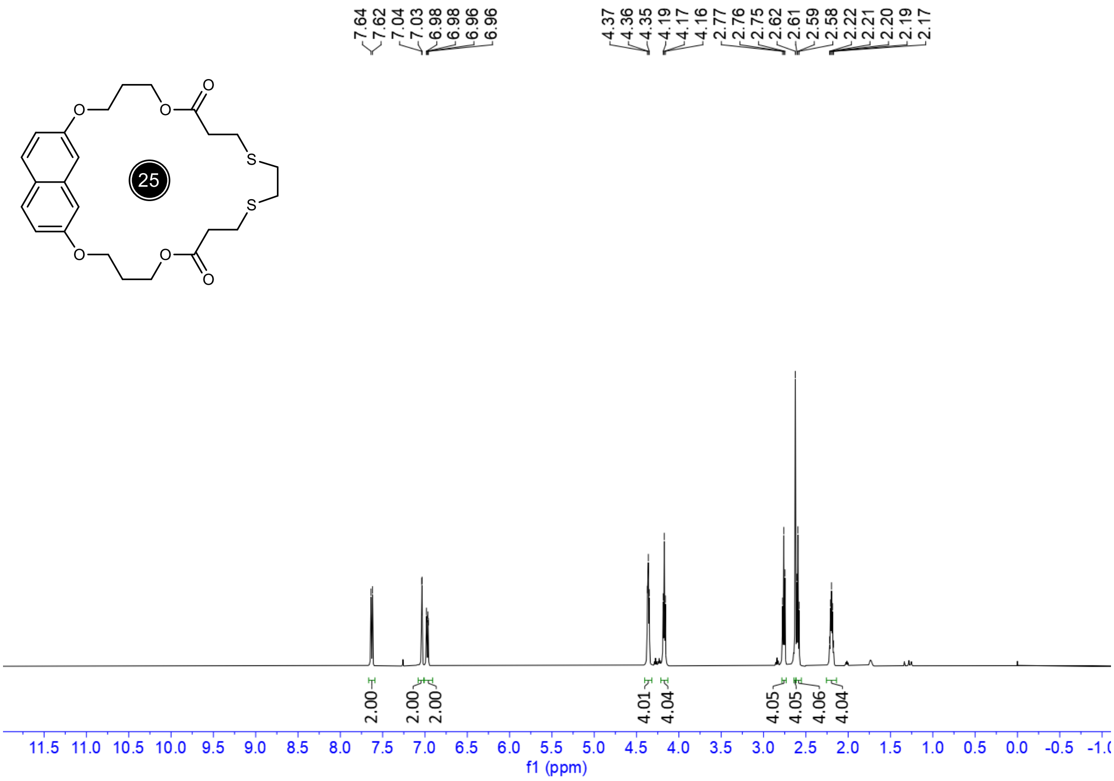

Supplementary Figure 245 | ^{13}C NMR (126 MHz, 298K, $CDCl_3$) of 2,6,17,21-Tetraoxa-10,13-dithia-1(2,7)-naphthalenacyclohenicosaphane-7,16-dione **(39)**

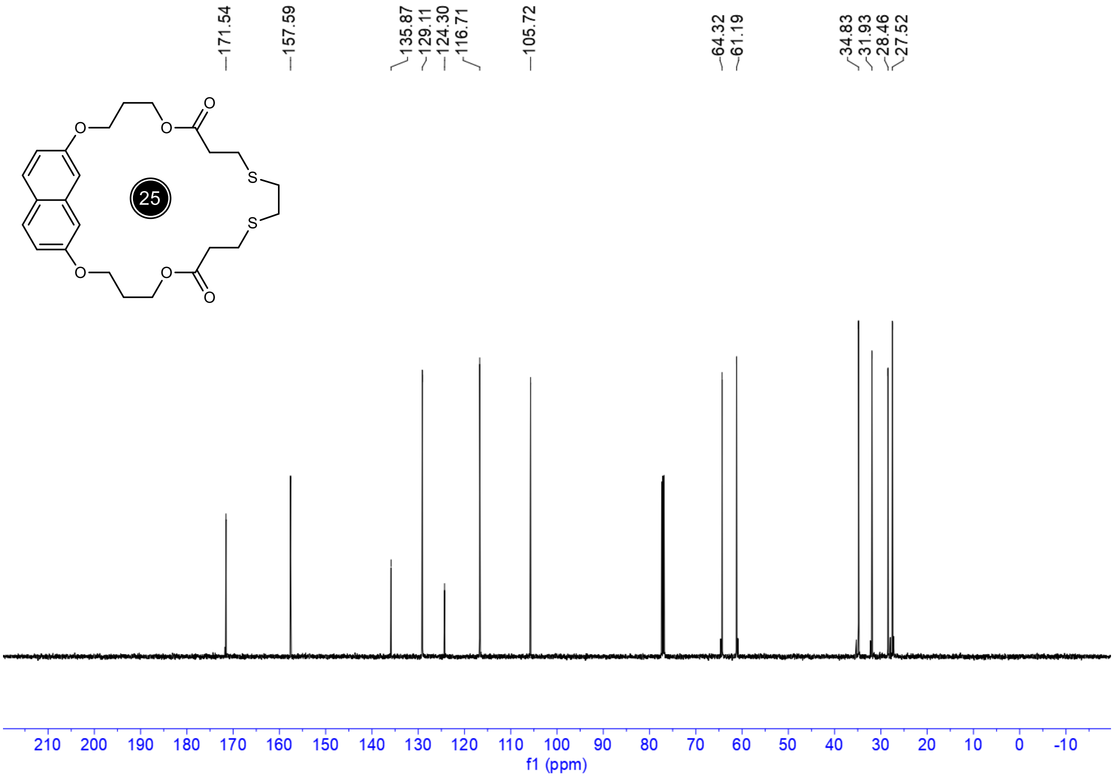

Supplementary Figure 246 | ^{1}H NMR (500 MHz, 298K, $CDCl_3$) of 3,14-Dioxa-7,10-dithia-1(2,6)-pyridinacyclopentadecaphane-4,13-dione **(40)**

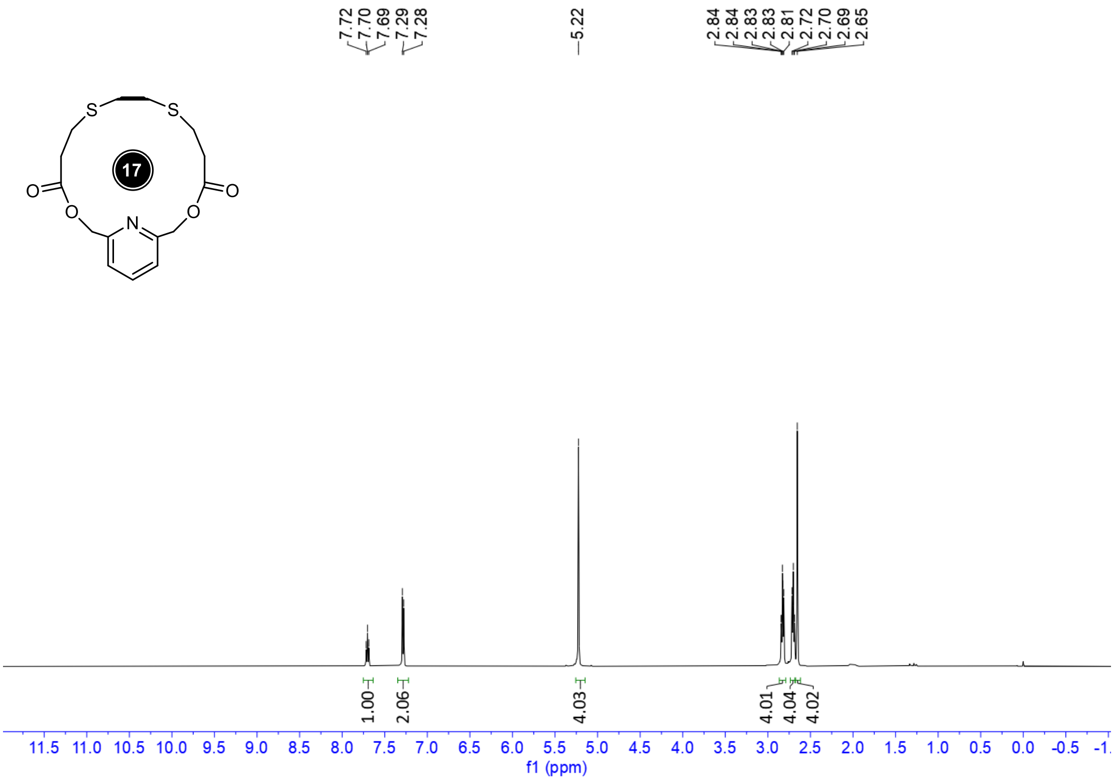

Supplementary Figure 247 | ^{13}C NMR (126 MHz, 298K, $CDCl_3$) of 3,14-Dioxa-7,10-dithia-1(2,6)-pyridinacyclopentadecaphane-4,13-dione **(40)**

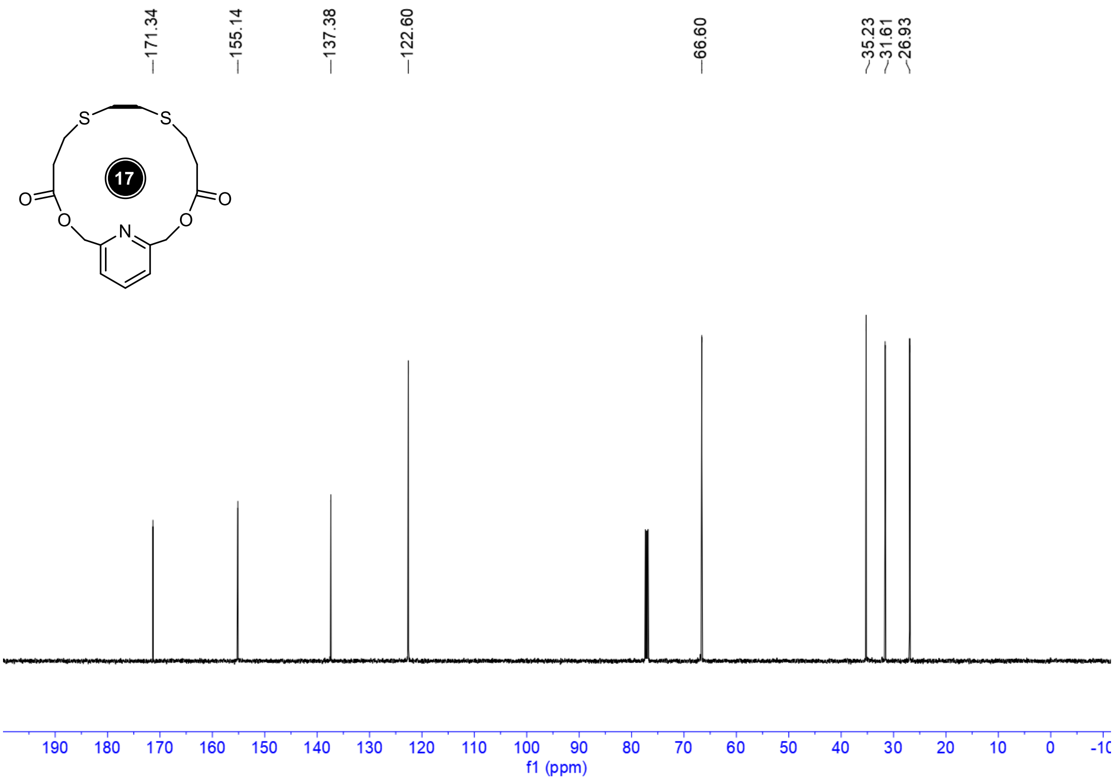

Supplementary Figure 248 | ^{1}H NMR (500 MHz, 298K, $CDCl_3$) of 3,14-Dioxa-7,10-dithia-1(2,5)-furanacyclopentadecaphane-4,13-dione **(41)**

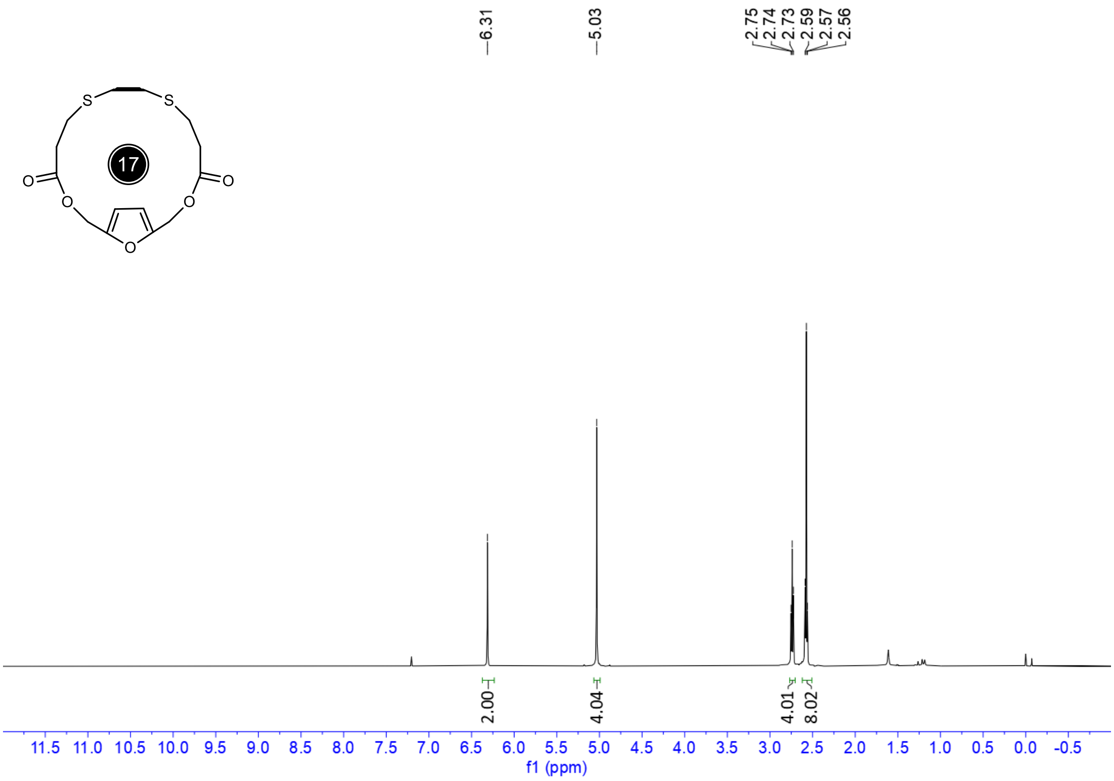

Supplementary Figure 249 | ^{13}C NMR (126 MHz, 298K, $CDCl_3$) of 3,14-Dioxa-7,10-dithia-1(2,5)-furanacyclopentadecaphane-4,13-dione **(41)**

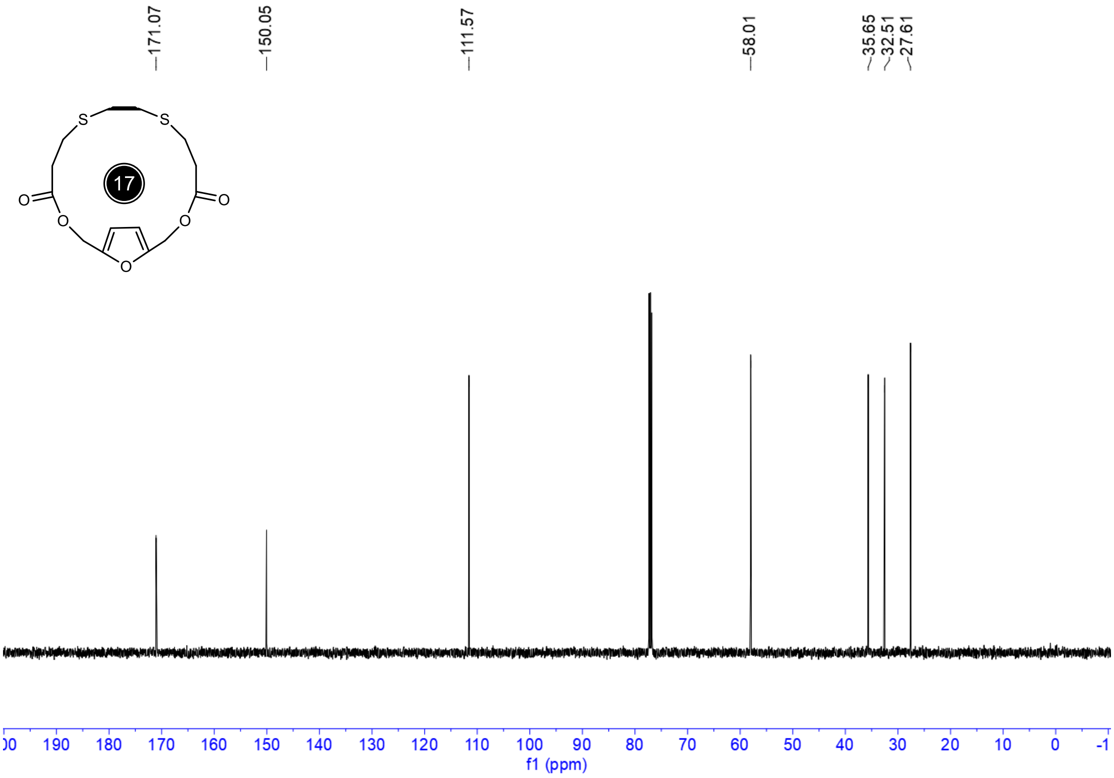

Supplementary Figure 250 | ^{1}H NMR (500 MHz, 298K, $CDCl_3$) of 3,20-Dioxa-10,13-dithiatricyclo phthalate-2,21-dione **(42)**

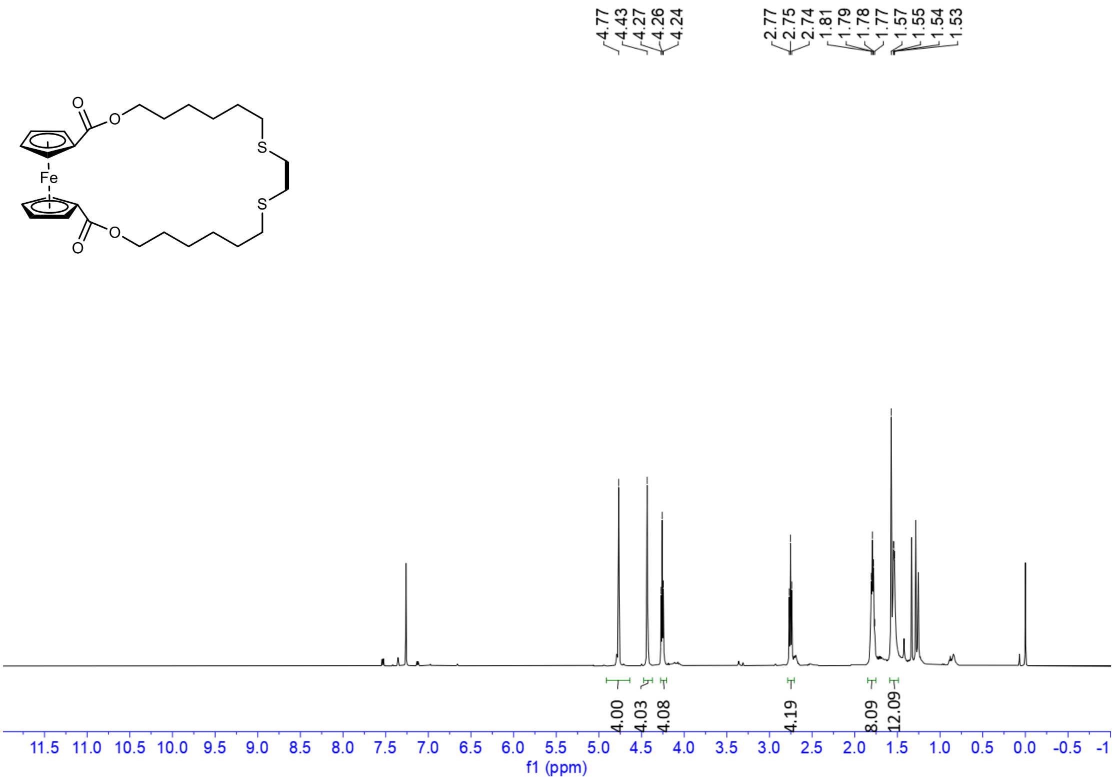

Supplementary Figure 251 | ^{13}C NMR (126 MHz, 298K, $CDCl_3$) of 3,20-Dioxa-10,13-dithiatricyclo phthalate-2,21-dione **(42)**

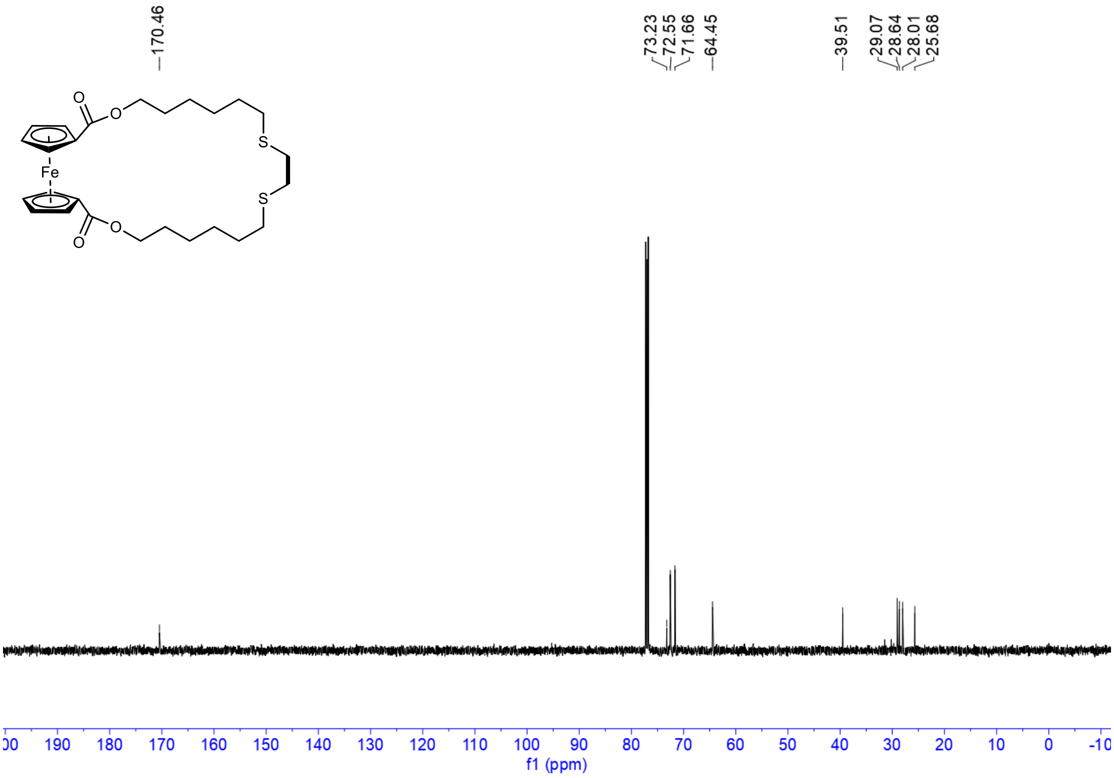

Supplementary Figure 252 | ^{1}H NMR (500 MHz, 298K, $CDCl_3$) of (*R*)-9,10,12,13,16,17-Hexahydro-8*H*,15*H*-dinaphtho[2,1-m:1',2'*o*][1,12]dioxa[5,8]dithiacyclohexadecine **(43)**

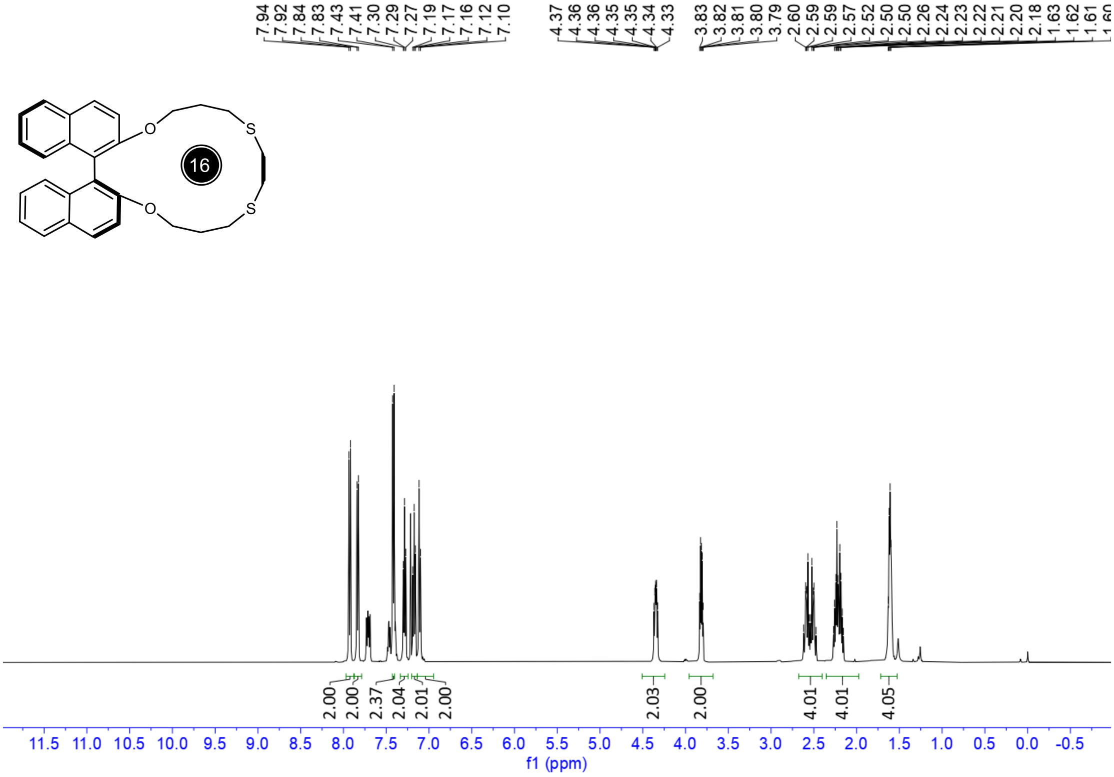

Supplementary Figure 253 | ^{13}C NMR (126 MHz, 298K, $CDCl_3$) of (*R*)-9,10,12,13,16,17-Hexahydro-8*H*,15*H*-dinaphtho[2,1-m:1',2'*o*][1,12]dioxa[5,8]dithiacyclohexadecine **(43)**

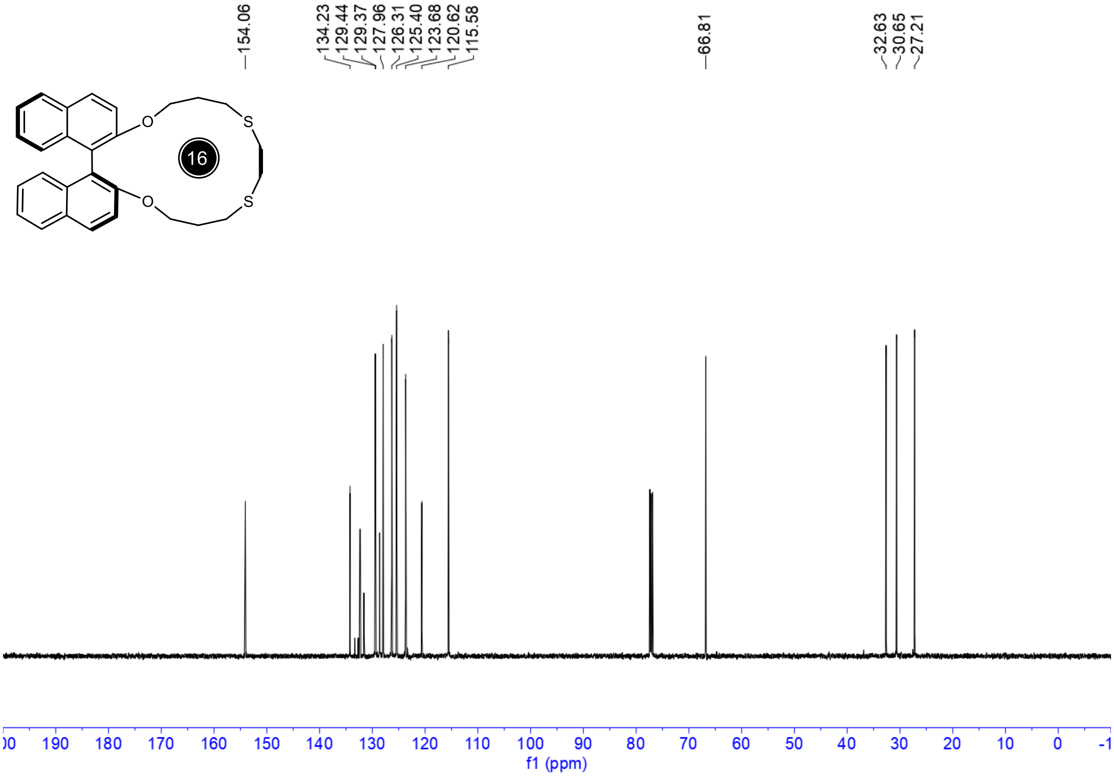

Supplementary Figure 254 | ^{1}H NMR (500 MHz, 298K, $CDCl_3$) of (*R*)-12,13,14,15,16,17,19,20,22,23,24,25,26,27-Tetradecahydrodinaphtho[2,1-b:1',2'*d*][1,6]dioxa[13,16]dithiacyclodocosine **(44)**

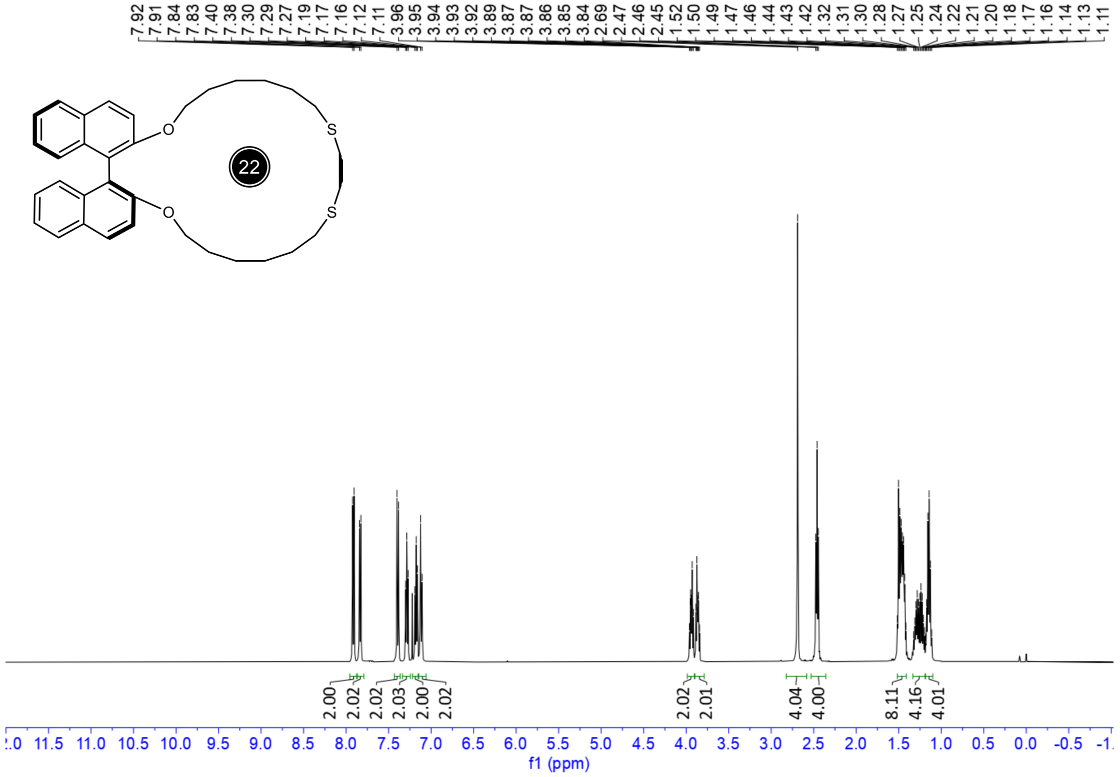

Supplementary Figure 255 | ^{13}C NMR (126 MHz, 298K, $CDCl_3$) of (*R*)-12,13,14,15,16,17,19,20,22,23,24,25,26,27-Tetradecahydrodinaphtho[2,1-b:1',2'-*d*][1,6]dioxa[13,16]dithiacyclodocosine **(44)**

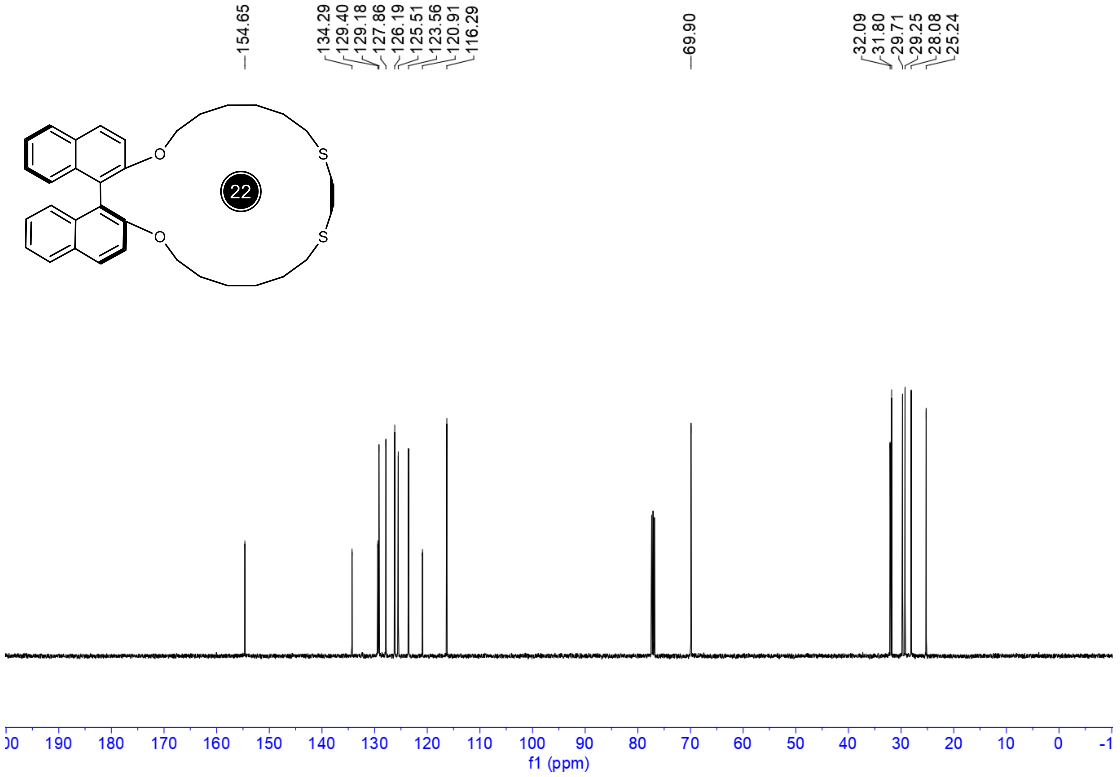

Supplementary Figure 256 | ^{1}H NMR (500 MHz, 298K, $CDCl_3$) of (*R*)-9,10,13,14,16,17,19,20,24,25-Decahydro-8*H*,12*H*,21*H*,23*H*-dinaphtho[2,1-*u*:1',2'*w*][1,5,16,20]tetraoxa[9,12]dithiacyclotetracosine-12,21-dione **(45)**

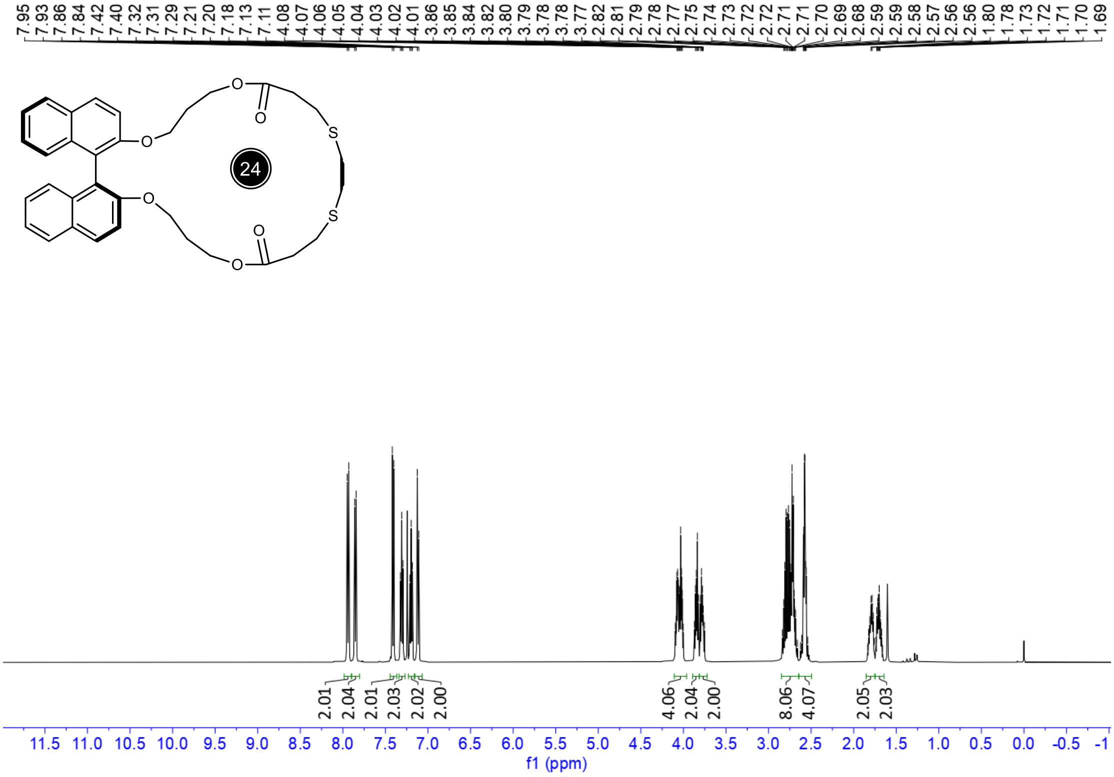

Supplementary Figure 257 | ^{13}C NMR (126 MHz, 298K, $CDCl_3$) of (*R*)-9,10,13,14,16,17,19,20,24,25-Decahydro-8*H*,12*H*,21*H*,23*H*-dinaphtho[2,1-*u*:1',2'*w*][1,5,16,20]tetraoxa[9,12]dithiacyclotetracosine-12,21-dione **(45)**

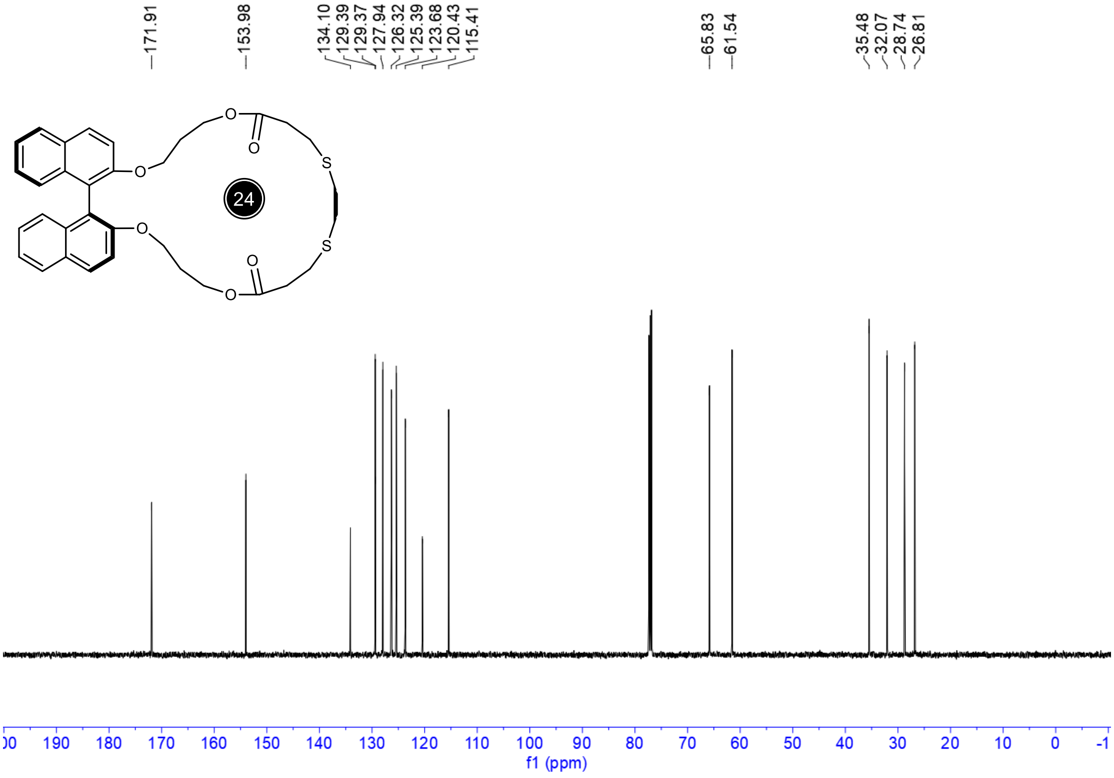

Supplementary Figure 258 | ^{1}H NMR (500 MHz, 298K, $CDCl_3$) of (*R*)-8,9,10,11,12,13,14,15,17,18,20,21,22,23,24,25,26,27-Octadecahydrodinaphtho[2,1-b:1',2'-*d*][1,6]dioxa[15,18]dithiacyclohexacosine **(46)**

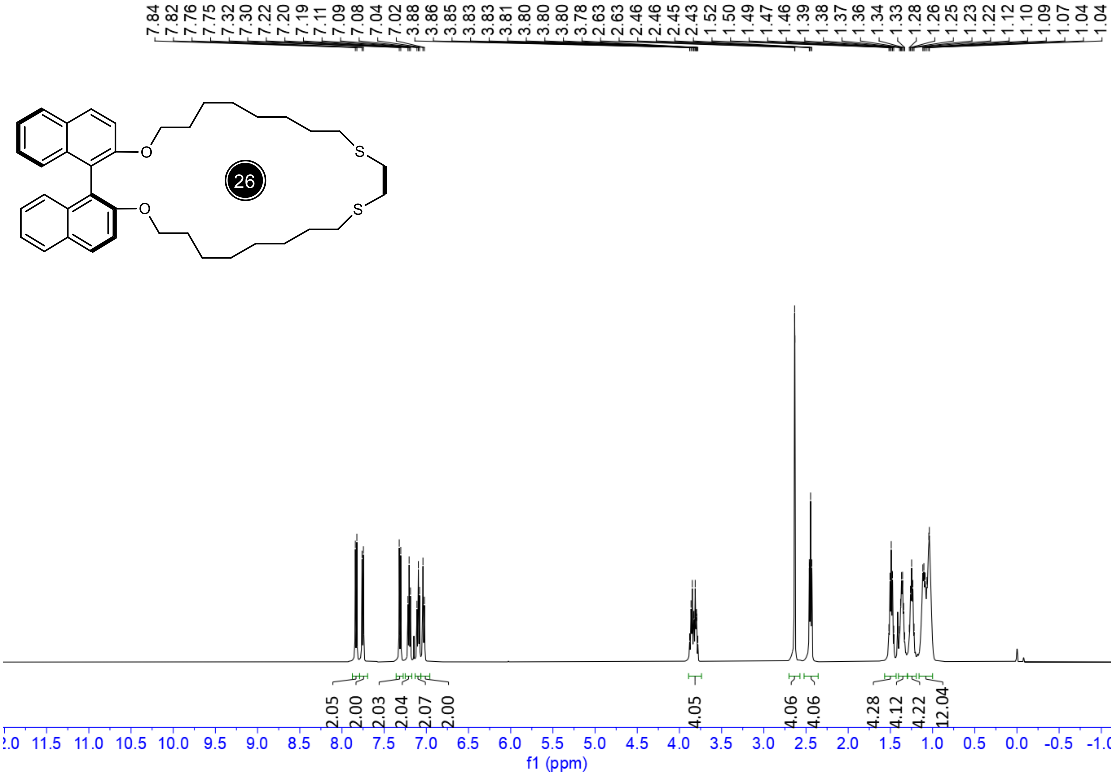

Supplementary Figure 259 | ^{13}C NMR (126 MHz, 298K, $CDCl_3$) of (*R*)-8,9,10,11,12,13,14,15,17,18,20,21,22,23,24,25,26,27-Octadecahydrodinaphtho[2,1-b:1',2'*d*][1,6] dioxa[15,18]dithiacyclohexacosine **(46)**

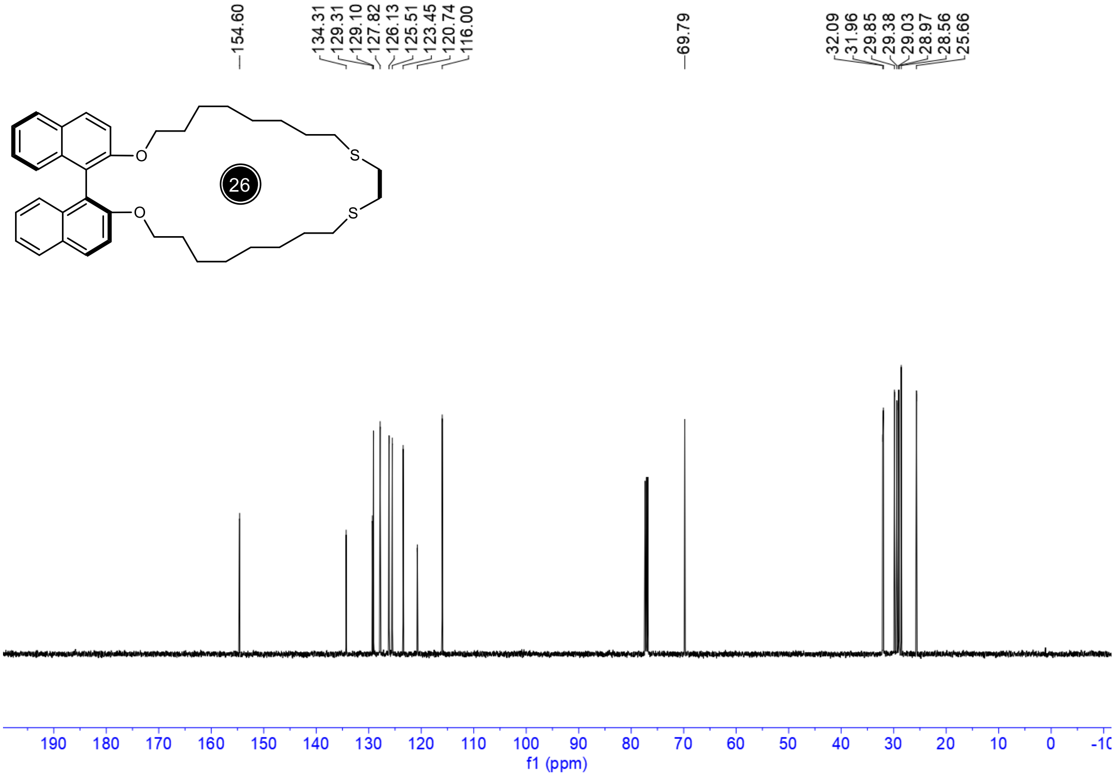

Supplementary Figure 260 | ^{1}H NMR (500 MHz, 298K, $CDCl_3$) of (*R*)-9,10,11,12,15,16,18,19,21,22,26,27,28,29-Tetradecahydro-8*H*,14*H*,23*H*,25*H*-dinaphtho[2,1-*s*:1',2'*u*][1,12,18,23]tetraoxa[5,8]dithiacyclooctacosine-14,23-dione **(47)**

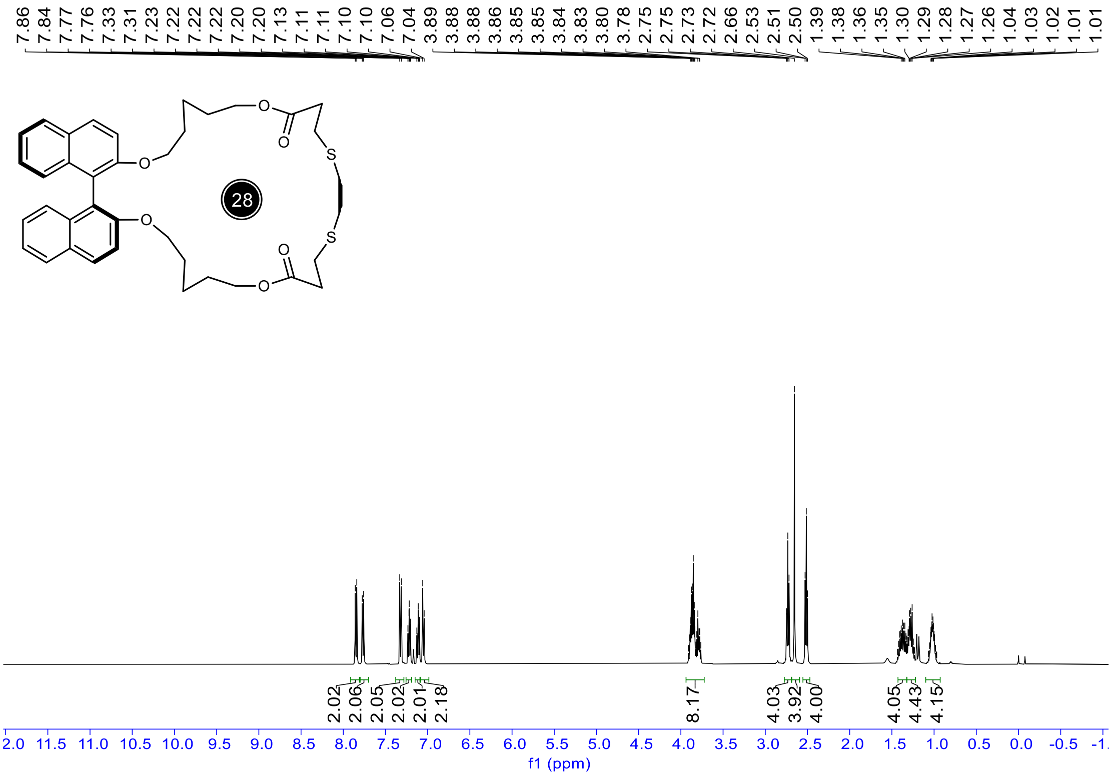

Supplementary Figure 261 | ^{13}C NMR (126 MHz, 298K, $CDCl_3$) of (*R*)-9,10,11,12,15,16,18,19,21,22,26,27,28,29-Tetradecahydro-8*H*,14*H*,23*H*,25*H*-dinaphtho[2,1-*s*:1',2'*u*][1,12,18,23]tetraoxa[5,8]dithiacyclooctacosine-14,23-dione **(47)**

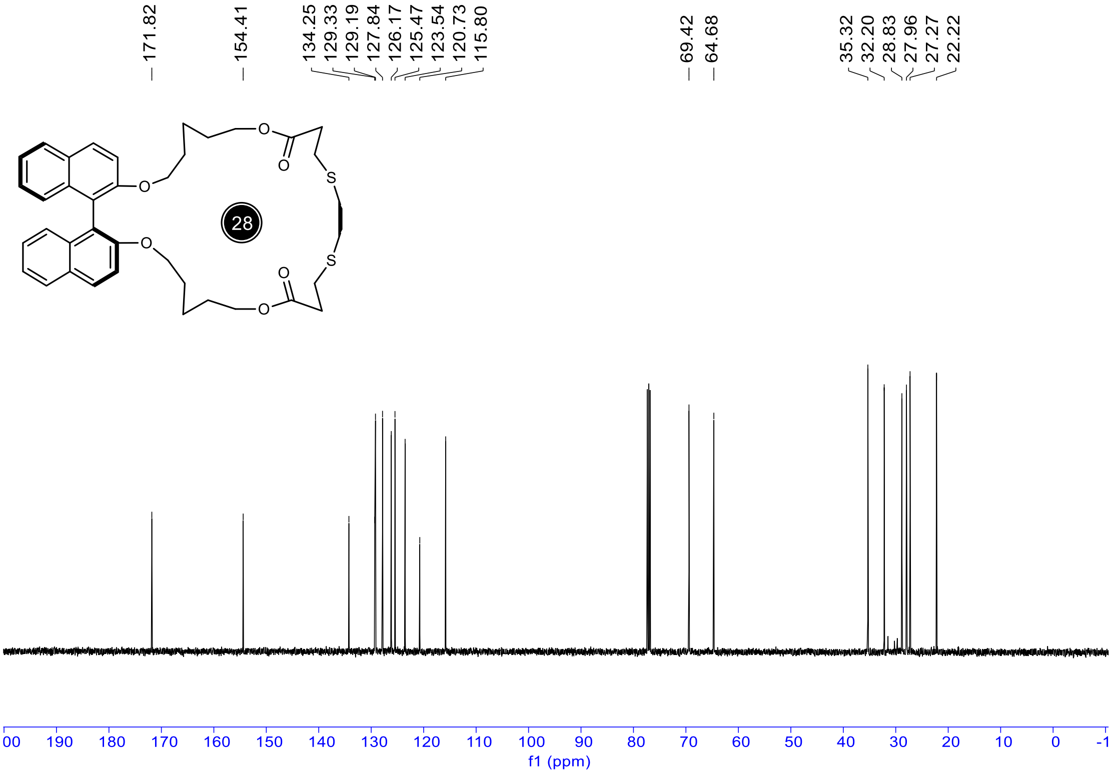

Supplementary Figure 262 | ^{1}H NMR (500 MHz, 298K, $CDCl_3$) of (*R*)-8,9,10,11,12,13,16,17,19,20,22,23,26,27,28,29,30,31-Octadecahydro-15*H*,24*H*-dinaphtho[2,1-t:1',2'-*v*][1,12,19,24]tetraoxa[5,8]dithiacyclotriacontine-15,24-dione **(48)**

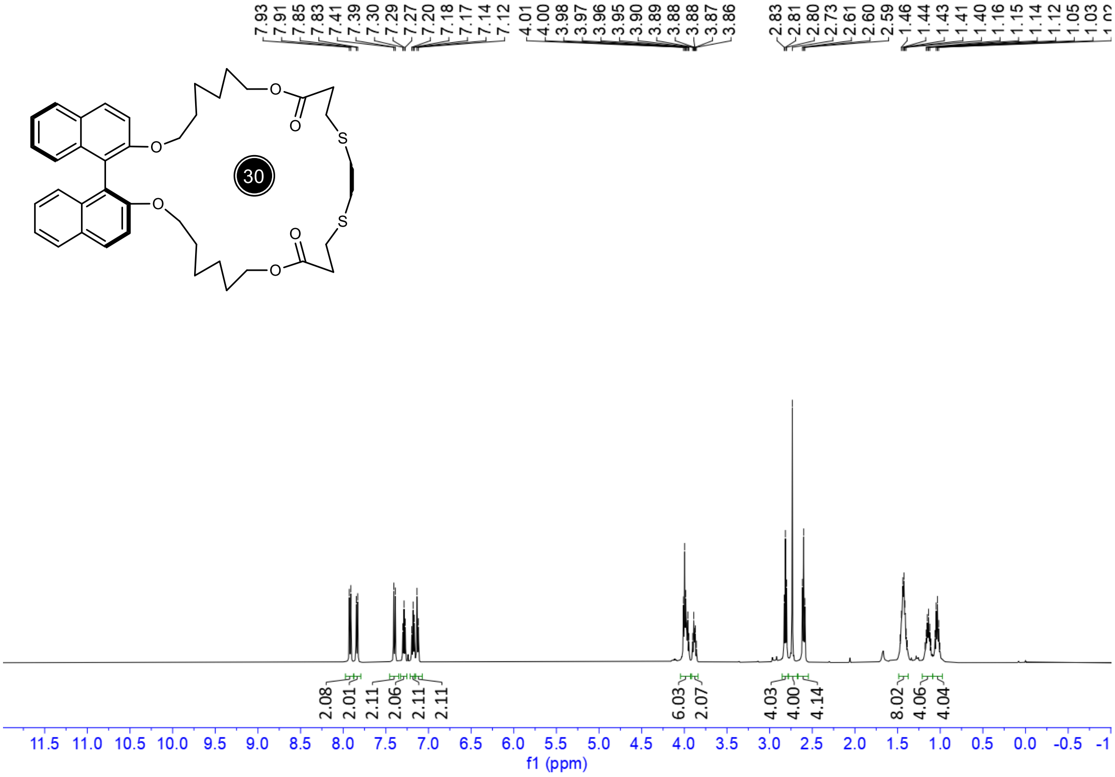

Supplementary Figure 263 | ^{13}C NMR (126 MHz, 298K, $CDCl_3$) of (*R*)-8,9,10,11,12,13,16,17,19,20,22,23,26,27,28,29,30,31-Octadecahydro-15*H*,24*H*-dinaphtho[2,1-t:1',2'-*v*][1,12,19,24]tetraoxa[5,8]dithiacyclotriacontine-15,24-dione **(48)**

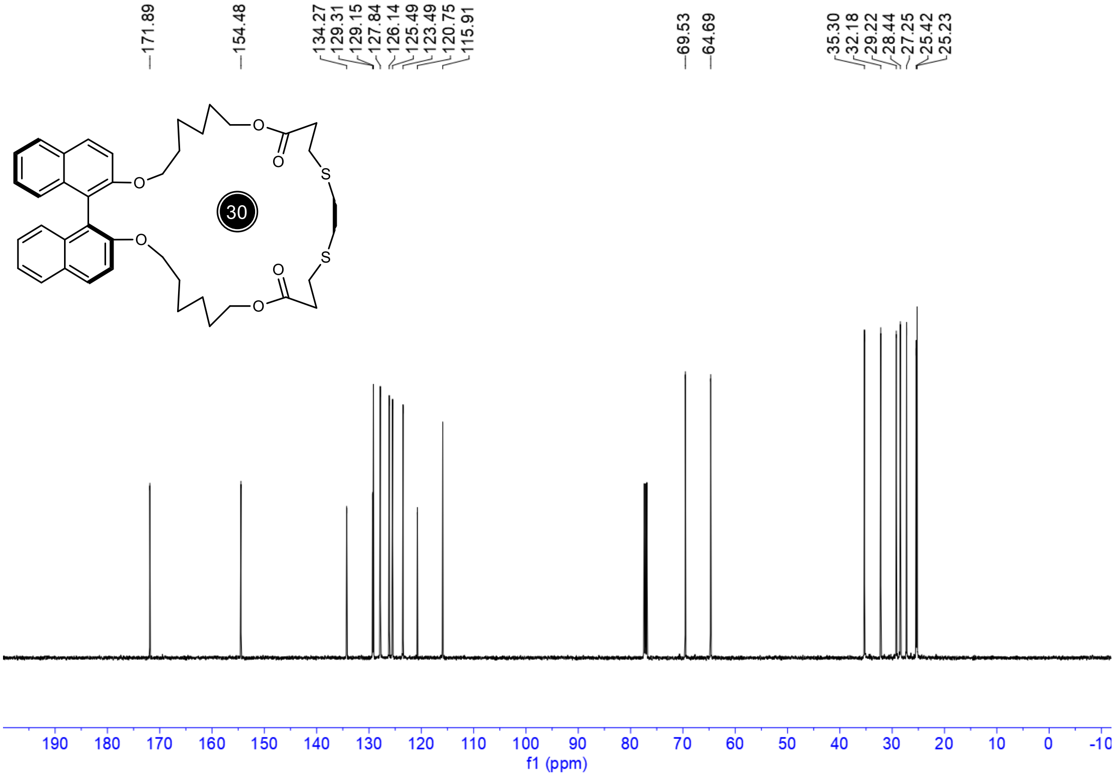

Supplementary Figure 264 | ^{1}H NMR (500 MHz, 298K, $CDCl_3$) of (12a*R*,16a*R*)-Dodecahydro-2*H*,11*H*-benzo[*b*][1,4]dioxa[8,11]dithiacyclotetradecine-2,11-dione **(49)**

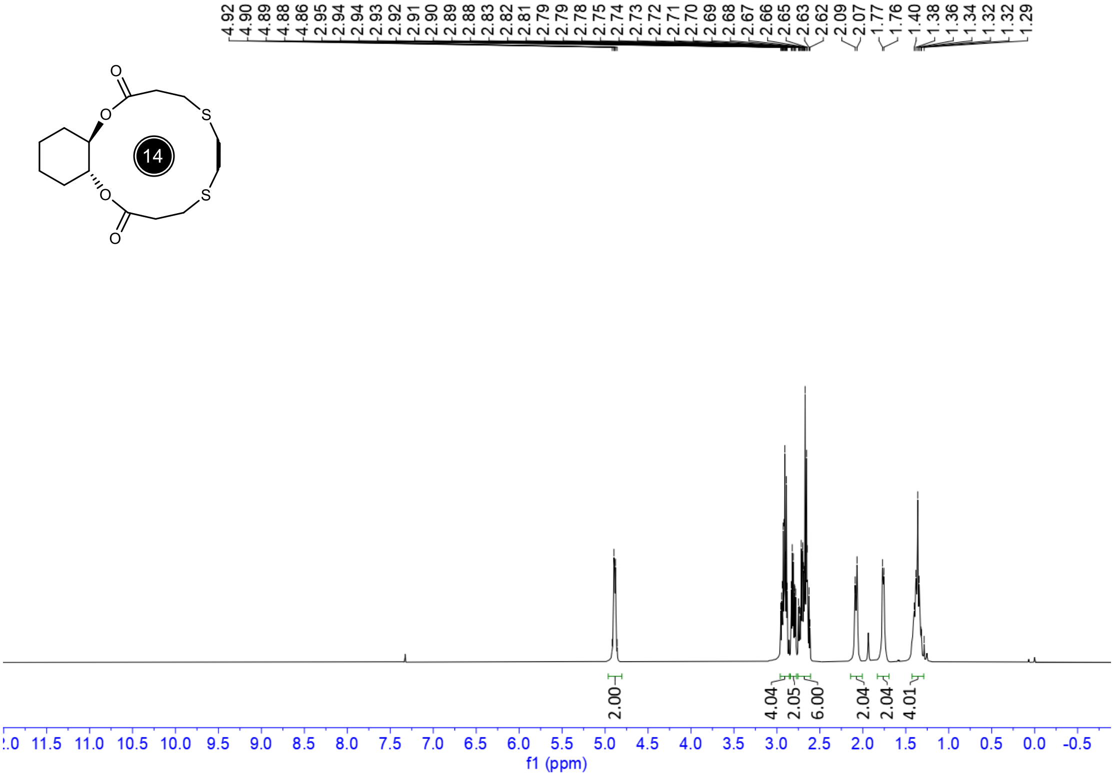

Supplementary Figure 265 | ^{13}C NMR (126 MHz, 298K, $CDCl_3$) of (12a*R*,16a*R*)-Dodecahydro-2*H*,11*H*-benzo[*b*][1,4]dioxa[8,11]dithiacyclotetradecine-2,11-dione **(49)**

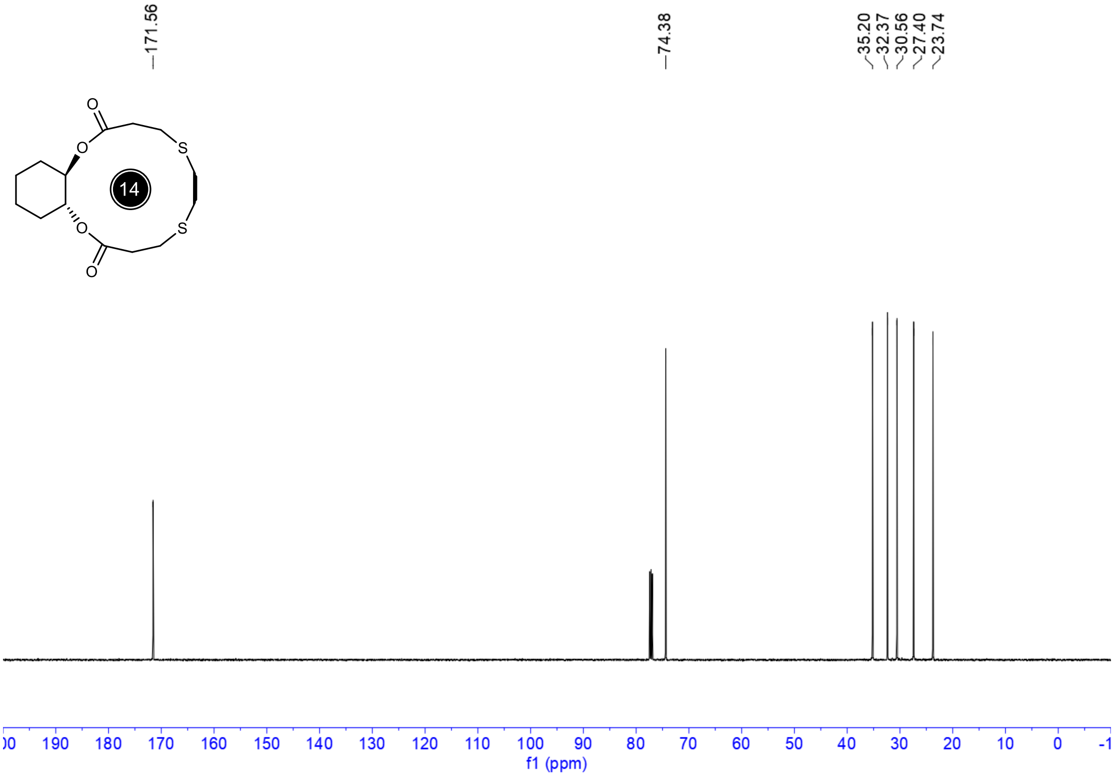

Supplementary Figure 266 | ^{1}H NMR (500 MHz, 298K, $CDCl_3$) of (*S*)-6-Methyl-1-oxa-7,10-dithia-4-azacyclohexadecane-2,5-dione **(50)**

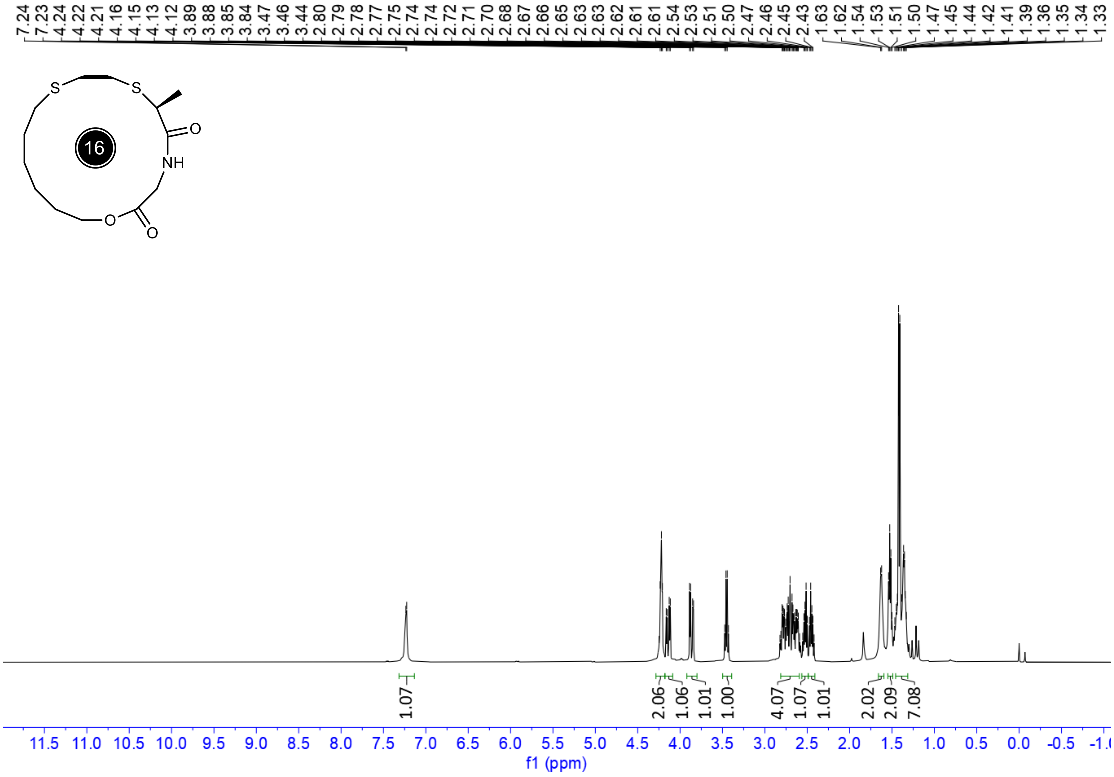

Supplementary Figure 267 | ^{13}C NMR (126 MHz, 298K, $CDCl_3$) of **(***S***)**-6-Methyl-1-oxa-7,10-dithia-4-azacyclohexadecane-2,5-dione **(50)**

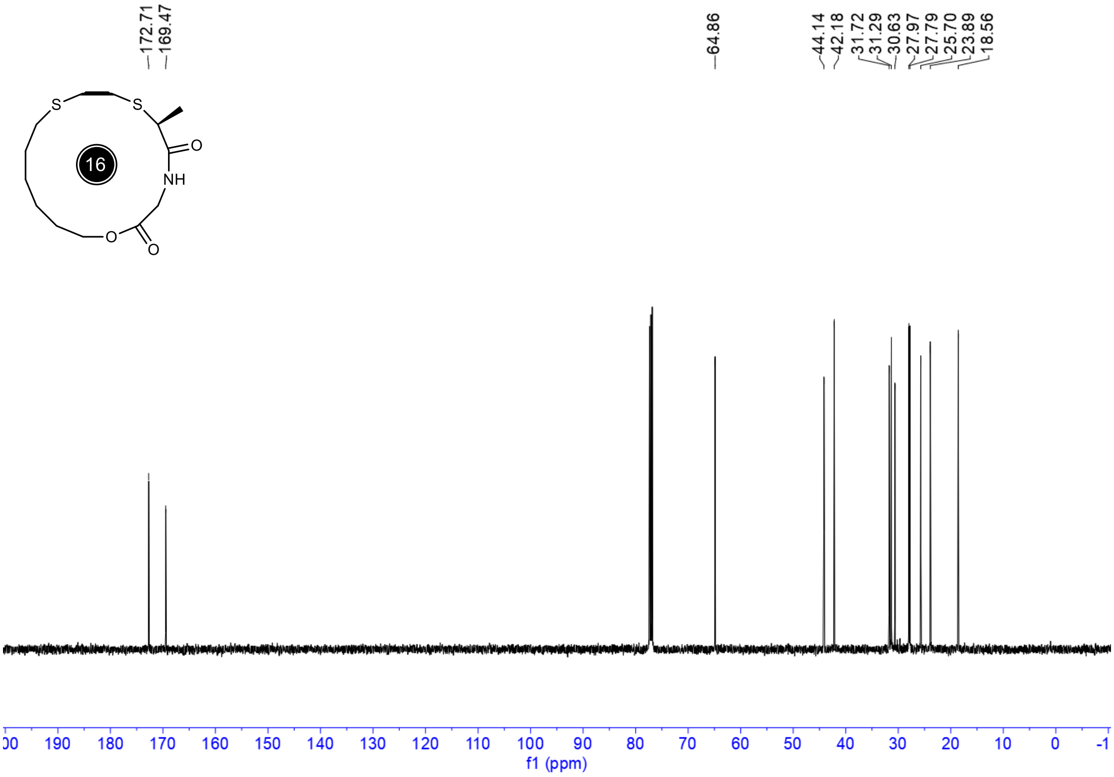

Supplementary Figure 268 | ^{1}H NMR (500 MHz, 298K, $CDCl_3$) of 1,2,7,8,9,10,11,12,15,16,18,19,21,22,25,26,27,28,29,30,35,36-Docosahydro-14*H*,23*H*-diindeno[7,1-*tu*:1',7'-*vw*][1,12,19,25]tetraoxa[5,8]dithiacyclohentriacontine-14,23-dione **(51)**

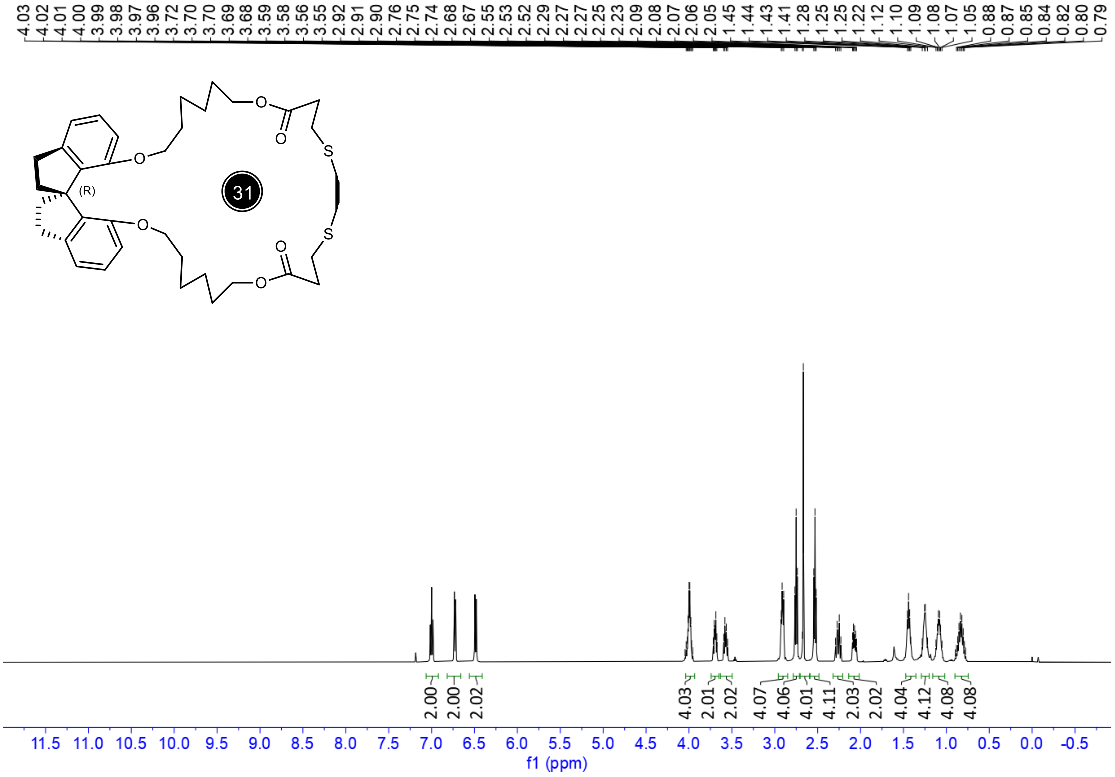

Supplementary Figure 269 | ^{13}C NMR (126 MHz, 298K, $CDCl_3$) of 1,2,7,8,9,10,11,12,15,16,18,19,21,22,25,26,27,28,29,30,35,36-Docosahydro-14*H*,23*H*-diindeno[7,1-*tu*:1',7'-*vw*][1,12,19,25]tetraoxa[5,8]dithiacyclohentriacontine-14,23-dione **(51)**

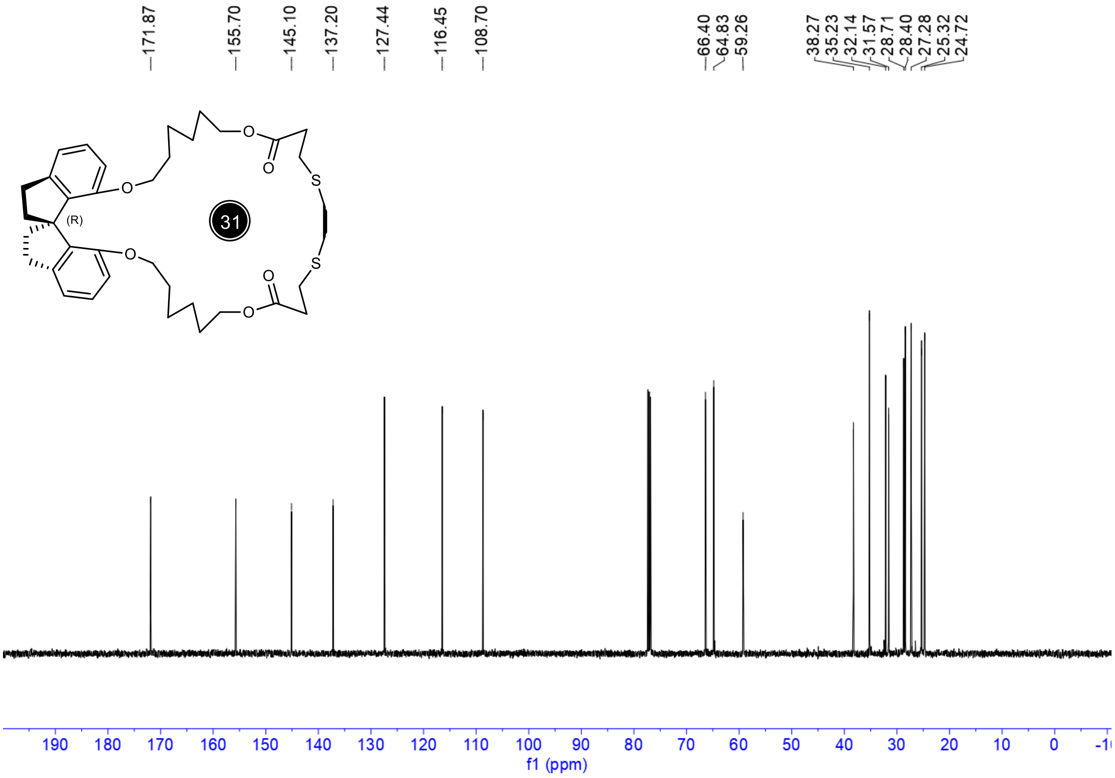

Supplementary Figure 270 | ^{1}H NMR (400 MHz, 298K, $CDCl_3$) of 6-Mercaptohexyl 2-(2-(2-oxo-2-((6-(styrylthio)hexyl)oxy)ethoxy)phenoxy)acetate **(b1)**

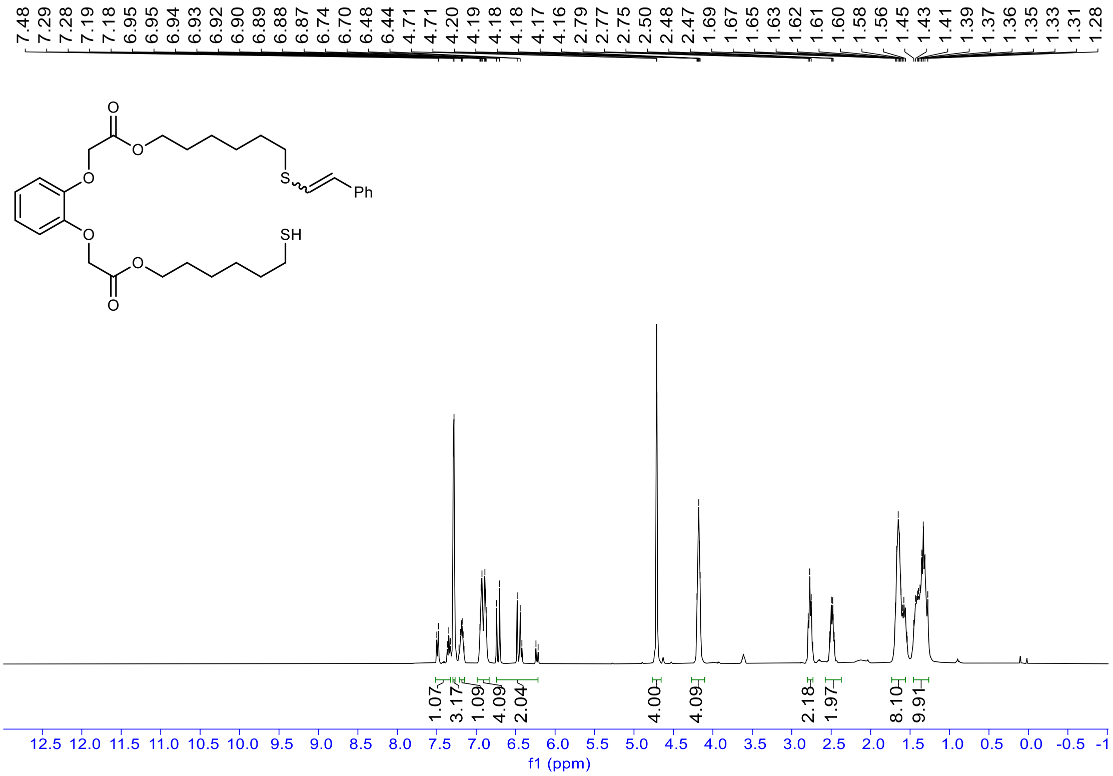

Supplementary Figure 271 | ^{13}C NMR (101 MHz, 298K, $CDCl_3$) of 6-mercaptohexyl 2-(2-(2-oxo-2-((6-(styrylthio)hexyl)oxy)ethoxy)phenoxy)acetate **(b1)**

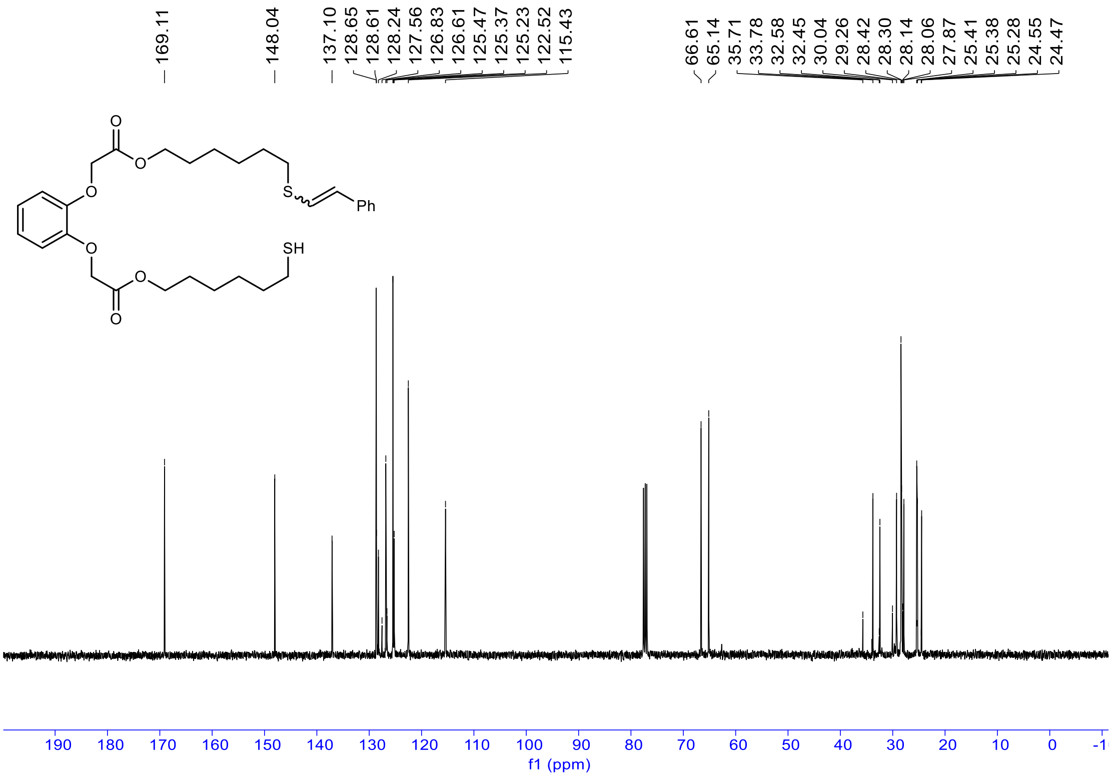

Supplementary Figure 272 | ^{1}H NMR (400 MHz, 298K, $CDCl_3$) of 6-(hex-1-en-1-ylthio)Hexyl 2-(2-(2-((6-mercaptohexyl)oxy)-2-oxoethoxy)phenoxy)acetate **(b2)**

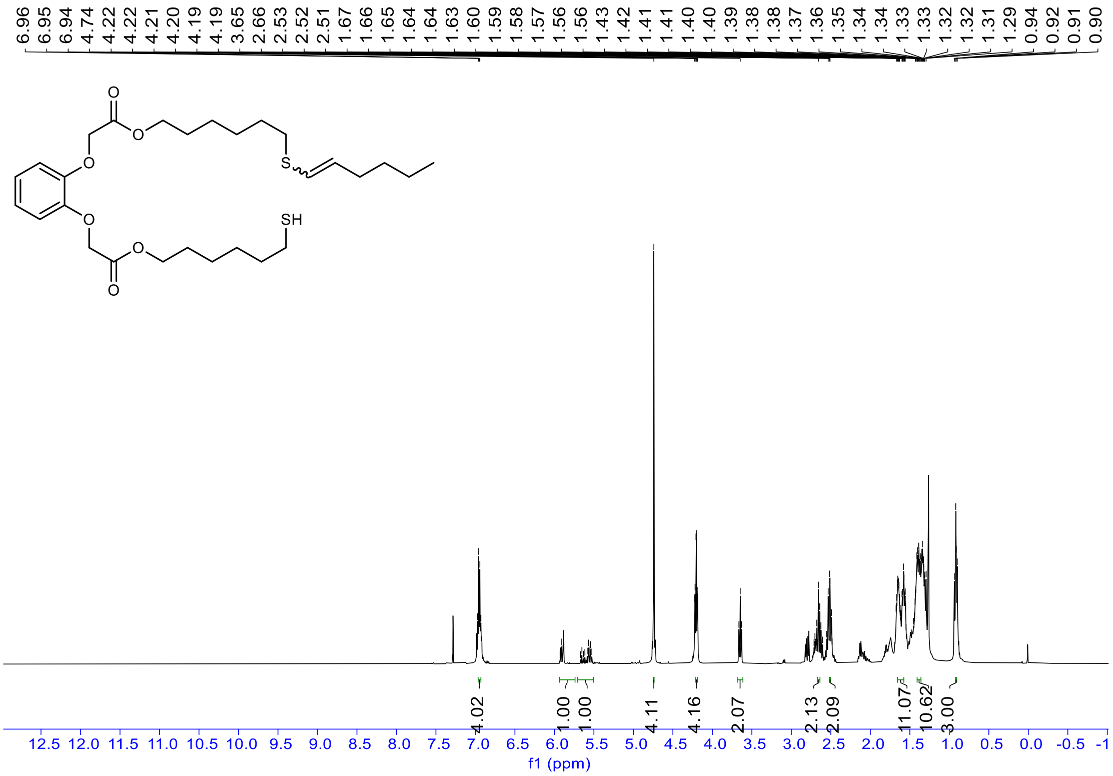

Supplementary Figure 273 | ^{13}C NMR (101 MHz, 298K, $CDCl_3$) of 6-(hex-1-en-1-ylthio)Hexyl 2-(2-(2-((6-mercaptohexyl)oxy)-2-oxoethoxy)phenoxy)acetate **(b2)**

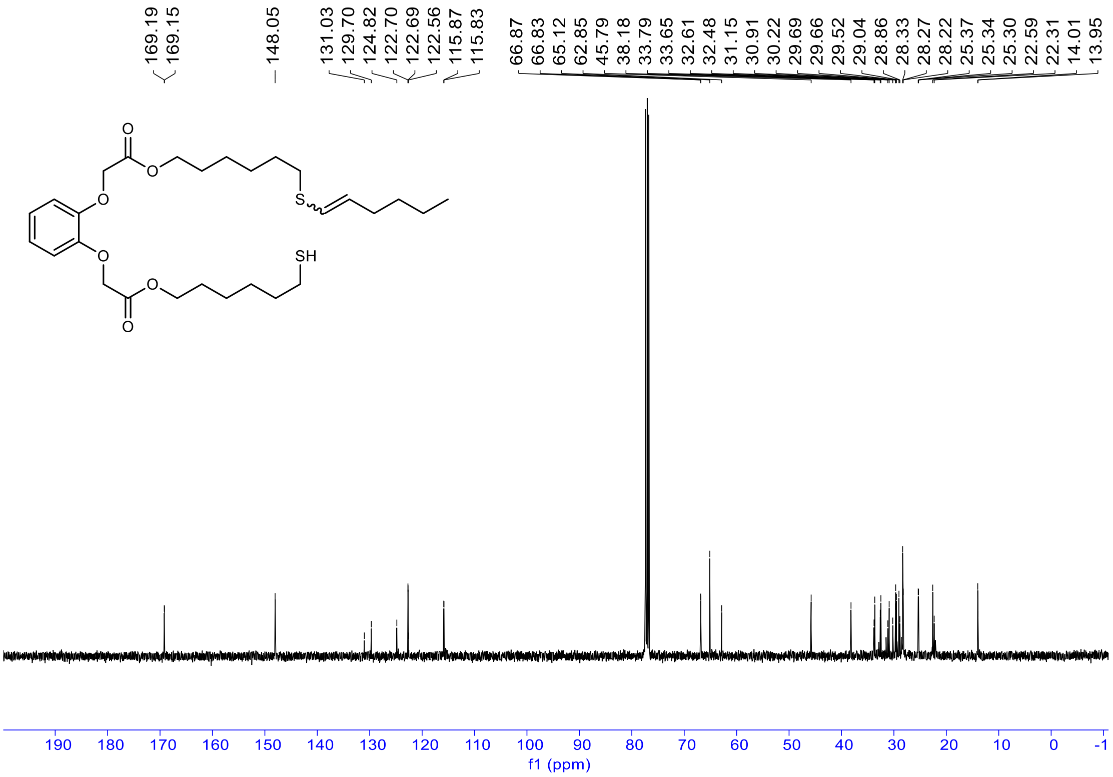

Supplementary Figure 274 | ^{1}H NMR (400 MHz, 298K, $CDCl_3$) of bis(6-(hex-1-en-1-ylthio)Hexyl) 2,2'-(1,2-phenylenebis(oxy))diacetate **(c1)**

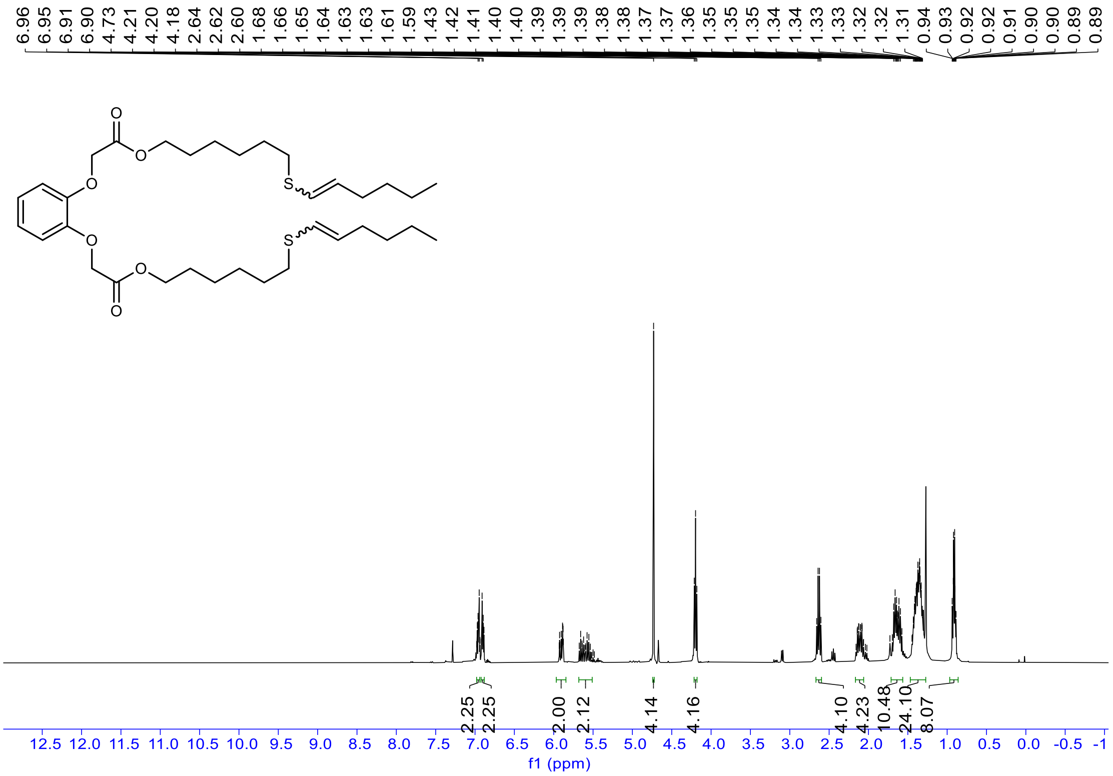

Supplementary Figure 275 | ^{13}C NMR (101 MHz, 298K, $CDCl_3$) of bis(6-(hex-1-en-1-ylthio)Hexyl) 2,2'-(1,2-phenylenebis(oxy))diacetate **(c1)**

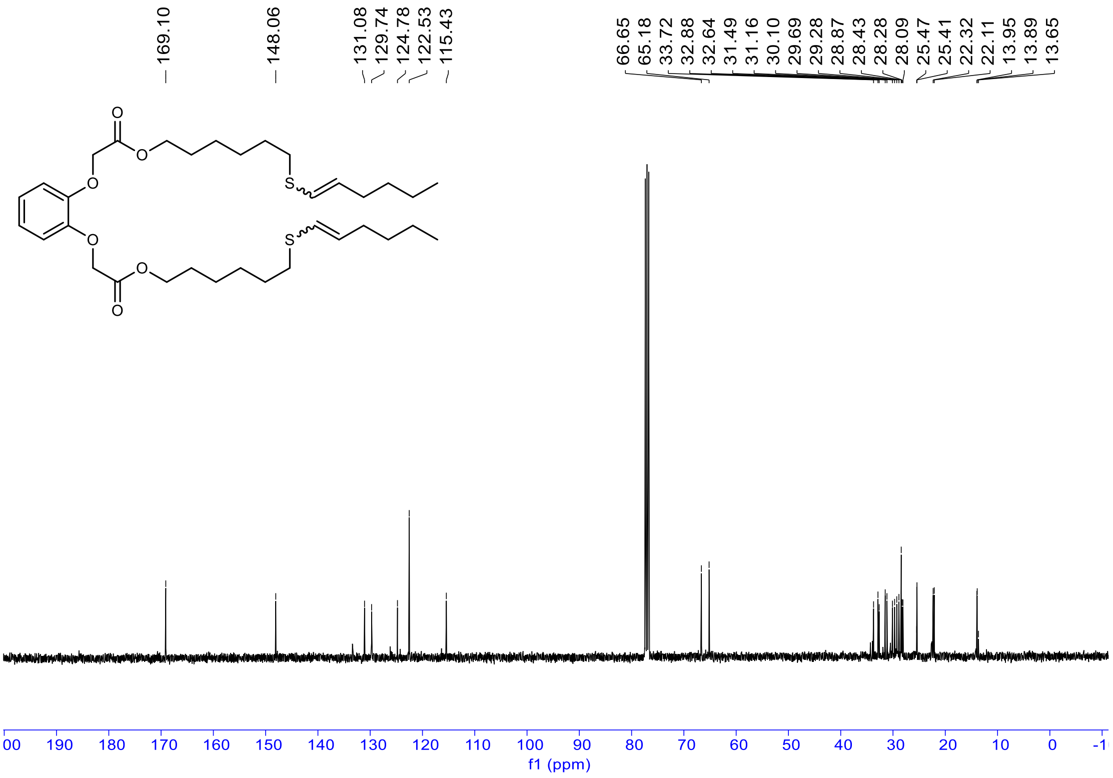

Supplementary Figure 276 | ^{1}H NMR (400 MHz, 298K, $CDCl_3$) of 1,4,7,10-Tetrathiacyclododecane **(52)**

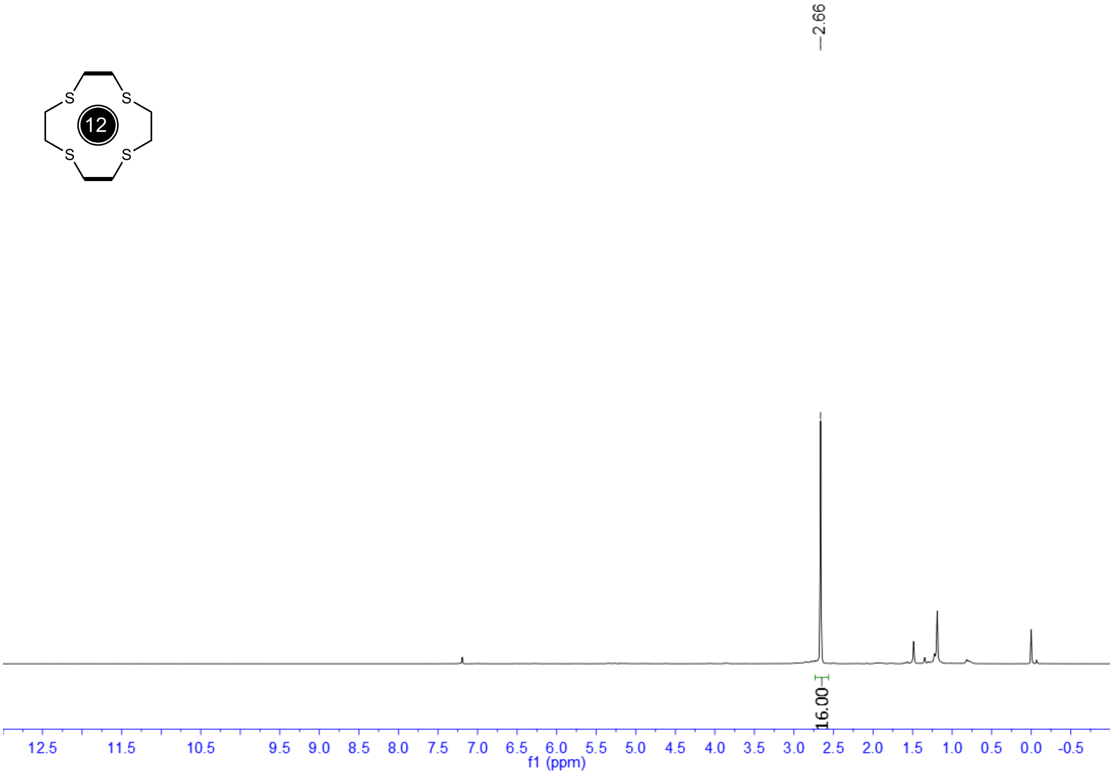

Supplementary Figure 277 | ^{13}C NMR (101 MHz, 298K, $CDCl_3$) of 1,4,7,10-Tetrathiacyclododecane **(52)**

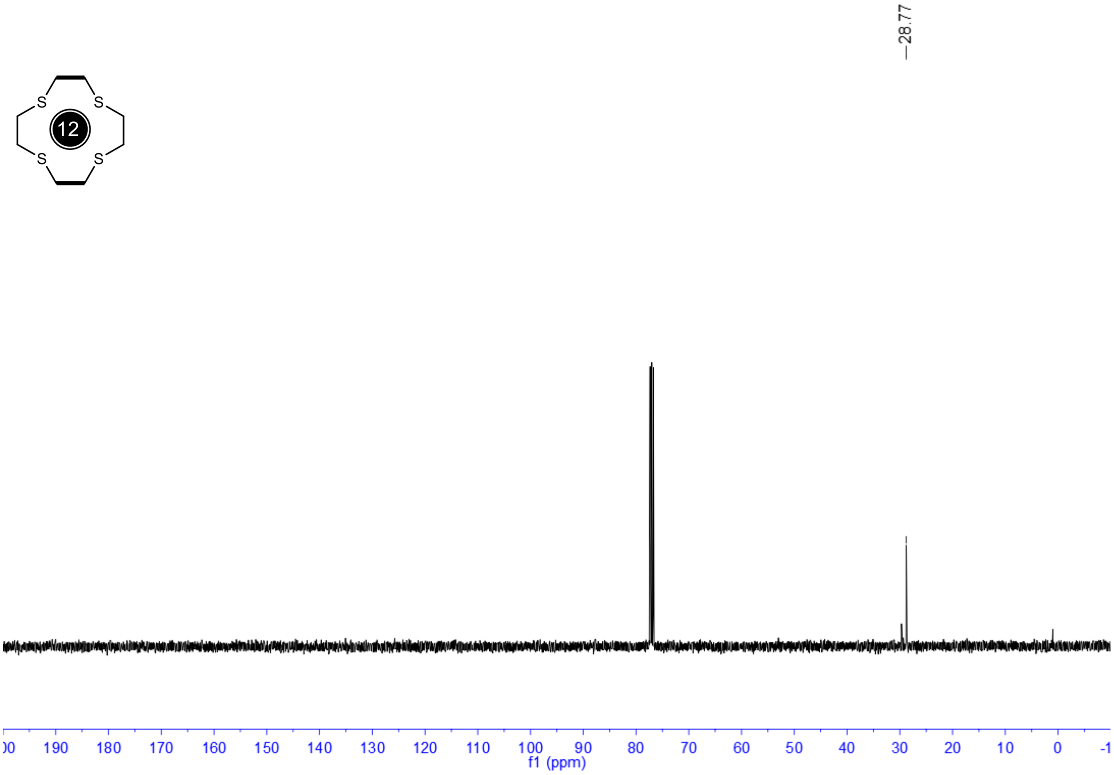

Supplementary Figure 278 | ^{1}H NMR (500 MHz, 298K, Methanol-d_4) of 1,4,9,12-Tetrathiacyclohexadecane-6,7,14,15-tetraol **(53)**

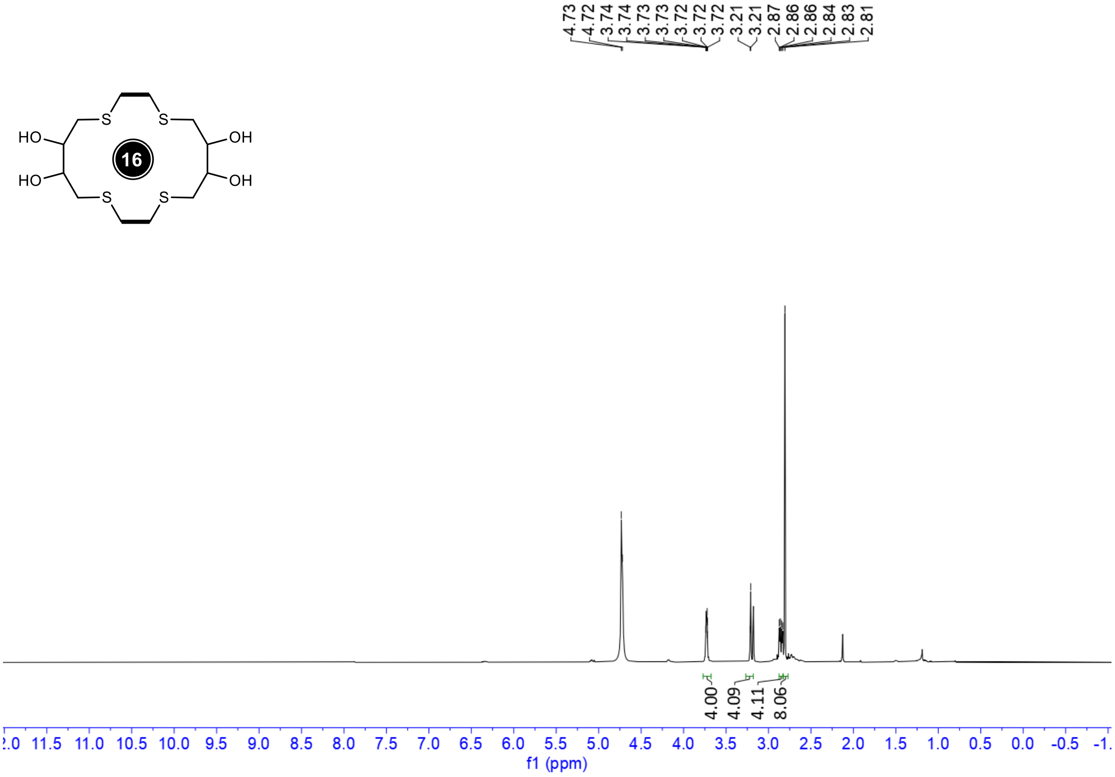

Supplementary Figure 279 | ^{13}C NMR (126 MHz, 298K, Methanol-d_4) of 1,4,9,12-Tetrathiacyclohexadecane-6,7,14,15-tetraol **(53)**

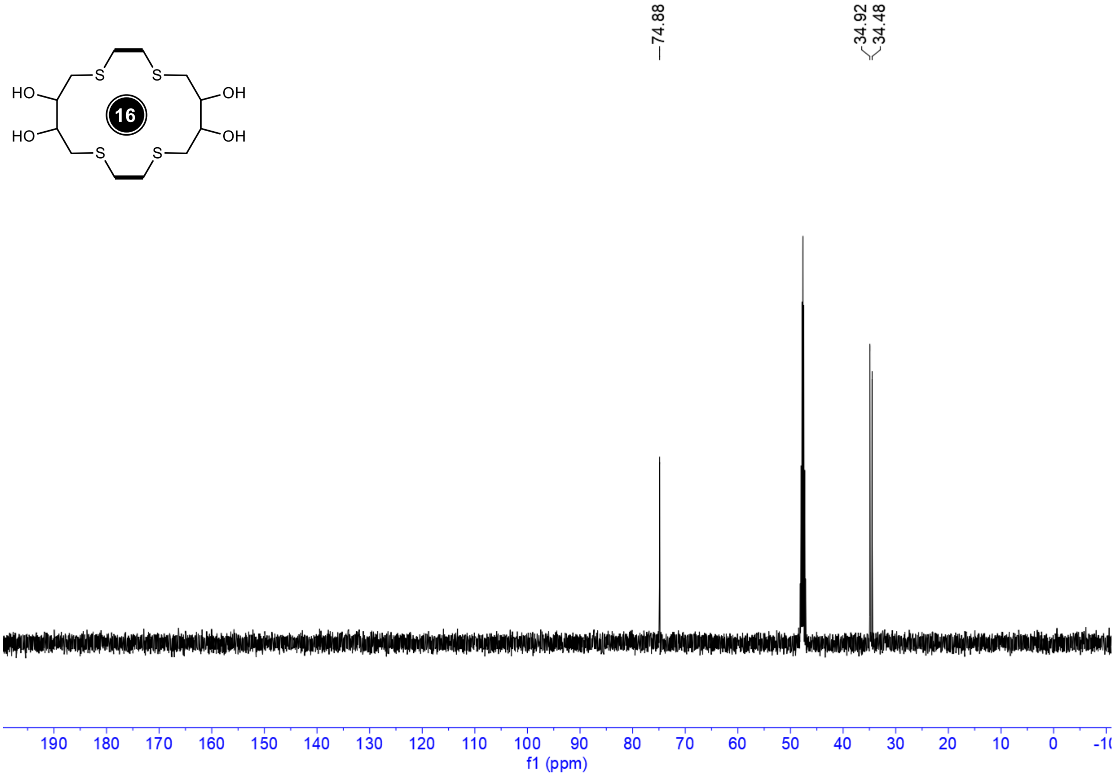

Supplementary Figure 280 | ^{1}H NMR (400 MHz, 298K, $CDCl_3$) of 1,4,8,11-Tetrathiacyclotetradecane **(54)**

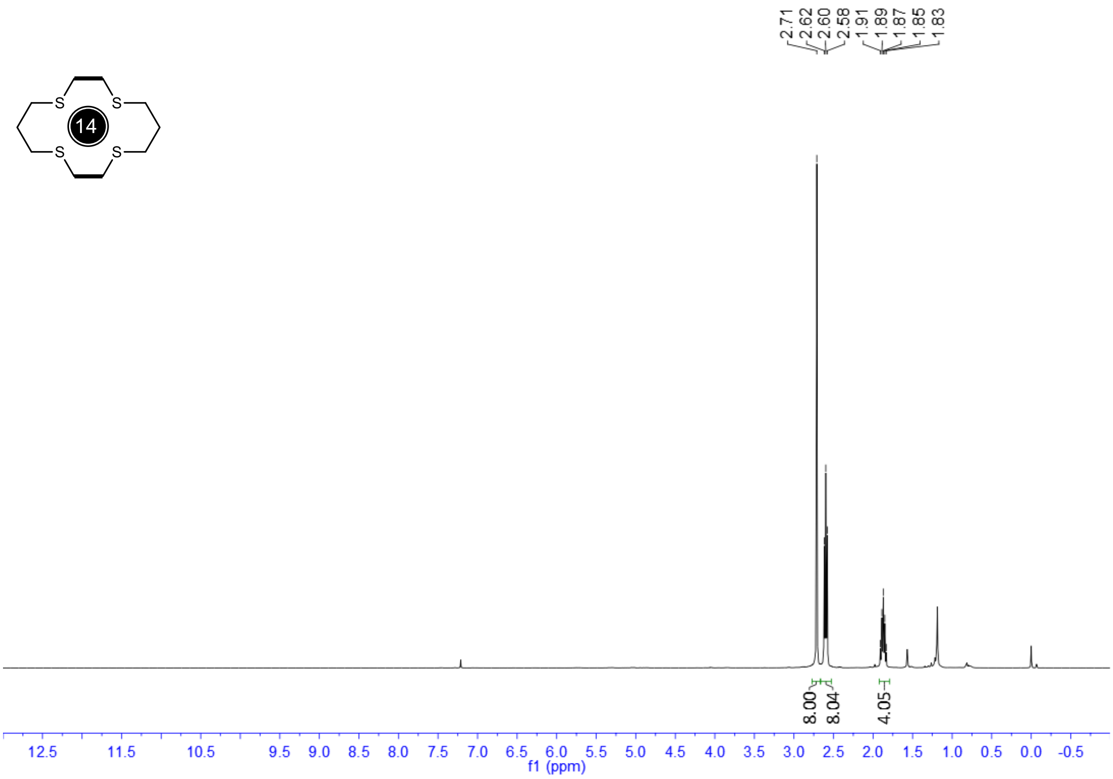

Supplementary Figure 281 | ^{13}C NMR (101 MHz, 298K, $CDCl_3$) of 1,4,8,11-Tetrathiacyclotetradecane **(54)**

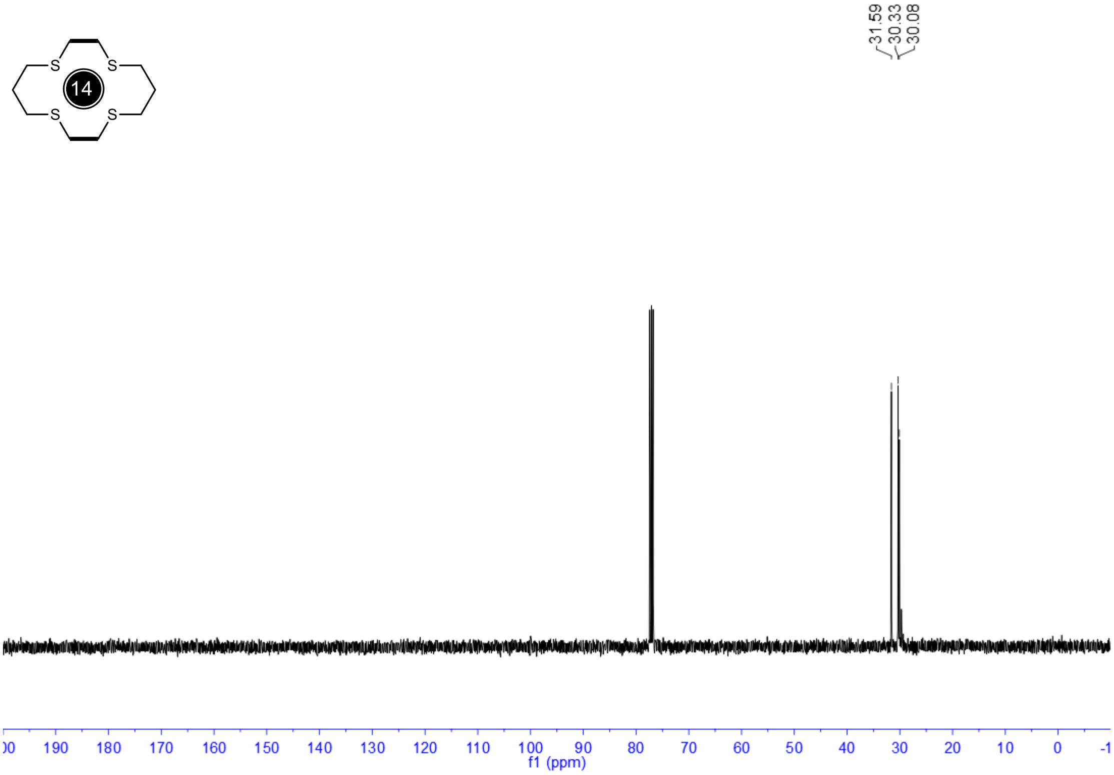

Supplementary Figure 282 | ^{1}H NMR (400 MHz, 298K, $CDCl_3$) of 1,4,9,12-Tetrathiacyclohexadecane **(55)**

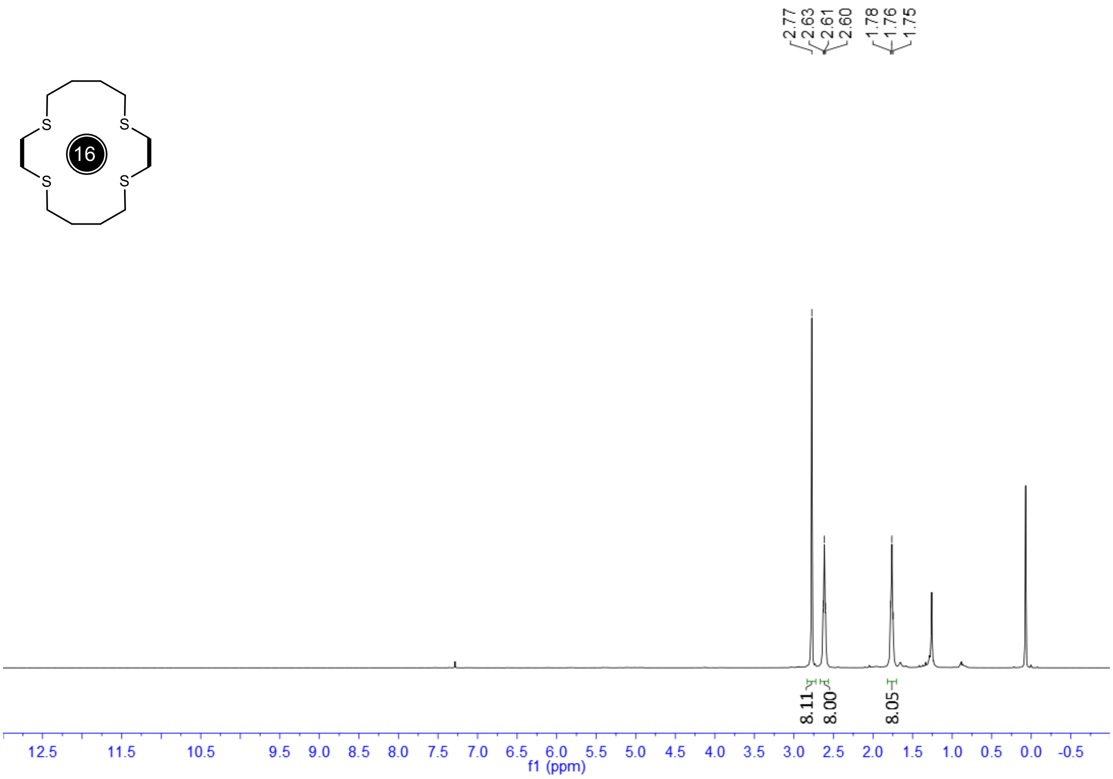

Supplementary Figure 283 | ^{13}C NMR (101 MHz, 298K, $CDCl_3$) of 1,4,9,12-Tetrathiacyclohexadecane **(55)**

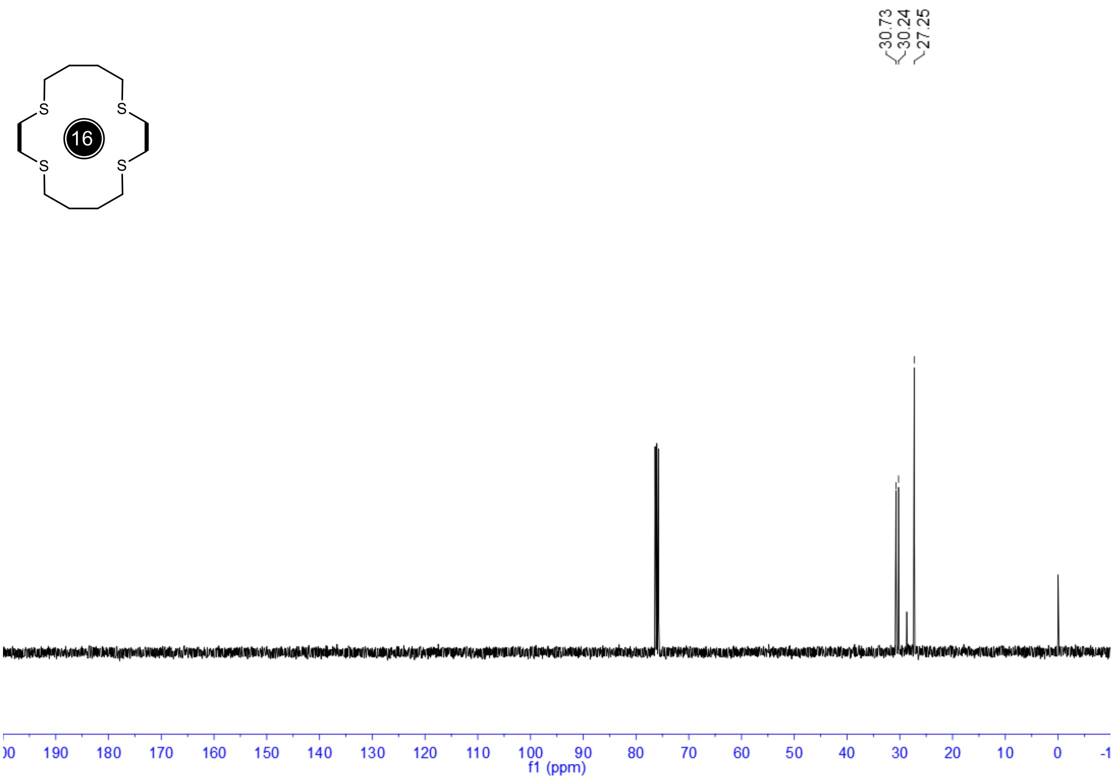

Supplementary Figure 284 | ^{1}H NMR (400 MHz, 298K, $CDCl_3$) of 1,4,10,13-Tetrathiacyclooctadecane **(56)**

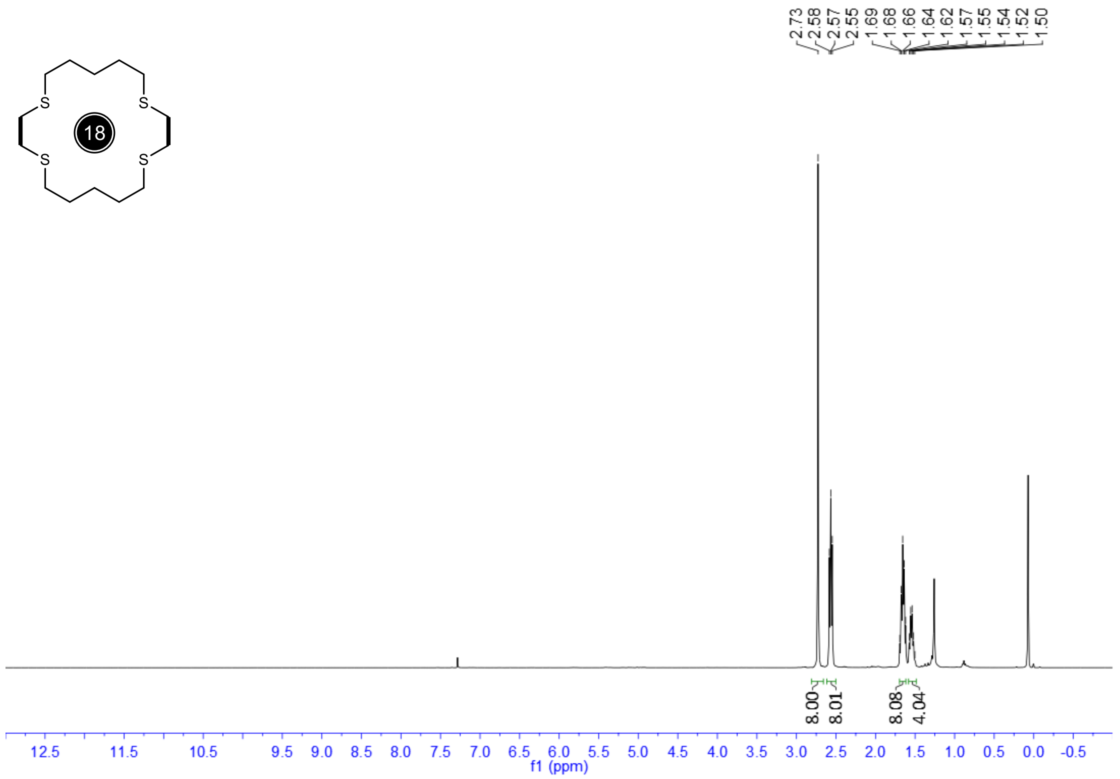

Supplementary Figure 285 | ^{13}C NMR (101 MHz, 298K, $CDCl_3$) of 1,4,10,13-Tetrathiacyclooctadecane **(56)**

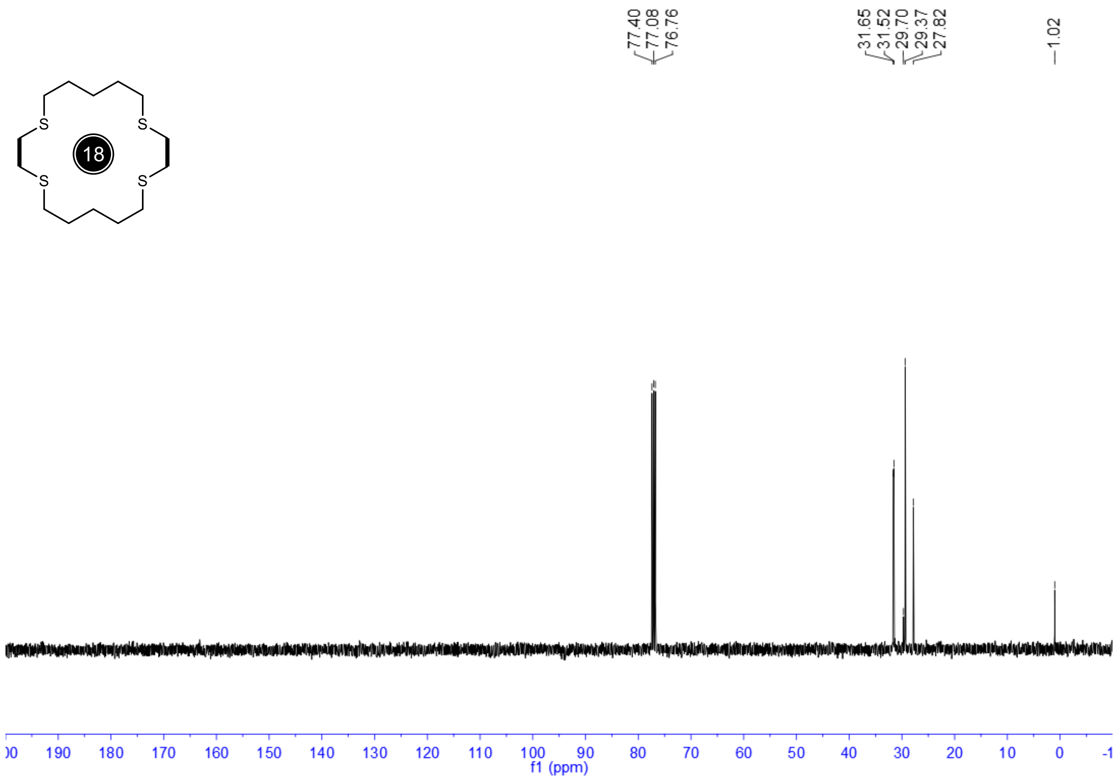

Supplementary Figure 286 | ^{1}H NMR (400 MHz, 298K, $CDCl_3$) of 1,4,11,14-Tetrathiacycloicosane **(57)**

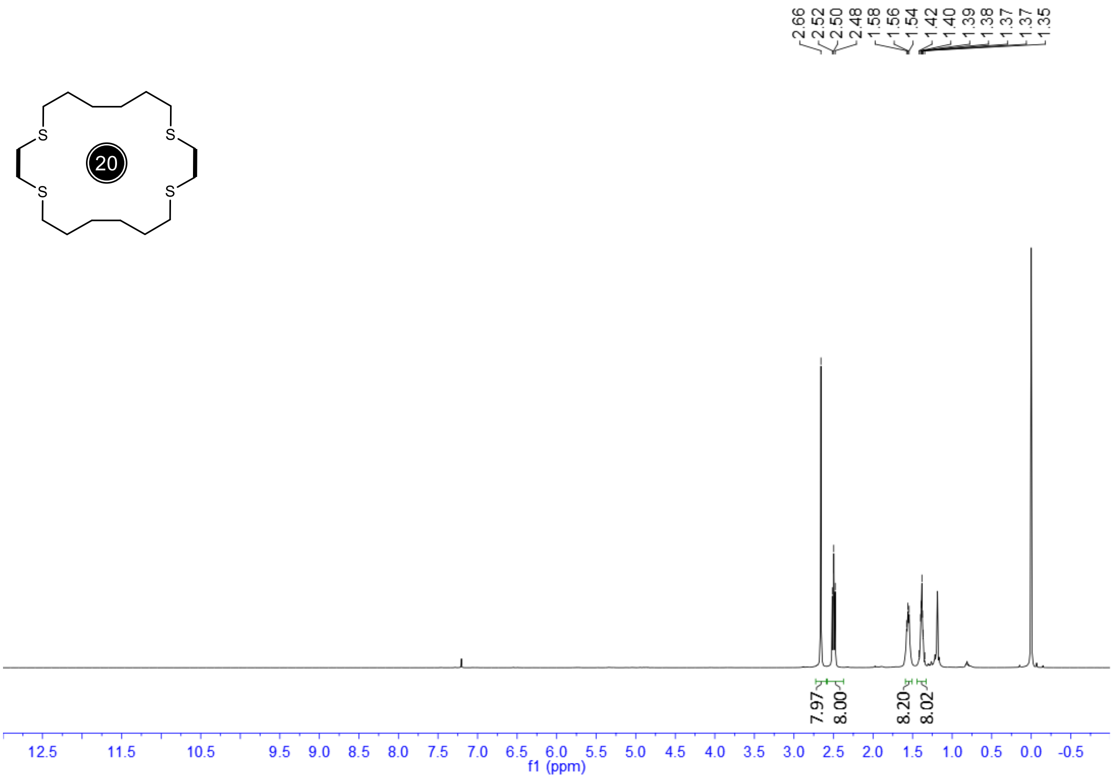

Supplementary Figure 287 | ^{13}C NMR (101 MHz, 298K, $CDCl_3$) of 1,4,11,14-Tetrathiacycloicosane **(57)**

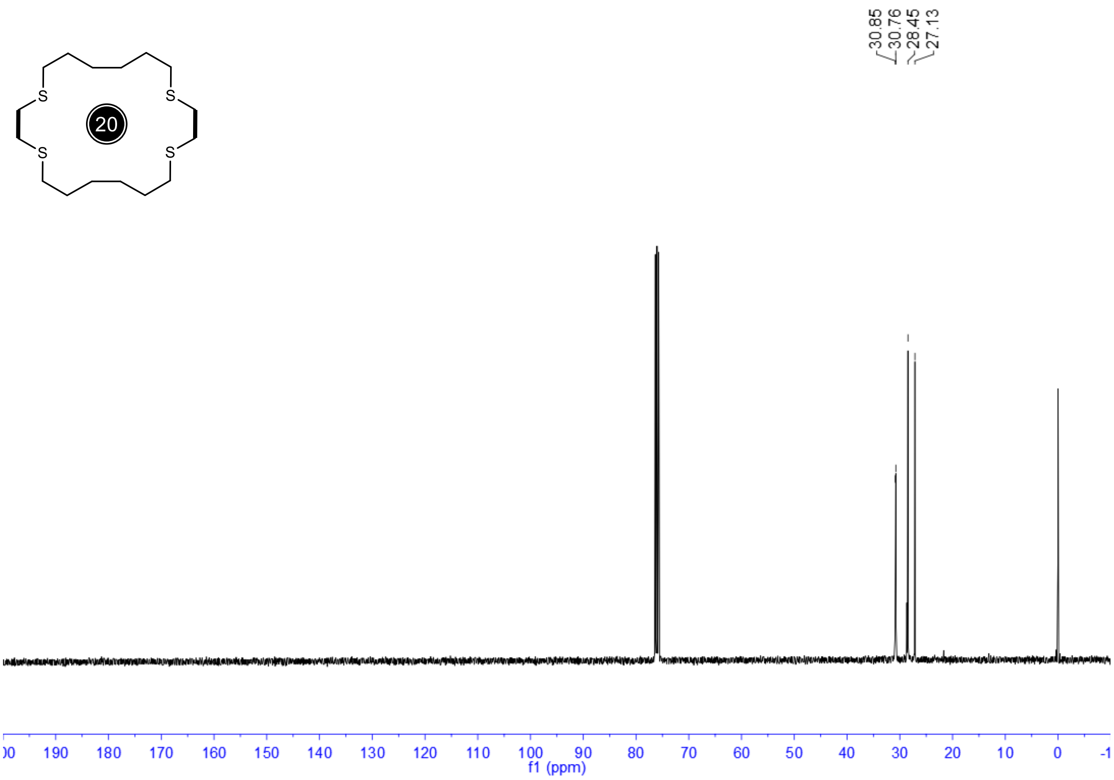

Supplementary Figure 288 | ^{1}H NMR (400 MHz, 298K, $CDCl_3$) of 1,4,13,16-Tetrathiacyclotetracosane **(58)**

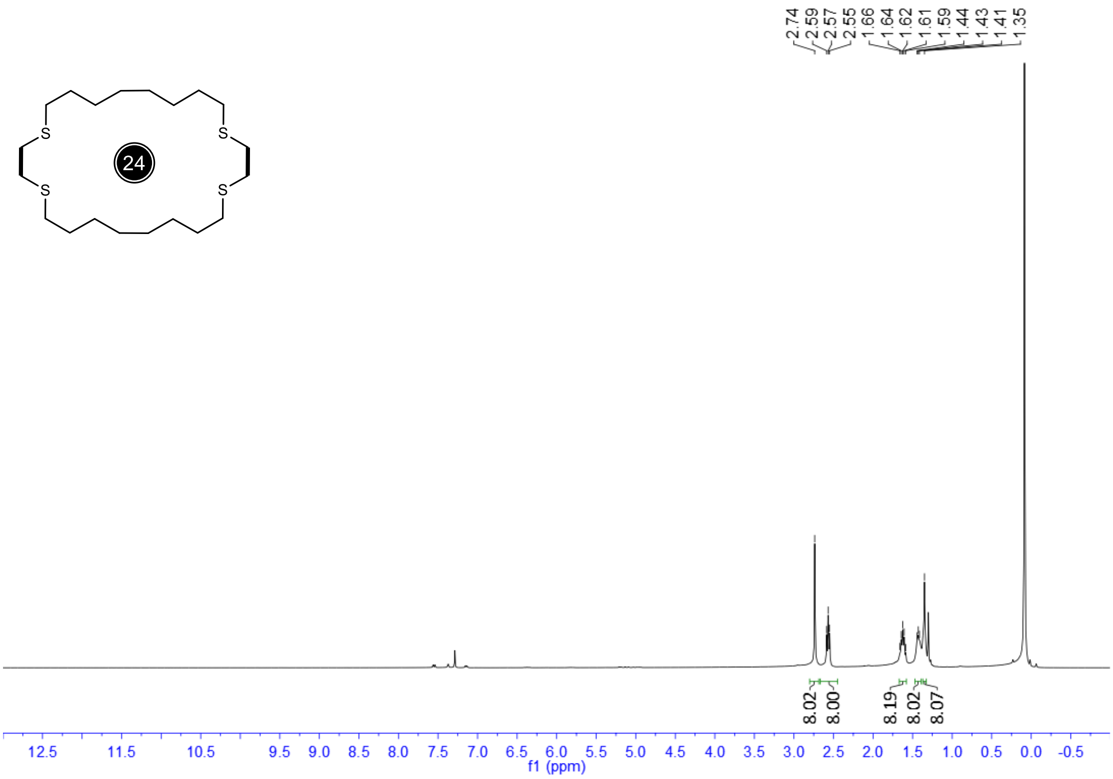

Supplementary Figure 289 | ^{13}C NMR (101 MHz, 298K, $CDCl_3$) of 1,4,13,16-Tetrathiacyclotetracosane **(58)**

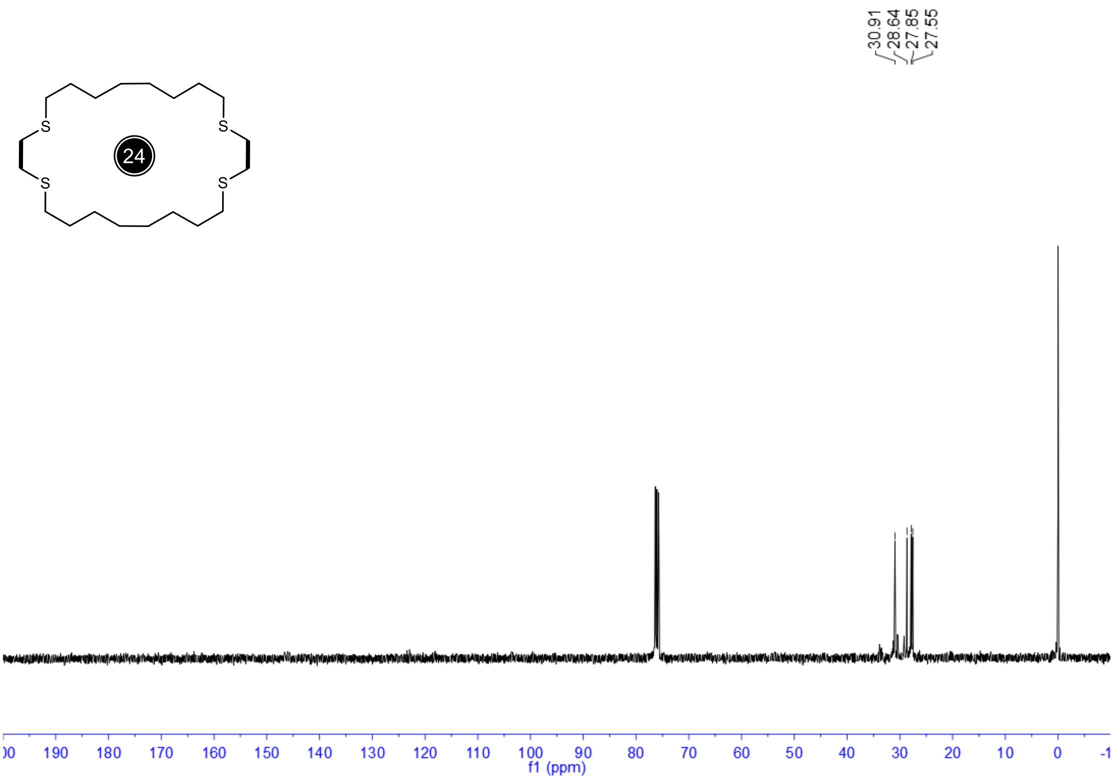

Supplementary Figure 290 | ^{1}H NMR (400 MHz, 298K, $CDCl_3$) of 1,4,14,17-Tetrathiacyclohexacosane **(59)**

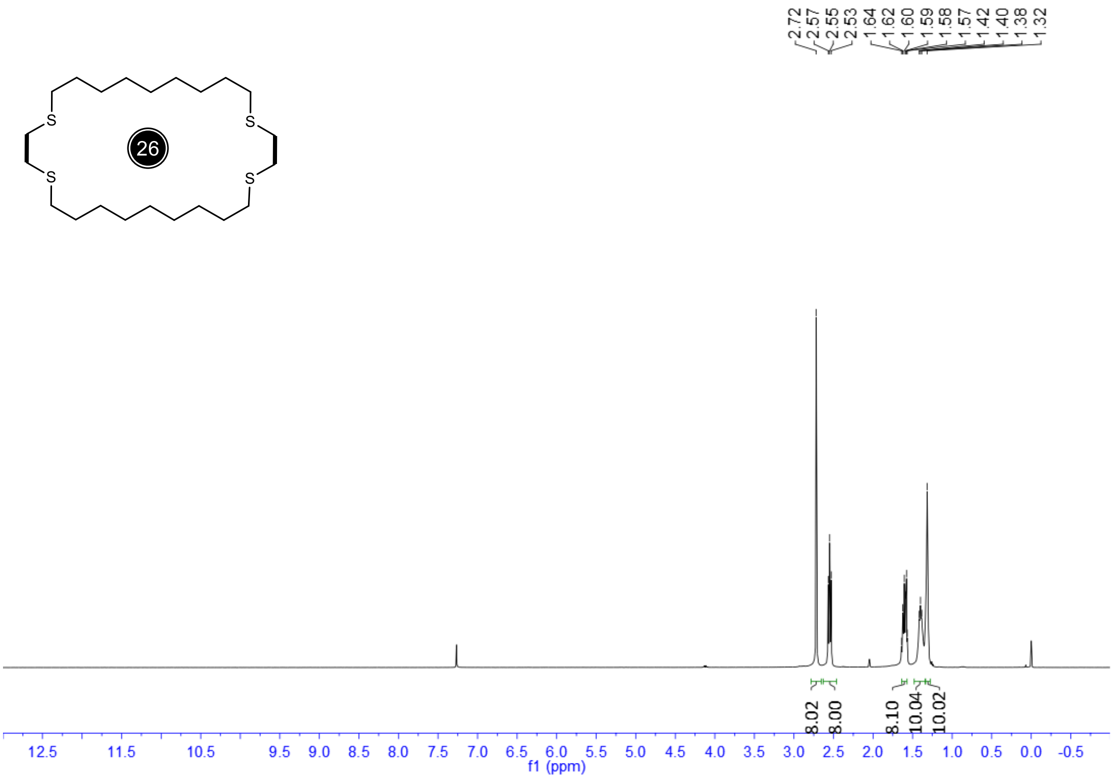

Supplementary Figure 291 | ^{13}C NMR (101 MHz, 298K, $CDCl_3$) of 1,4,14,17-Tetrathiacyclohexacosane **(59)**

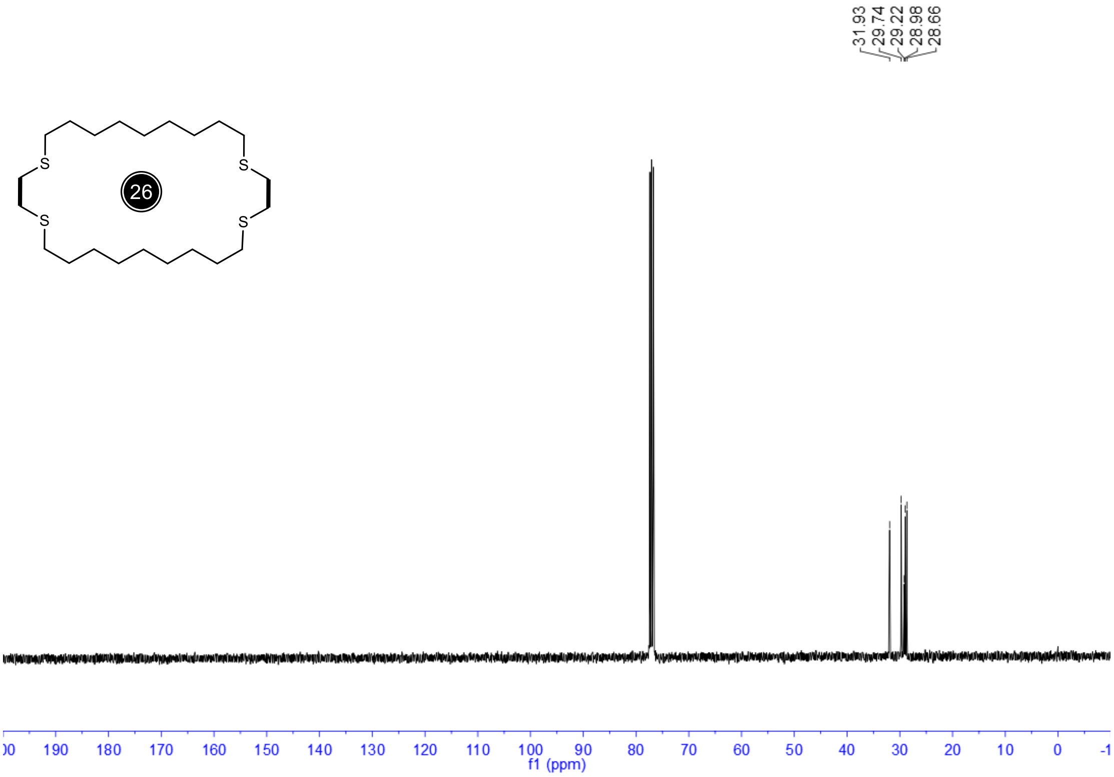

Supplementary Figure 292 | ^{1}H NMR (400 MHz, 298K, $CDCl_3$) of 1,4,15,18-Tetrathiacyclooctacosane **(60)**

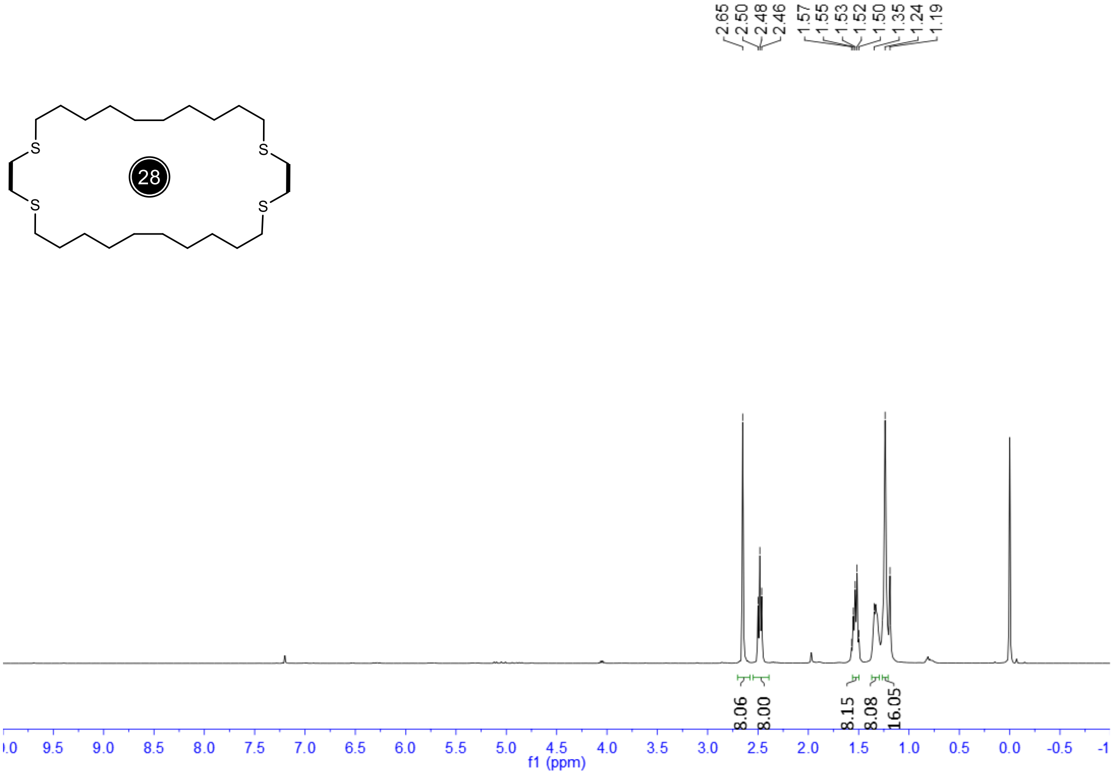

Supplementary Figure 293 | ^{13}C NMR (101 MHz, 298K, $CDCl_3$) of 1,4,15,18-Tetrathiacyclooctacosane **(60)**

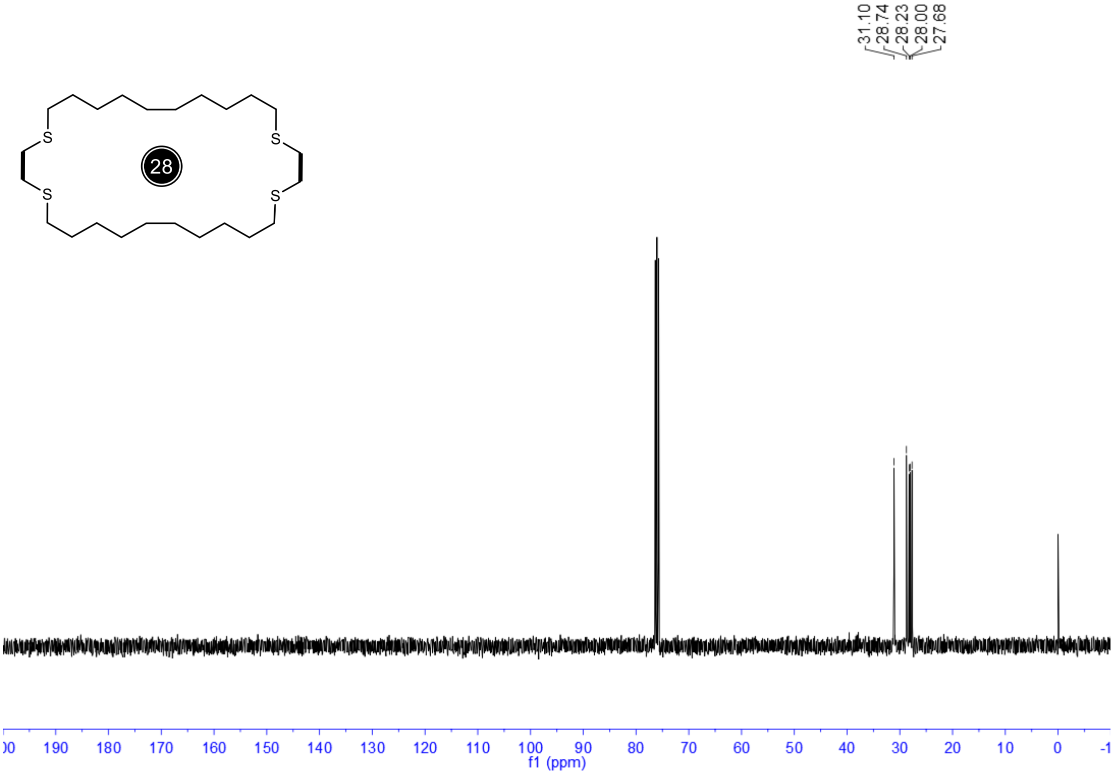

Supplementary Figure 294 | ^{1}H NMR (400 MHz, 298K, $CDCl_3$) of 1,4,13,16-Tetraoxa-7,10,19,22-tetrathiacyclotetracosane **(61)**

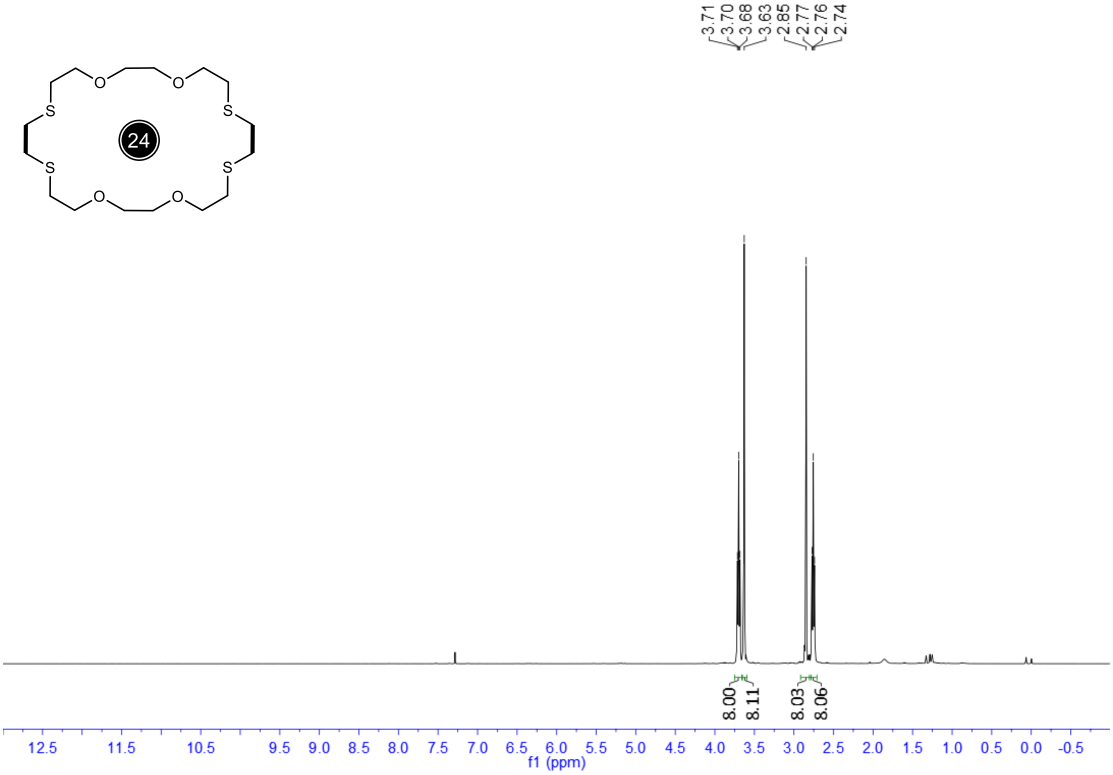

Supplementary Figure 295 | ^{13}C NMR (101 MHz, 298K, $CDCl_3$) of 1,4,13,16-Tetraoxa-7,10,19,22-tetrathiacyclotetracosane **(61)**

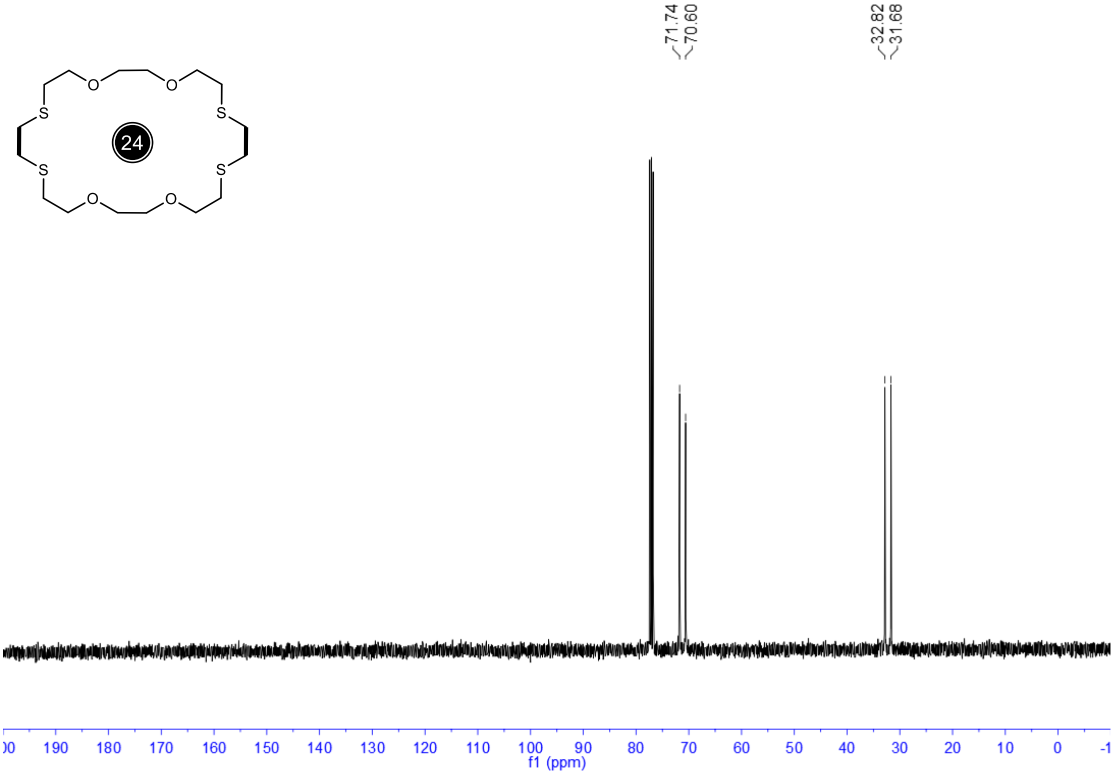

Supplementary Figure 296 | ^{1}H NMR (400 MHz, 298K, $CDCl_3$) of 1,4-Dioxa-7,10-dithiacyclododecane **(62)**

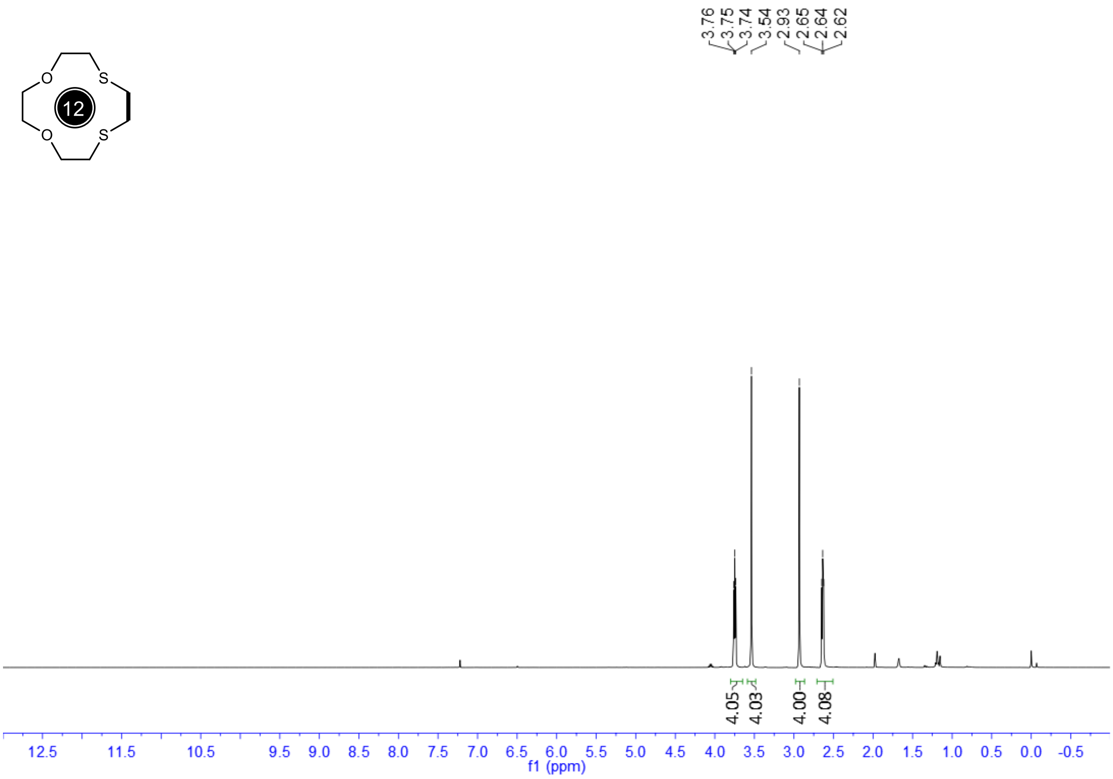

Supplementary Figure 297 | ^{13}C NMR (101 MHz, 298K, $CDCl_3$) of 1,4-Dioxa-7,10-dithiacyclododecane **(62)**

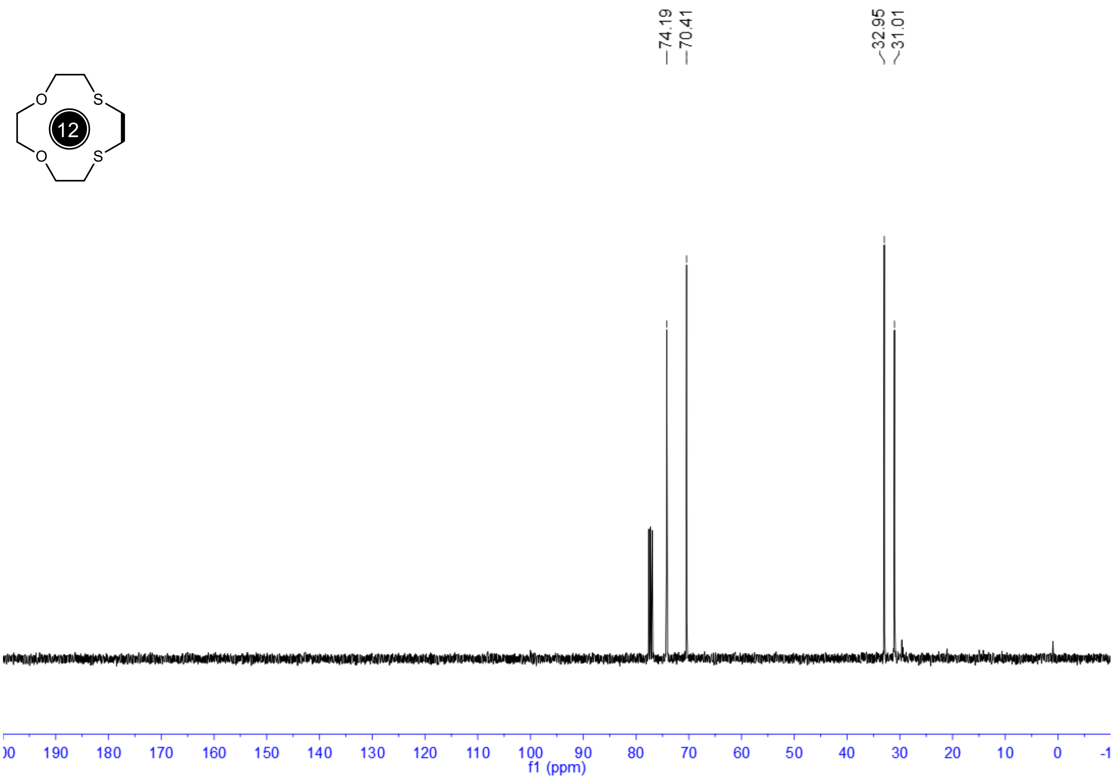

Supplementary Figure 298 | ^{1}H NMR (500 MHz, 298K, $CDCl_3$) of 1,10-Dioxa-4,7,13,16-tetrathiacyclooctadecane **(63)**

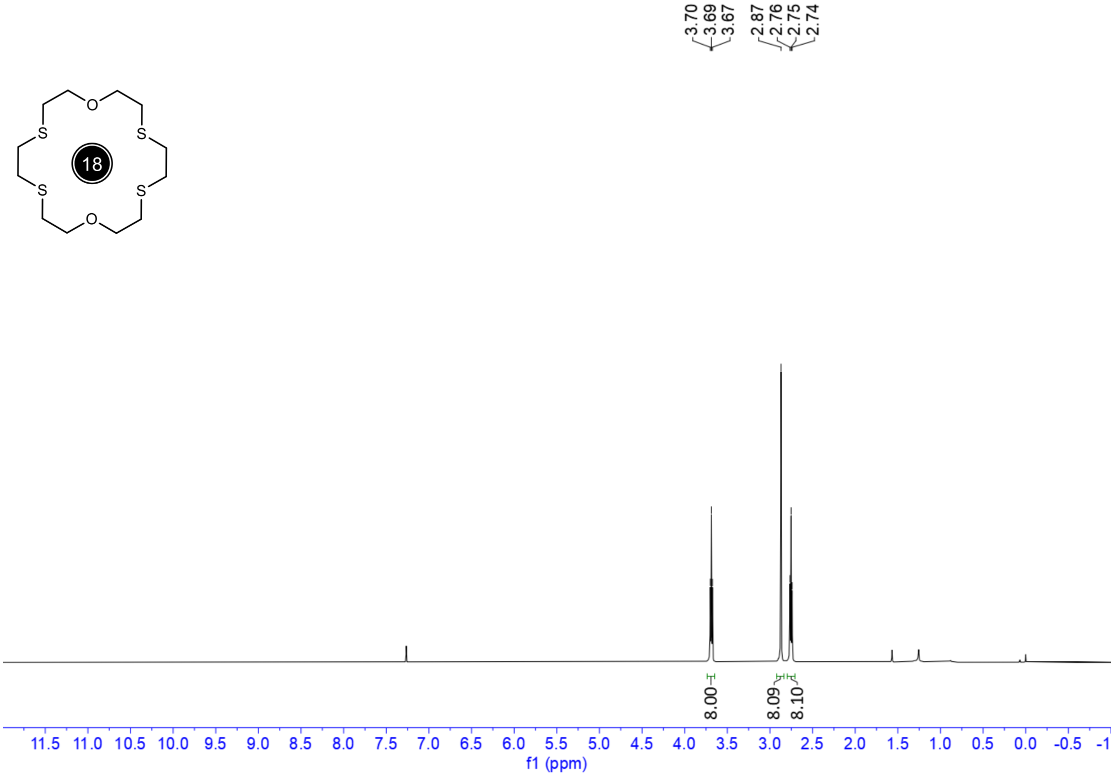

Supplementary Figure 299 | ^{13}C NMR (126 MHz, 298K, $CDCl_3$) of 1,10-Dioxa-4,7,13,16-tetrathiacyclooctadecane **(63)**

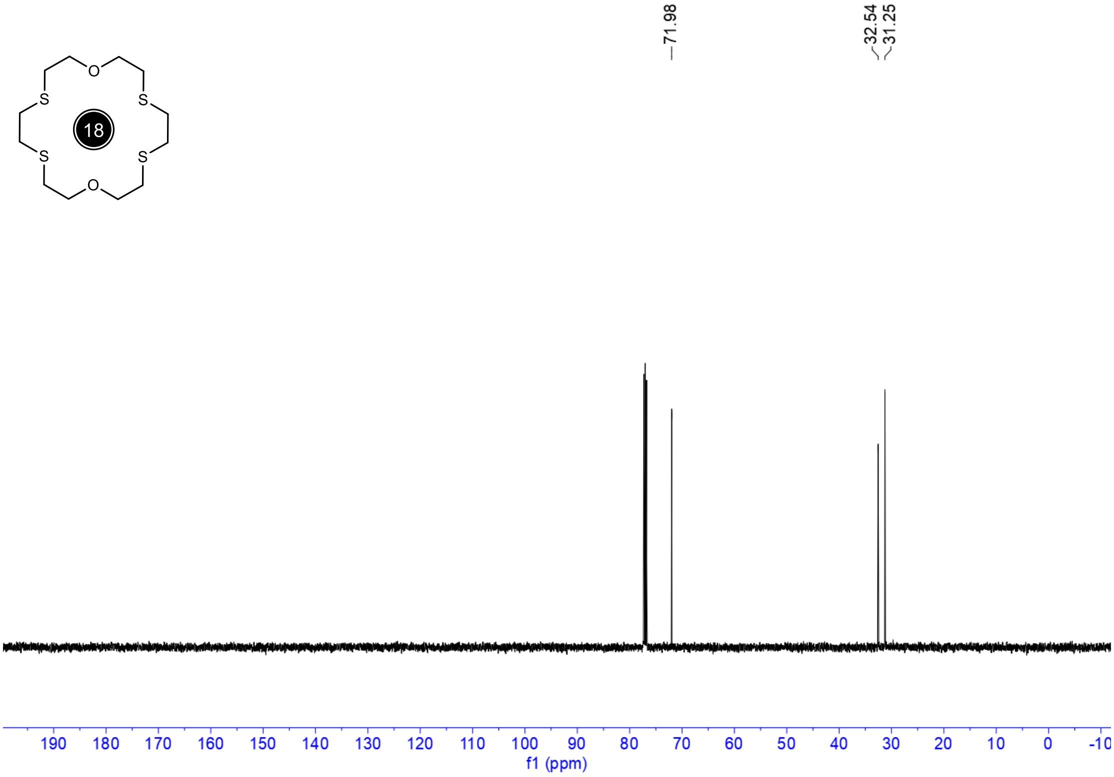

Supplementary Figure 300 | ^{1}H NMR (500 MHz, 298K, $CDCl_3$) of 1,4,7-Oxadithionane **(64)**

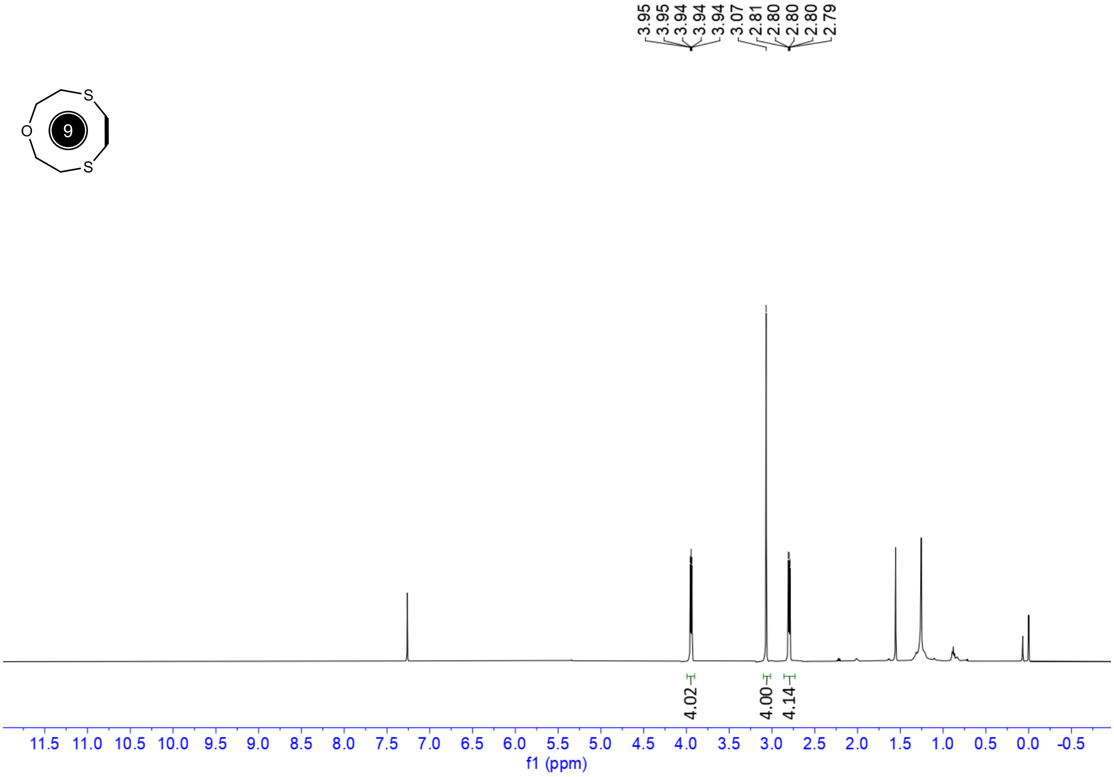

Supplementary Figure 301 | ^{13}C NMR (126 MHz, 298K, $CDCl_3$) of 1,4,7-Oxadithionane **(64)**

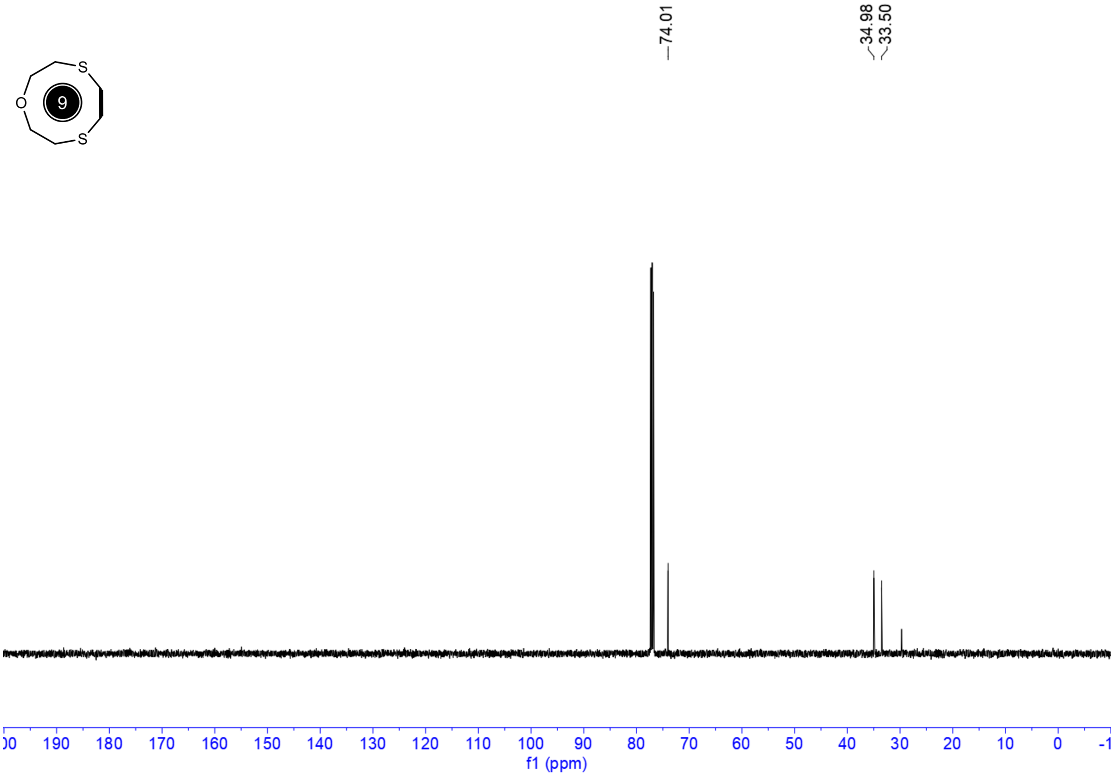

Supplementary Figure 302 | ^{1}H NMR (500 MHz, 298K, $CDCl_3$) of 1,4,7,10,14,17,20,23-Octathiacyclohexacosane **(65)**

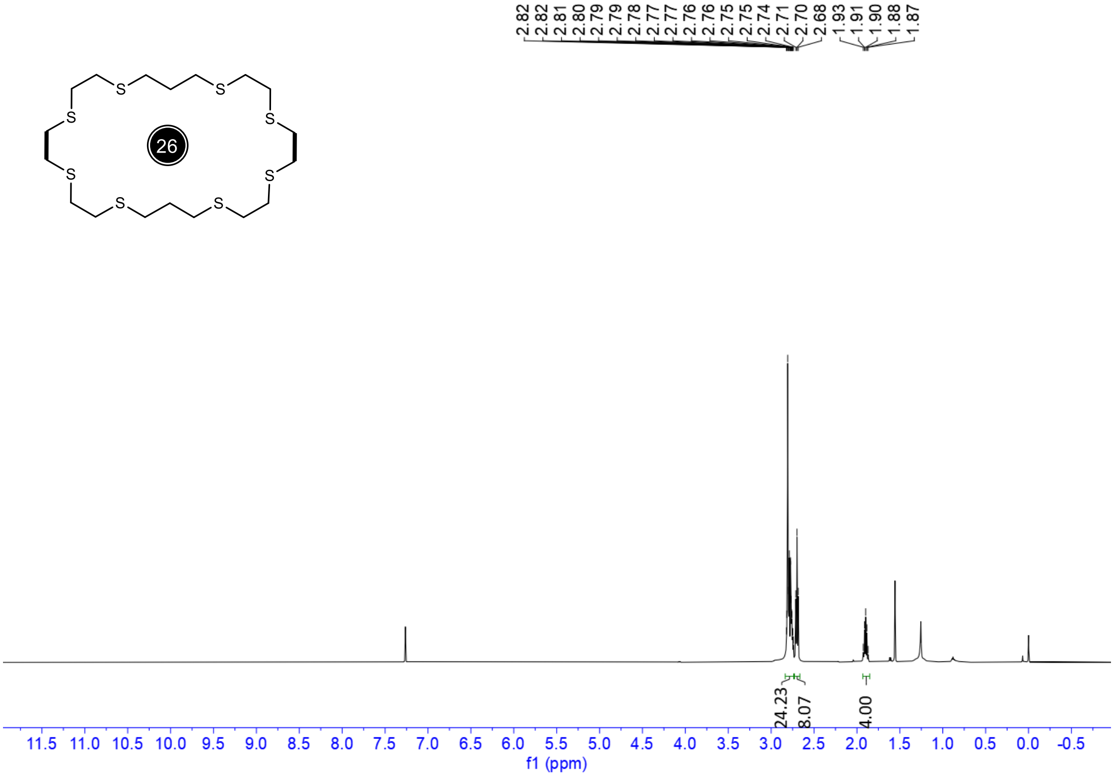

Supplementary Figure 303 | ^{13}C NMR (126 MHz, 298K, $CDCl_3$) of 1,4,7,10,14,17,20,23-Octathiacyclohexacosane **(65)**

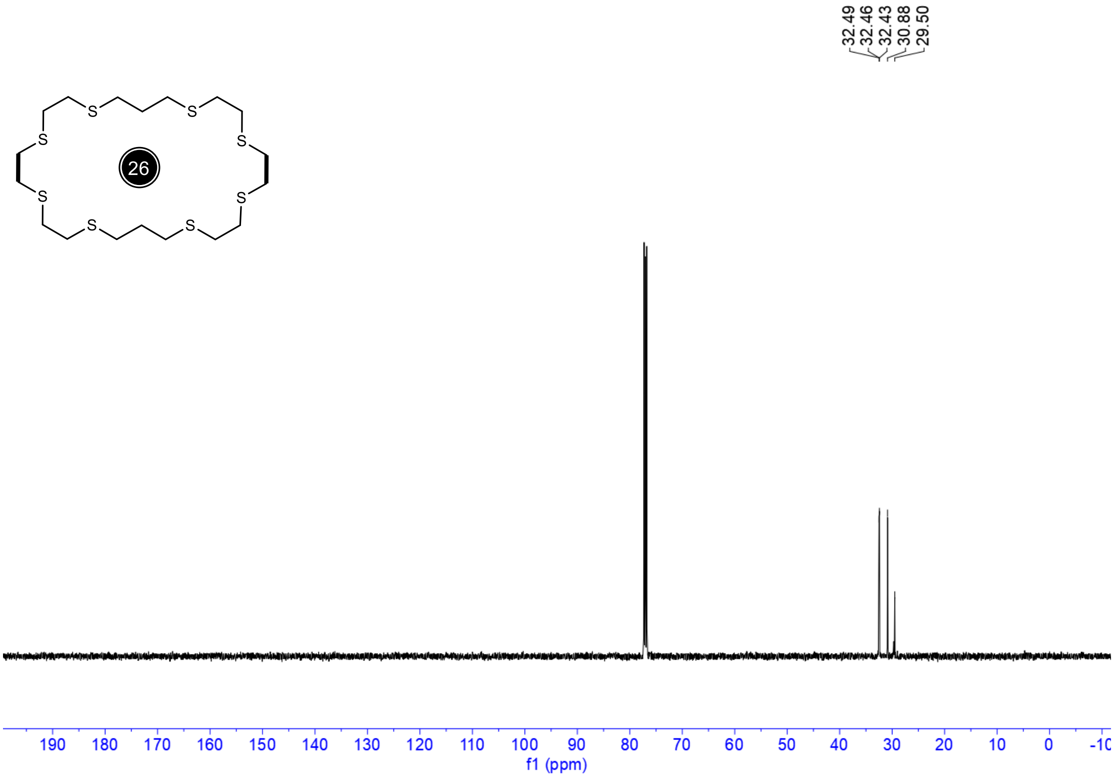

Supplementary Figure 304 | ^{1}H NMR (500 MHz, 298K, $CDCl_3$) of 1,4,7,10-Tetrathiacyclotridecane **(66)**

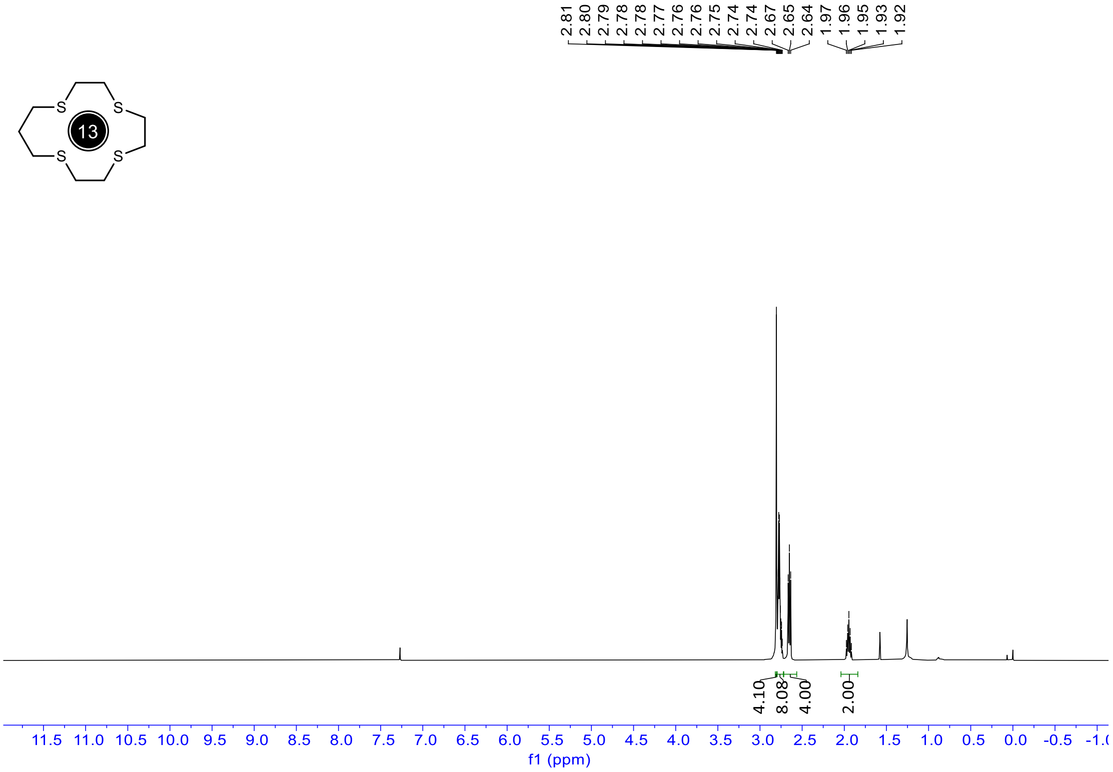

Supplementary Figure 305 | ^{13}C NMR (126 MHz, 298K, $CDCl_3$) of 1,4,7,10-Tetrathiacyclotridecane **(66)**

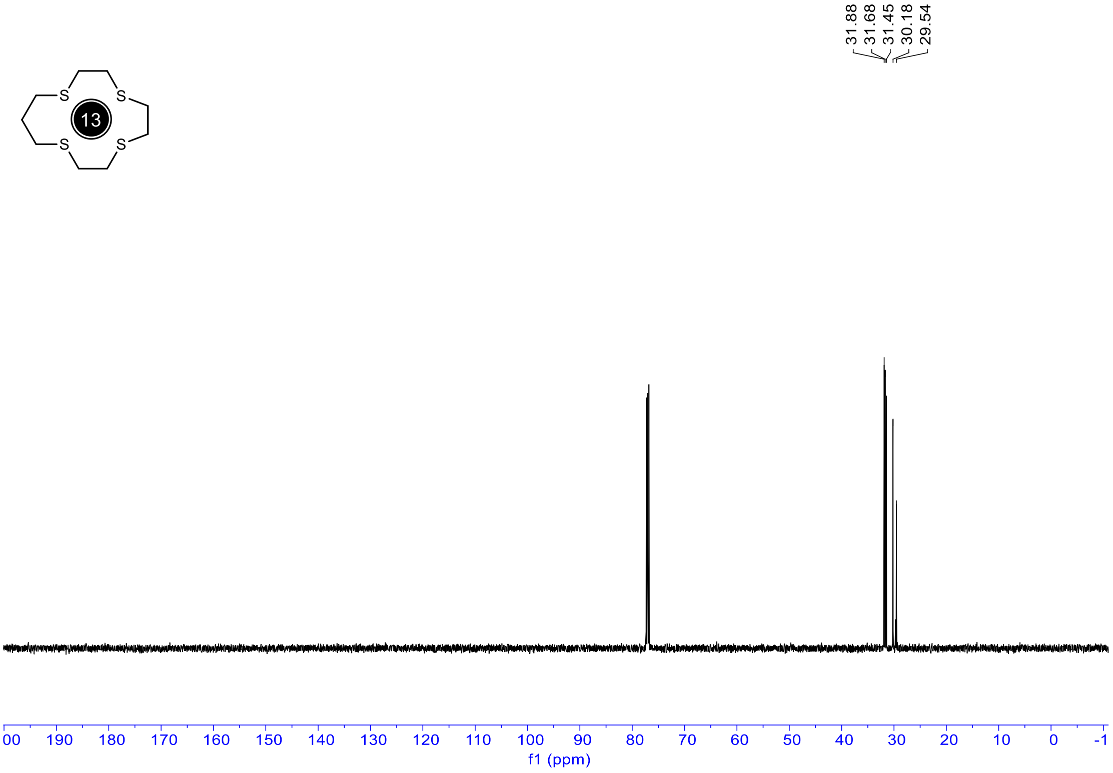

Supplementary Figure 306 | ^{1}H NMR (400 MHz, 298K, $CDCl_3$) of 2-Bis(phenylthio)ethane **(67)**

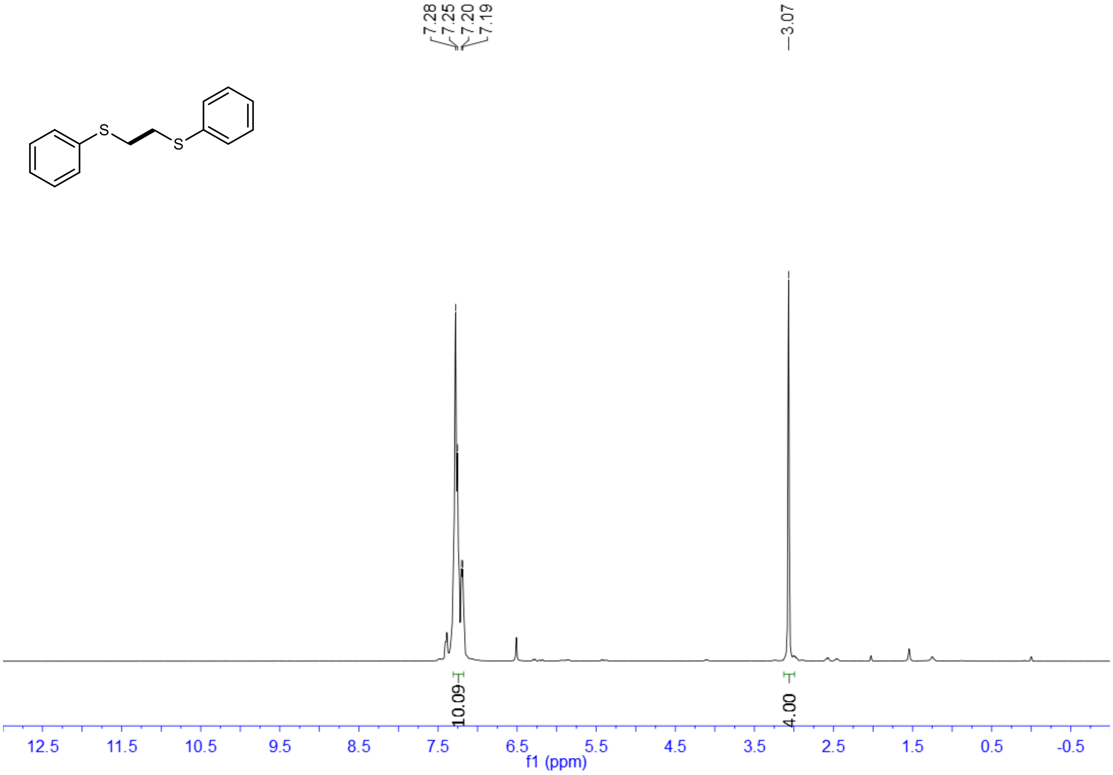

Supplementary Figure 307 | ^{13}C NMR (101 MHz, 298K, $CDCl_3$) of 2-Bis(phenylthio)ethane **(67)**

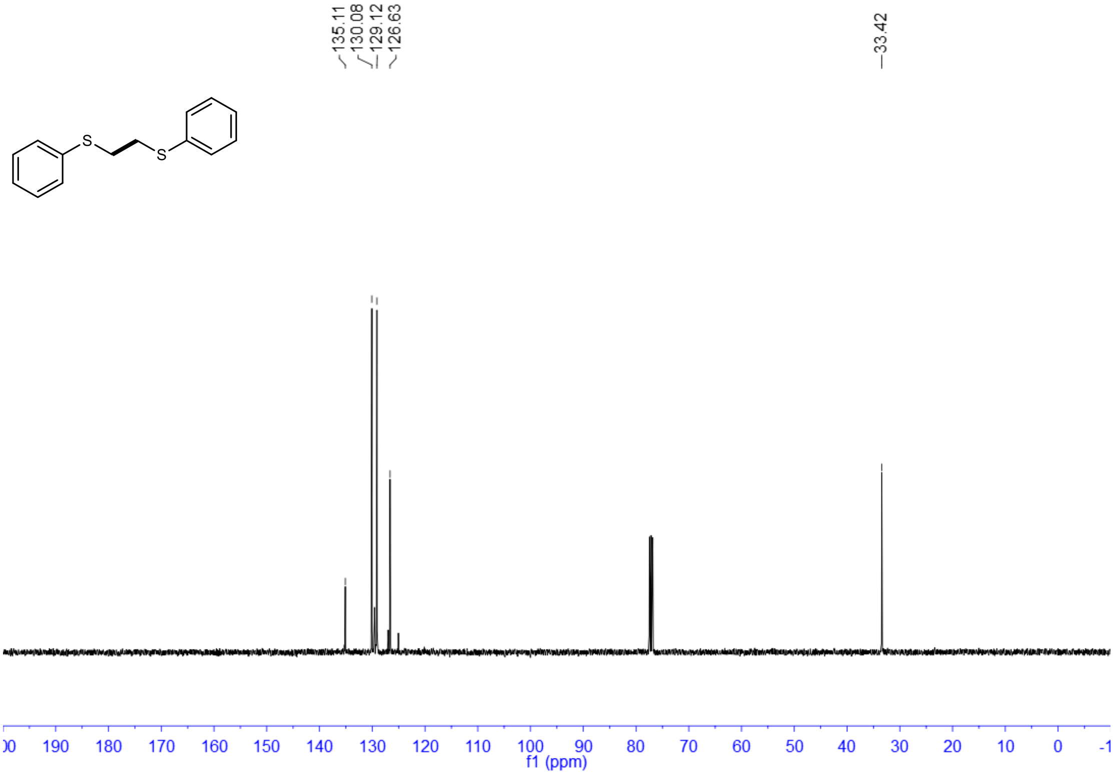

Supplementary Figure 308 | ^{1}H NMR (400 MHz, 298K, $CDCl_3$) of 2-bis(p-tolylthio)ethane **(68)**

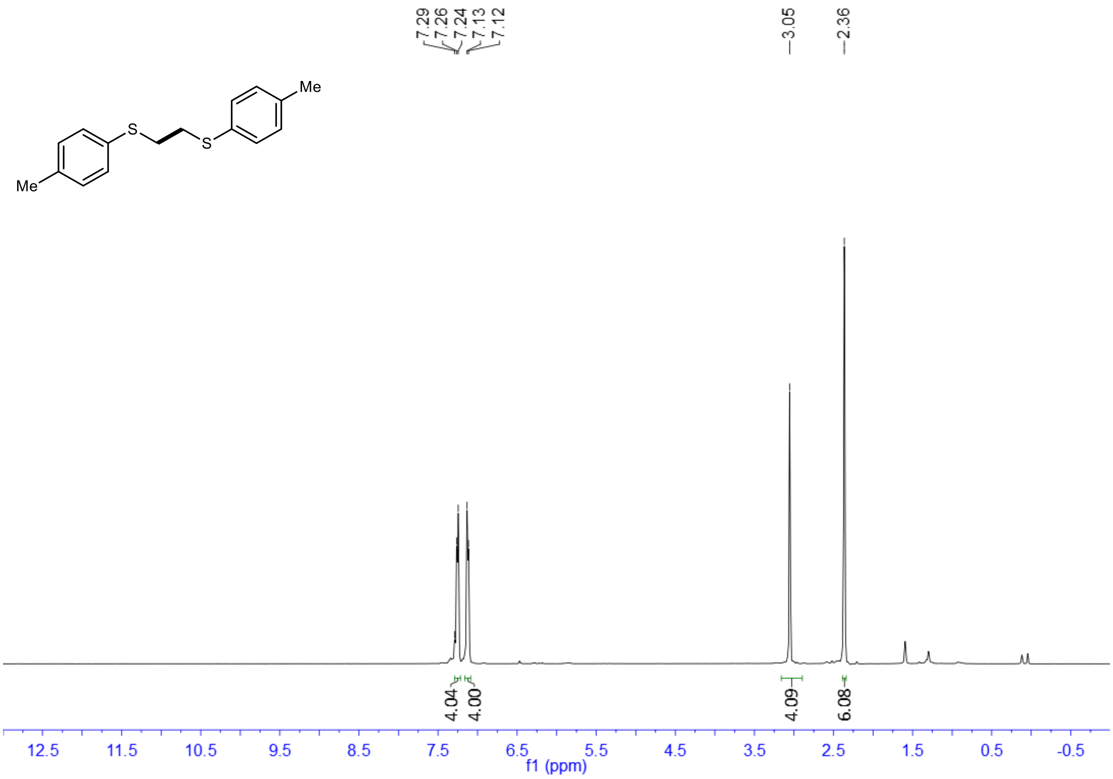

Supplementary Figure 309 | ^{13}C NMR (101 MHz, 298K, $CDCl_3$) of 2-Bis(p-tolylthio)ethane **(68)**

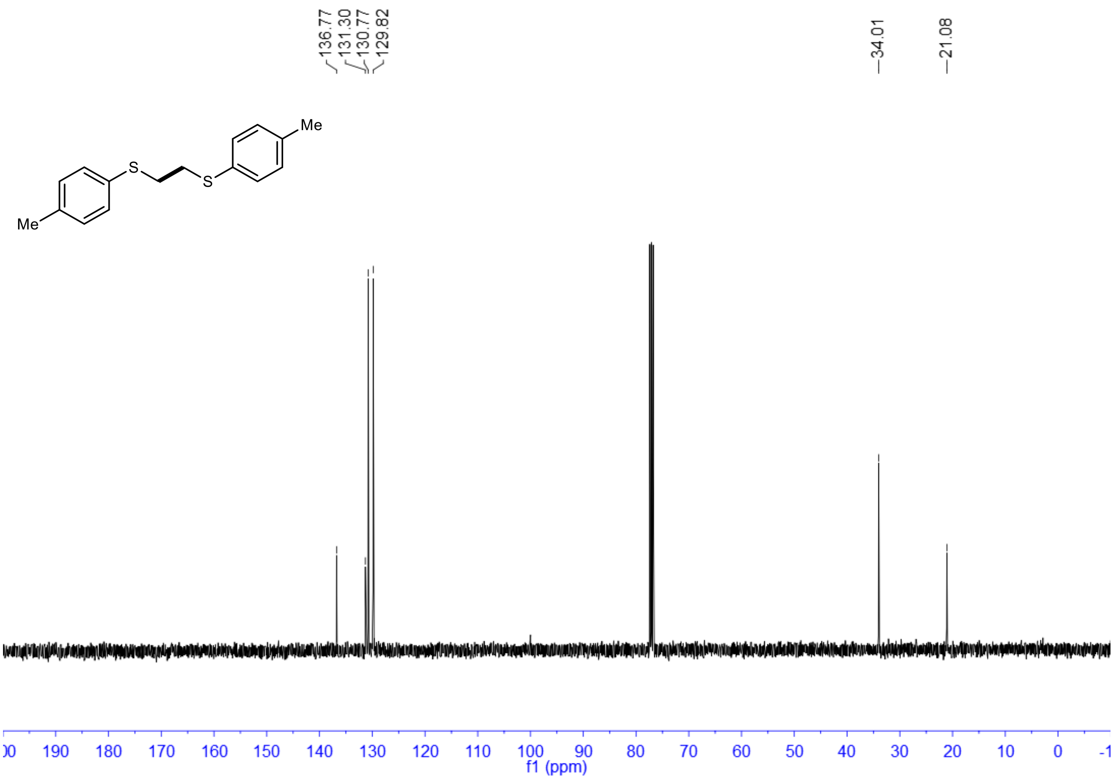

Supplementary Figure 310 | ^{1}H NMR (400 MHz, 298K, $CDCl_3$) of 1,2-Bis((4-(*tert*-butyl)phenyl)thio)ethane **(69)**

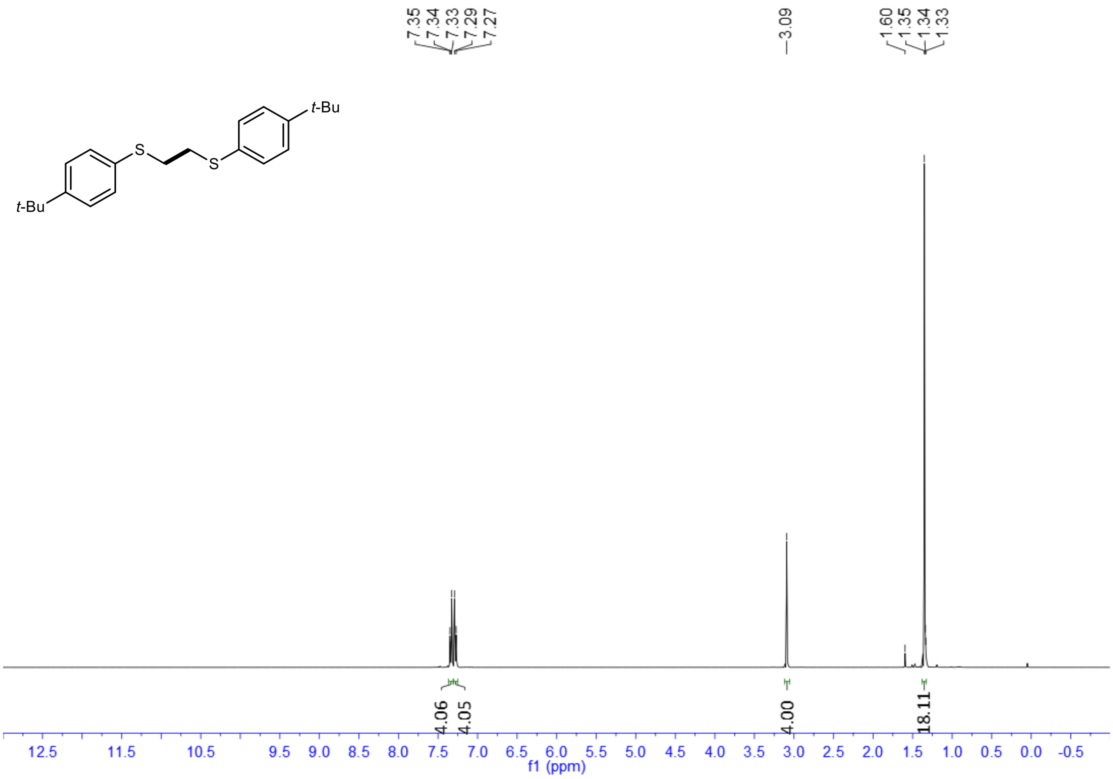

Supplementary Figure 311 | ^{13}C NMR (101 MHz, 298K, $CDCl_3$) of 1,2-bis((4-(tert-butyl)phenyl)thio)ethane **(69)**

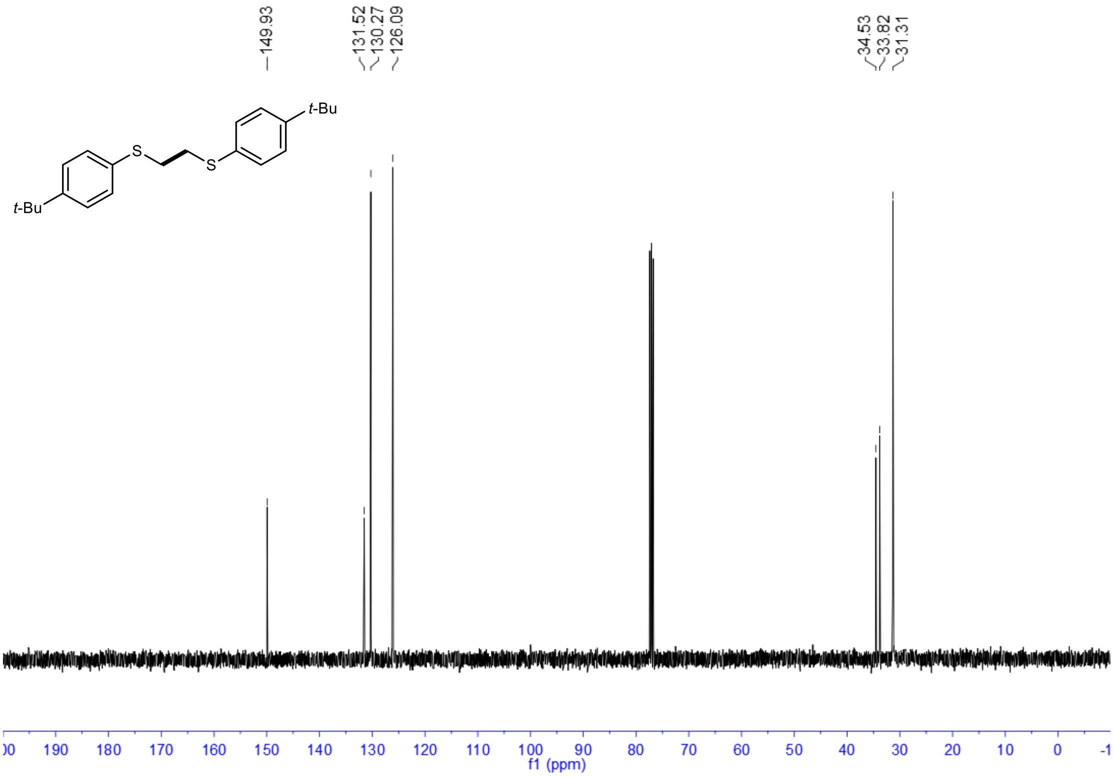

Supplementary Figure 312 | ^{1}H NMR (400 MHz, 298K, $CDCl_3$) of 1,2-Bis((4-fluorophenyl)thio)ethane **(70)**

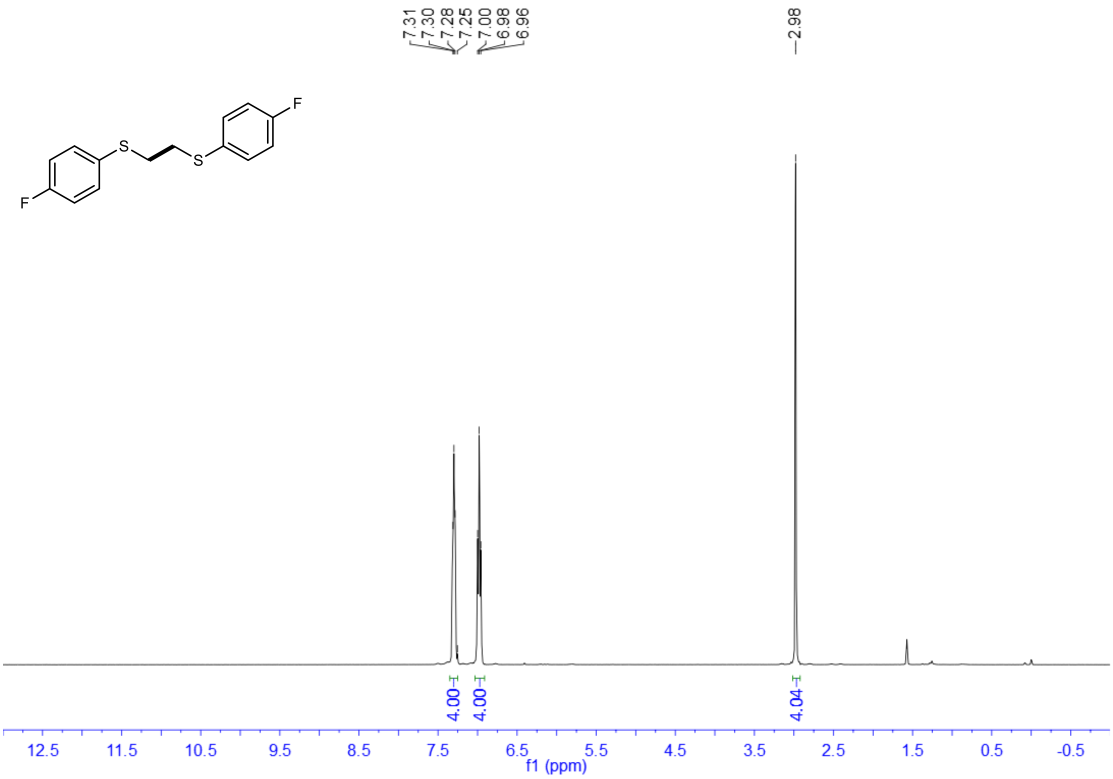

Supplementary Figure 313 | ^{13}C NMR (101 MHz, 298K, $CDCl_3$) of 1,2-bis((4-fluorophenyl)thio)ethane **(70)**

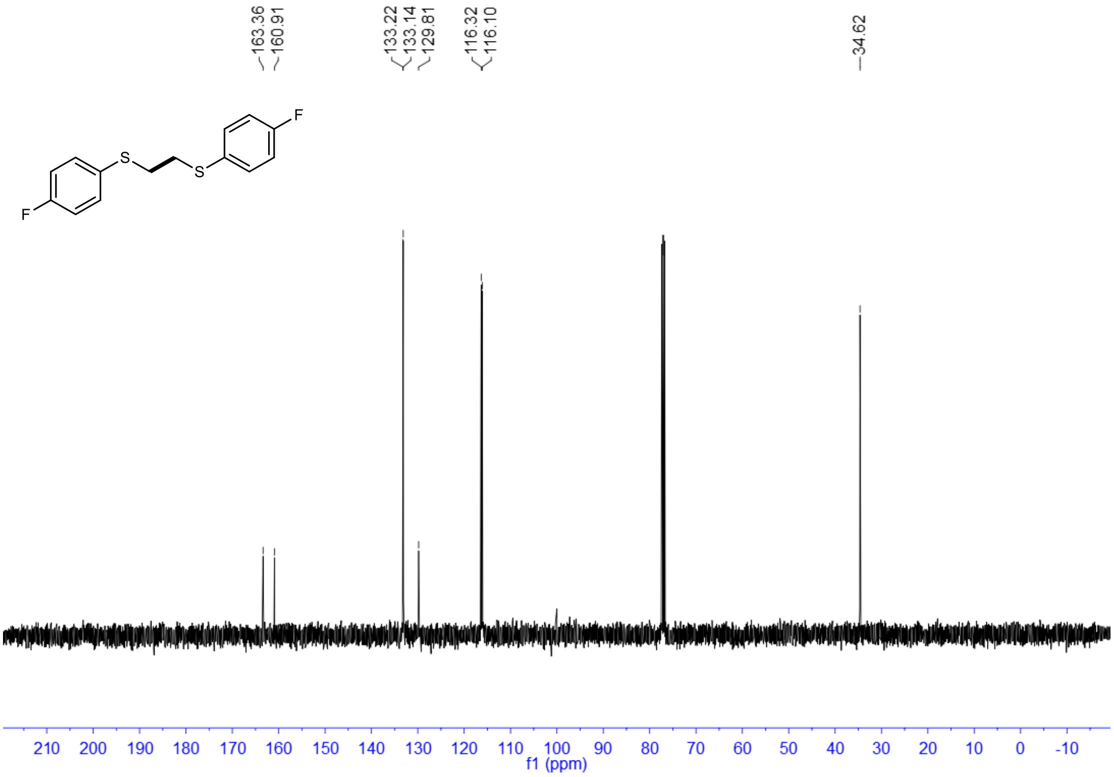

Supplementary Figure 314 | ^{19}F NMR (376 MHz MHz, 298K, $CDCl_3$) of 1,2-Bis((4-fluorophenyl)thio)ethane **(70)**

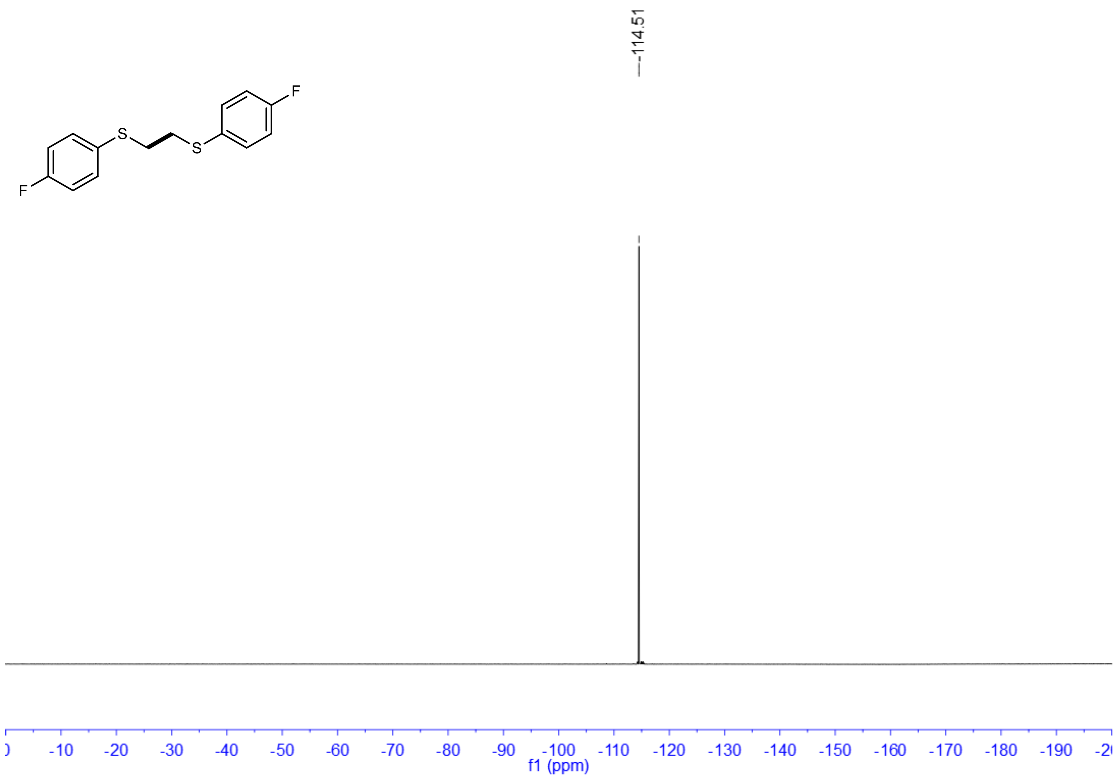

Supplementary Figure 315 | ^{1}H NMR (400 MHz, 298K, $CDCl_3$) of 1,2-Bis((4-chlorophenyl)thio)ethane **(71)**

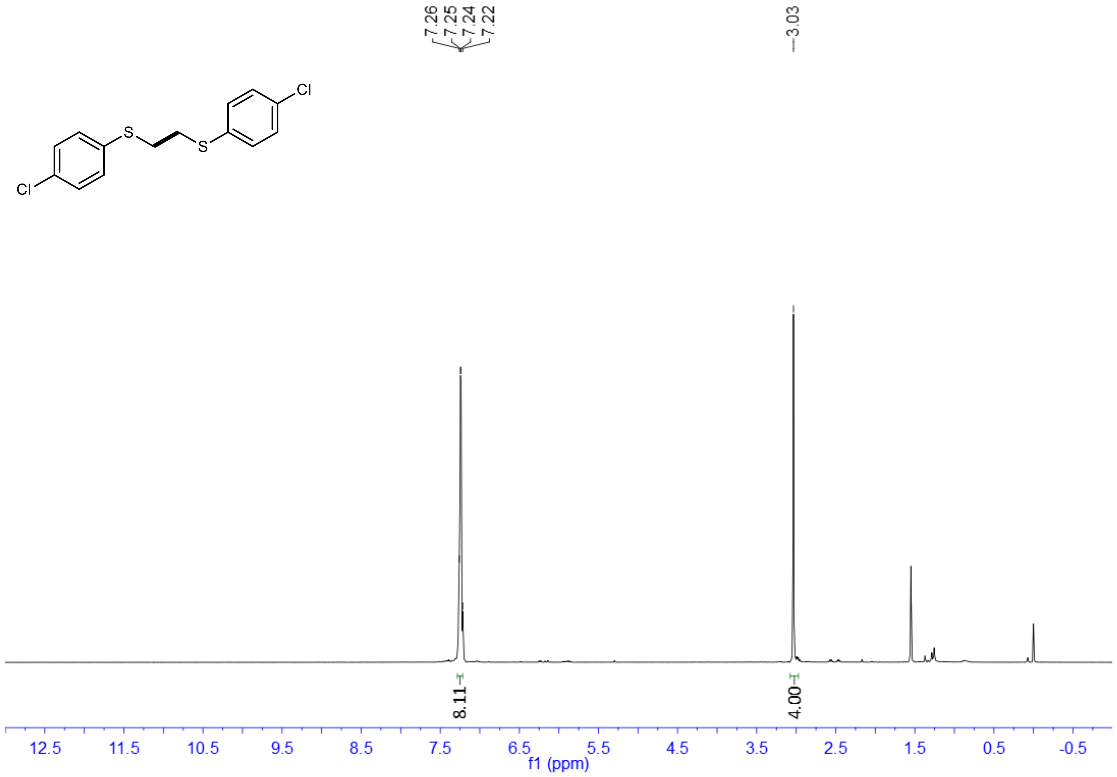

Supplementary Figure 316 | ^{13}C NMR (101 MHz, 298K, $CDCl_3$) of 1,2-Bis((4-chlorophenyl)thio)ethane **(71)**

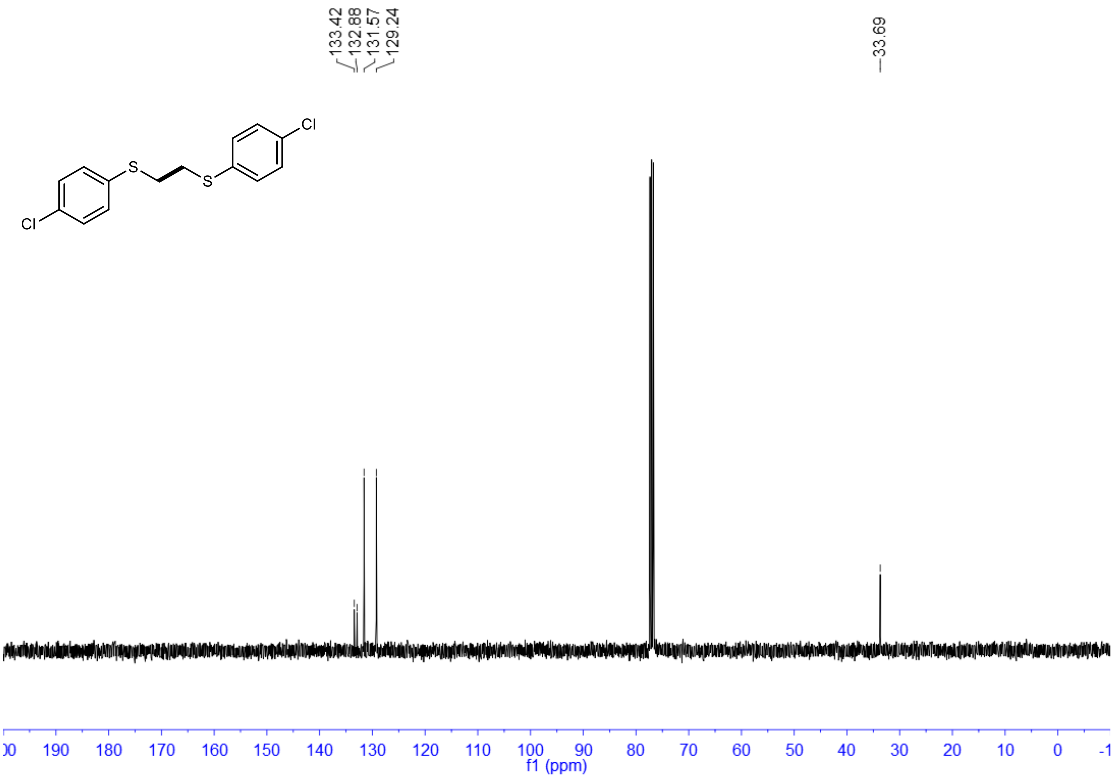

Supplementary Figure 317 | ^{1}H NMR (400 MHz, 298K, $CDCl_3$) of 1,2-Bis((4-bromophenyl)thio)ethane **(72)**

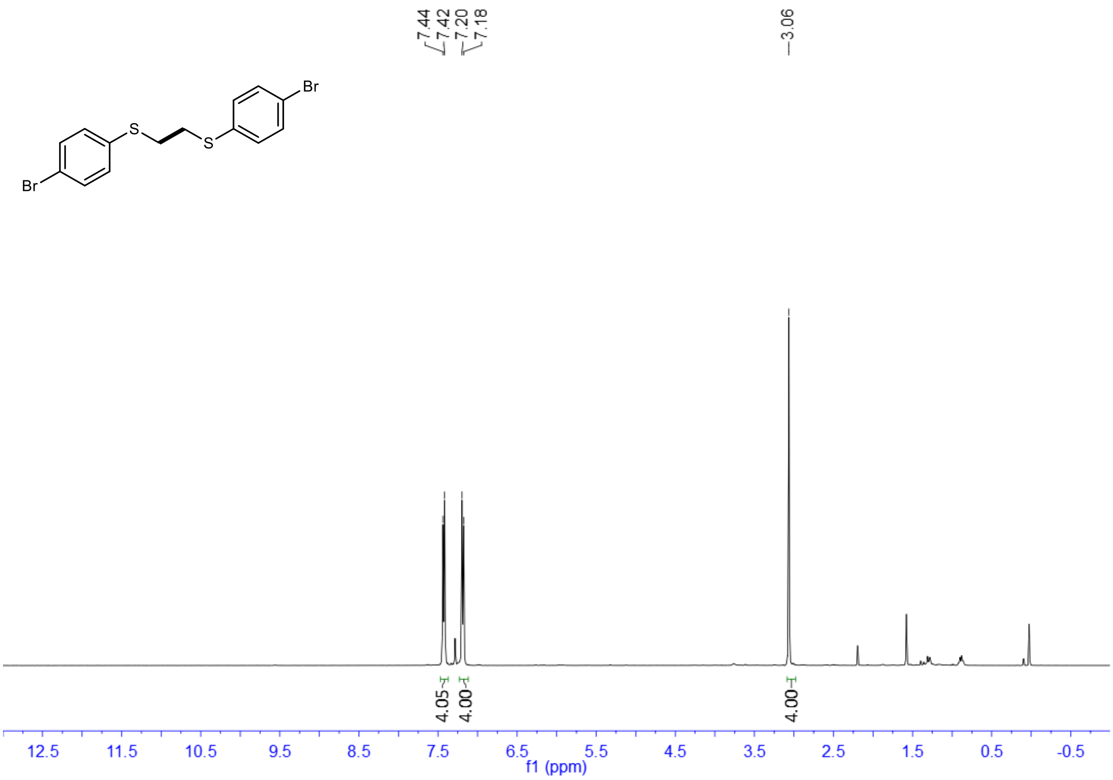

Supplementary Figure 318 | ^{13}C NMR (101 MHz, 298K, $CDCl_3$) of 1,2-Bis((4-bromophenyl)thio)ethane **(72)**

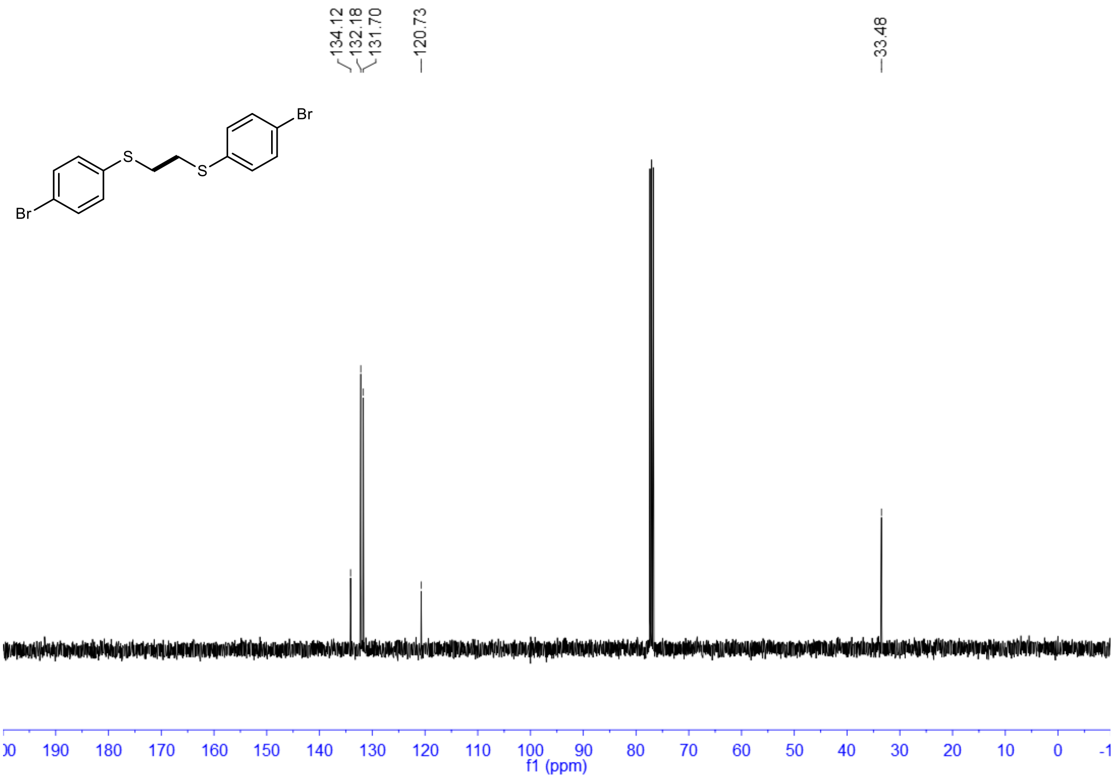

Supplementary Figure 319 | ^{1}H NMR (400 MHz, 298K, $CDCl_3$) of 1,2-Bis((furan-2-ylmethyl)thio)ethane **(73)**

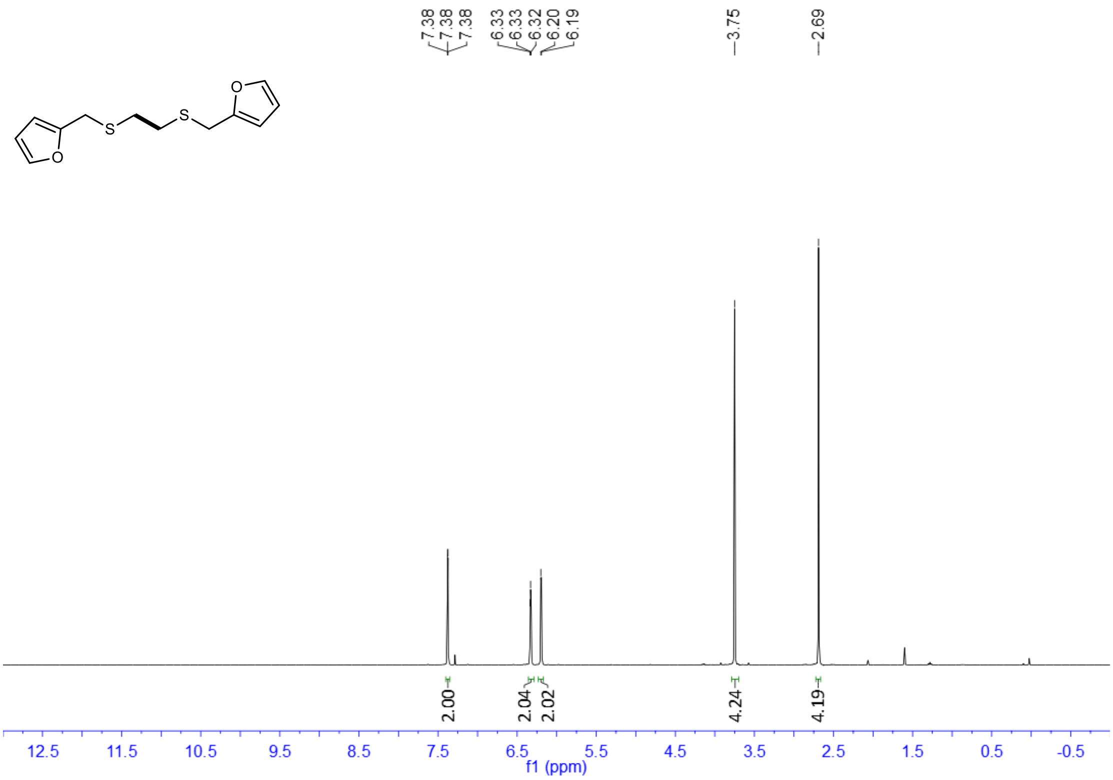

Supplementary Figure 320 | ^{13}C NMR (101 MHz, 298K, $CDCl_3$) of 1,2-Bis((furan-2-ylmethyl)thio)ethane **(73)**

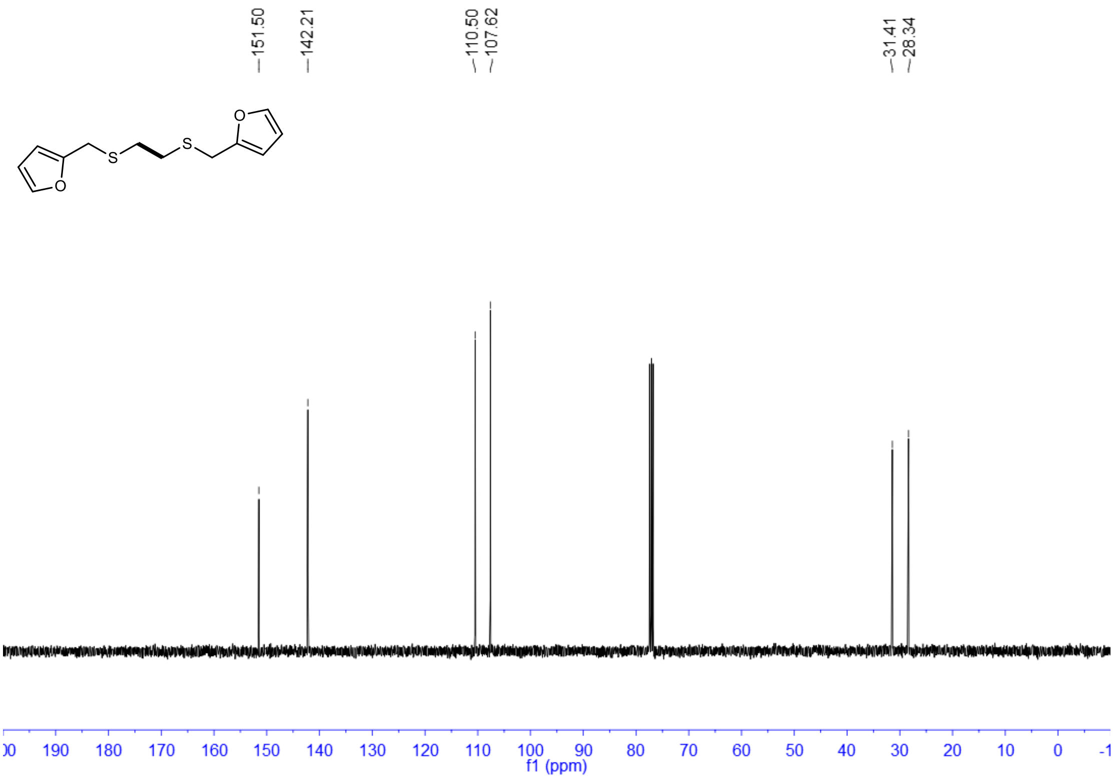

Supplementary Figure 321 | ^{1}H NMR (400 MHz, 298K, $CDCl_3$) of 1,2-Bis((2-(pyrazin-2-yl)ethyl)thio)ethane **(74)**

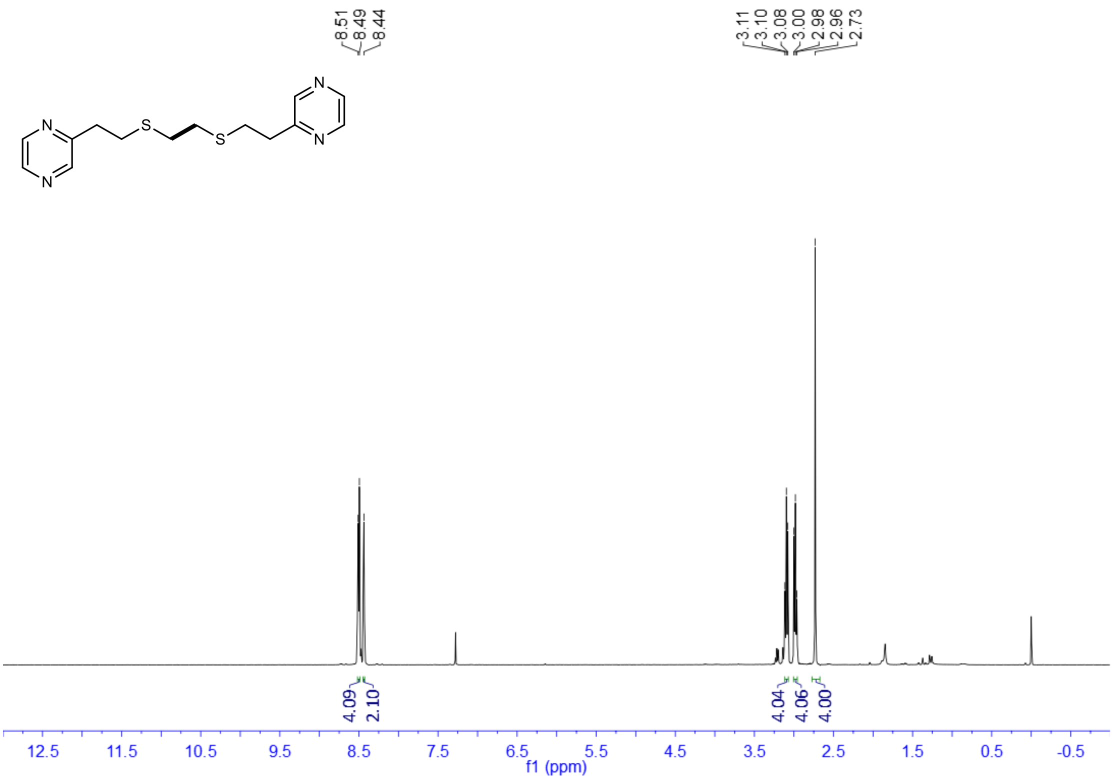

Supplementary Figure 322 | ^{13}C NMR (101 MHz, 298K, $CDCl_3$) of 1,2-Bis((2-(pyrazin-2-yl)ethyl)thio)ethane **(74)**

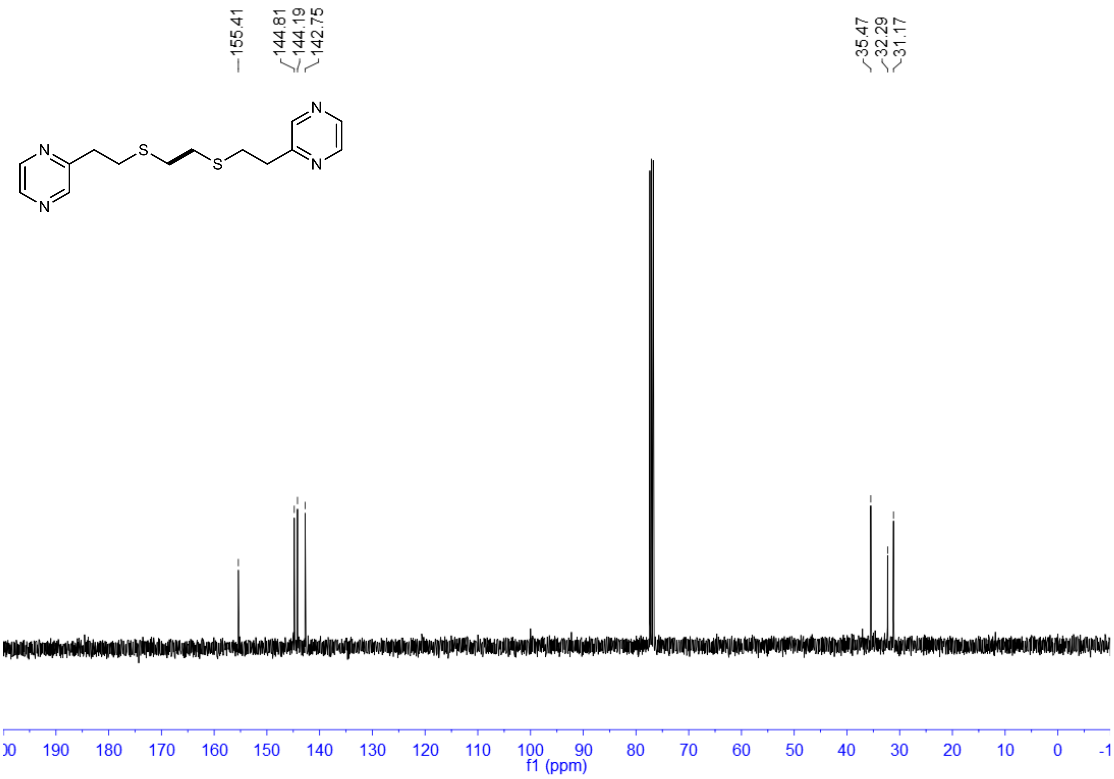

Supplementary Figure 323 | ^{1}H NMR (400 MHz, 298K, $CDCl_3$) of 1,2-Bis((4-(4,4,5,5-tetramethyl-1,3,2-dioxaborolan-2-yl)benzyl)thio)ethane **(75)**

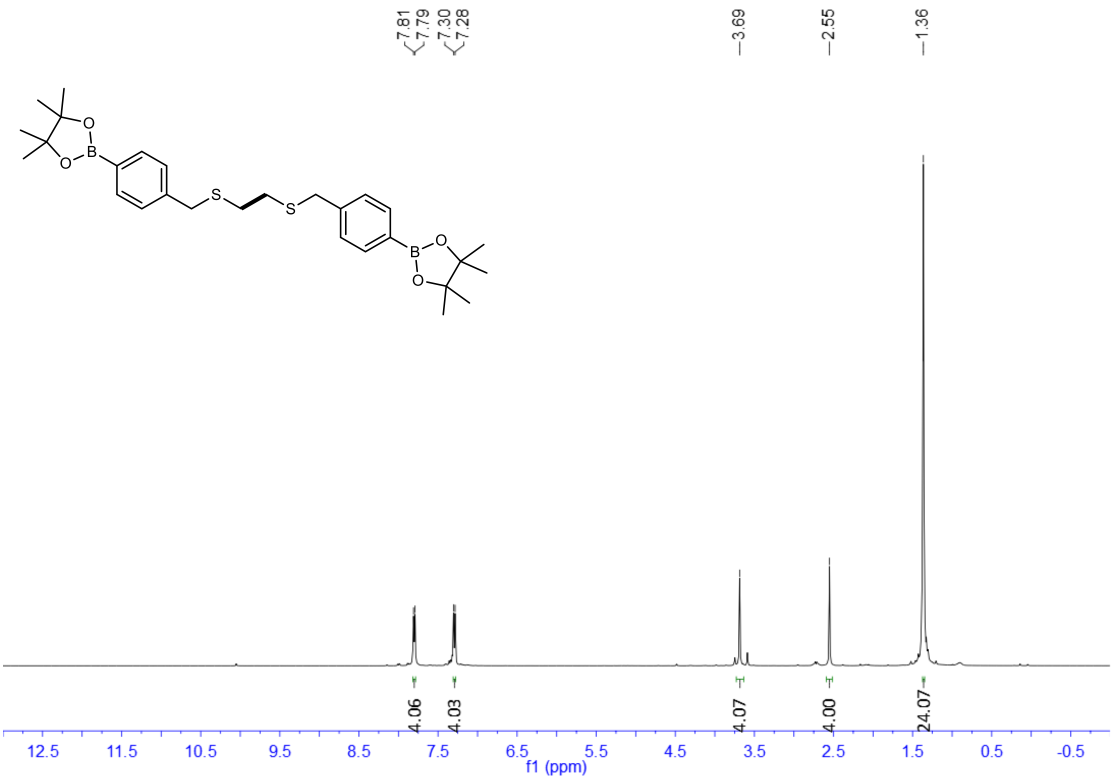

Supplementary Figure 324 | ^{13}C NMR (101 MHz, 298K, $CDCl_3$) of 1,2-Bis((4-(4,4,5,5-tetramethyl-1,3,2-dioxaborolan-2-yl)benzyl)thio)ethane **(75)**

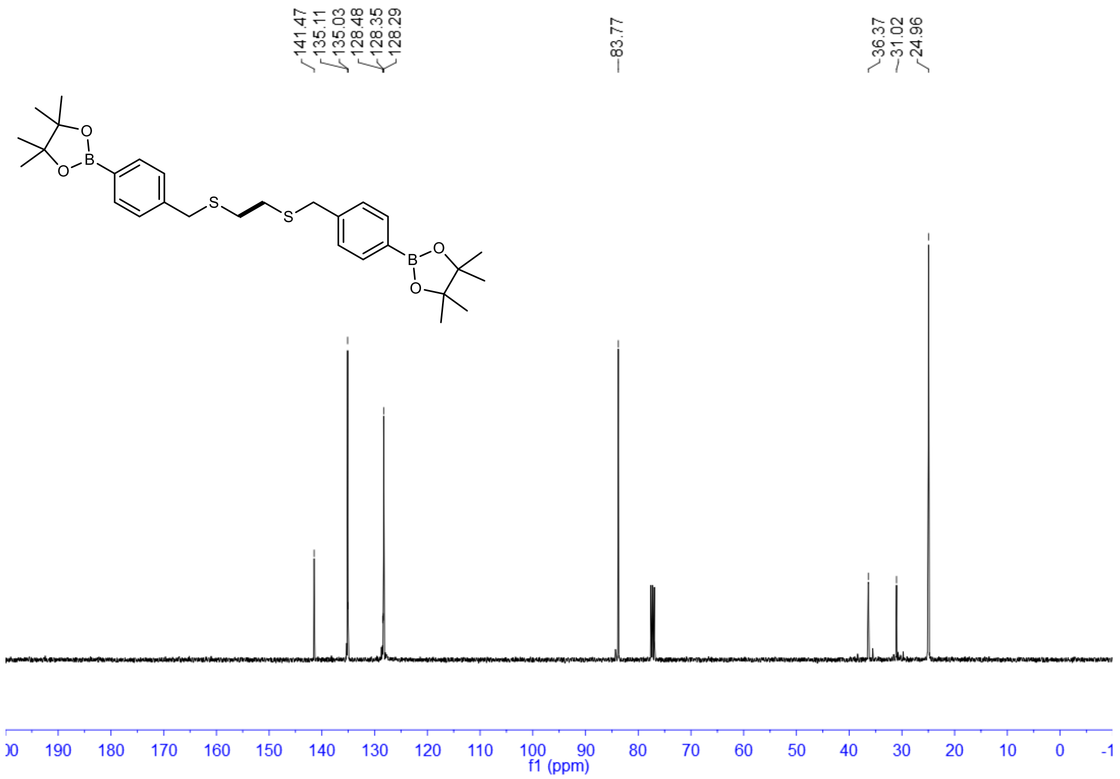

Supplementary Figure 325 | ^{1}H NMR (500 MHz, 298K, $CDCl_3$) of 4,4,13,13-Tetraethoxy-3,14-dioxa-7,10-dithia-4,13-disilahexadecane **(76)**

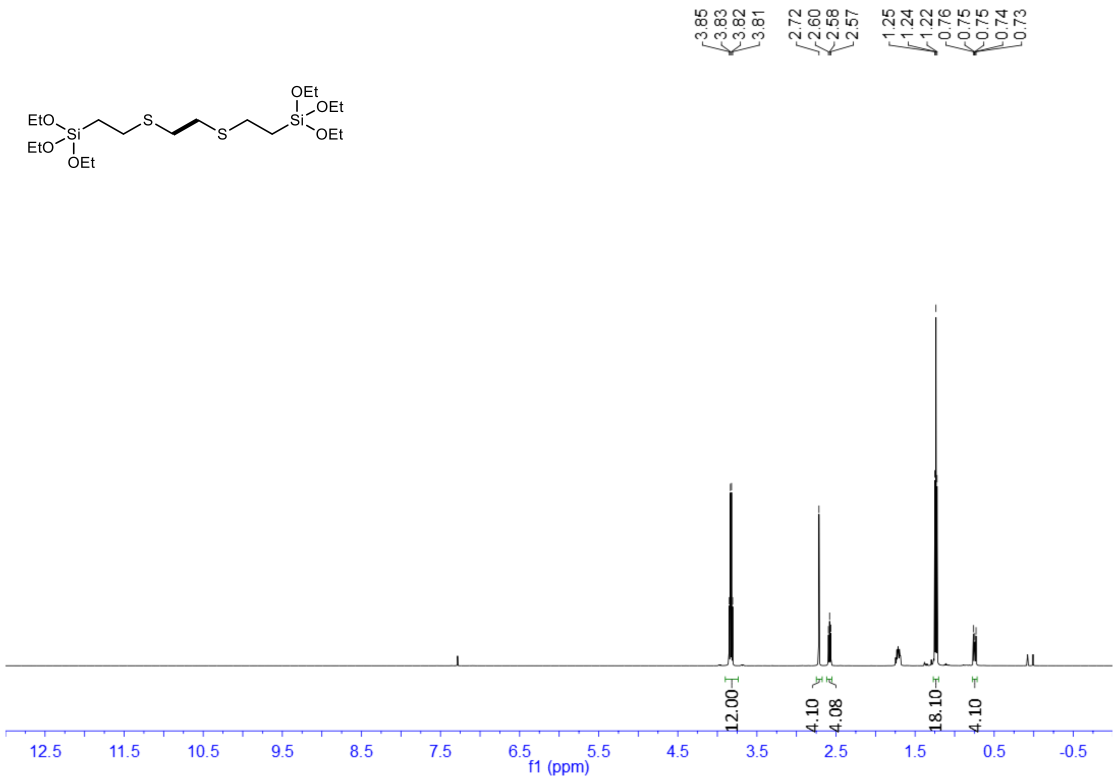

Supplementary Figure 326 | ^{13}C NMR (126 MHz, 298K, $CDCl_3$) of 4,4,13,13-Tetraethoxy-3,14-dioxa-7,10-dithia-4,13-disilahexadecane **(76)**

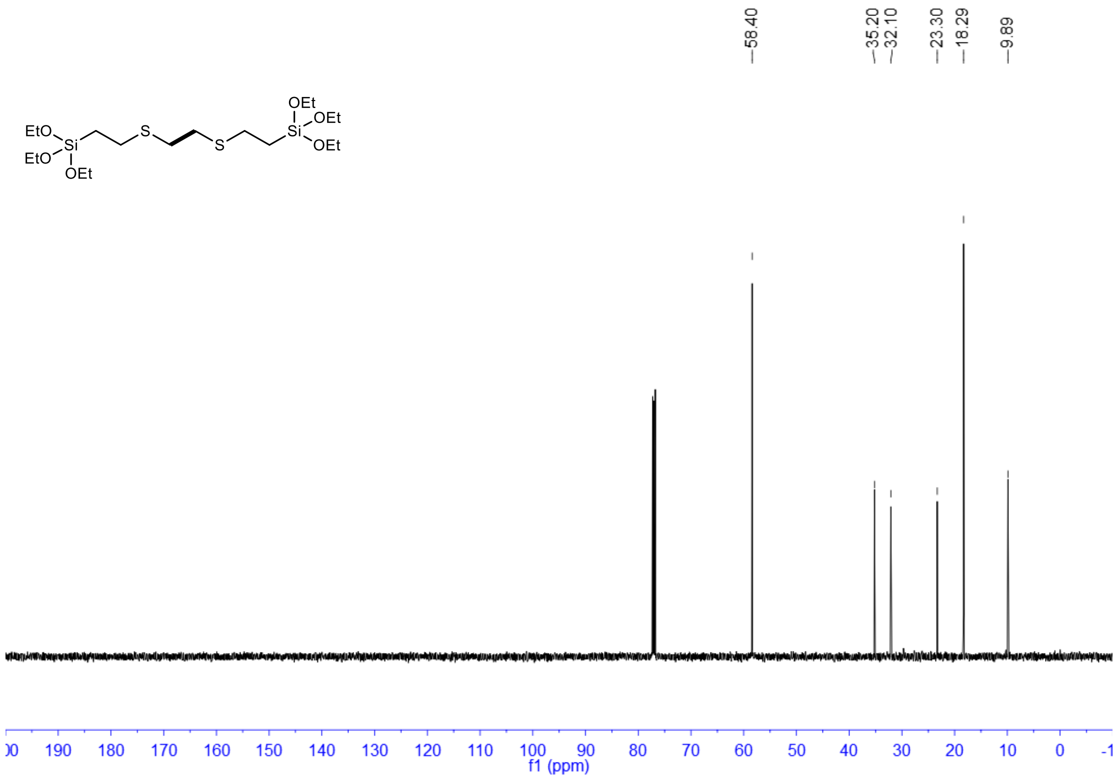

Supplementary Figure 327 | ^{1}H NMR (400 MHz, 298K, $CDCl_3$) of Diethyl 2,2'-(ethane-1,2-diylbis(sulfanediyl))diacetate **(77)**

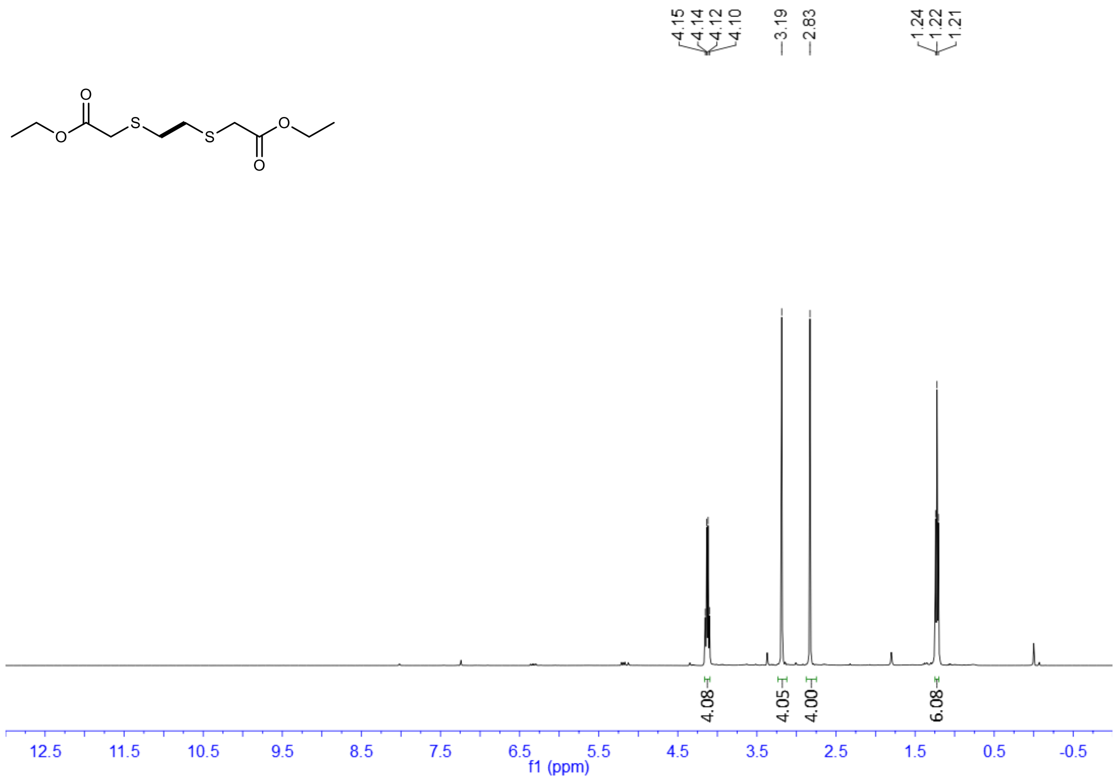

Supplementary Figure 328 | ^{13}C NMR (101 MHz, 298K, $CDCl_3$) of Diethyl 2,2'-(ethane-1,2-diylbis(sulfanediyl))diacetate **(77)**

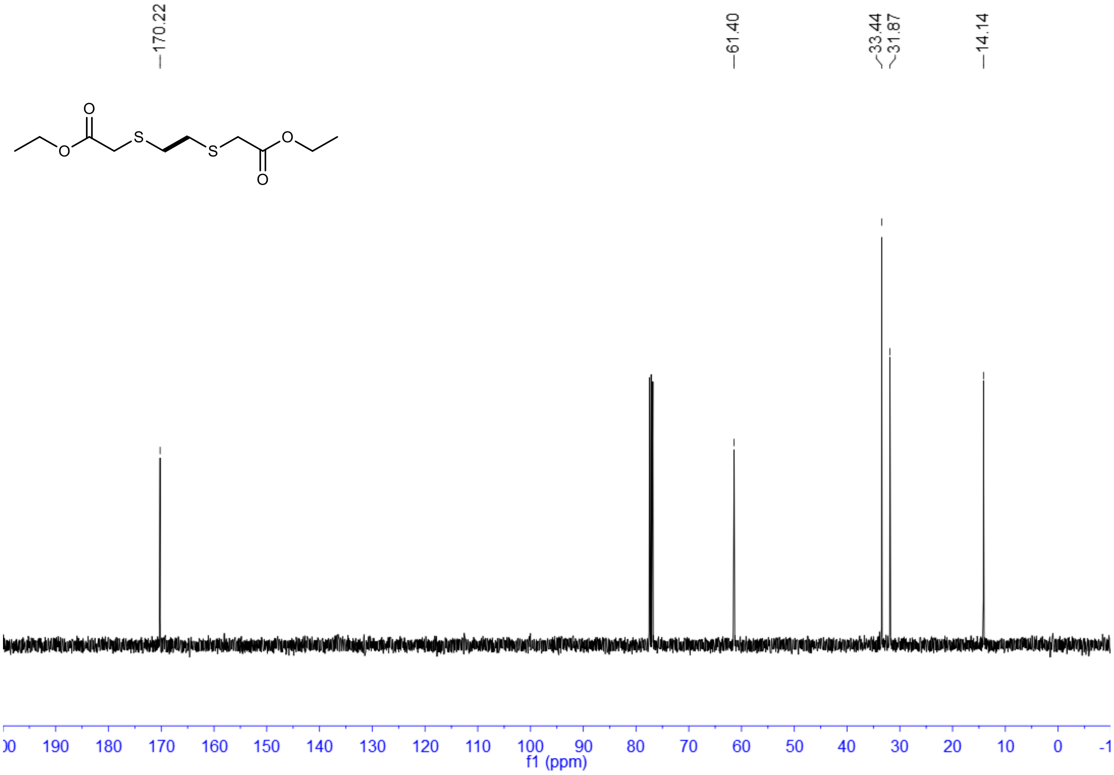

Supplementary Figure 329 | ^{1}H NMR (400 MHz, 298K, $CDCl_3$) of *S*,*S'*-(ethane-1,2-diyl) diethanethioate **(78)**

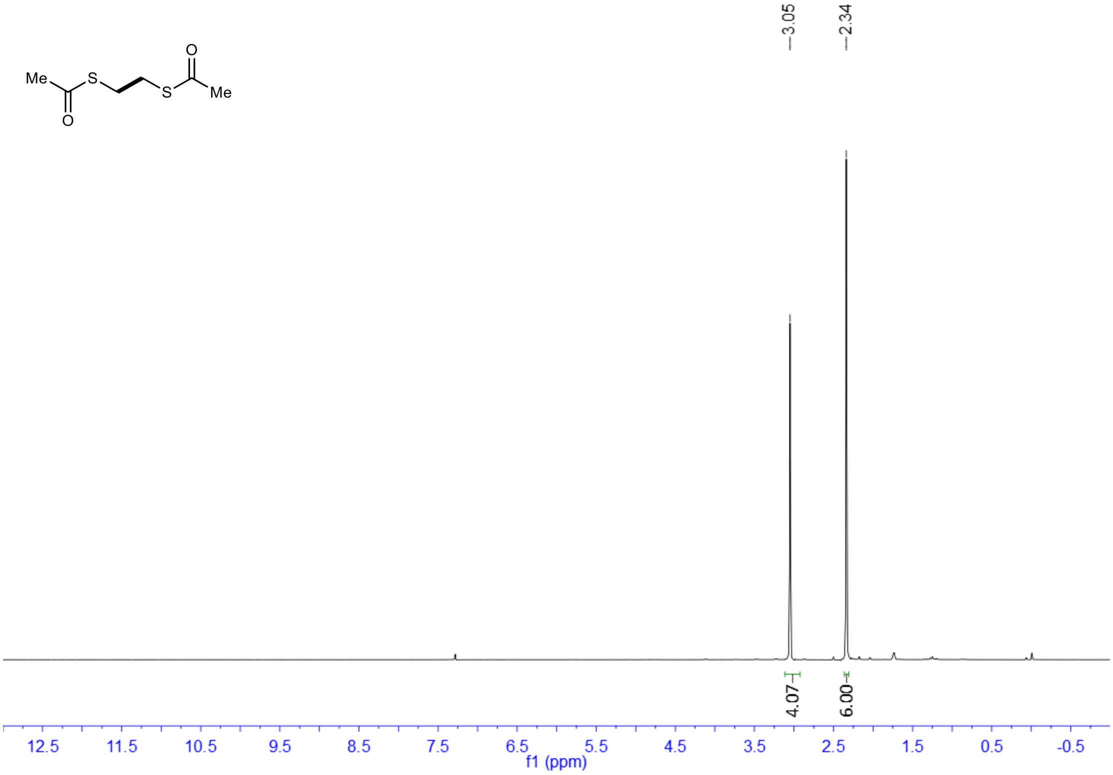

Supplementary Figure 330 | ^{13}C NMR (101 MHz, 298K, $CDCl_3$) of *S*,*S'*-(ethane-1,2-diyl) diethanethioate **(78)**

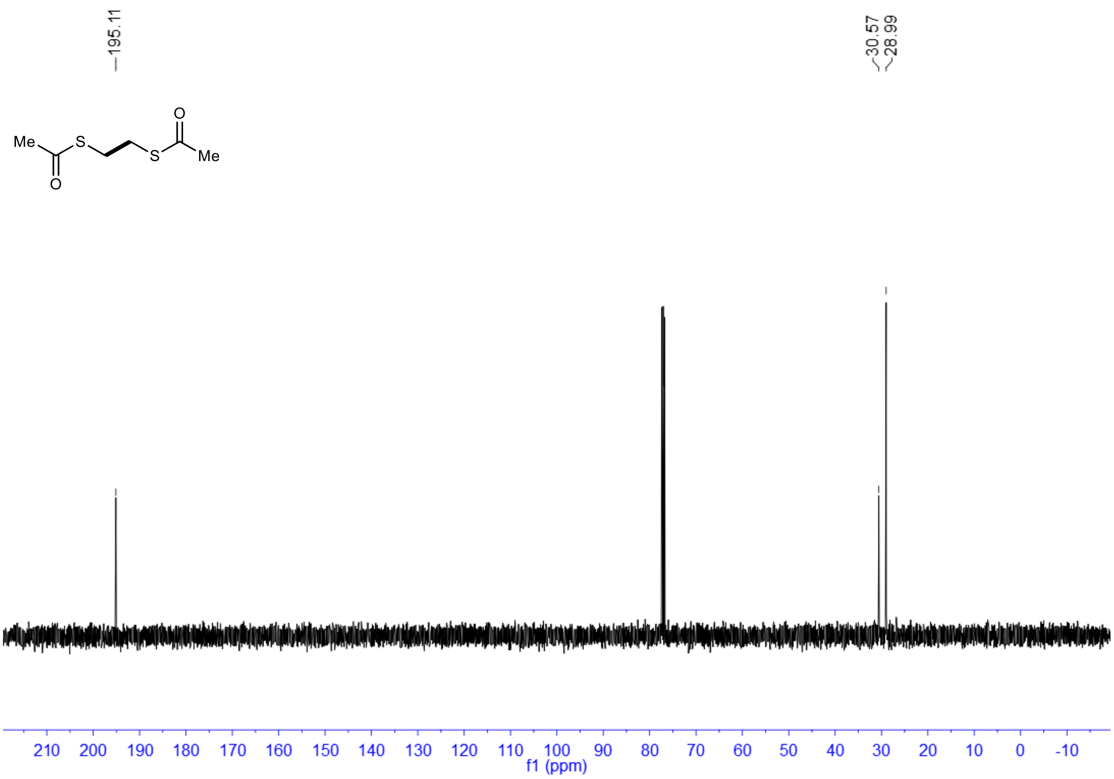

Supplementary Figure 331 | ^{1}H NMR (400 MHz, 298K, $CDCl_3$) of (ethane-1,2-diylbis(sulfanediyl))Bis(3-methylbutane-3,1-diyl) diformate **(79)**

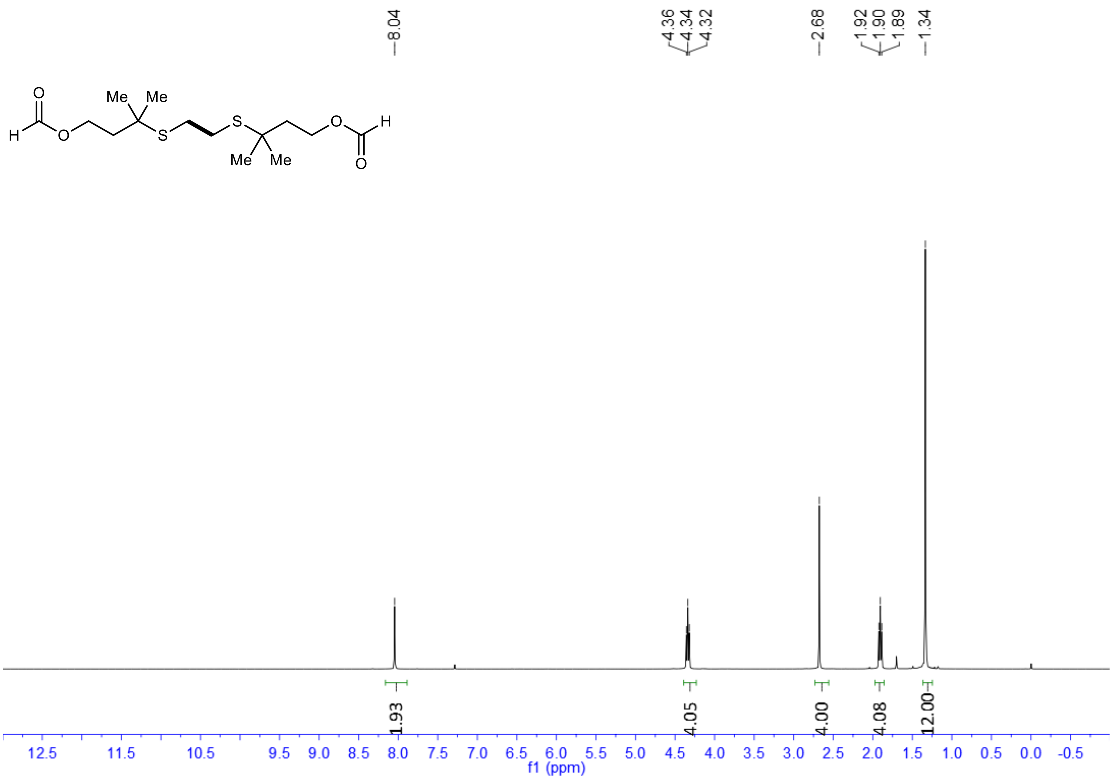

Supplementary Figure 332 | ^{13}C NMR (101 MHz, 298K, $CDCl_3$) of (ethane-1,2-diylbis(sulfanediyl))Bis(3-methylbutane-3,1-diyl) diformate **(79)**

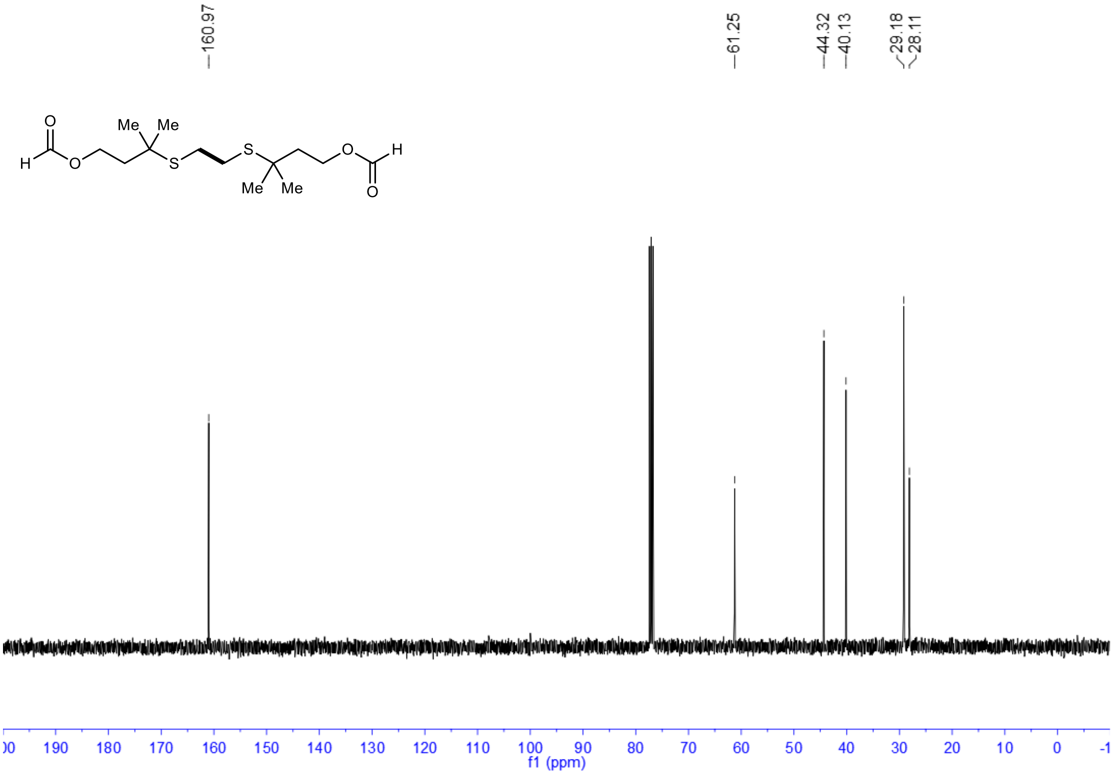

Supplementary Figure 333 | ^{1}H NMR (400 MHz, 298K, $CDCl_3$) of 4,4'-(ethane-1,2-diylbis(sulfanediyl))Bis(4-methylpentan-2-one) **(80)**

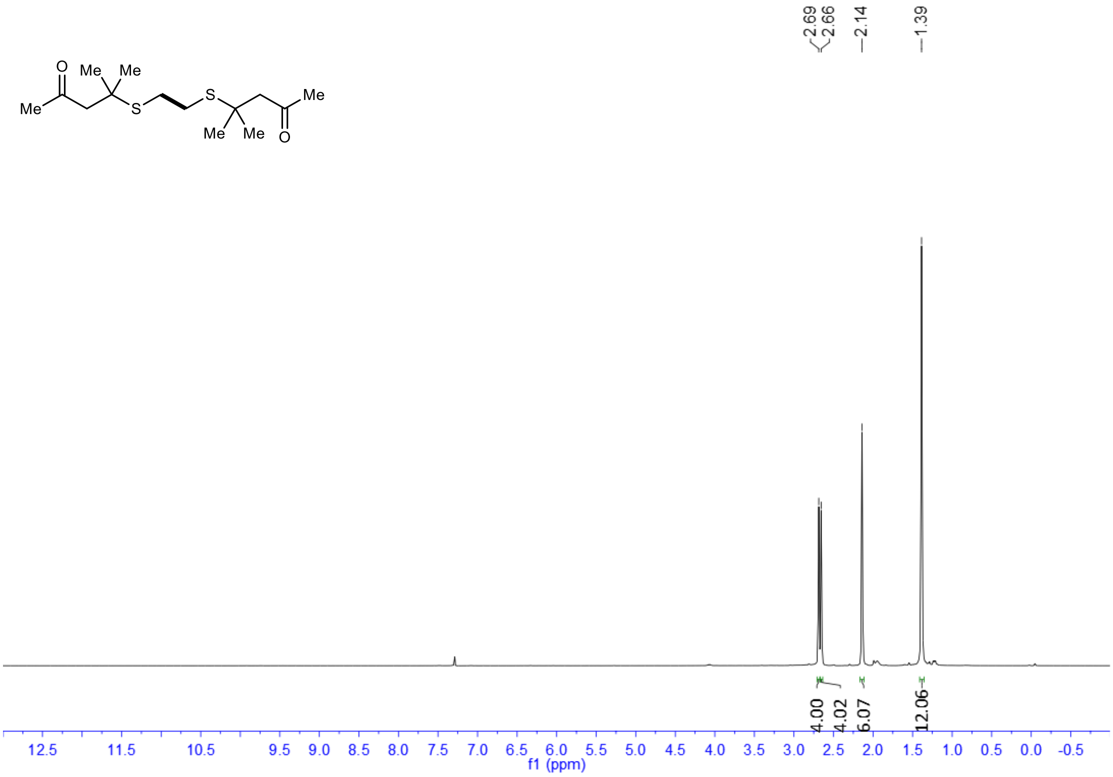

Supplementary Figure 334 | ^{13}C NMR (101 MHz, 298K, $CDCl_3$) of 4,4'-(ethane-1,2-diylbis(sulfanediyl))Bis(4-methylpentan-2-one) **(80)**

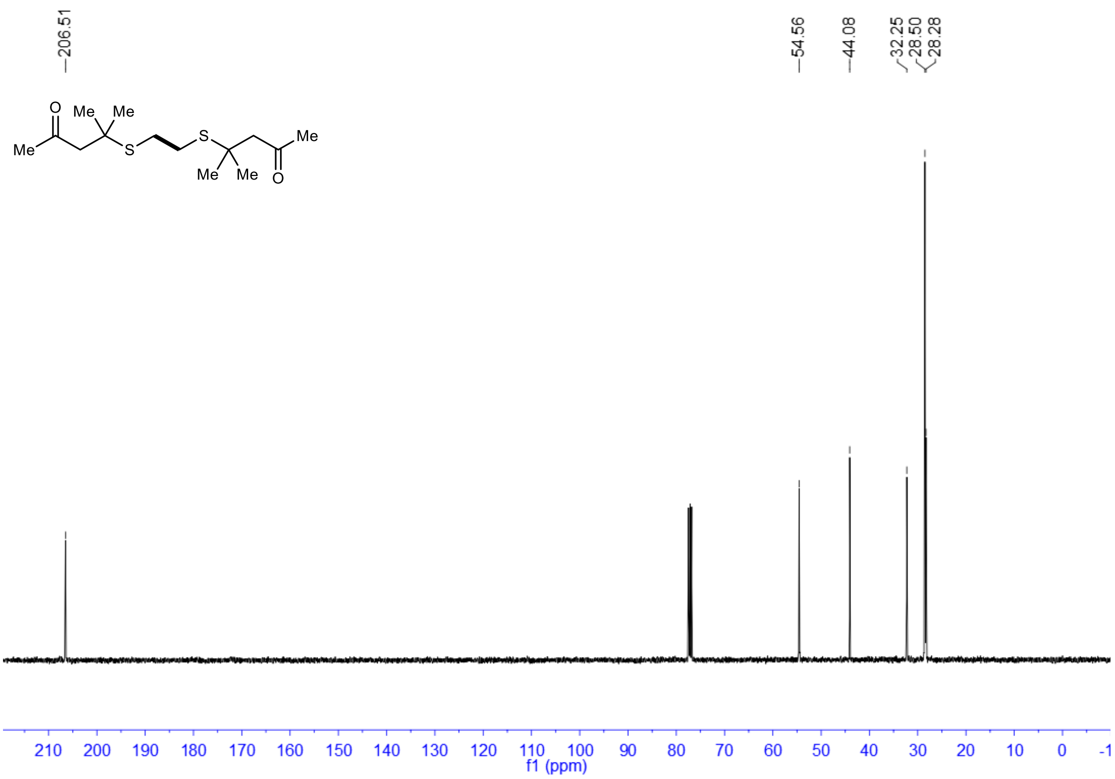

Supplementary Figure 335 | ^{1}H NMR (400 MHz, 298K, $CDCl_3$) of 2,2'-(ethane-1,2-diylbis(sulfanediyl))bis(ethan-1-ol) **(81)**

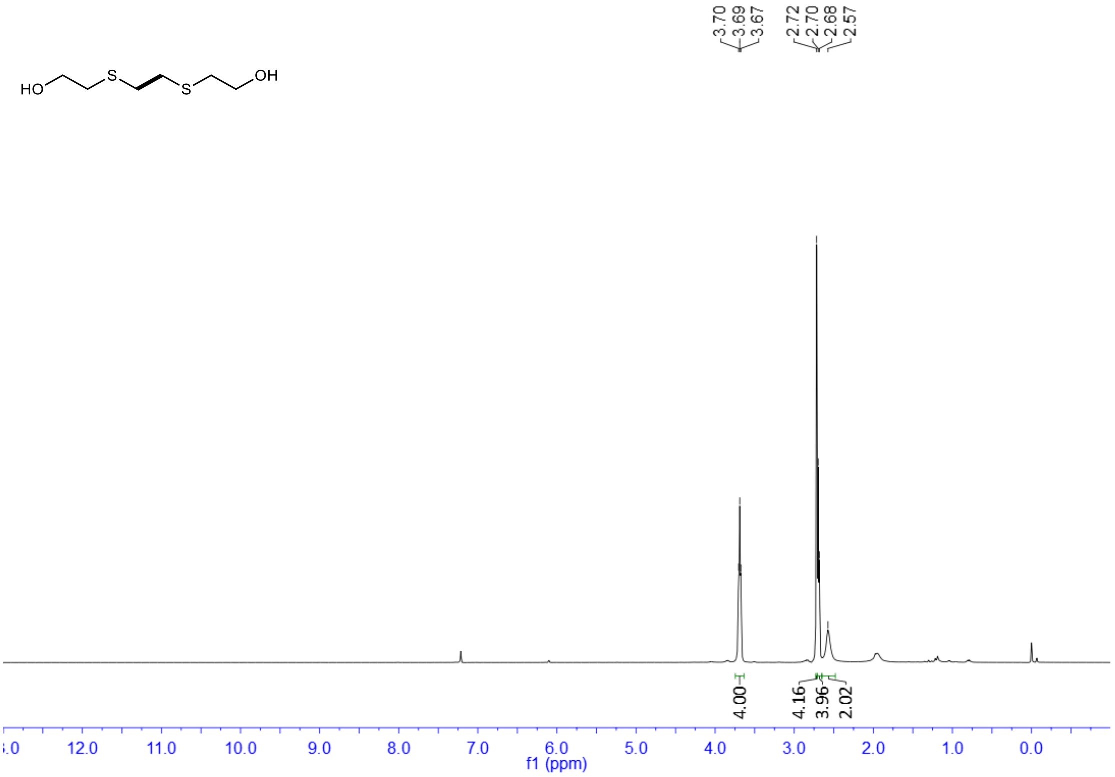

Supplementary Figure 336 | ^{13}C NMR (101 MHz, 298K, $CDCl_3$) of 2,2'-(ethane-1,2-diylbis(sulfanediyl))Bis(ethan-1-ol) **(81)**

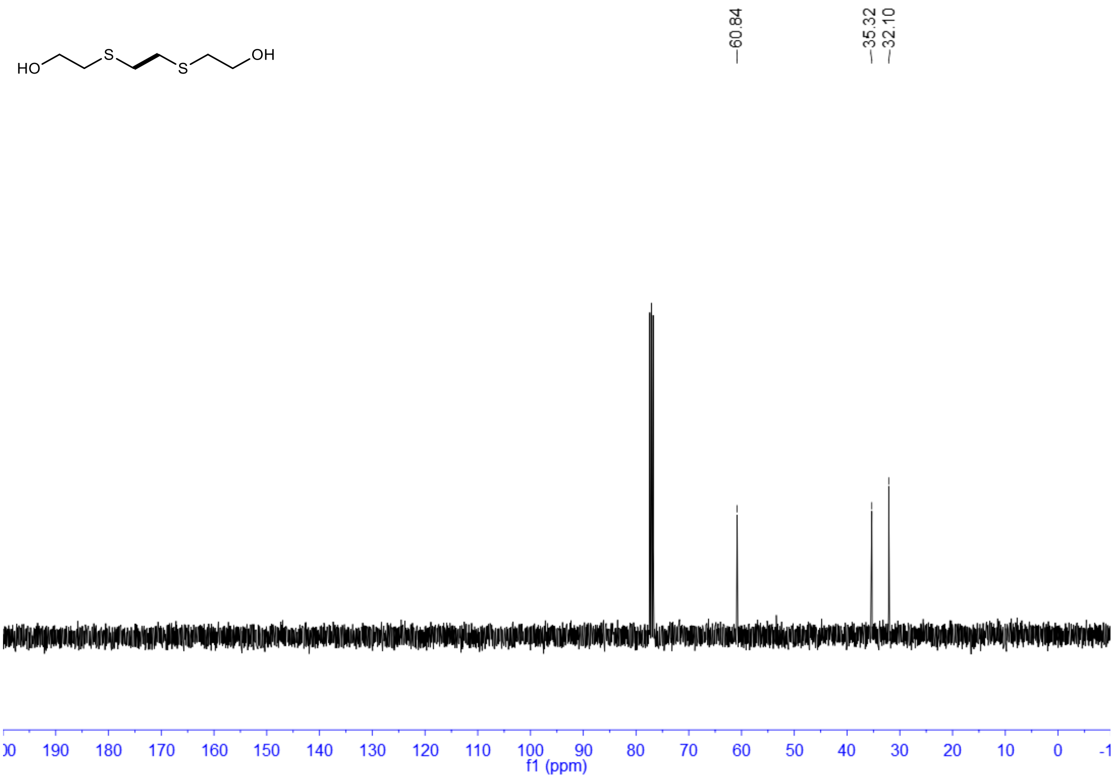

Supplementary Figure 337 | ^{1}H NMR (400 MHz, 298K, d_6-DMSO) of 3,3'-(ethane-1,2-diylbis(sulfanediyl))Dipropionic acid **(82)**

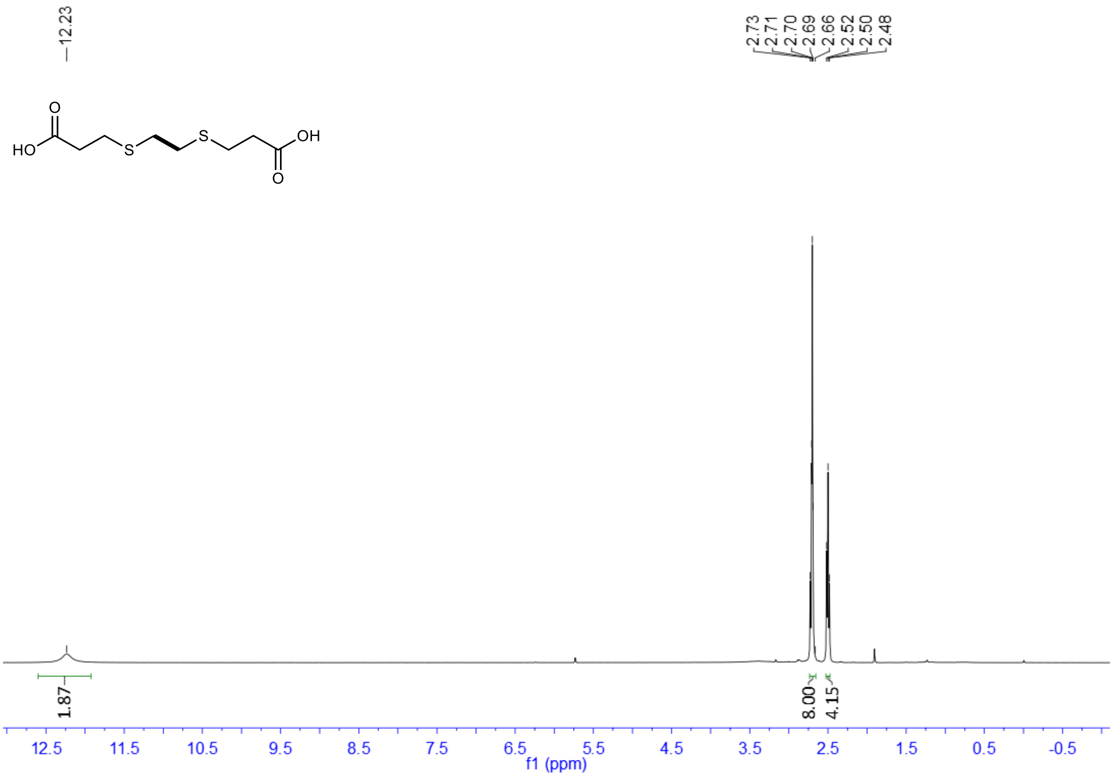

Supplementary Figure 338 | ^{13}C NMR (101 MHz, 298K, d_6-DMSO) of 3,3'-(ethane-1,2-diylbis(sulfanediyl))Dipropionic acid **(82)**

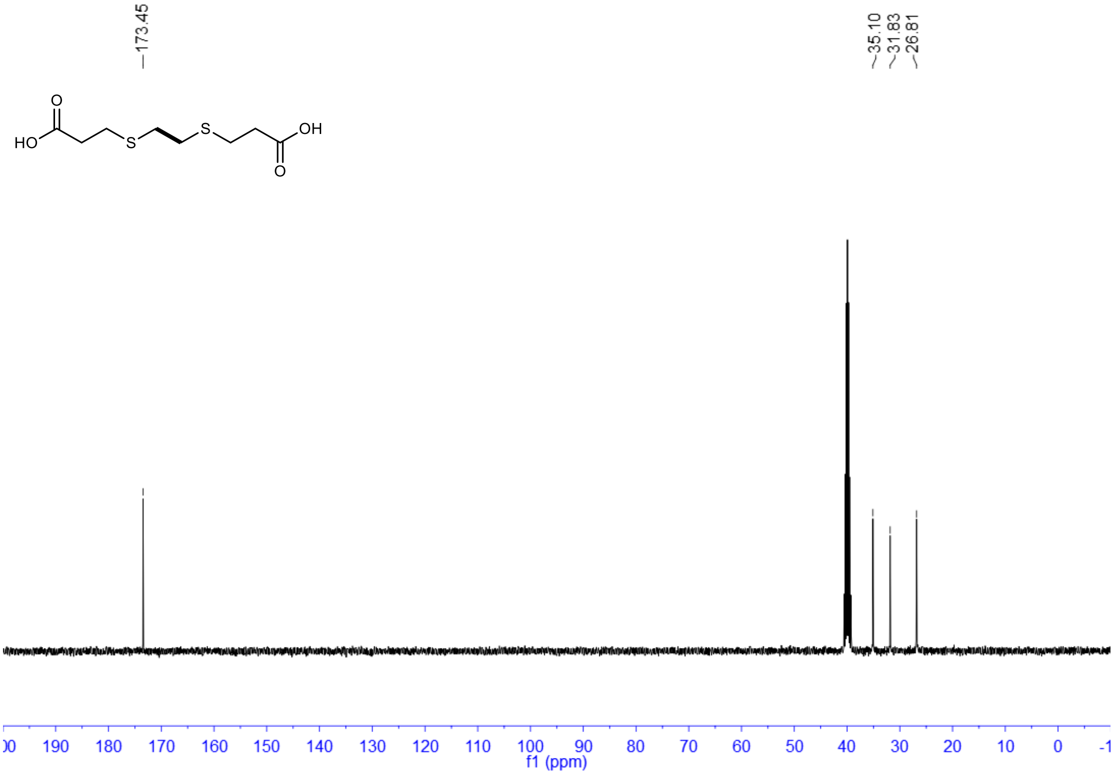

Supplementary Figure 339 | ^{1}H NMR (400 MHz, 298K, $CDCl_3$) of 1,2-Bis(((3*S*,5*S*,7*S*)-adamantan-1-yl)thio)ethane **(83)**

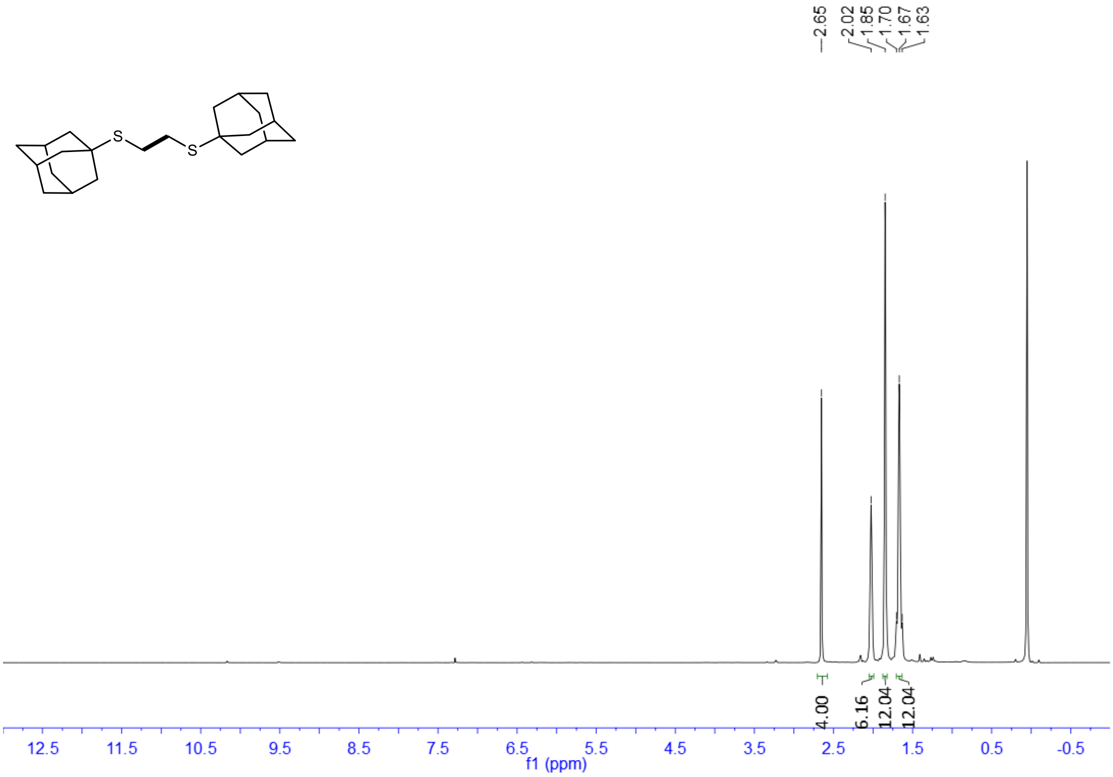

Supplementary Figure 340 | ^{13}C NMR (101 MHz, 298K, $CDCl_3$) of 1,2-Bis(((3*S*,5*S*,7*S*)-adamantan-1-yl)thio)ethane **(83)**

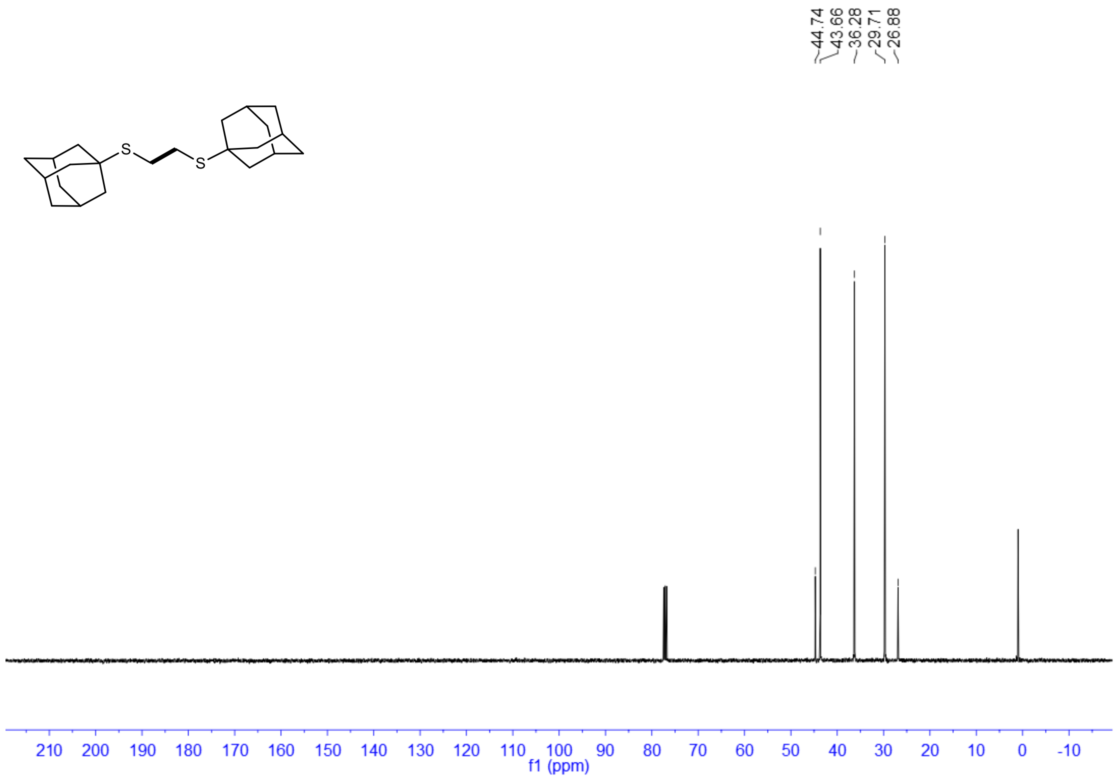

Supplementary Figure 341 | ^{1}H NMR (400 MHz, 298K, $CDCl_3$) of **(84)**

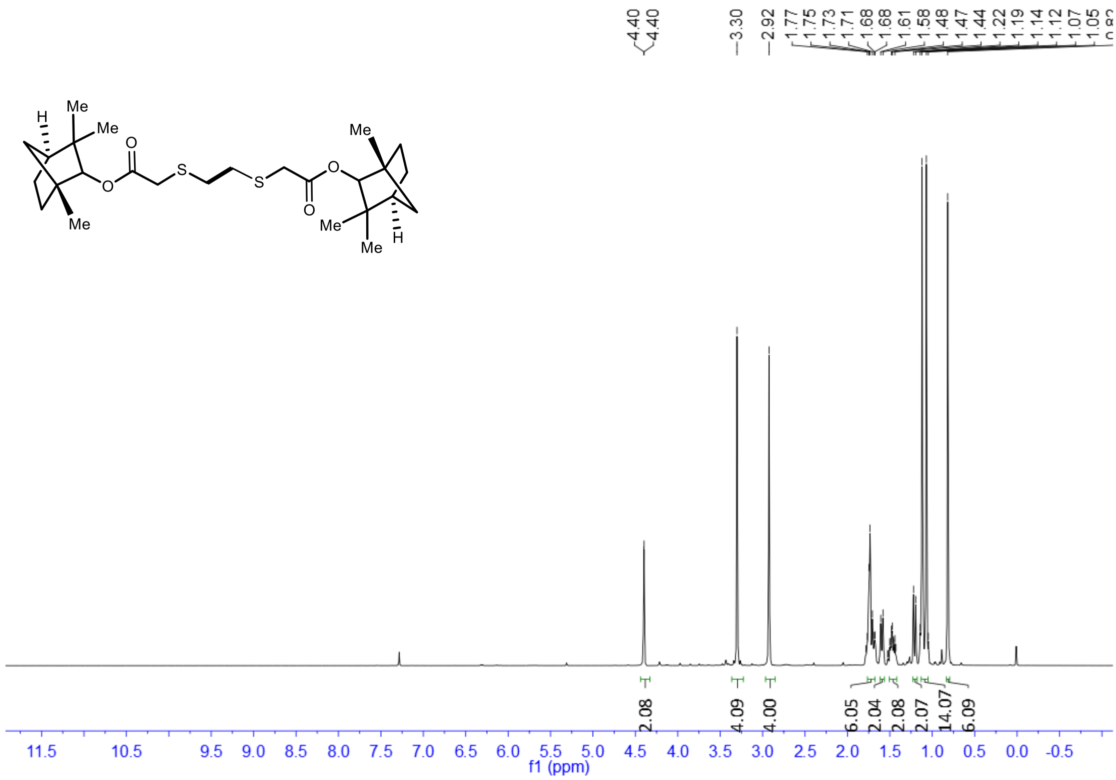

Supplementary Figure 342 | ^{13}C NMR (101 MHz, 298K, $CDCl_3$) of **(84)**

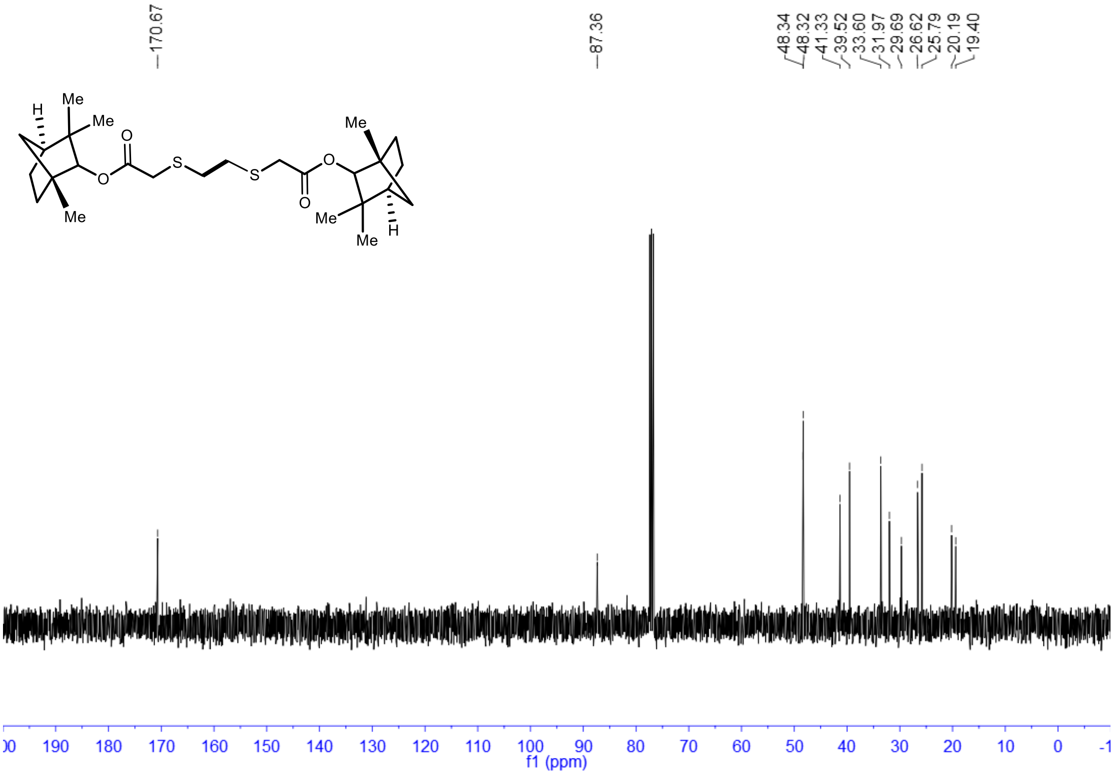

Supplementary Figure 343 | ^{1}H NMR (400 MHz, 298K, $CDCl_3$) of **(85)**

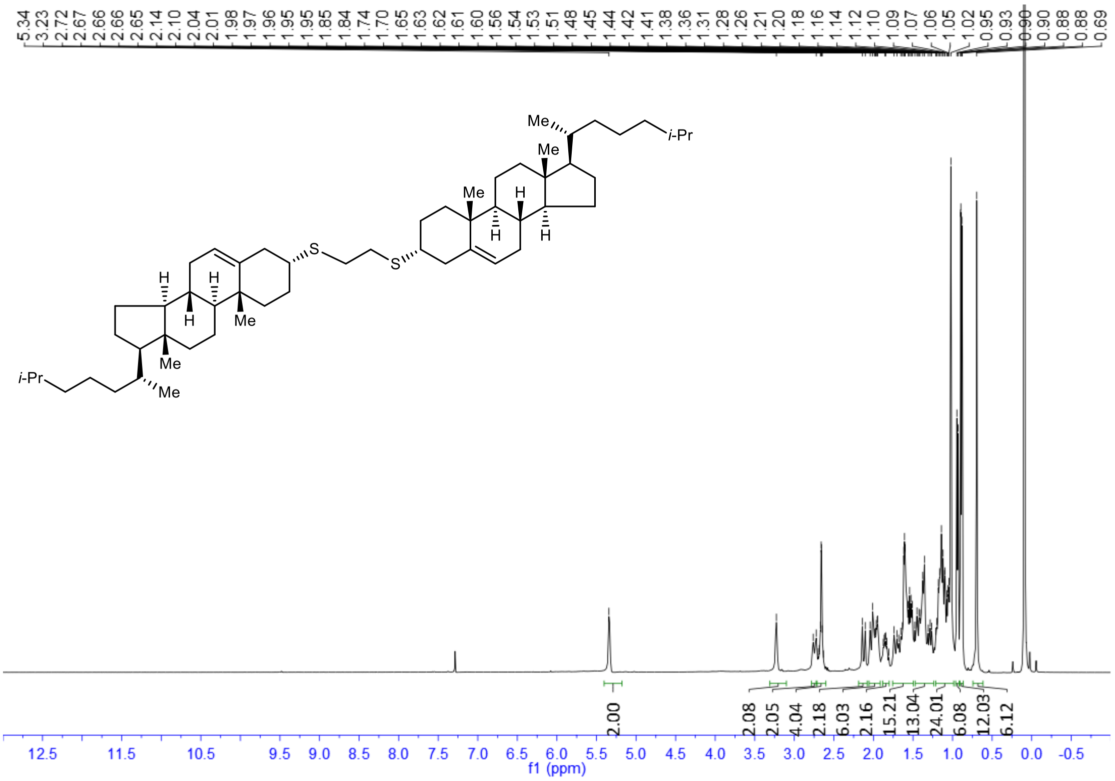

Supplementary Figure 344 | ^{13}C NMR (101 MHz, 298K, $CDCl_3$) of **(85)**

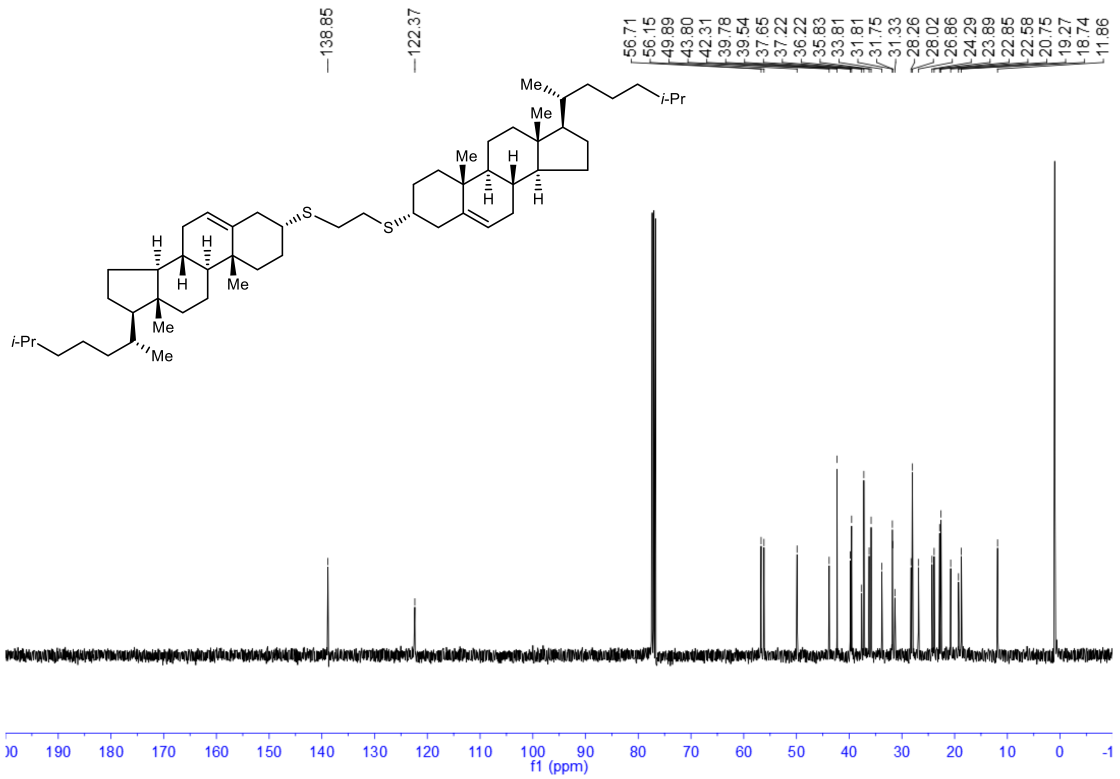

Supplementary Figure 345 | ^{1}H NMR (400 MHz, 298K, $CDCl_3$) of Mesityl(vinyl)sulfane (**86**)

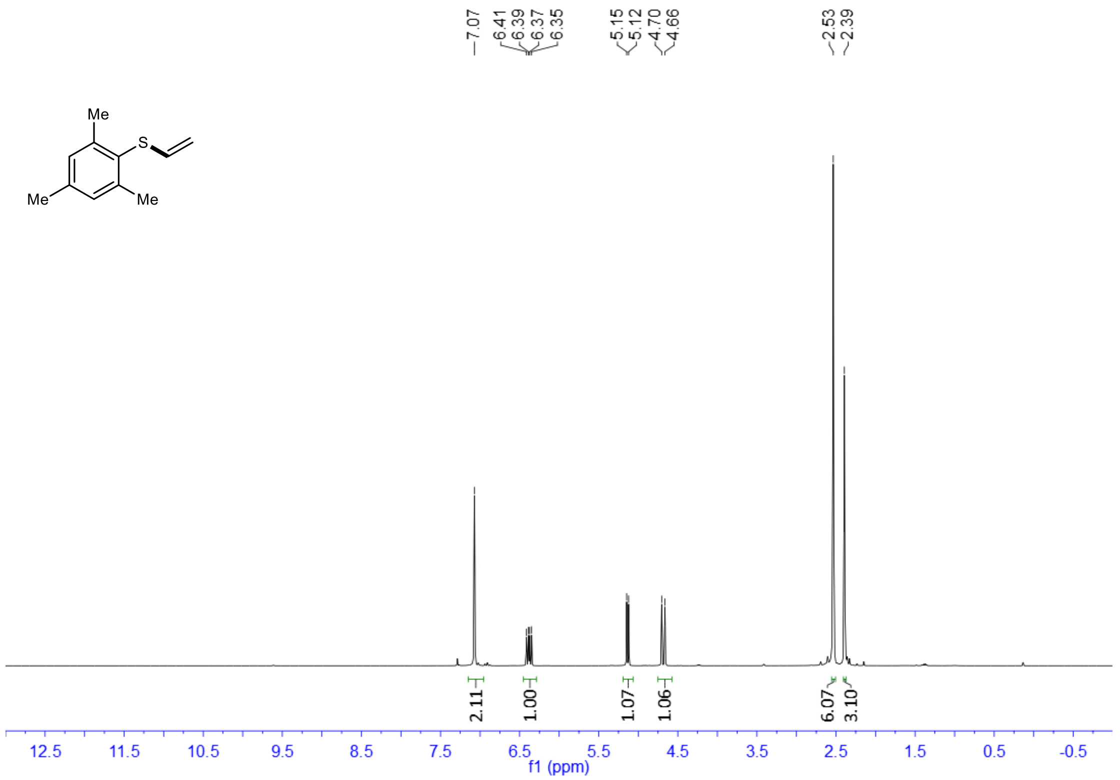

Supplementary Figure 346 | ^{13}C NMR (101 MHz, 298K, $CDCl_3$) of Mesityl(vinyl)sulfane (**86**)

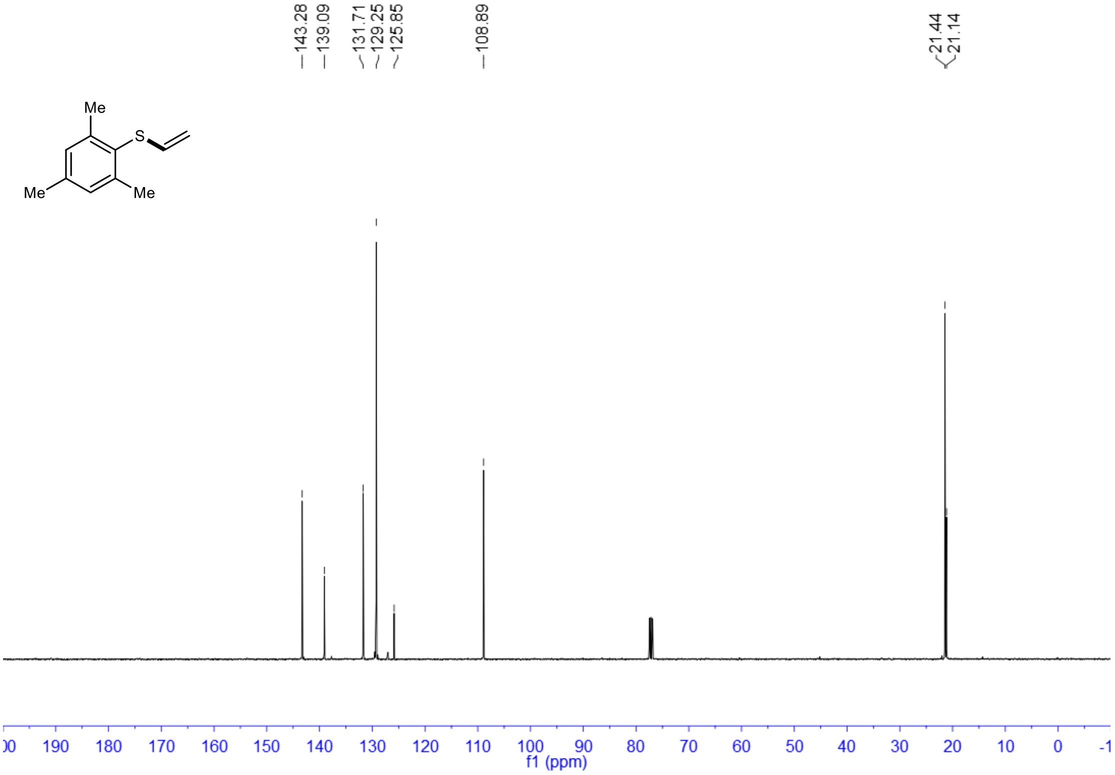

Supplementary Figure 347 | ^{1}H NMR (400 MHz, 298K, $CDCl_3$) of (4-nitrophenyl)(vinyl)Sulfane (**87**)

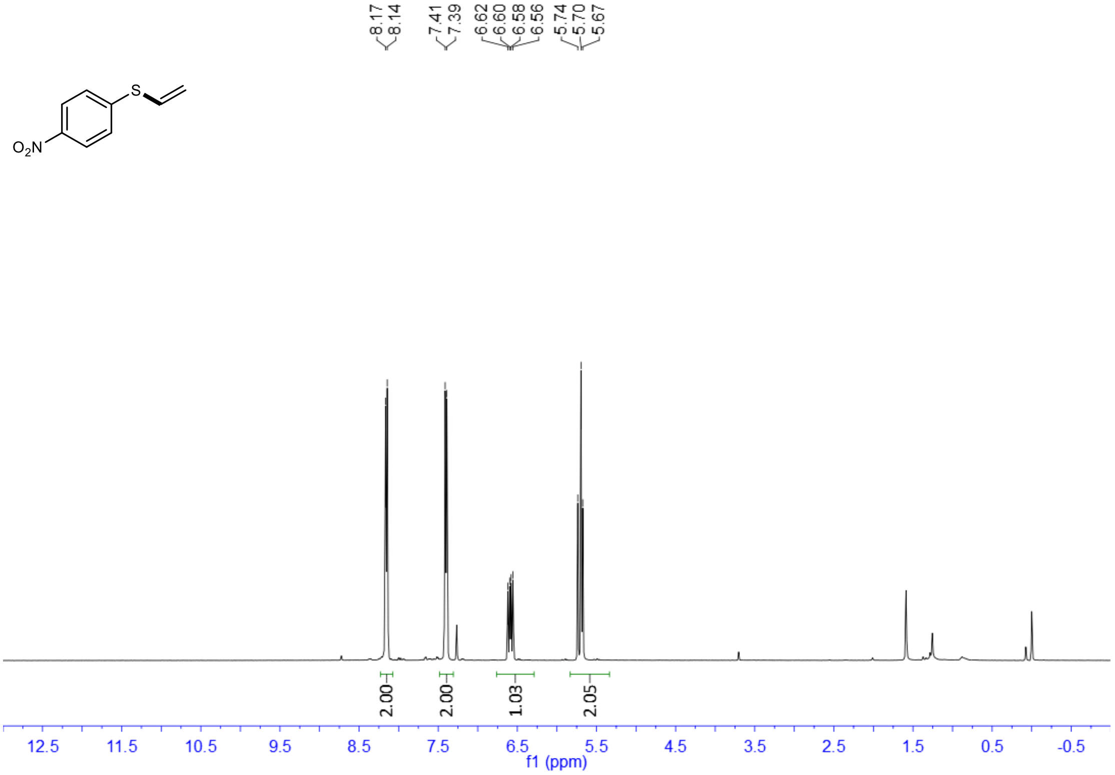

Supplementary Figure 348 | ^{13}C NMR (101 MHz, 298K, $CDCl_3$) of (4-nitrophenyl)(vinyl)Sulfane **(87)**

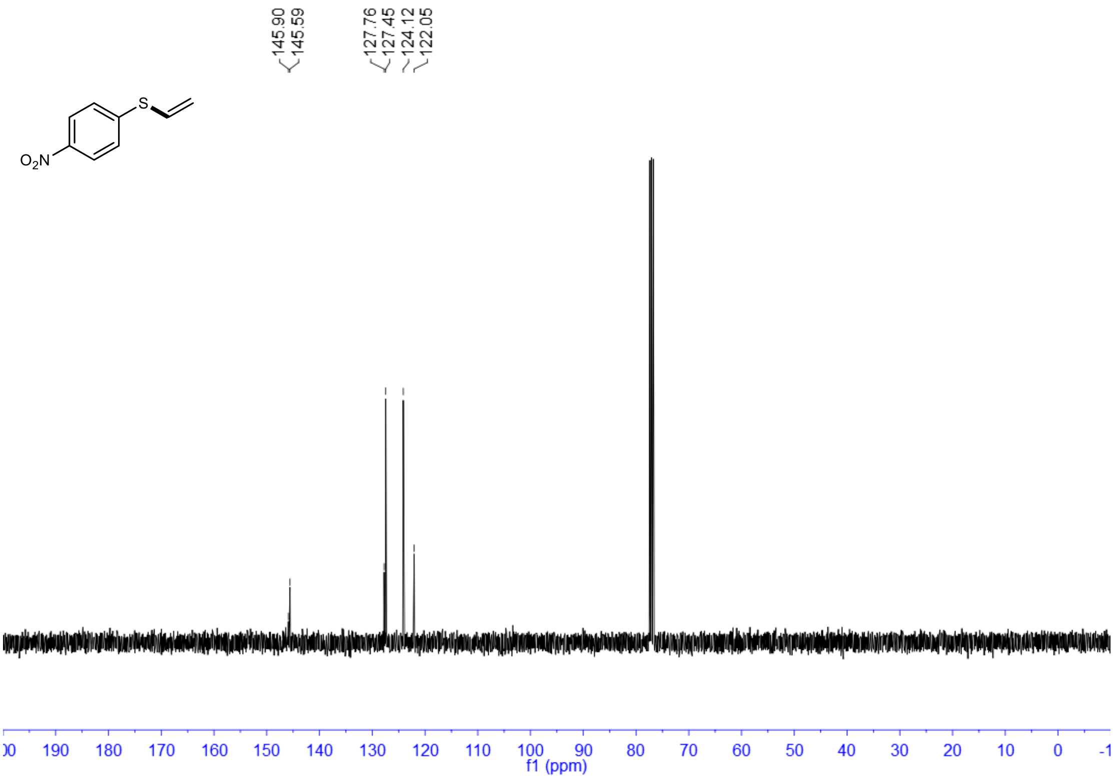

Supplementary Figure 349 | ^{1}H NMR (400 MHz, 298K, $CDCl_3$) of Methyl *N*-(4-chlorobenzyl)-*S*-vinylcysteinate **(88)**

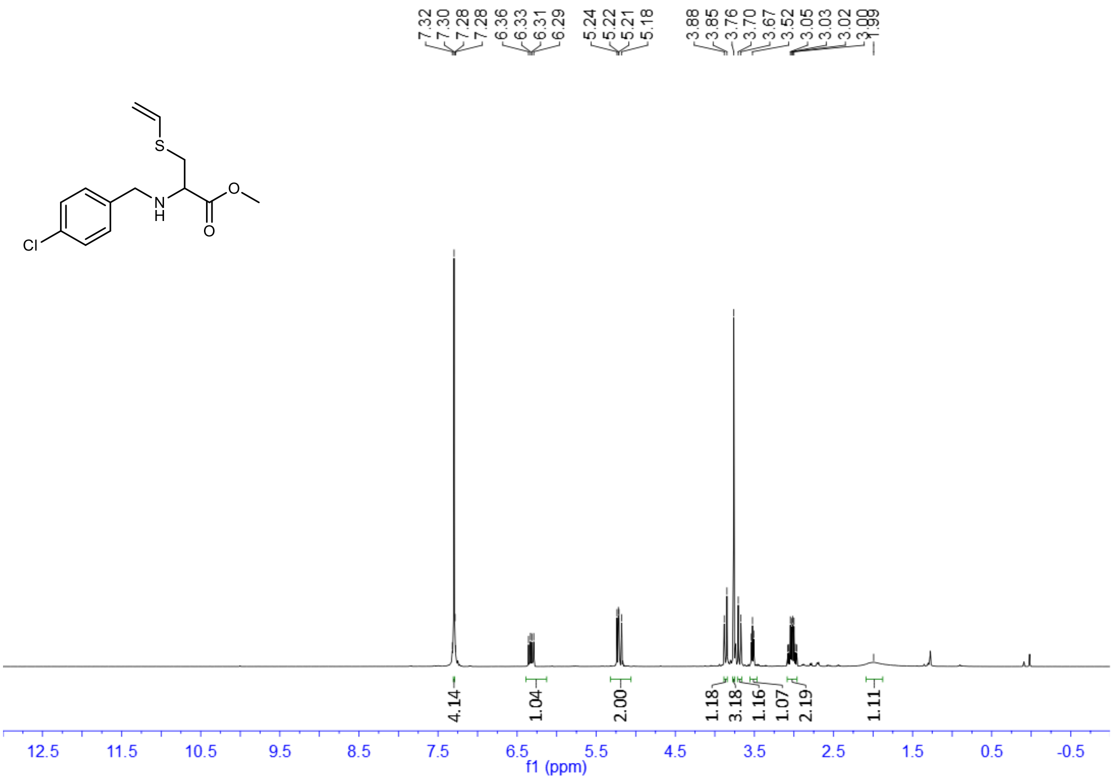

Supplementary Figure 350 | ^{13}C NMR (101 MHz, 298K, $CDCl_3$) of Methyl *N*-(4-chlorobenzyl)-*S*-vinylcysteinate **(88)**

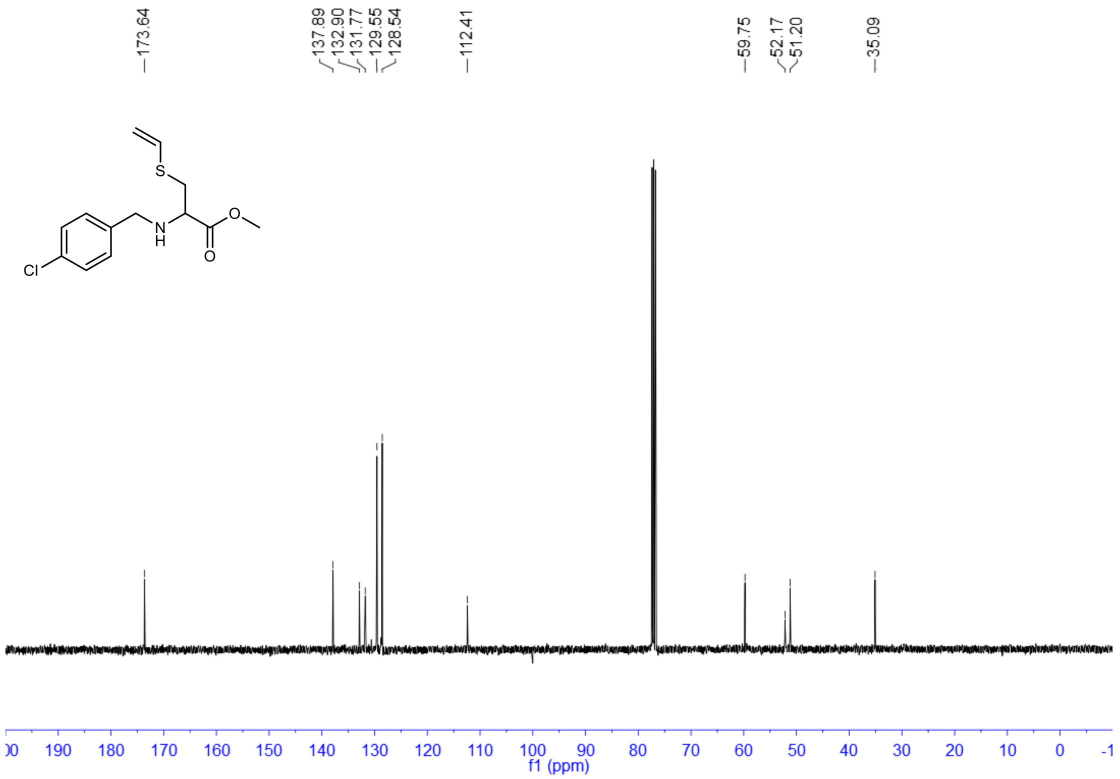

Supplementary Figure 351 | ^{1}H NMR (400 MHz, 298K, $CDCl_3$) of ((1*r*,3*r*)-Adamantan-1-yl)(2-((2,4,6-triisopropylphenyl)thio)ethyl)sulfane **(89)**

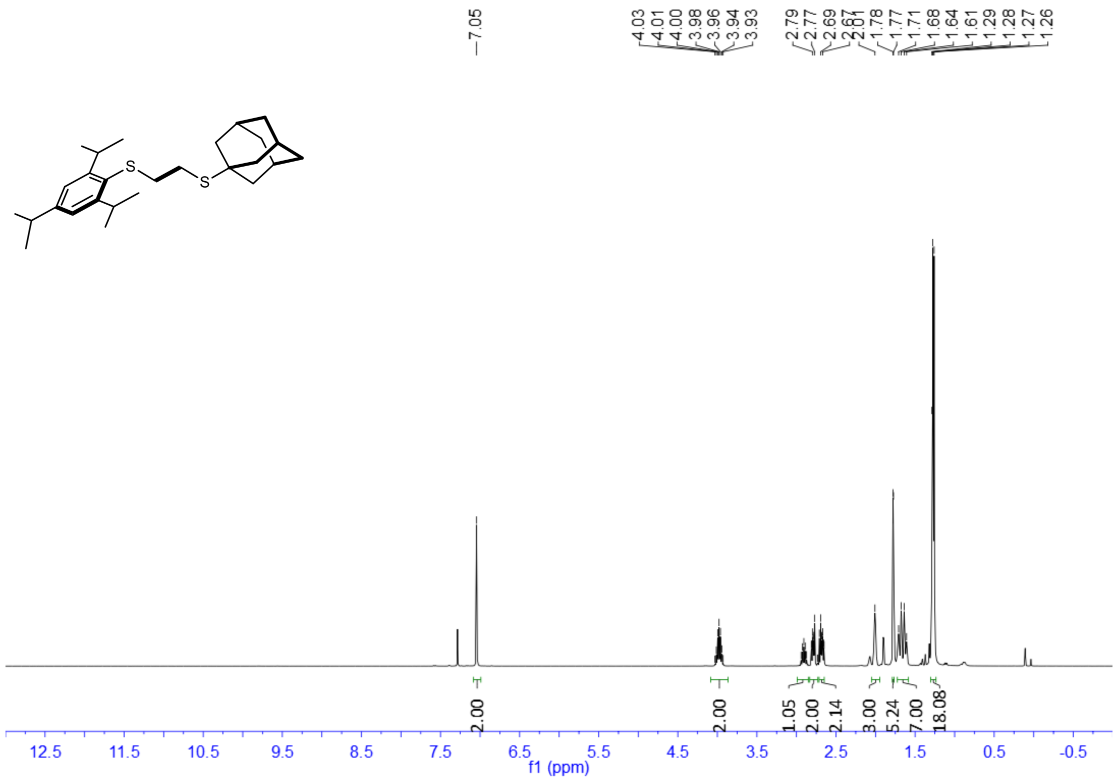

Supplementary Figure 352 | ^{13}C NMR (101 MHz, 298K, $CDCl_3$) of ((1*r*,3*r*)-Adamantan-1-yl)(2-((2,4,6-triisopropylphenyl)thio)ethyl)sulfane **(89)**

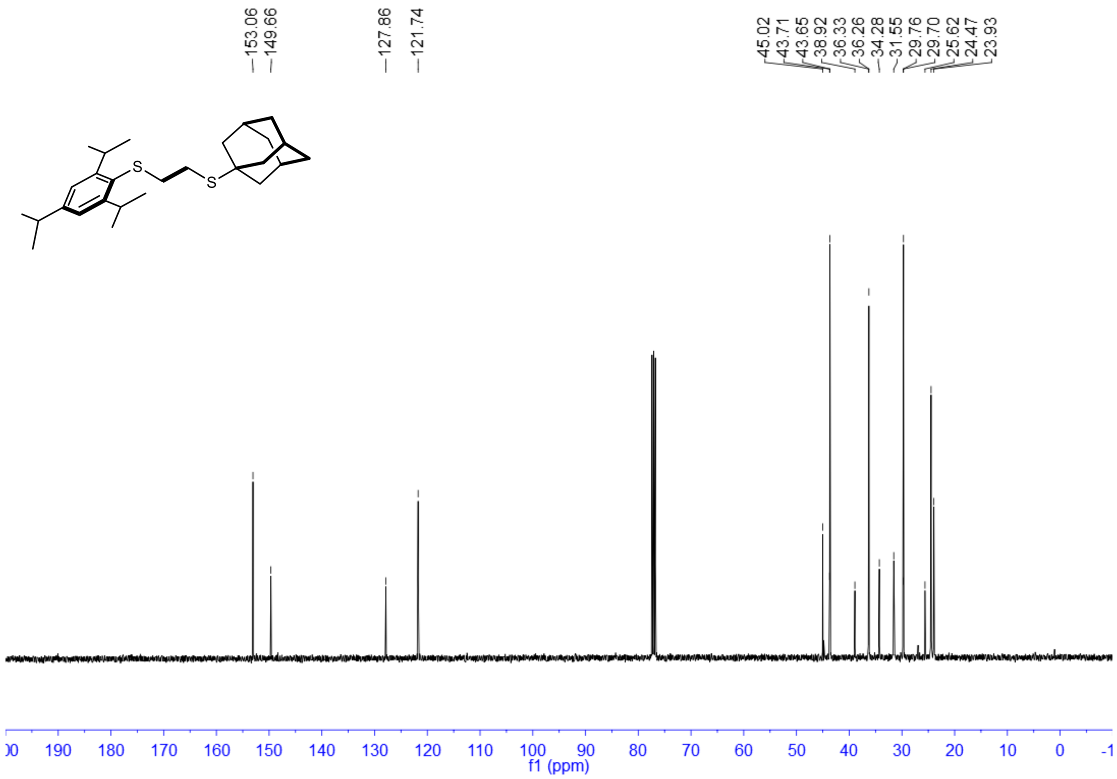

Supplementary Figure 353 | ^{1}H NMR (400 MHz, 298K, $CDCl_3$) of Ethyl 2-((2-((2,4,6-triisopropylphenyl)thio)ethyl)thio)acetate **(90)**

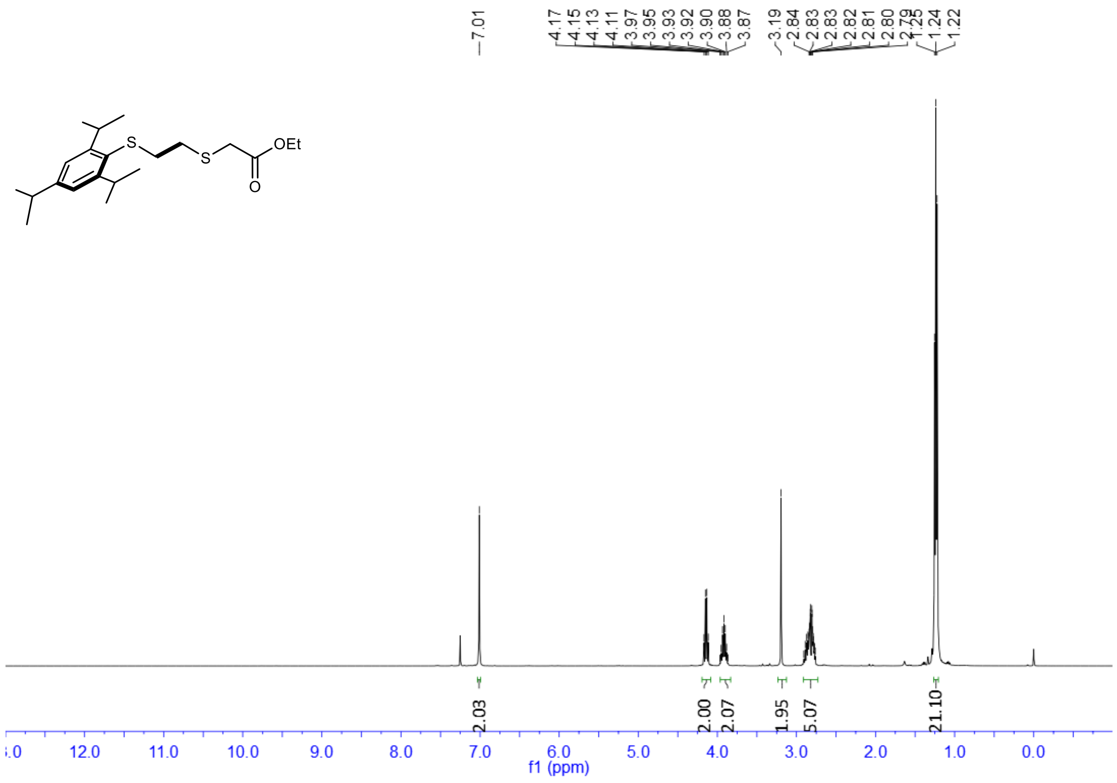

Supplementary Figure 354 | ^{13}C NMR (101 MHz, 298K, $CDCl_3$) of Ethyl 2-((2-((2,4,6-triisopropylphenyl)thio)ethyl)thio)acetate **(90)**

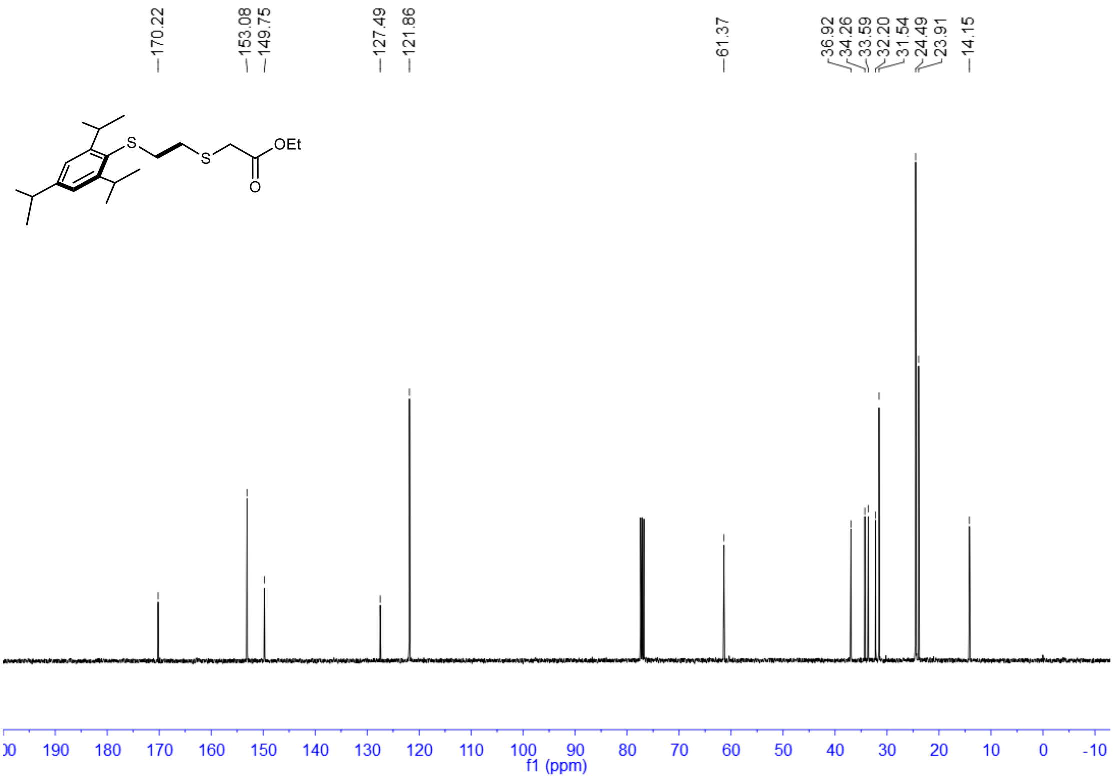

Supplementary Figure 355 | ^{1}H NMR (400 MHz, 298K, $CDCl_3$) of *Tert*-butyl (2-((2-((2,4,6-triisopropylphenyl)thio)ethyl)thio)ethyl)carbamate **(91)**

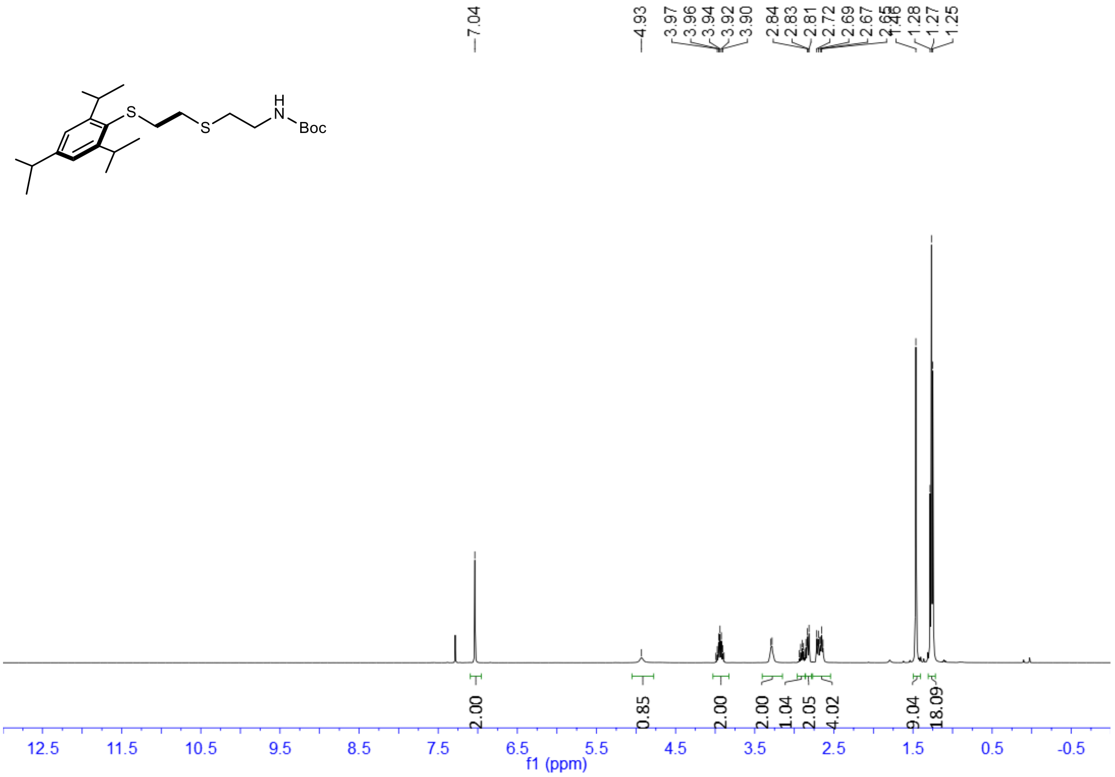

Supplementary Figure 356 | ^{13}C NMR (101 MHz, 298K, $CDCl_3$) of *Tert*-butyl (2-((2-((2,4,6-triisopropylphenyl)thio)ethyl)thio)ethyl)carbamate **(91)**

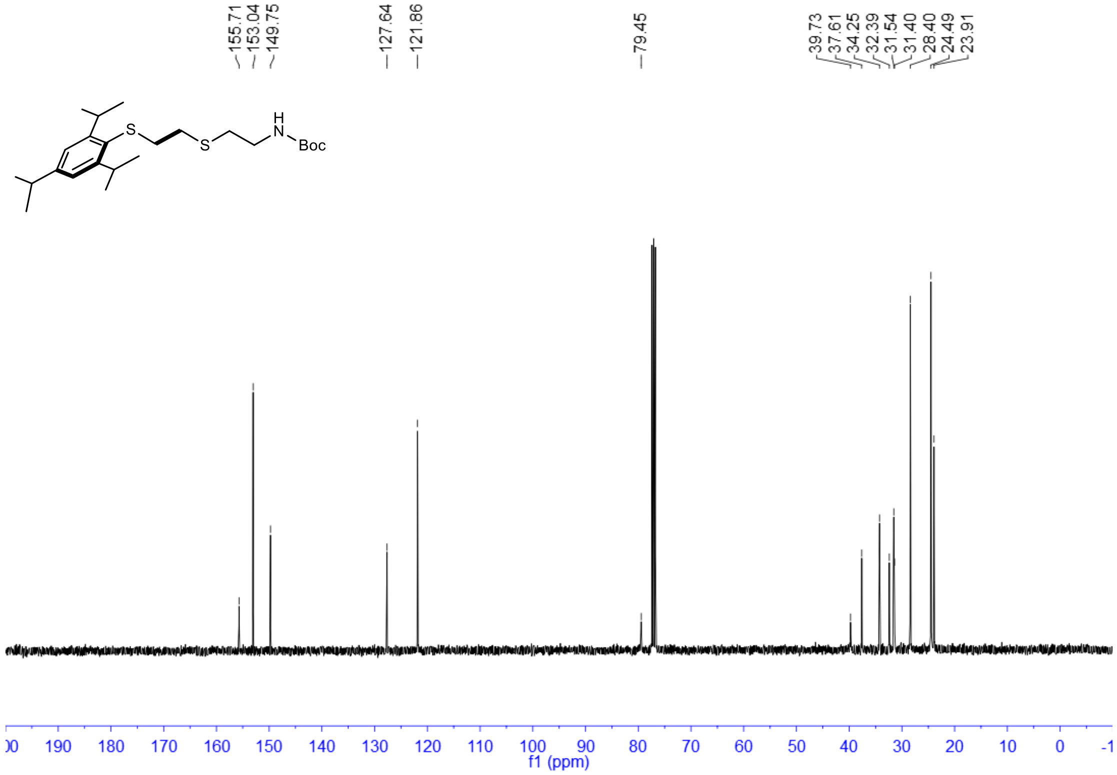

Supplementary Figure 357 | ^{1}H NMR (400 MHz, 298K, $CDCl_3$) of Methyl *N*-(2,2,2-trifluoroacetyl)-*S*-(2-((2,4,6-triisopropylphenyl)thio)ethyl)-L-cysteinate **(92)**

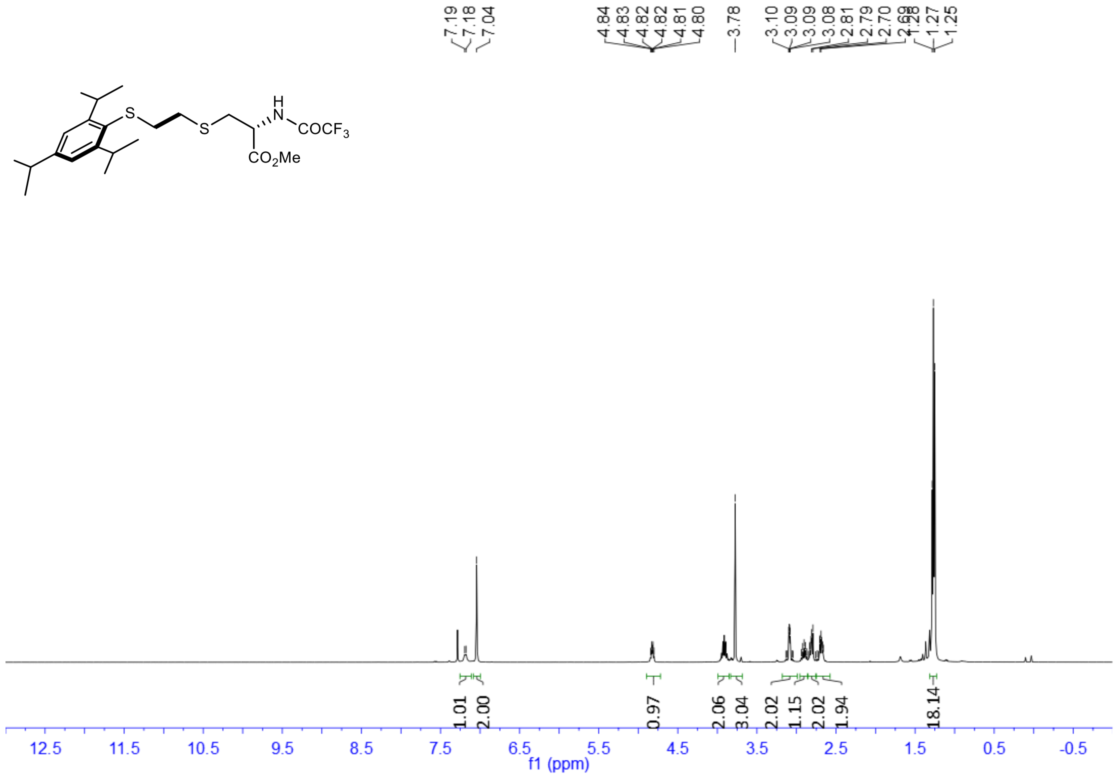

Supplementary Figure 358 | ^{13}C NMR (101 MHz, 298K, $CDCl_3$) of Methyl *N*-(2,2,2-trifluoroacetyl)-*S*-(2-((2,4,6-triisopropylphenyl)thio)ethyl)-L-cysteinate **(92)**

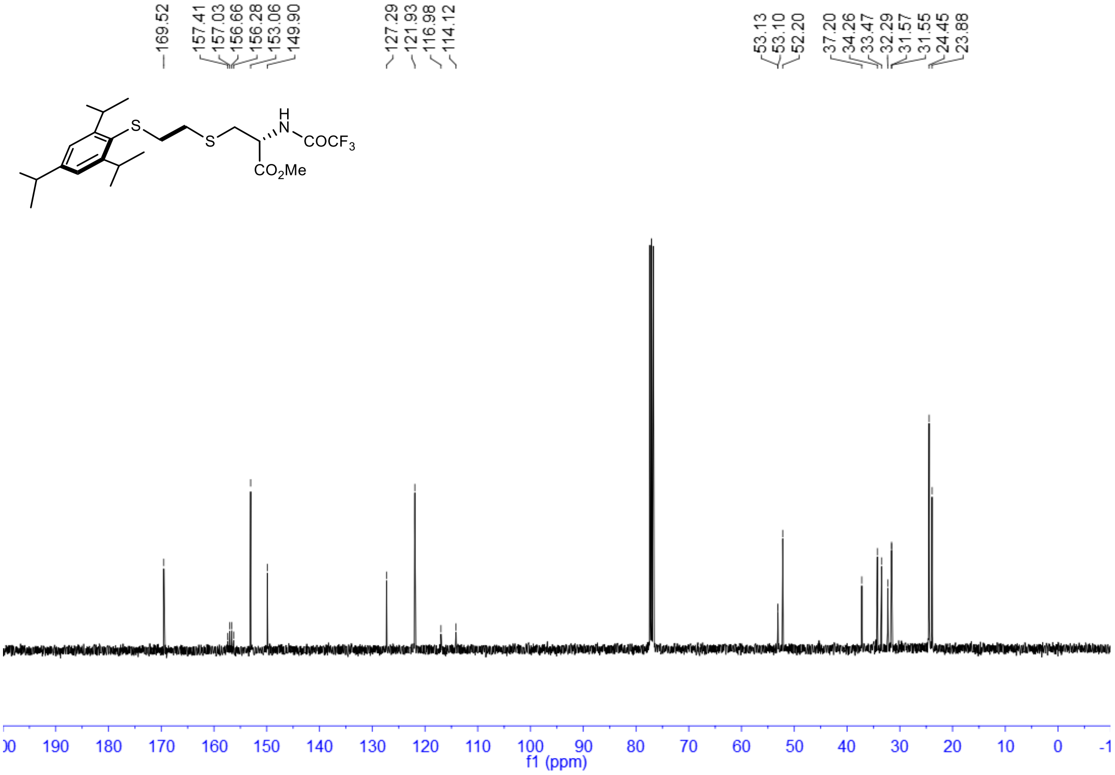

Supplementary Figure 359 | ^{19}F NMR (471 MHz, 298K, $CDCl_3$) of Methyl *N*-(2,2,2-trifluoroacetyl)-*S*-(2-((2,4,6-triisopropylphenyl)thio)ethyl)-L-cysteinate **(92)**

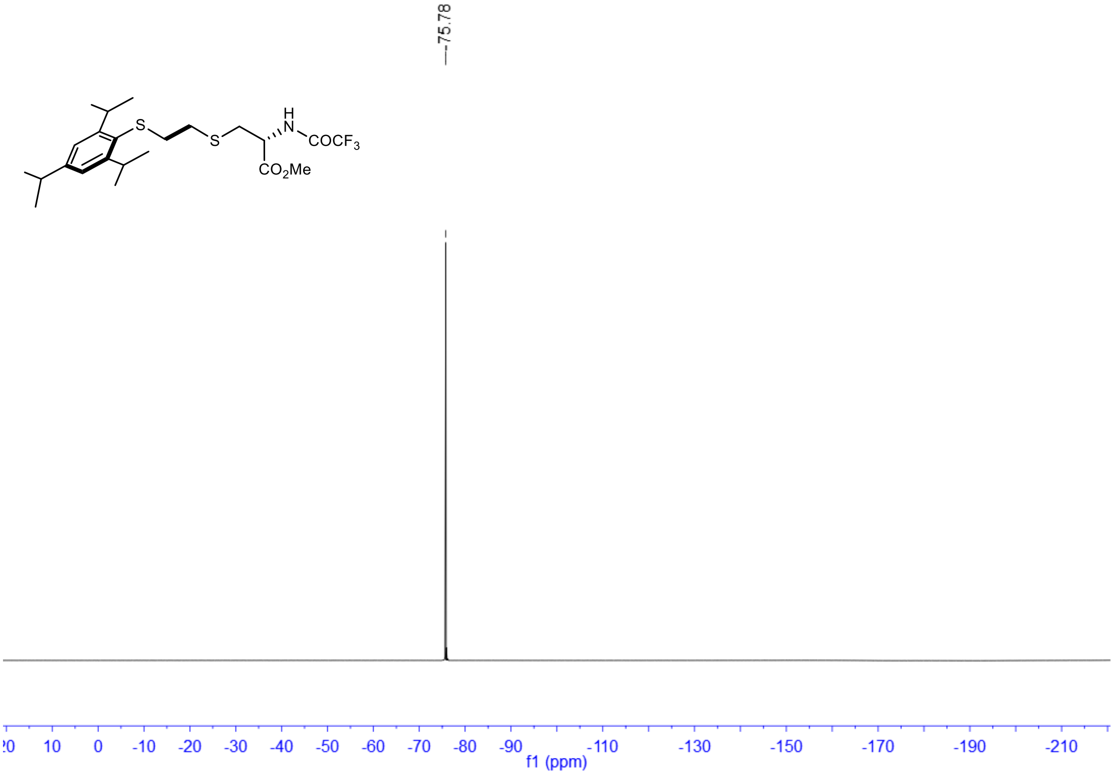

Supplementary Figure 360 | ^{1}H NMR (400 MHz, 298K, CDCl$_3$) of Diphenyl(2-((2,4,6-triisopropylphenyl)thio)ethyl)phosphine oxide **(93)**

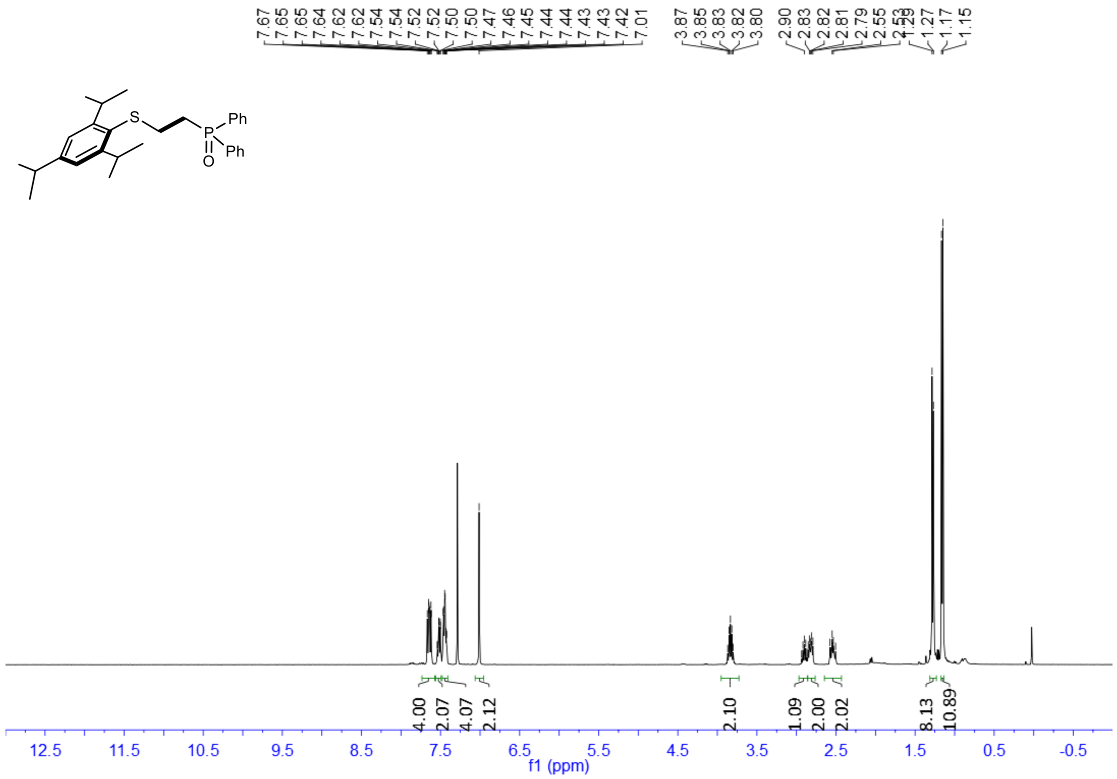

Supplementary Figure 361 | ^{13}C NMR (101 MHz, 298K, CDCl$_3$) of Diphenyl(2-((2,4,6-triisopropylphenyl)thio)ethyl)phosphine oxide **(93)**

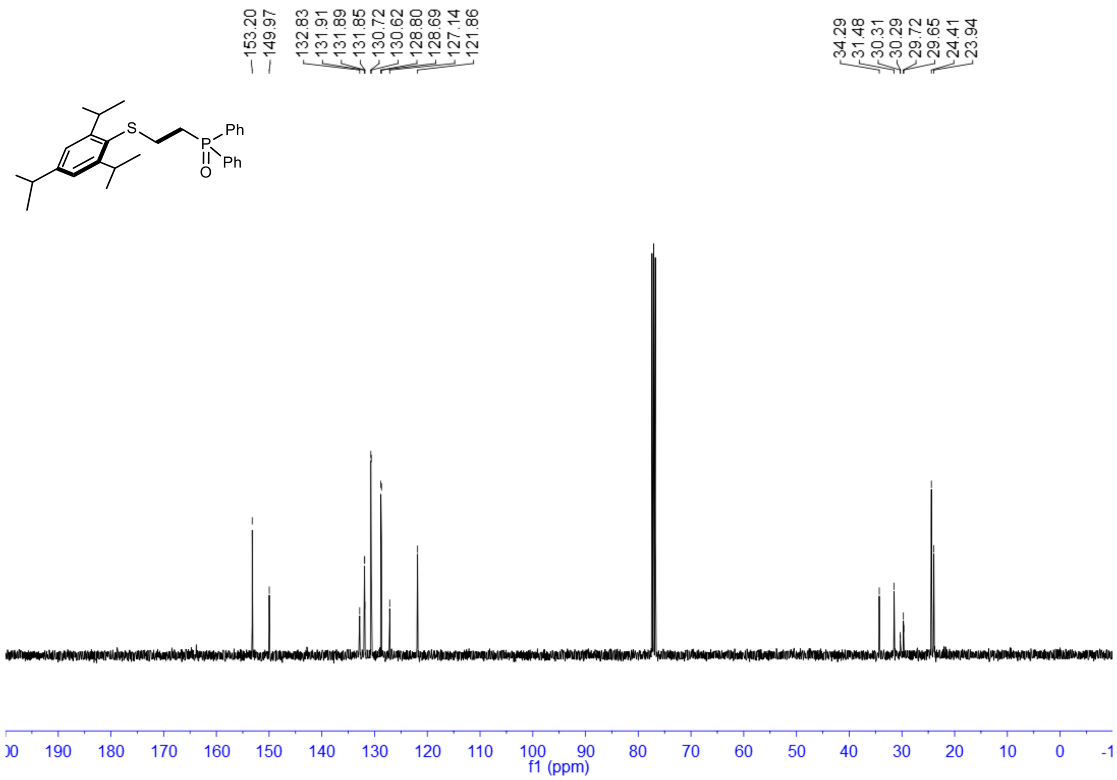

Supplementary Figure 362 | ^{31}P NMR (202 MHz, 298K, $CDCl_3$) of Diphenyl(2-((2,4,6-triisopropylphenyl)thio)ethyl)phosphine oxide **(93)**

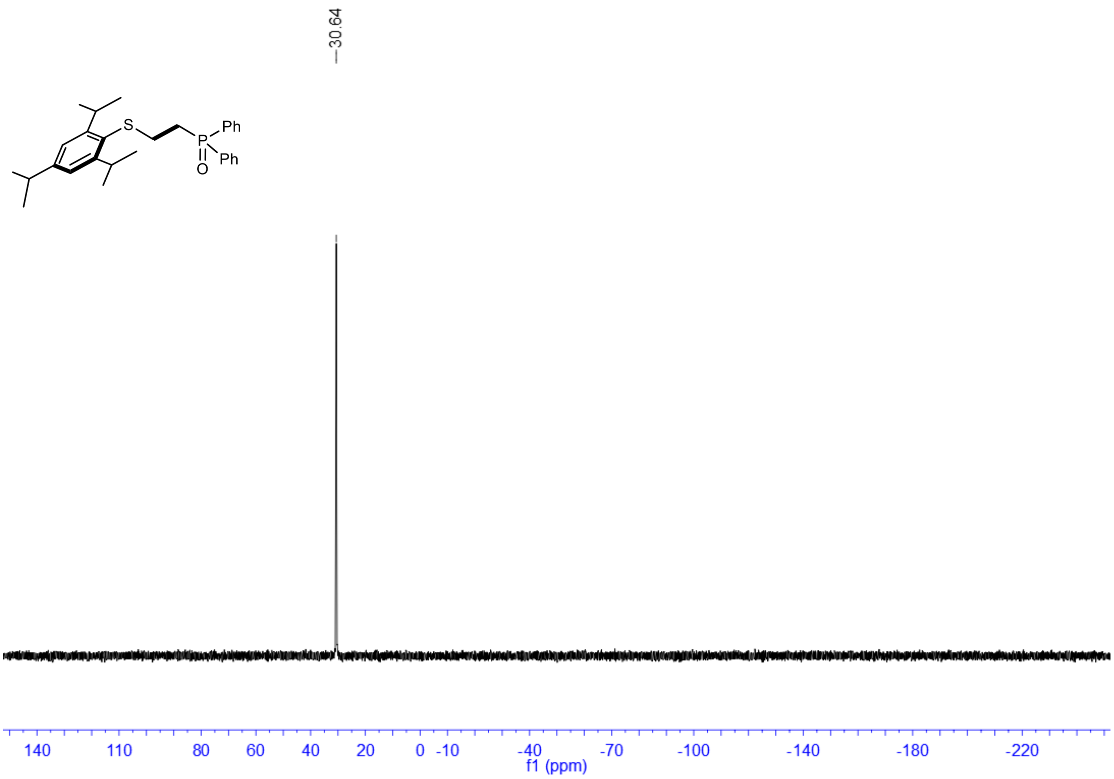

Supplementary Figure 363 | ^{1}H NMR (500 MHz, 298K, $CDCl_3$) of 1,4-Dioxa-7,10-dithiacyclododecane 7,10-dioxide **(94)**

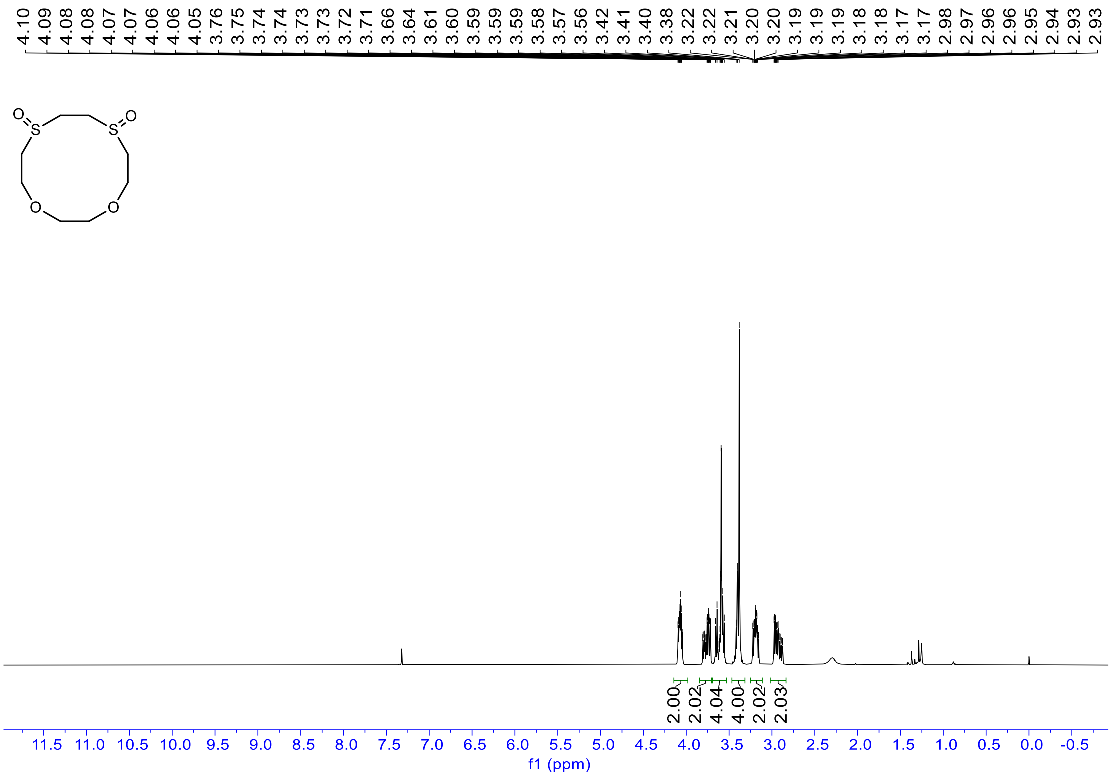

Supplementary Figure 364 | ^{13}C NMR (126 MHz, 298K, $CDCl_3$) of 1,4-Dioxa-7,10-dithiacyclododecane 7,10-dioxide **(94)**

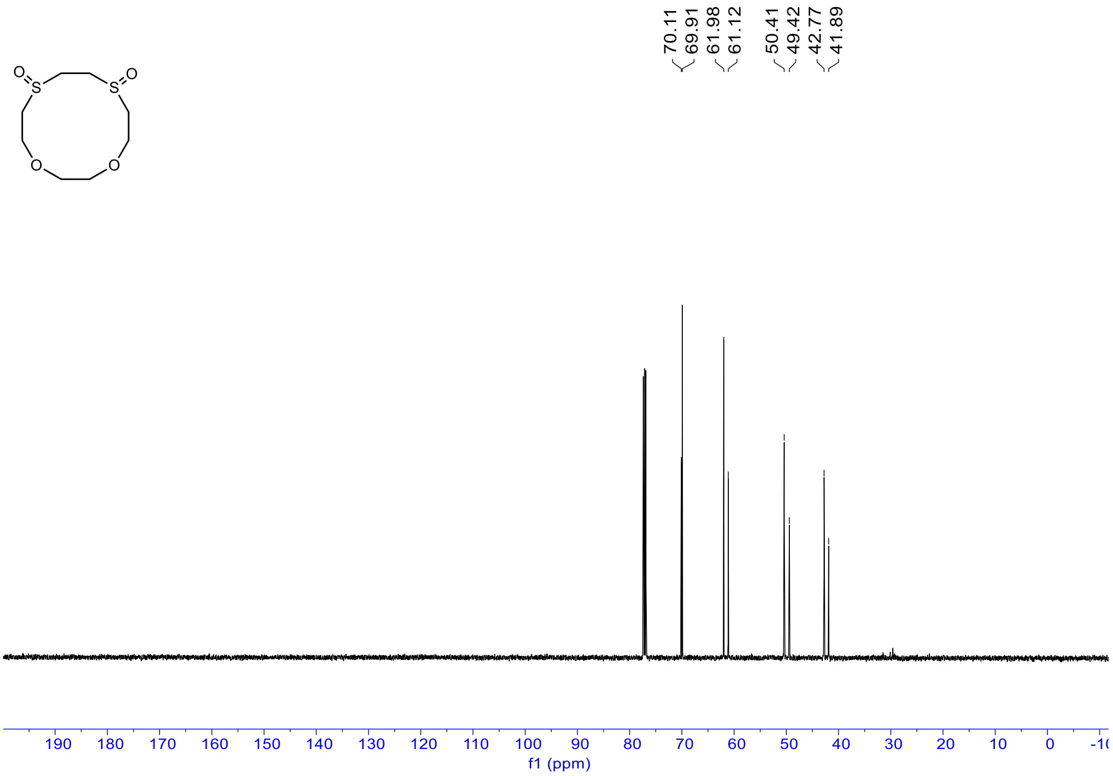

Supplementary Figure 365 | ^{1}H NMR (500 MHz, 298K, $CDCl_3$) of 1,4-Dioxa-7,10-dithiacyclododecane 7,7,10,10-tetraoxide **(95)**

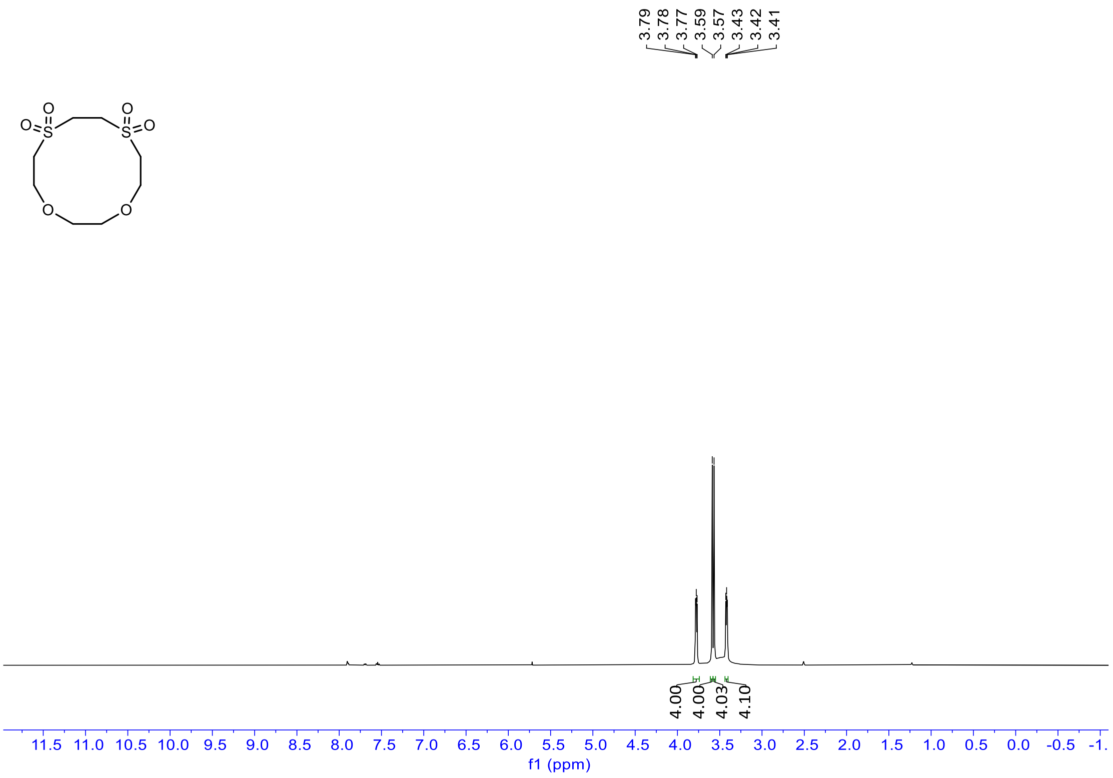

Supplementary Figure 366 | ^{13}C NMR (126 MHz, 298K, $CDCl_3$) of 1,4-Dioxa-7,10-dithiacyclododecane 7,7,10,10-tetraoxide **(95)**

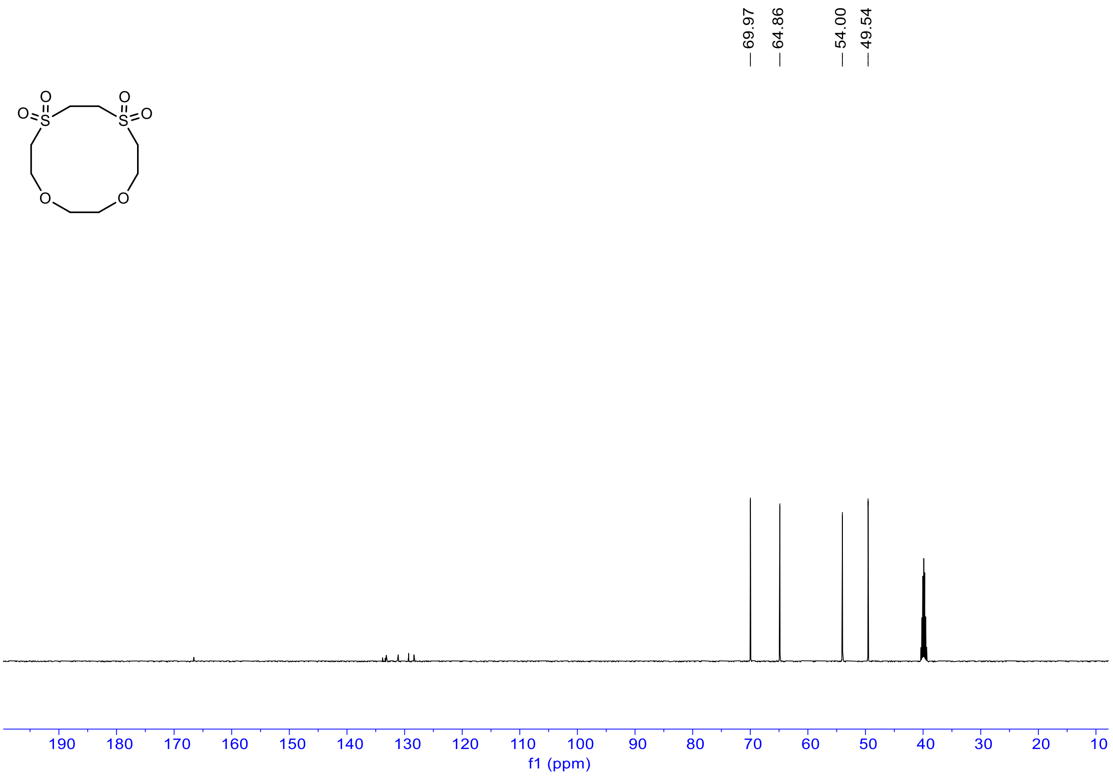

Supplementary Figure 367 | ^{1}H NMR (400 MHz, 298K, $CDCl_3$) of Diethyl (2-(*p*-tolylthio)ethyl)phosphonate (**87**)

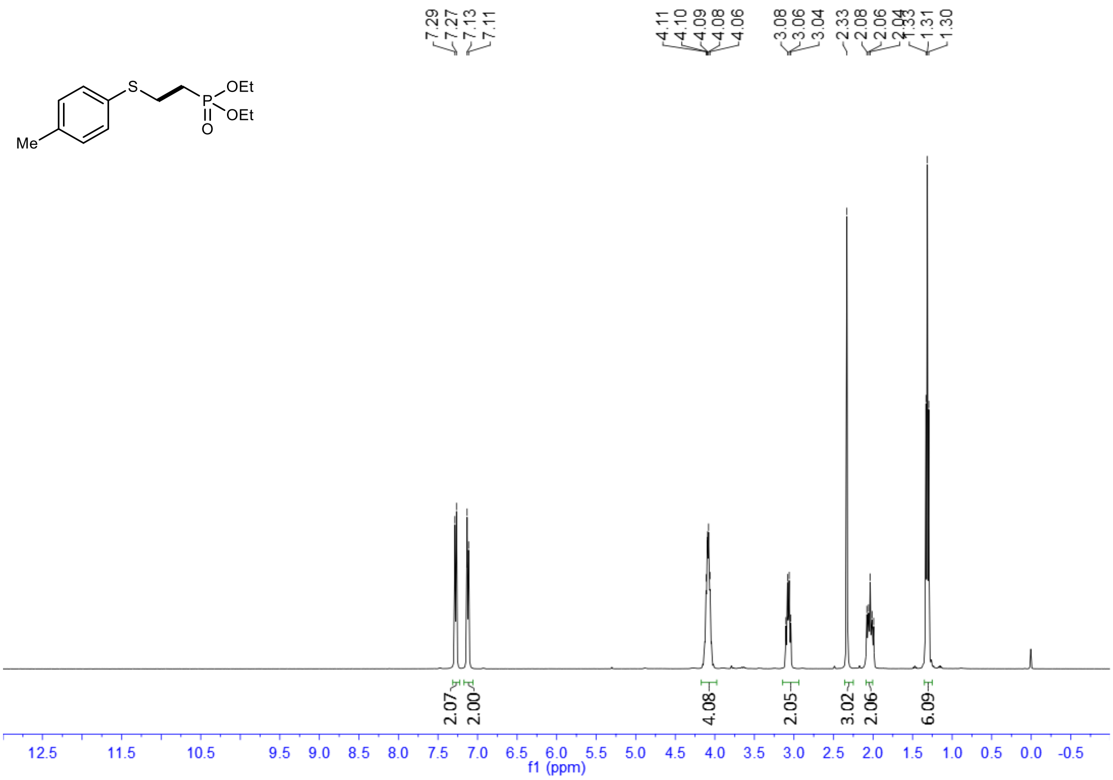

Supplementary Figure 368 | ^{1}H NMR (101 MHz, 298K, $CDCl_3$) of Diethyl (2-(*p*-tolylthio)ethyl)phosphonate (**87**)

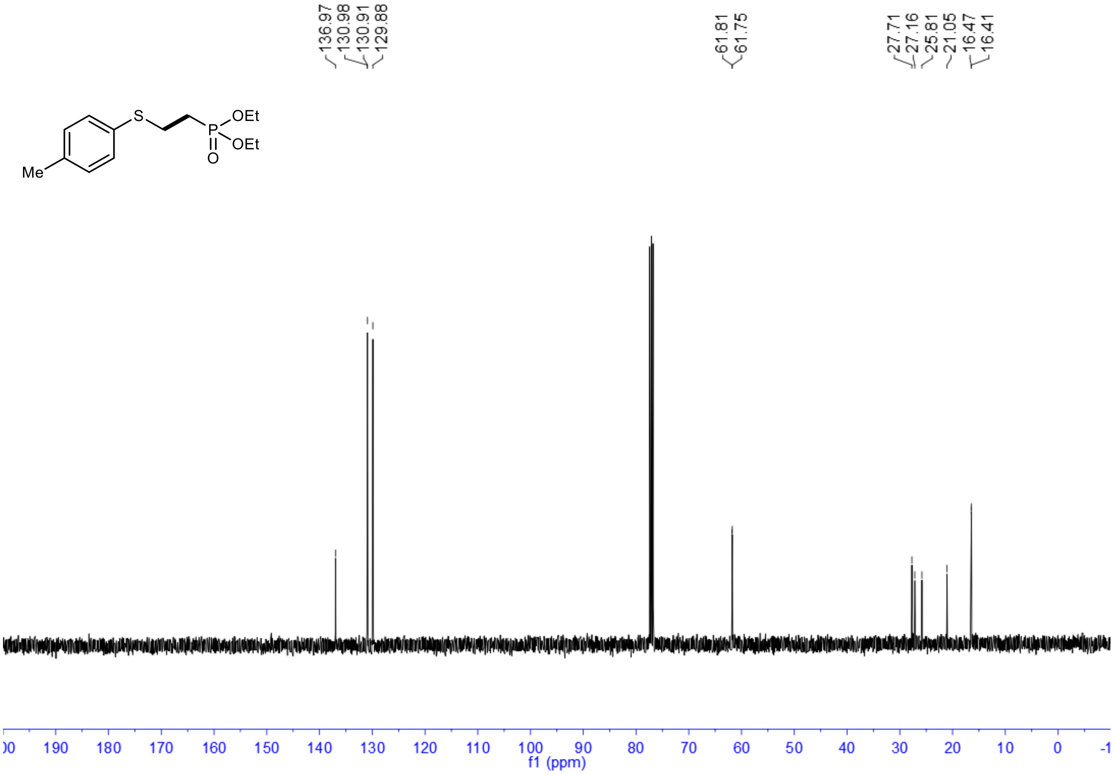

Supplementary Figure 369 | ^{31}P NMR (162 MHz, 298K, $CDCl_3$) of Diethyl (2-(*p*-tolylthio)ethyl)phosphonate (**87**)

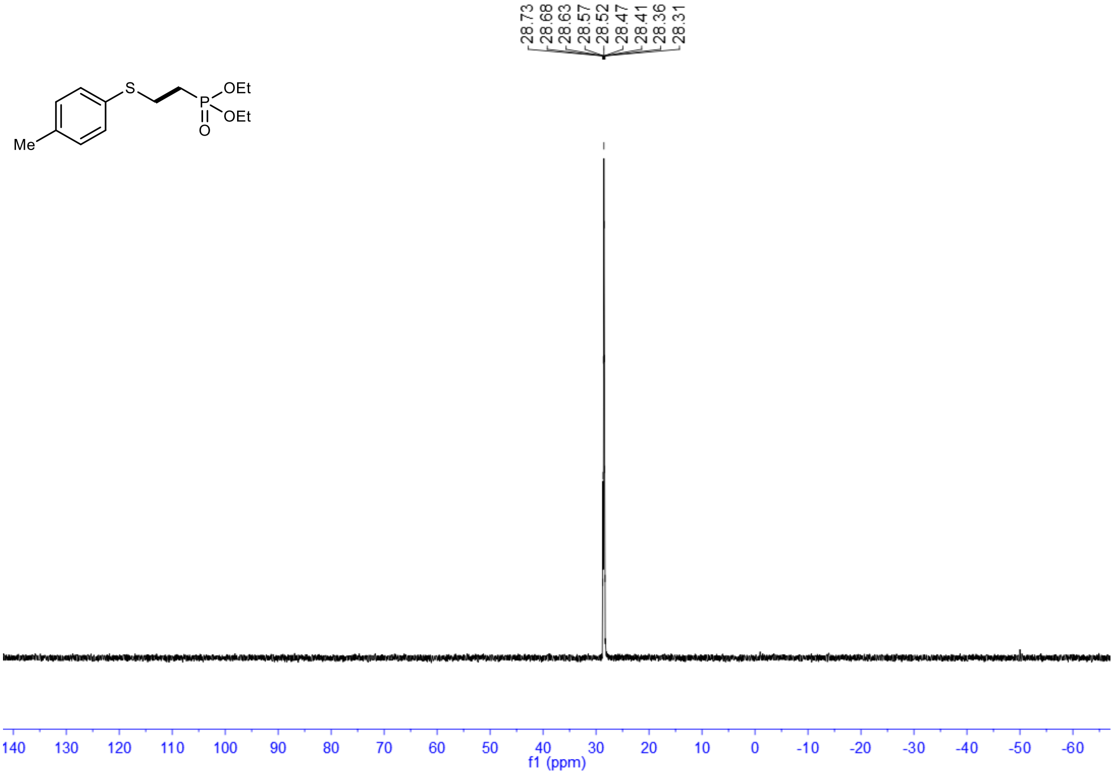

Supplementary Figure 370 | ^{1}H NMR (500 MHz, 298K, $CDCl_3$) of (*S*)-Diethyl (2-(*p*-tolylsulfinyl)ethyl)phosphonate **(89)**

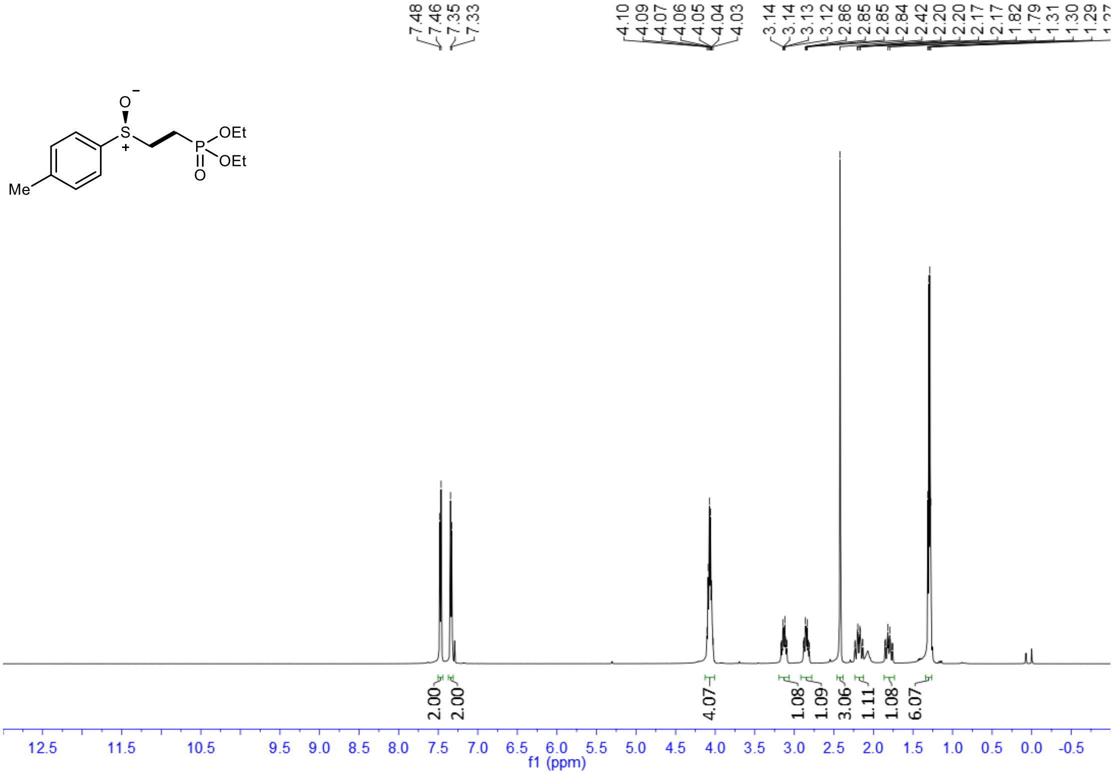

Supplementary Figure 371 | ^{13}C NMR (126 MHz, 298K, $CDCl_3$) of (*S*)-Diethyl (2-(*p*-tolylsulfinyl)ethyl)phosphonate **(89)**

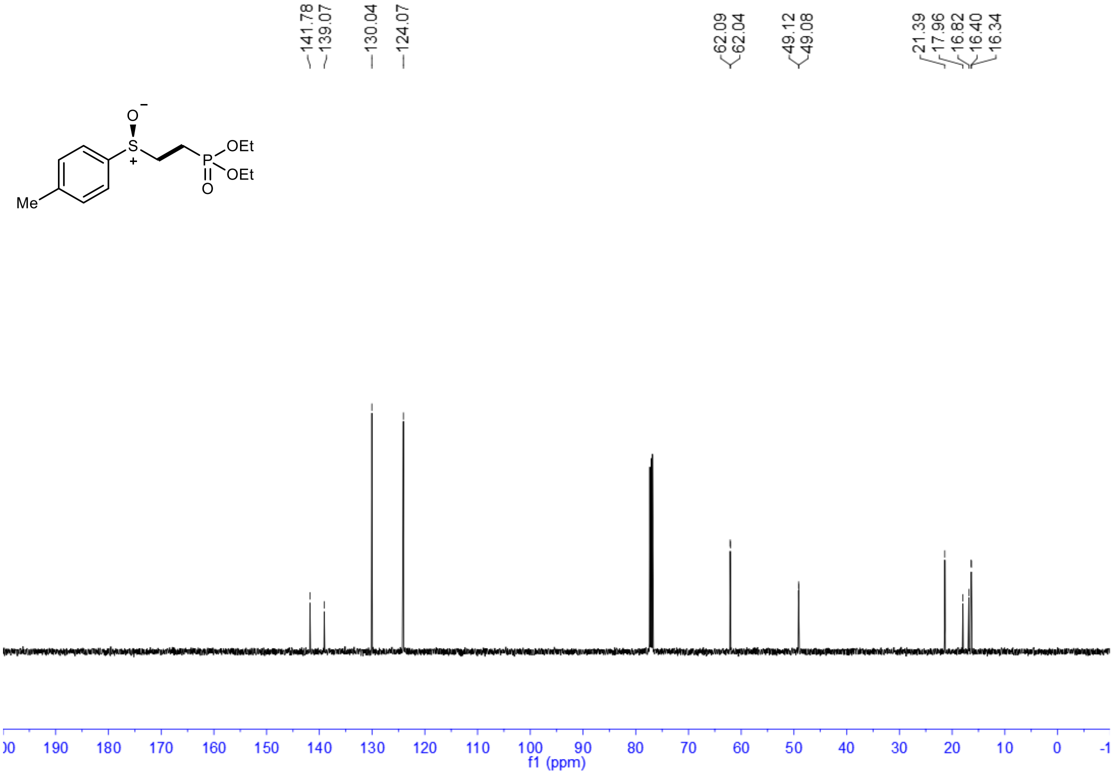

Supplementary Figure 372 | ^{31}P NMR (202 MHz, 298K, $CDCl_3$) of (*S*)-Diethyl (2-(*p*-tolylsulfinyl)ethyl)phosphonate **(89)**

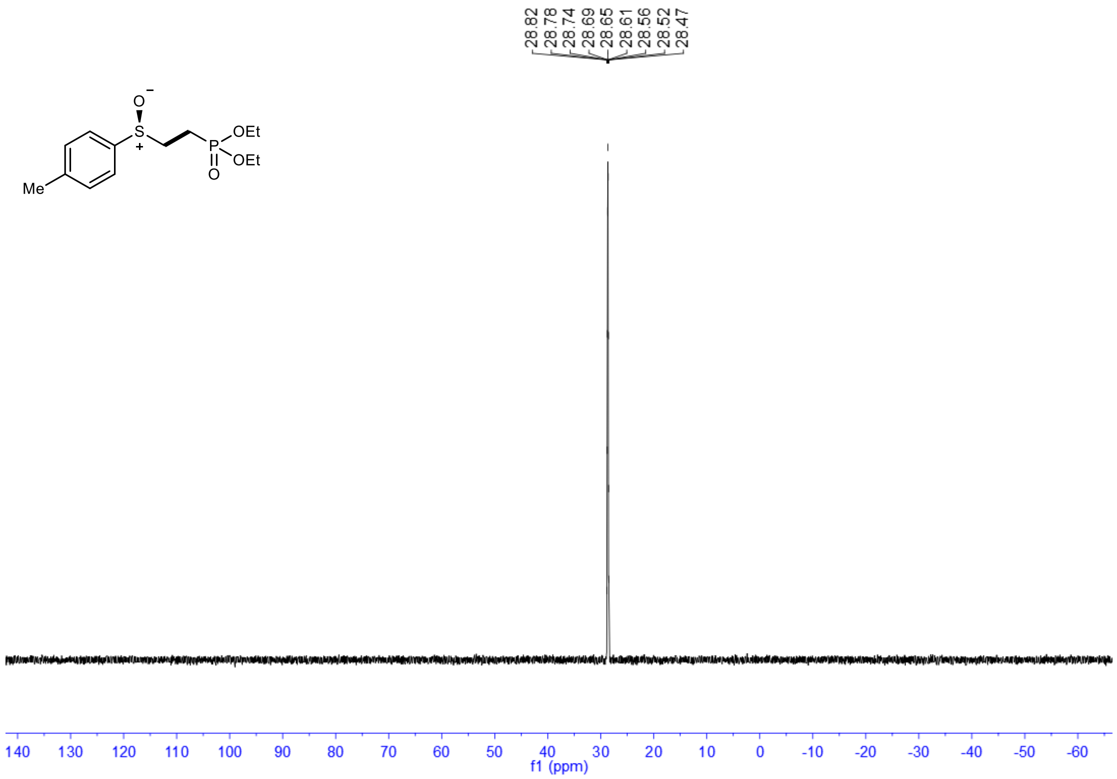

Supplementary Figure 373 | ^{1}H NMR (400 MHz, 298K, $CDCl_3$) of Diethyl (2-((4-(*tert*-butyl)phenyl)thio)ethyl)phosphonate **(88)**

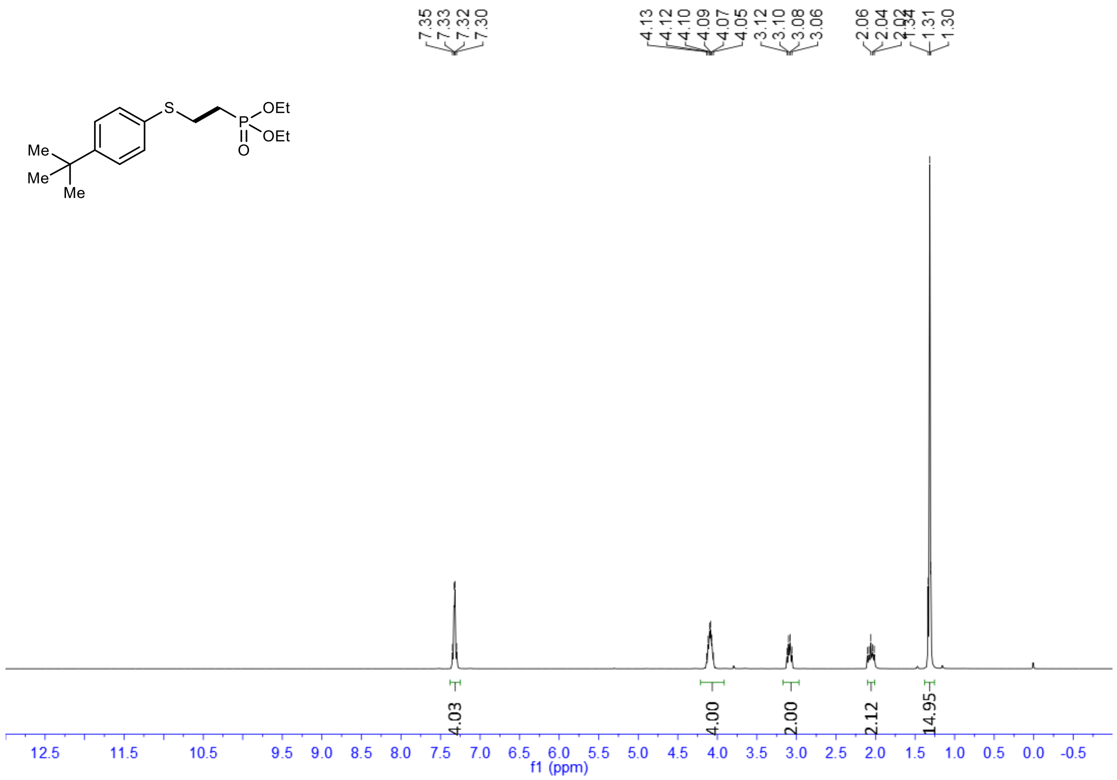

Supplementary Figure 374 | ^{13}C NMR (101 MHz, 298K, $CDCl_3$) of Diethyl (2-((4-(*tert*-butyl)phenyl)thio)ethyl)phosphonate **(88)**

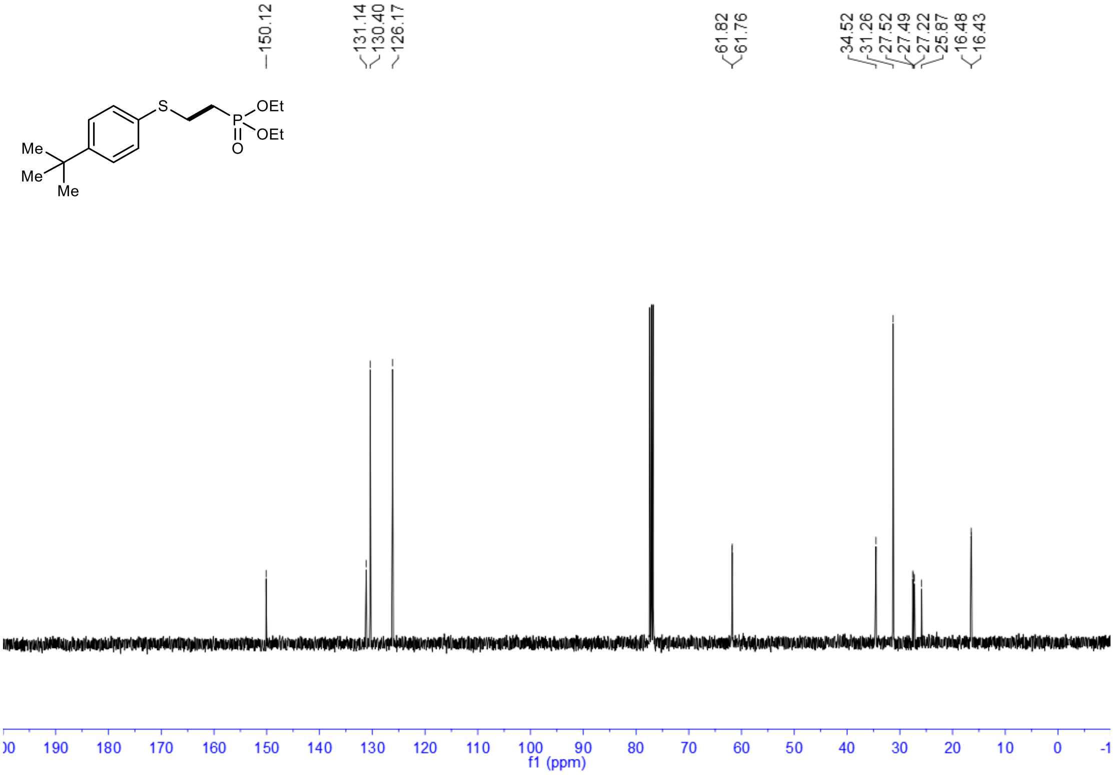

Supplementary Figure 375 | ^{31}P NMR (126 MHz, C298K, DCl_3) of Diethyl (2-((4-(*tert*-butyl)phenyl)thio)ethyl)phosphonate **(88)**

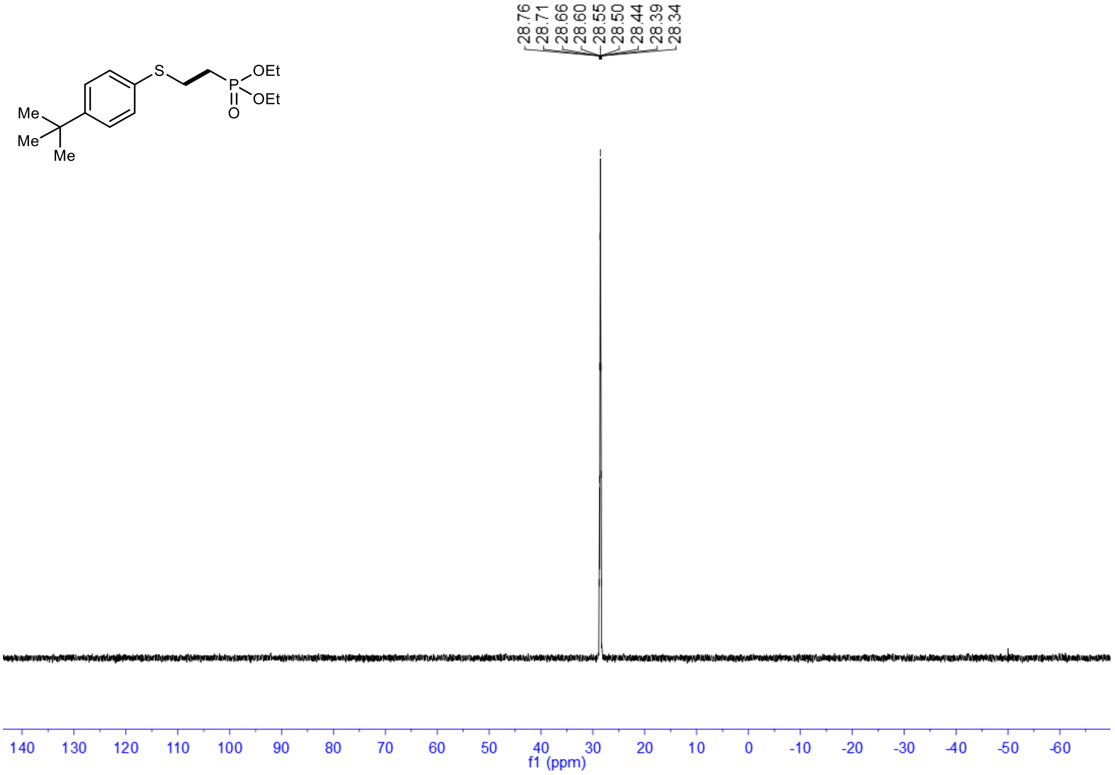

Supplementary Figure 376 | ^{1}H NMR (400 MHz, 298K, $CDCl_3$) of (*S*)-Diethyl (2-((4-(*tert*-butyl)phenyl)sulfinyl)ethyl)phosphonate (**90**)

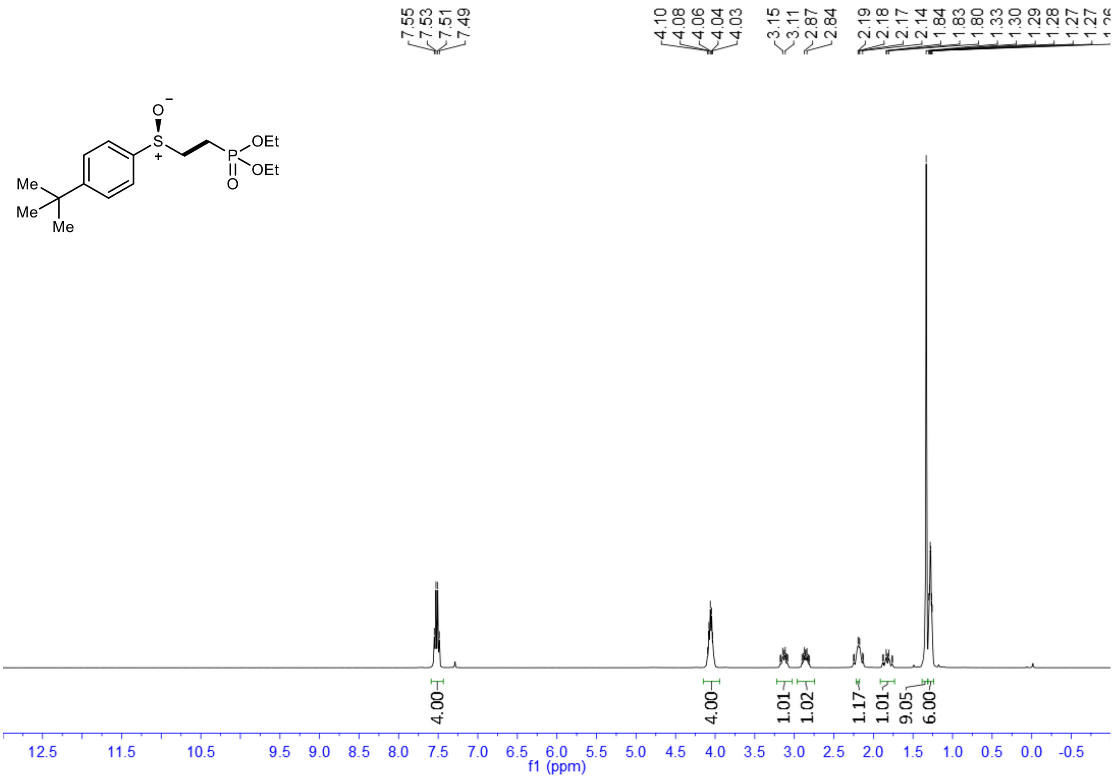

Supplementary Figure 377 | ^{13}C NMR (101 MHz, 298K, $CDCl_3$) of (*S*)-Diethyl (2-((4-(*tert*-butyl)phenyl)sulfinyl)ethyl)phosphonate (**90**)

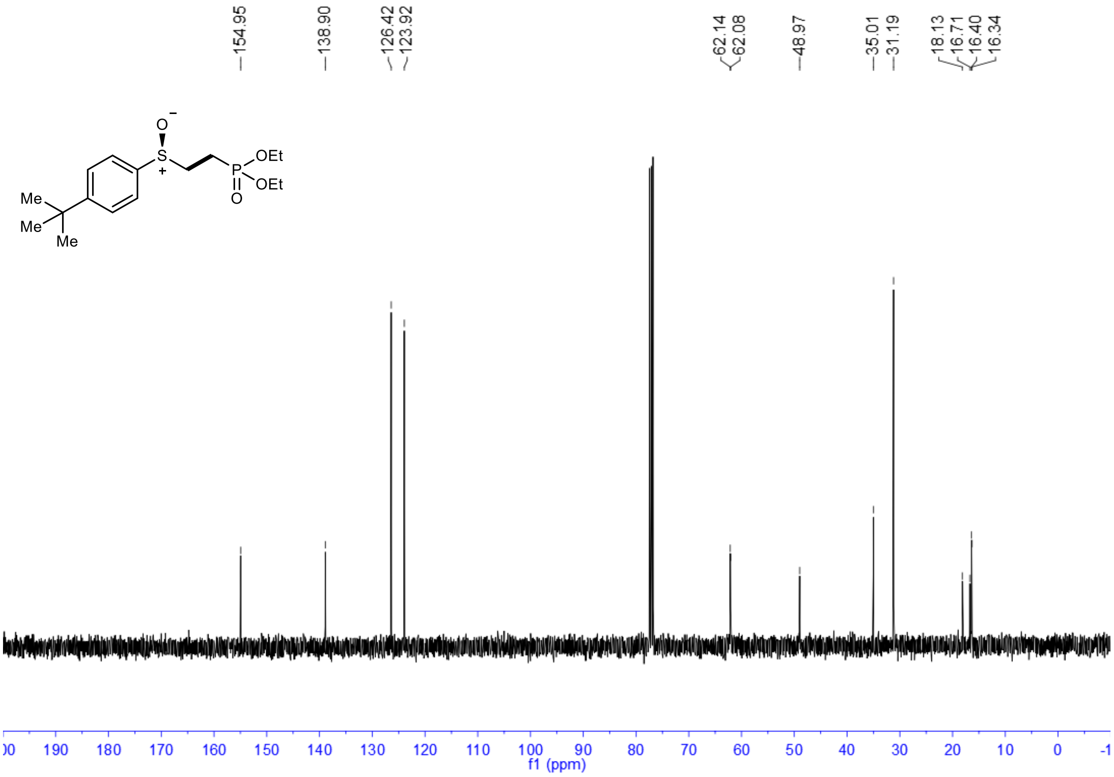

Supplementary Figure 378 | ^{31}P NMR (162 MHz, 298K, $CDCl_3$) of (*S*)-Diethyl (2-((4-(*tert*-butyl)phenyl)sulfinyl)ethyl)phosphonate (**90**)

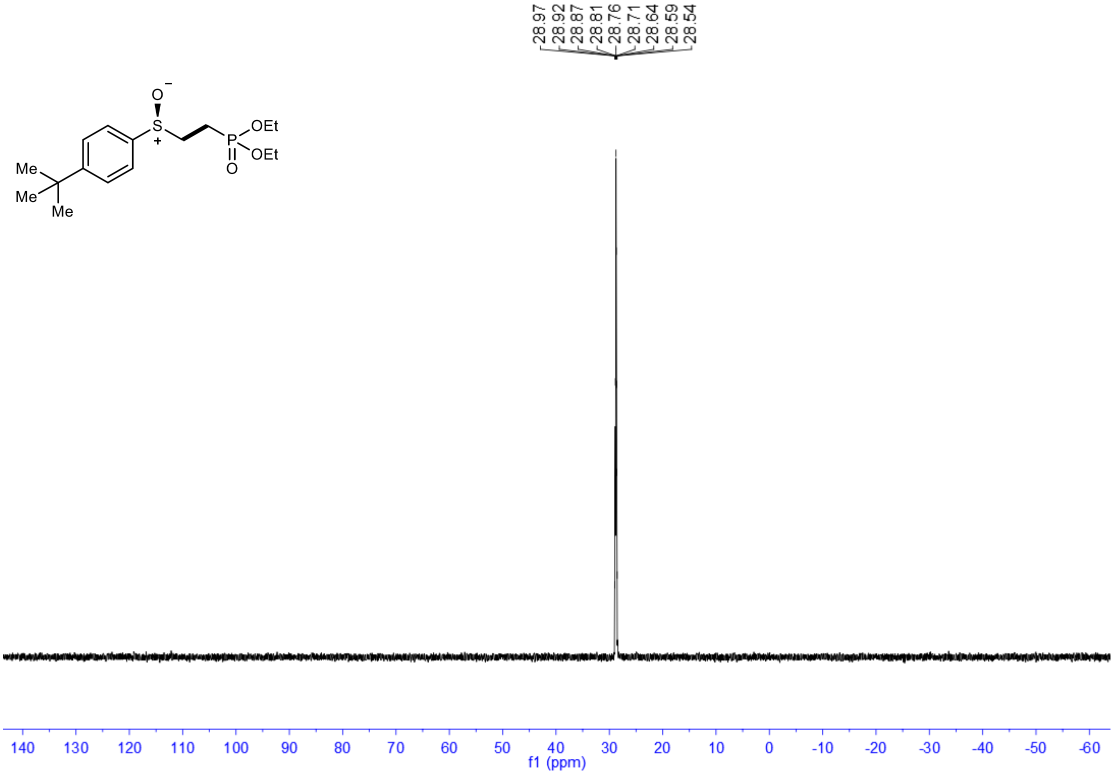

Supplementary Figure 379 | ^{1}H NMR (400 MHz, 298K, $CDCl_3$) of **(105)**

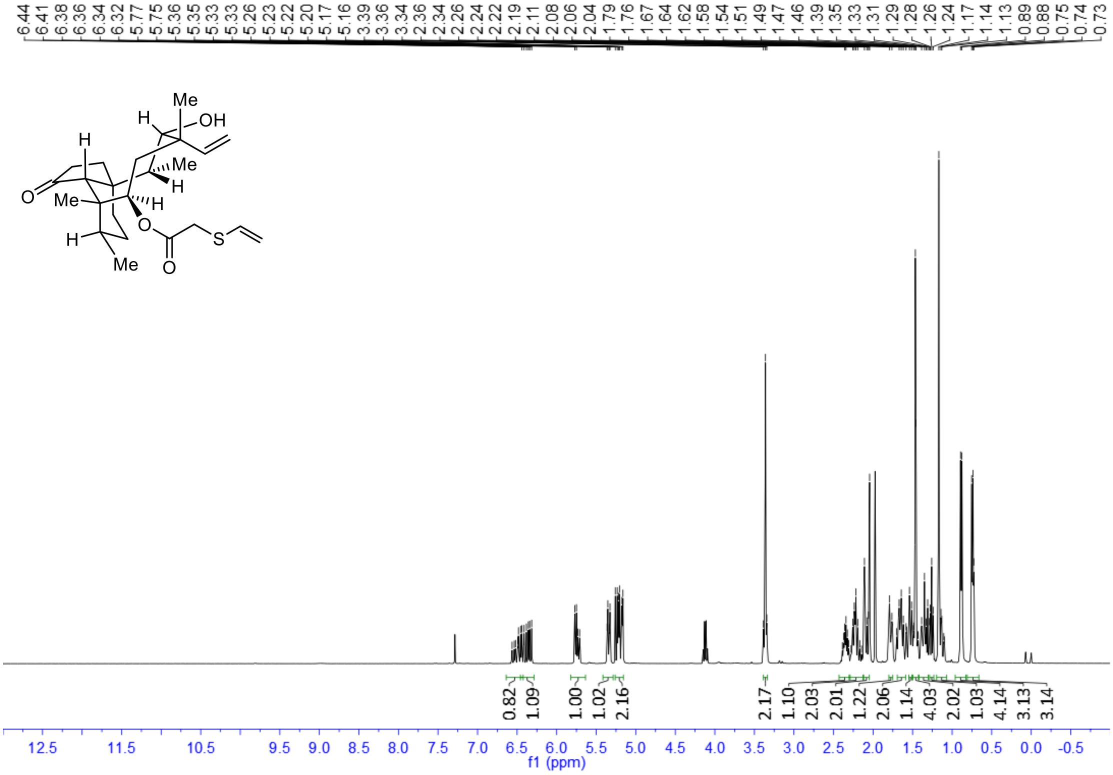

Supplementary Figure 380 | ^{13}C NMR (101 MHz, 298K, $CDCl_3$) of **(105)**

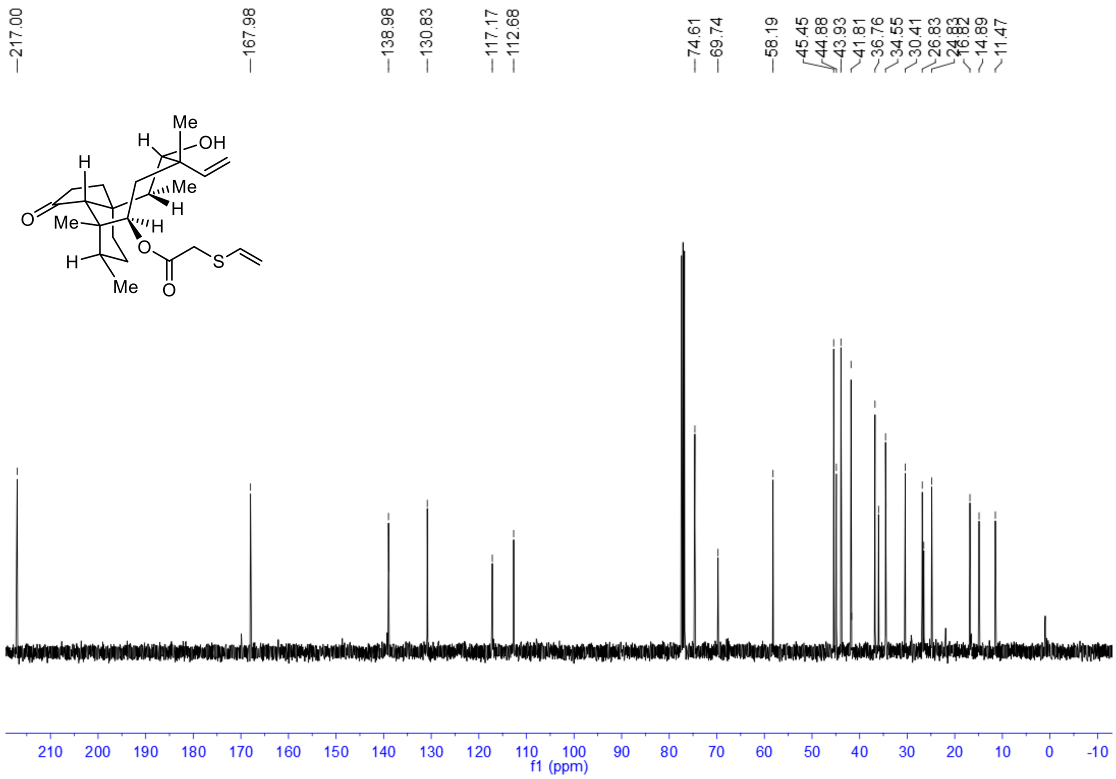

Supplementary Figure 381 | ^{1}H NMR (400 MHz, 298K, $CDCl_3$) of tiamulin **(106)**

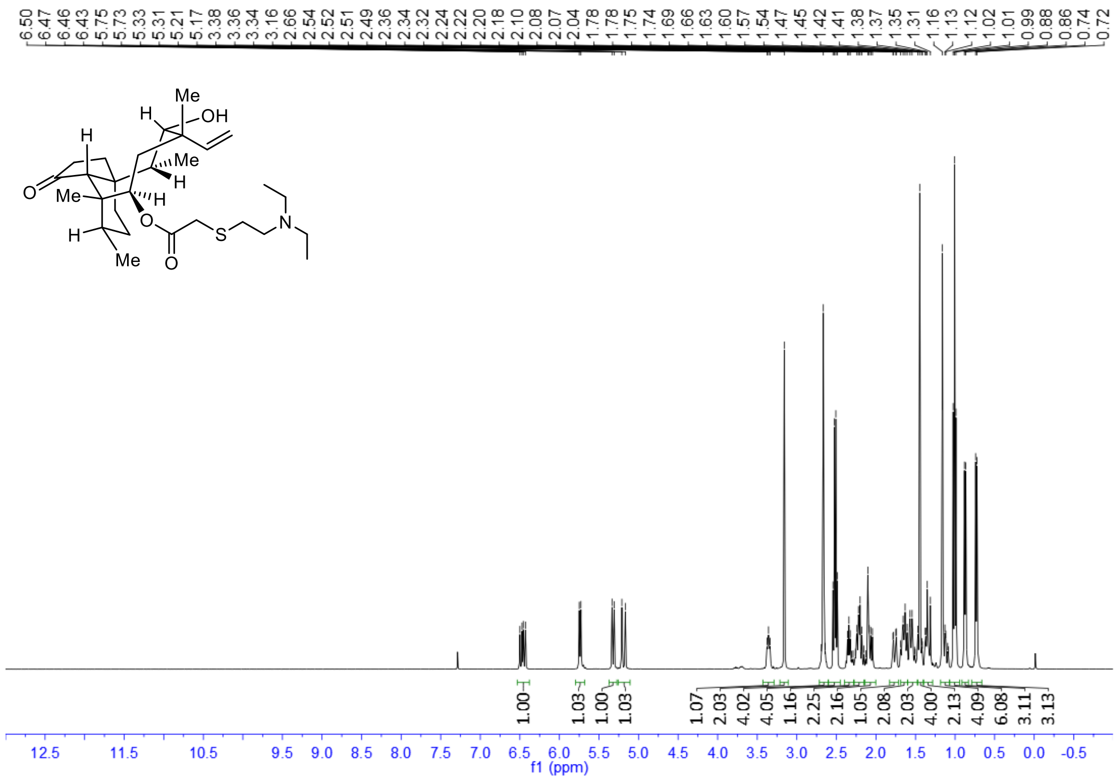

Supplementary Figure 382 | ^{13}C NMR (101 MHz, 298K, $CDCl_3$) of tiamulin **(106)**

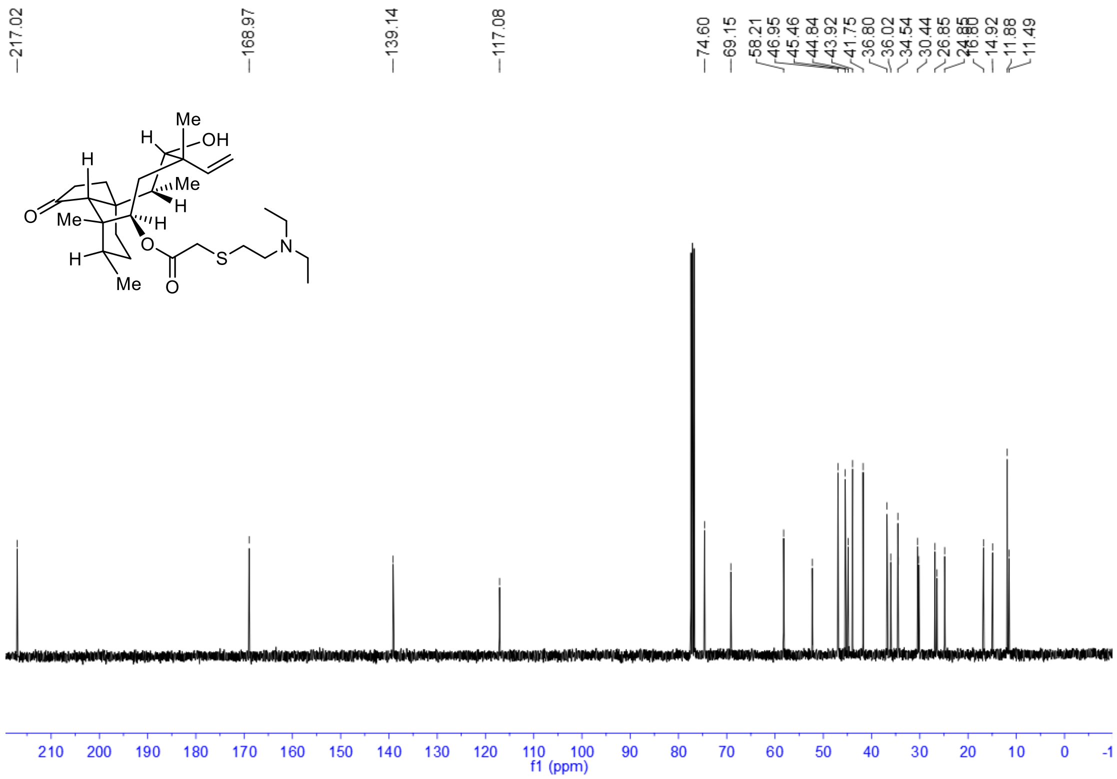

HPLC Datas

PDA Ch1 254nm

峰号	保留时间	面积	高度	浓度单位	标记	化合物名	面积%
1	6.334	14134104	961254	mg/L	M	RT:6.334	49.915
2	7.083	14182485	828309	mg/L	M	RT:7.083	50.085
总计		28316589	1789562				100.000

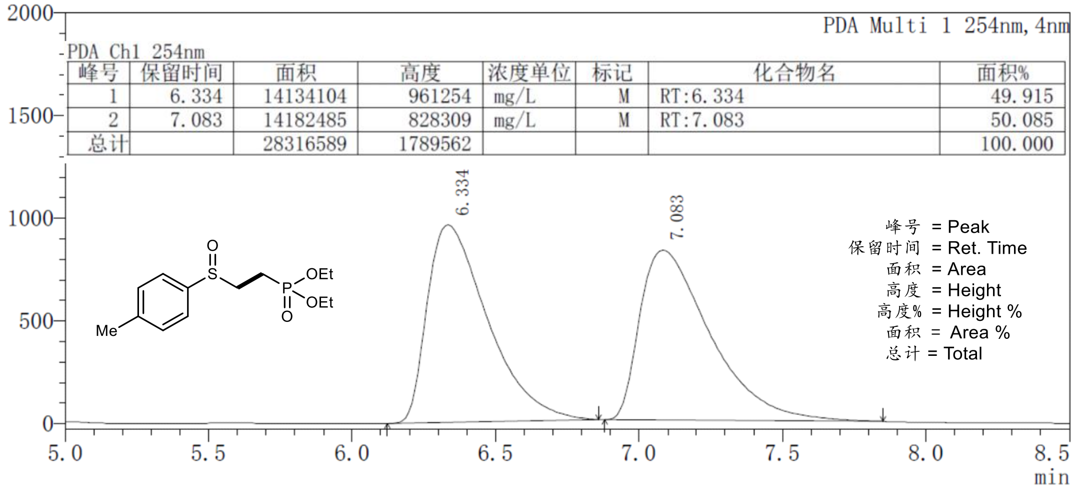

PDA Ch1 254nm

峰号	保留时间	面积	高度	浓度单位	标记	化合物名	面积%
1	6.493	1958392	127609	mg/L	M	RT:6.493	95.176
2	7.354	99255	6403	mg/L	M	RT:7.354	4.824
总计		2057648	134012				100.000

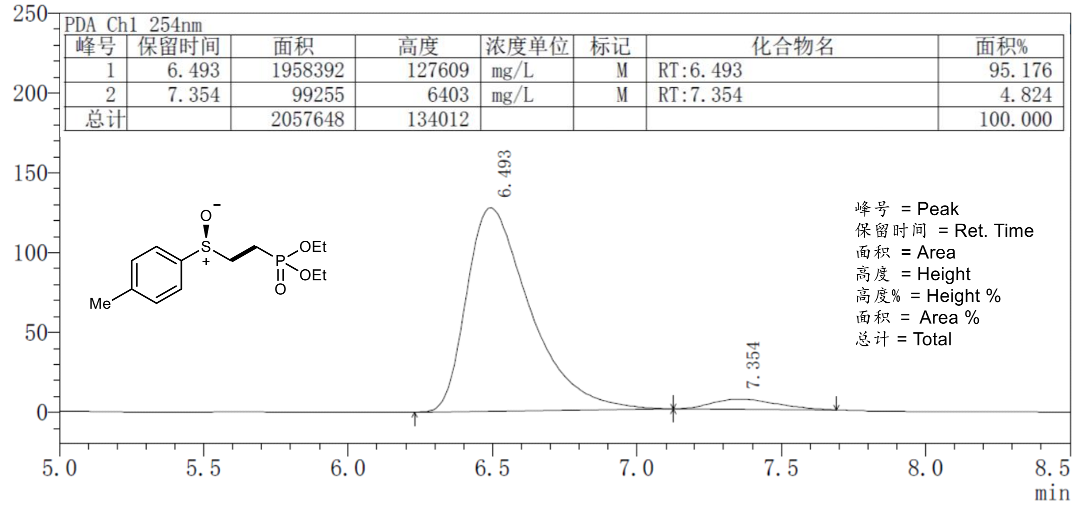

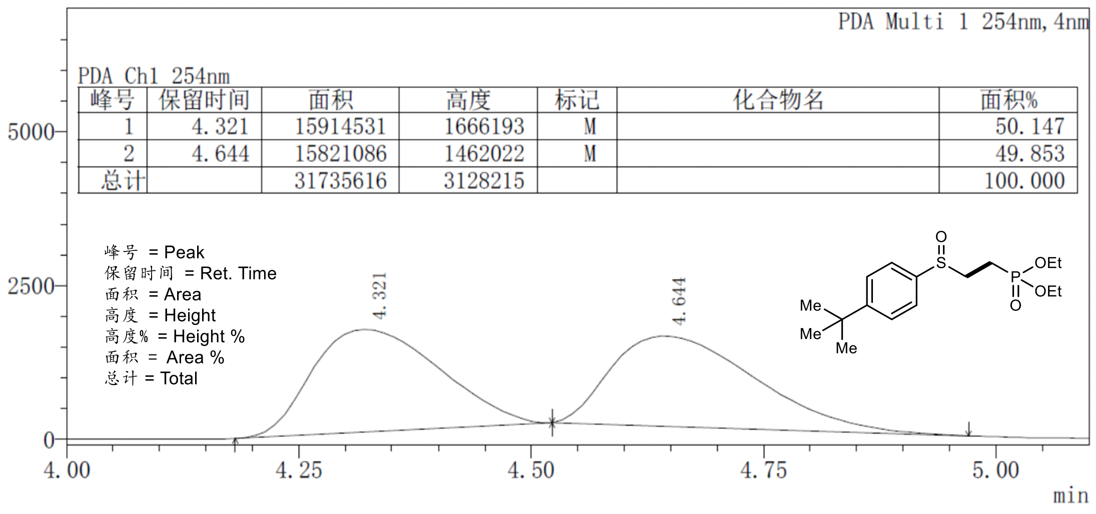

PDA Ch1 254nm

峰号	保留时间	面积	高度	标记	化合物名	面积%
1	4.321	15914531	1666193	M		50.147
2	4.644	15821086	1462022	M		49.853
总计		31735616	3128215			100.000

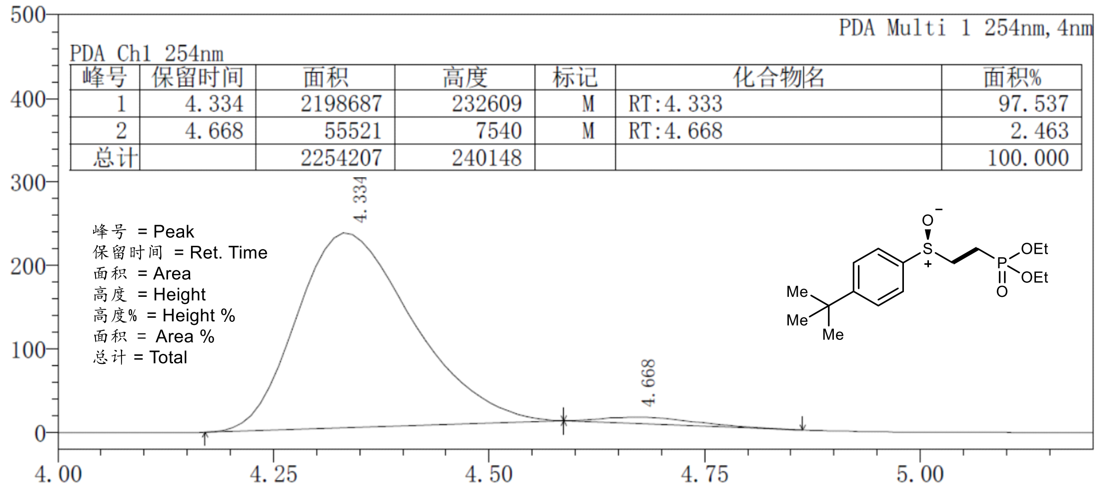

PDA Ch1 254nm

峰号	保留时间	面积	高度	标记	化合物名	面积%
1	4.334	2198687	232609	M	RT:4.333	97.537
2	4.668	55521	7540	M	RT:4.668	2.463
总计		2254207	240148			100.000

Supplementary Reference

1. https://encyclopedia.thefreedictionary.com/Acetylene.
2. Xie, D., Zhao, J. et al. Synthesis and application of novel multi-arm poly(carboxylic acid)s for glass-ionomer restoratives. *J Biomater Appl* **24**, 419−436 (2010).
3. Cornella, J. et al. Practical Ni-catalyzed aryl–alkyl cross-coupling of secondary redox-active esters. *J. Am. Chem. Soc.* **138**, 2174−2177 (2016).
4. Liu, Q. X. et al. Binaphthol bridged azacyclic dicarbine metal complex, preparation method and application as cross-coupling catalyst. *China Patent*, **CN** 104447700 A (2015).
5. Volante, R. P. A new highly efficient Method for the conversion of alcohols to thiolesters and thiols. *Tetrahedron Lett.* **22**, 3119−3122 (1981).
6. Han, C. C., Balakumar, R. Mild and efficient methods for the conversion of benzylic bromides to benzylic thiols. *Tetrahedron Lett.* **47**, 8255−8258 (2006).
7. Musacchio, A. J., Lainhart, B. C., Zhang, X., Naguib, S. G., Sherwood, T. C., Knowles, R. R. Catalytic intermolecular hydroaminations of unactivated olefins with secondary alkyl amines. *Science* **355**, 727−730 (2017).
8. Li, Y. G. et al. A bulky disulfoxide ligand for Pd-catalyzed oxidative allylic C-H amination with 2,2,2-trichloroethyl tosyl carbamate. *J. Org. Chem.* **82**, 4907−4917 (2017).
9. Sowell, J. W., Tang, Y. et al. Synthesis and cholinergic properties of bis[[(dimethylamino)methyl]furanyl] analogs of ranitidine. *J. Med. Chem.* **35**, 1102−110 (1992).
10. Ahmad S., Iqbal, J. Cobalt(II) chloride catalysed coupling of thiols and anhydrides: A new and efficient synthesis of thiol esters. *Tetrahedron Letters,* **27**, 3791−3794 (1986).
11. Teders, M., Henkel, C. et al. The energy-transfer-enabled biocompatible disulfide–ene reaction. *Nat. Chem.* **10**, 981−988 (2018).
12. Küppers, D. H. et al. Crown thioether complexes of AgI and CuI: the crystal structures of [{Ag_3L_3}AgL{AgL_2}]$(ClO_4)_4$ and [LCuI] (L = 1, 4, 7-Trithiacyclononane). *Angew. Chern. Int. Ed.* **26**, 575−576 (1987).
13. Wieghardt, K. et al. Weak Pd···S interactions in palladium(II) complexes with 1,4,7-trithiacyclononane as ligand. *Angew. Chern. Int. Ed.* **25**, 1101−1103 (1986).
14. Çiçek, B. et al. Synthesis, metal ion complexation and computational studies of thio oxocrown ethers. *Molecules* **16**, 8670−8683 (2011).
15. Chen, M. S. & White, M. C. A Sulfoxide-Promoted, Catalytic Method for the Regioselective Synthesis of Allylic Acetates from Monosubstituted Olefins via C−H Oxidation. *J. Am. Chem. Soc.* **126**, 1346−1347 (2004).
16. Zong, L. et al. Catalytic enantioselective alkylation of sulfenate anions to chiral heterocyclic sulfoxides using halogenated pentanidium salts. *Angew. Chem. Int. Ed.* **53**, 11849−11853 (2014).
17. Goodrow, M. H. et al. Synthesis of medium ring disulfides by titrimetry; an improvement on high dilution techniques. *Synthesis* **6**, 457−459 (1981).
18. Kim, H. et al. Visible-light-induced photocatalytic reductive transformations of organohalides. *Angew. Chern. Int. Ed.* **124**, 12469–12472 (2012).
19. Roberts, B. P. Polarity-reversal catalysis of hydrogen-atom abstraction reactions: concepts and applications in organic chemistry. *Chem. Soc. Rev.* **28**, 25–35 (1999).
20. Ye, J. H., Miao, M. et al. Visible light-driven and Iron-promoted thiocarboxylation of styrenes and acrylates with CO_2. *Angew. Chem. Int. Ed.* **56**, 15416−15420 (2017).

21. Qvortrup, K., Rankic, D. A. & MacMillan, D. W. C. A general strategy for organocatalytic activation of C−H bonds via photoredox catalysis: direct arylation of benzylic ethers. *J. Am. Chem. Soc.* **136**, 626−629 (2014).
